# Supplementary material for: Photoinduced Mn catalysis for efficient platform for C-heteroatom bond coupling of aryl halides
Source: Nat Commun. 2026 Mar 27;17:4509. doi: 10.1038/s41467-026-70925-y (PMC13187312; doi:10.1038/s41467-026-70925-y)
Supplement: Supplementary file 1 — Supplementary Information [file 41467_2026_70925_MOESM1_ESM.pdf]

Supplementary Information

Photoinduced Mn Catalysis for Efficient Platform for C-Heteroatom Bond Coupling of Aryl Halides

Geyang Song,[a#] Jiameng Song,[a#] Qi Li,[a] Xiaoli Shi,[a] Xinyi Liu,[a] Deng Pan,[b] Tengfei Kang,[a] Jianyang Dong,[a] Gang Li,[a] Huaming Sun,[a] Juan Fan,[a] Chao Wang[a] and Dong Xue[a,*]

[a] Key Laboratory of Applied Surface and Colloid Chemistry, Ministry of Education, and School of Chemistry and Chemical Engineering, Shaanxi Normal University, Xi'an, 710062, China.

[b] School of Chemistry and Materials Science, Hangzhou Institute for Advanced Study, University of Chinese Academy of Sciences, Hangzhou, China.

E-mail: xuedong_welcome@snnu.edu.cn

Contents

1. General Information. ...S1

2. Optimization of reaction condition. ...S3

3. General procedure for C-Heteroatom cross-coupling reaction. ...S12

4. Mechanistic investigations. ...S14

6. Analytical data of products. ...S24

7. Copies of ^{1}H NMR, ^{13}C NMR and ^{19}F NMR spectra of products ...S71

1. General Information.

Unless otherwise specified, the chemicals were obtained commercially and used without further purification. Analytical thin-layer chromatography (TLC) was conducted with TLC plates (Silica gel 60 F254, Qingdao Haiyang) and visualization on TLC was achieved by UV light. Flash column chromatography was performed on silica gel 200-300 mesh. ^{1}H NMR spectra were recorded on a Bruker Advance 400 MHz NMR spectrometer and reported in units of parts per million (ppm) relative to tetramethylsilane (δ 0 ppm), $CDCl_3$ (δ 7.26 ppm). Multiplicities are given as: brs (broad singlet), s (singlet), d (doublet), t (triplet), q (quartet), or m (multiplet). ^{13}C NMR spectra were recorded on a Bruker Advance 400 (100 MHz) NMR spectrometer and reported in ppm relative to tetramethylsilane (δ 0 ppm), $CDCl_3$ (δ 77.16 ppm). HRMS (APCI or ESI) were performed on a Fourier transform ion cyclotron resonance mass spectrometer (Maxis Ultimate 300hplc). UV-Vis absorption spectra were collected on a PerkinEImer Lambda 365 UV-VIS Spectrophotometer. Continuous-wave electron paramagnetic resonance (EPR) spectra was recorded at on a Bruker E-500 spectrometer. All reagents were obtained commercially and used without further purification. MeCN, DMF and dioxane were refluxed over CaH_2. Toluene and THF were refluxed over Na/benzophenone and distilled under an argon atmosphere. Solvents used for column chromatography were of technical grade and used after distillation. *d*-Mebpy = 4, 4'-dimethybipyridine. The purple LED lamp used in the experiment was assembled by ourselves (Figure S1) and the light-emitting angle of the lamp bead is 45 degrees. The LED lamp is assembled by connecting five components. The kit consists of three 3 W purple LED lamp beads, thermally conductive separation aluminum substrate, thermally conductive aluminum module, LED lamp bead switch and LED driver (XC-8W600-OS, http://www.xchly.com/en/produc tshow-388-389-683.html). The purple LED beads were purchased from Zhuhai Tianhui Electronics Co., Ltd. (TH-UV395T3WL-3535-60, http://www.tianhui-led.com/product/454-cn.html). The optical power was up to 320-340 mw at 0.5 cm axis distance detected by a Thorlabs' optical power and energy meter (PM 100D). The screening of light sources was performed on our self-made photocatalytic parallel reactor. We did not use band pass filters, and the specific wavelengths (390-395 nm) refer only to the max of irradiation. Furthermore, for all light sources, it only refers to the maximum value of the illumination.

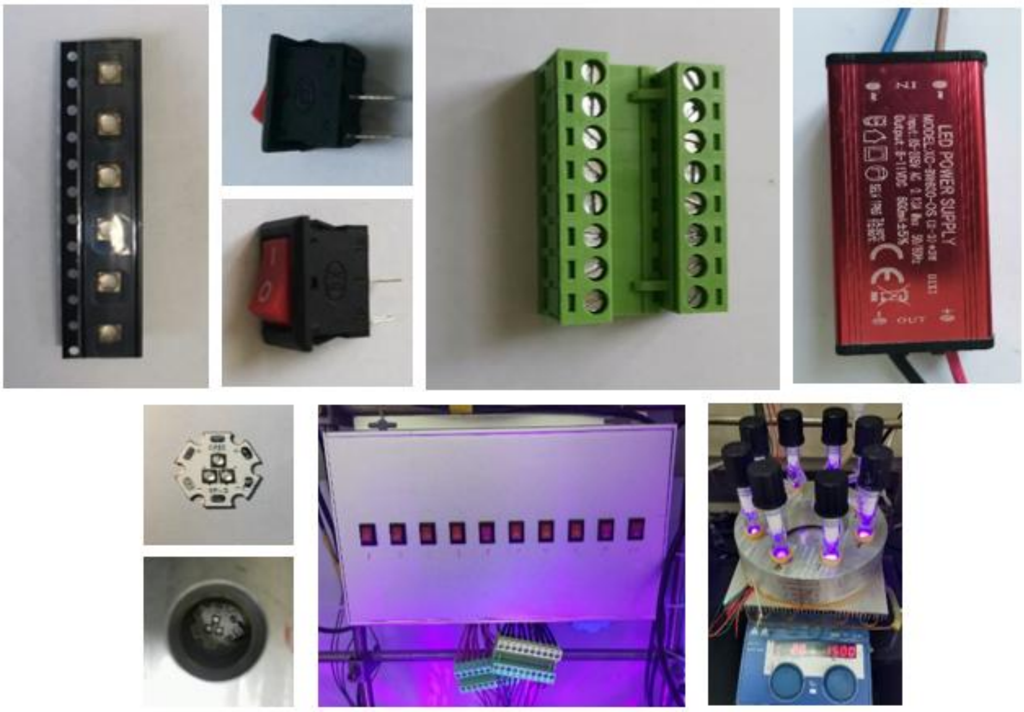

Figure S1. Light sources used in the reactions (3 x 3W purple LED lamp beads, LED driver, the thermal radiation of LEDs increased the temperature of reaction mixture as an average level at 85 °C approximately, and there are no external heating unit were equipped.)

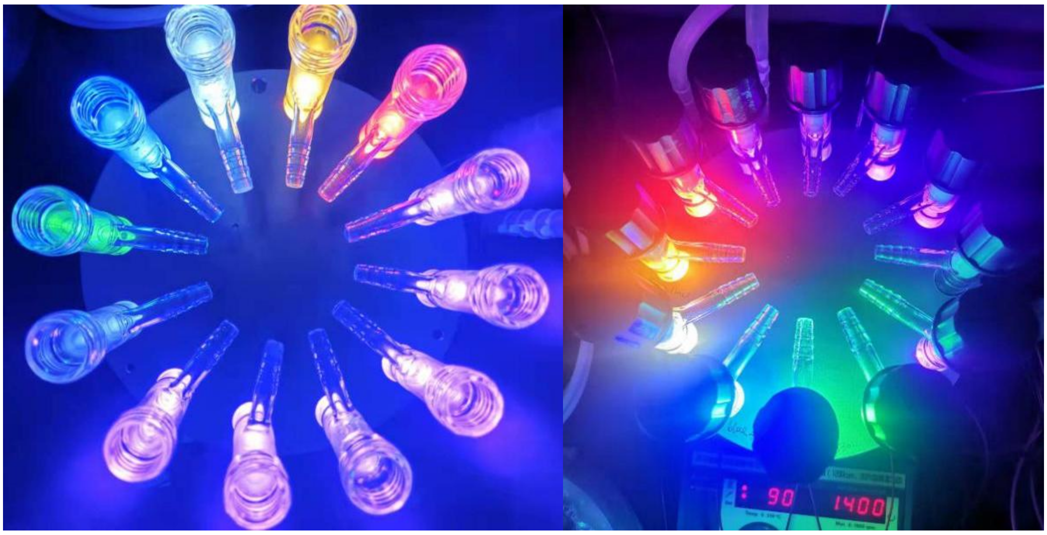

Figure S2. Our self-made photocatalytic parallel reactor. (3 x 3W LED lamp beads, LED driver).

2. Optimization of reaction condition.

Table S1. The effect of Mn(II) source

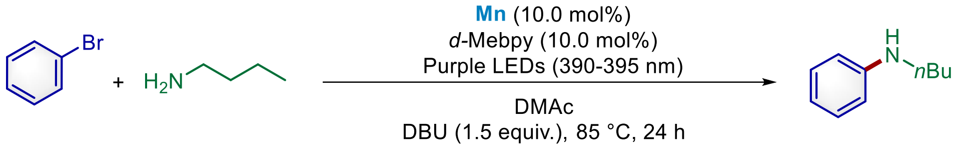

Entry	Mn source	Yield (%)[a]
1	$Mn(OAc)_2$	94
2	$Mn(acac)_2$	80
3	$MnBr_2$	76
4	$Mn(OTf)_2$	76
5	$MnCl_2$	66
6	$MnCO_3$	23
7	Mn(CO)	Trace
8	$Mn(NTf_2)_2$	Trace
9	$MnSO_4$	Trace
10	$Mn(NO)_3\cdot 6H_2O$	Trace
11	MnF_2	Trace

Reaction conditions: bromobenzene (0.2 mmol), *n*-butylamine (0.4 mmol), Mn source (10.0 mol%), *d*-Mebpy (10.0 mol%), DBU (1.5 equiv.), DMAc (2.0 mL), purple LEDs (390-395 nm), 85 °C, Ar, 24 h. [a] Yields determined by ^{1}H NMR, using 1,3-benzodioxole as internal standard.

Table S2. The effect of the $Mn(OAc)_2$ loading

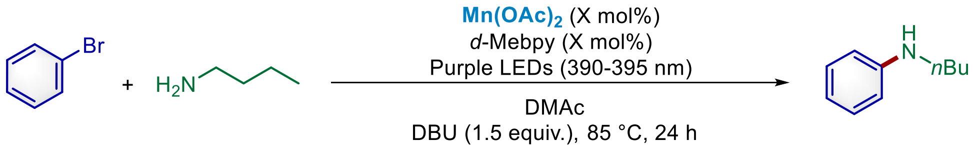

Entry	X	Yield (%)[a]
1	2	17
2	4	25
3	5	62
4	7	79
5	10	96

Reaction conditions: bromobenzene (0.2 mmol), *n*-butylamine (0.4 mmol), $Mn(OAc)_2$ (X mol%), *d*-Mebpy (X mol%), DBU (1.5 equiv.), DMAc (2.0 mL), purple LEDs (390-395 nm), 85 °C, Ar, 24 h. [a] Yields determined by ^{1}H NMR, using 1,3-benzodioxole as internal standard.

Table S3. The effect of light sources

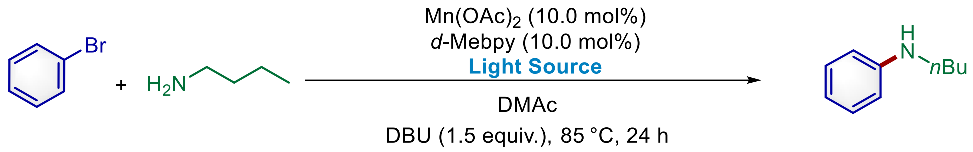

Entry	Light	Yield (%)^{a}
1	Blue LEDs (450 nm)	N.R.
2	Green LEDs (520 nm)	N.R.
3	White LEDs (6500K)	N.R.
4	Yellow LEDs (590 nm)	N.R.
5	Infrared LEDs (6500K)	N.R.
6	UV LEDs (365 nm)	25
7	395 nm LEDs	94

Reaction conditions: bromobenzene (0.2 mmol), *n*-butylamine (0.4 mmol), $Mn(OAc)_2$ (10.0 mol%), *d*-Mebpy (10.0 mol%), DBU (1.5 equiv.), DMAc (2.0 mL), light source, 85 °C, Ar, 24 h. [a] Yields determined by ^{1}H NMR, using 1,3-benzodioxole as internal standard.

Table S4. The effect of solvent

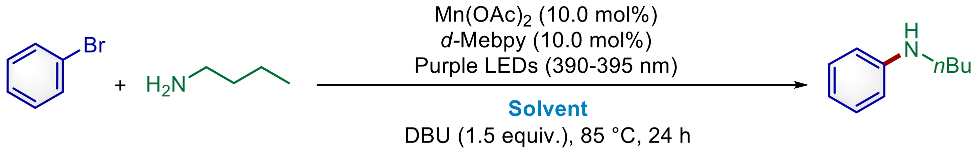

Entry	Solvent	Yield (%)^{a}
1	DMAc	94
2	NMP	90
3	DMF	82
4	DMSO	72
5	PhMe	Trace
6	1,4-dioxane	Trace
7	CH_3CN	Trace
8	THF	Trace

Reaction conditions: bromobenzene (0.2 mmol), *n*-butylamine (0.4 mmol), $Mn(OAc)_2$ (10.0 mol%), *d*-Mebpy (10.0 mol%), DBU (1.5 equiv.), solvent (2.0 mL), purple LEDs (390-395 nm), 85 °C, Ar, 24 h. [a] Yields determined by ^{1}H NMR, using 1,3-benzodioxole as internal standard.

Table S5. The effect of base

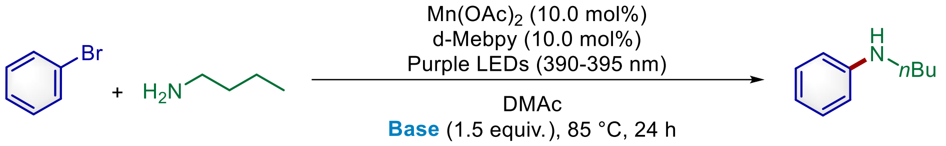

Entry	Base	Yield (%)^a
1	DBU	94
2	DIPEA	81
3	TBD	64
4	Et_3N	17
5	MTBD	10
6	DABCO	Trace
7	DBN	Trace
8	DMTHPM	Trace
9	TMG	Trace
10	^{t}BuTMG	Trace

Reaction conditions: bromobenzene (0.2 mmol), *n*-butylamine (0.4 mmol), Mn(OAc)$_2$ (10.0 mol%), *d*-Mebpy (10.0 mol%), base (1.5 equiv.), DMAc (2.0 mL), purple LEDs (390-395 nm), 85 °C, Ar, 24 h. [a] Yields determined by ^{1}H NMR, using 1,3-benzodioxole as internal standard.

Table S6. The effect of the DBU loading

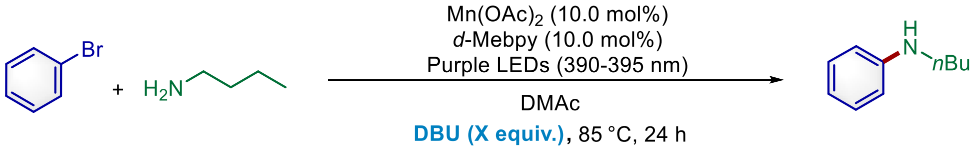

Entry	X	Yield (%)^a
1	0.5	56
2	1.0	78
3	1.5	95
4	2.0	96
5	2.5	90
6	3.0	90
7	3.5	90

Reaction conditions: bromobenzene (0.2 mmol), *n*-butylamine (0.4 mmol), Mn(OAc)$_2$ (10.0 mol%), *d*-Mebpy (10.0 mol%), DBU (X equiv.), DMAc (2.0 mL), purple LEDs (390-395 nm), 85 °C, Ar, 24 h. [a] Yields determined by ^{1}H NMR, using 1,3-benzodioxole as internal standard.

Table S7. The effect of ligand

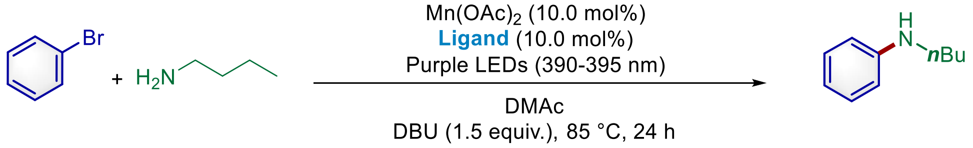

Ligand	Yield (%)[a]	Ligand	Yield (%)[a]
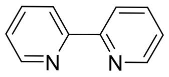	19	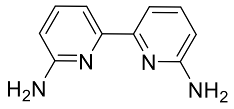	Trace
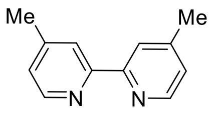	94	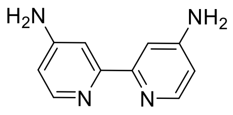	Trace
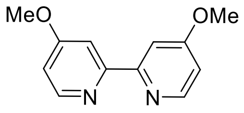	73	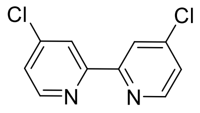	Trace
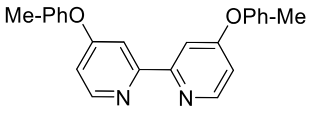	80	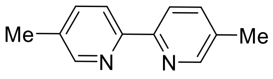	29
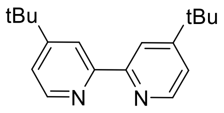	86	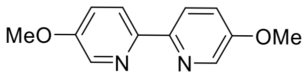	33
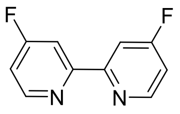	Trace	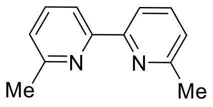	Trace
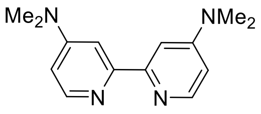	Trace	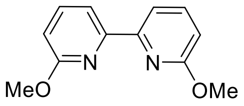	Trace

Reaction conditions: bromobenzene (0.2 mmol), *n*-butylamine (0.4 mmol), $Mn(OAc)_2$ (10.0 mol %), ligand (10.0 mol%), DBU (1.5 equiv.), DMAc (2.0 mL), purple LEDs (390-395 nm), 85 ºC, Ar, 24 h. [a] Yields determined by ^{1}H NMR, using 1,3-benzodioxole as internal standard.

Table S8. ICP-Ms detects trace metal content in $Mn(OAc)_2$

Entry	Metal type	ppb	Entry	Metal type	ppb
1	Ti	3.915	10	Sn	0.027
2	Cu^{63}	1.318	11	Sb	0.010
3	Cu^{65}	1.451	12	La	0.005
4	Ga	3.032	13	Co	0.783
5	As	0.061	14	Pt	0.001
6	Pd	0.013	15	Tl	0.018
7	Ag	0.613	16	Pb	0.788
8	Cd	0.010	17	Bi	0.115
9	In	0.003			

Table S9. The effect of Pd source

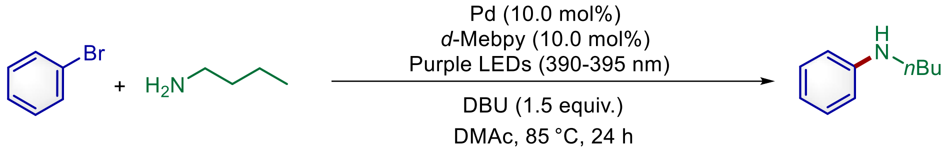

Entry	Pd Source	Yield (%)[a]
1	$Pd(OAc)_2$	N.R.
2	$PdCl_2$	N.R.
3	$PdBr_2$	N.R.
4	$Pd(acac)_2$	N.R.
5	$Pd(cod)Cl_2$	N.R.
6	$Pd(dppf)Cl_2$	N.R.
7	$Pd(dippp)Cl_2$	N.R.
8	$Pd(dtbpf)Cl_2$	N.R.

Reaction conditions: bromobenzene (0.2 mmol), *n*-butylamine (0.4 mmol), Pd (10.0 mol%), *d*-Mebpy (10.0 mol%), DBU (1.5 equiv.), DMAc (2.0 mL), purple LEDs (390-395 nm), 85 °C, Ar, 24 h. [a] Yields determined by ^{1}H NMR, using 1,3-benzodioxole as internal standard.

Table S10. The effect of the amount of Pd loading

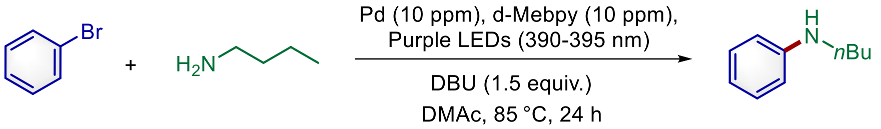

Entry	Pd Source	Yield (%)[a]
1	$Pd(OAc)_2$	N.R.
2	$PdCl_2$	N.R.
3	$PdBr_2$	N.R.
4	$Pd(acac)_2$	N.R.
5	$Pd(cod)Cl_2$	N.R.
6	$Pd(dppf)Cl_2$	N.R.
7	$Pd(dippp)Cl_2$	N.R.
8	$Pd(dtbpf)Cl_2$	N.R.

Reaction conditions: bromobenzene (0.2 mmol), *n*-butylamine (0.4 mmol), Pd (10 ppm), *d*-Mebpy (10 ppm), DBU (1.5 equiv.), DMAc (2.0 mL), purple LEDs (390-395 nm), 85 °C, Ar, 24 h. [a] Yields determined by ^{1}H NMR, using 1,3-benzodioxole as internal standard.

Table S11. The effect of Cu source

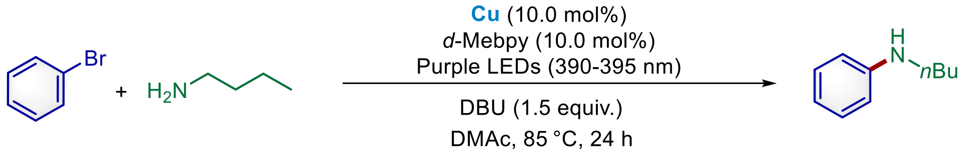

Entry	Cu Source	Yield (%)[a]
1	Cu_2O	N.R.
2	CuCl	N.R.
3	CuBr	N.R.
4	CuI	8
5	$CuBr_2$	N.R.
6	CuI_2	N.R.
7	$CuCl_2$	N.R.

Reaction conditions: bromobenzene (0.2 mmol), *n*-butylamine (0.4 mmol), Cu source (10.0 mol%), *d*-Mebpy (10.0 mol%), DBU (1.5 equiv.), DMAc (2.0 mL), purple LEDs (390-395 nm), 85 °C, Ar, 24 h. [a] Yields determined by ^{1}H NMR, using 1,3-benzodioxole as internal standard.

Table S12. The effect of the amount of Cu loading

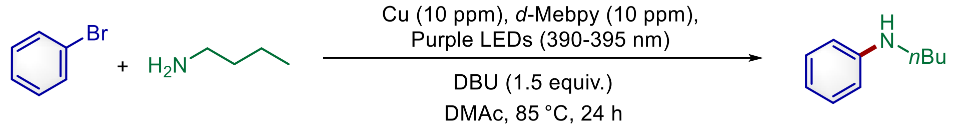

Entry	Cu Source	Yield (%)[a]
1	Cu_2O	N.R.
2	CuCl	N.R.
3	CuBr	N.R.
4	CuI	N.R.
5	$CuBr_2$	N.R.
6	CuI_2	N.R.
7	$CuCl_2$	N.R.

Reaction conditions: bromobenzene (0.2 mmol), *n*-butylamine (0.4 mmol), Cu (10 ppm), *d*-Mebpy (10 ppm), DBU (1.5 equiv.), DMAc (2.0 mL), purple LEDs (390-395 nm), 85 °C, Ar, 24 h. [a] Yields determined by ^{1}H NMR, using 1,3-benzodioxole as internal standard.

Table S13. The effect of Mn source from different supplier

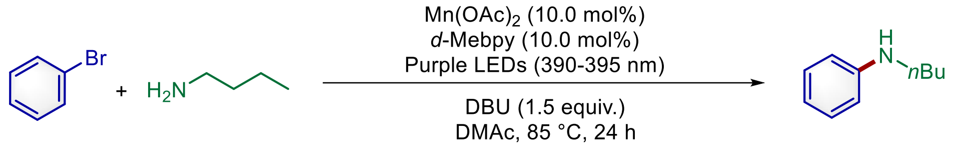

Entry	Mn Source	Yield (%)[a]
1	$Mn(OAc)_2$, Anergy Chemical, (99%)	81
2	$Mn(OAc)_2$, Alfa Chemistry, (99.99%)	87
3	$Mn(OAc)_2$, Sigma-Aldrich, (99.99%)	95
4	$Mn(OAc)_2$, Thermo Scientific, (99.99%)	94
5	$Mn(OAc)_2$, TCL, (99.99%)	93

Reaction conditions: bromobenzene (0.2 mmol), *n*-butylamine (0.4 mmol), $Mn(OAc)_2$ (10.0 mol%), *d*-Mebpy (10.0 mol%), DBU (1.5 equiv.), DMAc (2.0 mL), purple LEDs (390-395 nm), 85 °C, Ar, 24 h. [a] Yields determined by ^{1}H NMR, using 1,3-benzodioxole as internal standard.

Table S14. The effect of reaction time

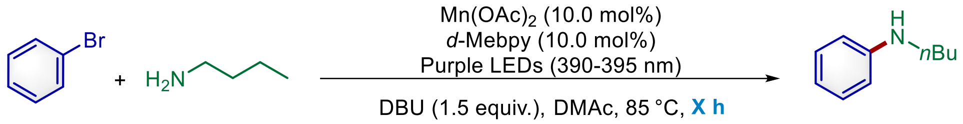

Entry	X	Yield (%)[a]
1	5	33
2	10	45
3	15	66
4	20	92
5	24	96

Reaction conditions: bromobenzene (0.2 mmol), *n*-butylamine (0.4 mmol), $Mn(OAc)_2$ (10.0 mol%), *d*-Mebpy (10.0 mol%), DBU (1.5 equiv.), DMAc (2.0 mL), purple LEDs (390-395 nm), 85 °C, Ar, X h. [a] Yields determined by ^{1}H NMR, using 1,3-benzodioxole as internal standard.

Table S15. The effect of concentration

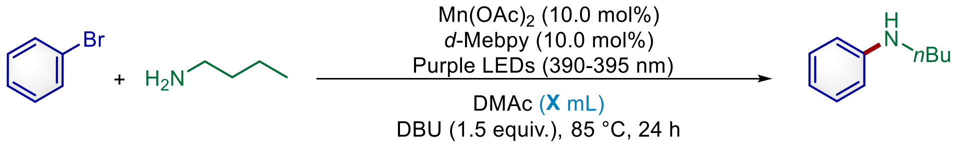

Entry	X	Yield (%)[a]
1	0.5	21
2	1	33
3	1.5	69
4	2	95
5	2.5	84
6	3	63
7	3.5	41
8	3.5	26

Reaction conditions: bromobenzene (0.2 mmol), *n*-butylamine (0.4 mmol), $Mn(OAc)_2$ (10.0 mol%), *d*-Mebpy (10.0 mol%), DBU (1.5 equiv.), DMAc (X mL), purple LEDs (390-395 nm), 85 °C, Ar, 24 h. [a] Yields determined by ^{1}H NMR, using 1,3-benzodioxole as internal standard.

Table S16. Control experiment

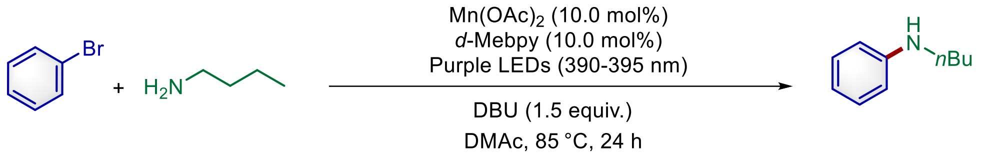

Entry	Condition	Yield (%)[a]
1	standard conditions	95
2	standard conditions, no light, heating to 120 °C	n.r.
3	standard conditions, no light, heating to 70 °C	n.r.
4	standard conditions, no light	n.r.
5	standard conditions, no Mn catalyst	n.r.
6	standard conditions, no base, no Mn catalyst	n.r.
7	standard conditions, no base	n.r.
8	standard conditions, no ligand	n.r.
9	standard conditions, Air	n.r.

Reaction conditions: bromobenzene (0.2 mmol), *n*-butylamine (0.4 mmol), $Mn(OAc)_2$ (10.0 mol%), *d*-Mebpy (10.0 mol%), DBU (1.5 equiv.), DMAc (2.0 mL), purple LEDs (390-395 nm), 85 °C, Ar, 24 h. [a] Yields determined by ^{1}H NMR, using 1,3-benzodioxole as internal standard.

Table S17. The effect of addtive

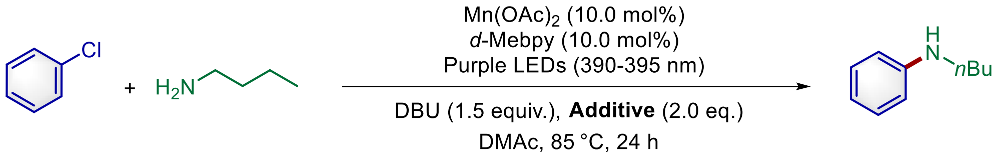

Entry	Additive	Yield (%)[a]
1	KBr	22
2	KI	37
3	NaI	52
4	NaBr	19
5	TBAB	76
6	TBAI	87
7	TBACl	11

Reaction conditions: chlorobenzene (0.2 mmol), *n*-butylamine (0.4 mmol), $Mn(OAc)_2$ (10.0 mol%), *d*-Mebpy (10.0 mol%), DBU (1.5 equiv.), DMAc (2.0 mL), additive (2.0 eq.), purple LEDs (390-395 nm), 85 °C, Ar, 24 h. [a] Yields determined by ^{1}H NMR, using 1,3-benzodioxole as internal standard.

Table S18. The effect of the amount of TBAI

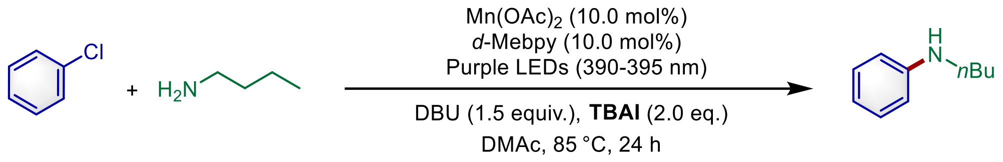

Entry	TBAI	Yield (%)[a]
1	0.5	28
2	1.0	57
3	1.5	77
4	2.0	87
5	2.5	88
6	3.0	89

Reaction conditions: chlorobenzene (0.2 mmol), *n*-butylamine (0.4 mmol), $Mn(OAc)_2$ (10.0 mol%), *d*-Mebpy (10.0 mol%), DBU (1.5 equiv.), DMAc (2.0 mL), TBAI (x eq.), purple LEDs (390-395 nm), 85 °C, Ar, 24 h. [a] Yields determined by ^{1}H NMR, using 1,3-benzodioxole as internal standard.

3. General procedure for C-Heteroatom cross-coupling reaction.

3.1 Standard procedure for C-Heteroatom of aryl halide

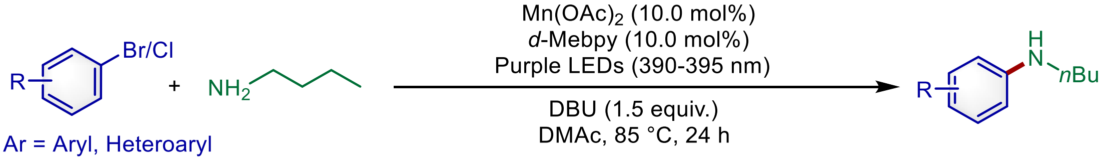

To an oven-dried 10 mL of storage tube were added $Mn(OAc)_2$ (10.0 mol%), *d*-Mebpy (4,4'-dimethyl-2,2'-bipyridine) (10.0 mol%), and 2 mL of DMAc with a magnetic stir bar under argon atmosphere. The mixture was evacuated and backfilled with Argon for 3 times. Then the aryl halide (0.2 mmol), *n*-butylamine (0.4 mmol) and DBU (1.5 equiv., 0.3 mmol) or TBAI (2.0 eq.) were added. The tube was sealed with the Teflon screw valve. The reaction mixture was then irradiated with 9 W purple LEDs (0.5 cm away from the tube, optical power: 320-340 mW/cm^2) at 85 °C. After the reaction was completed, the mixture was diluted with ethyl acetate and cooled to room temperature. The organic phases were washed with saturated ammonium chloride (3 × 10 mL), dried over anhydrous sodium sulfate, and concentrated under reduced pressure. The residue was purified by flash column chromatography using petroleum ether and

ethyl acetate as eluent to afford coupling products. (**Noted**: Mn salts are purchased on Strem, and the purity is 99.99%).

3.2 Standard procedure for C-Heteroatom of nucleophile

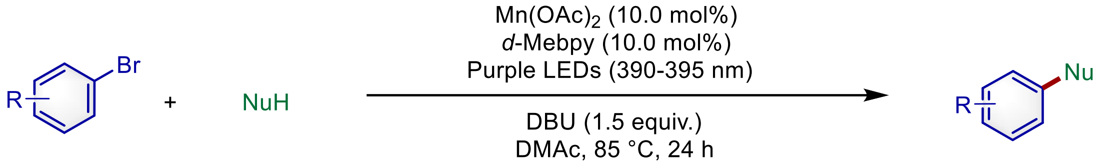

To an oven-dried 10 mL of storage tube were added $Mn(OAc)_2$ (10.0 mol%), *d*-Mebpy (4,4'-dimethyl-2,2'-bipyridine) (10.0 mol%), and 2 mL of DMAc with a magnetic stir bar under argon atmosphere. The mixture was evacuated and backfilled with Argon for 3 times. Then the aryl halide (0.2 mmol), NuH (0.4 mmol), and DBU (1.5 equiv., 0.3 mmol) were added. The tube was sealed with the Teflon screw valve. The reaction mixture was then irradiated with 9 W purple LEDs (0.5 cm away from the tube, optical power: 320-340 mW/cm^2) at 85 °C. After the reaction was completed, the mixture was diluted with ethyl acetate and cooled to room temperature. The organic phases were washed with saturated ammonium chloride (3 × 10 mL), dried over anhydrous sodium sulfate, and concentrated under reduced pressure. The residue was purified by flash column chromatography using petroleum ether and ethyl acetate as eluent to afford coupling products.

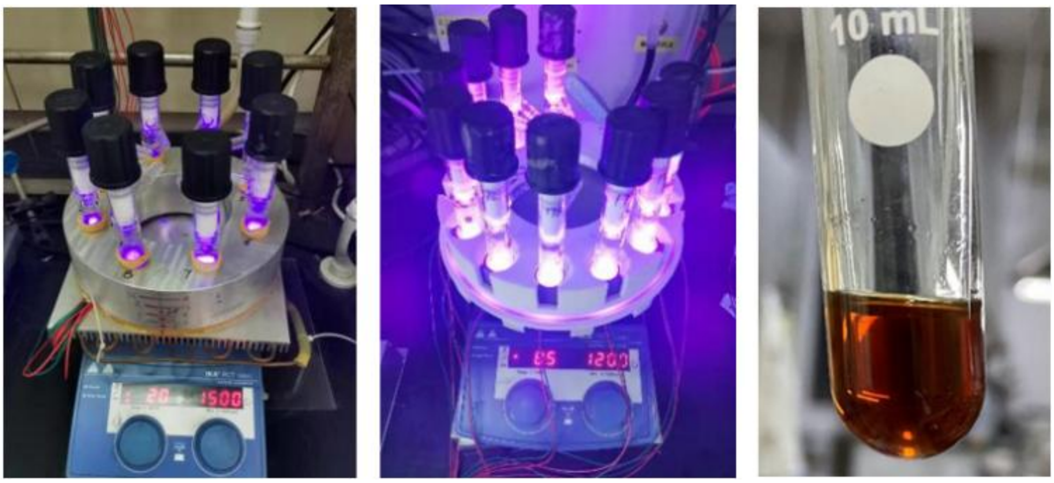

Figure S3. Pictures of the reaction setup.

3.3 Unsuccessful examples

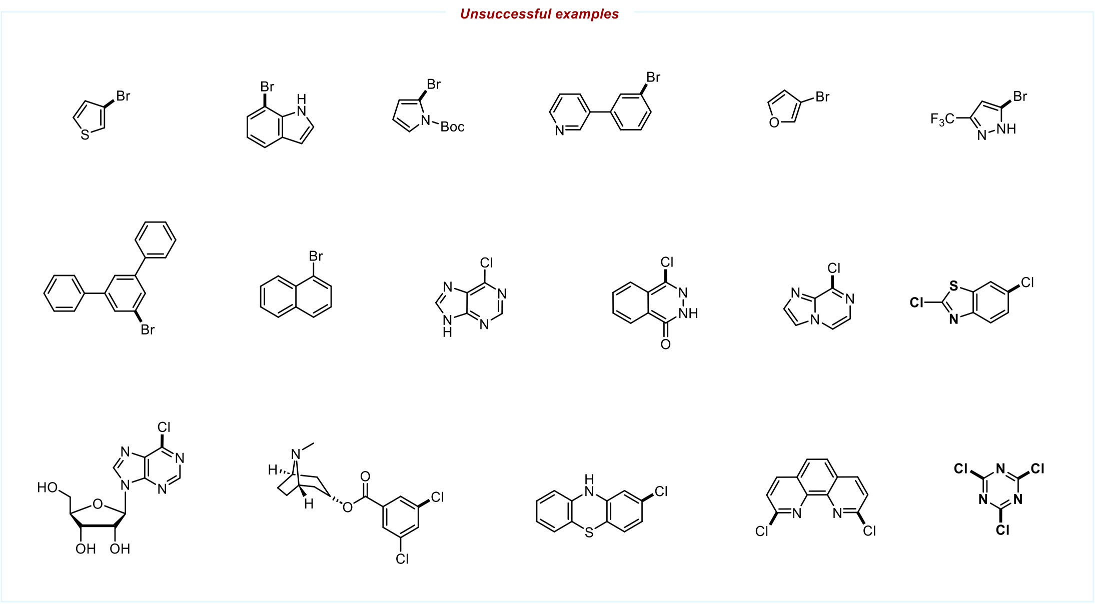

4. Mechanistic investigations.

4.1 UV-Vis studies

Linear absorption spectra were collected on a PerkinElmer Lambda 365 UV-VIS Spectrophotometer at room temperature. All reagents were dispensed in stock solutions and prepared volumetrically.

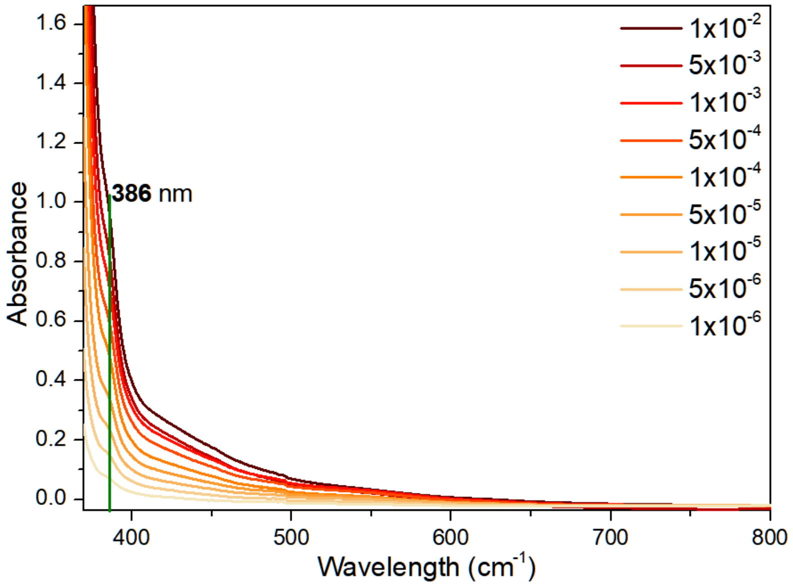

Figure S4. Absorption spectrum of Mn complex **174** (1 mm pathlength quartz cuvette) in DMAc.

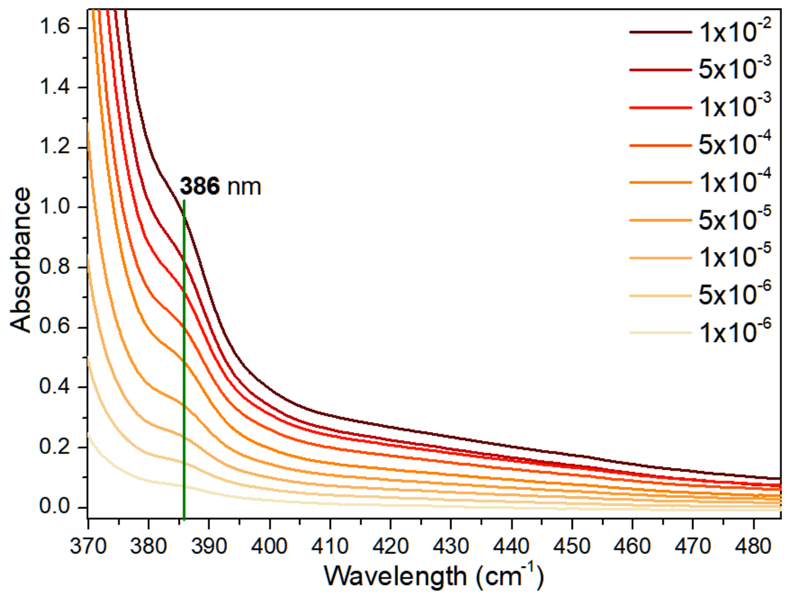

Figure S5. Absorption spectrum of Mn complex **174** (1 mm pathlength quartz cuvette) in DMAc.

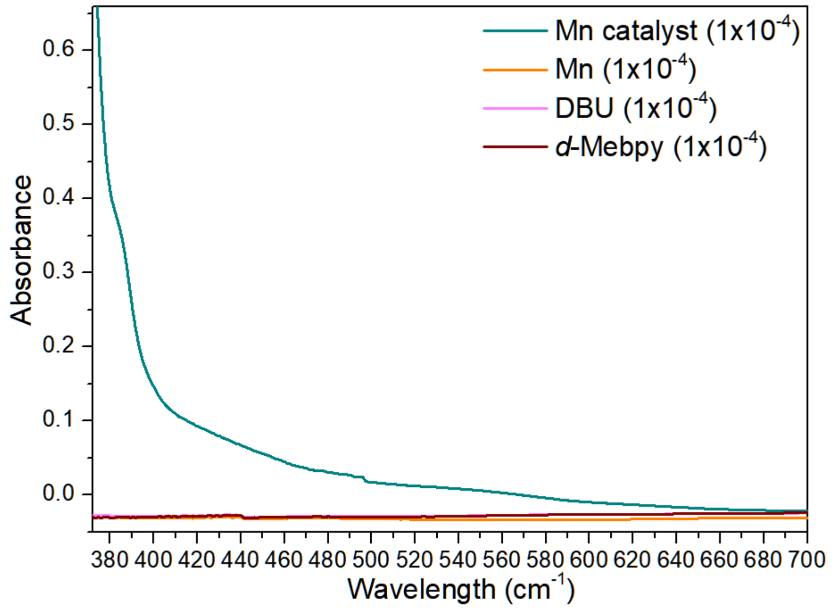

Figure S6. Absorption spectrum of all substances (1.0×10^{-4} M;1 mm pathlength quartz cuvette) in DMAc.

4.2 The EPR experiment

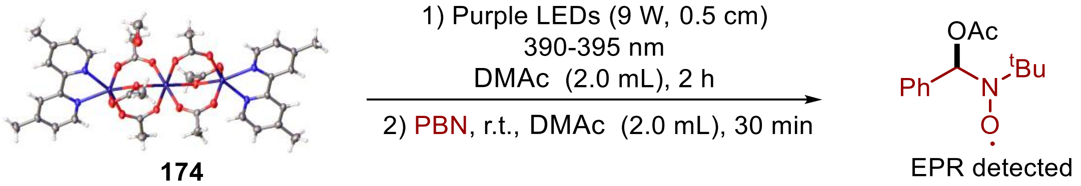

In a nitrogen-filled glove box, a magnetic stir bar, Mn complex **174** (0.01 mmol) and 2 mL of DMAc were placed into an oven-dried 10 mL storage tube with a high vacuum valve. The tube was sealed with the Teflon screw valve and removed from the glovebox. The reaction solution was then irradiated with purple LED lamps for 2 h at 85 °C. Then the tube was moved back to the glove box and *N*-*tert*-butyl-*α*-phenylnitrone (PBN, 0.05 mmol) was added. The tube was resealed with the Teflon screw valve and removed from the glove box and then stirred for 60 min at room temperature. The mixture was transferred to an oven-dried EPR tubes which were sealed with a rubber cap. EPR spectra was acquired at room temperature. The characteristic EPR signals were detected, indicating the formation of spin adducts (Figure S7).

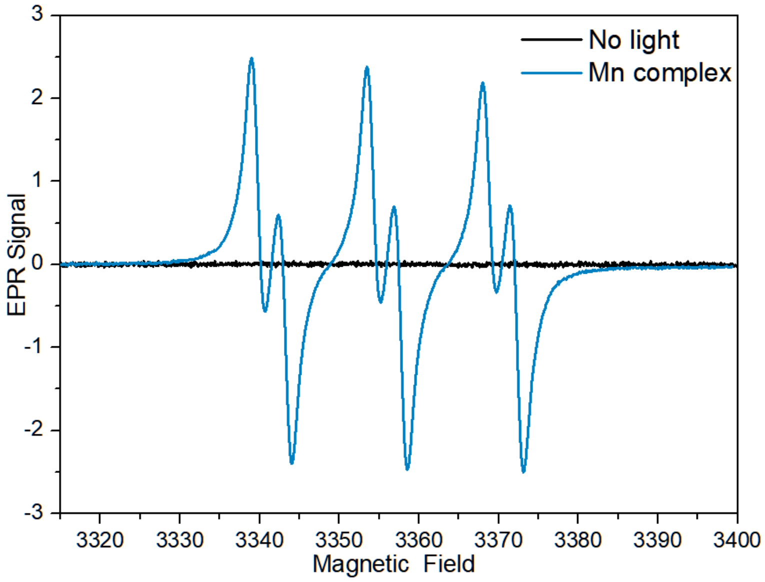

Figure S7. EPR spectrum of spin adduct (g = 2.006, a*N* = 14.56 G, a*H* = 2.79 G).

4.3 The cross-coupling reaction of aryl halide catalyzed by Mn complex 174

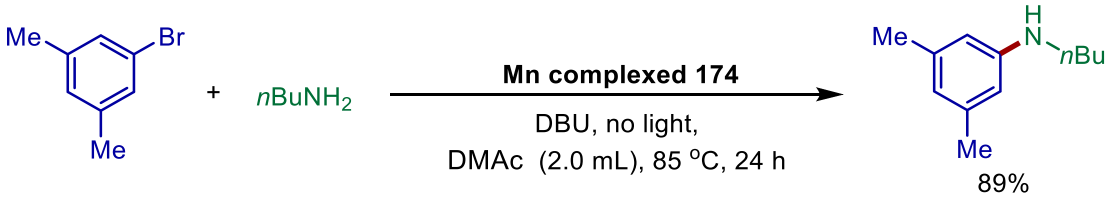

In a nitrogen-filled glove box, a magnetic stir bar, Mn complex **174** (5.0 mol%), and 2 mL of DMAc with a magnetic stir bar under argon atmosphere. The mixture was evacuated and backfilled with Argon for 3 times. Then the 3,5-dimethylbromobenzene (0.2 mmol), *n*-butylamine (0.4 mmol), and DBU (1.5 equiv., 0.3 mmol) were added in succession. The tube was sealed with the Teflon screw valve. The reaction mixture was then irradiated with 9 W purple LEDs (0.5 cm away from the tube, optical power: 320-340 mW/cm^2) at 85 °C. After cooling to room temperature, 1,3-benzodioxole (0.2 mmol) was added to the reaction solution.

The resulting mixture was diluted with 5 mL $CHCl_3$, the solvent was removed under reduced pressure and the residue was analyzed by ^{1}H NMR to give the yield of the reaction.

4.4 The cross-coupling reaction of aryl halide catalyzed by *in situ* generated Mn(I) complex via light irradiation

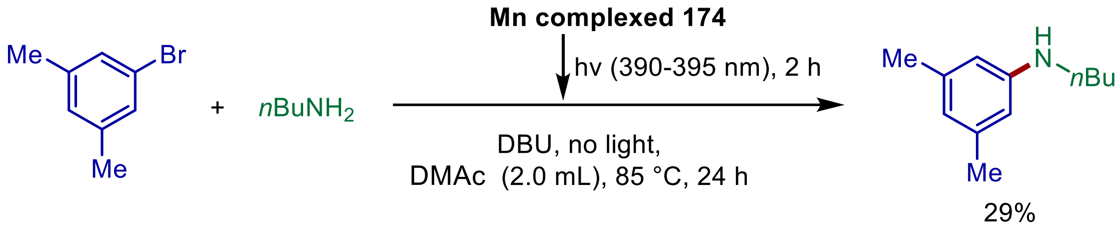

In a nitrogen-filled glove box, a magnetic stir bar, Mn complex **174** (10.0 mol%), DBU (1.5 equiv., 0.3 mmol) and 2 mL of DMAc were placed into an oven-dried 10 mL storage tube with a high vacuum valve. The tube was sealed with a Teflon screw valve and removed from the glovebox. The reaction solution was then irradiated with purple LED lamps for 2 h at 85 °C. Then tube was moved back to glove box and 3,5-dimethylbromobenzene (0.2 mmol), *n*-butylamine (0.4 mmol) were added in succession. The tube was resealed with a Teflon screw valve and removed from the glove box. The tube was wrapped with aluminium foil and then stirred for 24 h at 85 °C. After cooling to room temperature, 1,3-benzodioxole (0.2 mmol) was added to the reaction solution, the mixture was diluted with ethyl acetate. The organic phases were washed with saturated brine (3 × 10 mL), dried over anhydrous sodium sulfate, and concentrated under reduced pressure. The residue was analyzed by ^{1}H NMR to give the yield of the reaction.

4.5 The cross-coupling reaction of aryl halide catalyzed by Mn(I) complex with *d*-Mebpy as ligand

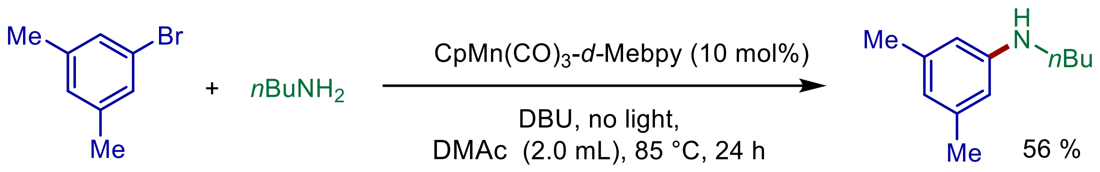

In a nitrogen-filled glove box, a magnetic stir bar, $CpMn(CO)_3$ (0.02 mmol), *d*-Mebpy (4,4'-Dimethyl-2,2'-bipyridine) (0.02 mmol), 3,5-dimethylbromobenzene (0.2 mmol), *n*-butylamine (0.4 mmol), DBU (1.5 equiv., 0.3 mmol) and 2 mL of DMAc were placed into an oven-dried 10 mL storage tube with a high vacuum valve and removed from the glove box. The reaction mixture was then irradiated with for 9 W purple LEDs at 85 °C or the tube was wrapped with aluminium foil and then stirred for 24 h at 85 °C. After cooling to room temperature, 1, 3-benzodioxole (0.2 mmol) was added to the reaction solution, the mixture was diluted with ethyl

acetate. The organic phases were washed with saturated brine (3 × 10 mL), dried over anhydrous sodium sulfate, and concentrated under reduced pressure. The residue was analyzed by ^{1}H NMR to give the yield of the reaction.

4.6 The catalytic reaction of alternating the light irradiation

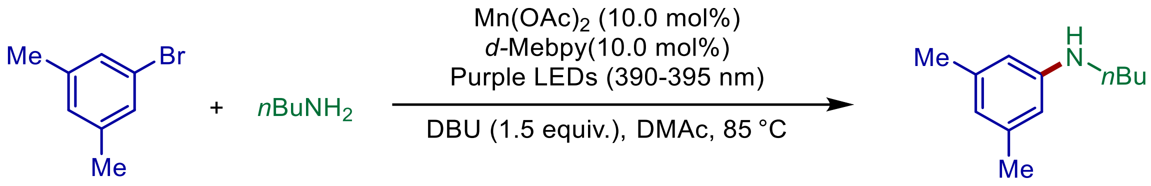

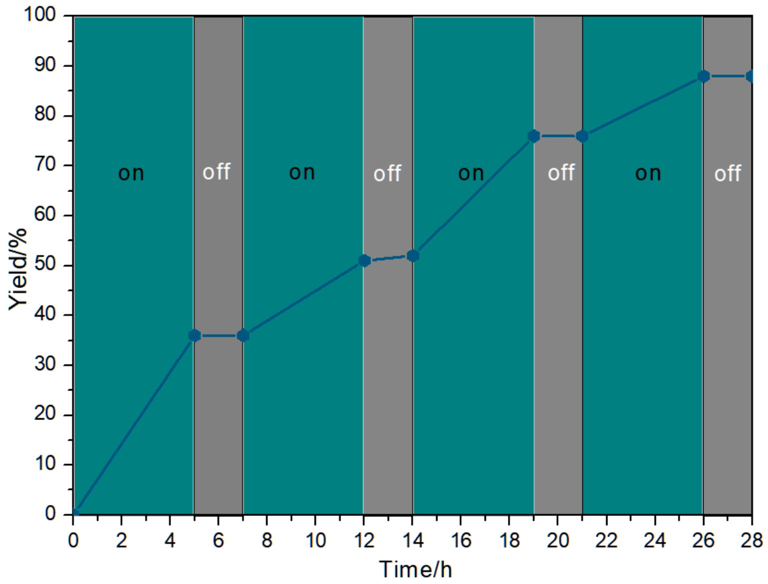

Figure S8. The experiment of the light irradiation.

To oven-dried 10 mL of storage tubes were added $Mn(OAc)_2$ (10.0 mol%), *d*-Mebpy (10.0 mol%), and 2 mL of DMAc with a magnetic stir bar under argon atmosphere. The mixture was evacuated and backfilled with Ar 3 times. Then the 3,5-dimethylbromobenzene (0.2 mmol), *n*-butylamine (0.4 mmol), and DBU (1.5 equiv., 0.3 mmol,) were added separately. The tubes were sealed with the Teflon screw valve. The reaction mixture was then irradiated with for 9 W purple LEDs (0.5 cm away from the tube, optical power: 320-340 mW/cm^2) at 85 °C. After a certain time, one was cooled to room temperature, and the other was wrapped with aluminium foil to continue stirring for 2 hours at 85 °C. Upon cooling to room temperature, 1,3-benzodioxole (0.2 mmol) was added to the reaction solution. The resulting mixtures were diluted with 5 mL $CHCl_3$, and the solvent was removed under reduced pressure and the residue was analyzed by ^{1}H NMR to give the yield of the reaction (Each reaction was repeated three times and the average yields were used in Figure S7, we selected 5 h, 10 h, 15 h, and 20 h respectively.).

4.7 Mn(III) reduction eliminates to yield C-N coupling product

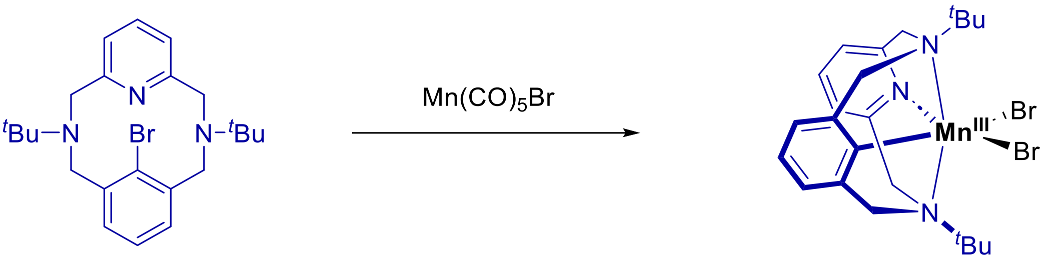

175 **176**

In a nitrogen-filled glove box, a magnetic stir bar, **175,** 43.0 mg (0.4 mmol) and MnBr(CO)$_5$ 110 mg (0.4 mmol) and 2 mL of DMAc were placed into an oven-dried 10 mL storage tube with a high vacuum valve to give a yellow suspension and removed from the glove box. The reaction mixture was then stirred at 110 °C. After 16 hours, the solution color turned wine-red and the solvent was completely evaporated under vacuum, without purification, proceed directly to the next step of the reaction.

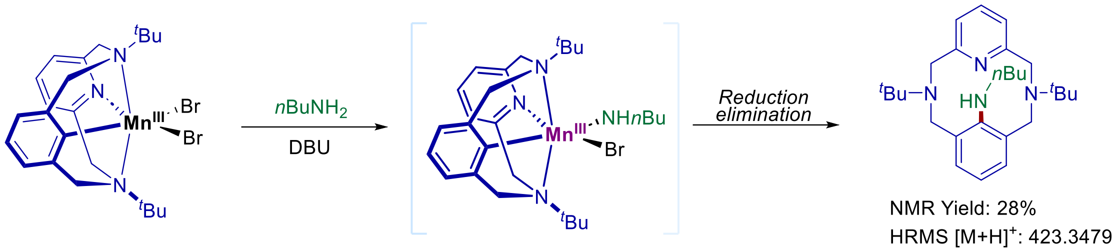

176 **177**

NMR Yield: 28%
HRMS [M+H]$^+$: 423.3479
178

In a nitrogen-filled glove box, a magnetic stir bar, the previously obtained 176, and *n*-butylamine (0.4 mmol), DBU (1.5 equiv., 0.3 mmol,) and 2 mL of DMAc were placed into an oven-dried 10 mL storage tube with a high vacuum valve separately and removed from the glove box. The reaction mixture was then stirred at 85 °C. After cooling to room temperature, the mixture was diluted with ethyl acetate. The organic phases were washed with saturated brine (3 × 10 mL), dried over anhydrous sodium sulfate, and concentrated under reduced pressure. The residue was analyzed by ^{1}H NMR to give the yield of the reaction.

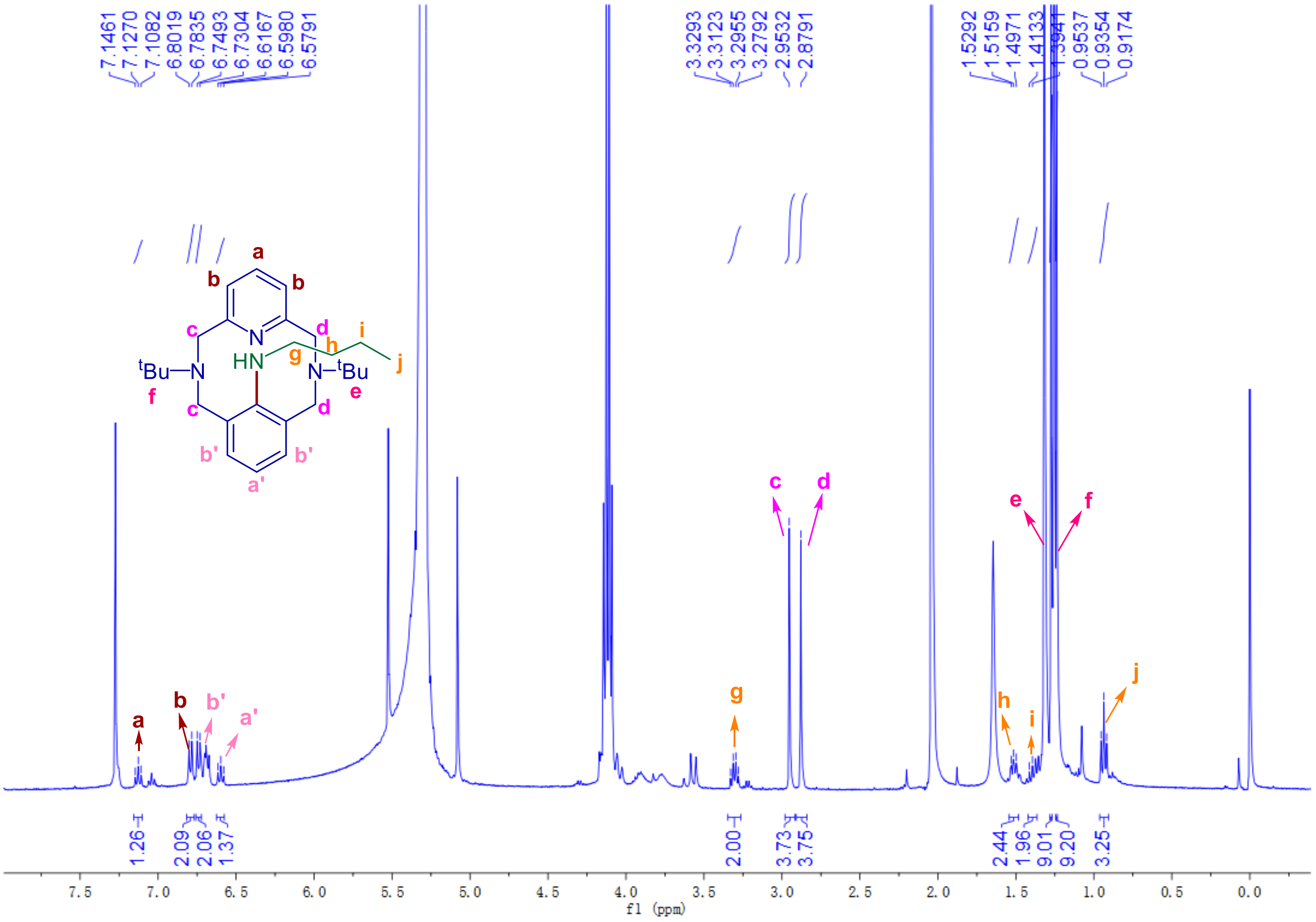

Figure S9. ^{1}H NMR of **178**

^{1}H NMR (400 MHz, $CDCl_3$) δ 7.13 (t, J = 7.6 Hz, 1H), 6.79 (d, J = 7.4 Hz, 2H), 6.74 (d, J = 7.6 Hz, 2H), 6.59 (t, J = 7.5 Hz, 1H), 3.38 – 3.30 (m, 2H), 2.95 (s, 4H), 2.88 (s, 4H), 1.54 – 1.48 (m, 2H), 1.48 – 1.40 (m, 2H), 1.27 (s, 9H), 1.24 (s, 9H), 0.94 (t, J = 7.3 Hz, 3H).

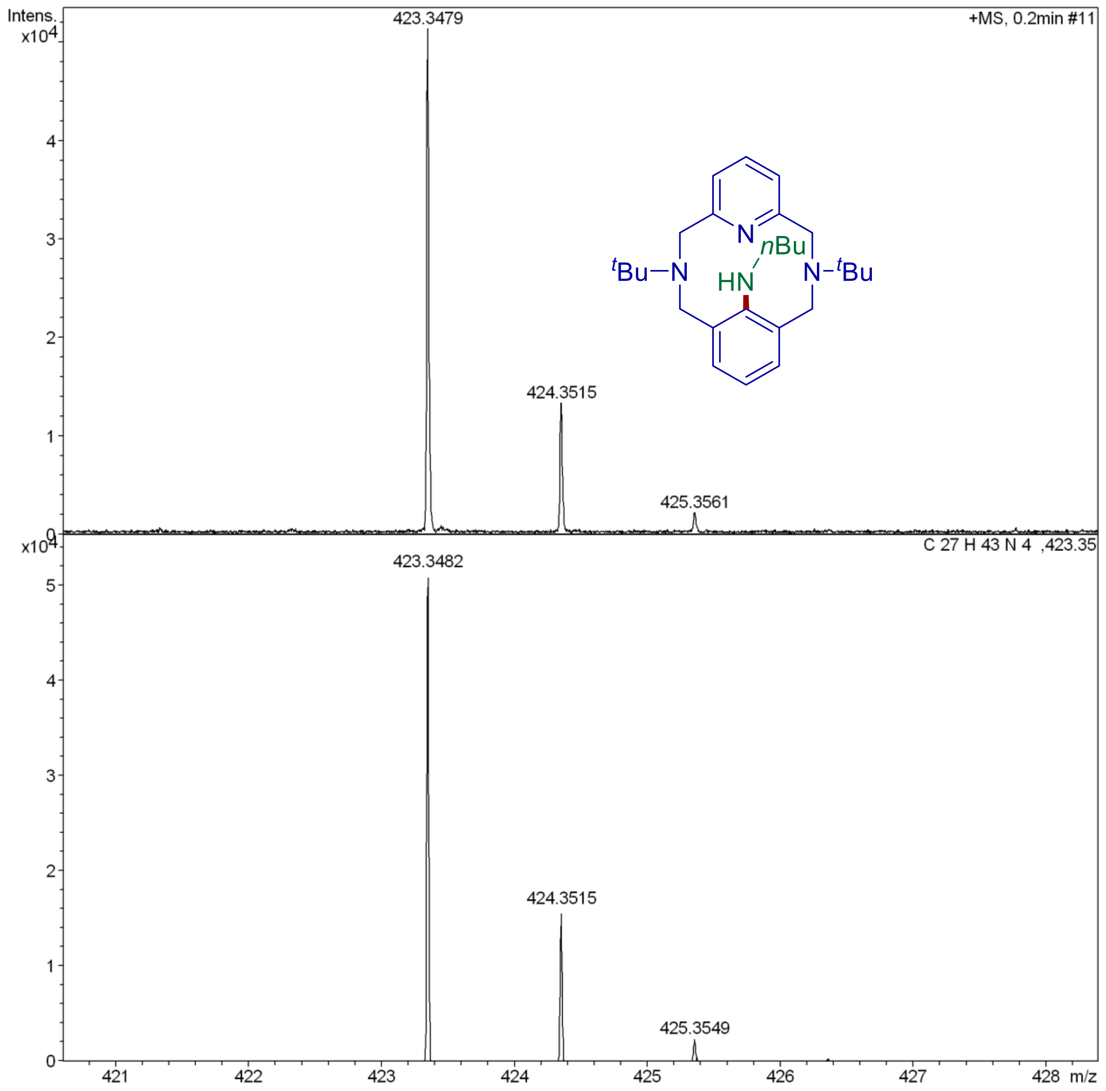

Figure S10. HRMS of **178**

5. Synthesis of precatalyst Mn complex 174.

To a dry MeOH (4 mL) suspension of $Mn(OAc)_2$ (0.4 mmol), *d*-Mebpy (0.4 mmol) in an oven-dried 25 mL screw-cap vials. The resulting mixture was stirred at 80 ˚C for 6 h under argon. After cooling to room temperature and removal of all the solvent, the precipitate was collected on a frit, rinsed with pentane and residual solvent was removed under reduced vacuum to give the title compound as a yellow powder (78% yield). Crystals suitable for X-ray single-crystal diffraction analysis was obtained through slowly evaporating the mixture solution of DCM and *n*-hexane at room temperature. CIF file for Mn complex 17 has been deposited at the Cambridge Crystallographic Data Centre with deposition number 2224253.

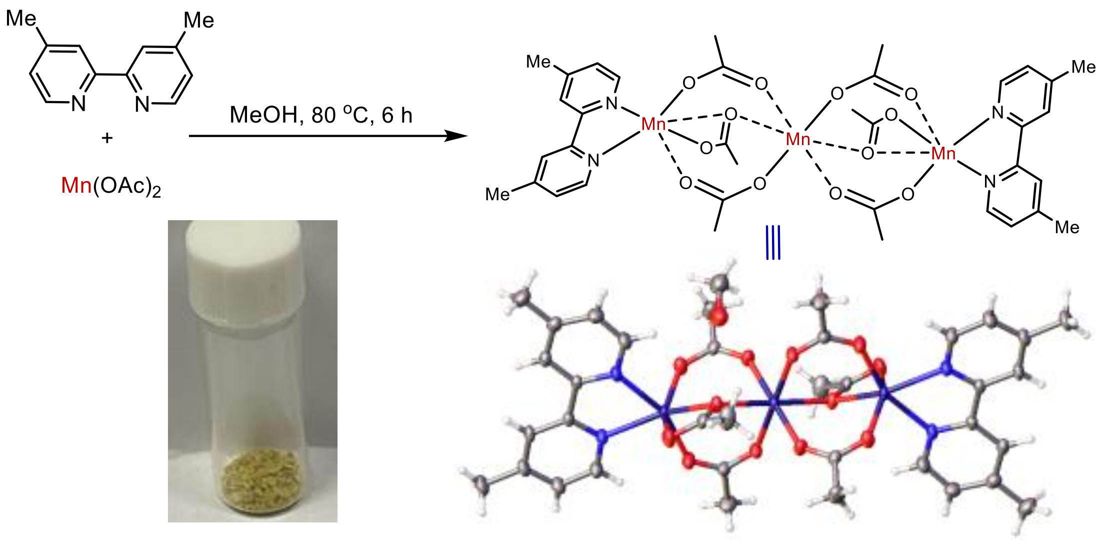

X -ray
Mn complex 174

CCDC number: 2224253

Table 1 Crystal data and structure refinement for Mn complex 174.

Identification code	Mn complex A
Empirical formula	$C_{38}H_{48}Mn_3N_4O_{14}$
Formula weight	949.62
Temperature/K	220.00
Crystal system	triclinic
Space group	P-1
a/Å	8.4280(4)
b/Å	10.2353(5)
c/Å	13.3259(6)
α/°	73.849(2)
β/°	87.729(2)
γ/°	77.075(2)
Volume/Å^3	1075.88(9)
Z	1
ρ_{calc}g/cm^3	1.466
μ/mm^{-1}	7.640
F(000)	491.0
Crystal size/mm^3	0.5 × 0.4 × 0.3
Radiation	CuKα (λ = 1.54178)
2Θ range for data collection/°	6.908 to 136.578
Index ranges	$-10 \le h \le 10$, $-12 \le k \le 11$, $-16 \le l \le 16$

Reflections collected	9218
Independent reflections	3826 [R_{int} = 0.0360, R_{sigma} = 0.0416]
Data/restraints/parameters	3826/0/274
Goodness-of-fit on F^2	0.981
Final R indexes [I>=2σ (I)]	R_1 = 0.0371, wR_2 = 0.1148
Final R indexes [all data]	R_1 = 0.0393, wR_2 = 0.1187
Largest diff. peak/hole / e $Å^{-3}$	0.45/-0.51

6. Analytical data of products.

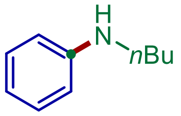

***N*-Butylaniline (3):** yellow oil; ^{1}H NMR (400 MHz, $CDCl_3$) δ 7.17 (t, *J* = 7.6 Hz, 2H), 6.69 (t, *J* = 7.3 Hz, 1H), 6.61 (d, *J* = 8.4 Hz, 2H), 3.12 (t, *J* = 7.1 Hz, 2H), 1.66 - 1.57 (m, 2H), 1.52 - 1.37 (m, 2H), 0.96 (t, *J* = 7.3 Hz, 3H); ^{13}C NMR (100 MHz, $CDCl_3$) δ 148.7, 129.4, 117.2, 112.9, 43.9, 31.9, 20.5, 14.0; HRMS (ESI) m/z calc. for $C_{10}H_{16}N$ $[M+H]^+$: 150.1277, found: 150.1279.

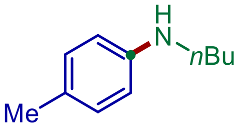

***N*-Butyl-4-methylaniline (4):** yellow oil; ^{1}H NMR (400 MHz, $CDCl_3$) δ 7.04 (d, *J* = 8.5 Hz, 2H), 6.58 (d, *J* = 8.3 Hz, 2H), 3.38 (br, 1H), 3.13 (t, *J* = 7.1 Hz, 2H), 2.29 (s, 3H), 1.69 - 1.69 (m, 2H), 1.54 - 1.42 (m, 2H), 1.01 (t, *J* = 7.3 Hz, 3H); ^{13}C NMR (100 MHz, $CDCl_3$) δ 146.4, 129.8, 126.4, 113.0, 44.2, 31.8, 20.5, 20.4, 14.0; HRMS (ESI) m/z calc. for $C_{11}H_{18}N$ $[M+H]^+$: 164.1434, found: 164.1435.

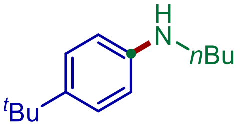

4-(Tert-butyl)-N-butylaniline (5):yellow oil; ^{1}H NMR (400 MHz, $CDCl_3$) δ 7.22 (d, *J* = 8.6 Hz, 2H), 6.58 (d, *J* = 8.6 Hz, 2H), 3.11 (t, *J* = 7.1 Hz, 2H), 1.65 - 1.57 (m, 2H), 1.50 - 1.39 (m, 2H), 1.30 (s, 9H), 0.97 (t, *J* = 7.3 Hz, 3H); ^{13}C NMR (100 MHz, $CDCl_3$) δ 146.4, 140.0, 126.1, 112.6, 44.1, 30.0, 31.9, 31.7, 20.5, 14.1; HRMS (ESI) m/z calc. for $C_{14}H_{24}N$ $[M+H]^+$: 206.1903, found: 206.1901.

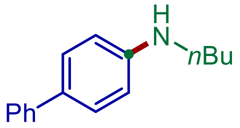

N-butyl-[1,1'-biphenyl]-4-amine (6): *yellow* oil; ^{1}H NMR (400 MHz, $CDCl_3$) δ 7.57 - 7.48 (m, 2H), 7.47 - 7.41 (m, 2H), 7.37 (t, *J* = 7.7 Hz, 2H), 7.27 - 7.20 (m, 1H), 6.74 - 6.61 (m, 2H), 5.66 - 5.64 (m, 1H), 3.14 (t, *J* = 7.1 Hz, 3H, NH, CH_2), 1.68 - 1.57 (m, 2H), 1.49 - 1.38 (m, 2H), 0.96 (t, *J* = 7.3 Hz, 3H); ^{13}C NMR (100 MHz, $CDCl_3$) δ 148.0, 141.4, 130.0, 128.7, 127.9, 126.3, 126.0, 113.0, 43.8, 31.7, 20.3, 14.0; HRMS (ESI) m/z calc. for $C_{16}H_{20}N$ $[M+H]^+$: 226.1590, found: 226.1595.

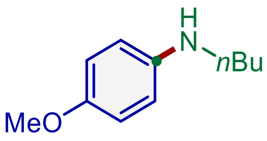

***N*-Butyl-4-methoxyaniline (7):** yellow oil; ^{1}H NMR (400 MHz, $CDCl_3$) δ 6.79 (d, J = 8.9 Hz, 2H), 6.59 (d, J = 8.9 Hz, 2H), 3.75 (s, 3H), 3.07 (t, J = 7.1 Hz, 2H), 1.66 - 1.54 (m, 2H), 1.49 - 1.37 (m, 2H), 0.96 (t, J = 7.3 Hz, 3H); ^{13}C NMR (100 MHz, $CDCl_3$) δ 152.2, 143.0, 115.1, 114.2, 56.0, 44.9, 31.9, 20.5, 14.1; HRMS (ESI) m/z calc. for $C_{11}H_{18}NO$ $[M+H]^+$: 180.1383, found: 180.1386.

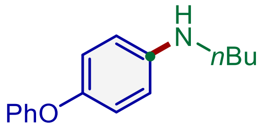

***N*-Butyl-4-phenoxyaniline (8):** yellow oil; ^{1}H NMR (400 MHz, $CDCl_3$) δ 7.35 - 7.18 (m, 2H), 6.98 (t, J = 7.3 Hz, 1H), 6.99 - 6.84 (m, 4H), 6.57 (d, J = 8.8 Hz, 2H), 3.35 (br, 1H), 3.08 (t, J = 7.1 Hz, 2H), 1.66 - 1.52 (m, 2H), 1.51 - 1.35 (m, 2H), 0.96 (t, J = 7.3 Hz, 3H); ^{13}C NMR (100 MHz, $CDCl_3$) δ 159.3, 147.4, 145.3, 129.5, 121.9 , 121.3, 117.1, 113.7 , 44.2, 31.8, 20.4, 14.0; HRMS (ESI) m/z calc. for $C_{16}H_{20}NO$ $[M+H]^+$: 242.1539, found: 242.1535.

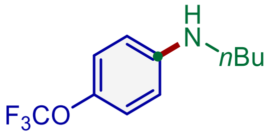

***N*-Butyl-4-(trifluoromethoxy)aniline (9):** yellow oil; ^{1}H NMR (400 MHz, $CDCl_3$) δ 7.02 (d, J = 8.6 Hz, 2H), 6.54 (d, J = 8.9 Hz, 2H), 3.66 (s, 1H), 3.09 (t, J = 7.1 Hz, 2H), 1.65 - 1.58 (m, 3H), 1.48 - 1.38 (m, 2H), 0.97 (t, J = 7.3 Hz, 3H). ^{13}C NMR (100 MHz, $CDCl_3$) δ 147.5, 140.4, 122.6, 122.5, (q, J = 250.2 Hz), 112.9, 110.1, 44.0, 31.7, 20.4, 14.0; ^{19}F NMR (376 MHz, $CDCl_3$) δ -59.40 (s, OCF_3); HRMS (ESI) m/z calc. for $C_{11}H_{15}F_3NO$ $[M+H]^+$: 234.1100, found: 234.1102.

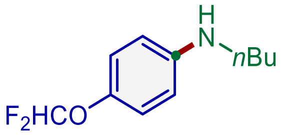

***N*-Butyl-4-(difluoromethoxy)aniline (10):** yellow oil; ^{1}H NMR (400 MHz, $CDCl_3$) δ 6.96 (d, J = 8.6 Hz, 2H), 6.54 (d, J = 8.7 Hz, 2H), 6.18 (t, J = 75.3 Hz, 1H), 3.59 (br, 1H), 3.08 (t, J = 7.1 Hz, 2H), 1.65 - 1.55 (m, 2H), 1.48 - 1.37 (m, 2H), 0.96 (t, J = 7.3 Hz, 3H); ^{13}C NMR (100 MHz, $CDCl_3$) δ 146.7, 142.4, 121.6, 116.8 (t, J = 258.6 Hz), 113.3, 44.1, 31.7, 20.4, 14.0; ^{19}F

NMR (376 MHz, $CDCl_3$) δ -79.86 (J = 75.2 Hz, OCF_2); HRMS (ESI) m/z calc. for $C_{11}H_{16}F_2NO$ $[M+H]^+$: 216.1194, found: 216.1195.

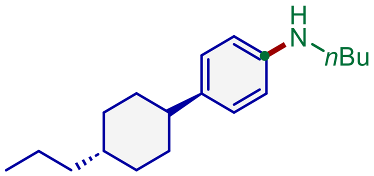

***N*-Butyl-4-(4-propylcyclohexyl)aniline (11):**yellow solid; ^{1}H NMR (400 MHz, $CDCl_3$) δ 7.04 (d, J = 8.4 Hz, 2H), 6.57 (d, J = 8.4 Hz, 2H), 3.19 (br, 1H), 3.11 (t, J = 7.1 Hz, 2H), 2.43 - 2.31 (m, 1H), 1.87 (t, J = 9.8 Hz, 4H), 1.66 - 1.56 (m, 2H), 1.48 - 1.34 (m, 6H), 1.33 - 1.17 (m, 4H), 1.06 - 1.02 (m, 1H), 0.97 (t, J = 7.3 Hz, 3H), 0.92 (t, J = 7.3 Hz, 3H); ^{13}C NMR (100 MHz, $CDCl_3$) δ 146.8, 136.9, 127.6, 112.9, 44.1, 43.8, 39.9, 37.2, 34.8, 33.9, 31.9, 20.5, 20.2, 14.6, 14.1; HRMS (ESI) m/z calc. for $C_{19}H_{32}N$ $[M+H]^+$: 274.2529, found: 274.2533.

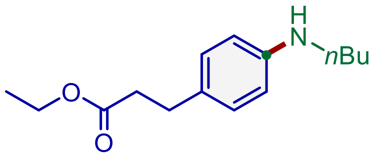

Ethyl 3-(4-(butylamino)phenyl)propanoate (12): yellow oil; ^{1}H NMR (400 MHz, $CDCl_3$) δ 7.01 (d, J = 8.3 Hz, 2H), 6.54 (d, J = 8.4 Hz, 2H), 4.13 (q, J = 7.1 Hz, 2H), 3.09 (t, J = 7.1 Hz, 2H), 2.89 - 2.78 (m, 2H), 2.62 - 2.52 (m, 2H), 1.65 - 1.54 (m, 2H), 1.50 - 1.37 (m, 2H), 1.24 (t, J = 7.1 Hz, 3H), 0.96 (t, J = 7.3 Hz, 3H); ^{13}C NMR (100 MHz, $CDCl_3$) δ 173.3, 147.1, 129.2, 113.0, 60.4, 44.0, 36.6, 31.9, 30.3, 20.4, 14.3, 14.0; HRMS (ESI) m/z calc. for $C_{15}H_{24}NO_2$ $[M+H]^+$: 250.1802, found: 250.1803.

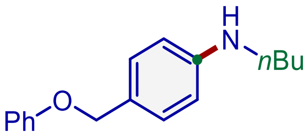

***N*-Butyl-4-(phenoxymethyl)aniline (13):** yellow oil; ^{1}H NMR (400 MHz, $CDCl_3$) δ 7.35 (d, J = 7.3 Hz, 2H), 7.29 (t, J = 7.3 Hz, 2H), 7.23 (d, J = 7.1 Hz, 1H), 6.77 (d, J = 8.9 Hz, 2H), 6.50 (d, J = 8.9 Hz, 2H), 4.91 (s, 2H), 2.99 (t, J = 7.1 Hz, 2H), 1.56 - 1.45 (m, 2H), 1.40 - 1.28 (m, 2H), 0.88 (t, J = 7.3 Hz, 3H); ^{13}C NMR (100 MHz, $CDCl_3$) δ 151.2, 143.0, 137.7, 128.5, 127.8, 127.58, 116.2, 114.0, 70.9, 44.7, 31.8, 20.3, 13.9; HRMS (ESI) m/z calc. for $C_{17}H_{22}NO$ $[M+H]^+$: 256.1696, found: 256.1698.

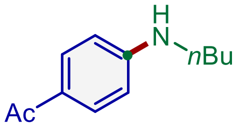

1-(4-(butylamino)phenyl)ethan-1-one (14): yellow solid; ^{1}H NMR (400 MHz, $CDCl_3$) δ 7.74 (d, J = 8.7 Hz, 2H), 6.47 (d, J = 8.7 Hz, 2H), 4.20 (br, 1H), 3.10 (t, J = 7.1 Hz, 2H), 2.41 (s, 3H), 1.57 - 1.50 (m, 2H), 1.39 - 1.30 (m, 2H), 0.88 (t, J = 7.3 Hz, 3H); ^{13}C NMR (100 MHz, $CDCl_3$) δ 196.3, 152.4, 130.8, 126.4, 111.2, 43.0, 31.4, 26.0, 20.2, 13.8; HRMS (ESI) m/z calc. for $C_{12}H_{18}NO$ $[M+H]^+$: 192.1383, found: 192.1380.

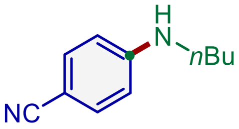

4-(Butylamino)benzonitrile (15): yellow solid; ^{1}H NMR (400 MHz, $CDCl_3$) δ 7.40 (d, J = 8.8 Hz, 1H), 6.54 (d, J = 8.8 Hz, 1H), 4.25 (br, 1H), 3.14 (t, J = 7.1 Hz, 1H), 1.64 - 1.59 (m, 7.2 Hz, 1H), 1.46 - 1.39 (m, 1H), 0.96 (t, J = 7.3 Hz, 1H); ^{13}C NMR (100 MHz, $CDCl_3$) δ 151.5, 133.7, 120.6, 112.0, 98.2, 42.9, 31.2, 20.2, 13.8; HRMS (ESI) m/z calc. for $C_{11}H_{15}N_2$ $[M+H]^+$: 175.1230, found: 175.1234.

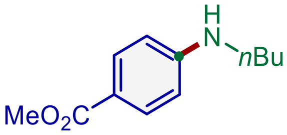

Methyl 4-(butylamino)benzoate (16): yellow solid; ^{1}H NMR (400 MHz, $CDCl_3$) δ 7.85 (d, J = 8.7 Hz, 2H), 6.53 (d, J = 8.6 Hz, 2H), 4.12 (br, 1H), 3.84 (s, 3H), 3.15 (t, J = 7.1 Hz, 2H), 1.72 - 1.52 (m, 2H), 1.51 - 1.34 (m, 2H), 0.96 (t, J = 7.3 Hz, 3H); ^{13}C NMR (100 MHz, $CDCl_3$) δ 167.4, 152.2, 131.6, 118.0, 111.3, 51.5, 43.1, 31.4, 20.2, 13.8; HRMS (ESI) m/z calc. for $C_{12}H_{18}NO_2$ $[M+H]^+$: 208.1332, found: 208.1337.

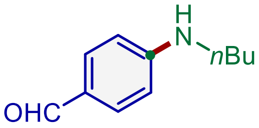

4-(butylamino)benzaldehyde (17): yellow solid; ^{1}H NMR (400 MHz, $CDCl_3$) δ 9.64 (s, 1H), 7.61 (d, J = 8.6 Hz, 2H), 6.52 (d, J = 8.6 Hz, 2H), 4.31 (br, 1H), 3.13 (t, J = 7.1 Hz, 2H), 1.60 - 1.52 (m, 2H), 1.44 - 1.38 (m, 2H), 0.90 (t, J = 7.3 Hz, 3H); ^{13}C NMR (100 MHz, $CDCl_3$) δ 190.2, 153.5, 132.4, 126.3, 111.7, 43.0, 31.3, 20.2, 13.8; HRMS (ESI) m/z calc. for $C_{11}H_{16}NO$ $[M+H]^+$:178.1226, found: 178.1223.

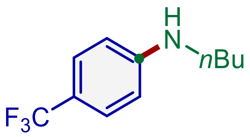

***N*-Butyl-4-(trifluoromethyl)aniline (18):** yellow oil; ^{1}H NMR (400 MHz, $CDCl_3$) δ 7.42 (d, *J* = 8.3 Hz, 2H), 6.62 (d, *J* = 8.3 Hz, 2H), 3.97 (br, 1H), 3.17 (t, *J* = 6.3 Hz, 2H), 1.71 - 1.60 (m, 2H), 1.56 - 1.42 (m, 2H), 1.00 (t, *J* = 7.3 Hz, 3H); ^{13}C NMR (100 MHz, $CDCl_3$) δ 151.0, 126.69 (q, *J* = 3.8 Hz), 122.5 (q, *J* = 270.2 Hz), 118.5 (q, *J* = 32.6 Hz), 111.8, 43.3, 31.5, 20.3, 13.9; ^{19}F NMR (376 MHz, $CDCl_3$) δ -60.93 (s, CF_3); HRMS (ESI) m/z calc. for $C_{11}H_{15}F_3N$ $[M+H]^+$: 218.1151, found: 218.1155.

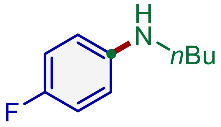

***N*-Butyl-4-fluoroaniline (19):** yellow oil; ^{1}H NMR (400 MHz, $CDCl_3$) δ 6.88 (t, *J* = 8.7 Hz, 2H), 6.59 - 6.49 (m, 2H), 3.47 (br, 1H), 3.07 (t, *J* = 7.1 Hz, 2H), 1.64 - 1.55 (m, 2H), 1.48 - 1.38 (m, 2H), 0.96 (t, *J* = 7.3 Hz, 3H); ^{13}C NMR (100 MHz, $CDCl_3$) δ 155.7 (d, *J* = 233.0 Hz), 144.9, 115.6 (d, *J* = 22.0 Hz), 113.5 (d, *J* = 7.5 Hz), 44.4, 31.7, 20.3, 13.9; ^{19}F NMR (376 MHz, $CDCl_3$) δ -128.59 (s, F); HRMS (ESI) m/z calc. for $C_{10}H_{15}FN$ $[M+H]^+$: 168.1183, found: 168.1185.

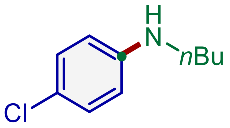

***N*-Butyl-4-chloroaniline (20):** yellow oil; ^{1}H NMR (400 MHz, $CDCl_3$) δ 7.11 (d, *J* = 8.7 Hz, 2H), 6.51 (d, *J* = 8.7 Hz, 2H), 3.60 (br, 1H), 3.08 (t, *J* = 7.1 Hz, 2H), 1.64 - 1.55 (m, 2H), 1.48 - 1.37 (m, 2H), 0.96 (t, *J* = 7.3 Hz, 3H); ^{13}C NMR (100 MHz, $CDCl_3$) δ 147.2, 129.1, 121.7, 113.8, 43.9, 31.7, 20.4, 14.0; HRMS (ESI) m/z calc. for $C_{10}H_{15}ClN$ $[M+H]^+$: 184.0888, found: 184.0886.

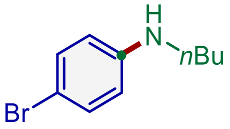

4-Bromo-*N*-butylaniline (21): yellow oil; ^{1}H NMR (400 MHz, $CDCl_3$) δ 7.23 (d, *J* = 8.8 Hz, 2H), 6.46 (d, *J* = 8.8 Hz, 2H), 3.57 (br, 1H), 3.06 (t, *J* = 7.1 Hz, 2H), 1.64 - 1.51 (m, 2H), 1.47 - 1.34 (m, 2H), 0.95 (t, *J* = 7.3 Hz, 3H); ^{13}C NMR (100 MHz, $CDCl_3$) δ 147.5, 131.9, 114.2,

108.5, 43.7, 31.5, 20.3, 13.9; HRMS (ESI) m/z calc. for $C_{10}H_{15}BrN$ $[M+H]^+$: 228.0382, found: 228.0383.

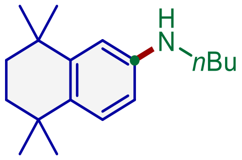

***N-Butyl-5,5,8,8-tetramethyl-5,6,7,8-tetrahydronaphthalen-2-amine* (22):** yellow oil; ^{1}H NMR (400 MHz, $CDCl_3$) δ 7.13 (t, J = 7.5 Hz, 1H), 6.55 (t, J = 3.2 Hz, 1H), 6.50 - 6.43 (m, 1H), 3.11 (t, J = 7.0 Hz, 2H), 1.73 - 1.76 (m, 4H), 1.64 - 1.57 (m, 2H), 1.52 - 1.39 (m, 2H), 1.36 - 1.00 (m, 12H), 0.98 (t, J = 7.3 Hz, 3H); ^{13}C NMR (100 MHz, $CDCl_3$) δ 146.3, 145.8, 134.0, 127.4, 111.2, 110.5, 44.1, 35.5, 35.4, 34.4, 33.6, 32.2, 32.0, 32.0, 20.5, 14.1; HRMS (ESI) m/z calc. for $C_{18}H_{30}N$ $[M+H]^+$: 260.2373, found: 260.2374.

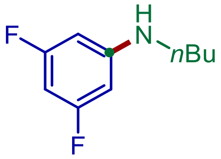

***3-Bromo-N-butylaniline* (23):** yellow oil; b^{1}H NMR (400 MHz, $CDCl_3$) δ 6.44 - 6.33 (m, 1H), 6.26 - 6.02 (m, 2H), 3.87 (br, 1H), 3.09 (t, J = 7.1 Hz, 2H), 1.65 - 1.58 (m, 2H), 1.45 (m, 2H), 0.99 (t, J = 7.3 Hz, 3H); ^{13}C NMR (100 MHz, $CDCl_3$) δ 164.19 (dd, J = 243.6, 16.1 Hz), 150.62 (dd, J = 25.1, 12.8 Hz), 108.38 (d, J = 2.4 Hz), 104.26 (d, J = 25.6 Hz), 97.66 (d, J = 25.3 Hz), 95.15 (d, J = 28.5 Hz), 91.94 (t, J = 26.3 Hz), 43.6, 31.4, 20.3, 14.0; ^{19}F NMR (376 MHz, $CDCl_3$) δ -110.76 (s, F); HRMS (ESI) m/z calc. for $C_{10}H_{14}F_2N$ $[M+H]^+$: 186.1089, found: 186.1093.

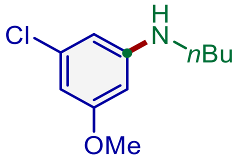

***N-Butyl-3-chloro-5-methoxyaniline* (24):** yellow oil; ^{1}H NMR (400 MHz, $CDCl_3$) δ 6.24 (d, J = 1.7 Hz, 1H), 6.20 (s, 1H), 6.01 (s, 1H), 3.75 (s, 3H), 3.69 (br, 1H), 3.06 (t, J = 7.1 Hz, 2H), 1.62 - 1.54 (m, 2H), 1.45 - 1.36 (m, 2H), 0.96 (t, J = 7.3 Hz, 3H); ^{13}C NMR (100 MHz, $CDCl_3$) δ 161.4, 150.4, 135.5, 105.9, 102.9, 97.1, 55.4, 43.6, 31.6, 20.4, 14.0; HRMS (ESI) m/z calc. for $C_{11}H_{17}ClNO$ $[M+H]^+$: 214.0993, found: 214.0995.

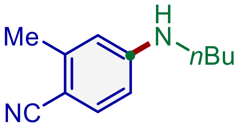

4-(Butylamino)-2-methylbenzonitrile (25): yellow oil; ^{1}H NMR (400 MHz, $CDCl_3$) δ 7.35 (d, *J* = 8.2 Hz, 1H), 6.41 - 6.32 (m, *J* = 10.7 Hz, 2H), 4.07 (br, 1H), 3.19 - 3.02 (m, 2H), 2.43 (s, 3H), 1.66 - 1.54 (m, 2H), 1.47 - 1.40 (m, 2H), 0.96 (t, *J* = 7.8 Hz, 3H); ^{13}C NMR (100 MHz, $CDCl_3$) δ 151.4, 143.5, 134.0, 119.9, 112.9, 109.9, 99.2, 42.9, 31.3, 20.7, 20.2, 13.8; HRMS (ESI) m/z calc. for $C_{12}H_{17}N_2$ $[M+H]^+$: 189.1386, found: 189.1390.

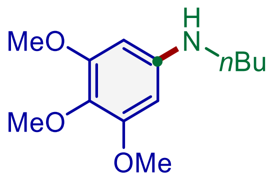

N-Butyl-3,4,5-trimethoxyaniline (26): yellow oil; ^{1}H NMR (400 MHz, $CDCl_3$) δ 5.84 (s, 2H), 3.82 (s, 6H), 3.76 (s, 3H), 3.48 (br, 1H), 3.08 (t, *J* = 7.0 Hz, 2H), 1.65 - 1.54 (m, 2H), 1.49 - 1.38 (m, 2H), 0.96 (t, *J* = 7.3 Hz, 3H); ^{13}C NMR (100 MHz, $CDCl_3$) δ 155.1, 146.5, 131.1, 91.4, 62.2, 57.1, 45.2, 32.9, 21.4, 15.1; HRMS (ESI) m/z calc. for $C_{13}H_{22}NO_3$ $[M+H]^+$: 240.1594, found: 240.1597.

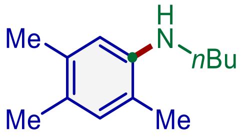

N-Butyl-2,4,5-trimethylaniline (27): yellow oil; ^{1}H NMR (400 MHz, $CDCl_3$) δ 6.84 (s, 1H), 6.46 (s, 1H), 3.16 (t, *J* = 7.0 Hz, 2H), 2.24 (s, 3H), 2.17 (s, 3H), 2.10 (s, 3H), 1.72 - 1.62 (m, 2H), 1.54 - 1.41 (m, 2H), 0.99 (t, *J* = 7.3 Hz, 3H); ^{13}C NMR (100 MHz, $CDCl_3$) δ 144.6, 134.8, 131.6, 124.4, 119.3, 111.9, 44.2, 32.0, 20.5, 20.0, 18.7, 17.0, 14.1; HRMS (ESI) m/z calc. for $C_{13}H_{22}N$ $[M+H]^+$: 192.1747, found: 192.1750.

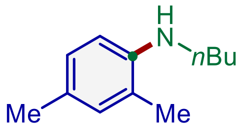

N-butyl-2,4-dimethylaniline (28): yellow oil; ^{1}H NMR (400 MHz, $CDCl_3$) δ 7.14 - 6.98 (m, 1H), 6.92 (d, *J* = 8.0 Hz, 1H), 6.87 (s, 1H), 6.53 (d, *J* = 8.0 Hz, 1H), 3.13 (t, *J* = 6.8 Hz, 2H), 2.22 (s, 3H), 2.10 (s, 3H), 1.74 - 1.59 (m, 2H), 1.52 - 1.37 (m, 2H), 0.96 (t, *J* = 6.9 Hz, 3H); ^{13}C NMR (100 MHz, $CDCl_3$) δ 144.2, 130.9, 127.3, 125.8, 121.9, 109.9, 44.0, 31.8, 20.4, 20.3, 17.4, 14.0; HRMS (ESI) m/z calc. for $C_{12}H_{20}N$ $[M+H]^+$: 178.1590, found: 178.1593.

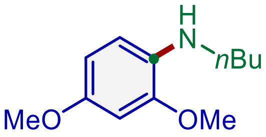

N-Butyl-2,4-dimethoxyaniline (29): yellow oil; ^{1}H NMR (400 MHz, $CDCl_3$) δ 6.53 (d, J = 8.4 Hz, 1H), 6.45 (s, 1H), 6.41 (d, J = 8.5 Hz, 1H), 3.82 (s, 3H), 3.76 (s, 3H), 3.08 (t, J = 7.1 Hz, 2H), 1.67 - 1.59 (m, 2H), 1.49 - 1.39 (m, 2H), 0.96 (t, J = 7.3 Hz, 3H); ^{13}C NMR (150 MHz, $CDCl_3$) δ 151.9, 148.0, 133.1, 110.3, 104.0, 99.3, 56.0, 55.6, 44.4, 31.9, 20.6, 14.1; HRMS (ESI) m/z calc. for $C_{12}H_{20}NO_2$ $[M+H]^+$: 210.1489, found: 210.1490.

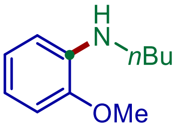

N-Butyl-2-methoxyaniline (30): yellow oil; ^{1}H NMR (400 MHz, $CDCl_3$) δ 6.89 (t, J = 7.5 Hz, 1H), 6.79 (d, J = 7.8 Hz, 1H), 6.73 - 6.64 (m, 2H), 4.19 (br, 1H), 3.86 (s, 3H), 3.15 (t, J = 7.0 Hz, 2H), 1.72 - 1.61 (m, 2H), 1.54 - 1.40 (m, 2H), 0.99 (t, J = 7.3 Hz, 3H); ^{13}C NMR (100 MHz, $CDCl_3$) δ 147.0, 138.8, 121.5, 116.3, 109.9, 109.6, 55.6, 43.6, 31.9, 20.6, 14.2; HRMS (ESI) m/z calc. for $C_{11}H_{18}NO$ $[M+H]^+$: 180.1383, found: 180.1386.

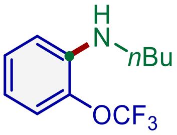

N-Butyl-2-(trifluoromethoxy)aniline (31): yellow oil; ^{1}H NMR (400 MHz, $CDCl_3$) δ 7.10 - 7.01 (m, 2H), 6.65 (d, J = 8.0 Hz, 1H), 6.61 - 6.48 (m, 1H), 3.98 (br, 1H), 3.08 (t, J = 7.1 Hz, 2H), 1.61 - 1.50 (m, 2H), 1.47 - 1.30 (m, 2H), 0.89 (t, J = 7.3 Hz, 3H); ^{13}C NMR (100 MHz, $CDCl_3$) δ 140.8, 136.1, 127.7, 122.2, 121.0 (q, J = 126.3 Hz), 120.8 116.0, 112.0, 43.2, 31.4, 20.2, 13.8; ^{19}F NMR (376 MHz, $CDCl_3$) δ -60.59 (s, OCF_3); HRMS (ESI) m/z calc. for $C_{11}H_{15}F_3NO$ $[M+H]^+$: 234.1100, found: 234.1098.

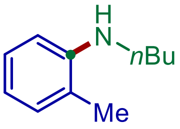

N-butyl-2-methylaniline (32): yellow oil; ^{1}H NMR (400 MHz, $CDCl_3$) δ 7.12 (t, J = 7.7 Hz, 1H), 7.04 (d, J = 7.2 Hz, 1H), 6.69 - 6.58 (m, 2H), 3.15 (t, J = 7.1 Hz, 2H), 2.12 (s, 3H), 1.68 – 1.62 (m, 2H), 1.49 -1.40 (m, 2H), 0.97 (t, J = 7.3 Hz, 3H); ^{13}C NMR (100 MHz, $CDCl_3$) δ 146.4,

130.02 (s), 127.2, 121.7, 116.7, 109.7, 43.7, 31.8, 20.4, 17.4, 14.0; HRMS (ESI) m/z calc. for $C_{11}H_{18}N$ [M+H]$^+$: 164.1434, found: 164.1430.

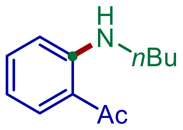

1-(2-(Butylamino)phenyl)ethan-1-one (33): yellow oil; ^{1}H NMR (400 MHz, $CDCl_3$) δ 8.86 (s, 1H), 7.73 (d, *J* = 8.0 Hz, 1H), 7.34 (t, *J* = 7.7 Hz, 1H), 6.70 (d, *J* = 8.4 Hz, 1H), 6.56 (t, *J* = 7.3 Hz, 1H), 3.26 - 3.18 (m, 2H), 2.57 (d, *J* = 1.3 Hz, 3H), 1.69 - 1.58 (m, 3H), 1.52 - 1.47 (m, 2H), 0.99 - 0.92 (m, 3H); ^{13}C NMR (100 MHz, $CDCl_3$) δ 200.7, 151.3, 135.0, 132.8, 117.3, 113.6 111.7, 42.3, 31.2, 27.9, 20.4, 13.8; HRMS (ESI) m/z calc. for $C_{12}H_{18}NO$ [M+H]$^+$: 192.1383, found: 192.1383.

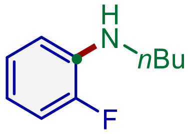

N-Butyl-2-fluoroaniline (34): yellow oil; ^{1}H NMR (400 MHz, $CDCl_3$) δ 6.96 - 6.75 (m, 2H), 6.65 - 6.60 (m, 1H), 6.59 - 6.51 (m, 1H), 3.77 (s, 1H), 3.06 (t, *J* = 7.1 Hz, 2H), 1.64 - 1.47 (m, 2H), 1.44 - 1.29 (m, 2H), 0.89 (t, *J* = 7.3 Hz, 3H); ^{13}C NMR (100 MHz, $CDCl_3$) δ 151.5 (d, *J* = 237.9 Hz), 137.0 (d, *J* = 11.5 Hz), 124.6 (d, *J* = 3.5 Hz), 116.2 (d, *J* = 7.0 Hz), 114.3 (d, *J* = 18.5 Hz), 112.0 (d, *J* = 3.5 Hz), 43.3, 31.6, 20.2, 13.9; ^{19}F NMR (376 MHz, $CDCl_3$) δ -137.12 (s, F); HRMS (ESI) m/z calc. for $C_{10}H_{15}FN$ [M+H]$^+$: 168.1183, found: 168.1187.

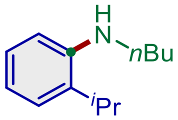

N-butyl-2-isopropylaniline (35): yellow oil; ^{1}H NMR (400 MHz, $CDCl_3$) δ 7.18 - 7.09 (m, 2H), 6.72 (t, *J* = 7.4 Hz, 1H), 6.64 (d, *J* = 8.0 Hz, 1H), 3.64 (br, 1H), 3.15 (t, *J* = 7.0 Hz, 2H), 2.89 - 2.80 (m, 1H), 1.69 - 1.60 (m, 2H), 1.54 - 1.35 (m, 2H), 1.25 (d, *J* = 6.8 Hz, 6H), 0.98 (t, *J* = 7.3 Hz, 2H); ^{13}C NMR (100 MHz, $CDCl_3$) δ 145.1, 131.9, 126.8, 124.9, 117.0, 110.4, 43.9, 31.8, 27.2, 22.3, 20.45, 14.0; HRMS (ESI) m/z calc. for $C_{13}H_{22}N$ [M+H]$^+$: 192.1747, found: 192.1749.

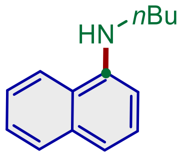

N-butylnaphthalen-1-amine (36): yellow oil; ^{1}H NMR (400 MHz, CDCl$_3$) δ 7.70 (d, J = 8.4 Hz, 2H), 7.45 - 7.29 (m, 2H), 7.26 (t, J = 7.9 Hz, 1H), 7.14 (s, 1H), 6.52 (d, J = 7.6 Hz, 1H), 4.19 (br, 1H), 3.18 (t, J = 7.1 Hz, 2H), 1.75 - 1.58 (m, 2H), 1.49 - 1.38 (m, 2H), 0.92 (t, J = 7.4 Hz, 3H); ^{13}C NMR (100 MHz, CDCl$_3$) δ 143.7, 134.4, 128.7, 126.7, 125.7, 124.6, 123.4, 119.8, 117.1, 104.3, 44.0, 31.6, 20.6, 14.0; HRMS (ESI) m/z calc. for $C_{14}H_{18}N$ [M+H]$^+$: 200.1434, found: 200.1430.

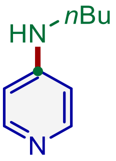

N-Butylpyridin-4-amine (37): yellow oil; ^{1}H NMR (400 MHz, CDCl$_3$) δ 8.10 (d, J = 4.9 Hz, 2H), 6.52 (d, J = 5.6 Hz, 2H), 3.16 (m, 2H), 1.63 (m, 2H), 1.43 (m, 2H), 0.96 (t, J = 7.3 Hz, 3H); ^{13}C NMR (100 MHz, CDCl$_3$) δ 154.6, 147.4, 107.4, 42.4, 31.0, 20.2, 13.8; HRMS (ESI) m/z calc. for $C_9H_{15}N_2$ [M+H]$^+$: 151.1230, found: 151.1233.

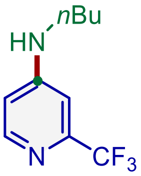

N-Butyl-2-(trifluoromethyl)pyridin-4-amine (38): yellow oil; ^{1}H NMR (400 MHz, CDCl$_3$) δ 8.17 (d, J = 5.2 Hz, 1H), 6.74 (s, 1H), 6.48 (d, J = 5.6 Hz, 1H), 4.91 (br, 1H), 3.18 - 3.10 (m, 2H), 1.64 - 1.49 (m, 2H), 1.43 - 1.30 (m, 2H), 0.91 (t, J = 7.3 Hz, 3H); ^{13}C NMR (100 MHz, CDCl$_3$) δ 154.6, 149.9, 148.6 (q, J = 33.0 Hz), 123.4 (q, J = 260.0 Hz), 108.9, 104.2, 42.5, 30.9, 20.1, 13.7; ^{19}F NMR (376 MHz, CDCl$_3$) δ -66.56 (s, CF$_3$); HRMS (ESI) m/z calc. for $C_{10}H_{14}F_3N_2$ [M+H]$^+$: 219.1104, found: 219.1106.

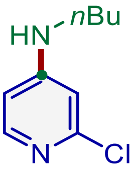

N-Butyl-2-chloropyridin-4-amine (39): yellow oil; ^{1}H NMR (400 MHz, CDCl$_3$) δ 7.88 (d, J = 5.8 Hz, 1H), 6.40 (d, J = 2.1 Hz, 1H), 6.32 (dd, J = 5.8, 2.2 Hz, 1H), 4.53 (br, 1H), 3.10 (m, 2H), 1.63 - 1.52 (m, 2H), 1.45 - 1.33 (m, 2H), 0.93 (t, J = 7.3 Hz, 3H); ^{13}C NMR (100 MHz, CDCl$_3$) δ 155.6, 152.3, 149.2, 107.3, 105.9, 42.6, 31.1, 20.2, 13.8; HRMS (ESI) m/z calc. for $C_9H_{14}ClN_2$ [M+H]$^+$: 185.0840, found: 185.0842.

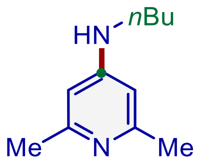

N-Butyl-2,6-dimethylpyridin-4-amine (40): yellow oil; ^{1}H NMR (600 MHz, $CDCl_3$) δ 6.14 (s, 2H), 4.16 (br, 1H), 3.11 (m, 2H), 2.37 (s, 6H), 1.62 - 1.54 (m, 2H), 1.45 - 1.36 (m, 2H), 0.95 (t, *J* = 7.4 Hz, 3H); ^{13}C NMR (100 MHz, $CDCl_3$) δ 157.5, 154.6, 104.1, 42.4, 31.3, 24.2, 20.2, 13.8; HRMS (ESI) m/z calc. for $C_{11}H_{19}N_2$ $[M+H]^+$: 179.1543, found: 164.1435. HRMS (ESI) m/z calc. for $C_{11}H_{19}N_2$ $[M+H]^+$: 179.1543, found: 179.1545.

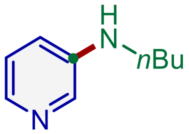

N-Butylpyridin-3-amine (41): yellow oil; ^{1}H NMR (400 MHz, $CDCl_3$) δ 8.00 (d, *J* = 2.7 Hz, 1H), 7.92 (d, *J* = 4.6 Hz, 1H), 7.07 (dd, *J* = 8.3, 4.6 Hz, 1H), 6.88 - 6.82 (m, 1H), 3.78 (br, 1H), 3.11 (t, *J* = 7.1 Hz, 2H), 1.65 - 1.55 (m, 2H), 1.48 - 1.37 (m, 2H), 0.95 (t, *J* = 7.3 Hz, 3H); ^{13}C NMR (100 MHz, $CDCl_3$) δ 144.6, 138.3, 135.8, 123.9, 118.6, 43.4, 31.6, 20.3, 14.0; HRMS (ESI) m/z calc. for $C_9H_{15}N_2$ $[M+H]^+$: 151.1230, found: 151.1233.

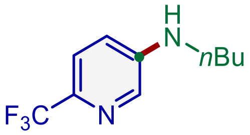

N-Butyl-6-(trifluoromethyl)pyridin-3-amine (42): yellow oil; ^{1}H NMR (400 MHz, $CDCl_3$) δ 8.29 (s, 1H), 7.55 (dd, *J* = 8.8, 2.3 Hz, 1H), 6.36 (d, *J* = 8.8 Hz, 1H), 5.16 (br, 1H), 3.35 - 3.46 (m, 2H), 1.66 - 1.51 (m, 2H), 1.47 - 1.34 (m, 2H), 0.94 (t, *J* = 7.3 Hz, 3H); ^{13}C NMR (100 MHz, $CDCl_3$) δ 160.7, 146.2, 134.5 (q, *J* = 3.1 Hz), δ 124.8 (q, *J* = 270.1 Hz), 115.2 (q, *J* = 33.0 Hz), 105.6, 41.9, 31.5, 20.2, 13.8; ^{19}F NMR (376 MHz, $CDCl_3$) δ -61.11 (s, CF_3); HRMS (ESI) m/z calc. for $C_{10}H_{14}F_3N_2$ $[M+H]^+$: 219.1104, found: 219.1108.

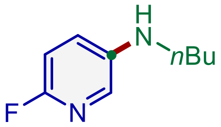

N-butyl-6-fluoropyridin-3-amine (43): yellow oil; ^{1}H NMR (400 MHz, $CDCl_3$) δ 8.01 (s, 1H), 7.35 (d, *J* = 8.9 Hz, 1H), 6.31 (d, *J* = 8.8 Hz, 1H), 4.53 (br, 1H), 3.22 (q, *J* = 6.5 Hz, 2H), 1.62 -1.59 (m, 2H), 1.48 - 1.41 (m, 2H), 0.95 (t, *J* = 7.3 Hz, 3H); ^{13}C NMR (100 MHz, $CDCl_3$) δ

157.3, 146.5, 137.1, 119.3, 107.1, 42.2, 31.5, 20.2, 13.8; ^{19}F NMR (376 MHz, $CDCl_3$) δ -109.3 (s, F); HRMS (ESI) m/z calc. for $C_9H_{14}FN_2$ $[M+H]^+$: 169.1136, found: 169.1138.

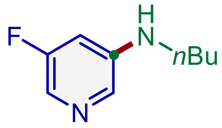

N-Butyl-5-fluoropyridin-3-amine (44): yellow oil; ^{1}H NMR (400 MHz, $CDCl_3$) δ 7.84 - 7.78 (m, 2H), 6.59 - 6.48 (m, 1H), 3.96 (br, 1H), 3.10 (t, *J* = 7.1 Hz, 2H), 1.68 - 1.60 (m, 2H), 1.49 - 1.38 (m, 2H), 0.97 (t, *J* = 7.3 Hz, 3H); ^{13}C NMR (100 MHz, $CDCl_3$) δ 160.7 (d, *J* = 256.4 Hz), 146.1, 132.1, 125.6 (d, *J* = 24.0 Hz), 104.8 (d, *J* = 21.9 Hz), 43.2, 31.2, 20.2, 13.8; ^{19}F NMR (376 MHz, $CDCl_3$) δ -127.8 (s, F); HRMS (ESI) m/z calc. for $C_9H_{14}FN_2$ $[M+H]^+$: 169.1136, found: 169.1134.

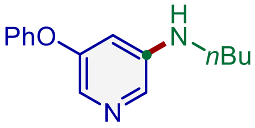

N-butyl-5-phenoxypyridin-3-amine (45): yellow oil; ^{1}H NMR (400 MHz, $CDCl_3$) δ 7.78 (s, 1H), 7.70 (s, 1H), 7.34 (t, *J* = 7.9 Hz, 2H), 7.12 (t, *J* = 7.4 Hz, 1H), 7.02 (d, *J* = 7.9 Hz, 2H), 6.49 (s, 1H), 3.80 (br, 1H), 3.07 (t, *J* = 7.1 Hz, 2H), 1.68 - 1.51 (m, 2H), 1.48 - 1.32 (m, 2H), 0.94 (t, *J* = 7.3 Hz, 3H); ^{13}C NMR (100 MHz, $CDCl_3$) δ 156.7, 154.5, 145.7, 131.0, 129.8 129.5, 123.6, 118.8, 108.4, 43.3, 31.4, 20.2, 13.8; HRMS (ESI) m/z calc. for $C_{15}H_{19}N_2O$ $[M+H]^+$: 243.1492, found: 243.1490.

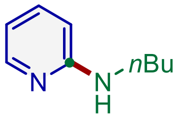

N-Butylpyridin-2-amine (46): yellow oil; ^{1}H NMR (400 MHz, $CDCl_3$) δ 8.05 (d, *J* = 4.3 Hz, 1H), 7.41 (t, *J* = 7.7 Hz, 1H), 6.60 - 6.48 (m, 1H), 6.37 (d, *J* = 8.4 Hz, 1H), 4.63 (br, 1H), 3.24 (t, *J* = 6.9 Hz, 2H), 1.67 - 1.54 (m, 2H), 1.49 - 1.37 (m, 2H), 0.95 (t, *J* = 7.3 Hz, 3H); ^{13}C NMR (100 MHz, $CDCl_3$) δ 158.9, 148.0, 137.5, 112.5, 106.3, 42.0, 31.6, 20.2, 13.8; HRMS (ESI) m/z calc. for $C_9H_{15}N_2$ $[M+H]^+$: 151.1230, found: 151.1233.

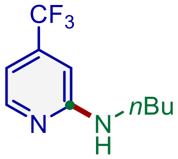

***N*-Butyl-4-(trifluoromethyl)pyridin-2-amine (47):** yellow oil; ^{1}H NMR (400 MHz, $CDCl_3$) δ 8.18 (d, *J* = 5.2 Hz, 1H), 6.71 (d, *J* = 5.2 Hz, 1H), 6.52 (s, 1H), 4.87 (br, 1H), 3.35 - 3.28 (m, 2H), 1.66 - 1.55 (m, 2H), 1.50 - 1.37 (m, 2H), 0.96 (t, *J* = 7.3 Hz, 3H); ^{13}C NMR (150 MHz, $CDCl_3$) δ 159.1, 149.4, 139.7 (q, *J* = 31.5 Hz), 107.8, 102.0, 100.0, 41.9, 31.4, 20.13, 13.8; ^{19}F NMR (376 MHz, $CDCl_3$) δ -61.1 (s, CF_3); HRMS (ESI) m/z calc. for $C_{10}H_{14}F_3N_2$ $[M+H]^+$: 219.1104, found: 219.1107.

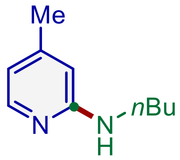

***N*-Butyl-4-methylpyridin-2-amine (48):** yellow oil; ^{1}H NMR (400 MHz, $CDCl_3$) δ 7.81 (s, 1H), 7.18 (d, *J* = 9.5 Hz, 1H), 6.25 (d, *J* = 8.4 Hz, 1H), 4.37 (s, 1H), 3.14 (t, *J* = 7.0 Hz, 2H), 2.09 (s, 3H), 1.56 - 1.47 (m, 2H), 1.41 - 1.29 (m, 2H), 0.88 (t, *J* = 7.3 Hz, 3H); ^{13}C NMR (100 MHz, $CDCl_3$) δ 157.4, 147.7, 138.7, 121.4, 106.1, 42.4, 31.9 20.4 17.5 14.0. HRMS (ESI) m/z calc. for $C_{10}H_{17}N_2$ $[M+H]^+$: 165.1386, found: 165.1390.

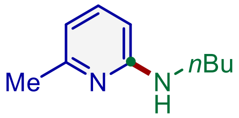

***N*-Butyl-6-methylpyridin-2-amine (49):** yellow oil; ^{1}H NMR (400 MHz, $CDCl_3$) δ 7.33 (t, *J* = 7.8 Hz, 1H), 6.43 (d, *J* = 7.3 Hz, 1H), 6.18 (d, *J* = 8.3 Hz, 1H), 4.51 (br, 1H), 3.24 - 3.10 (m, 2H), 2.35 (s, 3H), 1.67 - 1.52 (m, 2H), 1.50 - 1.36 (m, 2H), 0.95 (t, *J* = 7.3 Hz, 3H); ^{13}C NMR (100 MHz, $CDCl_3$) δ 159.8, 158.1, 139.1, 113.2, 103.3, 43.4, 32.8, 25.4, 21.3, 15.0; HRMS (ESI) m/z calc. for $C_{10}H_{17}N_2$ $[M+H]^+$: 165.1386, found: 165.1389.

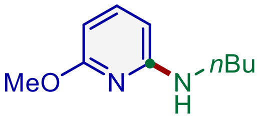

***N*-Butyl-6-methoxypyridin-2-amine (50):** yellow oil; ^{1}H NMR (400 MHz, $CDCl_3$) δ 7.34 (t, *J* = 7.9 Hz, 1H), 6.00 (d, *J* = 7.9 Hz, 1H), 5.92 (d, *J* = 7.9 Hz, 1H), 4.36 (br, 1H), 3.84 (s, 3H), 3.23 (m, 2H), 1.65 - 1.55 (m, 2H), 1.49 - 1.37 (m, 2H), 0.95 (t, *J* = 7.3 Hz, 3H); ^{13}C NMR (100 MHz, $CDCl_3$) δ 164.8, 159.1, 141.1, 98.4, 98.2, 54.5, 43.2, 32.9, 21.4, 15.0; HRMS (ESI) m/z calc. for $C_{10}H_{17}N_2O$ $[M+H]^+$: 181.1335, found: 181.1338.

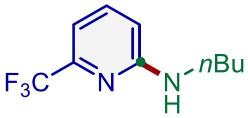

***N*-Butyl-6-(trifluoromethyl)pyridin-2-amine (51):** yellow oil; ^{1}H NMR (400 MHz, $CDCl_3$) δ 7.51 (t, *J* = 7.9 Hz, 1H), 6.89 (d, *J* = 7.3 Hz, 1H), 6.50 (d, *J* = 8.5 Hz, 1H), 4.77 (br, 1H), 3.33 - 3.24 (m, 2H), 1.64 - 1.55 (m, 2H), 1.47 - 1.37 (m, 2H), 0.95 (t, *J* = 7.3 Hz, 3H); ^{13}C NMR (100 MHz, $CDCl_3$) δ 158.9, 146.74 (q, *J* = 33.8 Hz), 138.3, 121.80 (q, *J* = 274.0 Hz), 109.2, 108.76 (q, *J* = 3.2 Hz), 42.0, 31.6, 20.2, 13.9; ^{19}F NMR (376 MHz, $CDCl_3$) δ -68.8 (s, CF_3); HRMS (ESI) m/z calc. for $C_{10}H_{14}F_3N_2$ $[M+H]^+$: 219.1104, found: 219.1107.

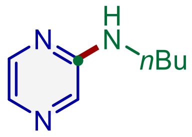

***N*-Butylpyrazin-2-amine (52):** yellow oil; ^{1}H NMR (400 MHz, $CDCl_3$) δ 7.99 (dd, *J* = 2.6, 1.5 Hz, 1H), 7.89 (d, *J* = 1.3 Hz, 1H), 7.80 (d, *J* = 2.8 Hz, 1H), 4.67 (s, 1H), 3.38 - 3.30 (m, 2H), 1.71 - 1.56 (m, 2H), 1.45 (dq, *J* = 14.4, 7.3 Hz, 2H), 0.98 (t, *J* = 7.3 Hz, 3H); ^{13}C NMR (100 MHz, $CDCl_3$) δ 154.8, 142.1, 132.6, 131.8, 41.4, 31.6, 20.2, 13.9; HRMS (ESI) m/z calc. for $C_8H_{14}N_3$ $[M+H]^+$: 152.1182, found: 152.1180.

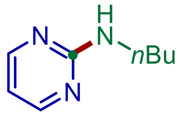

***N*-Butylpyrimidin-2-amine (53):** yellow oil; ^{1}H NMR (400 MHz, $CDCl_3$) ^{1}H NMR (400 MHz, $CDCl_3$) δ 8.18 (d, *J* = 4.4 Hz, 2H), 6.41 (t, *J* = 4.6 Hz, 1H), 5.54 (br, 1H), 3.36 - 3.27 (m, 2H), 1.62 - 1.43 (m, 2H), 1.39 -1.27 (m, 2H), 0.86 (t, *J* = 7.3 Hz, 3H); ^{13}C NMR (100 MHz, $CDCl_3$) δ 162.5, 157.9, 110.1, 41.2, 31.7, 20.1, 13.8; HRMS (ESI) m/z calc. for $C_8H_{14}N_3$ $[M+H]^+$: 152.1182, found: 152.1185.

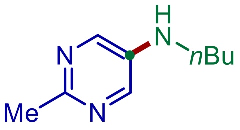

***N*,2-Dimethylpyrimidin-5-amine (54):** yellow oil; ^{1}H NMR (400 MHz, $CDCl_3$) δ 7.97 (s, 2H), 3.67 (br, 1H), 3.05 (t, *J* = 7.1 Hz, 2H), 2.52 (s, 3H), 1.59 - 1.50 (m, 2H), 1.41 - 1.34 (m, 2H), 0.89 (t, *J* = 7.3 Hz, 3H); ^{13}C NMR (100 MHz, $CDCl_3$) δ 156.6, 141.1, 139.6, 43.1, 31.3, 24.5, 20.1, 13.8; HRMS (ESI) m/z calc. for $C_9H_{16}N_3$ $[M+H]^+$: 166.1339, found: 166.1342.

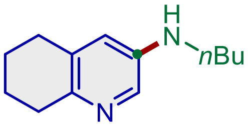

N-butyl-5,6,7,8-tetrahydroquinolin-3-amine (55): yellow oil; ^{1}H NMR (400 MHz, CDCl$_3$) δ 7.81 (d, J = 2.7 Hz, 1H), 6.59 (d, J = 2.5 Hz, 1H), 3.25 (br, 1H), 3.09 (t, J = 7.1 Hz, 2H), 2.80 (t, J = 6.4 Hz, 2H), 2.69 (t, J = 6.2 Hz, 2H), 1.89 - 1.77 (m, 2H), 1.82 - 1.71 (m, 2H), 1.68 - 1.57 (m, 2H), 1.46 - 1.38 (m, 2H), 0.95 (t, J = 7.3 Hz, 3H); ^{13}C NMR (100 MHz, CDCl$_3$) δ 145.9, 142.5, 133.5, 132.1, 119.6, 43.7, 31.5, 31.2, 29.1, 23.5, 22.9, 20.2, 13.9; HRMS (ESI) m/z calc. for $C_{13}H_{21}N_2$ [M+H]$^+$: 205.1699, found: 205.1696.

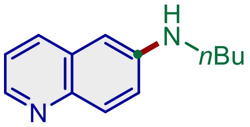

N-butylquinolin-6-amine (56): yellow oil; ^{1}H NMR (400 MHz, CDCl$_3$) δ 8.61 - 8.57 (m, 1H), 7.91 - 7.86 (m, 2H), 7.34 - 7.18 (m, 1H), 7.07 (dd, J = 9.0, 2.6 Hz, 1H), 6.67 (d, J = 2.5 Hz, 1H), 3.96 (br, 1H), 3.20 (t, J = 7.1 Hz, 2H), 1.79 - 1.58 (m, 2H), 1.53 - 1.47 (m, 2H), 0.98 (t, J = 7.3 Hz, 3H); ^{13}C NMR (100 MHz, CDCl$_3$) δ 146.4, 145.9, 143.1, 133.7, 130.2,130.1, 121.4, 121.3, 102.7, 43.7, 31.4, 20.4, 13.9; HRMS (ESI) m/z calc. for $C_{13}H_{17}N_2$ [M+H]$^+$: 201.1386, found: 201.1389.

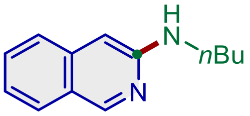

N-butylisoquinolin-3-amine (57): yellow oil; ^{1}H NMR (400 MHz, CDCl$_3$) δ 8.74 (s, 1H), 7.66 (d, J = 8.2 Hz, 1H), 7.46 (d, J = 8.3 Hz, 1H), 7.39 (t, J = 7.3 Hz, 1H), 7.11 (t, J = 7.3 Hz, 1H), 6.41 (s, 1H), 4.55 (br, 1H), 3.19 (s, 2H), 1.68 - 1.56 (m, 3H), 1.34 - 1.27 (m, 2H), 0.91 (t, J = 7.3 Hz, 3H); ^{13}C NMR (100 MHz, CDCl$_3$) δ 155.7, 151.8, 139.1, 130.4, 127.9, 124.8, 123.5, 122.3, 95.3, 42.8, 31.4, 20.3, 13.9; HRMS (ESI) m/z calc. for $C_{13}H_{17}N_2$ [M+H]$^+$: 201.1386, found: 201.1386.

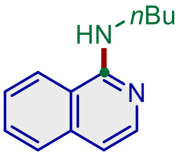

N-butylisoquinolin-1-amine (57): yellow oil; ^{1}H NMR (400 MHz, CDCl$_3$) δ 8.00 (d, J = 5.7 Hz, 1H), 7.74 (d, J = 8.3 Hz, 1H), 7.67 (d, J = 8.1 Hz, 1H), 7.57 (t, J = 7.5 Hz, 1H), 7.45 (t, J = 7.6 Hz, 1H), 6.91 (d, J = 5.8 Hz, 1H), 5.20 (br, 1H), 3.67 - 3.58 (m, 2H), 1.79 - 1.65 (m, 2H), 1.57 - 1.46 (m, 2H), 1.09 - 0.91 (m, 3H); ^{13}C NMR (100 MHz, CDCl$_3$) δ 141.4, 129.6, 127.2, 125.8, 121.3, 110.6, 41.7, 31.8, 20.4, 14.0; HRMS (ESI) m/z calc. for $C_{13}H_{17}N_2$ [M+H]$^+$: 201.1386, found: 201.1383.

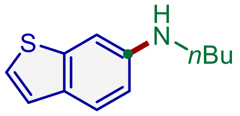

N-Butylbenzo[b]thiophen-6-amine (59): yellow oil; ^{1}H NMR (400 MHz, $CDCl_3$) δ 7.62 (d, J = 8.6 Hz, 1H), 7.37 (d, J = 5.4 Hz, 1H), 7.18 (d, J = 5.4 Hz, 1H), 6.99 (d, J = 2.1 Hz, 1H), 6.73 (dd, J = 8.6, 2.2 Hz, 1H), 3.56 (br, 1H), 3.18 (t, J = 7.1 Hz, 2H), 1.70 - 1.61 (m, 2H), 1.51 - 1.43 (m, 2H), 0.99 (t, J = 7.3 Hz, 3H); ^{13}C NMR (100 MHz, $CDCl_3$) δ 146.1, 141.1, 129.1, 126.8, 123.4, 122.8, 114.0, 104.8, 44.2, 31.7, 20.4, 14.0; HRMS (ESI) m/z calc. for $C_{12}H_{16}NS$ $[M+H]^+$: 206.0998, found: 206.0996.

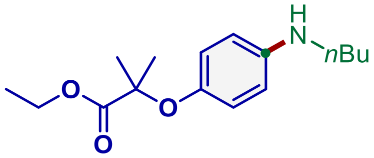

Ethyl 2-(4-(butylamino)phenoxy)-2-methylpropanoate (50): yellow oil; ^{1}H NMR (400 MHz, $CDCl_3$) δ 6.77 (d, J = 8.9 Hz, 2H), 6.49 (d, J = 8.9 Hz, 2H), 4.28 – 4.22 (m, 2H), 3.05 (t, J = 7.1 Hz, 2H), 1.61 – 1.54 (m, 2H), 1.51 (s, 6H), 1.45 – 1.40 (m, 2H), 1.29 (t, J = 7.1 Hz, 3H), 0.95 (t, J = 7.3 Hz, 3H); ^{13}C NMR (150 MHz, $CDCl_3$) δ 174.5, 146.6, 144.6, 122.3, 113.1, 79.7, 61.2, 44.3, 41.3, 31.8, 25.3, 20.3, 14.2; HRMS (ESI) m/z calc. for $C_{16}H_{26}NO_3$ $[M+H]^+$: 280.1907, found: 280.1909.

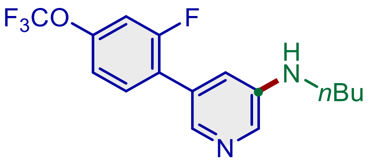

N-butyl-5-(2-fluoro-4-(trifluoromethoxy)phenyl)pyridin-3-amine (51): yellow oil; ^{1}H NMR (400 MHz, $CDCl_3$) δ 8.04 (d, J = 9.1 Hz, 2H), 7.46 (t, J = 8.4 Hz, 1H), 7.18 - 7.02 (m, 2H), 6.99 (s, 1H), 3.85 (br, 1H), 3.16 (t, J = 7.0 Hz, 2H), 1.77 - 1.58 (m, 2H), 1.57 - 1.35 (m, 2H), 0.97 (t, J = 7.3 Hz, 3H); ^{13}C NMR (100 MHz, $CDCl_3$) δ 159.7 (d, J = 251.4 Hz), 149.27 (d, J = 12.8 Hz), 144.1, 138.0, 135.4, 131.28 (d, J = 4.6 Hz), 130.6, 125.2 (d, J = 14.0 Hz), 120.4 (q, J = 258.4 Hz), 118.4 (q, J = 3.2 Hz), 116.9 (d, J = 3.1 Hz), 109.6 (d, J = 26.6 Hz), 43.3, 31.4, 20.2, 13.8; ^{19}F NMR (376 MHz, $CDCl_3$) δ -58.01 (s, CF_3), -113.04 (s, F); HRMS (ESI) m/z calc. for $C_{16}H_{17}F_4N_2O$ $[M+H]^+$: 329.1272, found: 329.1279.

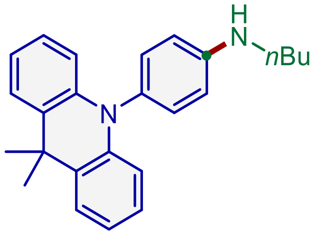

N-Butyl-4-(9,9-dimethylacridin-10(9H)-yl)anilinee (62): yellow oil; ^{1}H NMR (400 MHz, $CDCl_3$) δ 7.51 (d, *J* = 7.9 Hz, 2H), 7.16 (d, *J* = 7.6 Hz, 2H), 7.04 (d, *J* = 7.6 Hz, 2H), 6.98 (d, *J* = 7.7 Hz, 2H), 6.85 (d, *J* = 7.7 Hz, 2H), 6.47 (d, *J* = 8.2 Hz, 2H), 3.86 (br, 1H), 3.26 (t, *J* = 7.0 Hz, 2H), 1.75 (s, 6H), 1.72 (d, *J* = 7.4 Hz, 2H), 1.63 - 1.54 (m, 2H), 1.08 (t, *J* = 7.3 Hz, 3H); ^{13}C NMR (150 MHz, $CDCl_3$) δ 148.1, 141.6, 131.8, 130.9, 130.2, 129.9, 126.3, 125.1, 120.2, 114.2, 43.8, 36.0, 31.8, 31.3, 20.4, 14.0; HRMS (ESI) m/z calc. for $C_{25}H_{29}N_2$ $[M+H]^+$: 357.2325, found: 357.2331.

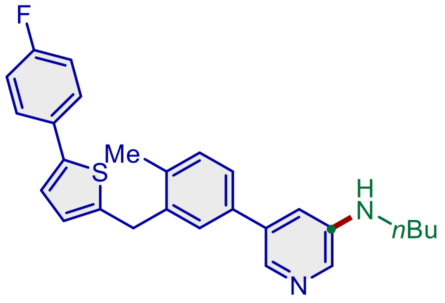

N-butyl-5-(3-((5-(4-fluorophenyl)thiophen-2-yl)methyl)-4-methylphenyl)pyridin-3-amine (63): yellow oil; ^{1}H NMR (400 MHz, $CDCl_3$) δ 8.16 (s, 1H), 7.97 (s, 1H), 7.52 - 7.33 (m, 4H), 7.26 (d, *J* = 6.4 Hz, 1H), 7.10 - 7.00 (m, 4H), 6.70 (d, *J* = 3.3 Hz, 1H), 4.18 (s, 2H), 3.75 (br, 1H), 3.16 (t, *J* = 7.0 Hz, 2H), 2.37 (s, 3H), 1.70 - 1.53 (m, 2H), 1.48 - 1.36 (m, 2H), 0.96 (t, *J* = 7.3 Hz, 3H); ^{13}C NMR (100 MHz, $CDCl_3$) δ 162.1 (d, *J* = 246.7 Hz), 144.4, 143.1, 141.7, 138.8, 137.0, 136.7, 136.5, 136.2, 134.5, 131.1, 130.8 (d, *J* = 3.4 Hz), 128.3, 127.1 (d, *J* = 8.0 Hz), 126.1, 125.7, 122.7, 116.7, 115.7 (d, *J* = 21.8 Hz), 43.3, 34.2, 31.5, 20.2, 19.2, 13.9; ^{19}F NMR (376 MHz, $CDCl_3$) δ -115.10 (s, F); HRMS (ESI) m/z calc. for $C_{27}H_{28}FN_2S$ $[M+H]^+$: 431.1952, found: 431.1958.

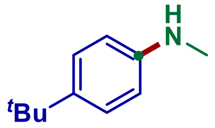

4-(Tert-butyl)-N-methylaniline (64): yellow oil; ^{1}H NMR (400 MHz, $CDCl_3$) δ 7.22 (d, *J* = 8.5 Hz, 2H), 6.58 (d, *J* = 8.6 Hz, 2H), 2.81 (s, 3H), 1.28 (s, 9H); ^{13}C NMR (100 MHz, $CDCl_3$) δ 147.2, 140.2, 126.1, 112.4, 34.0, 31.7, 31.1; HRMS (ESI) m/z calc. for $C_{11}H_{18}N$ $[M+H]^+$: 164.1434, found: 164.1435.

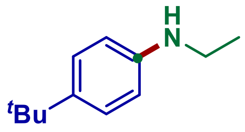

4-(Tert-butyl)-N-ethylaniline (65): yellow oil; ^{1}H NMR (400 MHz, $CDCl_3$) δ 7.23 (d, J = 8.5 Hz, 2H), 6.60 (d, J = 8.5 Hz, 2H), 3.17 (q, J = 7.1 Hz, 2H), 1.31 (s, 9H), 1.27 (t, J = 7.5 Hz, 2H); ^{13}C NMR (100 MHz, $CDCl_3$) δ 146.3, 140.1, 126.1, 112.7, 38.9, 30.0, 31.7, 15.2; HRMS (ESI) m/z calc. for $C_{12}H_{20}N$ $[M+H]^+$: 178.1590, found: 178.1593.

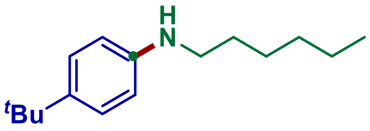

4-(Tert-butyl)-N-hexylaniline (66): yellow oil; ^{1}H NMR (400 MHz, $CDCl_3$) δ 7.21 (d, J = 8.5 Hz, 2H), 6.57 (d, J = 8.5 Hz, 2H), 3.10 (t, J = 7.1 Hz, 2H), 1.68 - 1.54 (m, 2H), 1.45 - 1.38 (m, 2H), 1.36 - 1.31 (m, 4H), 1.28 (d, J = 6.2 Hz, 9H), 0.91 (t, J = 6.5 Hz, 3H); ^{13}C NMR (100 MHz, $CDCl_3$) δ 146.2, 139.9, 126.0, 112.5, 44.3, 33.8, 31.7, 31.6, 29.7, 26.9, 22.7, 14.1; HRMS (ESI) m/z calc. for $C_{16}H_{28}N$ $[M+H]^+$: 234.2216, found: 234.2220.

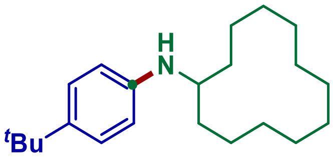

4-(Tert-butyl)-N-dodecylaniline (67): yellow oil; ^{1}H NMR (400 MHz, $CDCl_3$) δ 7.19 (d, J = 8.5 Hz, 2H), 6.55 (d, J = 8.5 Hz, 2H), 3.07 (t, J = 7.1 Hz, 2H), 1.64 - 1.53 (m, 2H), 1.41 - 1.21 (m, 27H), 0.88 (t, J = 6.6 Hz, 3H); ^{13}C NMR (100 MHz, $CDCl_3$) δ 146.4, 139.9, 126.1, 112.6, 44.4, 33.9, 32.1, 31.7, 29.8, 29.8, 29.8, 29.8, 29.5, 27.4, 22.8, 14.3; HRMS (ESI) m/z calc. for $C_{22}H_{38}N$ $[M+H]^+$: 316.2999, found: 316.3002.

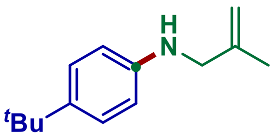

4-(Tert-butyl)-N-(2-methylallyl)aniline (68): yellow oil; ^{1}H NMR (400 MHz, $CDCl_3$) δ 7.20 (d, J = 8.6 Hz, 2H), 6.57 (d, J = 8.6 Hz, 2H), 4.50 - 4.86 (m, 2H), 3.67 (s, 2H), 1.79 (s, 3H), 1.28 (s, 9H); ^{13}C NMR (100 MHz, $CDCl_3$) δ 146.0, 143.1, 140.1, 125.9, 112.54, 110.8, 50.3, 33.8, 31.6, 20.5; HRMS (ESI) m/z calc. for $C_{14}H_{22}N$ $[M+H]^+$: 204.1747, found: 204.1750.

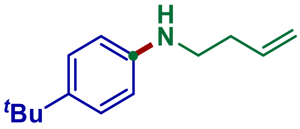

N-(But-3-en-1-yl)-4-(tert-butyl)aniline (69): yellow oil; ^{1}H NMR (400 MHz, $CDCl_3$) δ 7.22 (d, *J* = 8.5 Hz, 2H), 6.59 (d, *J* = 8.5 Hz, 2H), 5.90 - 5.76 (m, 1H), 5.19 - 5.10 (m, 2H), 3.19 (t, *J* = 6.7 Hz, 2H), 2.39 (q, *J* = 6.7 Hz, 2H), 1.29 (s, 9H); ^{13}C NMR (100 MHz, $CDCl_3$) δ 146.1, 140.3, 136.1, 126.1, 117.1, 112.8, 43.3, 34.0, 33.9, 31.7; HRMS (ESI) m/z calc. for $C_{14}H_{22}N$ $[M+H]^+$: 204.1747, found: 204.1751.

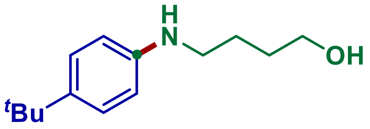

4-((4-(Tert-butyl)phenyl)amino)butan-1-ol (70): yellow oil; ^{1}H NMR (400 MHz, $CDCl_3$) δ 7.20 (d, *J* = 8.5 Hz, 2H), 6.58 (d, *J* = 8.5 Hz, 2H), 3.65 (t, *J* = 5.7 Hz, 2H), 3.11 (d, *J* = 6.3 Hz, 2H), 2.85 (br, 2H), 1.75 - 1.60 (m, 4H), 1.27 (s, 9H); ^{13}C NMR (100 MHz, $CDCl_3$) δ 146.0, 140.4, 126.0, 112.9, 62.6, 44.3, 33.9 31.6, 30.5, 26.3; HRMS (ESI) m/z calc. for $C_{14}H_{24}NO$ $[M+H]^+$: 222.1852, found: 222.1856.

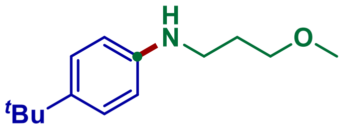

4-(Tert-butyl)-N-(3-methoxypropyl)aniline (71): yellow oil; ^{1}H NMR (400 MHz, $CDCl_3$) δ 7.19 (d, *J* = 8.7 Hz, 2H), 6.56 (d, *J* = 8.7 Hz, 2H), 3.49 (t, *J* = 6.0 Hz, 2H), 3.34 (s, 3H), 3.20 (t, *J* = 6.6 Hz, 2H), 1.87 (t, *J* = 6.2 Hz, 2H), 1.27 (s, 9H); ^{13}C NMR (100 MHz, $CDCl_3$) δ 146.2, 139.9, 126.0, 112.5, 71.3, 58.8, 42.0, 33.9, 31.6, 29.5; HRMS (ESI) m/z calc. for $C_{14}H_{24}NO$ $[M+H]^+$: 222.1852, found: 222.1855.

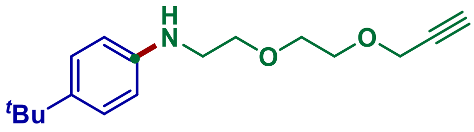

4-(Tert-butyl)-N-(2-(2-(prop-2-yn-1-yloxy)ethoxy)ethyl)aniline (72): yellow oil; ^{1}H NMR (400 MHz, $CDCl_3$) δ 7.17 (d, *J* = 7.4 Hz, 2H), 6.57 (d, *J* = 7.4 Hz, 2H), 4.18 (s, 2H), 3.71 - 3.62 (m, 6H), 3.27 (t, *J* = 4.4 Hz, 2H), 2.41 (s, 1H), 1.25 (d, *J* = 1.2 Hz, 9H); ^{13}C NMR (100 MHz, $CDCl_3$) δ 145.9, 140.3, 126.0, 112.9, 79.6, 74.6, 70.1, 69.8, 69.1, 58.5, 43.7, 33.9, 31.6; HRMS (ESI) m/z calc. for $C_{17}H_{26}NO_2$ $[M+H]^+$: 276.1958, found: 276.1964.

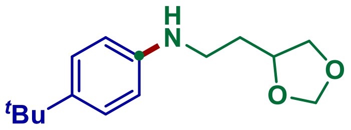

N-(2-(1,3-Dioxolan-4-yl)ethyl)-4-(tert-butyl)aniline (73): yellow oil; ^{1}H NMR (400 MHz, $CDCl_3$) δ 7.20 (d, J = 8.5 Hz, 2H), 6.58 (d, J = 8.5 Hz, 2H), 4.98 (t, J = 4.4 Hz, 1H), 3.98 (t, J = 6.9 Hz, 2H), 3.91 - 3.83 (m, 2H), 3.25 (t, J = 6.5 Hz, 2H), 2.03 - 1.97 (m, 2H), 1.27 (s, 9H); ^{13}C NMR (100 MHz, $CDCl_3$) δ 146.2, 140.2, 126.1, 112.7, 103.9, 65.0, 39.6, 34.0, 33.7, 33.2, 31.7; HRMS (ESI) m/z calc. for $C_{15}H_{24}NO_2$ $[M+H]^+$: 250.1802, found: 250.1805.

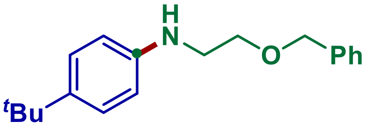

N-(2-(Benzyloxy)ethyl)-4-(tert-butyl)aniline (74): yellow oil; ^{1}H NMR (400 MHz, $CDCl_3$) δ 7.35 - 7.18 (m, 5H), 7.13 (d, J = 8.5 Hz, 2H), 6.51 (d, J = 8.5 Hz, 2H), 4.47 (s, 2H), 3.62 (t, J = 5.2 Hz, 2H), 3.24 (t, J = 5.2 Hz, 2H), 1.20 (s, 9H); ^{13}C NMR (100 MHz, $CDCl_3$) δ 145.8, 140.4, 138.1, 128.5, 127.8, 127.7, 126.0, 113.0, 73.1, 68.8, 43.9, 33.9, 31.6; HRMS (ESI) m/z calc. for $C_{19}H_{26}NO$ $[M+H]^+$: 284.2009, found: 284.2013.

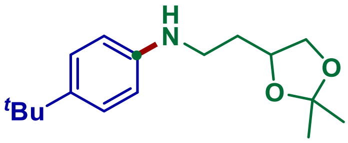

4-(Tert-butyl)-N-((2,2-dimethyl-1,3-dioxolan-4-yl)methyl)aniline (75): yellow oil; ^{1}H NMR (400 MHz, $CDCl_3$) δ 7.22 (d, J = 8.6 Hz, 2H), 6.60 (d, J = 8.6 Hz, 2H), 4.42 - 4.32 (m, 1H), 4.18 - 4.10 (m, 1H), 3.85 - 3.73 (m, 1H), 3.29 - 3.20 (m, 2H), 1.46 (s, 3H), 1.38 (s, 3H), 1.28 (s, 9H); ^{13}C NMR (100 MHz, $CDCl_3$) δ 145.6, 140.7, 126.1, 112.8, 109.5, 74.6, 67.3, 47.0, 33.9, 31.5, 26.9, 25.4; HRMS (ESI) m/z calc. for $C_{17}H_{28}NO_2$ $[M+H]^+$: 278.2115, found: 278.2117.

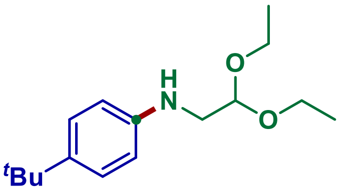

4-(Tert-butyl)-N-(2,2-diethoxyethyl)aniline (76): yellow oil; ^{1}H NMR (400 MHz, $CDCl_3$) δ 7.18 (d, J = 8.6 Hz, 2H), 6.55 (d, J = 8.6 Hz, 2H), 3.21 - 3.09 (m, 1H), 2.15 - 2.10 (m, 2H), 1.85 - 1.71 (m, 2H), 1.28 (s, 9H), 1.18 - 1.02 (m, 4H), 0.92 (d, J = 6.5 Hz, 3H); ^{13}C NMR (100 MHz, $CDCl_3$) δ 145.1, 139.7, 126.0, 112.9, 52.4, 34.2, 33.9, 33.7, 32.4, 31.6, 22.3; HRMS (ESI) m/z calc. for $C_{16}H_{28}NO_2$ $[M+H]^+$: 266.2115, found: 266.2119.

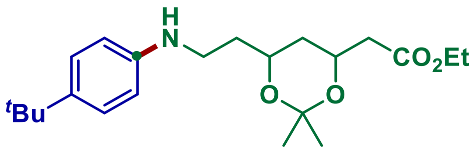

Tert-butyl2-(6-(2-((4-(tert-butyl)phenyl)amino)ethyl)-2,2-dimethyl-1,3-dioxan-4-yl)acetate (77): yellow oil; ^{1}H NMR (400 MHz, $CDCl_3$) δ 7.21 (d, J = 8.6 Hz, 2H), 6.56 (d, J = 8.6 Hz, 2H), 4.32 - 3.99 (m, 2H), 3.32 - 3.13 (m, 2H), 2.48 - 2.25 (m, 2H), 1.77 (d, J = 6.2 Hz, 2H), 1.59 - 1.53 (m, 1H), 1.45 (s, 12H), 1.40 (s, 3H), 1.29 (s, 9H), 1.11 - 0.90 (m, 2H); ^{13}C NMR (100 MHz, $CDCl_3$) δ 170.2, 146.2, 139.9, 125.9, 112.5, 98.7, 80.6, 68.0, 66.2, 42.7, 41.0, 36.4, 35.7, 33.8, 31.5, 30.1, 28.1, 19.8; HRMS (ESI) m/z calc. for $C_{22}H_{36}NO_4$ $[M+H]^+$: 378.2639, found: 378.2645.

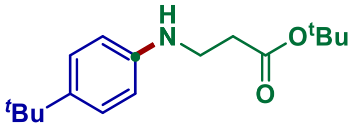

Tert-butyl 3-((4-(tert-butyl)phenyl)amino)propanoate (78): yellow oil; ^{1}H NMR (400 MHz, $CDCl_3$) δ 7.21 (d, J = 8.3 Hz, 2H), 6.59 (d, J = 8.3 Hz, 2H), 3.39 (t, J = 6.3 Hz, 2H), 2.52 (t, J = 6.3 Hz, 2H), 1.46 (s, 9H), 1.28 (s, 9H); ^{13}C NMR (100 MHz, $CDCl_3$) δ 171.8, 145.4, 140.5, 126.6, 112.9, 80.8, 40.0, 35.3, 33.9, 31.9, 28.1; HRMS (ESI) m/z calc. for $C_{17}H_{28}NO_2$ $[M+H]^+$: 278.2115, found: 278.2110.

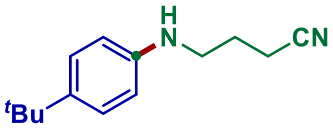

Tert-butyl 4-((4-(tert-butyl)phenyl)amino)butanoate (79): yellow oil; ^{1}H NMR (400 MHz, $CDCl_3$) δ 7.51 (d, J = 8.8 Hz, 2H), 7.38 (d, J = 8.8 Hz, 2H), 3.85 (t, J = 7.0 Hz, 2H), 2.60 (t, J = 8.1 Hz, 2H), 2.22 - 2.09 (m, 2H), 1.31 (s, 9H); ^{13}C NMR (100 MHz, $CDCl_3$) δ 174.1, 147.5, 136.8, 125.7, 119.9, 48.9, 34.4, 32.7, 31.3, 18.1; HRMS (ESI) m/z calc. for $C_{14}H_{21}N_2$ $[M+H]^+$: 217.1699, found: 217.1696.

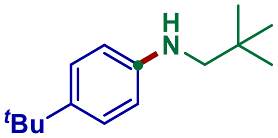

4-(Tert-butyl)-N-neopentylaniline (80): yellow oil; ^{1}H NMR (400 MHz, $CDCl_3$) δ 7.22 (d, J = 8.7 Hz, 2H), 6.60 (d, J = 8.7 Hz, 2H), 3.55 (br, 1H), 2.90 (s, 2H), 1.29 (d, J = 6.7 Hz, 9H), 1.01

(s, 9H); ^{13}C NMR (100 MHz, $CDCl_3$) δ 146.8, 139.7, 126.0, 112.4, 56.2, 33.8, 31.7, 31.5, 27.7; HRMS (ESI) m/z calc. for $C_{15}H_{26}N$ [M+H]$^+$: 220.2060, found: 220.2064.

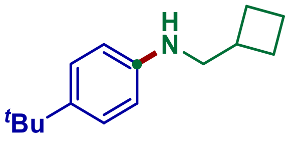

4-(Tert-butyl)-N-(cyclobutylmethyl)aniline (81): yellow oil; ^{1}H NMR (400 MHz, $CDCl_3$) δ 7.15 - 7.05 (m, 2H), 6.46 (d, *J* = 8.1 Hz, 2H), 3.01 (d, *J* = 7.3 Hz, 2H), 2.55 - 2.40 (m, 1H), 2.07 - 1.95 (m, 2H), 1.91 - 1.73 (m, 2H), 1.70 - 1.56 (m, 2H), 1.18 (s, 9H); ^{13}C NMR (100 MHz, $CDCl_3$) δ 146.3, 140.0, 126.0, 112.5, 50.1, 35.1, 33.9, 31.6, 26.1, 18.6; HRMS (ESI) m/z calc. for $C_{15}H_{24}N$ [M+H]$^+$: 218.1903, found: 218.1906.

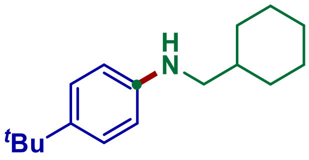

4-(Tert-butyl)-N-(cyclohexylmethyl)aniline (82): yellow oil; ^{1}H NMR (400 MHz, $CDCl_3$) δ 7.21 (d, *J* = 8.7 Hz, 2H), 6.57 (d, *J* = 8.7 Hz, 2H), 2.95 (d, *J* = 6.6 Hz, 2H), 1.83 (d, *J* = 13.1 Hz, 2H), 1.81 - 1.64 (m, 4H), 1.65 - 1.51 (m, 1H), 1.27 - 1.20 (m, 2H), 1.05 - 0.94 (m, 2H); ^{13}C NMR (100 MHz, $CDCl_3$) δ 146.4, 139.8, 126.1, 112.4, 51.0, 37.8, 33.9, 31.7, 31.5, 26.8, 26.1; HRMS (ESI) m/z calc. for $C_{17}H_{28}N$ [M+H]$^+$: 246.2216, found: 246.2220.

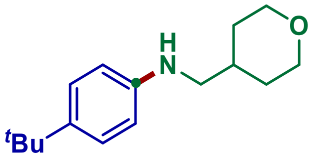

4-(Tert-butyl)-N-((tetrahydro-2H-pyran-4-yl)methyl)aniline (83): yellow oil; ^{1}H NMR (400 MHz, $CDCl_3$) δ 7.23 (d, *J* = 8.6 Hz, 2H), 6.58 (d, *J* = 8.6 Hz, 2H), 4.01 (dd, *J* = 10.9, 3.7 Hz, 2H), 3.41 (dd, *J* = 11.7, 9.9 Hz, 2H), 3.03 (d, *J* = 6.7 Hz, 2H), 1.93 - 1.79 (m, 1H), 1.79 - 1.67 (m, 2H), 1.41 - 1.34 (m, 2H), 1.30 (s, 9H); ^{13}C NMR (100 MHz, $CDCl_3$) δ 146.0, 140.0, 126.1, 112.4, 67.8, 50.3, 35.0, 33.9, 31.6, 31.2; HRMS (ESI) m/z calc. for $C_{16}H_{26}NO$ [M+H]$^+$: 248.2009, found: 248.2014.

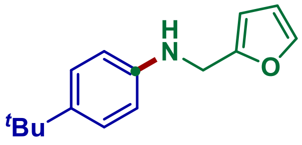

4-(Tert-butyl)-N-(furan-2-ylmethyl)aniline (84): yellow oil; ^{1}H NMR (400 MHz, $CDCl_3$) δ 7.36 (s, 1H), 7.22 (d, J = 8.5 Hz, 2H), 6.64 (d, J = 8.5 Hz, 2H), 6.32 (s, 1H), 6.23 (d, J = 3.0 Hz,

1H), 4.30 (s, 2H), 1.27 (s, 9H); ^{13}C NMR (100 MHz, $CDCl_3$) δ 153.0, 145.3, 141.9, 140.8, 126.0, 112.9, 110.3, 106.9, 41.7, 33.9, 31.5; HRMS (ESI) m/z calc. for $C_{15}H_{20}NO$ $[M+H]^+$: 230.1539, found: 230.1543.

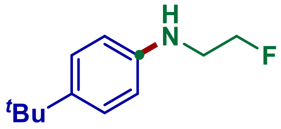

4-(Tert-butyl)-N-(2-fluoroethyl)aniline (85): yellow oil; ^{1}H NMR (400 MHz, $CDCl_3$) δ 7.24 (d, J = 8.6 Hz, 2H), 6.61 (d, J = 8.6 Hz, 2H), 4.70 - 4.54 (m, 2H), 3.89 (br, 1H), 3.49 - 3.39 (m, 2H), 1.29 (s, 9H); ^{13}C NMR (100 MHz, $CDCl_3$) δ 145.3, 141.1, 126.3, 113.1, 82.7 (d, J = 165 Hz), 44.6 (d, J = 20 Hz), 34.0, 31.7; ^{19}F NMR (376 MHz, $CDCl_3$) δ -221.72; HRMS (ESI) m/z calc. for $C_{12}H_{19}FN$ $[M+H]^+$: 196.1496, found: 196.1493.

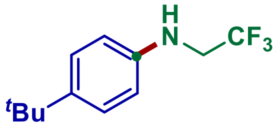

4-(Tert-butyl)-N-(2,2,2-trifluoroethyl)aniline (86): yellow oil; ^{1}H NMR (400 MHz, $CDCl_3$) δ 7.16 (d, J = 8.7 Hz, 2H), 6.56 (d, J = 8.6 Hz, 2H), 3.65 (q, J = 9.0 Hz, 2H), 1.20 (s, 9H); ^{13}C NMR (100 MHz, $CDCl_3$) δ 143.9, 142.0, 126.9, 125.1 (q, J = 278.6 Hz), 112.9, 46.3 (q, J = 33.4 Hz), 33.9, 31.5; ^{19}F NMR (376 MHz, $CDCl_3$) δ -62.13 (t, J = 10.9 Hz); HRMS (ESI) m/z calc. for $C_{12}H_{17}F_3N$ $[M+H]^+$: 232.1308, found: 232.1305.

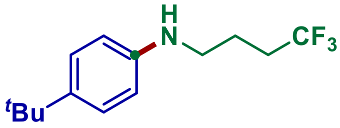

4-(Tert-butyl)-N-(4,4,4-trifluorobutyl)aniline (87): yellow oil; ^{1}H NMR (400 MHz, $CDCl_3$) δ 7.21 (d, J = 8.4 Hz, 2H), 6.56 (d, J = 8.4 Hz, 2H), 3.19 (t, J = 6.9 Hz, 2H), 2.27 - 2.12 (m, 2H), 1.93 - 1.79 (m, 2H), 1.28 (s, 9H); ^{13}C NMR (100 MHz, $CDCl_3$) δ 145.7, 140.7, 127.4 (q, J = 208 Hz), 126.3, 112.7, 43.1, 34.0, 31.7, 31.6 (q, J = 30 Hz), 22.4 (q, J =10 Hz); ^{19}F NMR (376 MHz, $CDCl_3$) δ -66.09 (t, J = 10.9 Hz); HRMS (ESI) m/z calc. for $C_{14}H_{21}F_3N$ $[M+H]^+$: 260.1621, found: 260.1624.

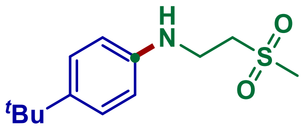

4-(Tert-butyl)-N-(2-(methylsulfonyl)ethyl)aniline (88): yellow oil; ^{1}H NMR (400 MHz, $CDCl_3$) δ 7.25 (d, J = 8.0 Hz, 2H), 6.59 (d, J = 7.7 Hz, 2H), 3.80 (s, 2H), 3.30 (s, 2H), 2.95 (s, 3H), 1.28 (s, 9H); ^{13}C NMR (100 MHz, $CDCl_3$) δ 145.5, 141.8, 126.2, 113.2, 54.1, 42.1, 37.9, 33.9, 31.5; HRMS (ESI) m/z calc. for $C_{13}H_{22}NO_2S$ $[M+H]^+$: 256.1366, found: 256.1370.

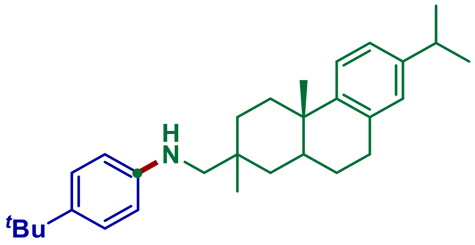

4-(Tert-butyl)-N-(((4aS)-7-isopropyl-2,4a-dimethyl-1,2,3,4,4a,9,10,10a-octahydrophenanthren-2-yl)methyl)aniline (89): yellow oil; ^{1}H NMR (400 MHz, $CDCl_3$) δ 7.21 (d, J = 8.6 Hz, 3H), 7.04 (s, 1H), 6.92 (s, 1H), 6.60 (d, J = 8.5 Hz, 2H), 3.55 (br, 1H), 3.12 - 3.06 (m, 1H), 2.95 - 2.81 (m, 4H), 2.39 - 2.42 (m, 1H), 1.85 - 1.75 (m, 3H), 1.74 - 1.61 (m, 2H), 1.55 - 1.44 (m, 3H), 1.30 (s, 9H), 1.32 - 1.21 (m, 9H), 1.04 (s, 3H); ^{13}C NMR (100 MHz, $CDCl_3$) δ 147.4, 146.6, 145.7, 139.8, 134.8, 126.9, 126.0, 124.3, 123.9, 112.5, 55.3, 45.3, 38.5, 37.5, 37.4, 36.3, 33.8, 33.5, 31.6, 30.1, 25.3, 24.0, 19.4, 18.9, 18.8; HRMS (ESI) m/z calc. for $C_{30}H_{44}N$ $[M+H]^+$: 418.3468, found: 418.3471.

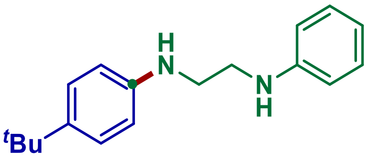

N1-(4-(Tert-butyl)phenyl)-N2-phenylethane-1,2-diamine (90): yellow oil; ^{1}H NMR (400 MHz, $CDCl_3$) δ 7.26 - 7.14 (m, 4H), 6.72 (t, *J* = 7.3 Hz, 1H), 6.61 (t, *J* = 8.9 Hz, 4H), 3.66 (br, 1H), 3.35 (s, 4H), 1.28 (s, 9H); ^{13}C NMR (100 MHz, $CDCl_3$) δ 148.2, 145.8, 140.8, 129.4, 126.2, 117.8, 113.1, 112.9, 43.6, 43.5, 39.0, 31.6; HRMS (ESI) m/z calc. for $C_{18}H_{25}N_2$ $[M+H]^+$: 269.2012, found: 269.2016.

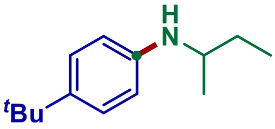

N-(Sec-butyl)-4-(tert-butyl)aniline (91): yellow oil; ^{1}H NMR (400 MHz, $CDCl_3$) δ 7.19 (d, *J* = 8.6 Hz, 2H), 6.54 (d, *J* = 8.5 Hz, 2H), 3.44 - 3.26 (m, 1H), 1.66 - 1.41 (m, 2H), 1.28 (s, 9H), 1.17 (d, *J* = 6.3 Hz, 3H), 0.95 (t, *J* = 7.4 Hz, 3H); ^{13}C NMR (100 MHz, $CDCl_3$) δ 145.3, 139.5, 126.0, 112.8, 50.0, 31.6, 33.8, 29.8, 20.4, 10.4; HRMS (ESI) m/z calc. for $C_{14}H_{24}N$ $[M+H]^+$: 206.1903, found: 206.1906.

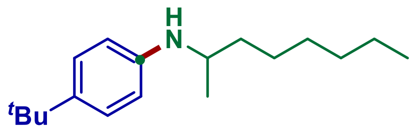

4-(Tert-butyl)-N-(octan-2-yl)aniline (92): yellow oil; ^{1}H NMR (400 MHz, CDCl$_3$) δ 7.11 (d, *J* = 8.7 Hz, 2H), 6.45 (d, *J* = 8.6 Hz, 2H), 3.39 - 3.28 (m, 1H), 1.58 - 1.42 (m, 1H), 1.38 - 1.20 (m, 9H), 1.20 (s, 9H), 1.07 (t, *J* = 9.6 Hz, 3H), 0.81 (t, *J* = 6.7 Hz, 3H); ^{13}C NMR (100 MHz, CDCl$_3$) δ 145.3, 139.5, 126.0, 112.7, 48.7, 37.4, 33.8, 31.9, 31.6, 29.4, 26.2, 22.6, 20.9, 14.1; HRMS (ESI) m/z calc. for $C_{18}H_{32}N$ [M+H]$^+$: 262.2529, found: 262.2534.

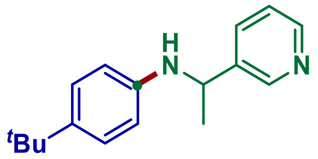

4-(Tert-butyl)-N-(1-(pyridin-3-yl)ethyl)aniline (93): yellow oil; ^{1}H NMR (400 MHz, CDCl$_3$) δ 8.65 (s, 1H), 8.49 (d, *J* = 3.6 Hz, 1H), 7.71 (d, *J* = 7.8 Hz, 1H), 7.26 - 7.21 (m, 1H), 7.13 (d, *J* = 8.7 Hz, 2H), 6.45 (d, *J* = 8.7 Hz, 2H), 4.50 (q, *J* = 6.7 Hz, 1H), 1.53 (d, *J* = 6.8 Hz, 3H), 1.24 (s, 9H); ^{13}C NMR (100 MHz, CDCl$_3$) δ 148.5, 148.4, 144.6, 141.0, 140.7, 133.7, 126.1, 123.8, 113.2, 51.8, 34.0, 31.6, 25.1; HRMS (ESI) m/z calc. for $C_{17}H_{23}N_2$ [M+H]$^+$: 255.1856, found: 255.1860.

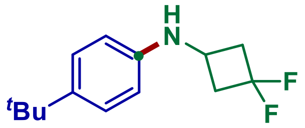

4-(Tert-butyl)-N-(3,3-difluorocyclobutyl)aniline (94): yellow oil; ^{1}H NMR (400 MHz, CDCl$_3$) δ 7.31 (d, *J* = 8.6 Hz, 2H), 6.49 (d, *J* = 8.6 Hz, 2H), 4.21 (t, *J* = 11.9 Hz, 4H), 1.31 (s, 9H); ^{13}C NMR (100 MHz, CDCl$_3$) δ 147.6, 141.8, 126.0, 116.1 (q, *J* = 273 Hz), 112.2, 63.5 (q, *J* = 25 Hz), 34.0, 31.5; ^{19}F NMR (376 MHz, CDCl$_3$) δ -99.21 (p, *J* = 11.8 Hz); HRMS (ESI) m/z calc. for $C_{14}H_{20}F_2N$ [M+H]$^+$: 240.1558, found: 240.1555.

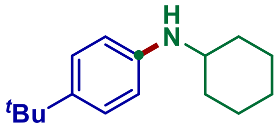

4-(Tert-butyl)-N-cyclohexylaniline (95): yellow oil; ^{1}H NMR (400 MHz, CDCl$_3$) δ 7.06 (d, *J* = 7.7 Hz, 2H), 6.43 (d, *J* = 7.8 Hz, 2H), 3.14 - 3.06 (m, 1H), 1.93 (d, *J* = 9.8 Hz, 2H), 1.66 - 1.61 (m, 2H), 1.55 - 1.50 (m, 1H), 1.32 - 1.23 (m, 2H), 1.16 (s, 9H), 1.09 - 097 (m, 2H); ^{13}C

NMR (100 MHz, $CDCl_3$) δ 145.2, 139.7, 126.1, 112.9, 52.0, 33.9, 33.7, 31.7, 26.1, 25.2; HRMS (ESI) m/z calc. for $C_{16}H_{26}N$ $[M+H]^+$: 232.2060, found: 232.2064.

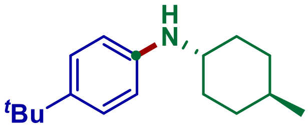

4-(Tert-butyl)-N-((1R,4R)-4-methylcyclohexyl)aniline (96): yellow oil; ^1H NMR (400 MHz, $CDCl_3$) δ 7.20 (d, J = 8.5 Hz, 2H), 6.60 (d, J = 8.5 Hz, 2H), 4.68 (t, J = 5.5 Hz, 1H), 3.78 - 3.67 (m, 2H), 3.61 - 3.52 (m, 2H), 3.24 (d, J = 5.5 Hz, 2H), 1.27 (s, 9H), 1.23 (t, J = 7.1 Hz, 6H); ^{13}C NMR (100 MHz, $CDCl_3$) δ 145.7, 140.6, 126.1, 113.0, 101.1, 62.3, 46.7, 34.0, 31.7, 15.5. HRMS (ESI) m/z calc. for $C_{17}H_{28}N$ $[M+H]^+$: 246.2216, found: 164.1435 .

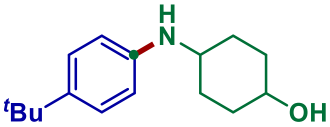

4-((4-(Tert-butyl)phenyl)amino)cyclohexan-1-ol (97): yellow oil; ^1H NMR (400 MHz, $CDCl_3$) δ 7.19 (d, J = 8.4 Hz, 2H), 6.55 (d, J = 8.4 Hz, 2H), 3.74 - 3.60 (m, 1H), 3.26 - 3.20 (m, 1H), 2.19 - 2.06 (m, 2H), 2.05 - 1.97 (m, 2H), 1.48 - 1.36 (m, 2H), 1.28 (s, 9H), 1.27 - 1.13 (m, 2H); ^{13}C NMR (100 MHz, $CDCl_3$) δ 144.8, 140.1, 126.1, 113.0, 70.2, 51.5, 34.1, 33.8, 31.6, 31.3. HRMS (ESI) m/z calc. for $C_{16}H_{26}NO$ $[M+H]^+$: 248.2009, found: 248.2011.

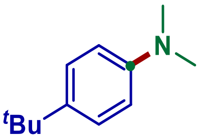

4-(Tert-butyl)-N,N-dimethylaniline (98): yellow oil; ^1H NMR (400 MHz, $CDCl_3$) δ 7.27 (d, J = 7.5 Hz, 2H), 6.72 (d, J = 7.7 Hz, 2H), 2.91 (s, 6H), 1.29 (s, 9H); ^{13}C NMR (100 MHz, $CDCl_3$) δ 148.6 , 139.4, 125.9, 112.7, 40.9, 33.8, 31.6; HRMS (ESI) m/z calc. for $C_{12}H_{20}N$ $[M+H]^+$: 178.1590, found: 178.1593.

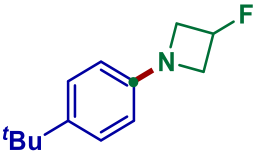

1-(4-(Tert-butyl)phenyl)-3-fluoroazetidine (99): yellow oil; ^1H NMR (400 MHz, $CDCl_3$) δ 7.18 (d, J = 8.6 Hz, 2H), 6.36 (d, J = 8.6 Hz, 2H), 5.44 - 5.12 (m, 1H), 4.16 - 3.93 (m, 2H), 3.91 - 3.75 (m, 2H), 1.21 (s, 9H); ^{13}C NMR (100 MHz, $CDCl_3$) δ 149.0, 141.0, 125.9, 111.7, 82.9 (d,

J = 204.3 Hz), 59.8 (d, J = 23.2 Hz), 34.0, 31.6; ^{19}F NMR (376 MHz, $CDCl_3$) δ -173.60 — -174.06 (m); HRMS (ESI) m/z calc. for $C_{13}H_{19}FN$ $[M+H]^+$: 208.1496, found: 208.1493.

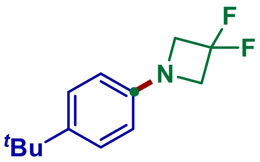

1-(4-(Tert-butyl)phenyl)-3,3-difluoroazetidine (100): yellow oil; ^{1}H NMR (400 MHz, $CDCl_3$) δ 7.29 (d, J = 8.6 Hz, 2H), 6.47 (d, J = 8.6 Hz, 2H), 4.18 (t, J = 11.9 Hz, 4H), 1.29 (s, 9H); ^{13}C NMR (100 MHz, $CDCl_3$) δ 147.6, 141.8, 126.0, 116.13 (t, J = 274.7 Hz), 112.2, 63.5 (t, J = 25.2 Hz), 34.0, 31.5; ^{19}F NMR (376 MHz, $CDCl_3$) δ -99.21 (p, J = 11.8 Hz); HRMS (ESI) m/z calc. for $C_{13}H_{18}F_2N$ $[M+H]^+$: 226.1402, found:226.1406.

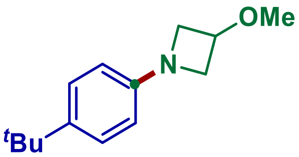

1-(4-(Tert-butyl)phenyl)-3-methoxyazetidine (101): yellow oil; ^{1}H NMR (400 MHz, $CDCl_3$) δ 7.24 (d, J = 7.5 Hz, 2H), 6.44 (d, J = 7.7 Hz, 2H), 4.39 - 4.27 (m, 1H), 4.08 (t, J = 6.8 Hz, 2H), 3.68 (dd, J = 7.1, 4.8 Hz, 2H), 3.32 (s, 3H), 1.28 (s, 9H); ^{13}C NMR (100 MHz, $CDCl_3$) δ 149.5, 140.4, 125.7, 111.5, 70.2, 59.1, 56.0, 33.9, 31.6; HRMS (ESI) m/z calc. for $C_{14}H_{22}NO$ $[M+H]^+$: 220.1696, found: 220.1693.

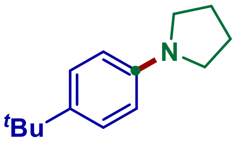

1-(4-(Tert-butyl)phenyl)pyrrolidinem (102): yellow oil; ^{1}H NMR (400 MHz, $CDCl_3$) δ 7.26 (d, J = 8.8 Hz, 1H), 6.55 (d, J = 8.7 Hz, 2H), 3.27 (m, 4H), 2.03 – 1.91 (m, 4H), 1.29 (s, 9H); ^{13}C NMR (100 MHz, $CDCl_3$) δ 145.8, 138.2, 125.95, 111.53, 47.90, 33.77, 31.61, 25.47; HRMS (ESI) m/z calc. for $C_{14}H_{22}N$ $[M+H]^+$: 204.1747, found: 204.1750.

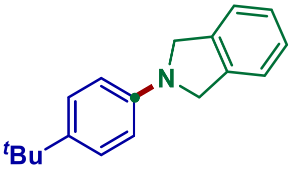

2-(4-(Tert-butyl)phenyl)isoindoline (103): yellow oil; ^{1}H NMR (400 MHz, $CDCl_3$) δ 7.37 – 7.31 (m, 4H), 7.36 – 7.27 (m, 2H), 6.65 (d, J = 8.7 Hz, 2H), 4.64 (s, 4H), 1.32 (s, 9H); ^{13}C NMR

(100 MHz, $CDCl_3$) δ 145.0, 138.2, 127.1, 126.2, 122.6, 119.8, 111.3, 109.8, 53.8, 33.8, 31.6; HRMS (ESI) m/z calc. for $C_{18}H_{22}N$ $[M+H]^+$: 252.1747, found: 252.1749.

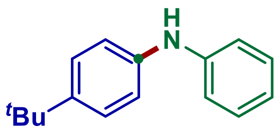

4-(Tert-butyl)-N-phenylaniline (104): yellow oil; ^1H NMR (400 MHz, $CDCl_3$) δ 7.32 - 7.20 (m, 4H), 7.09 - 6.98 (m, 4H), 6.88 (t, J = 6.7 Hz, 1H), 5.62 (br, 1H), 1.31 (s, 9H); ^{13}C NMR (100 MHz, $CDCl_3$) δ 144.2, 143.8, 140.4, 129.3, 126.1, 120.4, 118.2, 117.1, 34.2, 31.5; HRMS (ESI) m/z calc. for $C_{16}H_{20}N$ $[M+H]^+$: 226.1590, found: 226.1593.

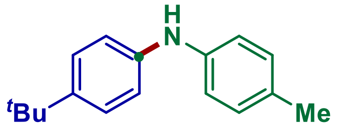

4-***(Tert-butyl)-N-(p-tolyl)aniline (105):*** yellow oil; ^1H NMR (400 MHz, $CDCl_3$) δ 7.31 (d, J = 3.0 Hz, 2H), 7.11 (d, J = 6.2 Hz, 2H), 7.06 - 6.98 (m, 4H), 2.34 (s, 3H), 1.36 (s, 9H); ^{13}C NMR (100 MHz, $CDCl_3$) δ 143.5, 141.2, 141.0, 130.3, 129.8, 126.1, 118.2, 117.2, 34.2, 31.5, 20.7; HRMS (ESI) m/z calc. for $C_{17}H_{22}N$ $[M+H]^+$: 240.1747, found: 240.1749.

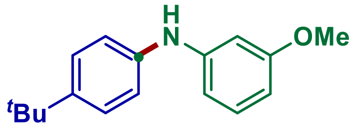

N-(4-(Tert-butyl)phenyl)-3-methoxyaniline (106): yellow oil; ^1H NMR (400 MHz, $CDCl_3$) δ 7.31 (dd, J = 8.6, 3.2 Hz, 2H), 7.19 - 7.11 (m, 1H), 7.06 (dd, J = 8.6, 3.2 Hz, 2H), 6.62 (dd, J = 6.2, 2.4 Hz, 2H), 6.49 - 6.41 (m, 1H), 3.78 (s, 3H), 1.32 (m, 9H); ^{13}C NMR (100 MHz, $CDCl_3$) δ 160.9, 145.4, 144.6, 140.2, 130.2, 126.3, 118.8, 109.8, 105.7, 102.8, 55.4, 34.3, 31.6; HRMS (ESI) m/z calc. for $C_{17}H_{22}NO$ $[M+H]^+$: 256.1696, found: 256.1699.

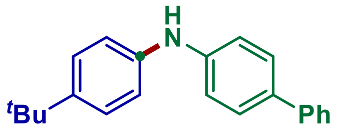

N-(4-(Tert-butyl)phenyl)-[1,1'-biphenyl]-4-amine (107): yellow oil; ^1H NMR (400 MHz, $CDCl_3$) δ 7.56 (d, J = 7.3 Hz, 2H), 7.48 (d, J = 8.5 Hz, 2H), 7.44 - 7.37 (m, 2H), 7.36 - 7.24 (m, 3H), 7.07 (t, J = 8.7 Hz, 4H), 5.70 (br, 1H), 1.32 (s, 9H); ^{13}C NMR (100 MHz, $CDCl_3$) δ 144.5,

143.2, 141.0, 140.1, 133.2, 128.8, 128.0, 126.5, 126.2, 118.5, 117.2, 34.2, 31.5; HRMS (ESI) m/z calc. for $C_{22}H_{24}N$ $[M+H]^+$: 302.1903, found: 302.1907.

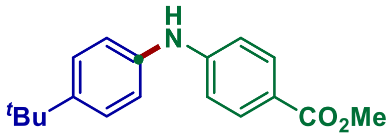

Methyl 4-((4-(tert-butyl)phenyl)amino)benzoate (108): yellow oil; ^{1}H NMR (400 MHz, $CDCl_3$) δ 7.90 (d, J = 8.7 Hz, 2H), 7.36 (d, J = 8.6 Hz, 2H), 7.12 (d, J = 8.6 Hz, 2H), 6.95 (d, J = 8.7 Hz, 2H), 5.94 (br, 1H), 3.87 (s, 3H), 1.33 (s, 9H); ^{13}C NMR (100 MHz, $CDCl_3$) δ 167.1, 148.6, 146.4, 138.1, 131.5, 126.3, 120.7, 114.8, 114.1, 51.7, 34.4, 31.4; HRMS (ESI) m/z calc. for $C_{18}H_{22}NO_2$ $[M+H]^+$: 284.1645, found: 284.1649.

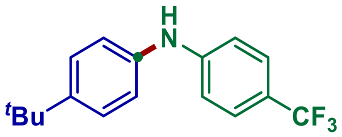

4-(Tert-butyl)-N-(4-(trifluoromethyl)phenyl)aniline (109): yellow oil; ^{1}H NMR (400 MHz, $CDCl_3$) δ 7.45 (d, J = 8.5 Hz, 2H), 7.36 (d, J = 8.5 Hz, 2H), 7.10 (d, J = 8.5 Hz, 2H), 7.00 (d, J = 8.5 Hz, 2H), 5.86 (br, 1H), 1.33 (s, 9H); ^{13}C NMR (100 MHz, $CDCl_3$) δ 147.5, 146.4, 138.5, 126.8 (q, J = 3.7 Hz), 124.8 (q, J = 269.5 Hz), 121.3 (q, J = 33.1 Hz), 120.5, 114.9, 34.5, 31.6; ^{19}F NMR (376 MHz, $CDCl_3$) δ -61.36 (s, CF_3); HRMS (ESI) m/z calc. for $C_{17}H_{19}F_3N$ $[M+H]^+$: 294.1464, found: 294.1460.

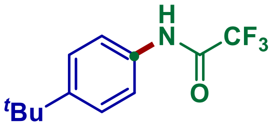

N-(4-(Tert-butyl)phenyl)-2,2,2-trifluoroacetamide (110): yellow oil; ^{1}H NMR (400 MHz, $CDCl_3$) δ 8.06 (br, 1H), 7.49 (d, J = 8.2 Hz, 2H), 7.40 (d, J = 8.3 Hz, 2H), 1.32 (s, 9H); ^{13}C NMR (100 MHz, $CDCl_3$) δ155.0, (q, J = 40 Hz), 132.6 , 126.3, 120.5 , 120.3, 116.0 (q, J = 286.9 Hz), 34.7, 31.4; ^{19}F NMR (376 MHz, $CDCl_3$) δ -77.55 (s, CF_3); HRMS (ESI) m/z calc. for $C_{12}H_{15}F_3NO$ $[M+H]^+$: 246.1100, found: 246.1103.

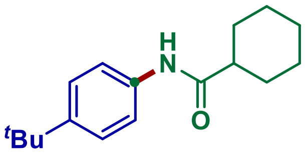

N-(4-(Tert-butyl)phenyl)cyclohexanecarboxamide (111): white solid; ^{1}H NMR (400 MHz, $CDCl_3$) δ 7.44 (d, J = 8.4 Hz, 2H), 7.33 (d, J = 8.5 Hz, 2H), 7.09 (br, 1H), 2.23 – 2.20 (m, 1H), 1.97 – 1.92 (m, 2H), 1.88 – 1.80 (m, 2H), 1.73 – 1.69 (m, 1H), 1.57 – 1.47 (m, 2H), 1.34 – 1.29 (m, 3H), 1.30 (s, 9H); ^{13}C NMR (100 MHz, $CDCl_3$) δ 174.2, 135.4, 125.8, 119.5, 114.7, 46.6, 34.3, 31.5, 31.4, 29.7, 25.7; HRMS (ESI) m/z calc. for $C_{17}H_{26}NO$ $[M+H]^+$: 260.2009, found: 260.2012.

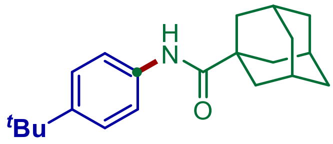

(3r,5r,7r)-N-(4-(Tert-butyl)phenyl)adamantane-1-carboxamide (112): white solid; ^{1}H NMR (400 MHz, $CDCl_3$) δ 7.45 (d, J = 8.6 Hz, 2H), 7.33 (d, J = 8.7 Hz, 2H), 7.25 (br, 1H), 2.13 – 2.19 (m, 3H), 1.98 – 1.92 (m, 6H), 1.78 – 1.71 (m, 6H), 1.30 (s, 9H); ^{13}C NMR (100 MHz, $CDCl_3$) δ 175.9, 147.1, 135.4, 125.8, 119.7, 41.4, 39.3, 36.5, 34.3, 31.4, 28.2; HRMS (ESI) m/z calc. for $C_{21}H_{30}NO$ $[M+H]^+$: 312.2322, found: 312.2324.

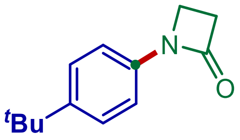

1-(4-(Tert-butyl)phenyl)azetidin-2-one (113): white solid; ^{1}H NMR (400 MHz, $CDCl_3$) δ 7.35 (d, J = 8.7 Hz, 2H), 7.29 (d, J = 8.7 Hz, 2H), 3.60 (t, J = 4.4 Hz, 2H), 3.09 (t, J = 4.4 Hz, 2H), 1.30 (s, 9H); ^{13}C NMR (100 MHz, $CDCl_3$) δ 164.3, 146.8, 136.1, 125.9, 115.9, 38.0, 36.1, 34.4, 31.4; HRMS (ESI) m/z calc. for $C_{13}H_{18}NO$ $[M+H]^+$: 204.1383, found: 204.1285.

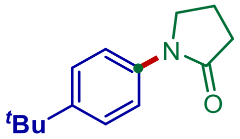

1-(4-(Tert-butyl)phenyl)pyrrolidin-2-one (114): white solid; ^{11}H NMR (400 MHz, $CDCl_3$) δ 7.51 (d, J = 8.8 Hz, 2H), 7.38 (d, J = 8.8 Hz, 2H), 3.85 (t, J = 7.0 Hz, 2H), 2.60 (t, J = 8.1 Hz, 2H), 2.19 – 2.10 (m, 2H), 1.31 (s, 9H); ^{13}C NMR (100 MHz, $CDCl_3$) δ 174.1, 147.5, 136.8, 125.7, 119.9, 48.9, 34.4, 32.7, 31.3, 18.11; HRMS (ESI) m/z calc. for $C_{14}H_{20}NO$ $[M+H]^+$: 218.1539, found: 218.1541.

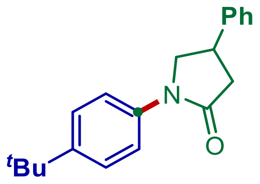

1-(4-(Tert-butyl)phenyl)-4-phenylpyrrolidin-2-one (115): white solid; ^{1}H NMR (400 MHz, $CDCl_3$) δ 7.54 (d, *J* = 8.7 Hz, 2H), 7.40 (d, *J* = 8.8 Hz, 2H), 7.35 (d, *J* = 6.9 Hz, 1H), 7.29 (d, *J* = 7.5 Hz, 2H), 7.25 – 7.22 (m, 1H), 7.22 – 7.16 (m, 1H), 4.19 (dd, *J* = 9.5, 8.2 Hz, 1H), 3.89 (dd, *J* = 9.6, 7.3 Hz, 1H), 3.75 – 3.64 (m, 1H), 3.05 – 2.96 (m, 1H), 2.80 – 2.91 (m, 1H), 1.31 (s, 9H); ^{13}C NMR (100 MHz, $CDCl_3$) δ 173.0, 147.8, 141.8, 136.5, 129.0, 126.8, 125.8, 119.9, 114.8, 55.8, 40.3, 37.2, 31.6, 31.4; HRMS (ESI) m/z calc. for $C_{20}H_{24}NO$ $[M+H]^+$: 294.1852, found: 294.1854.

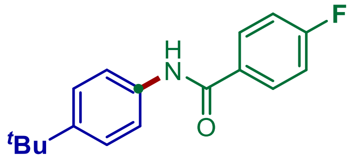

N-(4-(Tert-butyl)phenyl)-2,6-difluorobenzamide (116): white solid; ^{1}H NMR (400 MHz, $CDCl_3$) δ 7.88 (dd, *J* = 8.5, 5.3 Hz, 2H), 7.76 (br, 1H), 7.54 (d, *J* = 8.5 Hz, 2H), 7.39 (d, *J* = 8.6 Hz, 2H), 7.15 (t, *J* = 8.5 Hz, 2H), 1.32 (s, 9H); ^{13}C NMR (100 MHz, $CDCl_3$) δ 164.7 (d, *J* = 250.8 Hz), 147.8, 135.1, 129.4 (d, *J* = 9.0 Hz), 126.0, 120.1, 115.8 (d, *J* = 21.9 Hz), 114.8, 34.5, 31.4; ^{19}F NMR (376 MHz, $CDCl_3$) δ -105.63 (s, F); HRMS (ESI) m/z calc. for $C_{17}H_{19}FNO$ $[M+H]^+$: 272.1445, found: 272.1448.

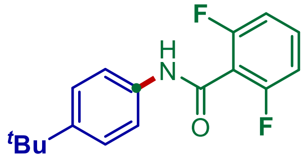

N-(4-(Tert-butyl)phenyl)-4-fluorobenzamide (117): white solid; ^{1}H NMR (400 MHz, $CDCl_3$) δ 7.62 (br, 1H), 7.55 (d, *J* = 8.6 Hz, 2H), 7.39 (d, *J* = 8.5 Hz, 3H), 6.98 (t, *J* = 8.1 Hz, 2H), 1.32 (s, 9H); ^{13}C NMR (100 MHz, $CDCl_3$) δ 160.1 (dd, *J* = 252.8, 6.7 Hz), 158.2, 148.1, 134.7, 132.0 (dd, *J* = 10.2, 110.3 Hz), 125.9, 120.0, 112.53 – 111.68 (m), 34.5, 31.4; ^{19}F NMR (376 MHz, $CDCl_3$) δ -107.24 (s, F); HRMS (ESI) m/z calc. for $C_{17}H_{18}F_2NO$ $[M+H]^+$: 290.1351, found: 290.1354.

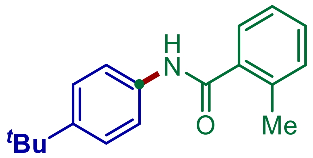

N-(4-(Tert-butyl)phenyl)-2-methylbenzamide (118): white solid; ^{1}H NMR (400 MHz, $CDCl_3$) δ 7.54 (d, J = 8.2 Hz, 2H), 7.47 (d, J = 7.6 Hz, 2H), 7.37 (d, J = 7.9 Hz, 3H), 6.76 (d, J = 8.5 Hz, 2H), 5.00 (br, 1H), 2.50 (s, 3H), 1.32 (s, 9H); ^{13}C NMR (100 MHz, $CDCl_3$) δ 168.1, 153.3, 143.4, 136.4, 135.3, 131.3, 130.3, 126.4, 126.0, 119.8, 114.8, 34.4, 31.6, 19.8; HRMS (ESI) m/z calc. for $C_{18}H_{22}NO$ $[M+H]^+$: 268.1696, found: 268.1698.

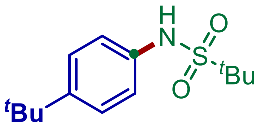

N-(4-(Tert-butyl)phenyl)-2-methylpropane-2-sulfonamide (119): yellow solid; ^{1}H NMR (400 MHz, $CDCl_3$) δ 7.31 – 7.27 (m, 2H), 7.24 – 7.20 (m, 2H), 6.93 (br, 1H), 1.40 (s, 9H), 1.29 (s, 9H); ^{13}C NMR (100 MHz, $CDCl_3$) δ 147.6, 135.9, 126.2, 120.4, 61.9, 34.4, 31.4, 24.9; HRMS (ESI) m/z calc. for $C_{14}H_{24}NO_2S$ $[M+H]^+$: 270.1522, found: 270.1524.

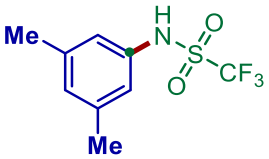

N-(3,5-Dimethylphenyl)-1,1,1-trifluoromethanesulfonamide (120): yellow solid; ^{1}H NMR (400 MHz, $CDCl_3$) δ 6.94 (s, 1H), 6.88 (s, 2H), 6.79 (br, 1H), 2.32 (s, 6H); ^{13}C NMR (100 MHz, $CDCl_3$) δ 139.6, 133.4, 129.3, 121.3, 119.8 (q, J = 322.9 Hz). 21.2; ^{19}F NMR (376 MHz, $CDCl_3$) δ -73.78 (s, CF_3); HRMS (ESI) m/z calc. for $C_9H_{11}F_3NO_2S$ $[M+H]^+$: 254.0457, found: 254.0458.

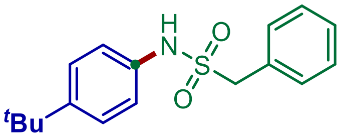

N-(4-(Tert-butyl)phenyl)-1-phenylmethanesulfonamide (121): yellow solid; ^{1}H NMR (400 MHz, $CDCl_3$) δ 7.36 – 7.32 (m, 5H), 7.30 – 7.26 (m, 2H), 7.11 – 7.07 (m, 2H), 6.42 (br, 1H), 4.30 (s, 2H), 1.32 (s, 9H); ^{13}C NMR (100 MHz, $CDCl_3$) δ 148.3, 134.1, 131.0, 129.1, 129.0, 128.9, 126.5, 120.4, 57.5, 34.5, 31.4; HRMS (ESI) m/z calc. for $C_{17}H_{22}NO_2S$ $[M+H]^+$: 304.1366, found: 304.1367.

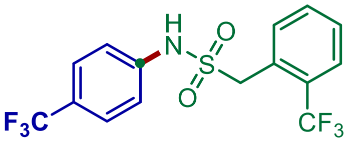

1-(2-(Trifluoromethyl)phenyl)-N-(4-(trifluoromethyl)phenyl)methanesulfonamide (122): yellow solid; ^{1}H NMR (400 MHz, DMSO) δ 10.67 (br, 1H), 7.75 (t, *J* = 6.8 Hz, 1H), 7.72 – 7.64 (m, 3H), 7.64 – 7.54 (m, 2H), 7.36 (d, *J* = 8.5 Hz, 2H), 4.73 (s, 2H); ^{13}C NMR (100 MHz, DMSO) δ 142.5, 134.3, 132.9, 129.7, 129.0 (q, *J* = 29.8 Hz), 127.5 (q, *J* = 1.5 Hz), 127.15 – 126.80 (m, CF_3, CF_3), 124.78 (q, *J* = 271.3 Hz), 124.43 (q, *J* = 274.3 Hz), 123.88 (q, *J* = 32.3 Hz), 118.6, 54.9; HRMS (ESI) m/z calc. for $C_{15}H_{12}F_6NO_2S$ $[M+H]^+$: 384.0487, found: 384.0490.

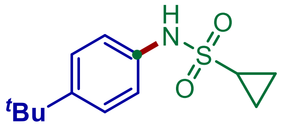

N-(4-(Tert-butyl)phenyl)cyclopropanesulfonamide (123): yellow solid; ^{1}H NMR (400 MHz, $CDCl_3$) δ 7.34 (d, *J* = 8.6 Hz, 2H), 7.21 (d, *J* = 8.6 Hz, 2H), 6.98 (br, 1H), 2.57 – 2.44 (m, 1H), 1.30 (s, 9H), 1.21 – 1.10 (m, 2H), 0.98 – 0.89 (m, 2H); ^{13}C NMR (100 MHz, $CDCl_3$) δ 148.6, 134.1, 126.3, 122.0, 34.4, 31.4, 29.7, 5.6; HRMS (ESI) m/z calc. for $C_{13}H_{20}NO_2S$ $[M+H]^+$: 254.1209, found: 254.1211.

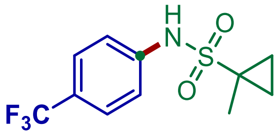

1-Methyl-N-(4-(trifluoromethyl)phenyl)cyclopropane-1-sulfonamide (124): yellow solid; ^{1}H NMR (400 MHz, $CDCl_3$) δ 7.59 (br, 1H), 7.57 (d, *J* = 8.3 Hz, 2H) , 7.37 (d, *J* = 8.4 Hz, 2H), 1.52 (s, 3H), 1.45 – 1.39 (m, 2H), 0.83 – 0.75 (m, 2H); ^{13}C NMR (100 MHz, $CDCl_3$) δ 140.8, 126.7 (q, *J* = 3.7 Hz), 126.6 (q, *J* = 32.8 Hz), 124.0 (q, *J* = 271.6 Hz), 119.9, 36.5, 18.4, 12.9; ^{19}F NMR (376 MHz, $CDCl_3$) δ -62.18 (s, CF_3); HRMS (ESI) m/z calc. for $C_{11}H_{13}F_3NO_2S$ $[M+H]^+$: 280.0614, found: 280.0616.

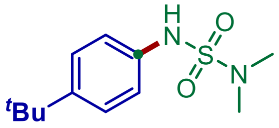

N-(4-(Tert-butyl)phenyl)-N,N-Dimethylsulfamide (125): yellow solid; ^{1}H NMR (400 MHz, $CDCl_3$) δ 7.32 (d, *J* = 8.6 Hz, 2H), 7.12 (d, *J* = 8.7 Hz, 2H), 6.74 (br, 1H), 2.84 (s, 6H), 1.30 (s, 9H); ^{13}C NMR (100 MHz, $CDCl_3$) δ 147.7, 134.7, 126.2, 120.3, 38.3, 34.5, 31.5; HRMS (ESI) m/z calc. for $C_{12}H_{21}N_2O_2S$ $[M+H]^+$: 257.1318, found: 257.1321.

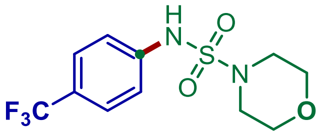

N-(4-(Trifluoromethyl)phenyl)morpholine-4-sulfonamide (126): yellow solid; ^{1}H NMR (400 MHz, $CDCl_3$) δ 7.58 (d, *J* = 8.5 Hz, 2H), 7.34 (br, 1H), 7.33 – 7.19 (m, 2H), 3.73 – 3.60 (m, 4H), 3.35 – 3.23 (m, 4H); ^{13}C NMR (100 MHz, $CDCl_3$) δ 140.4, 126.7 (q, *J* = 3.8 Hz), 126.3 (q, *J* = 33.0 Hz), 123.9 (q, *J* = 271.5 Hz), 118.8, 66.0, 46.3; ^{19}F NMR (376 MHz, $CDCl_3$) δ -59.16 (s, CF_3); HRMS (ESI) m/z calc. for $C_{11}H_{14}F_3N_2O_3S$ $[M+H]^+$: 311.0672, found: 311.0674.

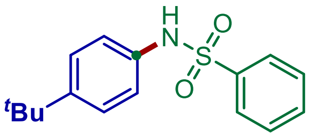

N-(4-(Tert-butyl)phenyl)benzenesulfonamide (127): yellow solid; ^{1}H NMR (400 MHz, $CDCl_3$) δ 7.80 – 7.77 (m, 2H), 7.56 – 7.50 (m, 1H), 7.46 – 7.41 (m, 2H), 7.26 – 7.21 (m, 3H), 7.01 – 6.96 (m, 2H), 6.81 (br, 1H), 1.25 (s, 9H); ^{13}C NMR (100 MHz, $CDCl_3$) δ 148.8, 139.5, 133.7, 133.0, 129.1, 127.3, 126.3, 121.9, 34.5, 31.4; HRMS (ESI) m/z calc. for $C_{16}H_{20}NO_2S$ $[M+H]^+$: 290.1209, found: 290.1208.

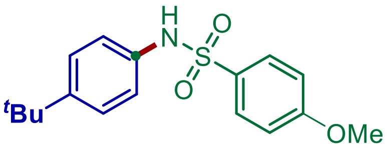

N-(4-(Tert-butyl)phenyl)-4-methoxybenzenesulfonamide (128): yellow solid; ^{1}H NMR (400 MHz, $CDCl_3$) δ 7.73 (d, *J* = 8.7 Hz, 2H), 7.23 (d, *J* = 8.4 Hz, 2H), 6.99 (d, *J* = 8.4 Hz, 2H), 6.94 (br, 1H), 6.89 (d, *J* = 8.7 Hz, 2H), 3.82 (s, 3H), 1.25 (s, 9H); ^{13}C NMR (100 MHz, $CDCl_3$) δ 163.1, 148.5, 134.0, 131.0, 129.6, 126.3, 121.6, 114.3, 55.7, 34.5, 31.4; HRMS (ESI) m/z calc. for $C_{17}H_{22}NO_3S$ $[M+H]^+$: 320.1315, found: 320.1317.

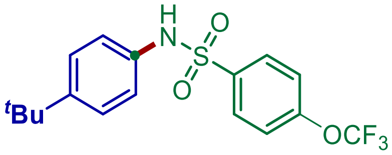

N-(4-(Tert-butyl)phenyl)-4-(trifluoromethoxy)benzenesulfonamide (129): yellow solid; ^{1}H NMR (400 MHz, $CDCl_3$) δ 7.82 (d, *J* = 7.9 Hz, 2H), 7.27 (d, *J* = 7.4 Hz, 4H), 6.99 (d, *J* = 8.0 Hz, 2H), 6.75 (br, 1H), 1.26 (s, 9H); ^{13}C NMR (100 MHz, $CDCl_3$) δ 152.5, 149.3, 137.8, 133.2, 129.6, 126.56, 122.2, 120.9, 120.19 (q, *J* = 259.7 Hz), 34.6, 31.4; ^{19}F NMR (376 MHz, $CDCl_3$) δ -57.61 (s, CF_3); HRMS (ESI) m/z calc. for $C_{17}H_{19}F_3NO_3S$ $[M+H]^+$: 374.1032, found: 374.1035.

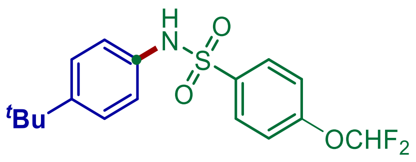

N-(4-(Tert-butyl)phenyl)-4-(difluoromethoxy)benzenesulfonamide (130): yellow solid; ^{1}H NMR (400 MHz, $CDCl_3$) δ 7.80 (d, *J* = 7.5 Hz, 2H), 7.25 (d, *J* = 7.2 Hz, 2H), 7.20 (br, 1H), 7.13 (d, *J* = 7.8 Hz, 2H), 7.00 (d, *J* = 7.2 Hz, 2H), 6.52 (t, *J* = 57.1 Hz, 1H), 1.25 (s, 9H); ^{13}C NMR (100 MHz, $CDCl_3$) δ 154.3, 148.8, 135.9, 133.4, 129.5, k126.3, 121.8, 119.2, 115.7 (t, *J* = 264.2 Hz), 34.4, 31.3; ^{19}F NMR (376 MHz, $CDCl_3$) δ -80.90 (d, *J* = 72.5 Hz); HRMS (ESI) m/z calc. for $C_{17}H_{20}F_2NO_3S$ $[M+H]^+$: 356.1126, found: 356.1130.

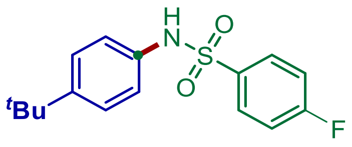

N-(4-(Tert-butyl)phenyl)-4-fluorobenzenesulfonamide (131): yellow solid; ^{1}H NMR (400 MHz, $CDCl_3$) δ 7.83 – 7.77 (m, 2H), 7.27 – 7.23 (m, 2H), 7.12 – 7.06 (m, 2H), 7.04 (br, 1H), 7.02 – 6.97 (m, 2H), 1.26 (s, 9H); ^{13}C NMR (100 MHz, $CDCl_3$) δ 165.2 (d, *J* = 255.1 Hz), 148.9, 135.3 (d, *J* = 3.2 Hz), 133.4, 130.0 (d, *J* = 9.4 Hz), 126.3, 121.9, 116.3 (d, *J* = 22.6 Hz), 34.4, 31.3; ^{19}F NMR (376 MHz, $CDCl_3$) δ -106.69 (s, F); HRMS (ESI) m/z calc. for $C_{16}H_{19}FNO_2S$ $[M+H]^+$: 308.1115, found: 308.1117.

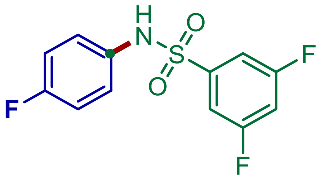

3,5-Difluoro-N-(4-fluorophenyl)benzenesulfonamide (132): yellow solid; ^{1}H NMR (400 MHz, $CDCl_3$) δ 7.31 – 7.21 (m, 2H), 7.11 – 7.04 (m, 2H), 7.03 – 6.95 (m, 3H), 6.78 (br, 1H); ^{13}C NMR (100 MHz, $CDCl_3$) δ 162.7 (dd, *J* = 255.3, 11.6 Hz), 161.2 (d, *J* = 247.1 Hz), 142.0 (t, *J* = 8.4 Hz), 131.2 (d, *J* = 3.1 Hz), 125.4 (d, *J* = 8.5 Hz), 116.5 (d, *J* = 22.9 Hz), 111.43 – 110.25 (m), 108.8 (t, *J* = 25.0 Hz); ^{19}F NMR (376 MHz, $CDCl_3$) δ -110.19 (s, F), -117.31 (s, F); HRMS (ESI) m/z calc. for $C_{12}H_9F_3NO_2S$ $[M+H]^+$: 288.0301, found: 288.0303.

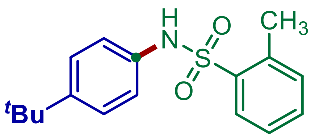

N-(4-(Tert-butyl)phenyl)-2-methylbenzenesulfonamide (133): yellow solid; ^{1}H NMR (400 MHz, $CDCl_3$) ^{1}H NMR (400 MHz, $CDCl_3$) δ 8.02 – 7.94 (m, 1H), 7.43 (t, *J* = 6.9 Hz, 1H), 7.28 (d, *J* = 7.4 Hz, 2H), 7.22 (d, *J* = 8.6 Hz, 2H), 6.98 – 6.84 (m, 2H), 6.69 (br, 1H), 2.65 (s, 3H),

1.24 (s, 9H); ^{13}C NMR (100 MHz, $CDCl_3$) δ 148.4, 137.0, 137.4, 133.8, 133.1, 132.7, 130.0, 126.4, 126.4, 121.0, 34.5, 31.4, 20.5; HRMS (ESI) m/z calc. for $C_{17}H_{22}NO_2S$ $[M+H]^+$: 304.1366, found: 304.1370.

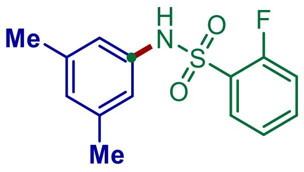

N-(3,5-Dimethylphenyl)-2-fluorobenzenesulfonamide (134): yellow solid; ^{1}H NMR (400 MHz, $CDCl_3$) δ 7.84 (td, J = 7.5, 1.5 Hz, 1H), 7.60 – 7.46 (m, 1H), 7.24 – 7.12 (m, 2H), 6.85 (br, 1H), 6.72 (s, 3H), 2.20 (s, 6H), ^{13}C NMR (100 MHz, $CDCl_3$) δ 158.8 (d, J = 254.3 Hz), 139.1, 135.6, 135.3 (d, J = 8.7 Hz), 131.0, 127.4, 124.5 (d, J = 3.8 Hz), 118.9, 116.8 (d, J = 21.1 Hz), 21.2; ^{9}F NMR (376 MHz, $CDCl_3$) δ -112.63 (s, F); HRMS (ESI) m/z calc. for $C_{14}H_{15}FNO_2S$ $[M+H]^+$: 280.0802, found: 280.0804.

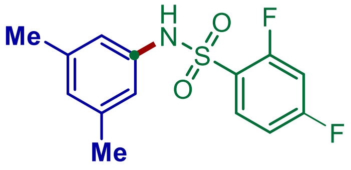

N-(3,5-Dimethylphenyl)-2,4-difluorobenzenesulfonamide: (135): yellow solid; ^{1}H NMR (400 MHz, $CDCl_3$) δ 7.96 – 7.80 (m, 1H), 6.93 (d, J = 3.2 Hz, 2H), 6.81 (s, 1H), 6.77 – 6.67 (m, 3H), 2.21 (s, 6H); ^{13}C NMR (100 MHz, $CDCl_3$) δ 166.0 (dd, J = 257.9, 11.7 Hz), 159.5 (dd, J = 257.5, 12.9 Hz), 139.3, 135.5, 132.5 (d, J = 11.6 Hz), 127.5, 123.3 (dd, J = 13.7, 3.8 Hz), 118.8, 112.0 (dd, J = 21.9, 3.6 Hz), 105.5 (dd, J = 26.0, 25.1 Hz) , 21.2; ^{19}F NMR (376 MHz, $CDCl_3$) δ -99.79 (s, F), -105.20 (s, F); HRMS (ESI) m/z calc. for $C_{14}H_{14}F_2NO_2S$ $[M+H]^+$: 298.0708, found: 298.0710.

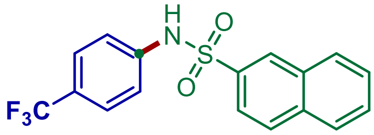

N-(4-(Trifluoromethyl)phenyl)naphthalene-2-sulfonamide (136): yellow solid; ^{1}H NMR (400 MHz, $CDCl_3$) δ 8.46 (s, 1H), 7.97 – 7.90 (m, 2H), 7.88 (d, J = 8.0 Hz, 1H), 7.81 (d, J = 8.7 Hz, 1H), 7.63 (dt, J = 15.1, 6.9 Hz, 2H), 7.46 (d, J = 8.4 Hz, 3H, NH, CH_2), 7.23 (d, J = 8.4 Hz, 2H); ^{13}C NMR (100 MHz, $CDCl_3$) δ 139.8, 135.6, 135.1, 132.0, 129.8, 129.4, 129.3, 129.1, 128.0, 127.8, 126.69 (q, J = 3.8 Hz), 125.2, 121.9, 121.1 (q, J = 270.0 Hz), 119.9; ^{19}F NMR (376 MHz, $CDCl_3$) δ -62.17 (s, CF_3); HRMS (ESI) m/z calc. for $C_{17}H_{13}F_3NO_2S$ $[M+H]^+$: 352.0614, found: 352.0616.

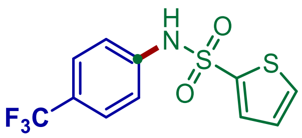

N-(4-(Trifluoromethyl)phenyl)thiophene-2-sulfonamide (137): yellow solid; ^{1}H NMR (400 MHz, $CDCl_3$) δ 7.63 – 7.56 (m, 2H), 7.54 (d, J = 8.5 Hz, 2H), 7.45 (br, 1H), 7.27 (d, J = 7.9

Hz, 2H), 7.07 – 7.01 (m, 1H); ^{13}C NMR (100 MHz, $CDCl_3$) δ 139.5, 139.0, 133.4, 133.1, 127.6, 127.1, 126.7 (q, *J* = 3.7 Hz), 123.9 (q, *J* = 271.7 Hz), 120.2; ^{19}F NMR (376 MHz, $CDCl_3$) δ -62.16 (s, CF_3); HRMS (ESI) m/z calc. for $C_{11}H_9F_3NO_2S_2$ $[M+H]^+$: 308.0021, found: 308.0022.

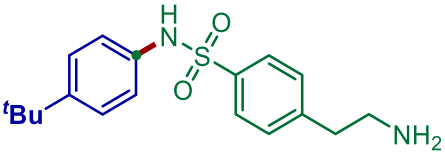

4-(2-Aminoethyl)-N-(4-(tert-butyl)phenyl)benzenesulfonamide (138): yellow solid; ^{1}H NMR (400 MHz, $CDCl_3$) δ 7.87 (d, *J* = 7.9 Hz, 2H), 7.38 (d, *J* = 8.0 Hz, 2H), 7.22 (d, *J* = 8.2 Hz, 2H), 6.58 (d, *J* = 8.1 Hz, 2H), 4.80 (br, 2H), 3.43 (t, *J* = 6.8 Hz, 2H), 2.99 (t, *J* = 6.8 Hz, 2H), 1.28 (s, 9H); ^{13}C NMR (100 MHz, $CDCl_3$) δ 148.9, 145.1, 140.0, 136.2, 129.6, 126.2, 118.8, 112.8, 44.9, 35.6, 33.9, 31.5; HRMS (ESI) m/z calc. for $C_{18}H_{25}N_2O_2S$ $[M+H]^+$: 333.1631, found: 333.1634.

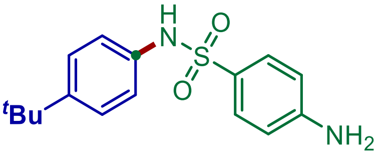

4-Amino-N-(4-(tert-butyl)phenyl)benzenesulfonamide (139): yellow solid; ^{1}H NMR (400 MHz, DMSO) δ 9.76 (br, 1H), 7.38 (d, *J* = 8.7 Hz, 2H), 7.21 (d, *J* = 8.6 Hz, 2H), 6.97 (d, *J* = 8.6 Hz, 2H), 6.52 (d, *J* = 8.7 Hz, 2H), 1.19 (s, 9H); ^{13}C NMR (100 MHz, DMSO) δ 152.7, 145.6, 135.8, 128.7, 125.6, 124.8, 119.3, 112.6, 33.9, 31.2; HRMS (ESI) m/z calc. for $C_{16}H_{21}N_2O_2S$ $[M+H]^+$: 305.1318, found: 305.1320.

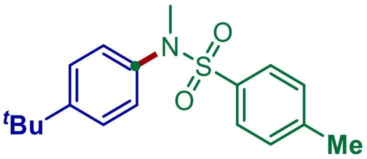

N-(4-(Tert-butyl)phenyl)-N,4-dimethylbenzenesulfonamide (140): yellow solid; ^{1}H NMR (400 MHz, $CDCl_3$) δ 7.68 – 7.64 (m, 2H), 7.28 – 7.24 (m, 2H), 7.23 (s, 2H), 6.97 (d, *J* = 8.7 Hz, 2H), 6.58 (br, 1H), 2.69 (s, 3H), 2.38 (s, 3H), 1.26 (s, 9H); ^{13}C NMR (100 MHz, $CDCl_3$) δ 148.5, 136.5, 133.7, 129.6 , 127.8, 127.3, 126.2, 121.7, 31.5, 31.3; HRMS (ESI) m/z calc. for $C_{18}H_{24}NO_2S$ $[M+H]^+$: 318.1522, found: 318.1524.

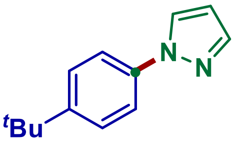

1-(4-(Tert-butyl)phenyl)-1H-pyrazole (141): yellow oil; ^{1}H NMR (400 MHz, $CDCl_3$) δ 7.89 (d, *J* = 2.4 Hz, 1H), 7.71 (d, *J* = 1.1 Hz, 1H), 7.61 (d, *J* = 8.7 Hz, 2H), 7.46 (d, *J* = 8.7 Hz, 2H),

6.44 (t, J = 2.0 Hz, 1H); 1.34 (s, 9H); ^{13}C NMR (100 MHz, CDCl$_3$) δ 149.6, 140.8, 137.9, 126.7, 126.3, 118.9, 107.3, 34.5, 31.4; HRMS (ESI) m/z calc. for $C_{13}H_{17}N_2$ [M+H]$^+$: 201.1386, found: 201.1389.

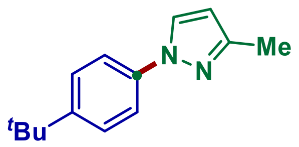

1-(4-(Tert-butyl)phenyl)-3-methyl-1H-pyrazole (142): yellow oil; ^{1}H NMR (400 MHz, CDCl$_3$) δ 7.77 (d, J = 1.9 Hz, 1H), 7.55 (d, J = 8.6 Hz, 2H), 7.43 (d, J = 8.6 Hz, 2H), 6.21 (d, J = 1.9 Hz, 1H), 2.37 (s, 3H), 1.33 (s, 9H); ^{13}C NMR (100 MHz, CDCl$_3$) δ 150.2, 149.0, 137.9, 127.3, 126.2, 118.6, 107.2, 34.5, 31.4, 13.7; HRMS (APSI) m/z calc. for $C_{14}H_{19}N_2$ [M+H]$^+$: 215.1543, found: 215.1546.

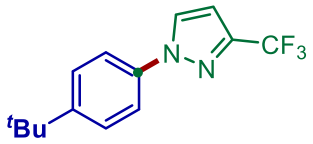

1-(4-(Tert-butyl)phenyl)-3-(trifluoromethyl)-1H-pyrazole (143): yellow oil; ^{1}H NMR (400 MHz, CDCl$_3$) δ 7.91 (s, 1H), 7.61 (d, J = 8.7 Hz, 2H), 7.49 (d, J = 8.7 Hz, 2H), 6.70 (d, J = 2.2 Hz, 1H), 1.35 (s, 9H); ^{13}C NMR (100 MHz, CDCl$_3$) δ 151.1, 143.6, 137.1, 128.2, 126.4, 126.1, 121.2 (q, J = 268.7 Hz), 119.7, 105.7, 34.7, 31.3; ^{19}F NMR (376 MHz, CDCl$_3$) δ -60.67 (s, CF$_3$); HRMS (ESI) m/z calc. for $C_{14}H_{16}F_3N_2$ [M+H]$^+$: 269.1260, found: 269.1262.

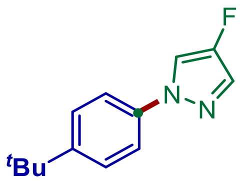

1-(4-(Tert-butyl)phenyl)-4-fluoro-1H-pyrazole (144): yellow oil; ^{1}H NMR (400 MHz, CDCl$_3$) δ 7.77 (d, J = 4.8 Hz, 1H), 7.57 – 7.51 (m, 3H), 7.46 (d, J = 8.7 Hz, 2H), 1.34 (s, 9H); ^{13}C NMR (100 MHz, CDCl$_3$) δ 154.8 (d, J = 241.9 Hz), 149.9, 137.8, 128.1 (d, J = 13.6 Hz), 126.4, 118.5, 112.9 (d, J = 28.3 Hz), 34.6, 31.3; ^{19}F NMR (376 MHz, CDCl$_3$) δ -178.66 (s, F).HRMS (ESI) m/z calc. for $C_{13}H_{16}FN_2$ [M+H]$^+$: 219.1292, found: 219.1296.

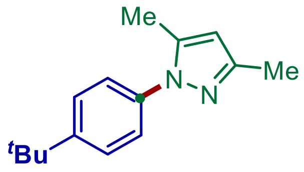

1-(4-(Tert-butyl)phenyl)-3,5-dimethyl-1H-pyrazole (145): yellow oil; ^{1}H NMR (400 MHz, $CDCl_3$) δ 7.44 (d, *J* = 8.5 Hz, 2H), 7.33 (d, *J* = 8.5 Hz, 2H), 5.97 (s, 1H), 2.29 (s, 6H), 1.34 (s, 9H); ^{13}C NMR (100 MHz, $CDCl_3$) δ 150.3, 148.7, 139.3, 137.4, 125.9, 124.4, 106.6, 31.3, 12.3; HRMS (ESI) m/z calc. for $C_{15}H_{21}N_2$ $[M+H]^+$: 229.1699, found: 229.1695.

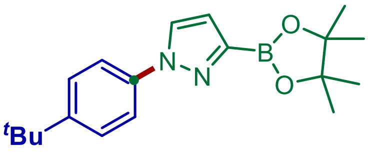

1-(4-(Tert-butyl)phenyl)-3-(4,4,5,5-tetramethyl-1,3,2-dioxaborolan-2-yl)-1H-pyrazole (146): yellow oil; ^{1}H NMR (400 MHz, $CDCl_3$) δ 8.21 (s, 1H), 7.96 (s, 1H), 7.62 (d, *J* = 8.7 Hz, 2H), 7.46 (d, *J* = 8.7 Hz, 2H), 1.35 (s, 9H), 1.24 (s, 12H); ^{13}C NMR (100 MHz, $CDCl_3$) δ 146.6, 133.5, 126.3, 119.1, 83.5, 35.2, 31.3, 24.8; HRMS (ESI) m/z calc. for $C_{19}H_{28}BN_2O_2$ $[M+H]^+$: 327.2238, found: 327.2243.

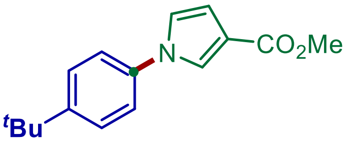

Methyl 1-(4-(tert-butyl)phenyl)-1H-pyrrole-3-carboxylate (147): yellow oil; ^{1}H NMR (400 MHz, $CDCl_3$) δ 7.67 (s, 1H), 7.47 (d, *J* = 8.6 Hz, 2H), 7.33 (d, *J* = 8.6 Hz, 2H), 7.00 (t, *J* = 2.6 Hz, 1H), 6.76 – 6.71 (m, 1H), 3.84 (s, 3H), 1.35 (s, 9H); ^{13}C NMR (100 MHz, $CDCl_3$) δ 165.2, 150.1, 142.0, 137.4, 135.3, 126.6, 124.4, 120.6, 117.6, 111.3, 51.2, 34.6, 31.3; HRMS (ESI) m/z calc. for $C_{16}H_{20}NO_2$ $[M+H]^+$: 258.1489, found: 258.1491.

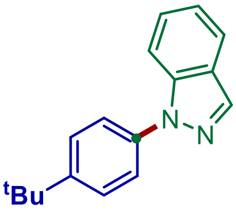

1-(4-(Tert-butyl)phenyl)-1H-indazole (148): yellow oil; ^{1}H NMR (400 MHz, $CDCl_3$) δ 8.18 (s, 1H), 7.76 (d, 8.3 Hz, 2H), 7.64 (d, *J* = 8.4 Hz, 2H), 7.54 (d, *J* = 8.4 Hz, 2H), 7.40 (t, *J* = 7.7 Hz, 1H), 7.21 (d, *J* = 6.0 Hz, 1H), 1.38 (s, 9H); ^{13}C NMR (100 MHz, $CDCl_3$) δ 149.8, 138.8, 137.7, 135.1, 127.0, 126.5, 125.2, 122.4, 121.4, 121.3, 110.5, 34.7, 31.4; HRMS (ESI) m/z calc. for $C_{17}H_{19}N_2$ $[M+H]^+$: 251.1543, found: 251.1545.

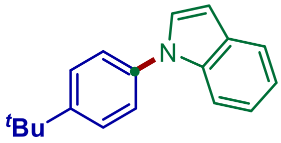

1-(4-(Tert-butyl)phenyl)-1H-indole (149): yellow oil; ^{1}H NMR (400 MHz, $CDCl_3$) δ 7.68 (d, J = 7.7 Hz, 1H), 7.57 (d, J = 8.1 Hz, 1H), 7.52 (d, J = 8.5 Hz, 2H), 7.43 (d, J = 8.5 Hz, 2H), 7.33 (d, J = 3.2 Hz, 1H), 7.24 – 7.12 (m, 2H), 6.66 (d, J = 3.1 Hz, 1H), 1.39 (s, 9H); ^{13}C NMR (100 MHz, $CDCl_3$) δ 149.5, 137.2, 136.0, 129.2, 128.1, 126.5, 124.0, 122.2, 121.0, 120.2, 110.6, 103.2, 34.6, 31.4; HRMS (ESI) m/z calc. for $C_{18}H_{20}N$ $[M+H]^+$: 250.1590, found: 250.1593.

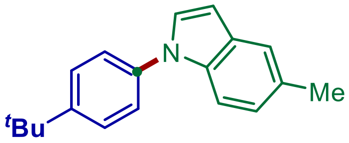

1-(4-(Tert-butyl)phenyl)-5-methyl-1H-indole (150): yellow oil; ^{1}H NMR (400 MHz, $CDCl_3$) δ 7.51 (d, J = 8.5 Hz, 2H), 7.48 – 7.44 (m, 2H), 7.41 (d, J = 8.5 Hz, 2H), 7.28 (d, J = 3.2 Hz, 1H), 7.03 (d, J = 8.7 Hz, 1H), 6.57 (d, J = 3.2 Hz, 1H), 2.46 (s, 3H), 1.38 (s, 9H); ^{13}C NMR (100 MHz, $CDCl_3$) δ 149.3, 137.4, 134.3, 129.5, 129.4, 128.0, 126.4, 123.8, 128.7, 120.7, 110.3, 102.7, 34.6, 31.4, 21.4; HRMS (ESI) m/z calc. for $C_{19}H_{22}N$ $[M+H]^+$: 264.1747, found: 264.1750.

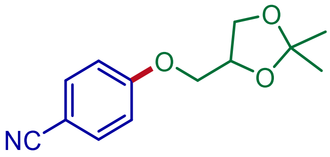

4-((2,2-Dimethyl-1,3-dioxolan-4-yl)methoxy)benzonitrile (151): yellow oil; ^{1}H NMR (400 MHz, $CDCl_3$) δ 7.59 (d, J = 9.0 Hz, 2H), 6.98 (d, J = 9.0 Hz, 2H), 4.58 – 4.07 (m, 1H), 4.18 (dd, J = 8.6, 6.5 Hz, 1H), 4.09 (dd, J = 9.6, 5.4 Hz, 1H), 4.01 (dd, J = 9.6, 5.6 Hz, 1H), 3.90 (dd, J = 8.6, 5.7 Hz, 1H), 1.46 (s, 3H), 1.41 (s, 3H); ^{13}C NMR (100 MHz, $CDCl_3$) δ 161.8, 134.0, 119.1, 115.3, 110.0, 104.5, 73.7, 69.0, 66.6, 26.8, 25.3; HRMS (APCI) m/z calc. for $C_{13}H_{16}NO_3$ $[M+H]^+$: 234.1125, found: 234.1121.

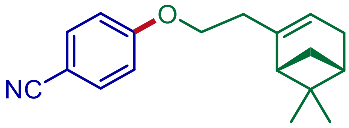

4-(2-((1R,5S)-6,6-Dimethylbicyclo[3.1.1]hept-2-en-2-yl)ethoxy)benzonitrile (152): yellow oil; ^{1}H NMR (400 MHz, $CDCl_3$) δ 7.56 (d, J = 8.9 Hz, 2H), 6.92 (d, J = 8.9 Hz, 2H), 5.43 – 5.25 (m, 1H), 4.01 (t, J = 6.9 Hz, 2H), 2.51 – 2.41 (m, 2H), 2.41 – 2.35 (m, 1H), 2.33 – 2.16 (m, 3H), 2.13 – 2.06 (m, 2H), 1.28 (s, 3H), 0.82 (s, 3H); ^{13}C NMR (100 MHz, $CDCl_3$) δ 162.3, 143.9,

133.9, 129.1, 119.1, 115.2, 103.7, 66.7, 45.9, 40.7, 36.2, 31.6, 31.4, 26.3, 21.2; HRMS (APCI) m/z calc. for $C_{18}H_{22}NO$ [M+H]$^+$: 268.1696, found: 268.1693.

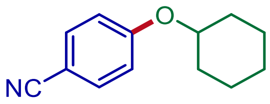

4-(Cyclohexyloxy)benzonitrile (153): yellow oil; ^{1}H NMR (400 MHz, $CDCl_3$) δ 7.55 (d, *J* = 8.9 Hz, 2H), 6.92 (d, *J* = 8.9 Hz, 2H), 4.41 – 4.22 (m, 1H), 2.04 – 1.90 (m, 2H), 1.88 – 1.74 (m, 2H), 1.61 – 1.51 (m, 3H), 1.46 – 1.29 (m, 3H); ^{13}C NMR (100 MHz, $CDCl_3$) δ 161.3, 134.0, 119.4, 116.2, 103.3, 75.7, 31.5, 25.4, 23.6; HRMS (ESI) m/z calc. for $C_{13}H_{16}NO$ [M+H]$^+$: 202.1226, found: 202.1229.

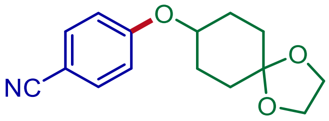

4-((1,4-Dioxaspiro[4.5]decan-8-yl)oxy)benzonitrile (154): yellow oil; ^{1}H NMR (600 MHz, $CDCl_3$) δ 7.57 (d, *J* = 8.6 Hz, 2H), 6.94 (d, *J* = 8.7 Hz, 2H), 4.59 – 3.44 (m, 1H), 3.99 – 3.88 (m, 4H), 2.02 – 1.92 (m, 4H), 1.92 – 1.86 (m, 2H), 1.67 – 1.61 (m, 2H); ^{13}C NMR (100 MHz, $CDCl_3$) δ 134.0, 116.2, 108.3, 68.1, 64.2, 32.0, 31.6, 28.0; HRMS (APCI) m/z calc. for $C_{15}H_{18}NO_3$ [M+H]$^+$: 260.1281, found: 260.1284.

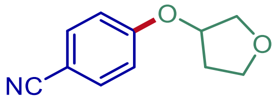

4-((Tetrahydrofuran-3-yl)oxy)benzonitrile (155): yellow oil; ^{1}H NMR (400 MHz, $CDCl_3$) δ 7.59 (d, *J* = 8.9 Hz, 2H), 6.92 (d, *J* = 8.9 Hz, 2H), 5.12 – 4.90 (m, 1H), 4.07 – 3.89 (m, 4H), 2.36 – 2.16 (m, 1H), 2.21 – 2.08 (m, 1H); ^{13}C NMR (100 MHz, $CDCl_3$) δ 160.7, 134.1, 119.1, 115.9, 104.2, 77.9, 72.9, 67.2, 32.9; HRMS (APCI) m/z calc. for $C_{11}H_{12}NO_2$ [M+H]$^+$: 190.0863, found: 190.0866.

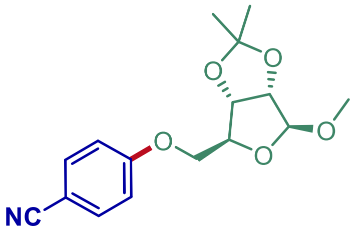

4-(((3aS,4S,6S,6aS)-6-Methoxy-2,2-dimethyltetrahydrofuro[3,4-d][1,3]dioxol-4-yl)methoxy) benzonitrile (156): yellow oil; ^{1}H NMR (400 MHz, $CDCl_3$) δ 7.59 (d, *J* = 8.8 Hz, 2H), 6.97 (d, *J* = 8.8 Hz, 2H), 4.78 (d, *J* = 5.9 Hz, 1H), 4.64 (d, *J* = 5.9 Hz, 1H), 4.57 – 4.50 (m, 1H), 4.16 –

4.0 (m, 1H), 3.62 (d, J = 3.3 Hz, 1H), 3.33 (s, 3H), 3.27 (d, J = 2.6 Hz, 1H), 1.51 (s, 3H), 1.34 (s, 3H); ^{13}C NMR (100 MHz, $CDCl_3$) δ 161.6, 134.0, 119.9, 115.3, 112.7, 109.9, 109.5, 104.5, 85.0, 84.2, 81.9, 68.6, 55.0, 26.4, 24.9; HRMS (ESI) m/z calc. for $C_{16}H_{20}NO_5$ $[M+H]^+$: 306.1336, found: 306.1332.

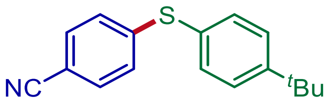

4-((4-(Tert-butyl)phenyl)thio)benzonitrile (157): yellow oil; ^{1}H NMR (400 MHz, $CDCl_3$) δ 7.43 (t, J = 4.3 Hz, 6H), 7.13 (d, J = 8.6 Hz, 2H), 1.34 (s, 9H); ^{13}C NMR (100 MHz, $CDCl_3$) δ 153.0, 146.4, 134.5, 132.3, 127.1, 127.0, 126.9, 118.9, 108.4, 34.8, 31.3; HRMS (APCI) m/z calc. for $C_{17}H_{18}NS$ $[M+H]^+$: 268.1154, found: 268.1157.

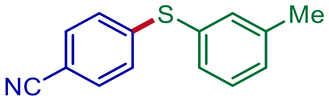

4-(m-Tolylthio)benzonitrile (158): yellow oil; ^{1}H NMR (400 MHz, $CDCl_3$) δ 7.45 (d, J = 8.4 Hz, 2H), 7.32 (s, 1H), 7.31 – 7.28 (m, 2H), 7.23 (d, J = 8.2 Hz, 1H), 7.14 (d, J = 8.4 Hz, 2H), 2.36 (s, 3H); ^{13}C NMR (100 MHz, $CDCl_3$) δ 146.0, 139.9, 135.1, 132.4, 131.6, 130.6, 130.4, 129.8, 127.2, 118.9, 108.6, 21.3; HRMS (APCI) m/z calc. for $C_{14}H_{12}NS$ $[M+H]^+$: 226.0685 found: 226.0689.

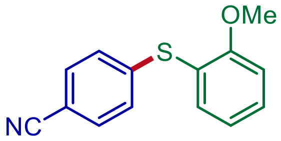

4-((2-Methoxyphenyl)thio)benzonitrile (159): yellow oil; ^{1}H NMR (400 MHz, $CDCl_3$) δ 7.51 – 7.40 (m, 4H), 7.11 (d, J = 8.4 Hz, 2H), 7.03 – 6.96 (m, 2H), 3.80 (s, 3H); ^{13}C NMR (100 MHz, $CDCl_3$) δ 159.6, 145.2, 136.6, 132.2, 131.7, 126.8, 121.6, 119.0, 118.0, 111.8, 108.2, 56.0; HRMS (APCI) m/z calc. for $C_{14}H_{12}NOS$ $[M+H]^+$: 242.0634, found: 242.0637.

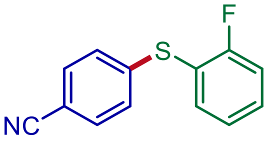

4-((2-Fluorophenyl)thio)benzonitrile (160): yellow oil; ^{1}H NMR (400 MHz, $CDCl_3$) δ 7.58 – 7.41 (m, 4H), 7.27 – 7.20 (m, 2H), 7.19 – 7.16 (m, 2H); ^{13}C NMR (100 MHz, $CDCl_3$) δ 162.6 (d, J = 249.8 Hz), 143.7, 136.7, 132.5, 132.1 (d, J = 8.0 Hz), 127.2, 125.3 (d, J = 4.0 Hz), 118.7 ,

117.9 (d, J = 18.2 Hz), 116.7 (d, J = 22.5 Hz), 109.1; ^{19}F NMR (376 MHz, $CDCl_3$) δ -105.88 (s, F); HRMS (APCI) m/z calc. for $C_{13}H_9FNS$ $[M+H]^+$: 230.0434, found: 230.0438.

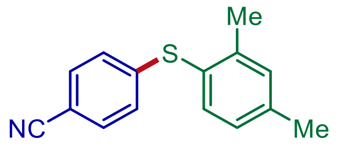

4-((2,4-Dimethylphenyl)thio)benzonitrile (161): yellow oil; ^{1}H NMR (400 MHz, $CDCl_3$) δ 7.42 (ddd, J = 7.8, 4.1, 2.2 Hz, 3H), 7.17 (s, 1H), 7.07 (d, J = 7.8 Hz, 1H), 7.00 (d, J = 8.6 Hz, 2H); ^{13}C NMR (101 MHz, $CDCl_3$) δ 146.26 (s), 142.56 (s), 140.72 (s), 136.64 (s), 132.25 (d, J = 12.2 Hz), 128.23 (s), 125.97 (s), 125.51 (s), 118.98 (s), 107.94 (s), 21.27 (s), 20.58 (s); HRMS (APCI) m/z calc. for $C_{15}H_{14}NS$ $[M+H]^+$: 240.0841, found: 240.0844.

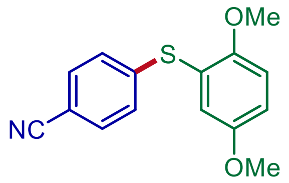

4-((2,5-Dimethoxyphenyl)thio)benzonitrile (162): yellow oil; ^{1}H NMR (400 MHz, $CDCl_3$) δ 7.46 (d, J = 8.4 Hz, 2H), 7.14 (d, J = 8.4 Hz, 2H), 7.02 (d, J = 2.9 Hz, 1H), 6.96 (d, 5.9 Hz, 2H), 3.76 (s, 3H), 3.75 (s, 3H); ^{13}C NMR (100 MHz, $CDCl_3$) δ 153.9, 153.8, 144.7, 132.2, 127.1, 121.3, 118.9, 118.8, 116.6, 112.8, 108.4, 56.5, 55.8; HRMS (APCI) m/z calc. for $C_{15}H_{14}NO_2S$ $[M+H]^+$: 272.0740, found: 272.0743.

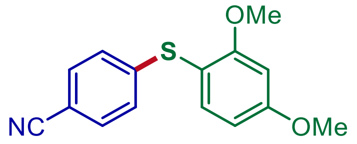

4-((2,4-Dimethoxyphenyl)thio)benzonitrile (163): yellow oil; ^{1}H NMR (400 MHz, $CDCl_3$) δ 7.50 – 7.37 (m, 3H), 7.08 – 6.99 (m, 2H), 6.56 (dd, J = 4.4, 2.1 Hz, 2H); ^{13}C NMR (100 MHz, $CDCl_3$) δ 163.2, 161.2, 146.6, 138.6, 132.1, 125.6, 119.1, 108.2, 107.6, 105.8, 99.6, 56.0, 55.6; HRMS (APCI) m/z calc. for $C_{15}H_{14}NO_2S$ $[M+H]^+$: 272.0740, found: 272.0741.

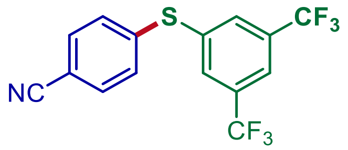

4-((3,5-Bis(trifluoromethyl)phenyl)thio)benzonitrile (164): yellow oil; ^{1}H NMR (400 MHz, $CDCl_3$) δ 7.83 (s, 3H), 7.63 (d, J = 8.6 Hz, 2H), 7.38 (d, J = 8.6 Hz, 2H); ^{13}C NMR (100 MHz,

$CDCl_3$) δ 140.9, 136.7, 133.1, 133.1 (q, J = 33.8 Hz), 131.8 (q, J = 2.8 Hz), 130.2, 122.7 (q, J = 273.2 Hz), 122.1 (dq, J = 7.5, 3.7 Hz), 118.1, 111.4; ^{19}F NMR (376 MHz, $CDCl_3$) δ -63.06 (s, CF_3); HRMS (APCI) m/z calc. for $C_{15}H_8F_6NS$ [M+H]$^+$: 348.0276, found: 348.0280.

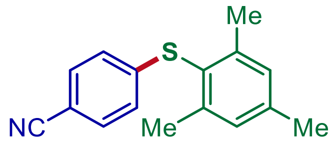

4-(Mesitylthio)benzonitrile (165): yellow oil; ^{1}H NMR (400 MHz, $CDCl_3$) δ 7.40 (dd, J = 8.6, 1.8 Hz, 2H), 7.03 (s, 2H), 6.93 (dd, J = 8.6, 1.7 Hz, 2H), 2.34 (s, 3H), 2.34 (s, 3H), 2.32 (s, 3H); ^{13}C NMR (100 MHz, $CDCl_3$) δ 146.0, 143.7, 140.4, 132.4, 129.8, 125.2, 124.7, 119.1, 107.6, 21.5, 21.2; HRMS (APCI) m/z calc. for $C_{16}H_{16}NS$ [M+H]$^+$: 254.0998, found: 254.1001.

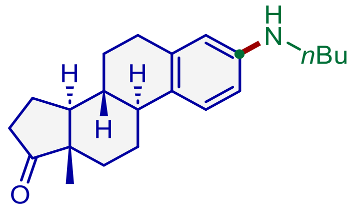

(8R,9S,13S,14S)-3-(Butylamino)-13-Methyl-6,7,8,9,11,12,13,14,15,16-decahydro-17H-cyclopenta[a]phenanthren-17-one (166): yellow oil; ^{1}H NMR (400 MHz, $CDCl_3$) δ 7.09 (d, J = 8.4 Hz, 1H), 6.44 (dd, J = 8.4, 2.4 Hz, 1H), 6.36 (d, J = 2.2 Hz, 1H), 3.44 (br, 1H), 3.09 (t, J = 7.1 Hz, 2H), 2.92 - 2.84 (m, 2H), 2.59 - 2.42 (m, 1H), 2.42 - 2.32 (m, 1H), 2.22 (t, J = 10.2 Hz, 1H), 2.11 - 2.06 (m, 2H), 2.01 - 1.91 (m, 2H), 1.75 - 1.54 (m, 11H), 0.95 (t, J = 7.3 Hz, 3H), 0.90 (s, 3H); ^{13}C NMR (100 MHz, $CDCl_3$) δ 221.1, 146.6, 137.2, 128.6, 126.1, 112.75, 110.9, 50.4, 48.1, 44.0, 43.9, 38.6, 35.9, 31.9, 31.8, 29.7, 26.7, 26.0, 21.6, 20.3, 14.0, 13.9; HRMS (ESI) m/z calc. for $C_{22}H_{32}N_2O$ [M+H]$^+$: 326.2478, found: 326.2480.

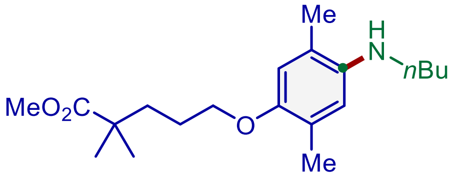

Methyl 5-(4-(butylamino)-2,5-dimethylphenoxy)-2,2-dimethylpentanoate (167): yellow oil; ^{1}H NMR (400 MHz, $CDCl_3$) δ 6.59 (s, 1H), 6.44 (s, 1H), 3.84 (d, J = 4.5 Hz, 2H), 3.65 (s, 3H), 3.09 (t, J = 7.1 Hz, 2H), 2.91 - 2.86 (m, 1H), 2.19 (s, 3H), 2.09 (s, 3H), 1.75 - 1.65 (m, 4H), 1.67 - 1.55 (m, 2H), 1.52 - 1.36 (m, 2H), 1.21 (s, 6H), 0.96 (t, J = 7.3 Hz, 3H); ^{13}C NMR (100 MHz, $CDCl_3$) δ 178.4, 148.8, 140.5, 125.3, 120.0, 115.8, 113.3, 69.6, 51.7, 44.6, 42.1, 37.2,

32.0, 25.5, 25.2, 20.4, 17.4, 16.1, 14.0; HRMS (ESI) m/z calc. for $C_{20}H_{34}NO_3$ $[M+H]^+$: 336.2533, found: 336.2535.

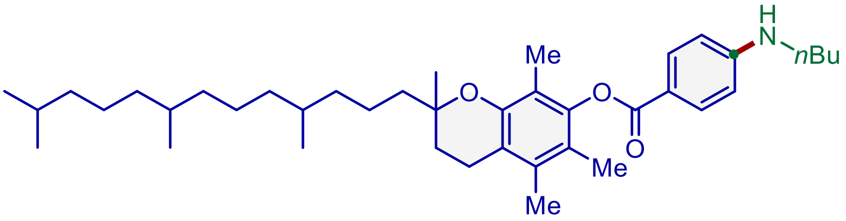

2,5,6,8-Tetramethyl-2-(4,8,12-trimethyltridecyl)chroman-7-yl 4-(butylamino) benzoate (168): yellow oil; ^{1}H NMR (400 MHz, $CDCl_3$) δ 7.22 (d, J = 8.4 Hz, 2H), 6.55 (d, J = 8.4 Hz, 2H), 4.48 (s, 2H), 3.06 (t, J = 7.1 Hz, 2H), 2.51 (t, J = 6.8 Hz, 2H), 2.15 (s, 3H), 2.09 (s, 3H), 2.02 (s, 3H), 1.79 - 1.69 (m, 2H), 1.55 - 1.48 (m, 6H), 1.38 - 1.20 (m, 11H), 1.10 - 0.96 (m, 8H), 0.89 (t, J = 7.3 Hz, 3H), 0.81 - 0.76 (m, 15H); ^{13}C NMR (100 MHz, $CDCl_3$) δ 148.4, 148.3, 147.7, 129.7, 128.1, 126.5, 126.1, 122.8, 117.5, 112.6, 75.1, 74.8, 43.8 , 40.1, 39.4, 37.5, 37.4, 32.8, 31.7 , 31.6, 31.4, 28.0, 24.8, 24.5, 23.9, 22.7, 22.6 , 21.1, 20.7, 20.3, 19.8, 19.6, 13.9, 13.0, 12.1, 11.8; HRMS (ESI) m/z calc. for $C_{40}H_{64}NO_3$ $[M+H]^+$: 606.4881, found: 606.4880.

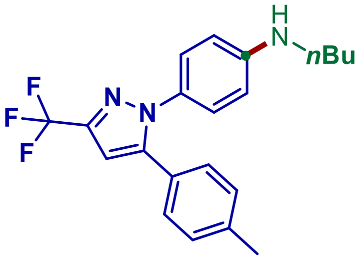

N-Butyl-4-(5-(p-tolyl)-3-(trifluoromethyl)-1H-pyrazol-1-yl)aniline (169): yellow oil; ^{1}H NMR (400 MHz, $CDCl_3$) δ 7.19 – 7.13 (m, 5H), 7.11 (d, J = 8.7 Hz, 2H), 6.70 (s, 1H), 6.54 (d, J = 8.6 Hz, 2H), 3.79 (br, 1H), 3.13 (t, J = 7.1 Hz, 2H), 2.36 (s, 3H), 1.63 (dt, J = 14.3, 6.9 Hz, 2H), 1.51 – 1.40 (m, 2H), 0.98 (t, J = 7.3 Hz, 3H); ^{13}C NMR (100 MHz, $CDCl_3$) δ 148.5, 144.4, 142.32 (d, J = 38.0 Hz)138.6, 129.2,128.6, 126.7, 126.6, 125.5, 121.5 (d, J = 268.7 Hz), 112.3, 104.5, 43.6, 31.5, 21.3, 20.3, 13.9; ^{19}F NMR (376 MHz, $CDCl_3$) δ -62.67 (s, CF_3); HRMS (ESI) m/z calc. for $C_{21}H_{23}F_3N_3$ $[M+H]^+$: 374.1839, found: 374.1841.

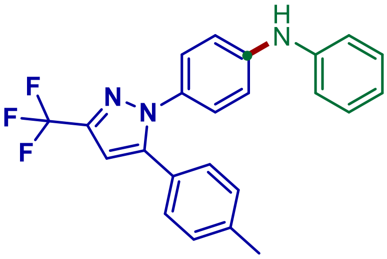

N-Phenyl-4-(5-(p-tolyl)-3-(trifluoromethyl)-1H-pyrazol-1-yl)aniline (170): yellow oil; ^{1}H NMR (400 MHz, $CDCl_3$) δ 7.33 – 7.23 (m, 3H), 7.21 – 7.13 (m, 6H), 7.08 (d, *J* = 8.0 Hz, 2H), 6.98 (d, *J* = 7.9 Hz, 2H), 6.69 (s, 1H), 5.81 (br, 1H), 2.35 (s, 3H); ^{13}C NMR (100 MHz, $CDCl_3$) δ 144.6, 143.7, 142.7 (q, *J* = 38.0 Hz). 141.9, 138.9, 132.0, 129.5, 129.4, 128.6, 126.7, 126.5, 122.1, 121.43 (q, *J* = 268.8 Hz), 119.0, 116.7, 104.9, 21.3; ^{19}F NMR (376 MHz, $CDCl_3$) δ -62.06 (s, CF_3); HRMS (ESI) m/z calc. for $C_{23}H_{19}F_3N_3$ $[M+H]^+$: 394.1526, found: 394.1530.

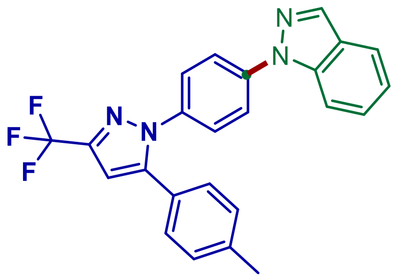

1-(4-(5-(p-Tolyl)-3-(trifluoromethyl)-1H-pyrazol-1-yl)phenyl)-1H-indazole (171): yellow oil; ^{1}H NMR (400 MHz, $CDCl_3$) δ 8.20 (s, 1H), 7.83 – 7.66 (m, 4H), 7.52 – 7.41 (m, 3H), 7.24 (t, *J* = 7.5 Hz, 1H), 7.20 – 7.10 (m, 4H), 6.75 (s, 1H), 2.35 (s, 3H); ^{13}C NMR (100 MHz, $CDCl_3$) δ 145.0, 143.4 (q, *J* = 38.4 Hz), 140.0, 139.4, 138.7, 137.3, 136.1, 129.6, 128.8, 127.6, 126.5, 126.1, 125.6, 122.7, 122.0, 121.6, 121.3 (q, *J* = 269.1 Hz).110.3, 105.6 (q, *J* = 1.8 Hz), 21.30; ^{19}F NMR (376 MHz, $CDCl_3$) δ -62.15 (s, CF_3); HRMS (ESI) m/z calc. for $C_{24}H_{18}F_3N_4$ $[M+H]^+$: 419.1478, found: 419.1480.

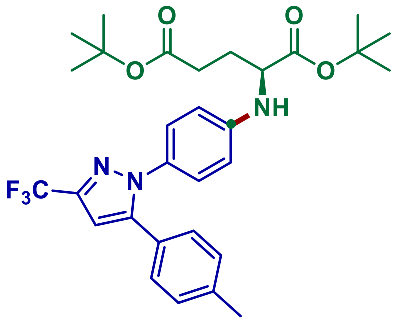

Di-tert-butyl (4-(5-(p-tolyl)-3-(trifluoromethyl)-1H-pyrazol-1-yl)phenyl)-L-glutamate (172): yellow oil; ^{1}H NMR (400 MHz, $CDCl_3$) δ 7.22 – 7.02 (m, 5H), 7.07 (s, 1H), 6.67 (s, 1H), 6.55 (d, *J* = 8.7 Hz, 2H), 4.37 (d, *J* = 8.8 Hz, 1H), 4.01 – 3.92 (m, 1H), 2.43 – 2.29 (m, 5H), 2.13 – 1.95 (m, 2H), 1.44 (s, 9H), 1.43 (s, 9H); ^{13}C NMR (100 MHz, $CDCl_3$) δ 172.4, 172.1, 147.0, 144.5, 142.4 (q, *J* = 38.0 Hz), 138.7, 130.2, 129.3, 128.6, 126.6, 126.1, 121.45 (q, *J* = 268.9 Hz), 113.3, 104.6, 82.2, 80.8, 56.6, 31.6, 28.1, 28.0, 27.8, 21.3; ^{19}F NMR (376 MHz, $CDCl_3$) δ -62.04 (s, CF_3); HRMS (ESI) m/z calc. for $C_{30}H_{37}F_3N_3O_4$$[M+H]^+$: 560.2731, found: 560.2727.

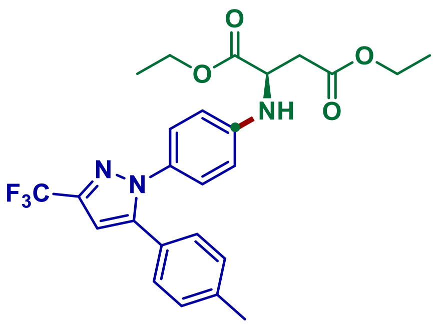

Diethyl (4-(5-(p-tolyl)-3-(trifluoromethyl)-1H-pyrazol-1-yl)phenyl)-D-aspartate (173): yellow oil; ^{1}H NMR (400 MHz, $CDCl_3$) δ 7.14 – 7.09 (m, 6H), 6.68 (s, 1H), 6.60 (d, *J* = 8.8 Hz, 2H), 4.65 (d, *J* = 8.7 Hz, 1H), 4.46 – 4.35 (m, 1H), 4.28 – 4.07 (m, 4H), 2.87 – 2.84 (m, 1H), 2.34 (s, 3H), 1.26 (s, 6H); ^{13}C NMR (100 MHz, $CDCl_3$) δ 171.9, 170.4, 146.3, 144.5, 142.33 (q, *J* = 38.5 Hz), 138.8, 130.6, 129.3, 128.6, 126.8, 126.5, 121.43 (q, *J* = 268.7 Hz), 113.6, 104.7, 61.8, 61.1, 53.3, 37.2, 29.7, 21.3, 14.1; ^{19}F NMR (376 MHz, $CDCl_3$) δ -62.07 (s, CF_3); HRMS (ESI) m/z calc. for $C_{25}H_{27}F_3N_3O_4[M+H]^+$: 490.1848, found: 490.1840.

7. Copies of ^{1}H NMR, ^{13}C NMR and ^{19}F NMR spectra of products

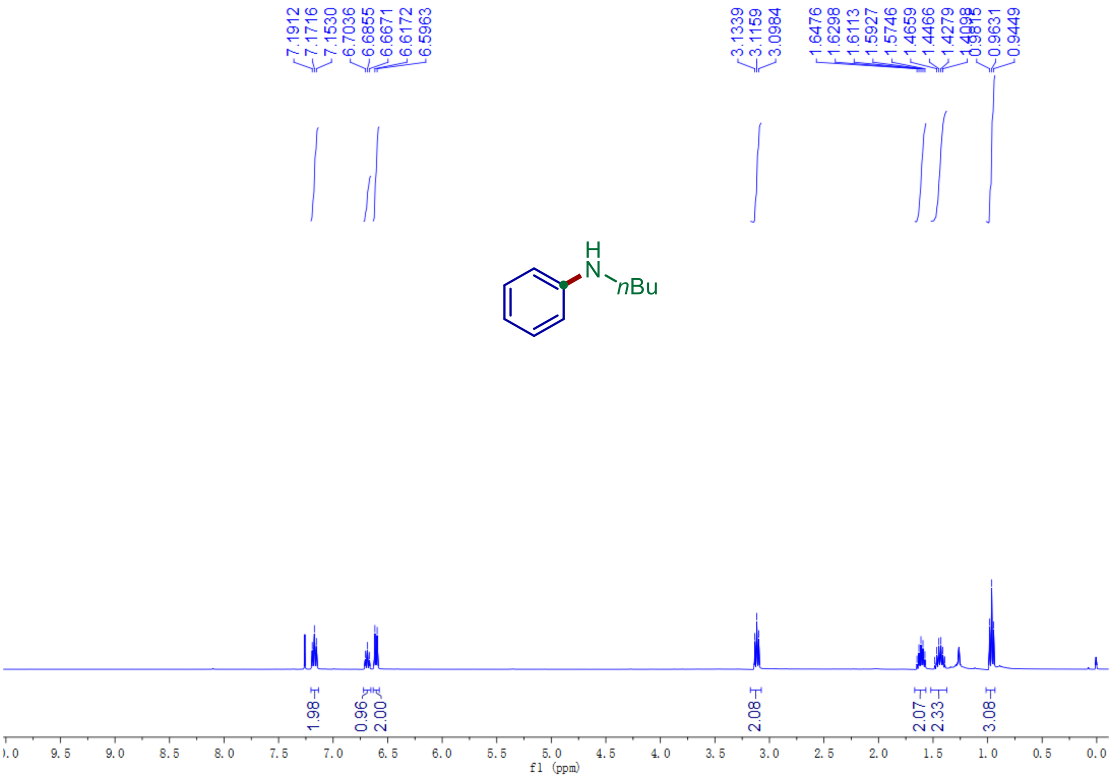

^{1}H NMR (400 MHz, $CDCl_3$) spectrum of compound 3

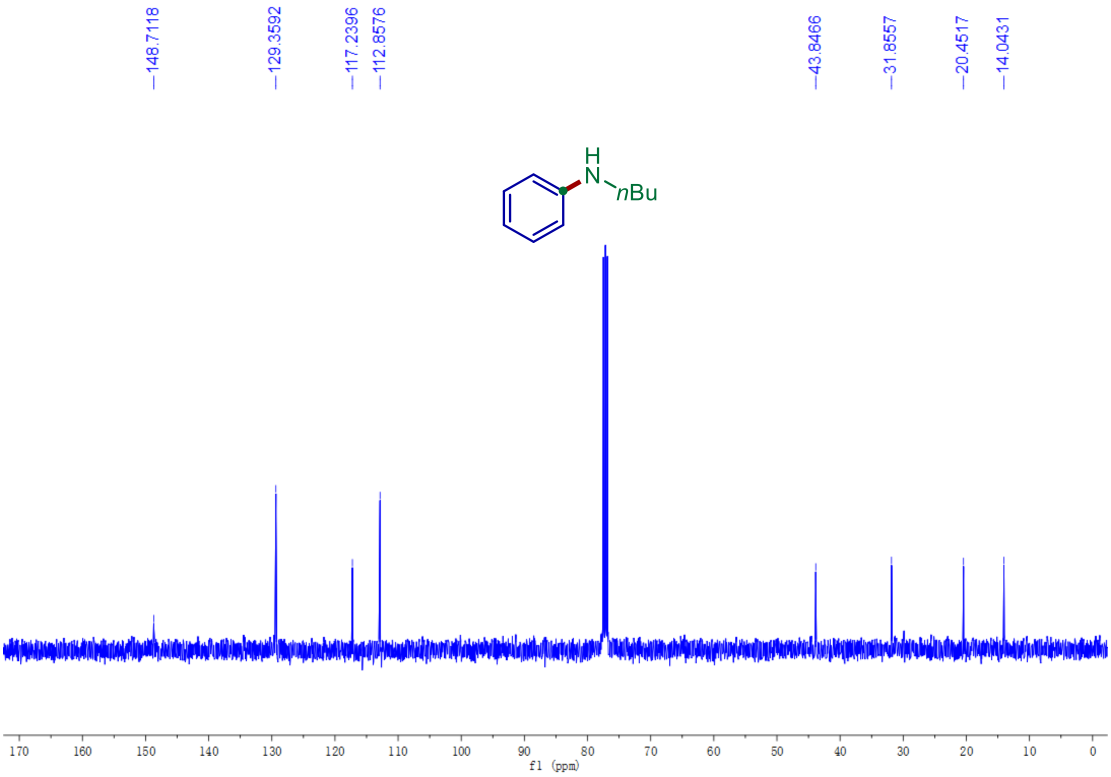

^{13}C NMR (100 MHz, $CDCl_3$) spectrum of compound 3

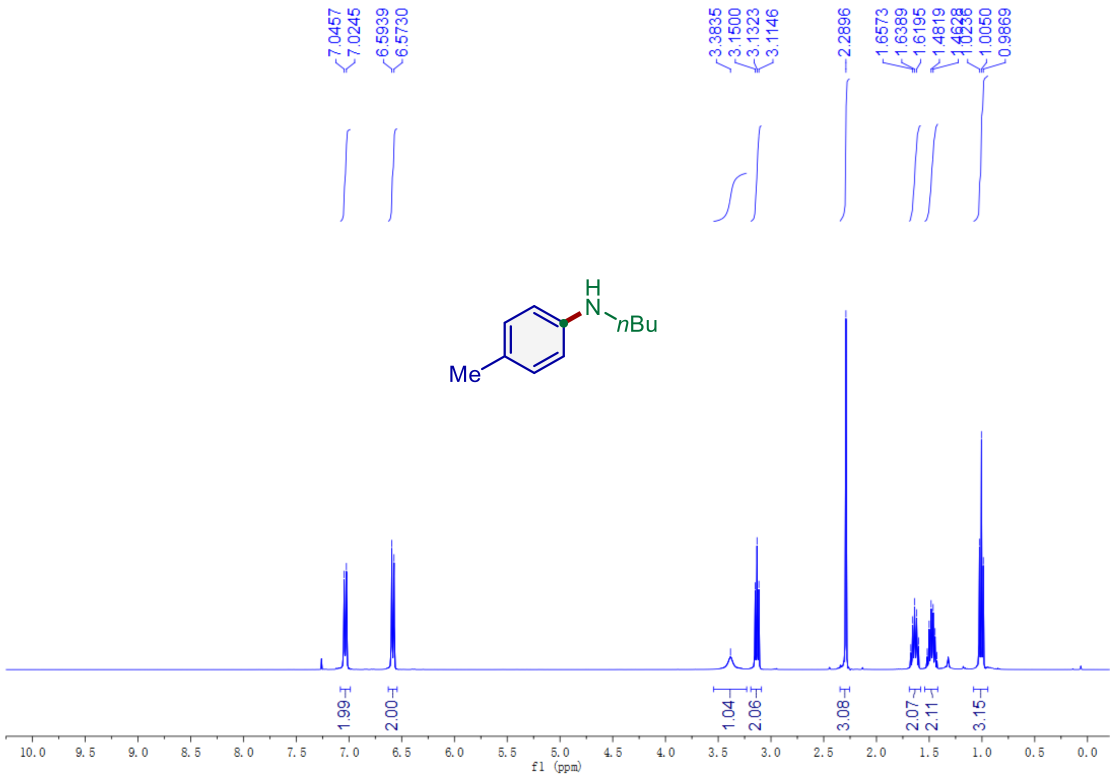

^{1}H NMR (400 MHz, $CDCl_3$) spectrum of compound 4

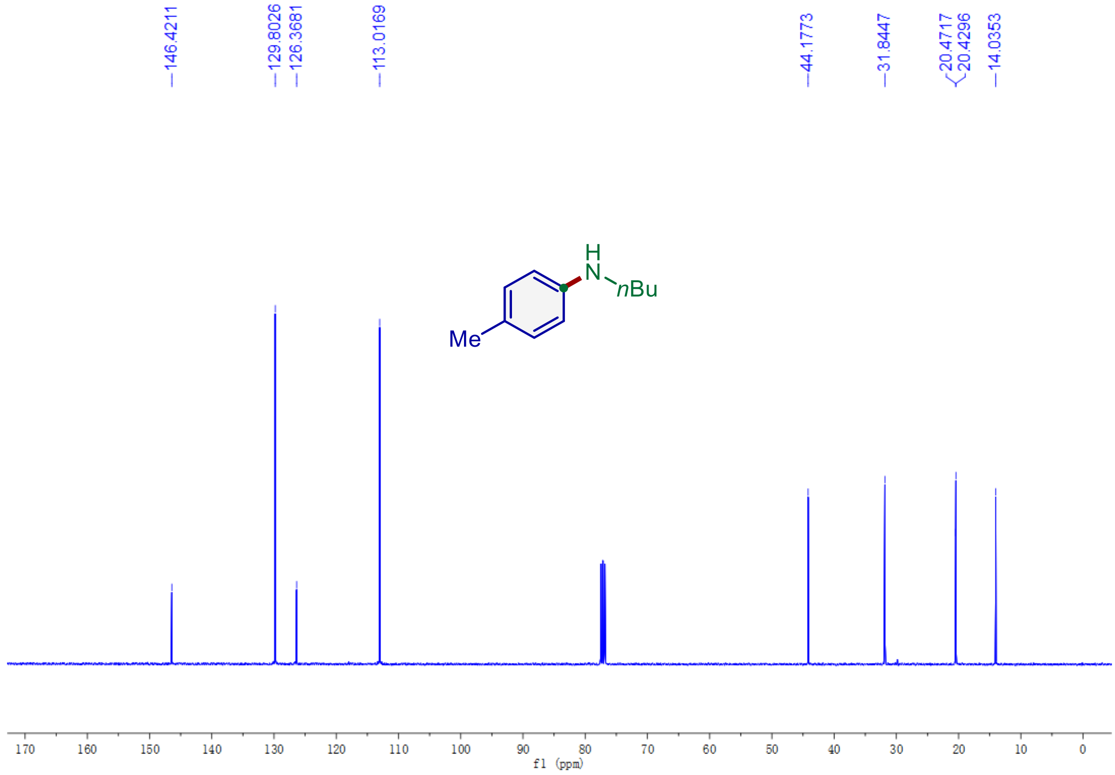

^{13}C NMR (100 MHz, $CDCl_3$) spectrum of compound 4

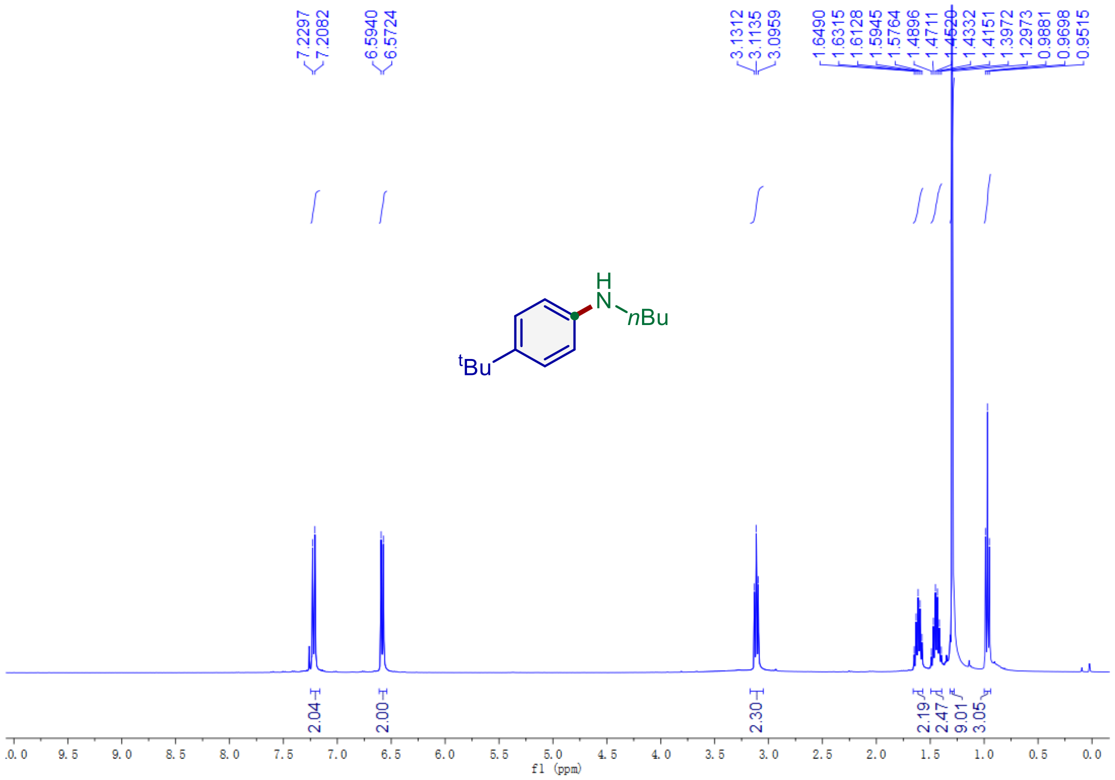

^{1}H NMR (400 MHz, $CDCl_3$) spectrum of compound 5

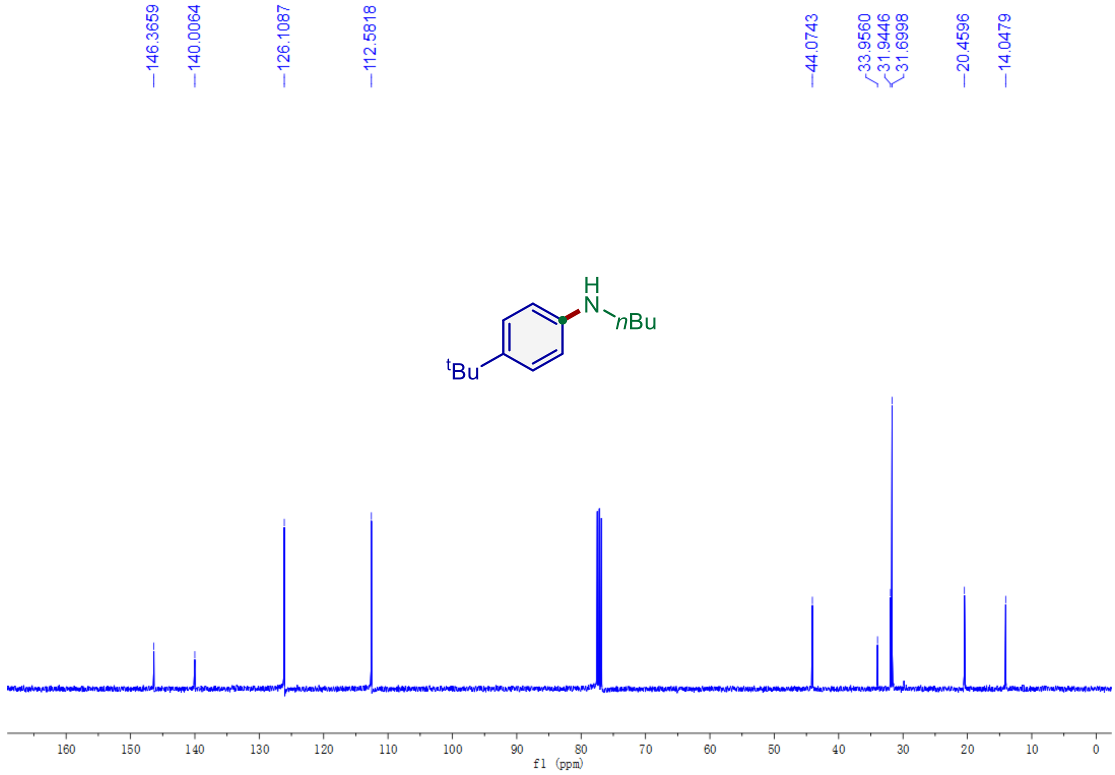

^{13}C NMR (100 MHz, $CDCl_3$) spectrum of compound 5

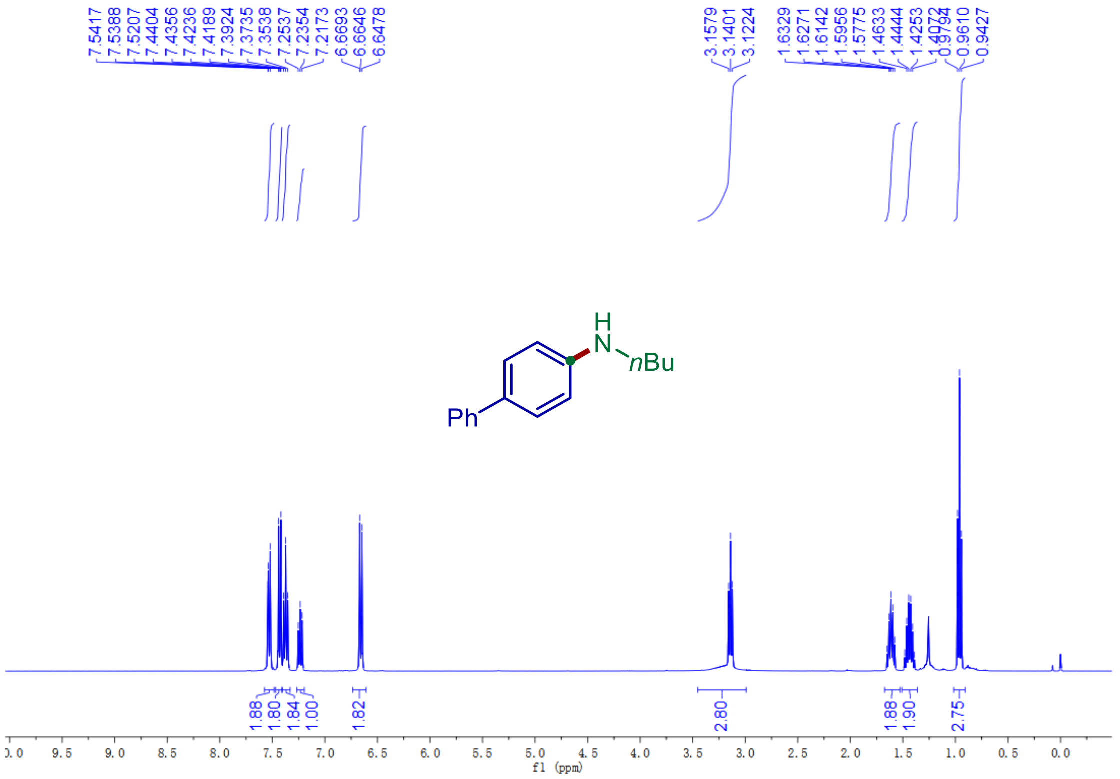

^{1}H NMR (400 MHz, $CDCl_3$) spectrum of compound 6

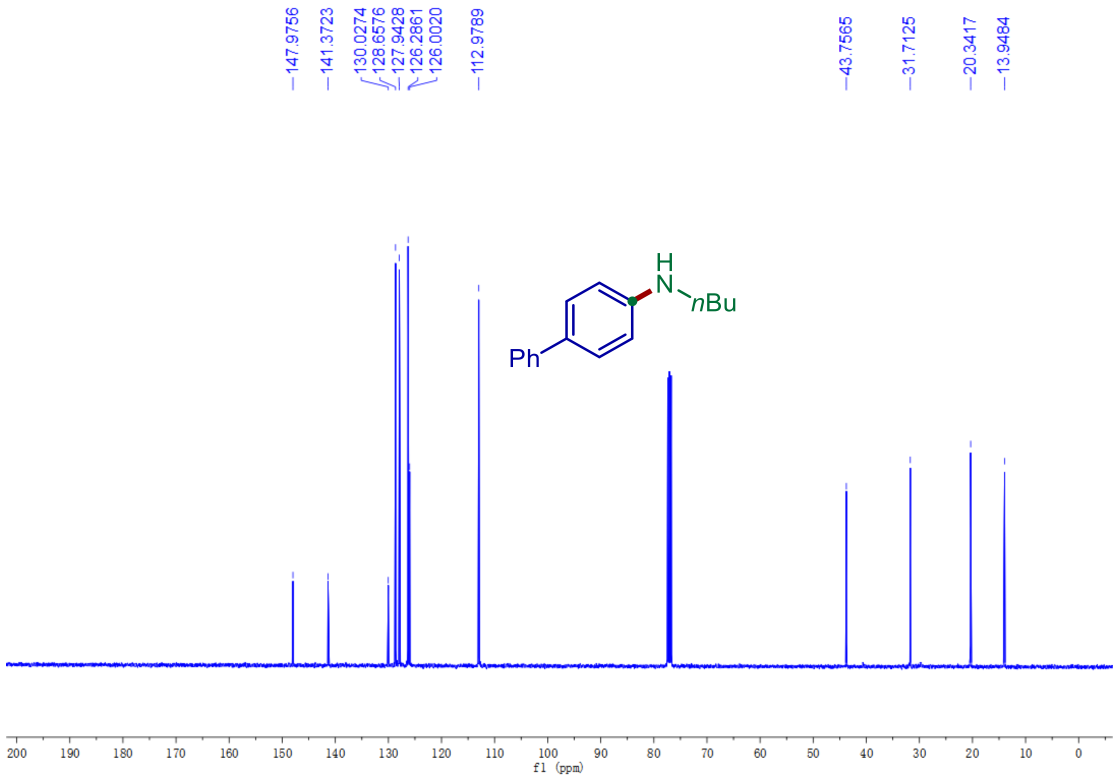

^{13}C NMR (100 MHz, $CDCl_3$) spectrum of compound 6

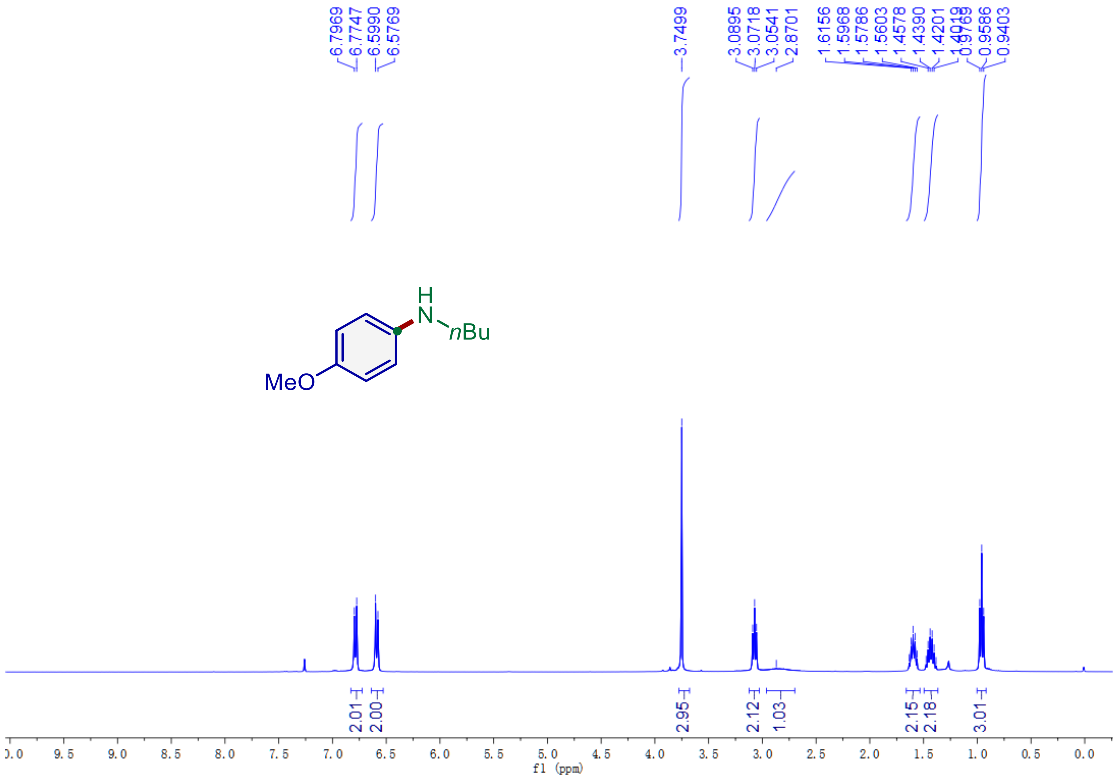

^{1}H NMR (400 MHz, $CDCl_3$) spectrum of compound 7

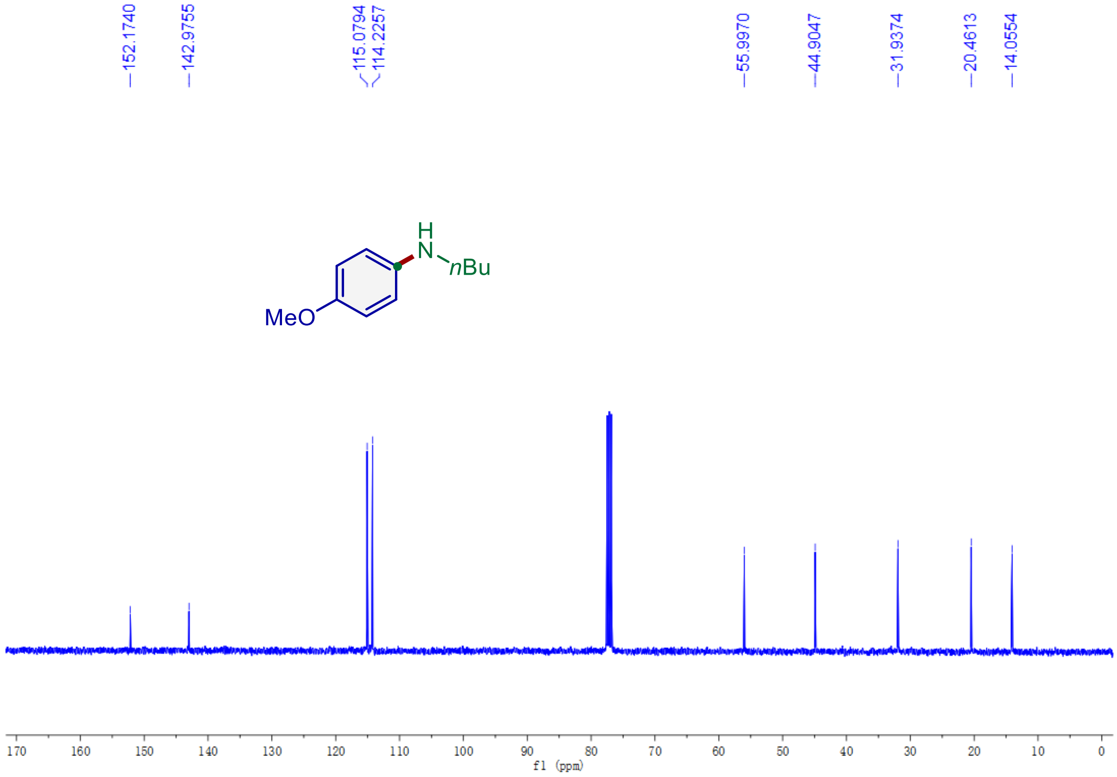

^{13}C NMR (100 MHz, $CDCl_3$) spectrum of compound 7

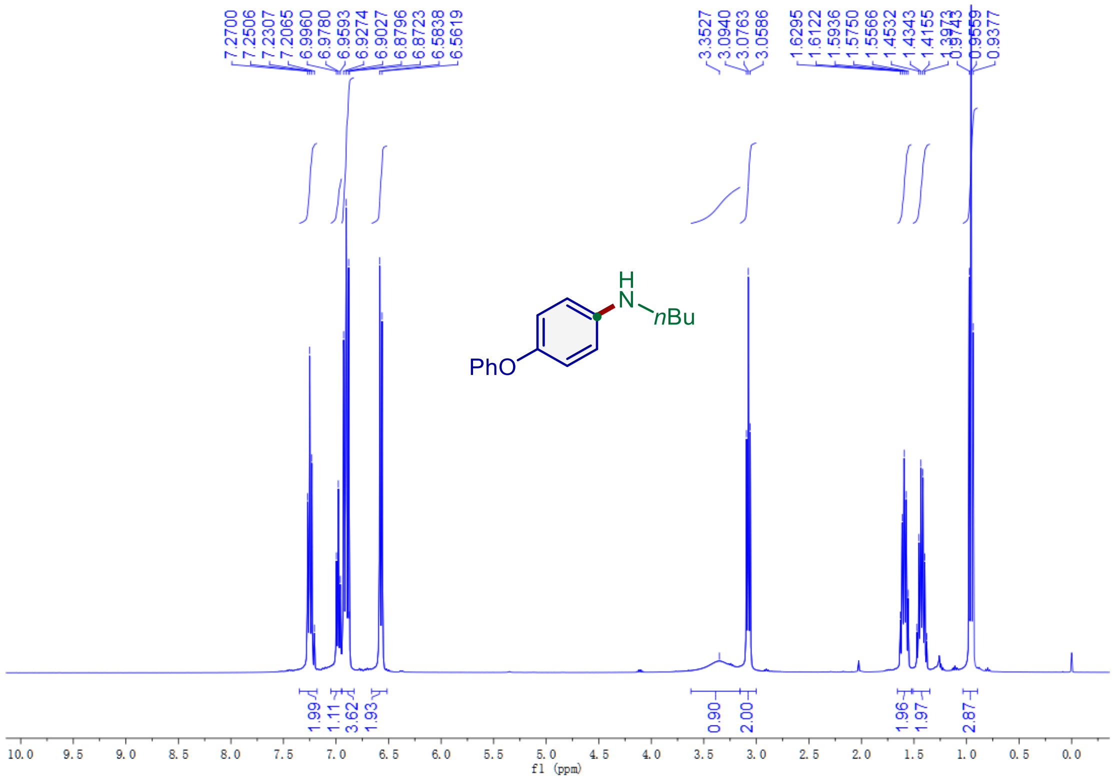

^{1}H NMR (400 MHz, $CDCl_3$) spectrum of compound 8

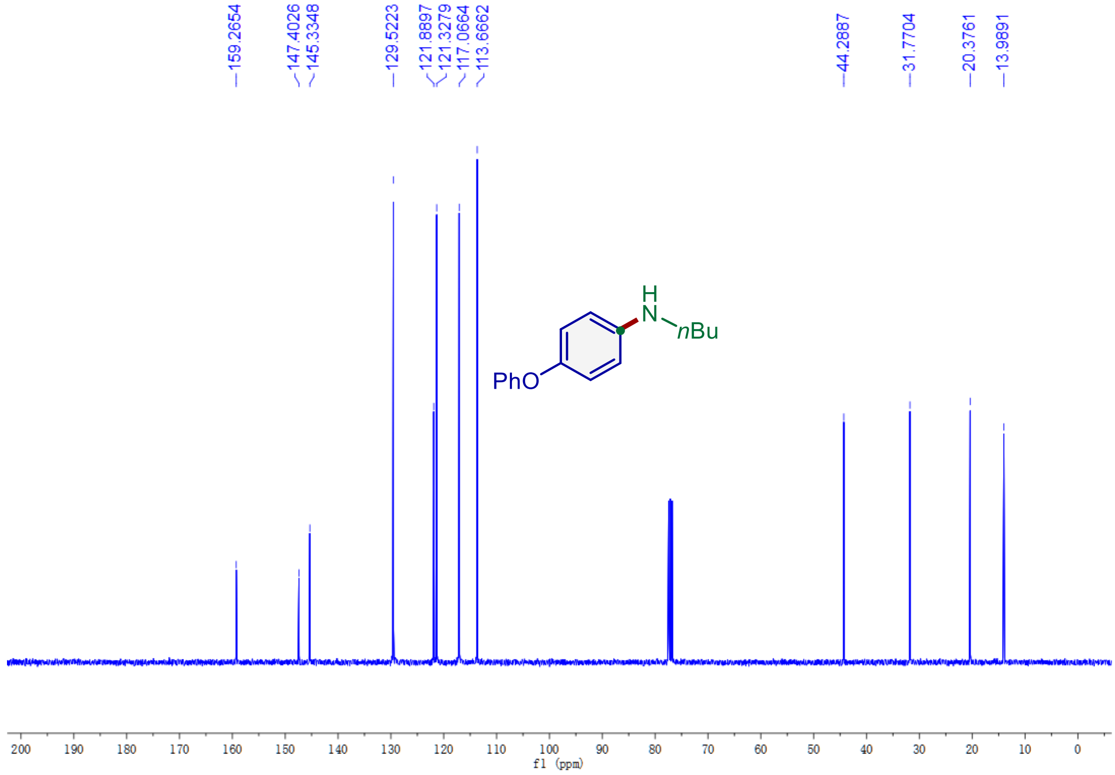

^{13}C NMR (100 MHz, $CDCl_3$) spectrum of compound 8

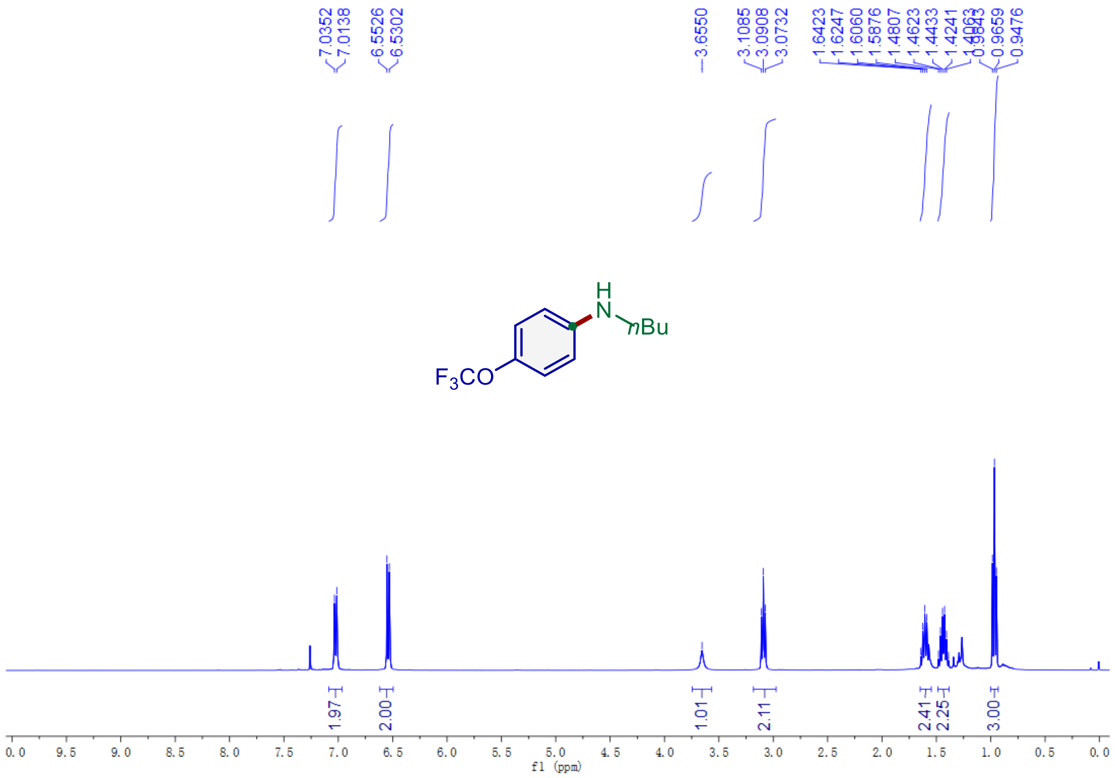

^{1}H NMR (400 MHz, $CDCl_3$) spectrum of compound 9

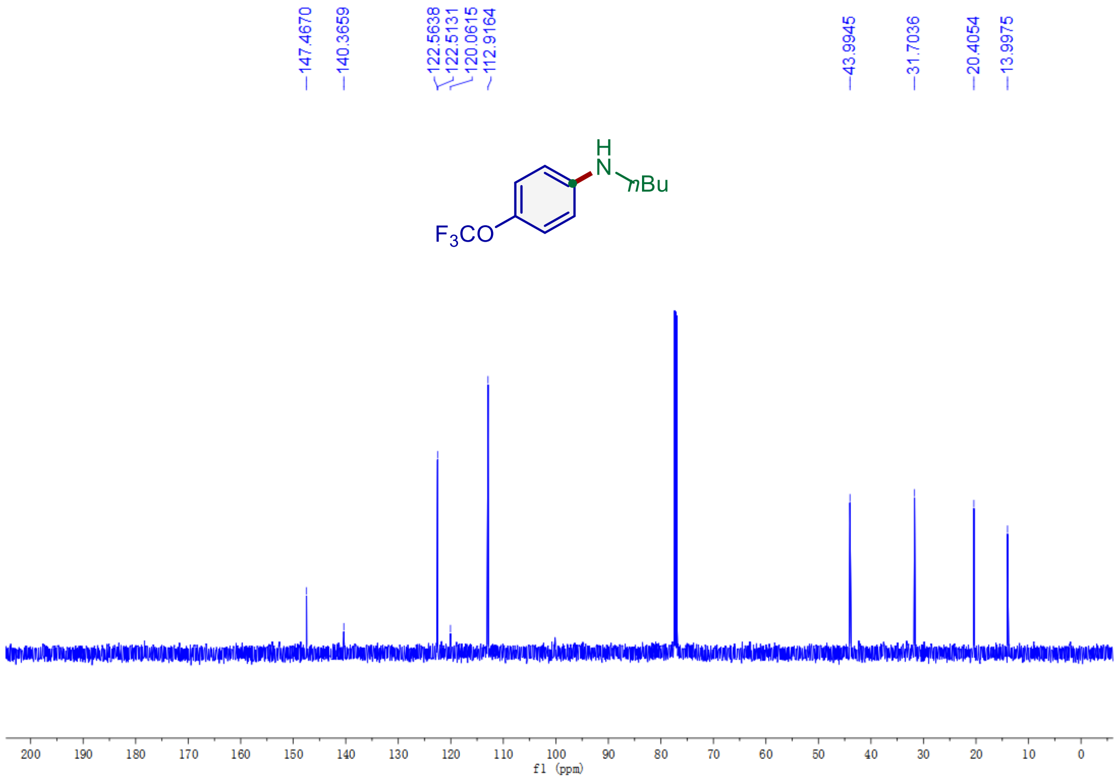

^{13}C NMR (100 MHz, $CDCl_3$) spectrum of compound 9

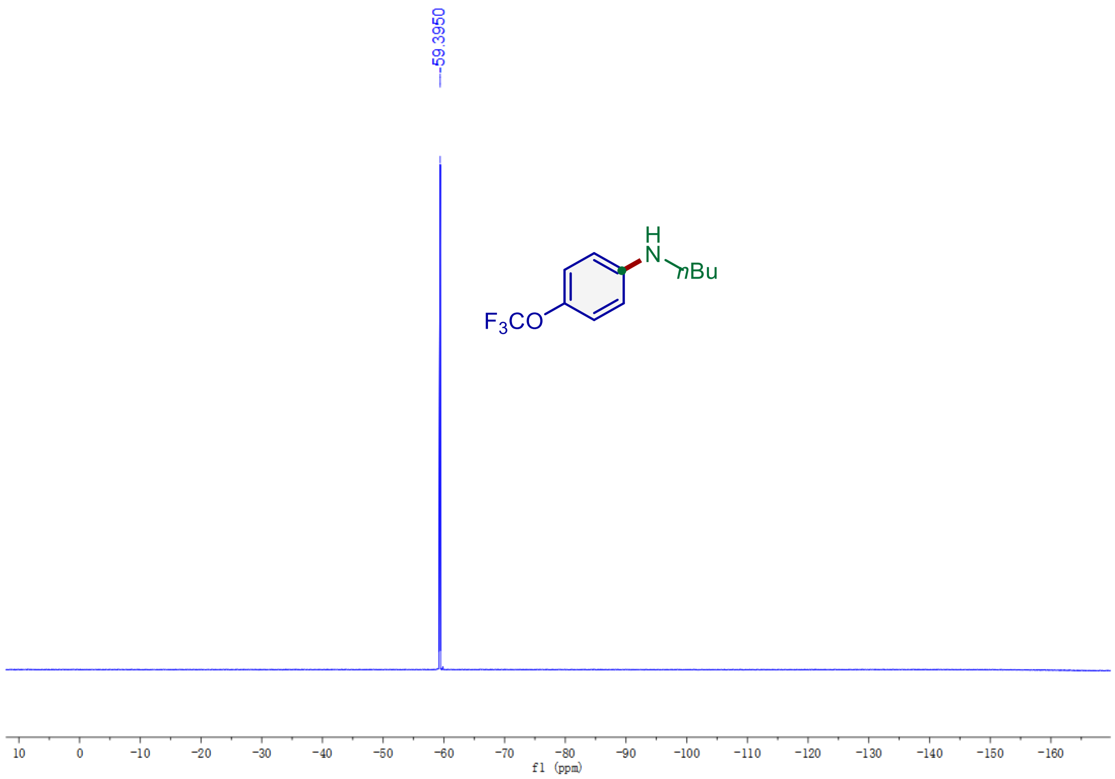

^{19}F NMR (376 MHz, $CDCl_3$) spectrum of compound 9

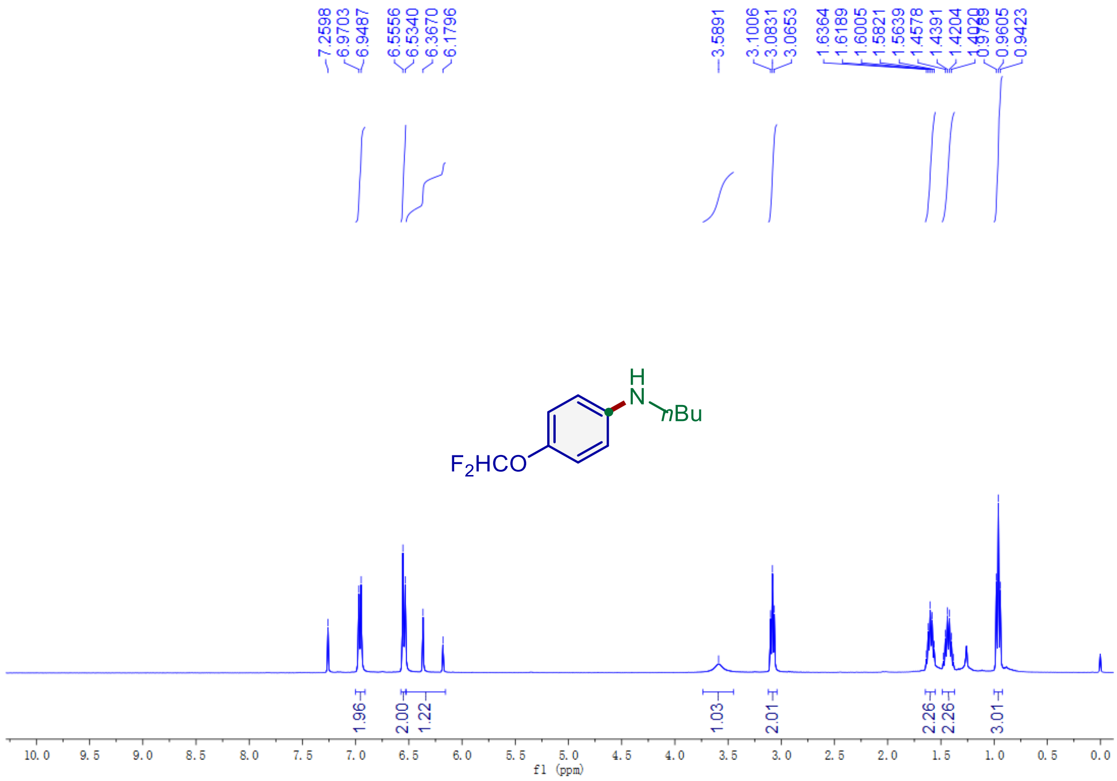

^{1}H NMR (400 MHz, $CDCl_3$) spectrum of compound 10

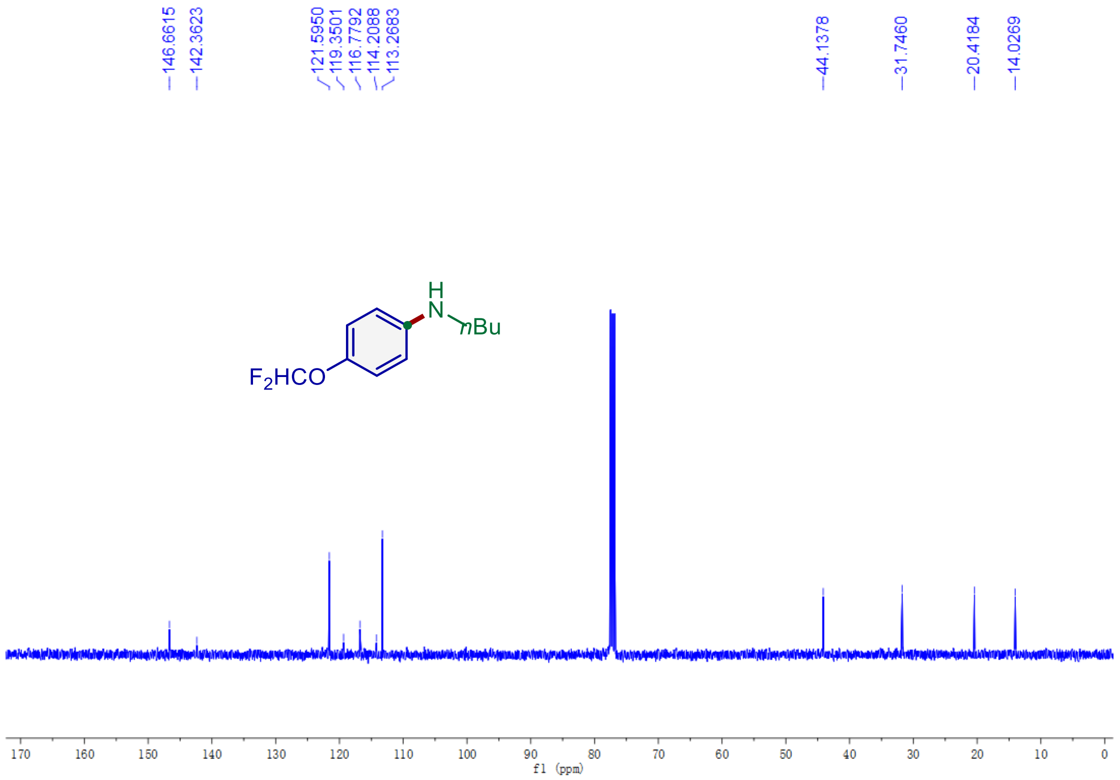

^{13}C NMR (100 MHz, $CDCl_3$) spectrum of compound 10

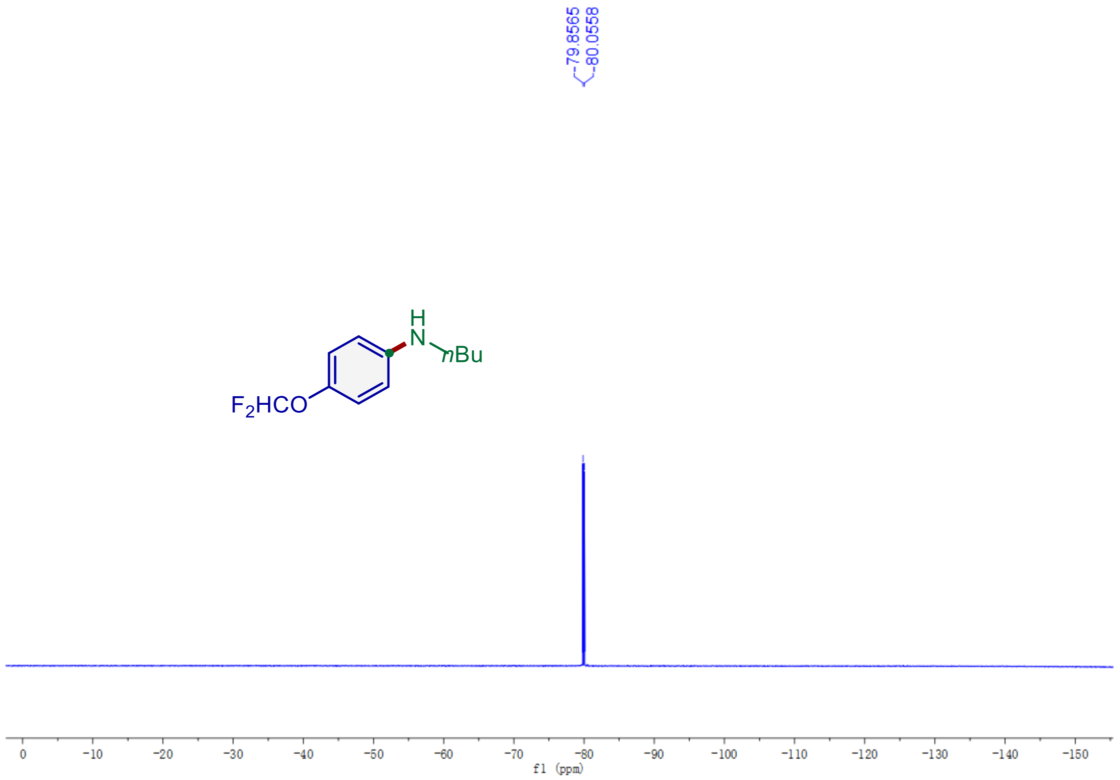

^{19}F NMR (376 MHz, $CDCl_3$) spectrum of compound 10

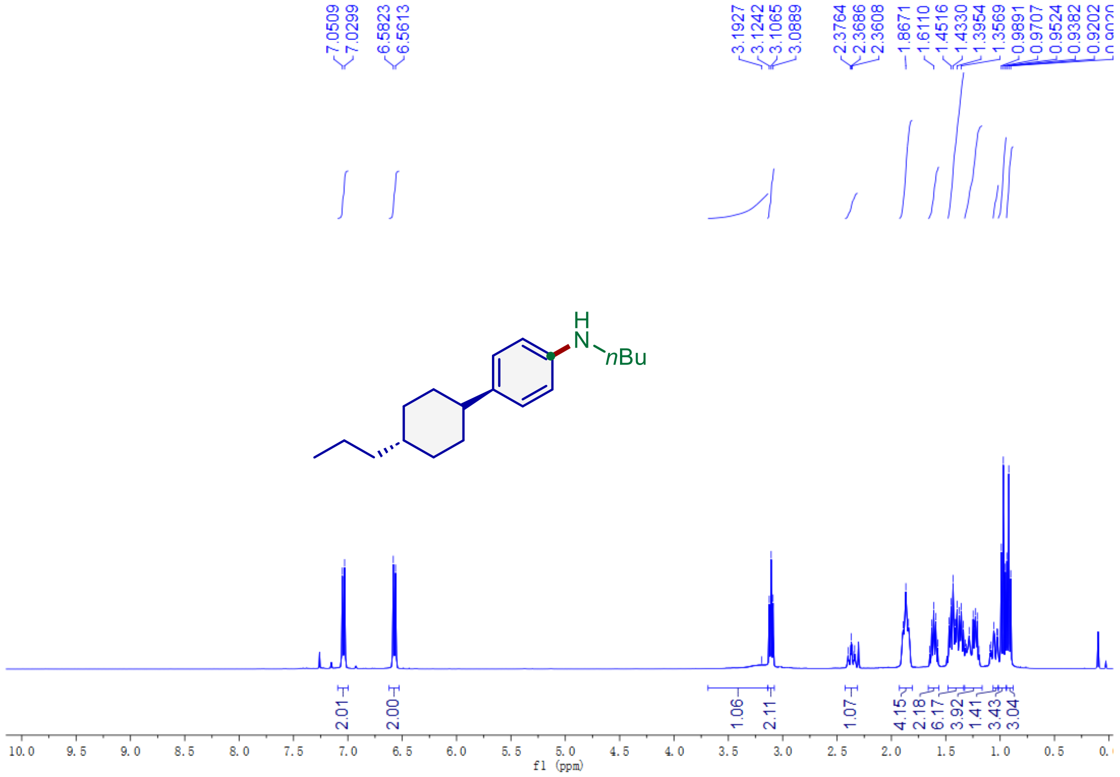

^{1}H NMR (400 MHz, $CDCl_3$) spectrum of compound 11

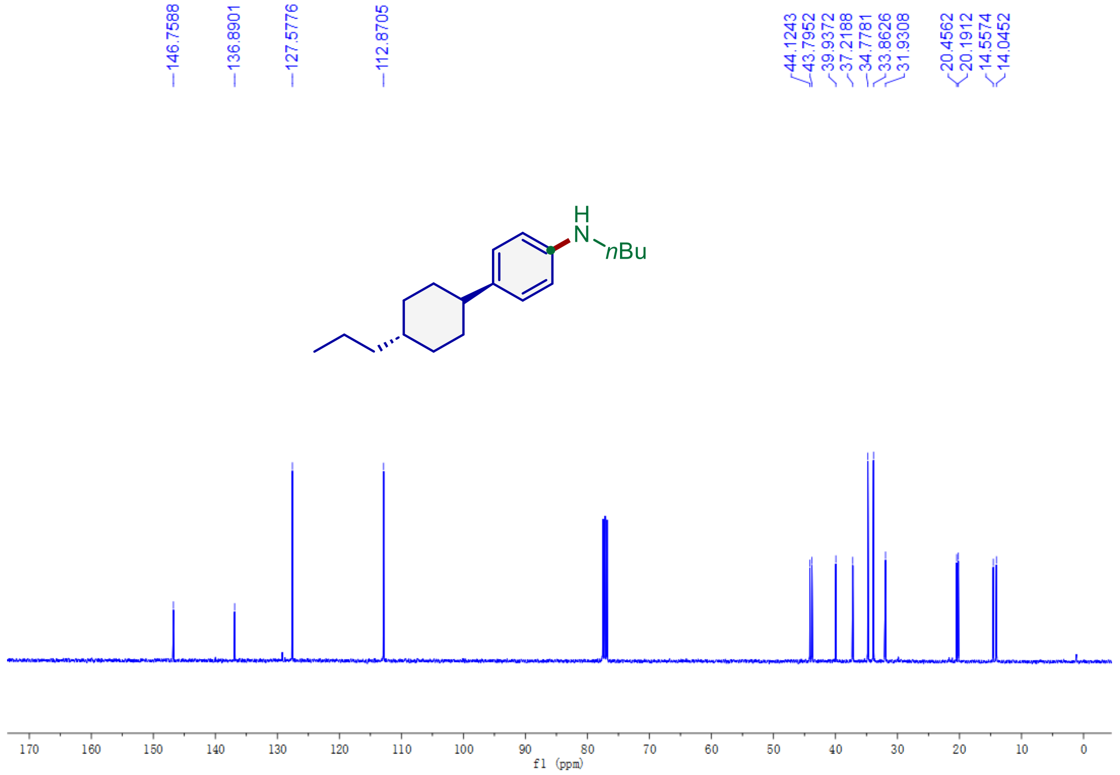

^{13}C NMR (100 MHz, $CDCl_3$) spectrum of compound 11

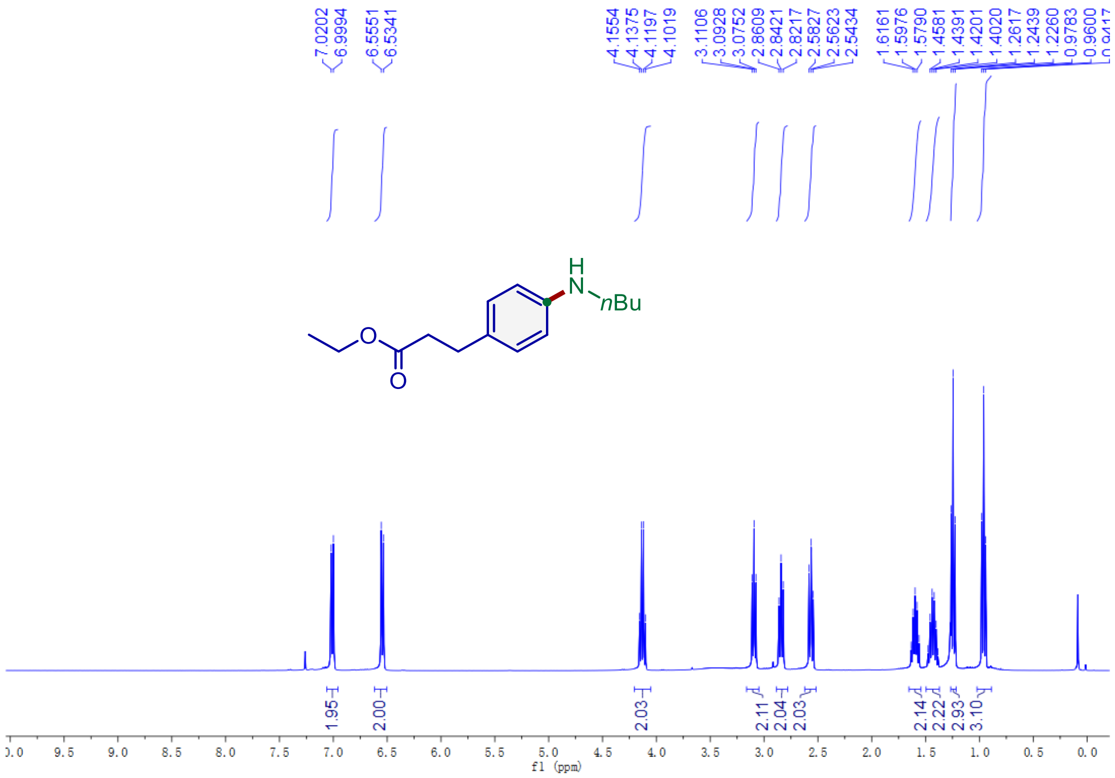

^{1}H NMR (400 MHz, $CDCl_3$) spectrum of compound 12

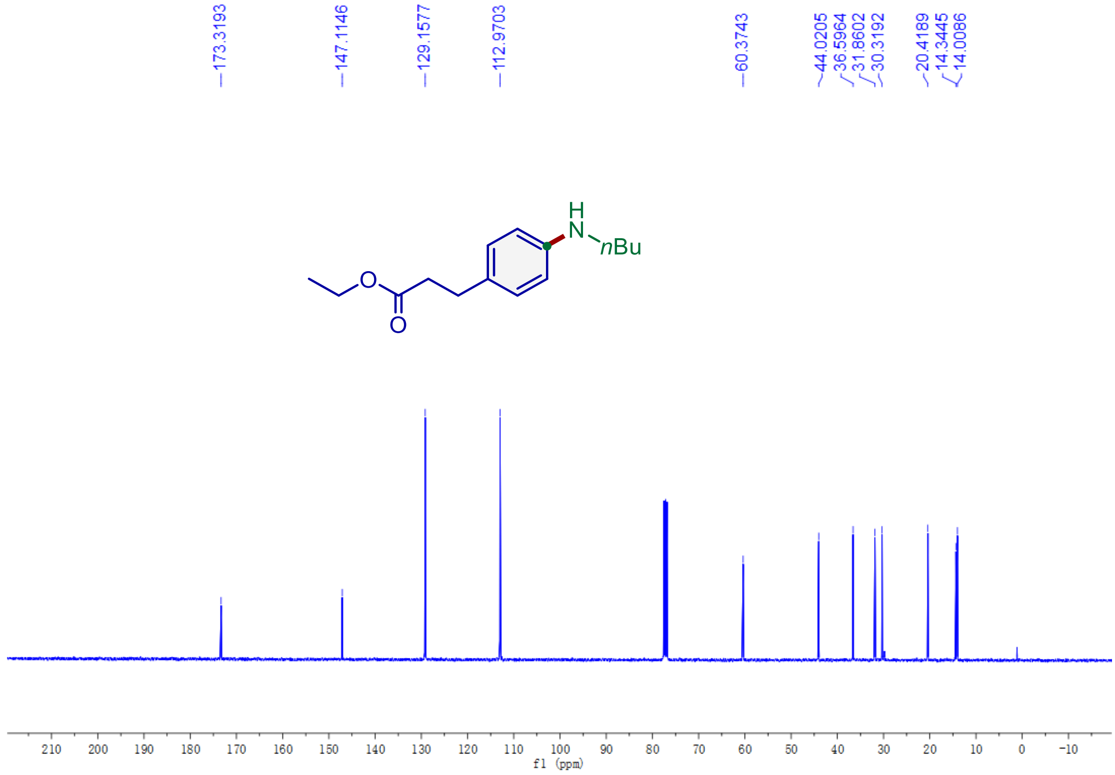

^{13}C NMR (100 MHz, $CDCl_3$) spectrum of compound 12

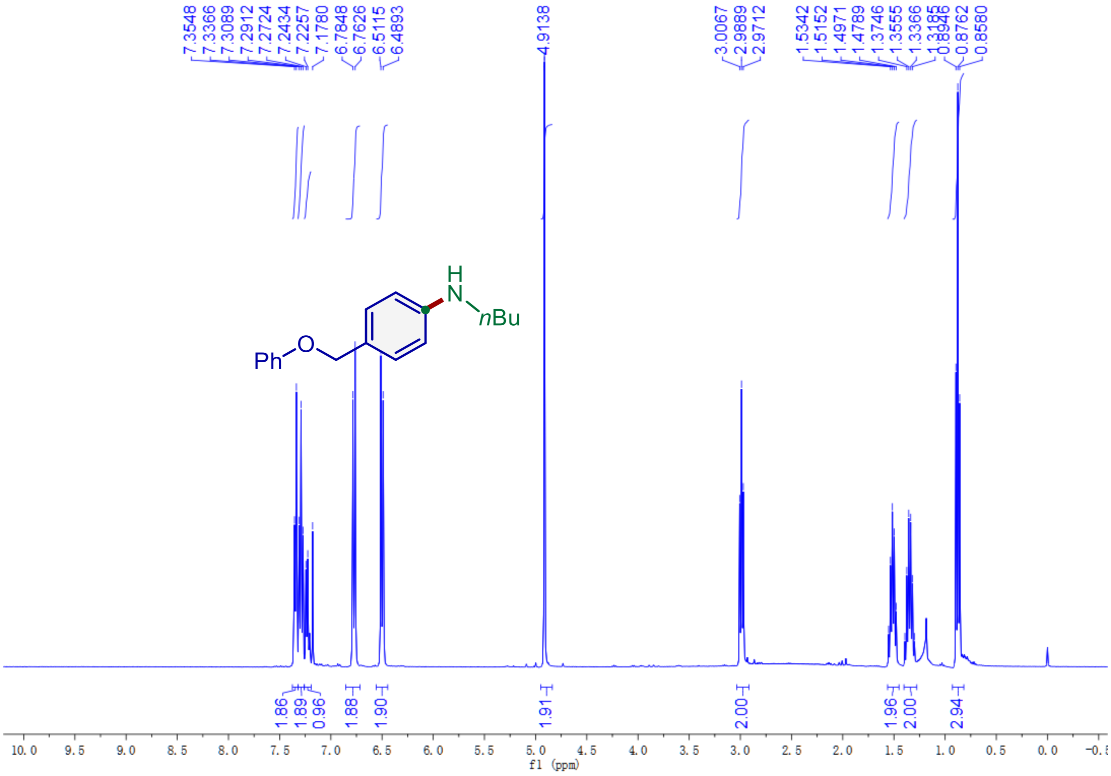

^{1}H NMR (400 MHz, $CDCl_3$) spectrum of compound 13

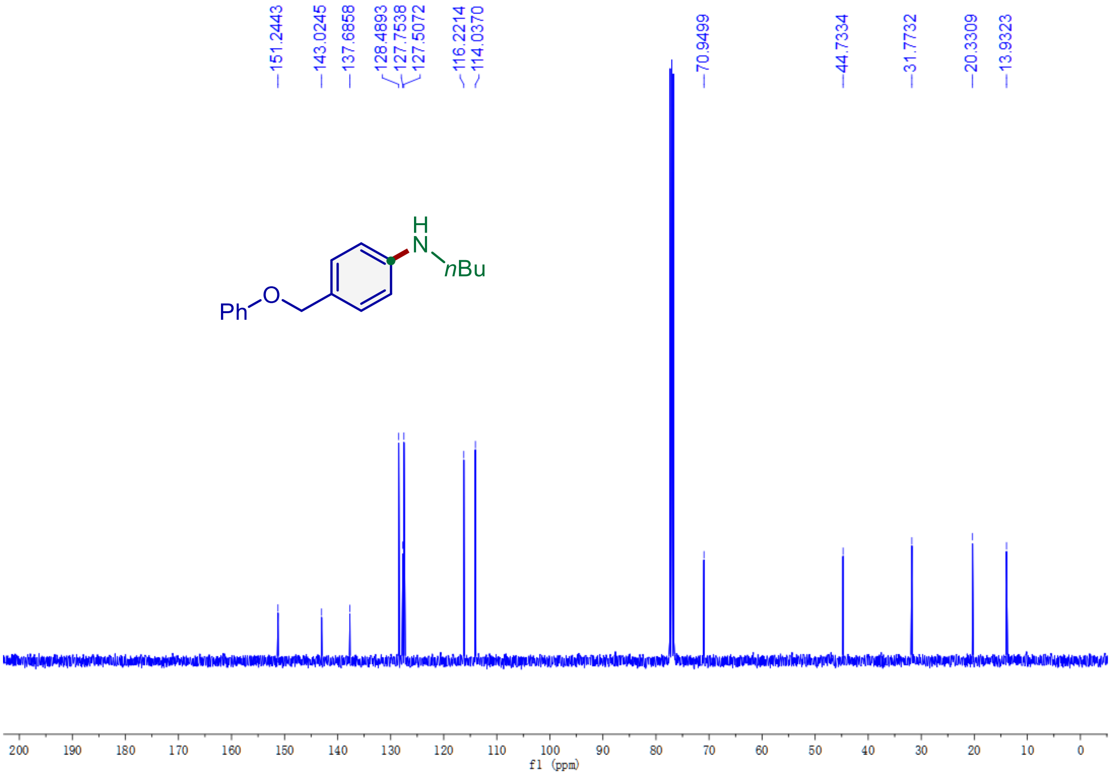

^{13}C NMR (100 MHz, $CDCl_3$) spectrum of compound 13

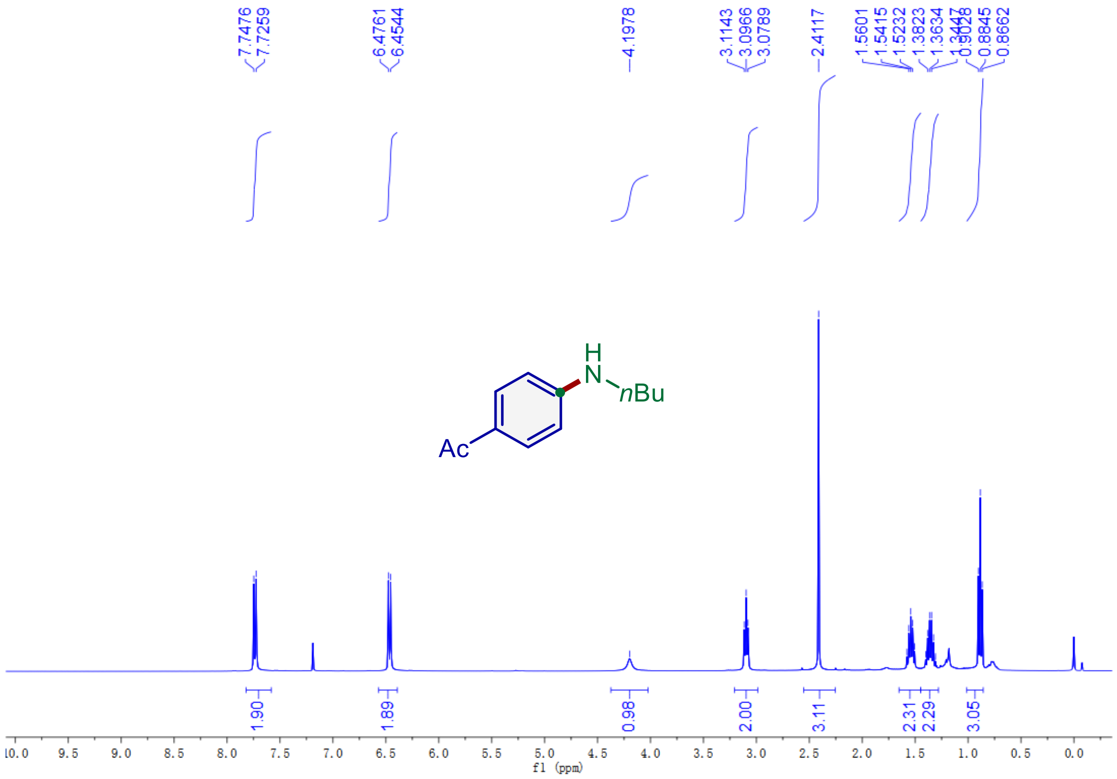

^{1}H NMR (400 MHz, $CDCl_3$) spectrum of compound 14

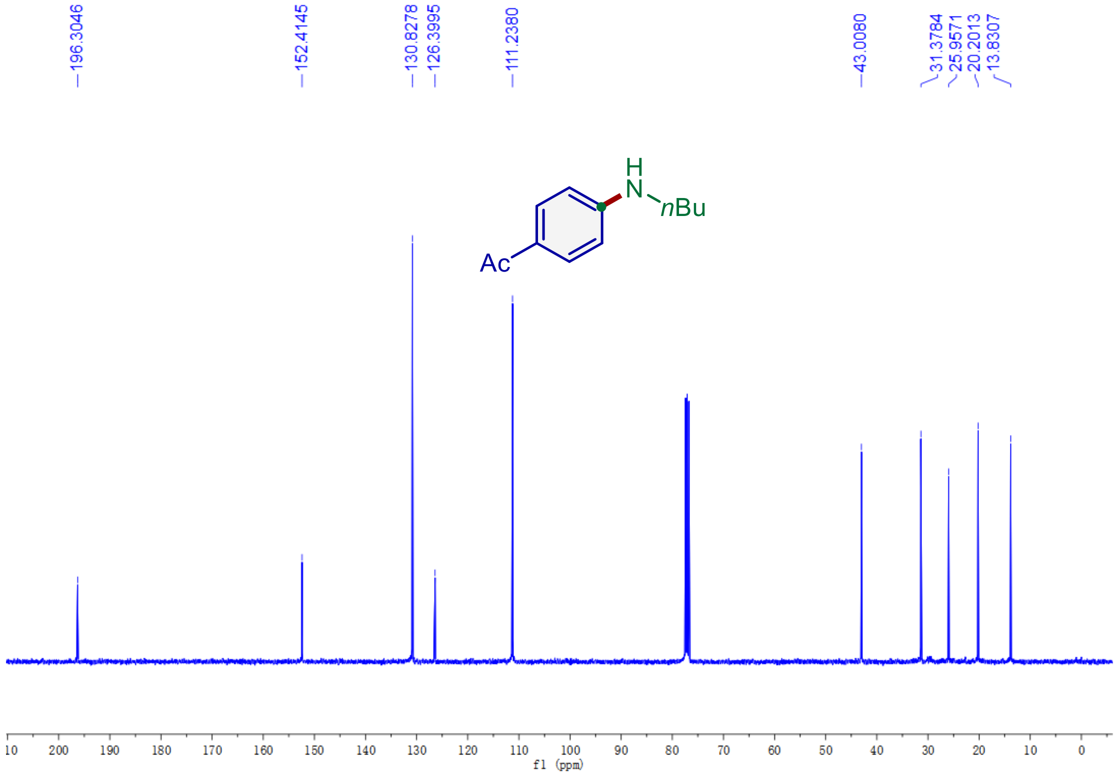

^{13}C NMR (100 MHz, $CDCl_3$) spectrum of compound 14

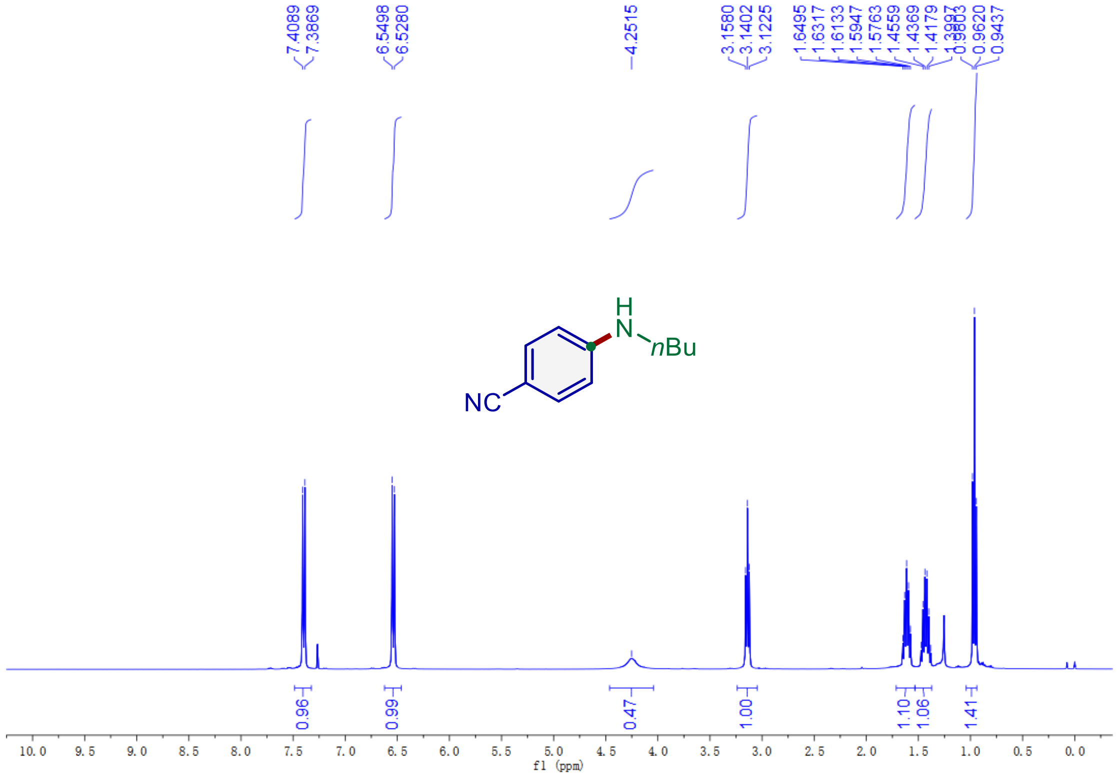

^{1}H NMR (400 MHz, $CDCl_3$) spectrum of compound 15

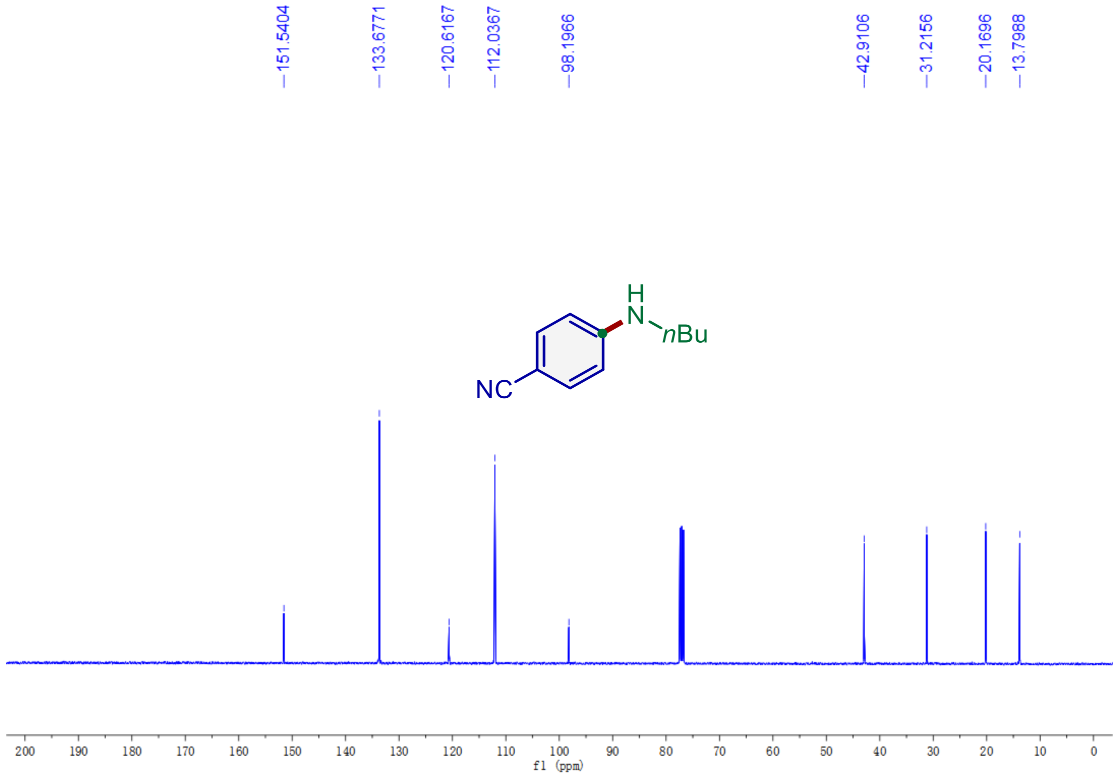

^{13}C NMR (100 MHz, $CDCl_3$) spectrum of compound 15

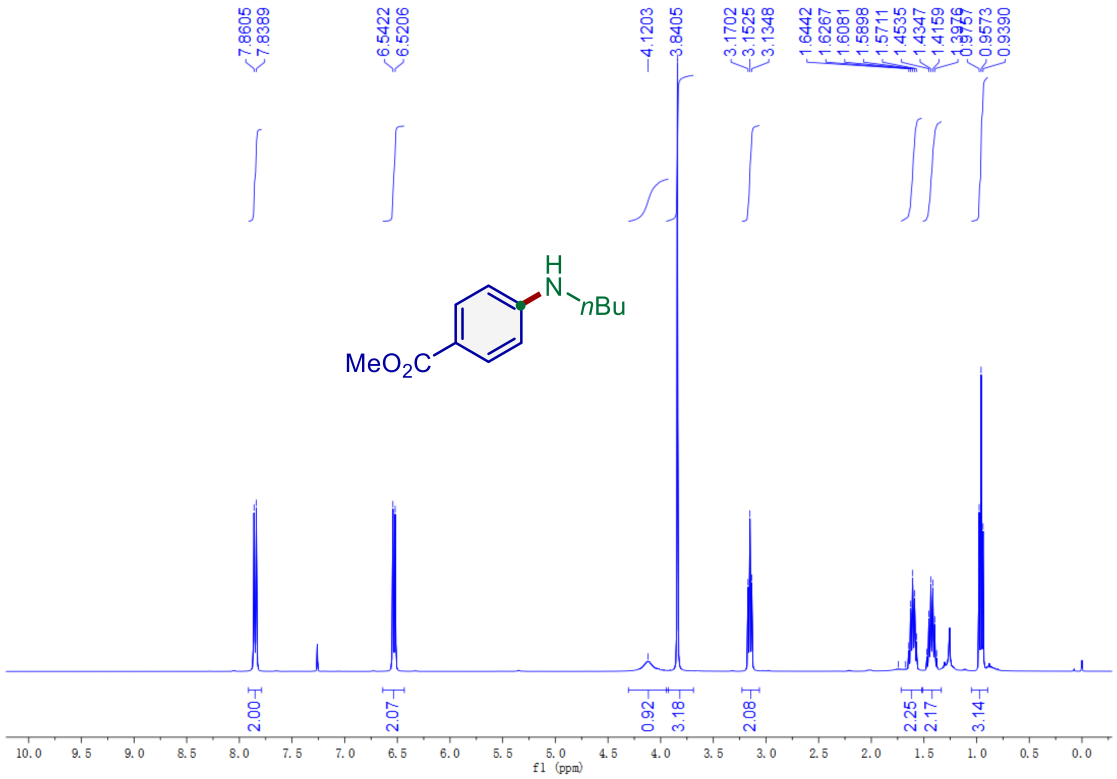

^{1}H NMR (400 MHz, $CDCl_3$) spectrum of compound 16

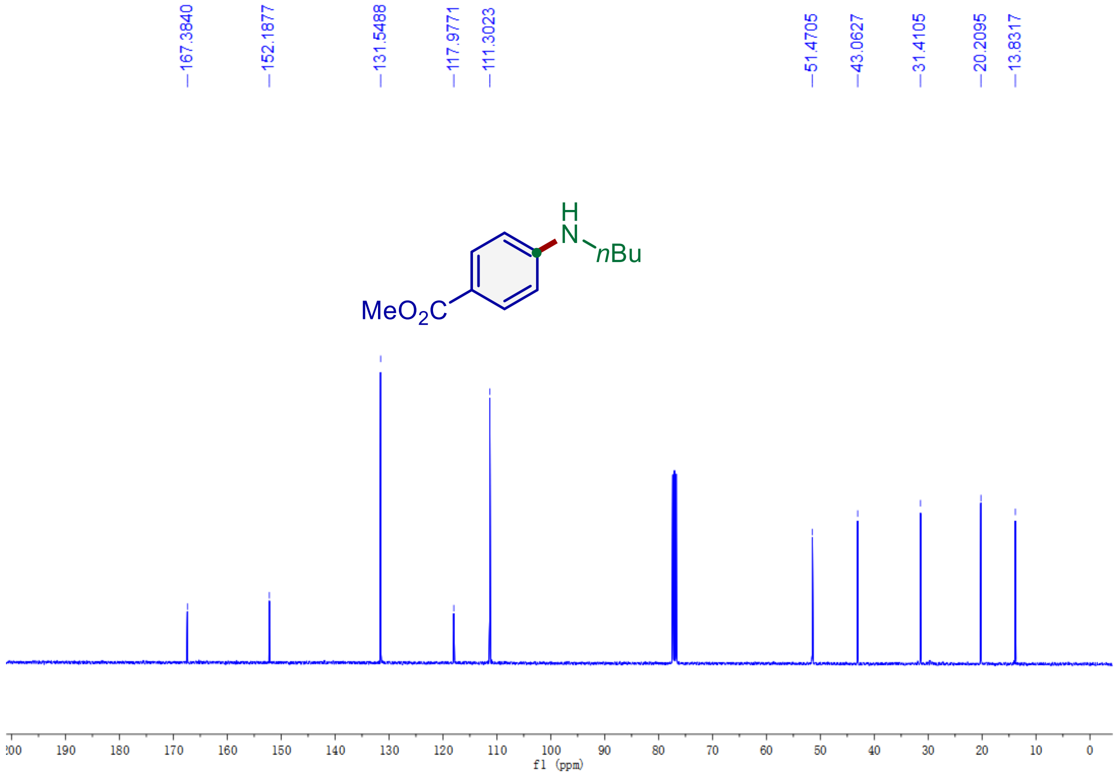

^{13}C NMR (100 MHz, $CDCl_3$) spectrum of compound 16

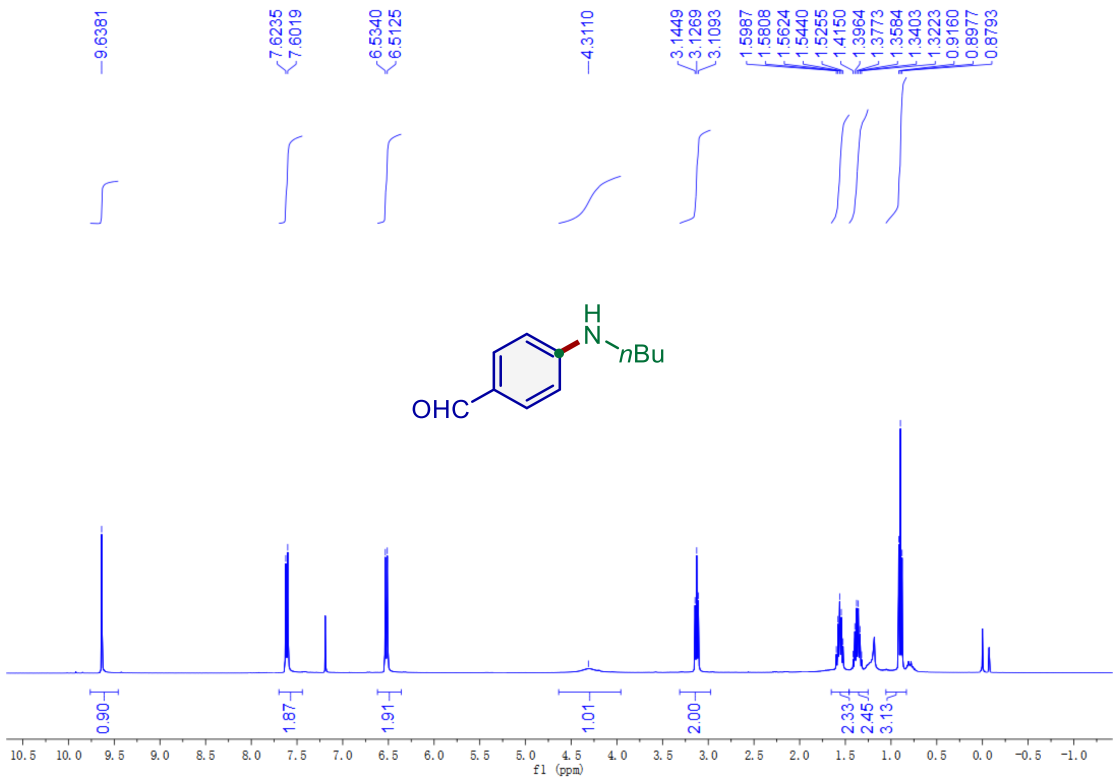

^{1}H NMR (400 MHz, $CDCl_3$) spectrum of compound 17

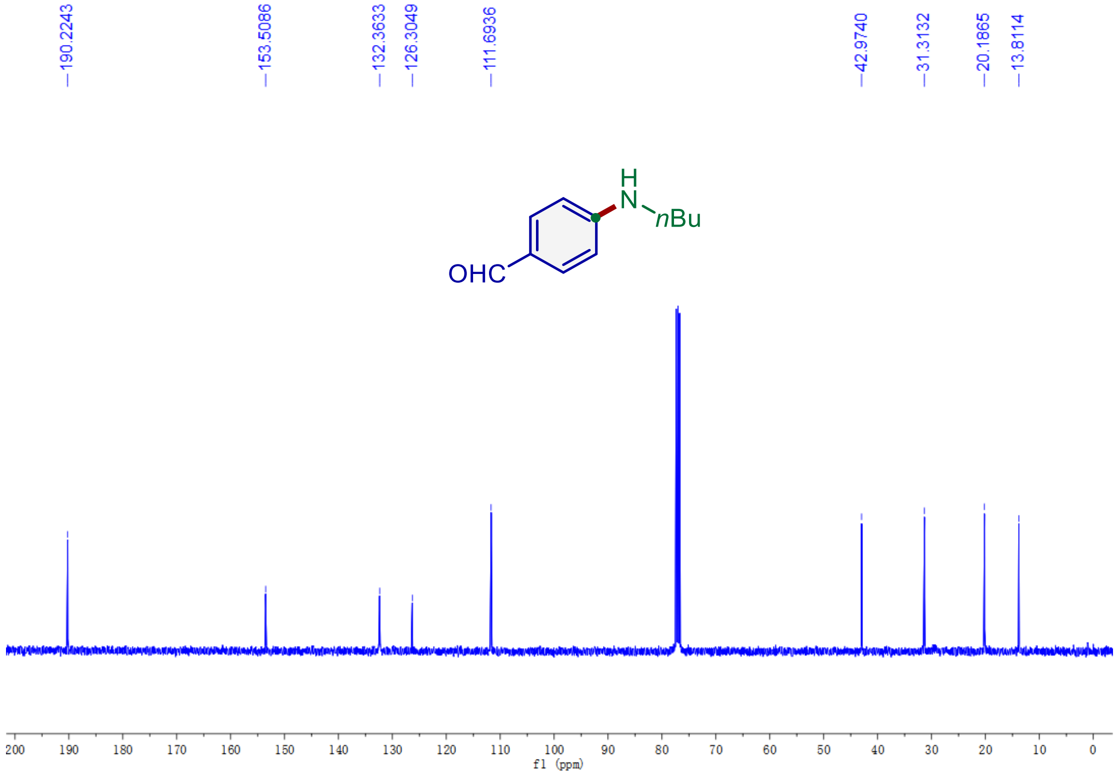

^{13}C NMR (100 MHz, $CDCl_3$) spectrum of compound 17

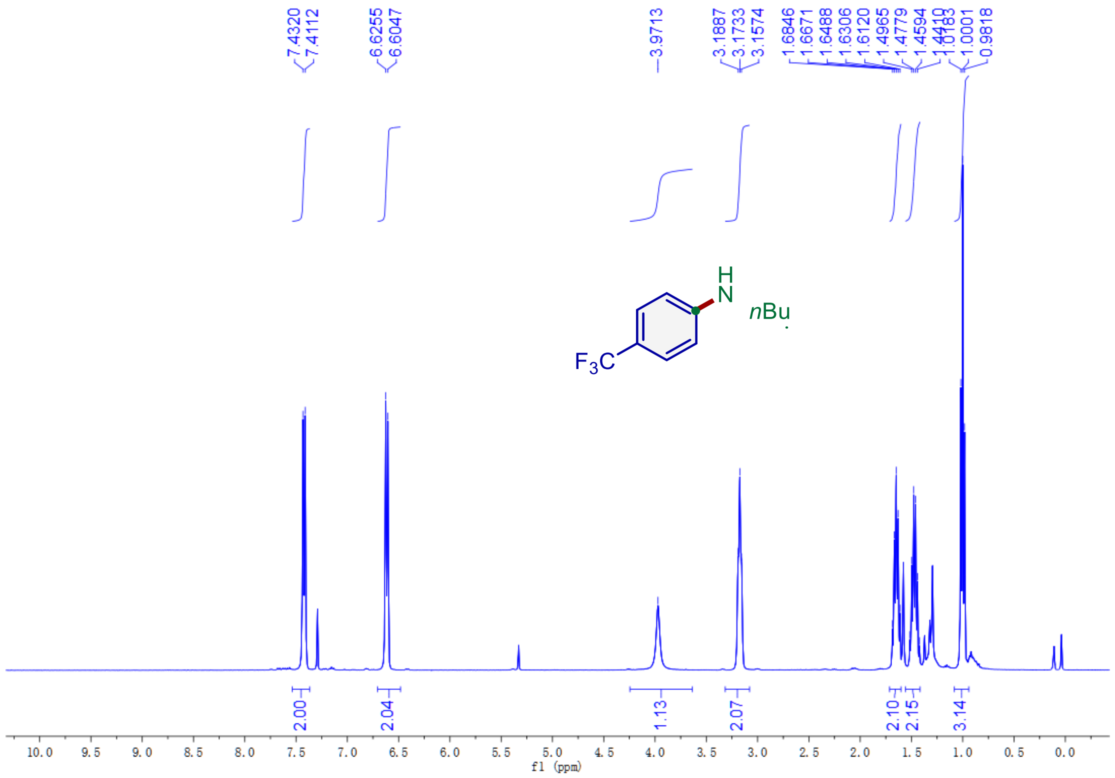

^{1}H NMR (400 MHz, $CDCl_3$) spectrum of compound 18

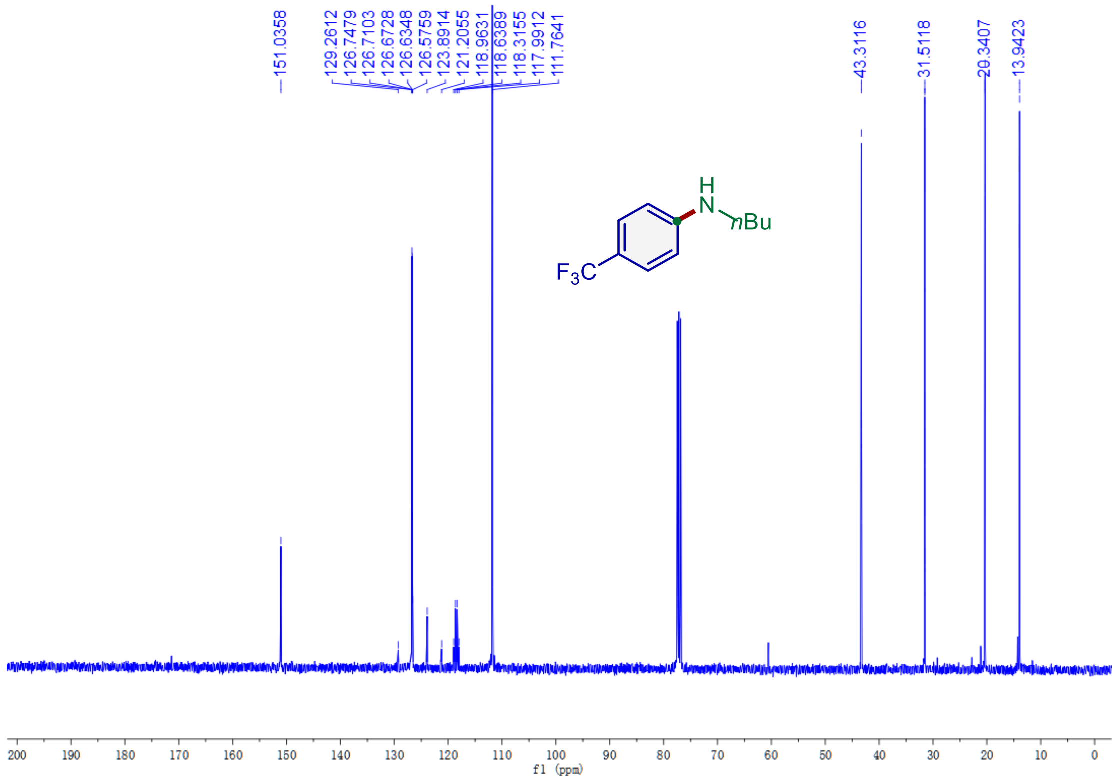

^{13}C NMR (100 MHz, $CDCl_3$) spectrum of compound 18

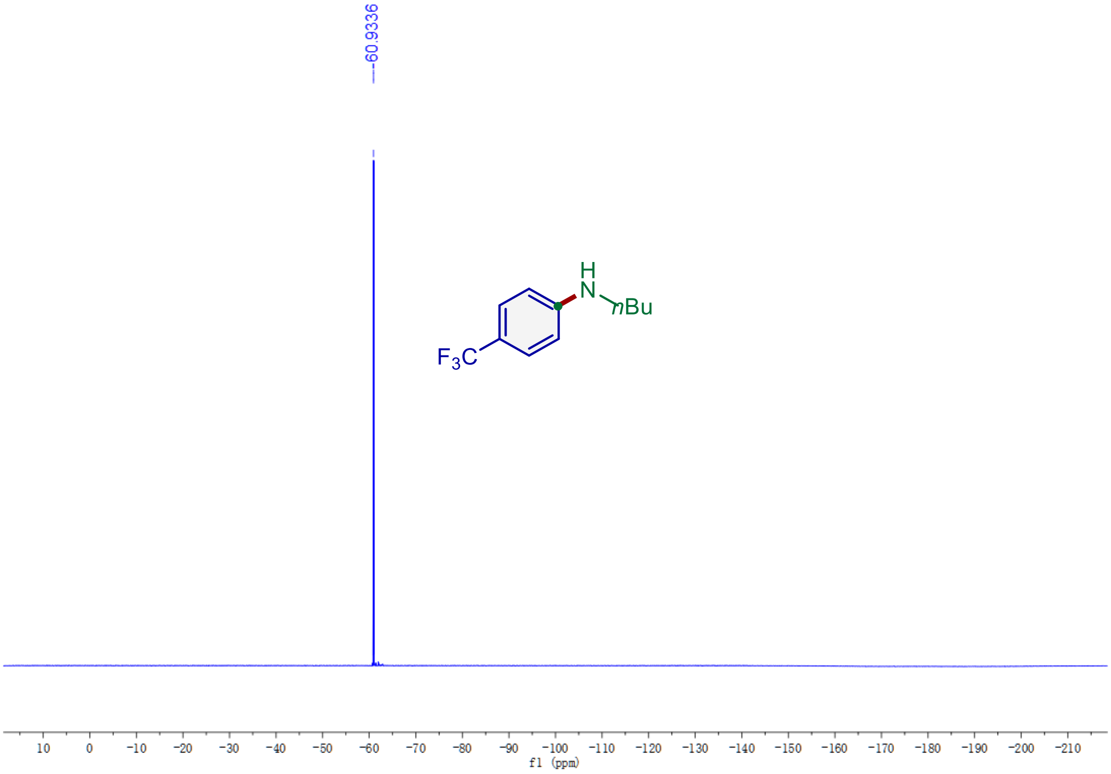

^{19}F NMR (376 MHz, $CDCl_3$) spectrum of compound 18

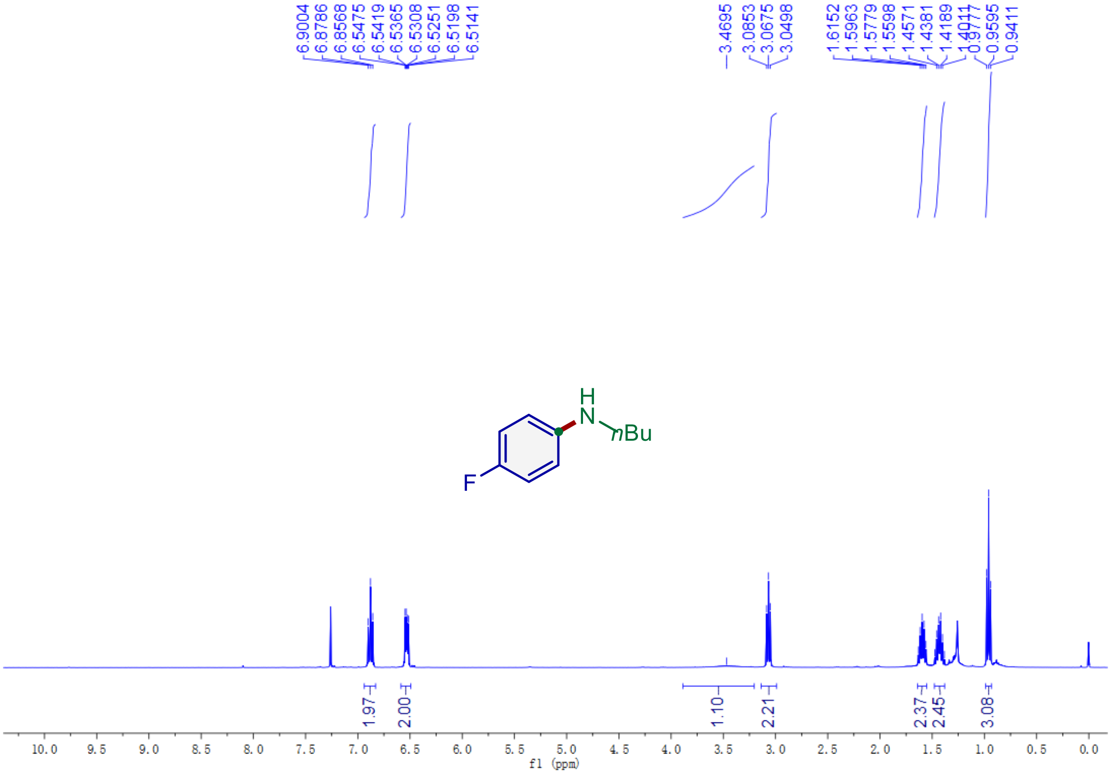

^{1}H NMR (400 MHz, $CDCl_3$) spectrum of compound 19

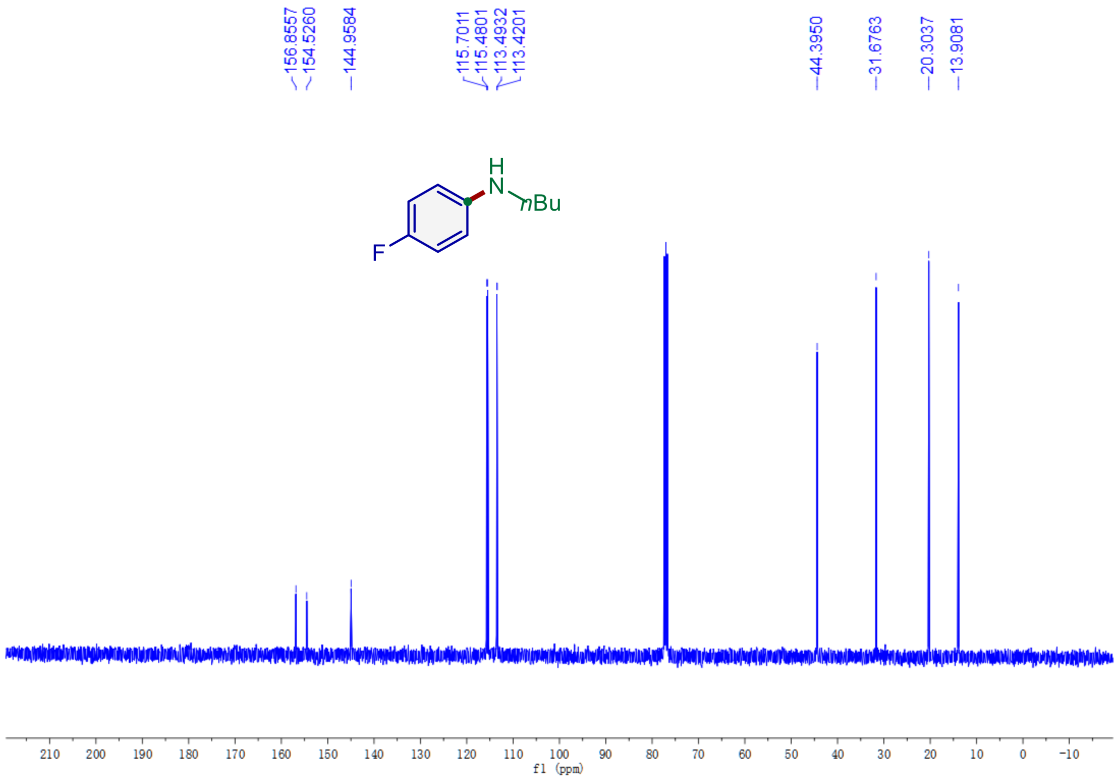

^{13}C NMR (100 MHz, $CDCl_3$) spectrum of compound 19

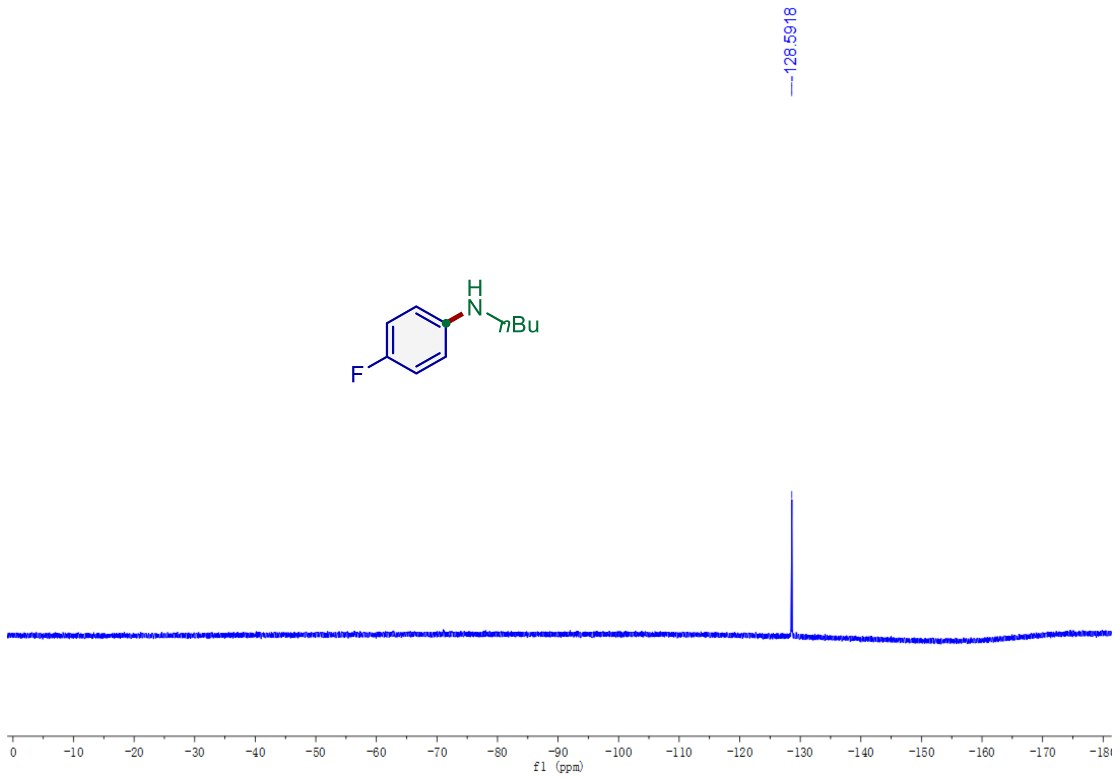

^{19}F NMR (376 MHz, $CDCl_3$) spectrum of compound 19

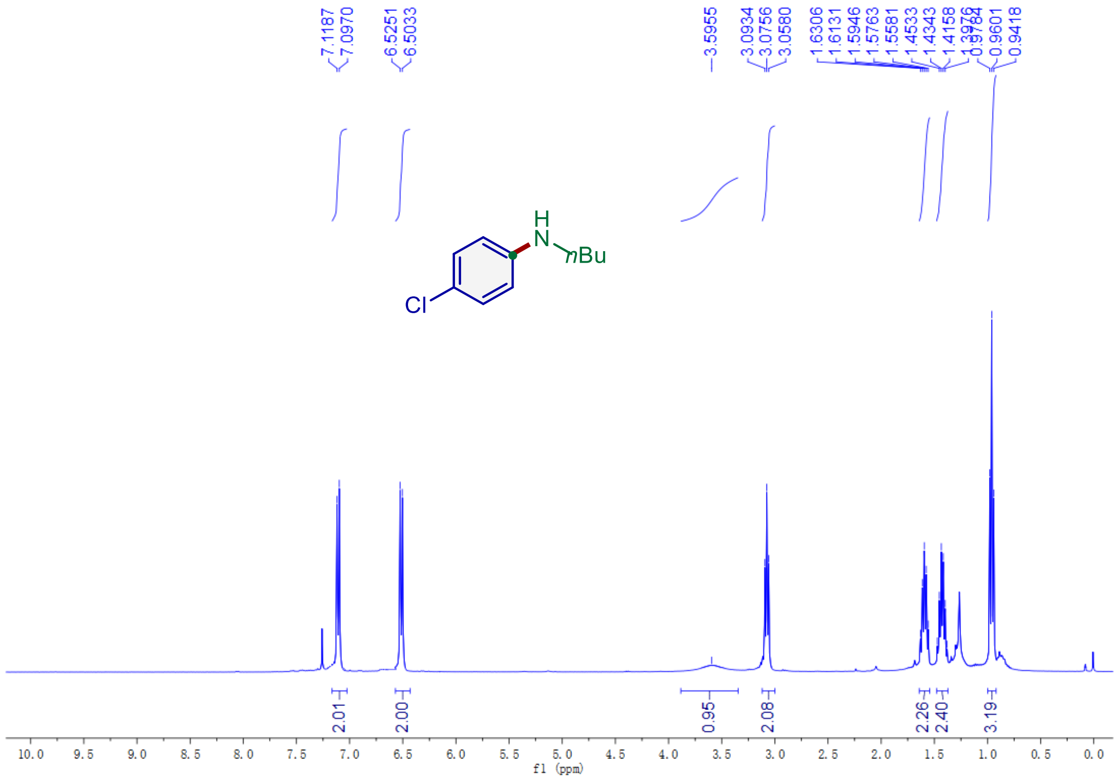

^{1}H NMR (400 MHz, $CDCl_3$) spectrum of compound 20

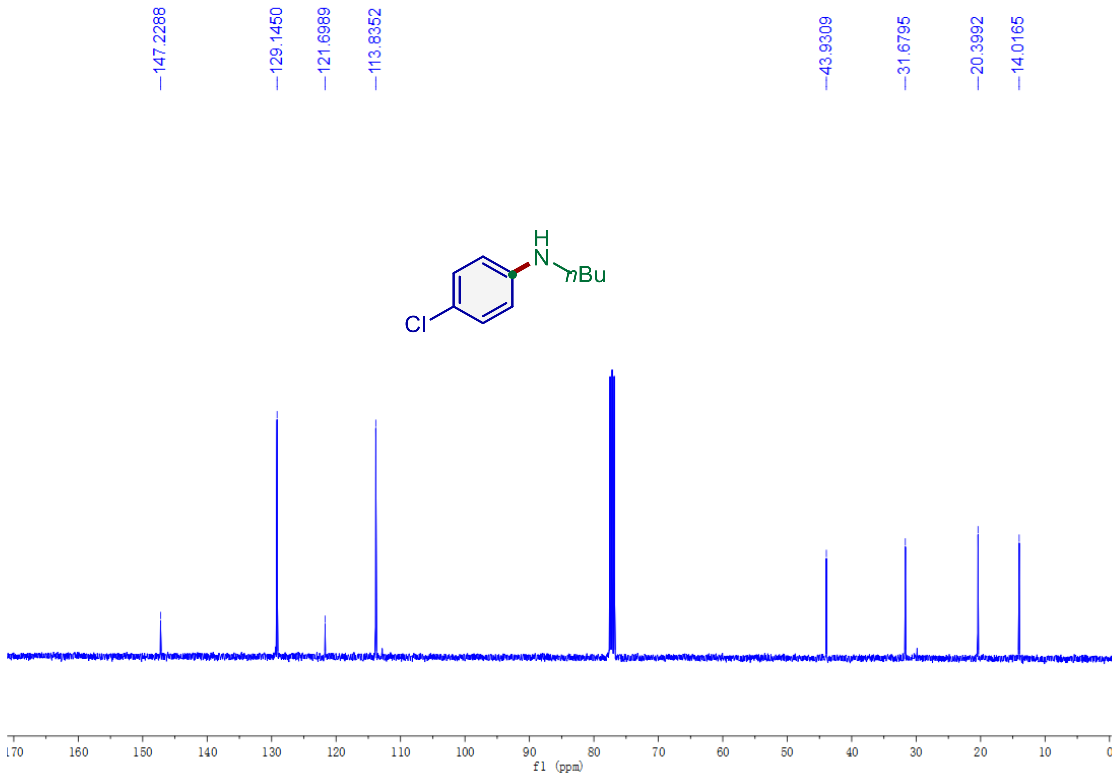

^{13}C NMR (100 MHz, $CDCl_3$) spectrum of compound 20

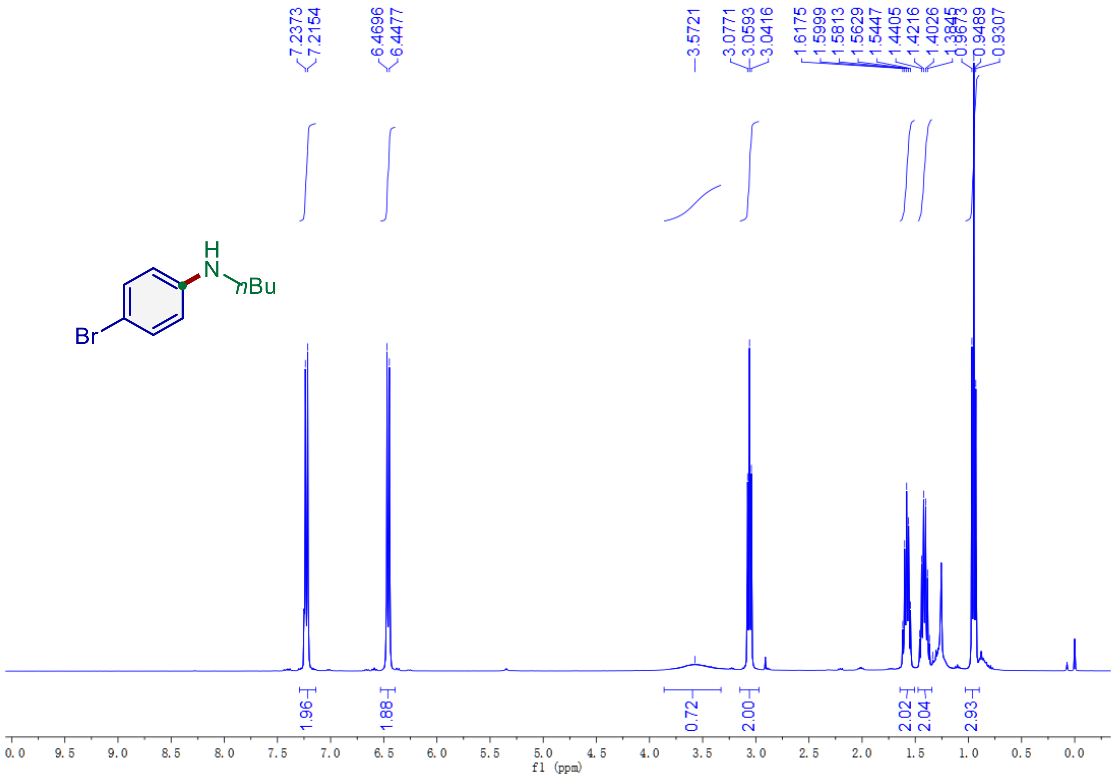

^{1}H NMR (400 MHz, $CDCl_3$) spectrum of compound 21

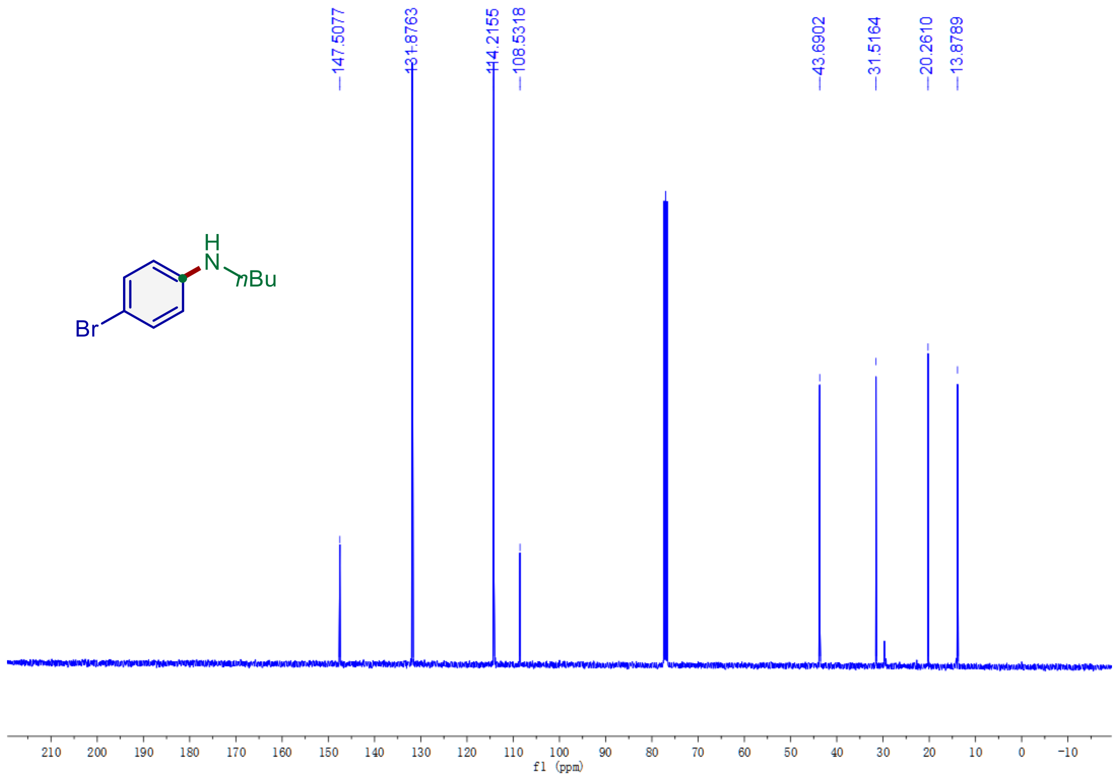

^{13}C NMR (100 MHz, $CDCl_3$) spectrum of compound 21

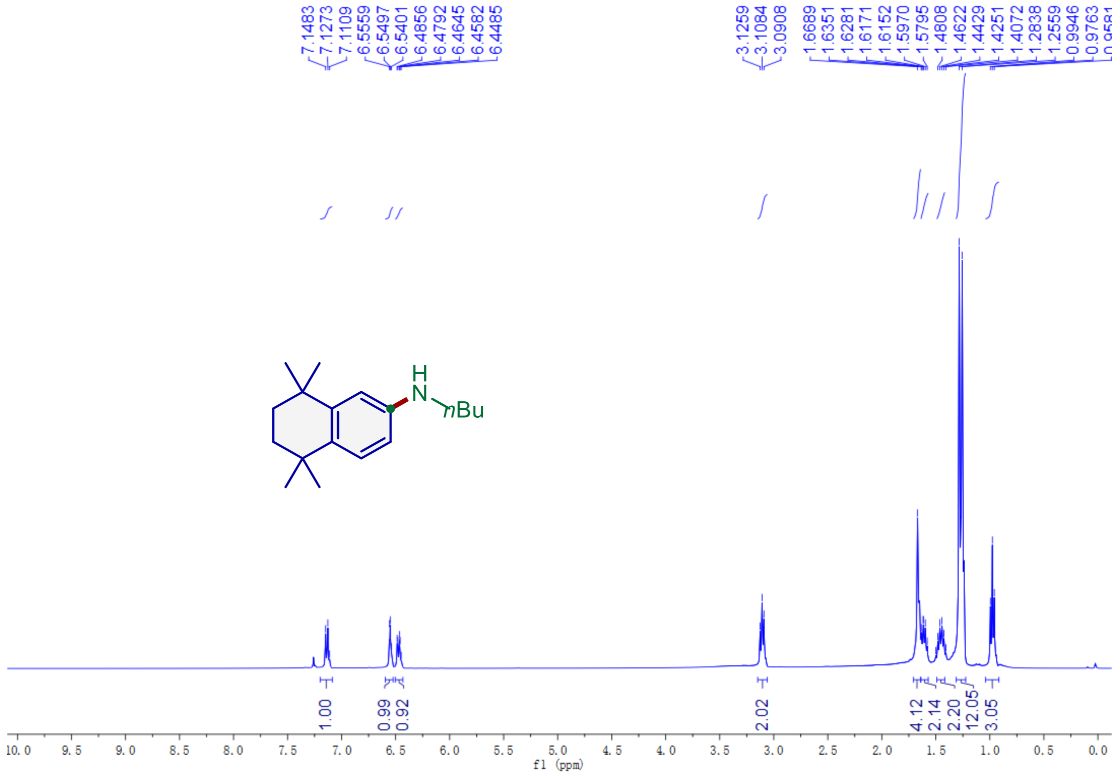

^{1}H NMR (400 MHz, $CDCl_3$) spectrum of compound 22

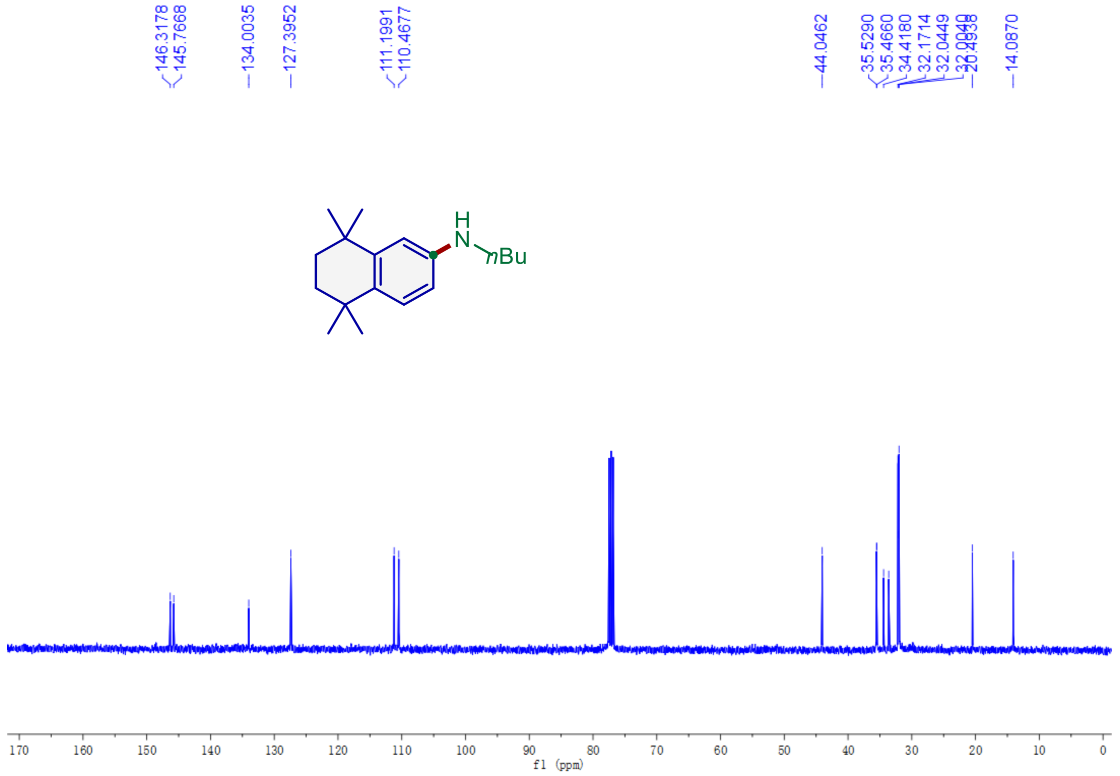

^{13}C NMR (100 MHz, $CDCl_3$) spectrum of compound 22

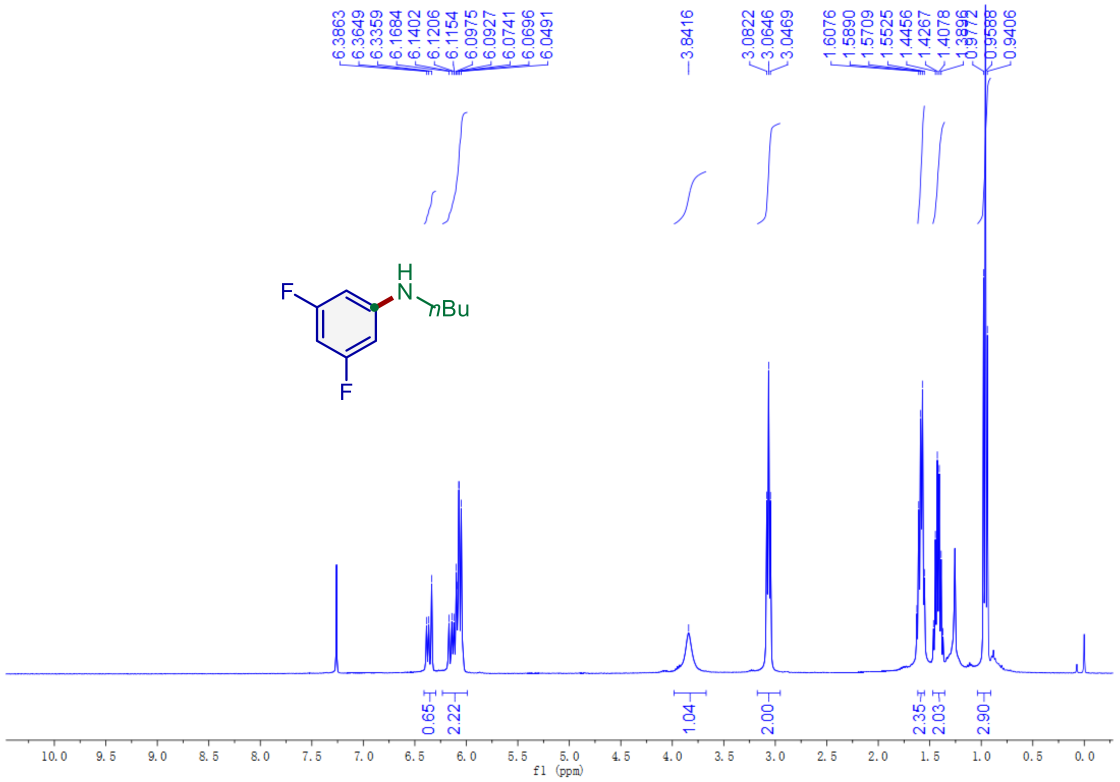

^{1}H NMR (400 MHz, $CDCl_3$) spectrum of compound 23

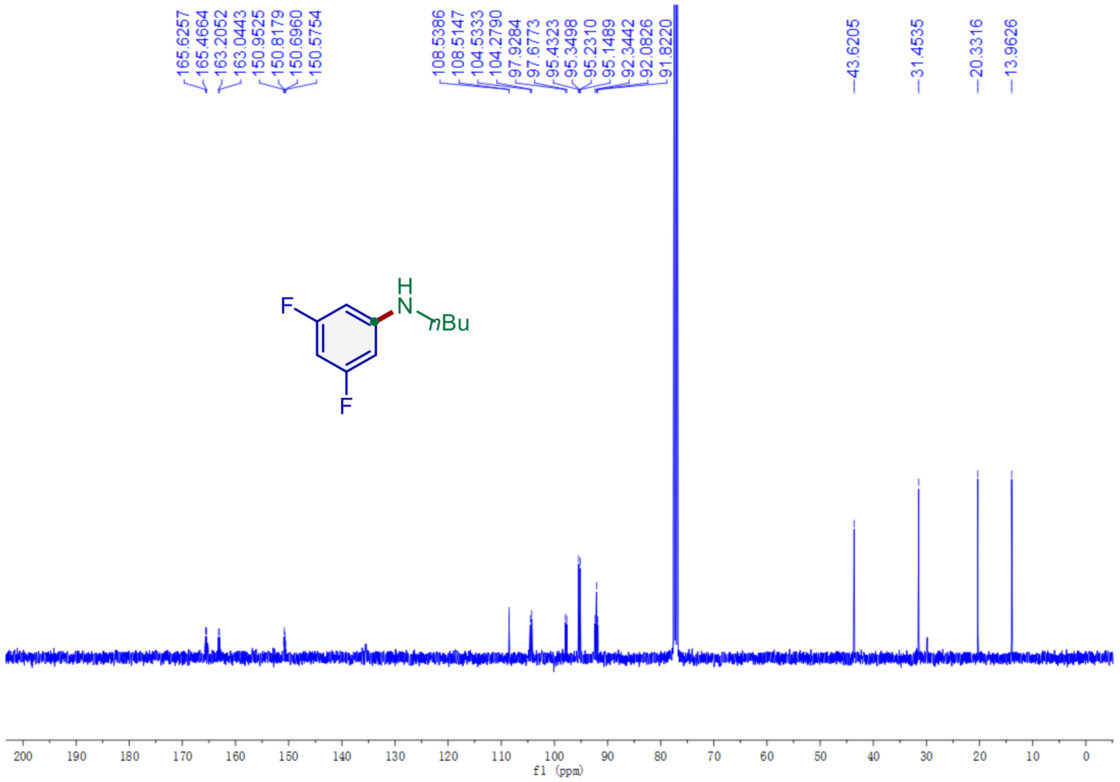

^{13}C NMR (100 MHz, $CDCl_3$) spectrum of compound 23

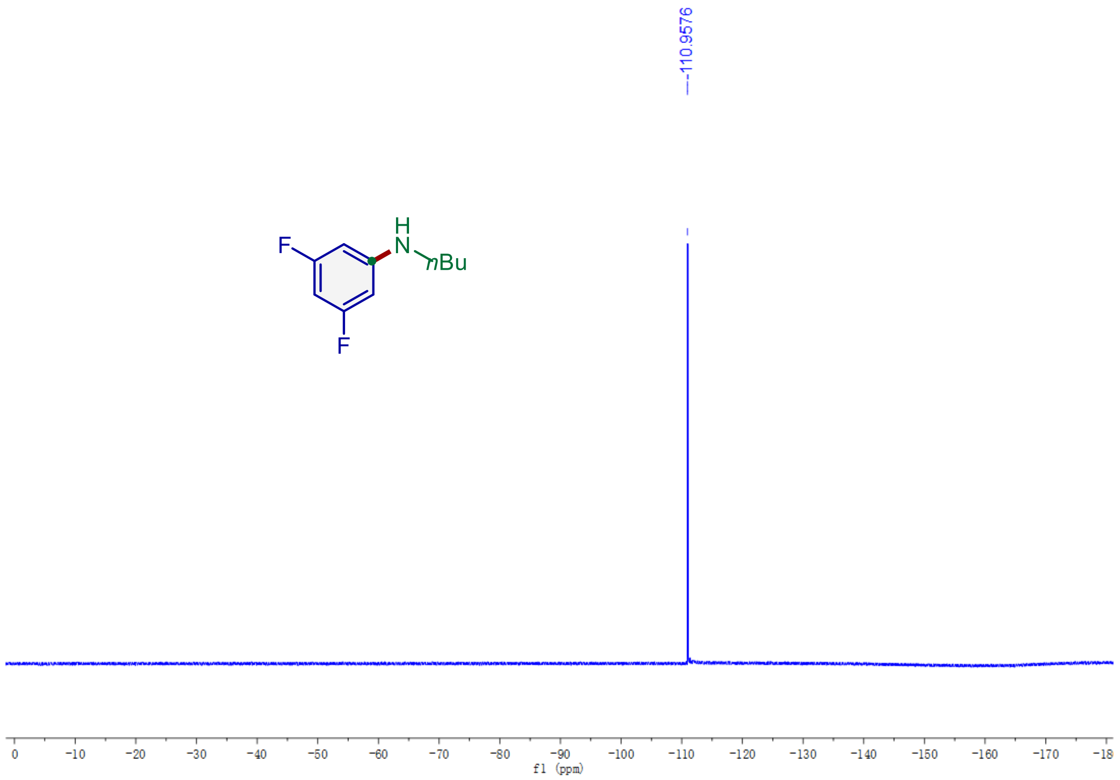

^{19}F NMR (376 MHz, $CDCl_3$) spectrum of compound 23

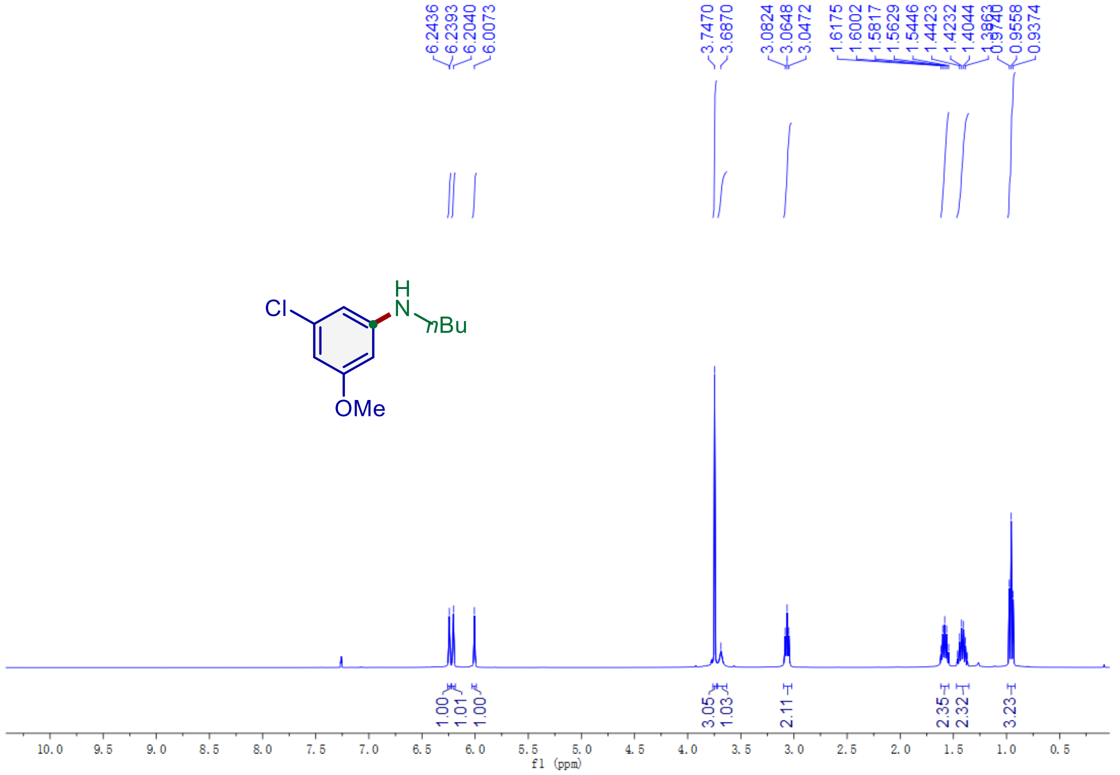

^{1}H NMR (400 MHz, $CDCl_3$) spectrum of compound 24

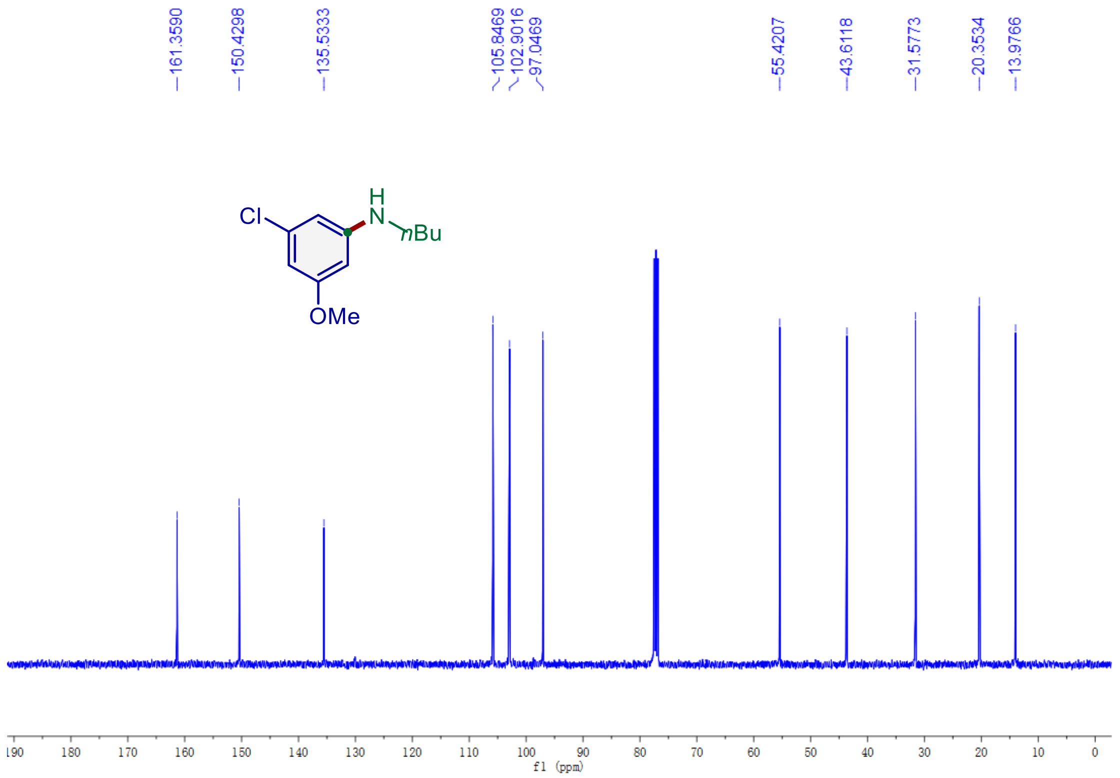

^{13}C NMR (100 MHz, $CDCl_3$) spectrum of compound 24

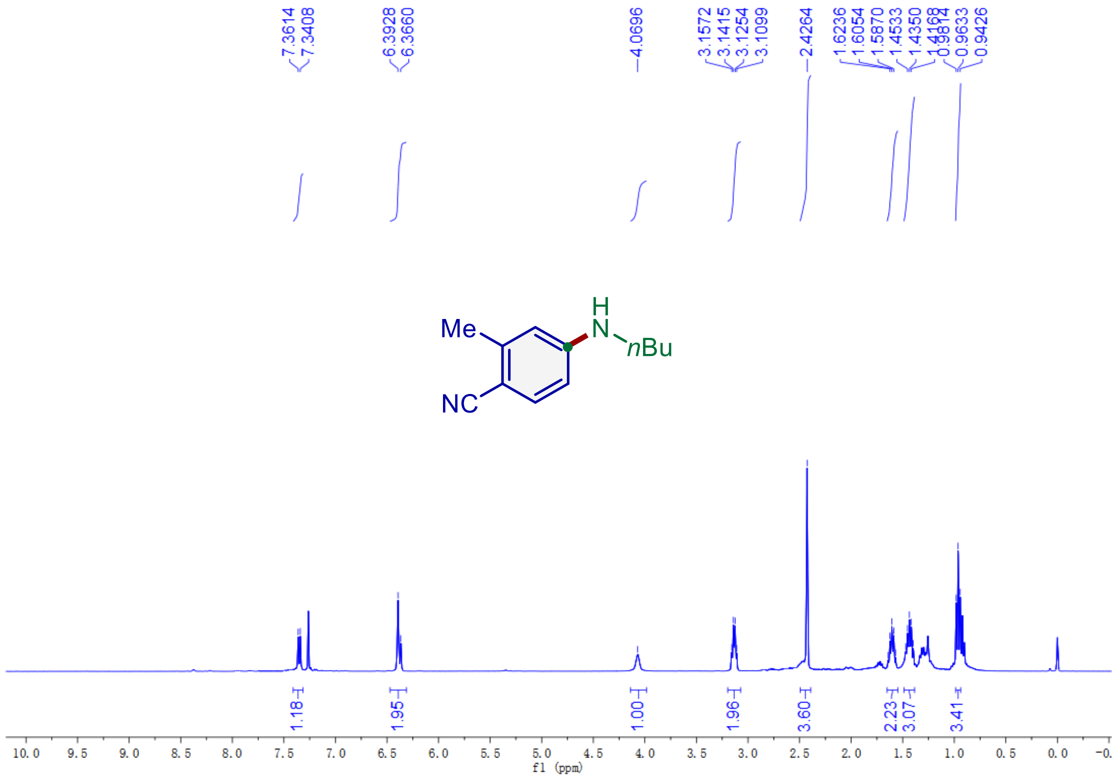

^{1}H NMR (400 MHz, $CDCl_3$) spectrum of compound 25

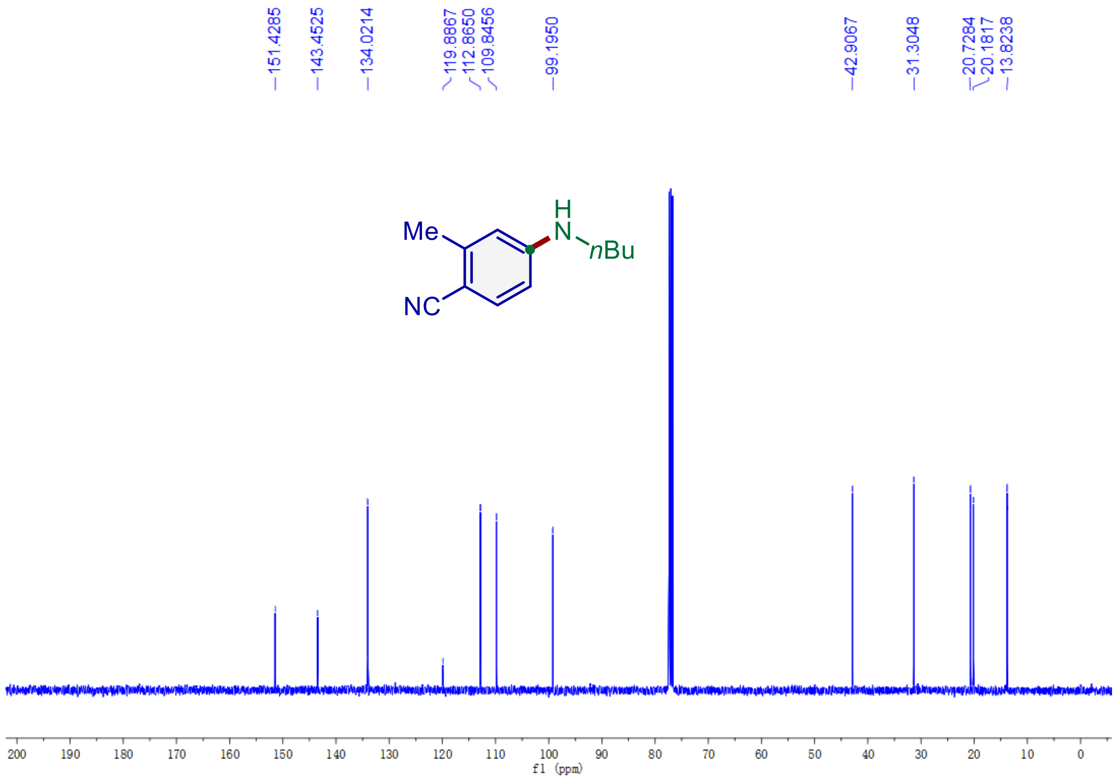

^{13}C NMR (100 MHz, $CDCl_3$) spectrum of compound 25

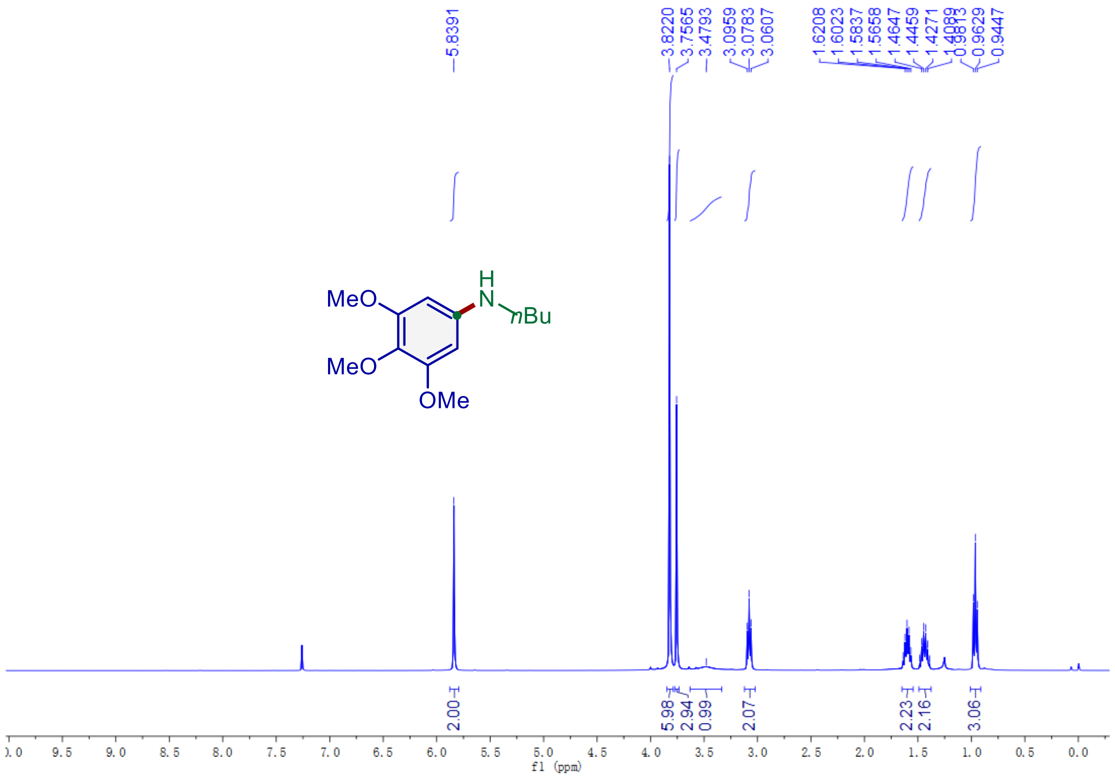

^{1}H NMR (400 MHz, $CDCl_3$) spectrum of compound 26

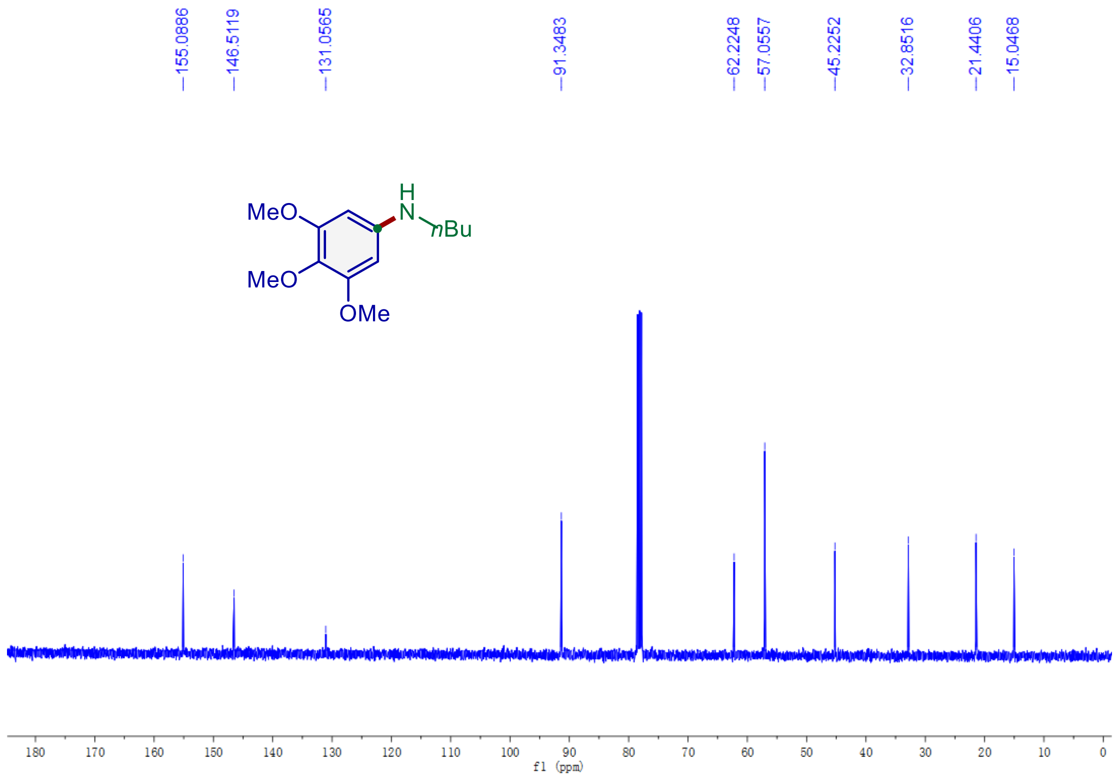

^{13}C NMR (100 MHz, $CDCl_3$) spectrum of compound 26

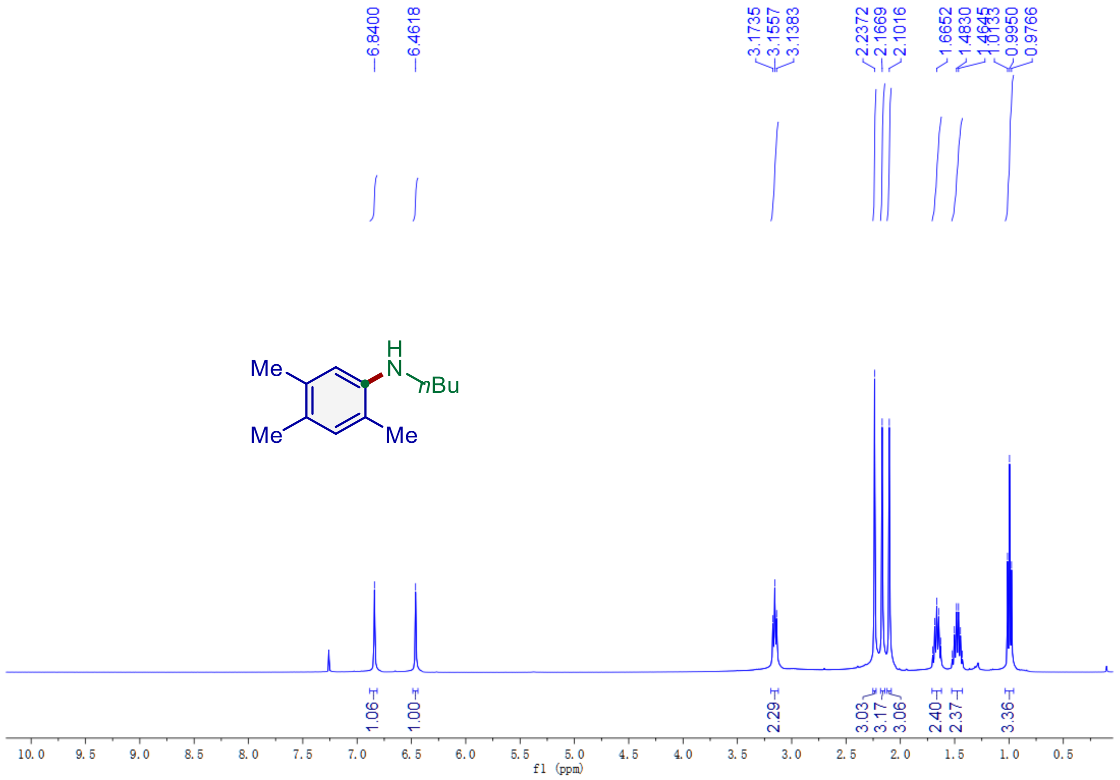

^{1}H NMR (400 MHz, $CDCl_3$) spectrum of compound 27

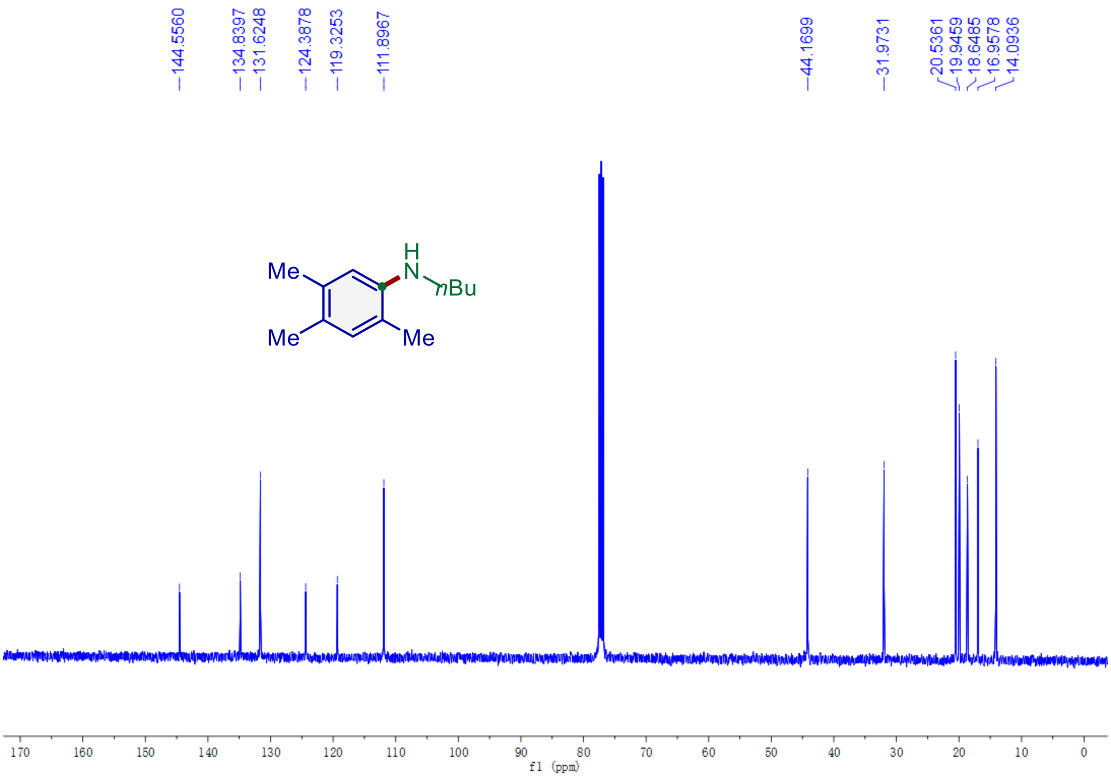

^{13}C NMR (100 MHz, $CDCl_3$) spectrum of compound 27

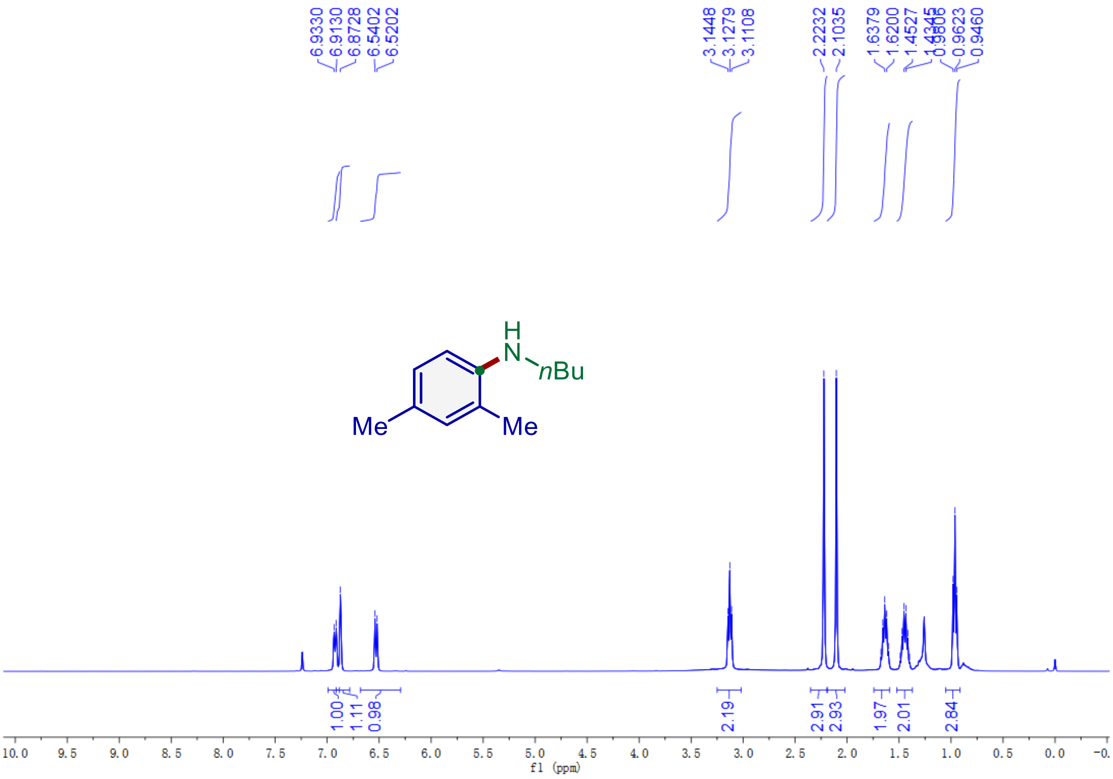

^{1}H NMR (400 MHz, $CDCl_3$) spectrum of compound 28

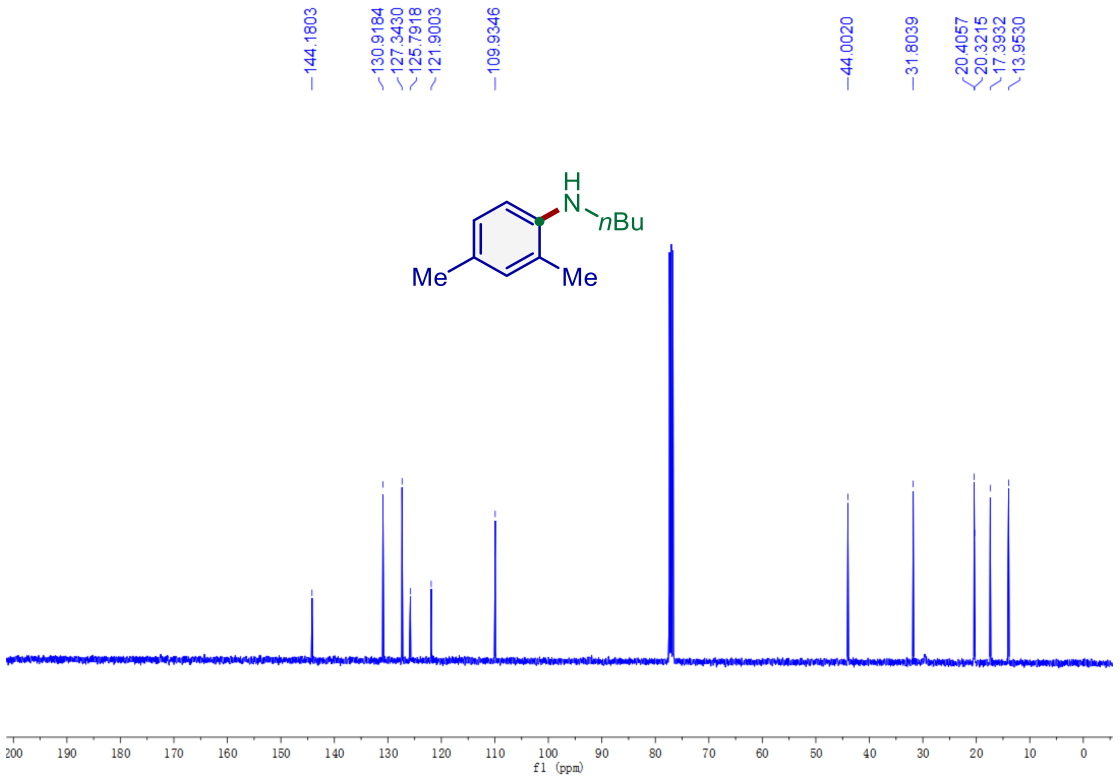

^{13}C NMR (100 MHz, $CDCl_3$) spectrum of compound 28

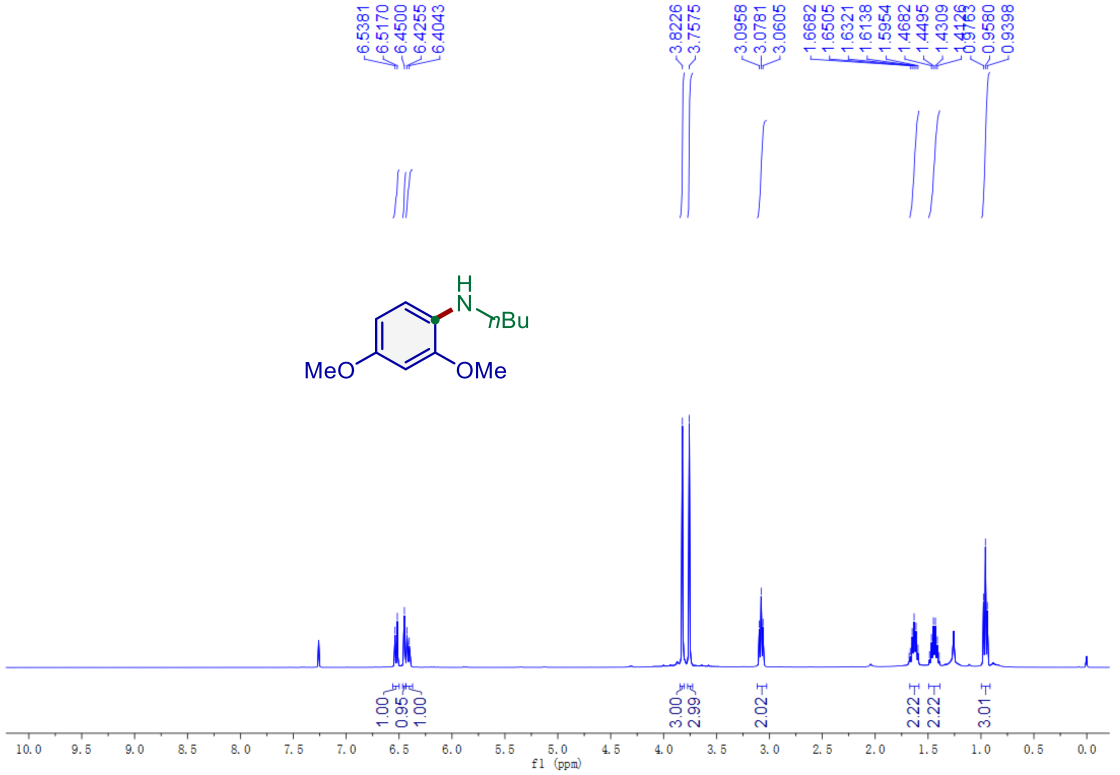

^{1}H NMR (400 MHz, $CDCl_3$) spectrum of compound 29

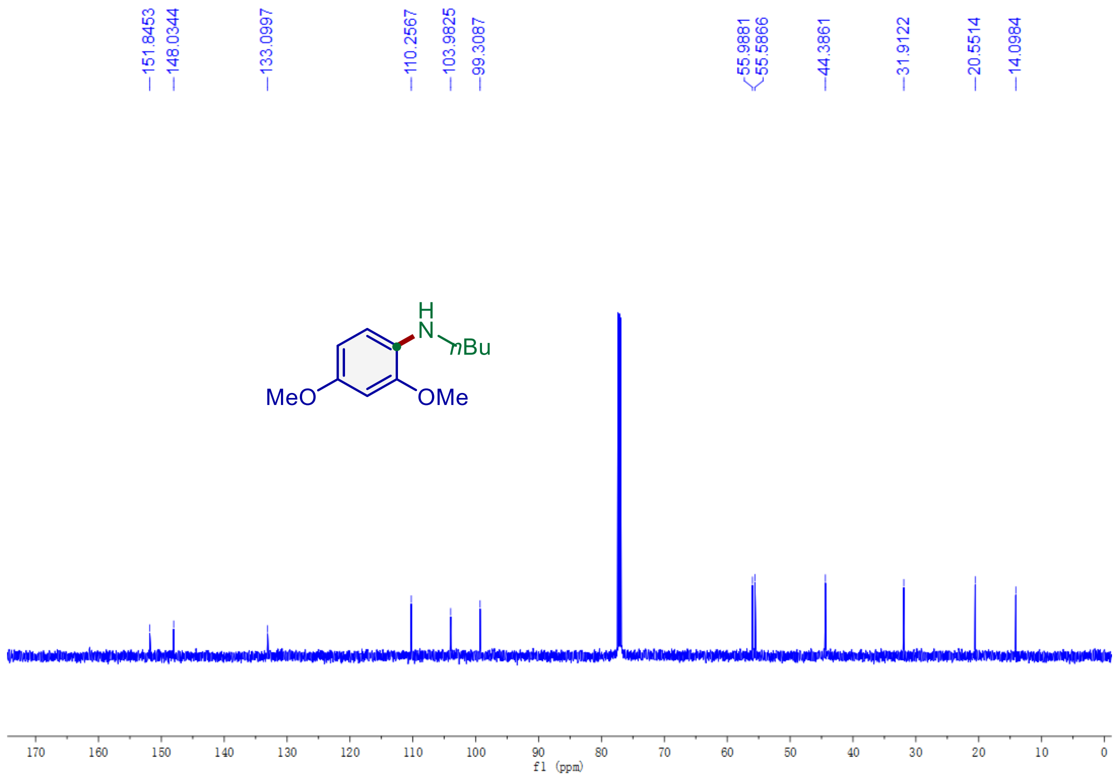

^{13}C NMR (100 MHz, $CDCl_3$) spectrum of compound 29

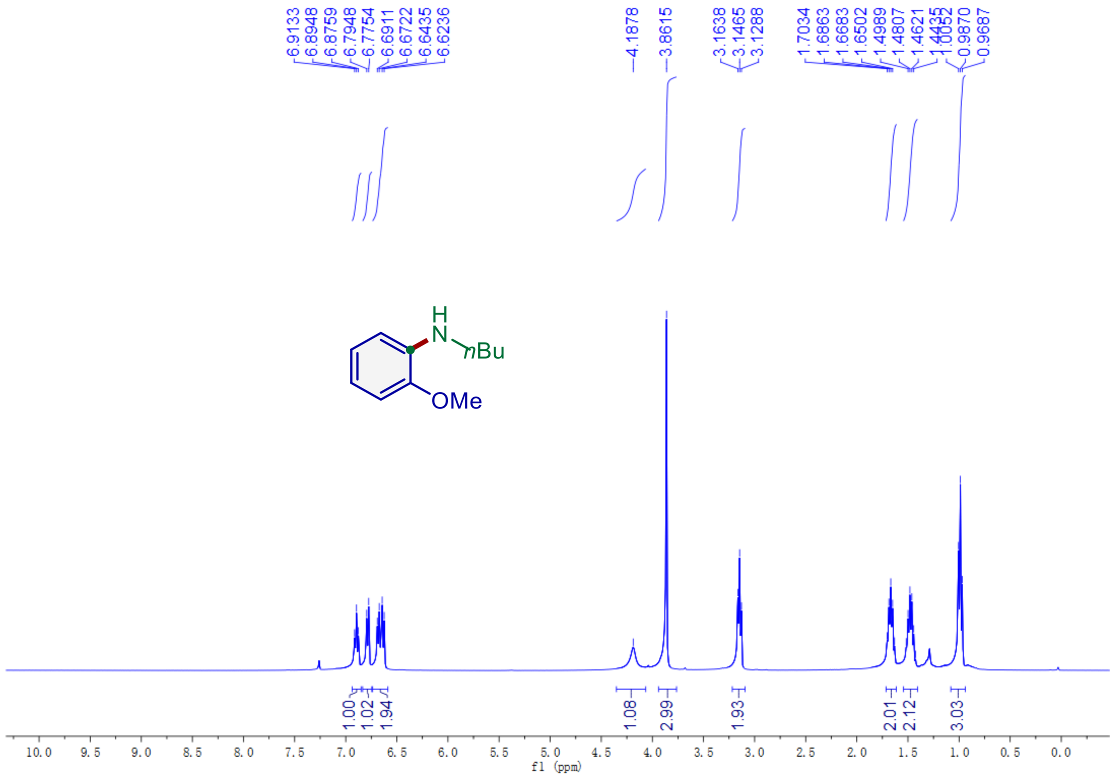

^{1}H NMR (400 MHz, $CDCl_3$) spectrum of compound 30

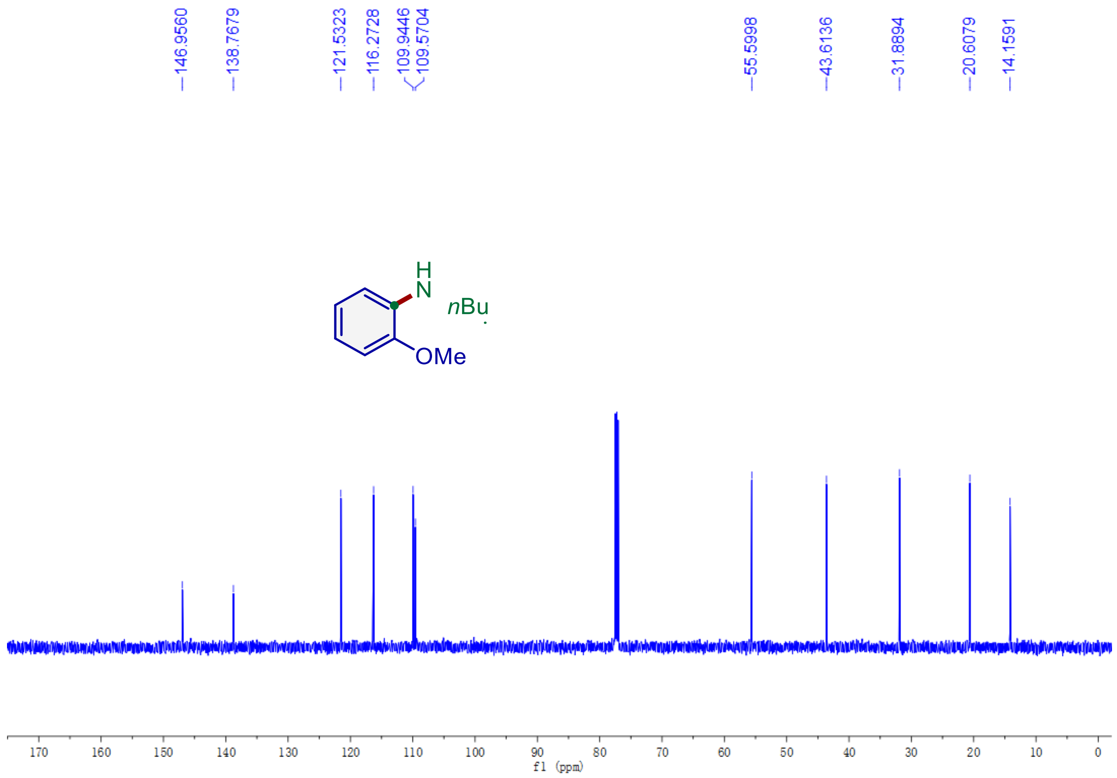

^{13}C NMR (100 MHz, $CDCl_3$) spectrum of compound 30

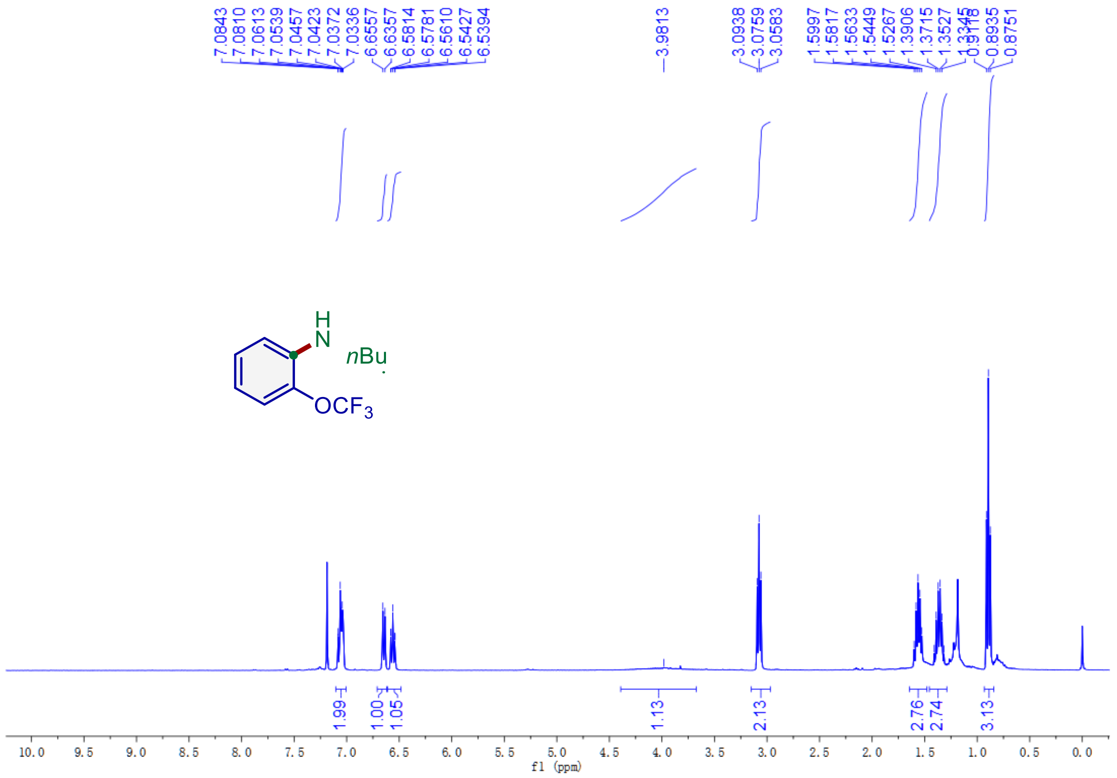

^{1}H NMR (400 MHz, $CDCl_3$) spectrum of compound 31

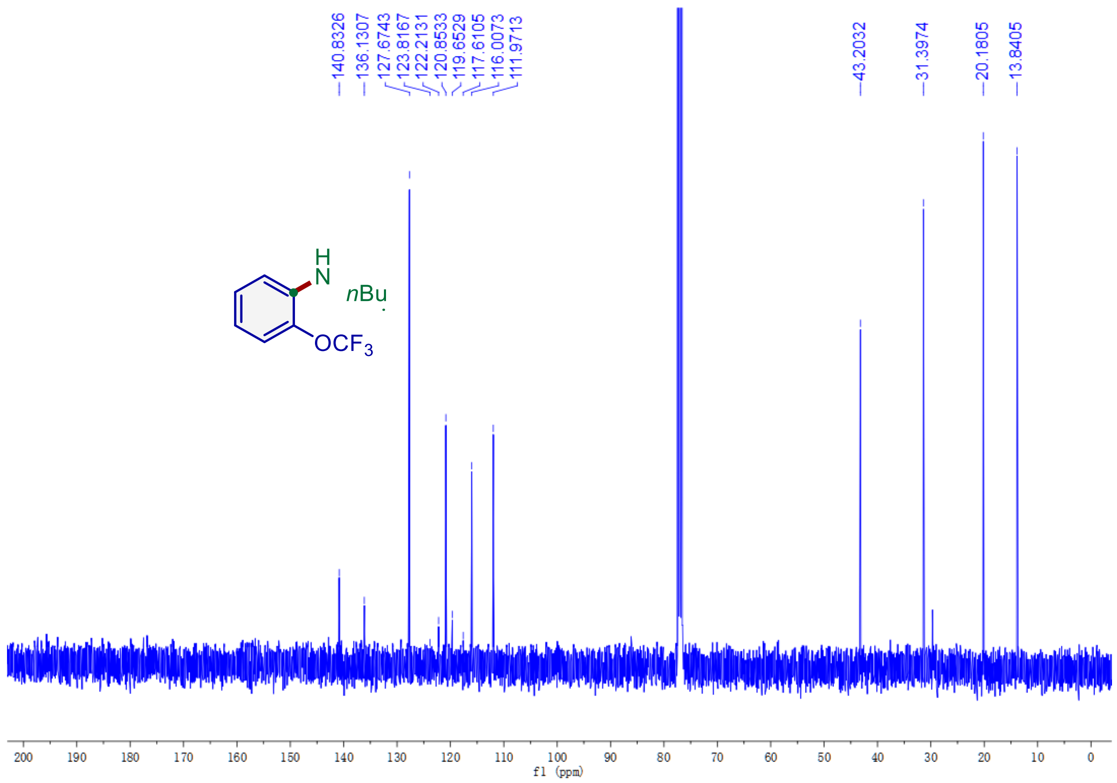

^{13}C NMR (100 MHz, $CDCl_3$) spectrum of compound 31

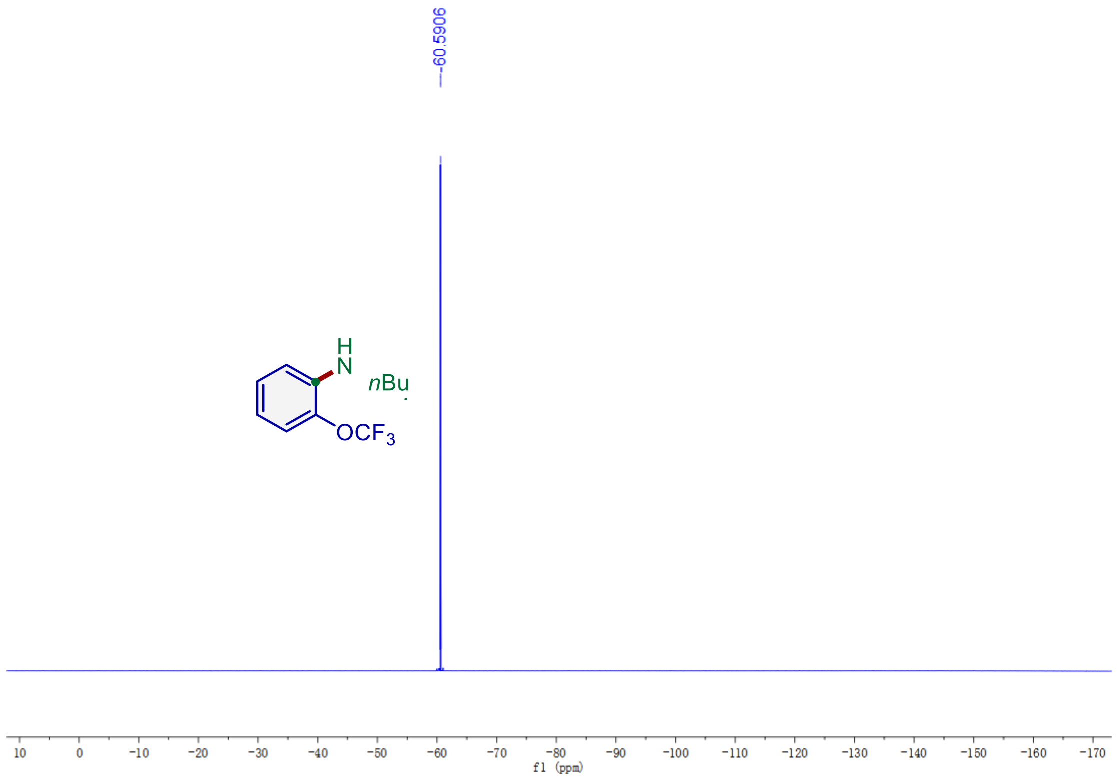

^{19}F NMR (376MHz, $CDCl_3$) spectrum of compound 31

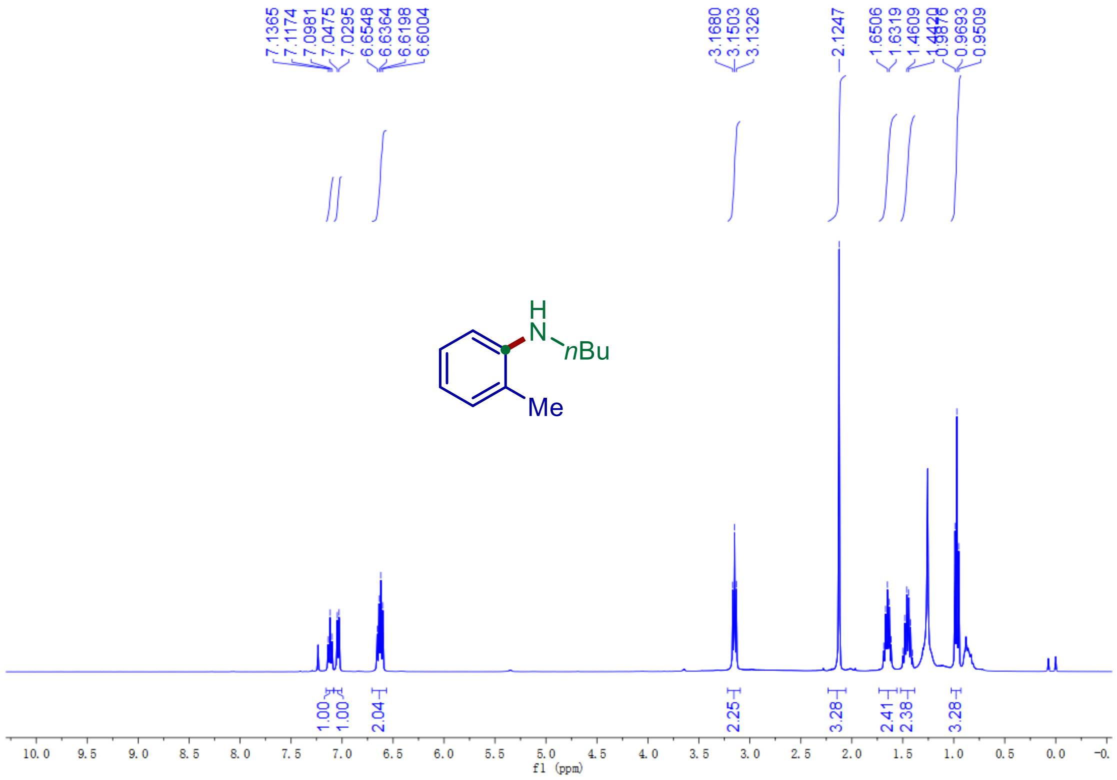

^{1}H NMR (400 MHz, $CDCl_3$) spectrum of compound 32

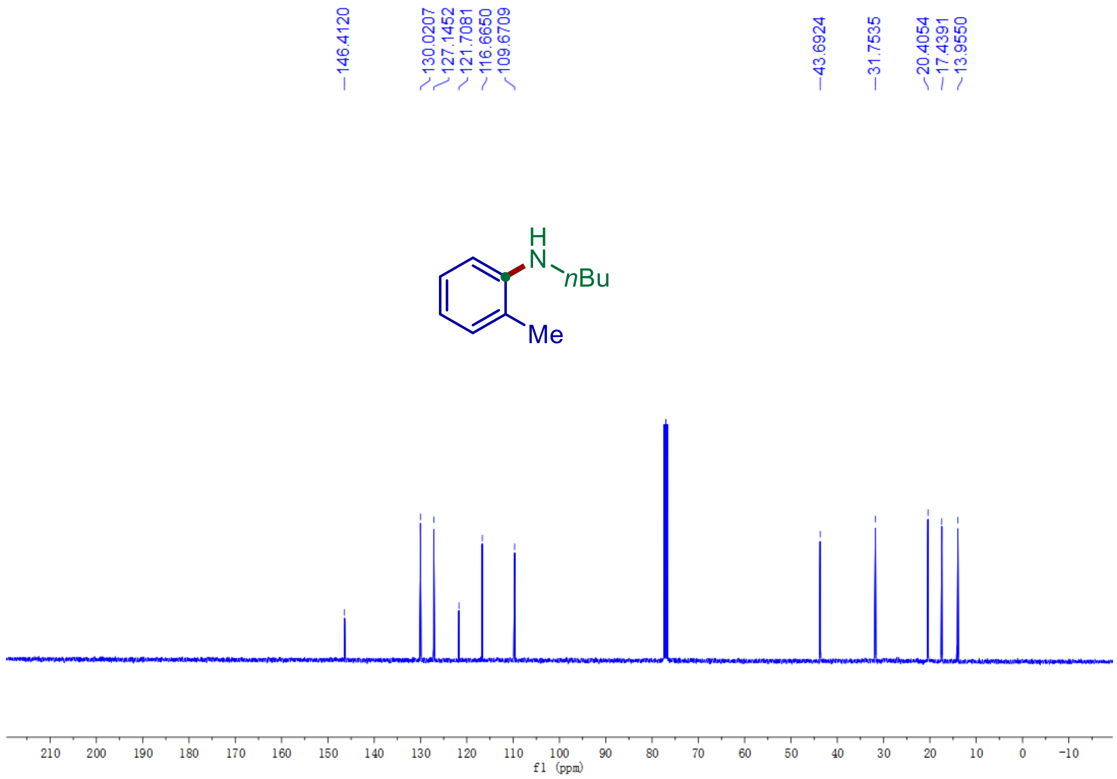

^{13}C NMR (100 MHz, $CDCl_3$) spectrum of compound 32

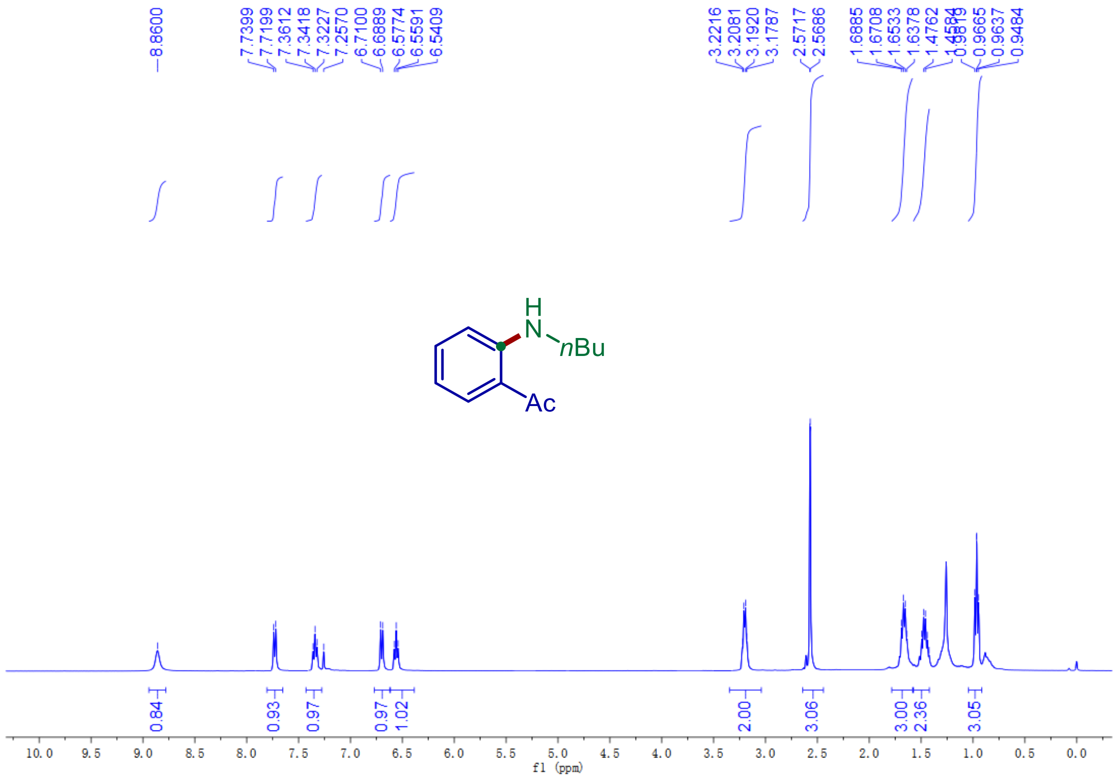

^{1}H NMR (400 MHz, $CDCl_3$) spectrum of compound 33

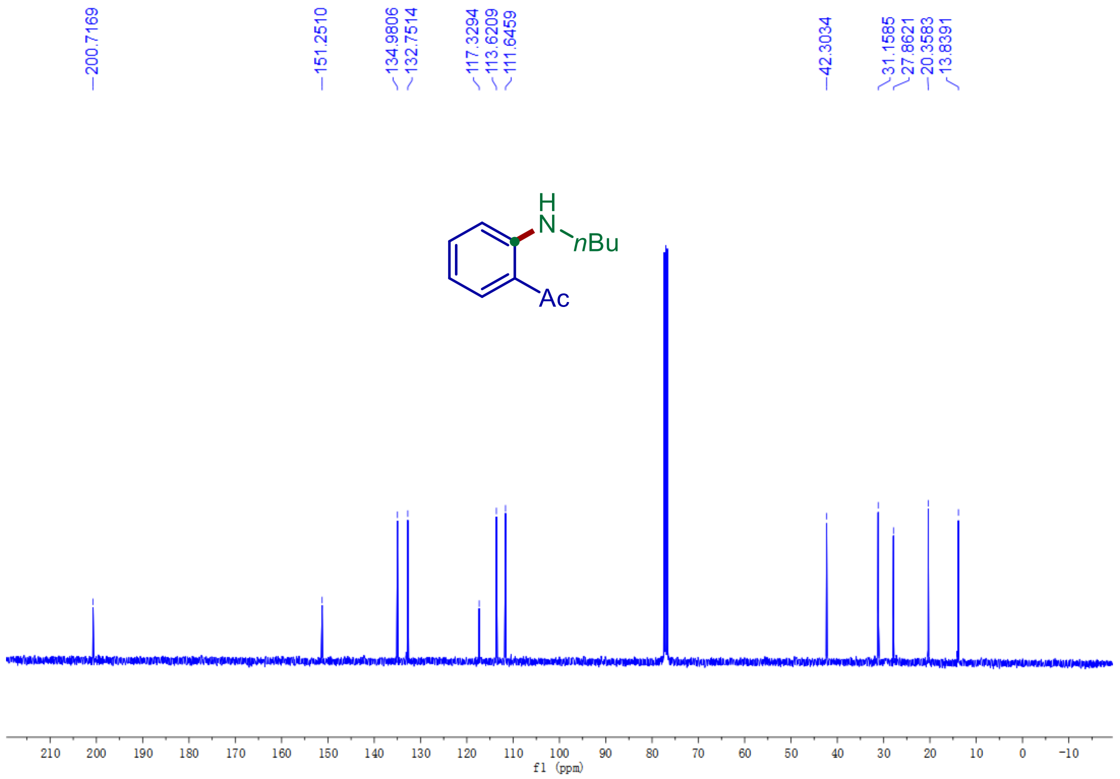

^{13}C NMR (100 MHz, $CDCl_3$) spectrum of compound 33

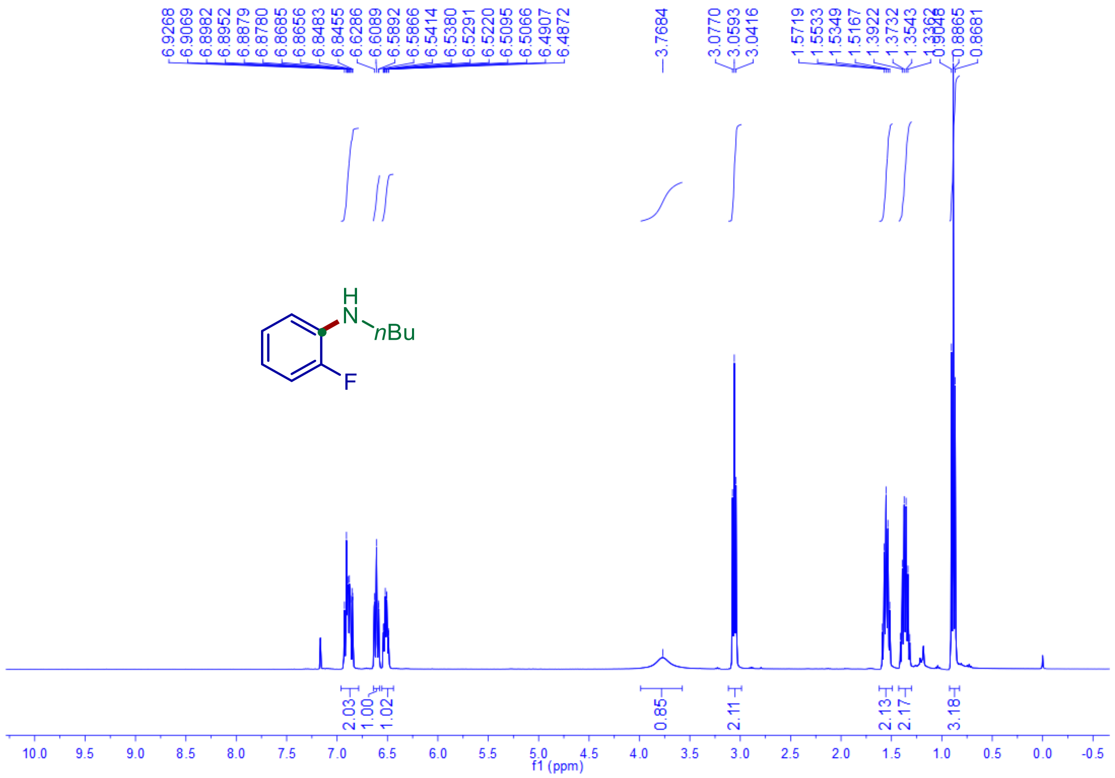

^{1}H NMR (400 MHz, $CDCl_3$) spectrum of compound 34

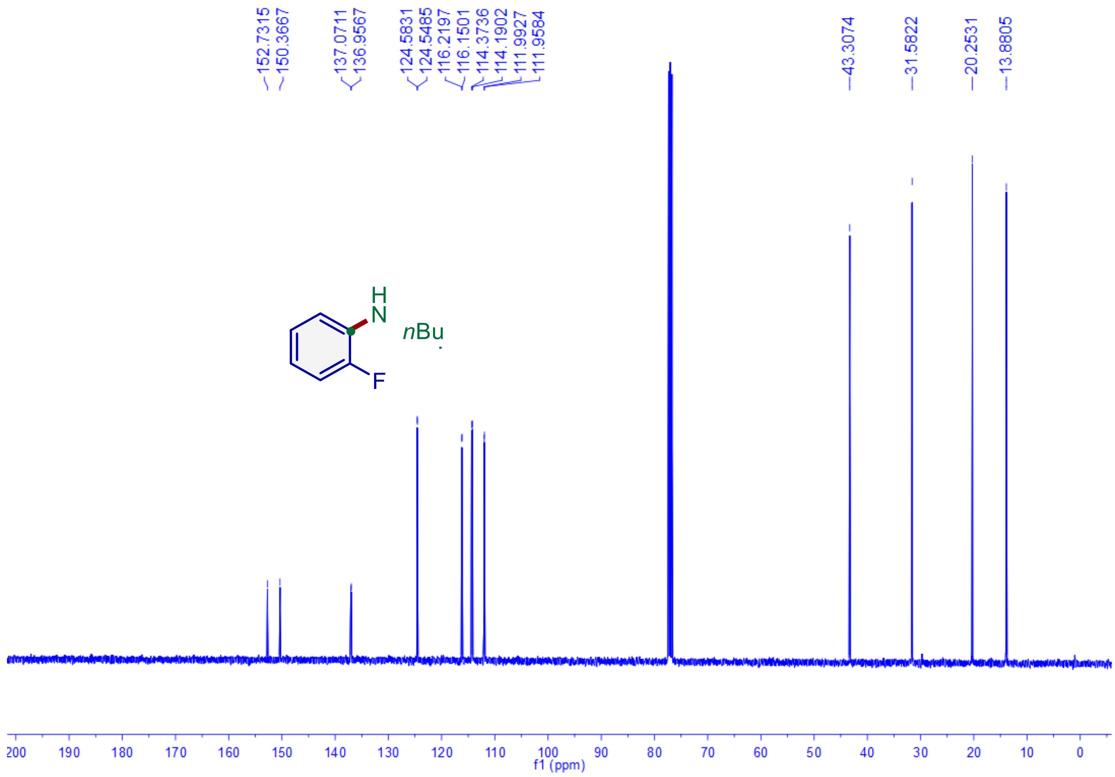

^{13}C NMR (100 MHz, $CDCl_3$) spectrum of compound 34

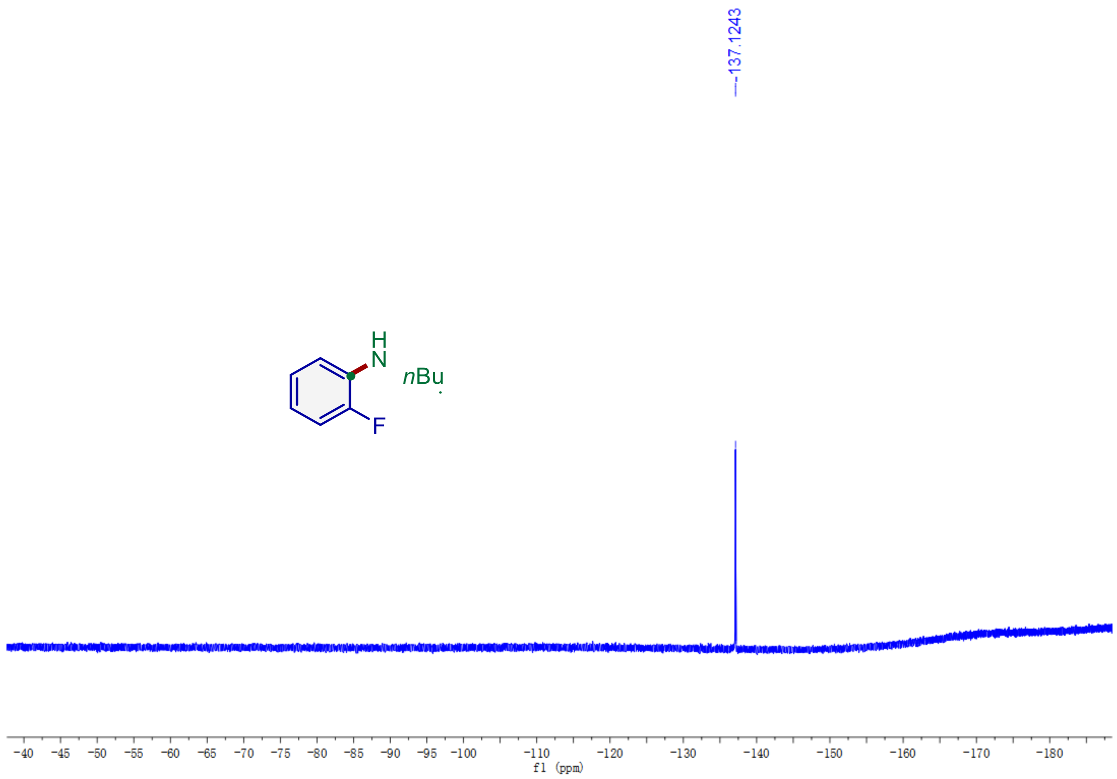

^{19}F NMR (376 MHz, $CDCl_3$) spectrum of compound 34

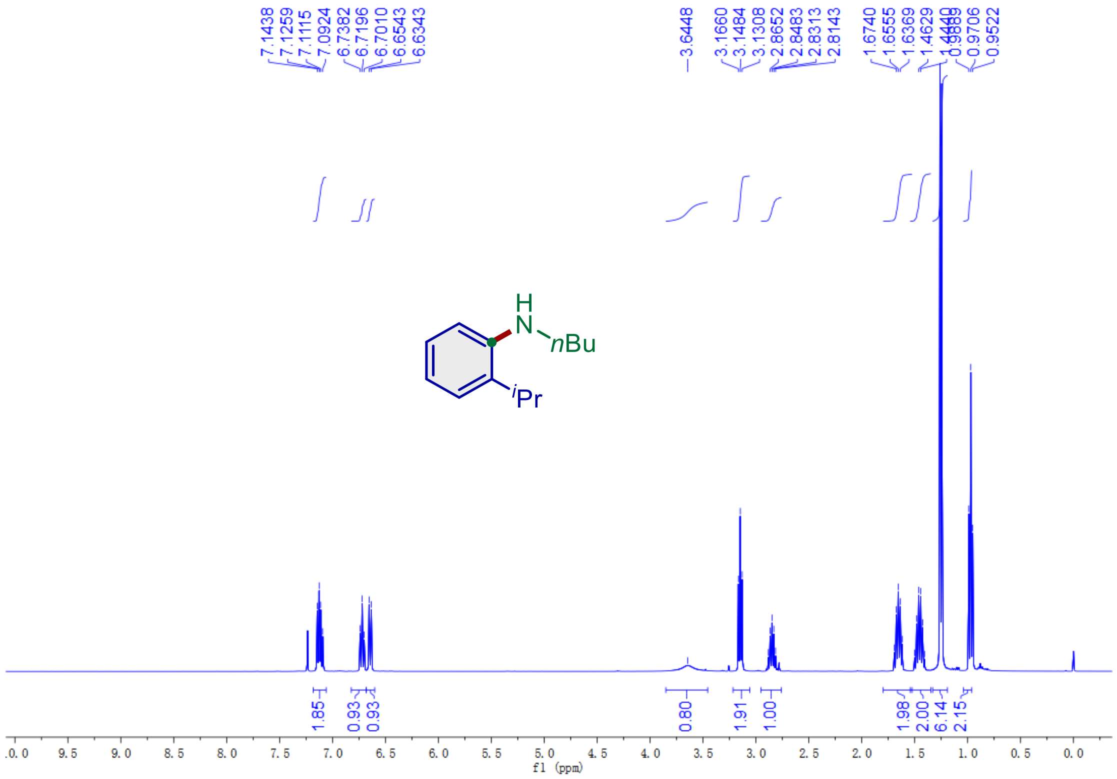

^{1}H NMR (400 MHz, $CDCl_3$) spectrum of compound 35

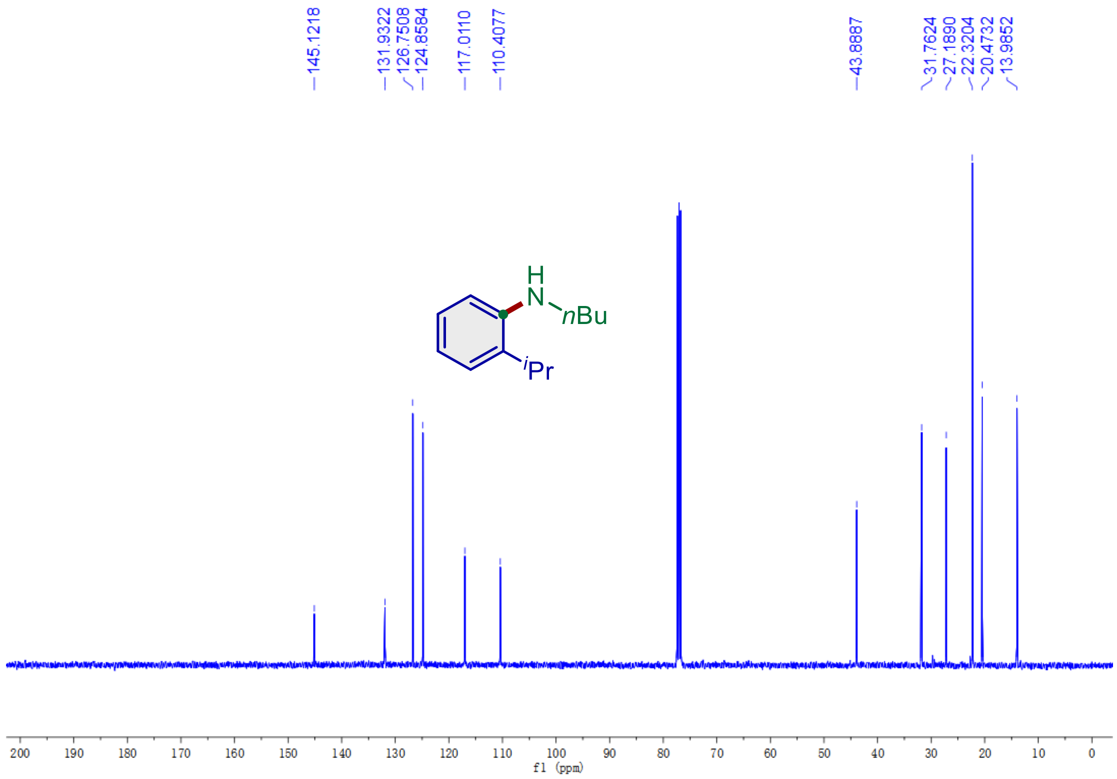

^{13}C NMR (100 MHz, $CDCl_3$) spectrum of compound 35

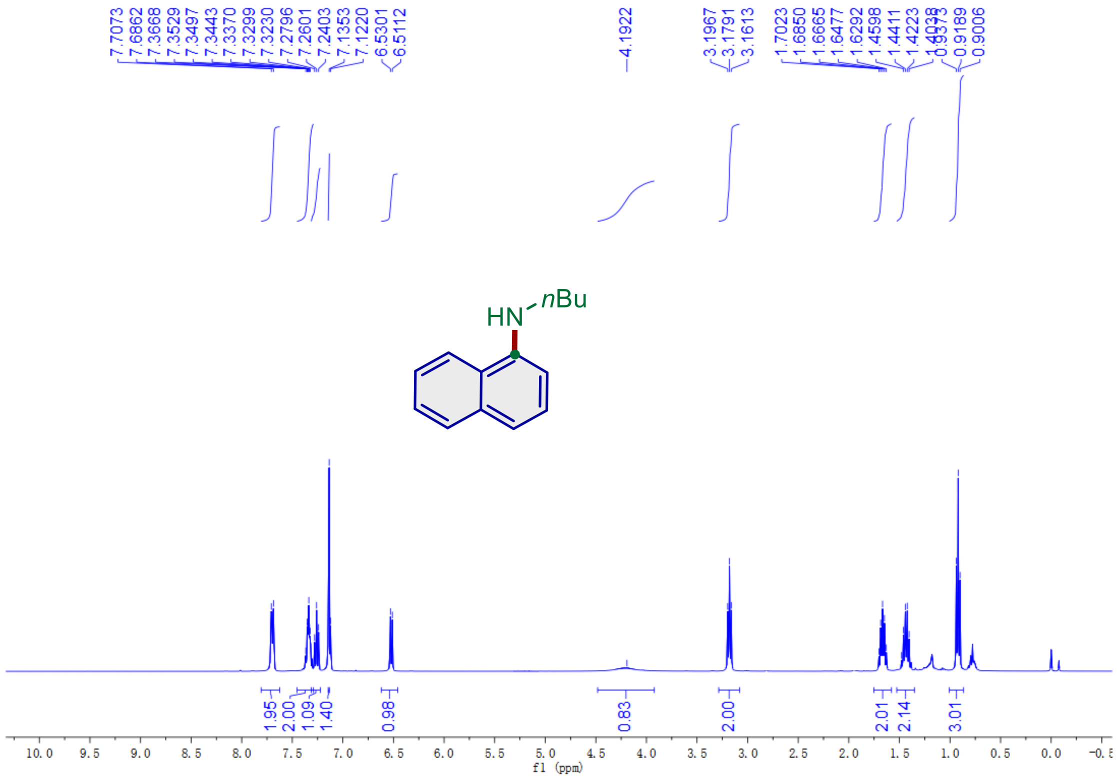

^{1}H NMR (400 MHz, $CDCl_3$) spectrum of compound 36

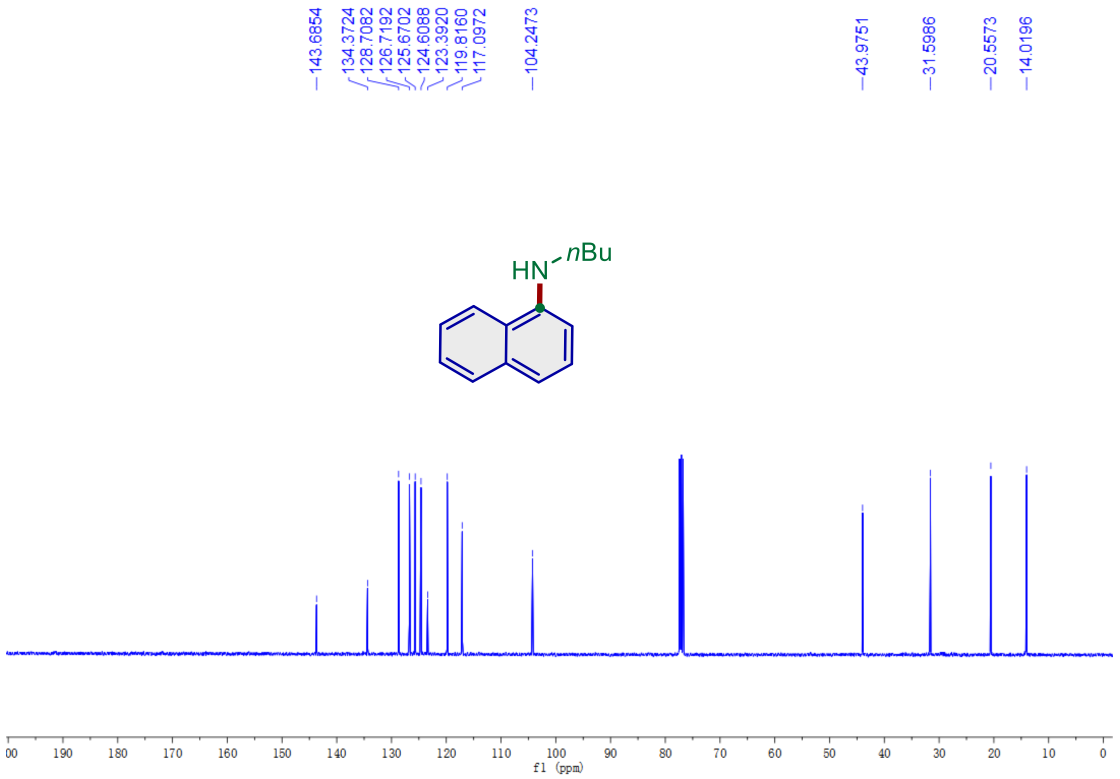

^{13}C NMR (100 MHz, $CDCl_3$) spectrum of compound 36

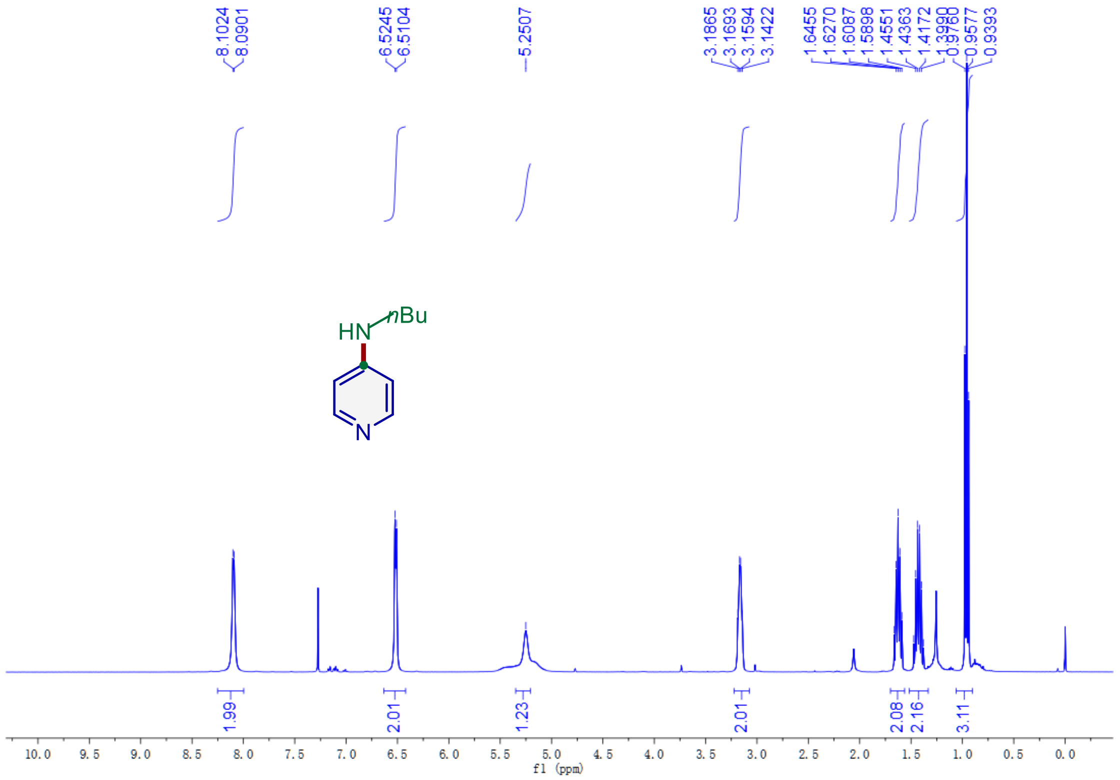

^{1}H NMR (400 MHz, $CDCl_3$) spectrum of compound 37

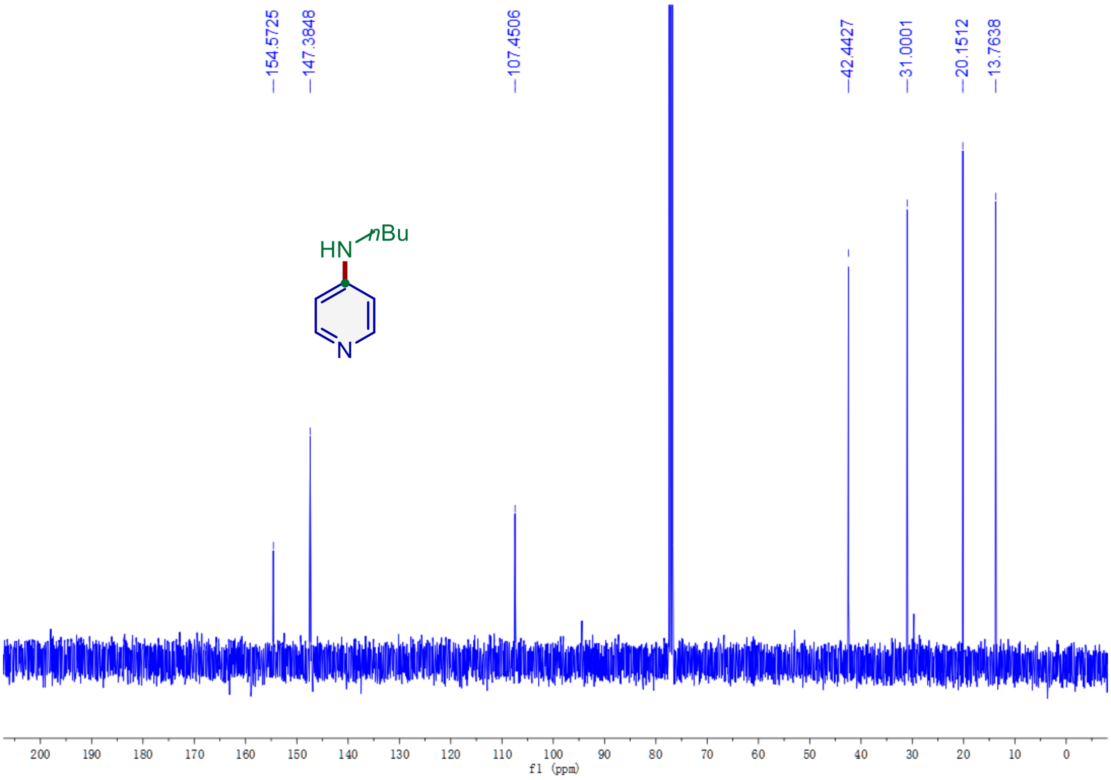

^{13}C NMR (100 MHz, $CDCl_3$) spectrum of compound 37

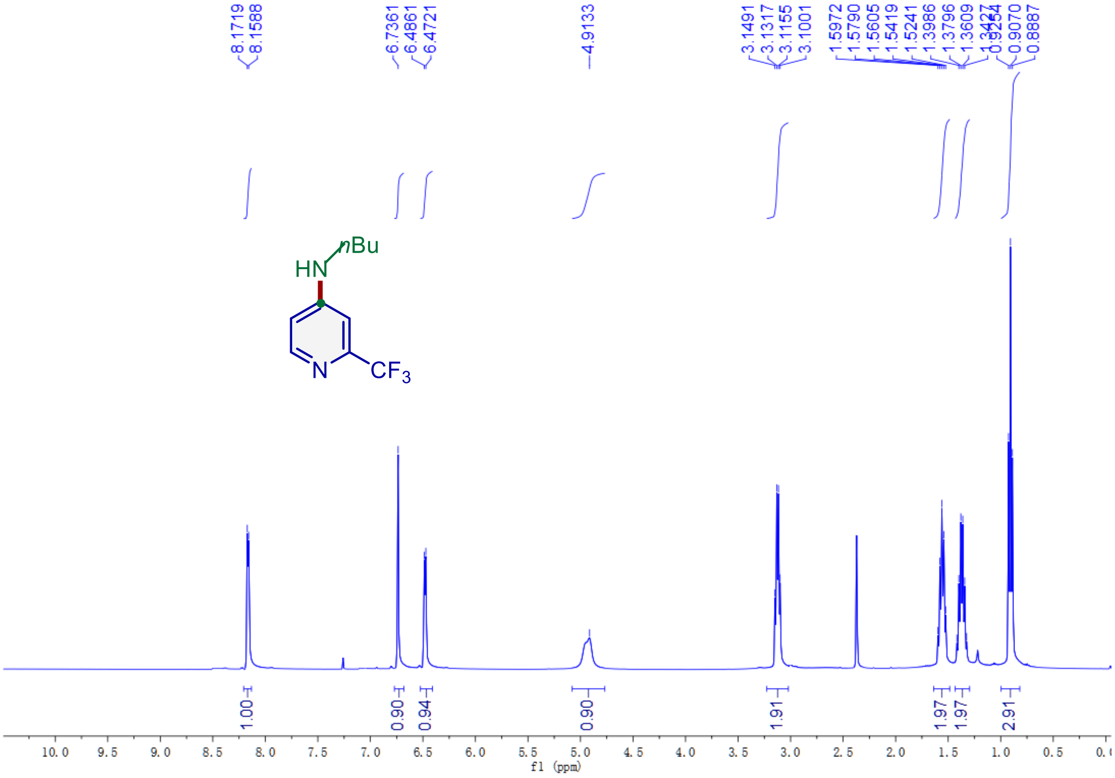

^{1}H NMR (400 MHz, $CDCl_3$) spectrum of compound 38

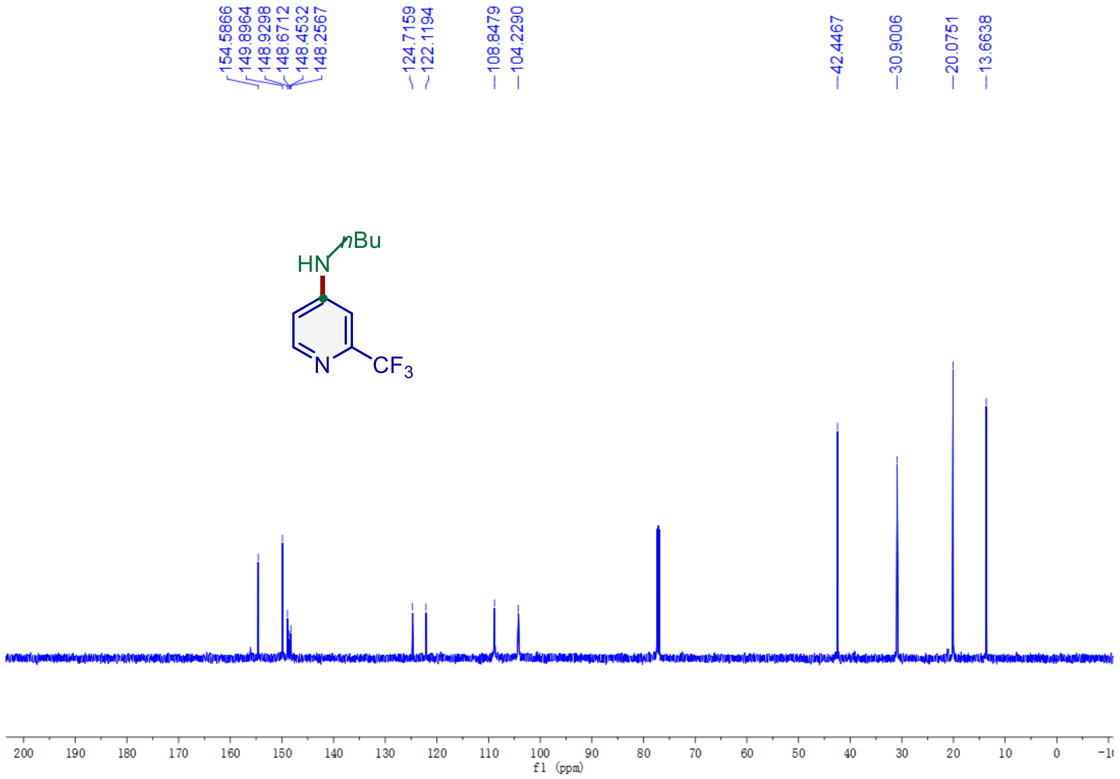

^{13}C NMR (100 MHz, $CDCl_3$) spectrum of compound 38

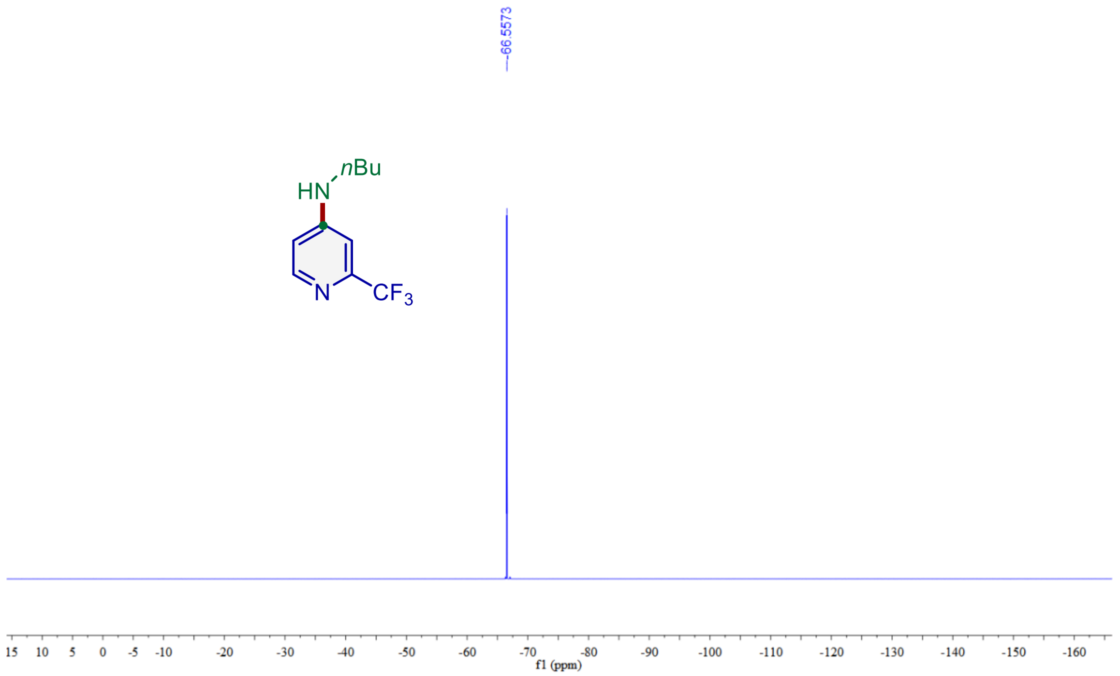

^{19}F NMR (376 MHz, $CDCl_3$) spectrum of compound 38

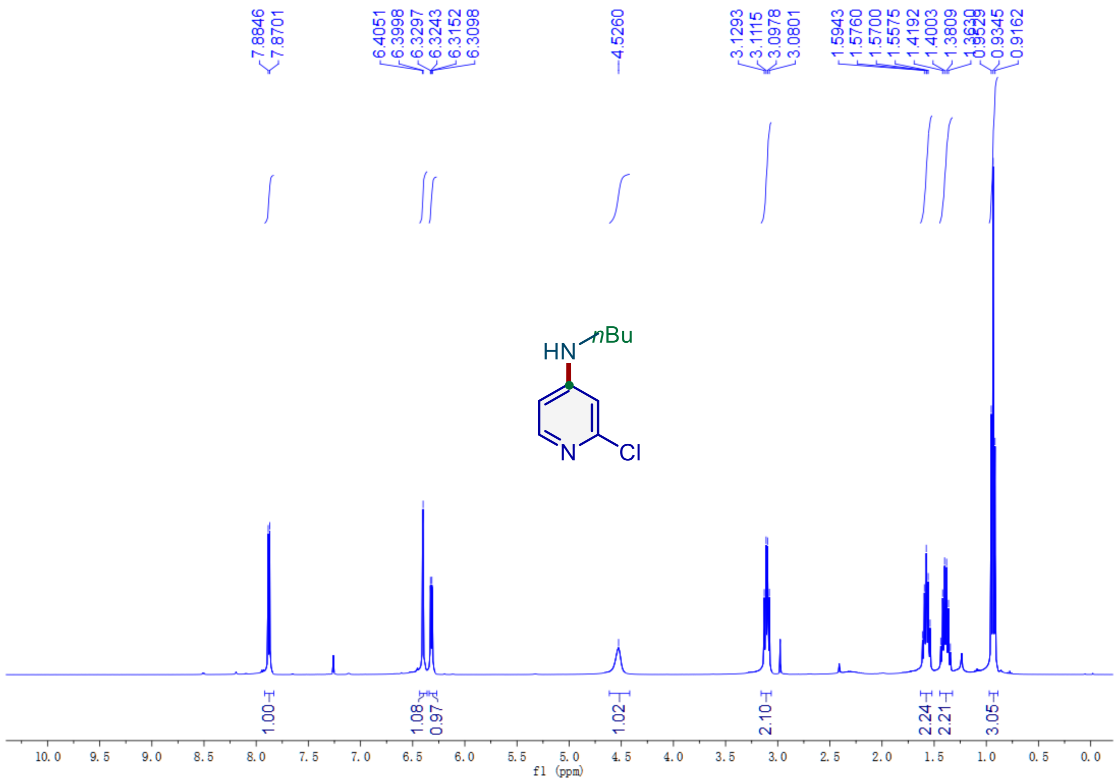

^{1}H NMR (400 MHz, $CDCl_3$) spectrum of compound 39

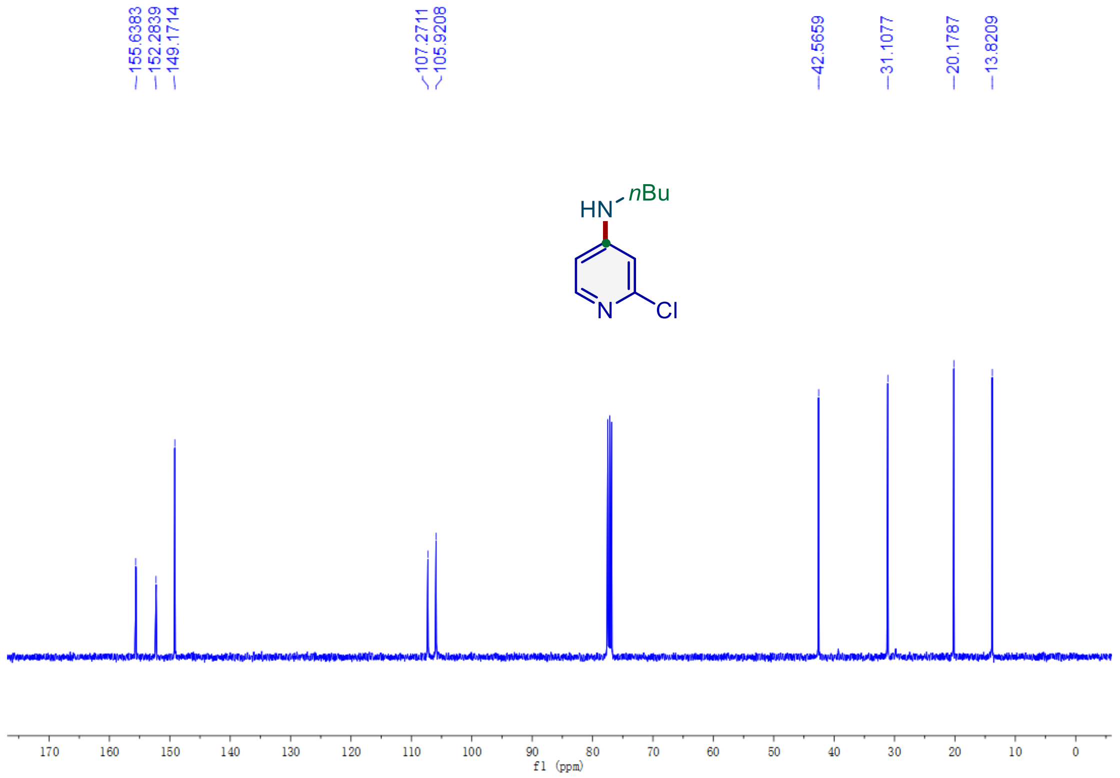

^{13}C NMR (100 MHz, $CDCl_3$) spectrum of compound 39

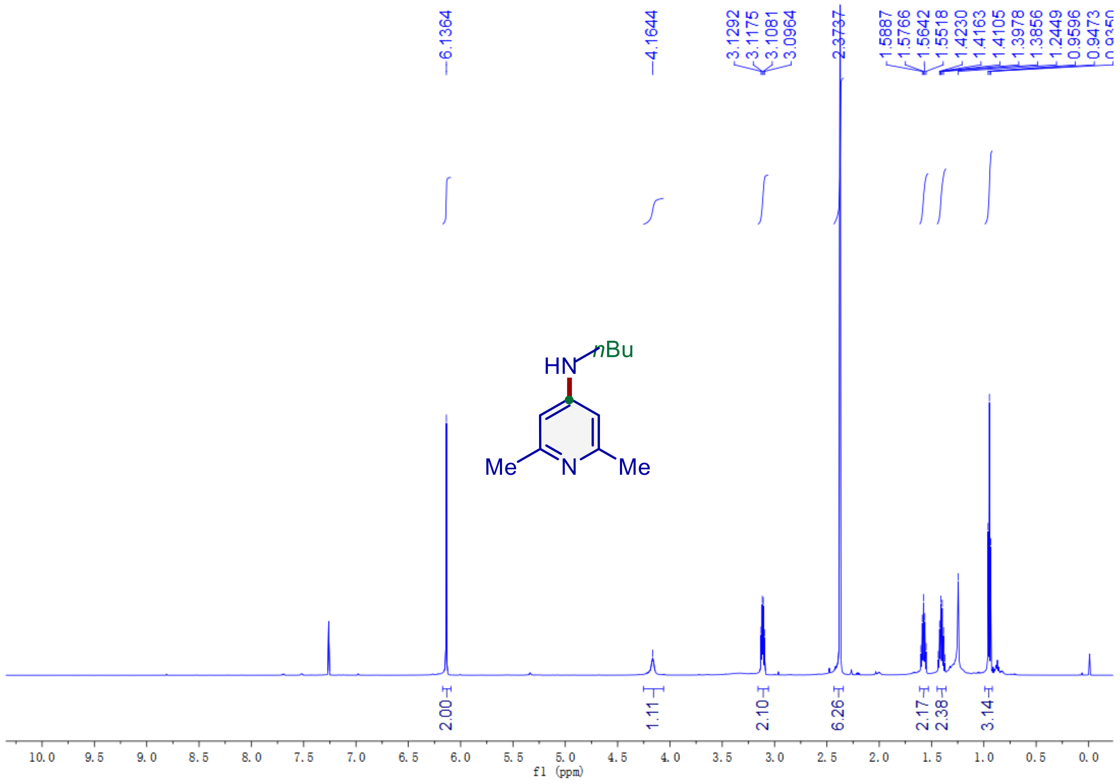

^{1}H NMR (400 MHz, $CDCl_3$) spectrum of compound 40

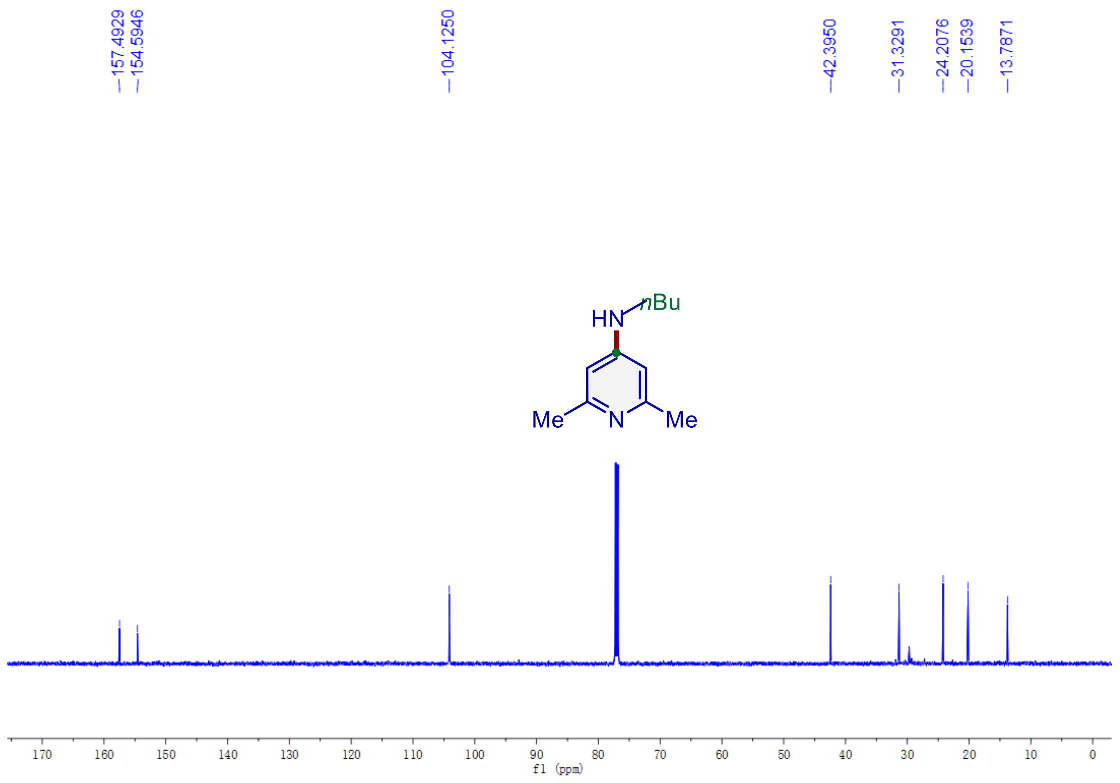

^{13}C NMR (100 MHz, $CDCl_3$) spectrum of compound 40

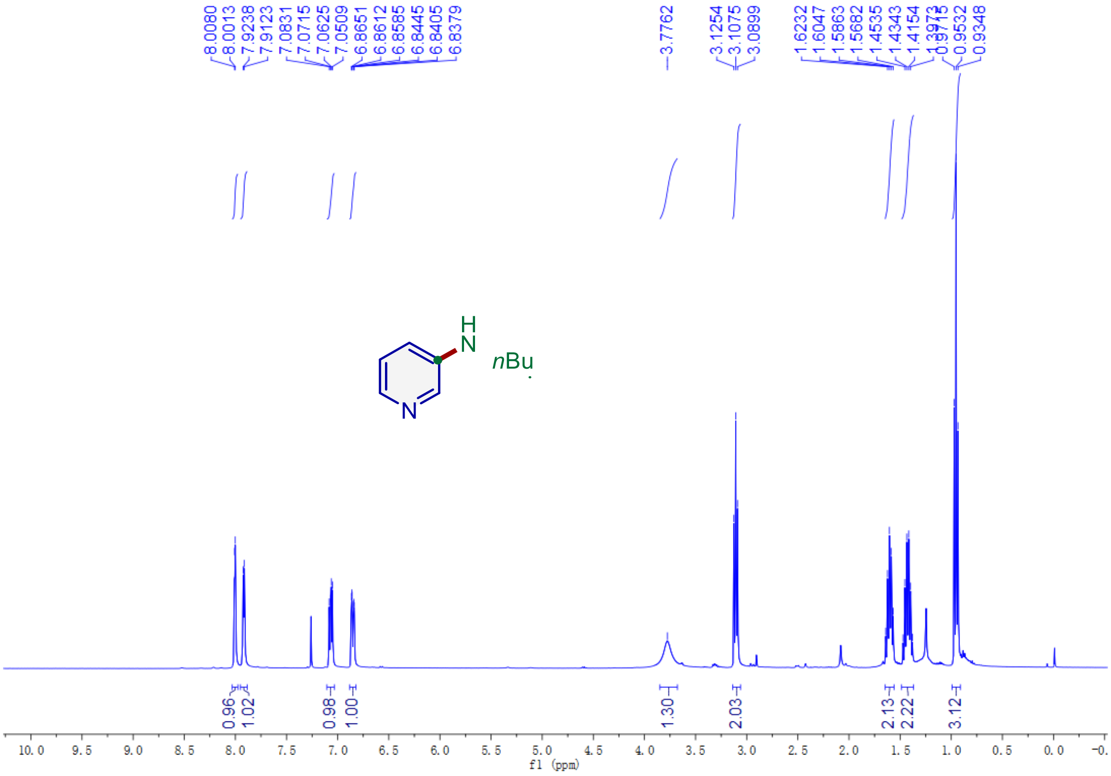

^{1}H NMR (400 MHz, $CDCl_3$) spectrum of compound 41

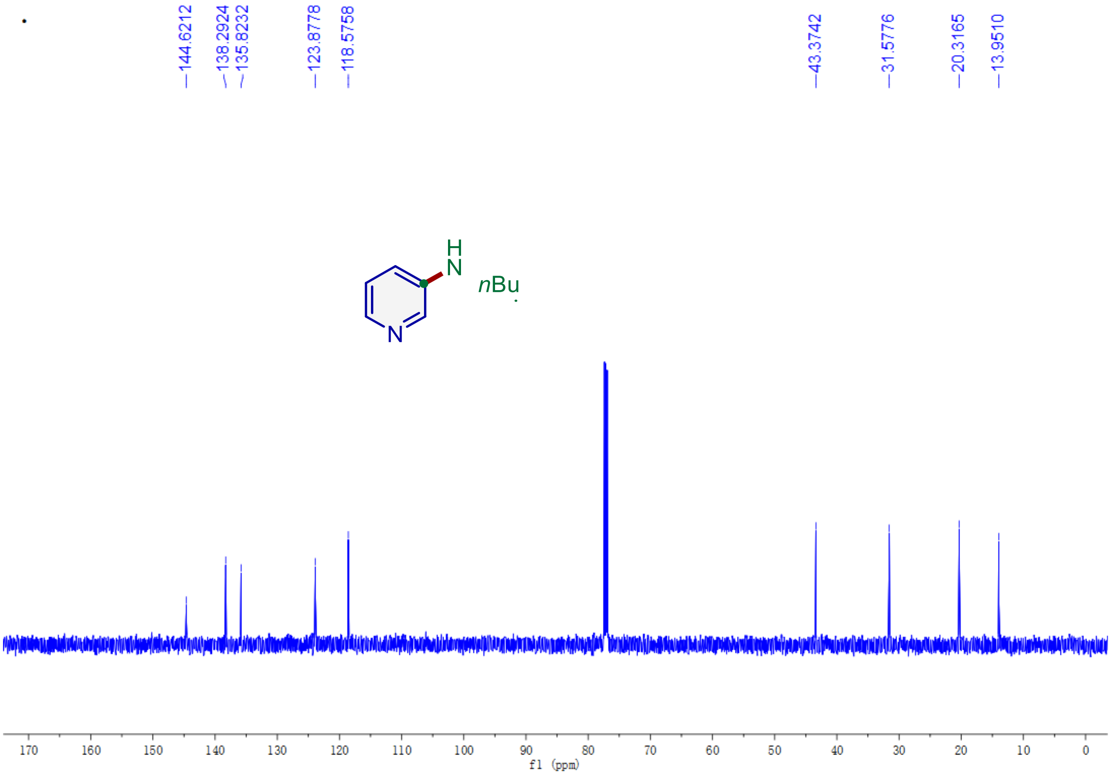

^{13}C NMR (100 MHz, $CDCl_3$) spectrum of compound 41

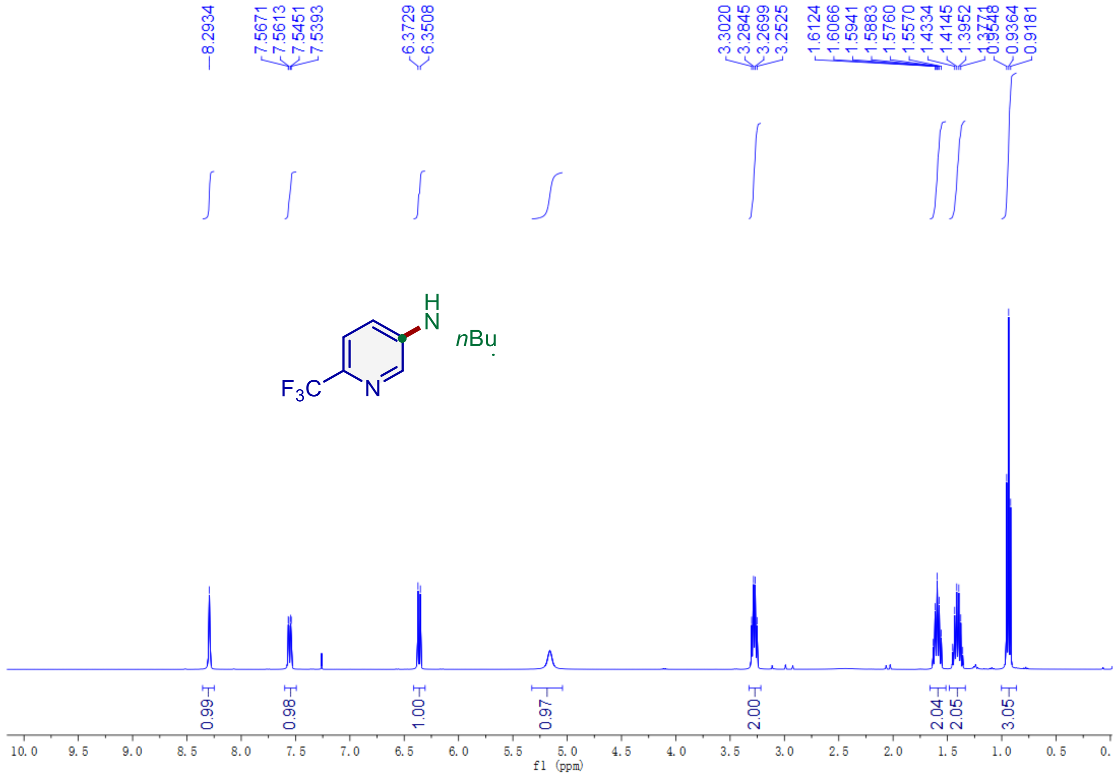

^{1}H NMR (400 MHz, $CDCl_3$) spectrum of compound 42

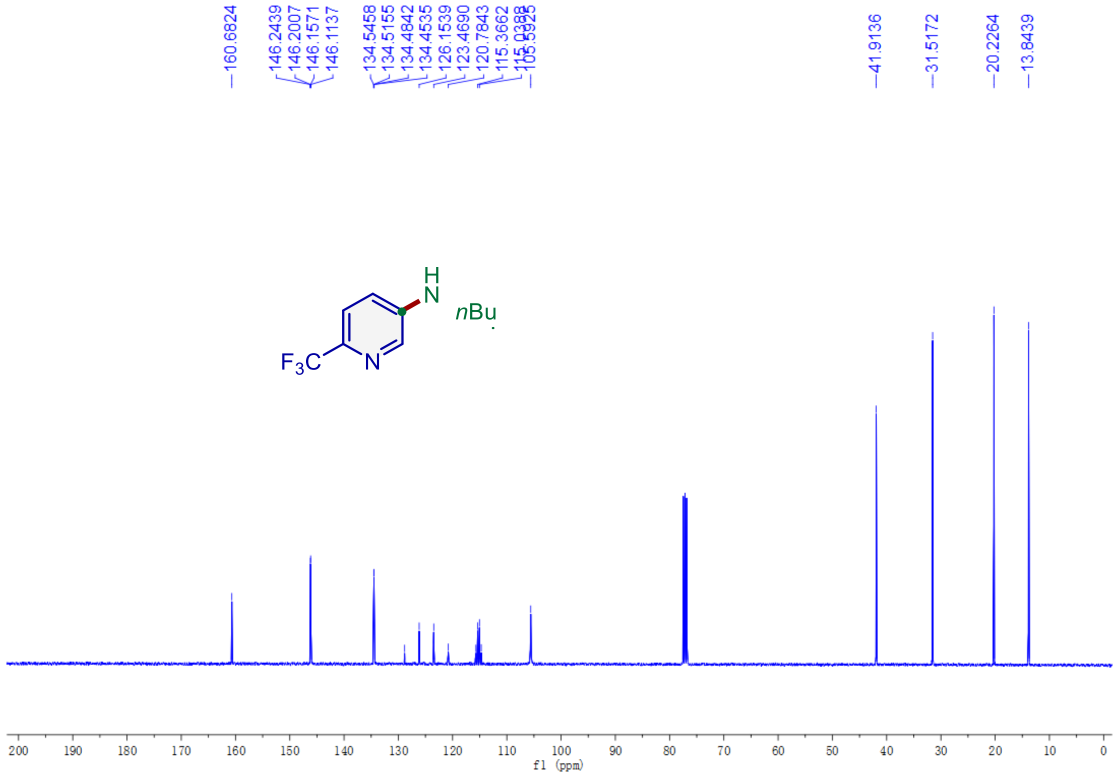

^{13}C NMR (100 MHz, $CDCl_3$) spectrum of compound 42

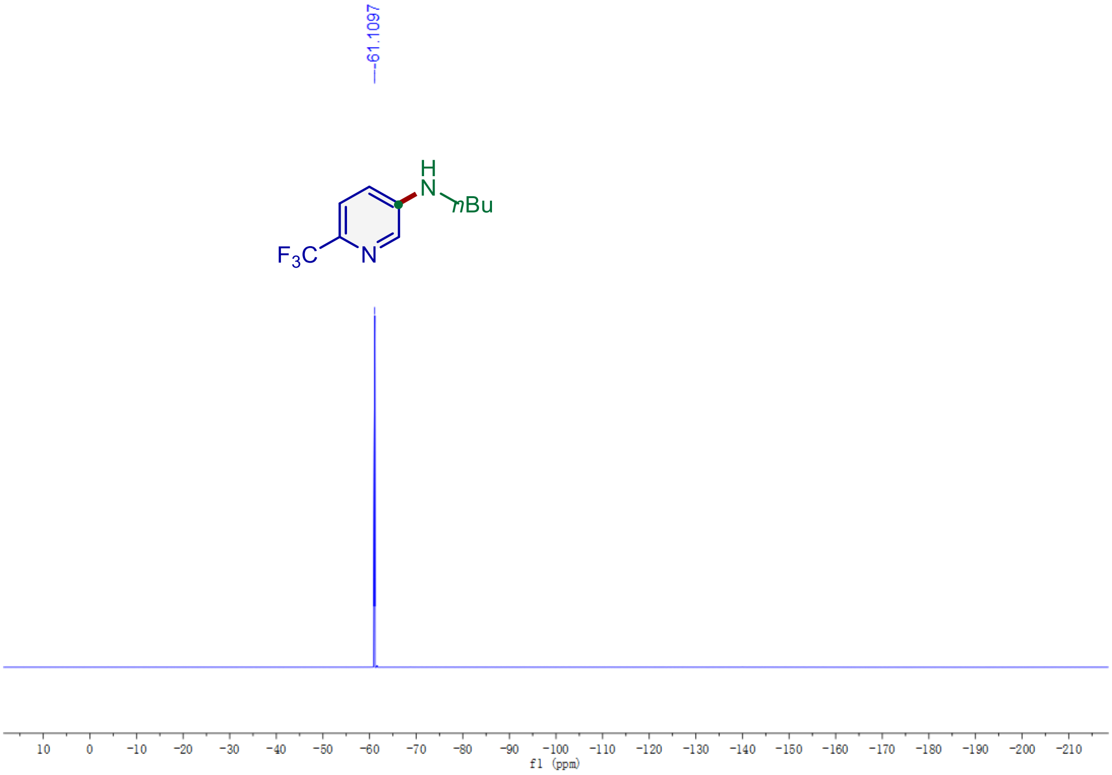

^{19}F NMR (376 MHz, $CDCl_3$) spectrum of compound 42

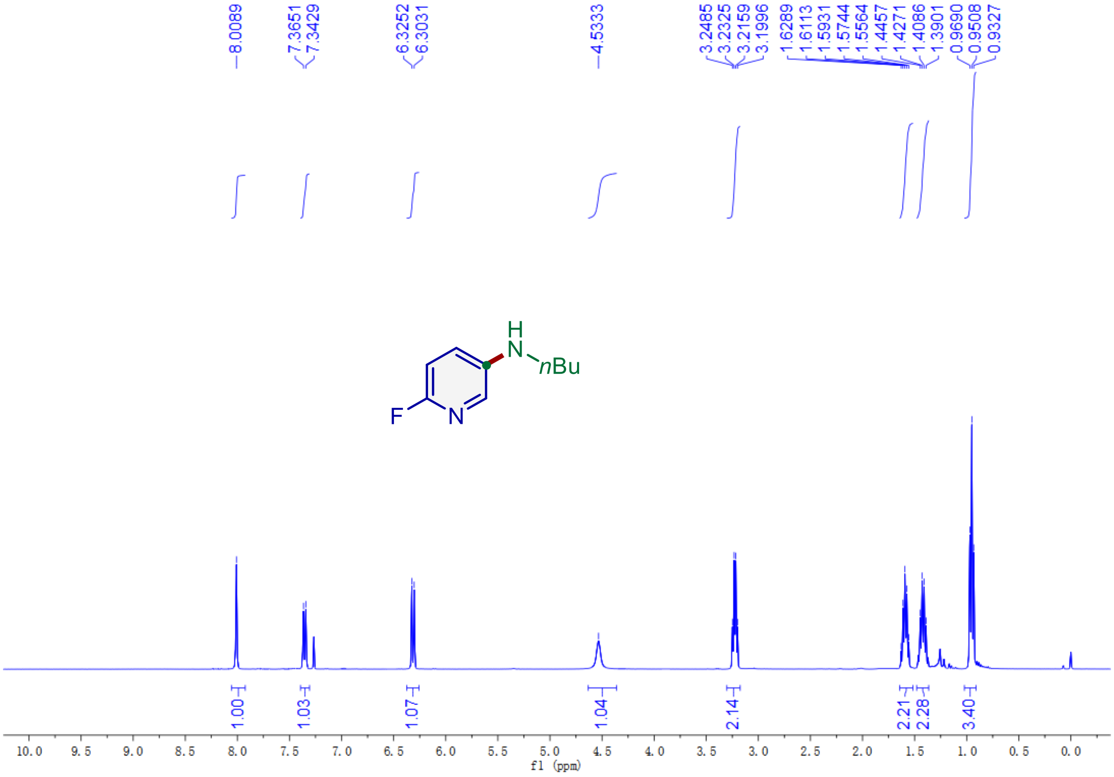

^{1}H NMR (400 MHz, $CDCl_3$) spectrum of compound 43

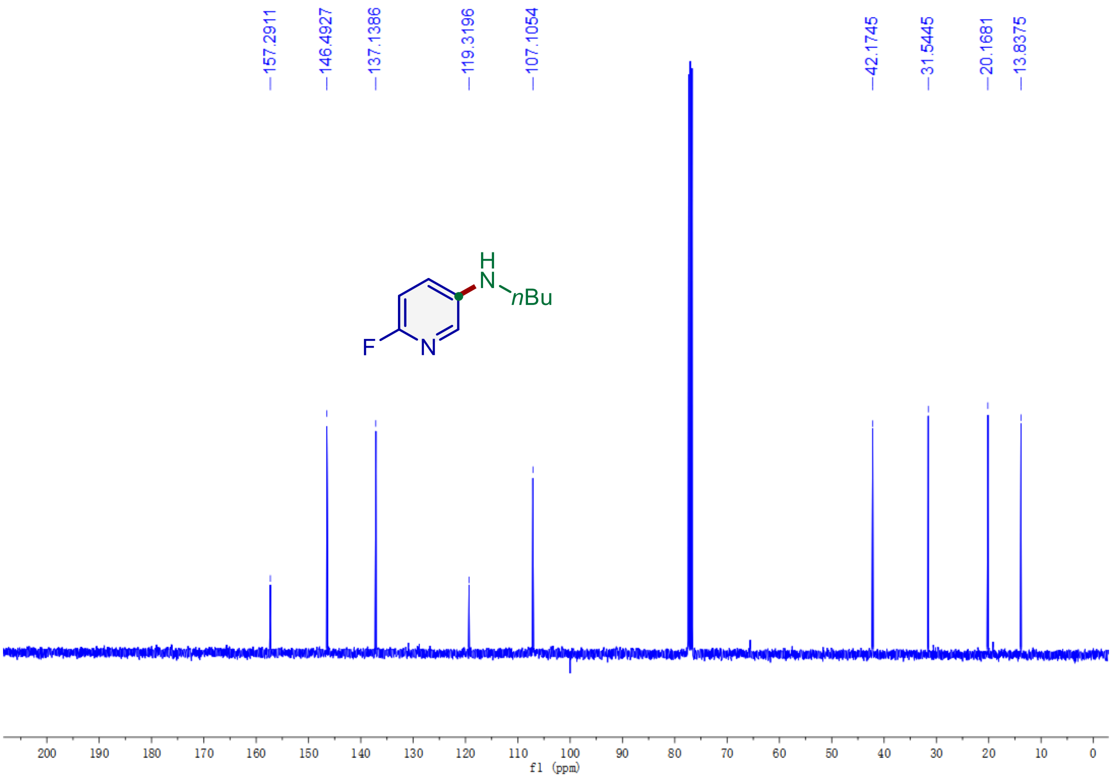

^{13}C NMR (100 MHz, $CDCl_3$) spectrum of compound 43

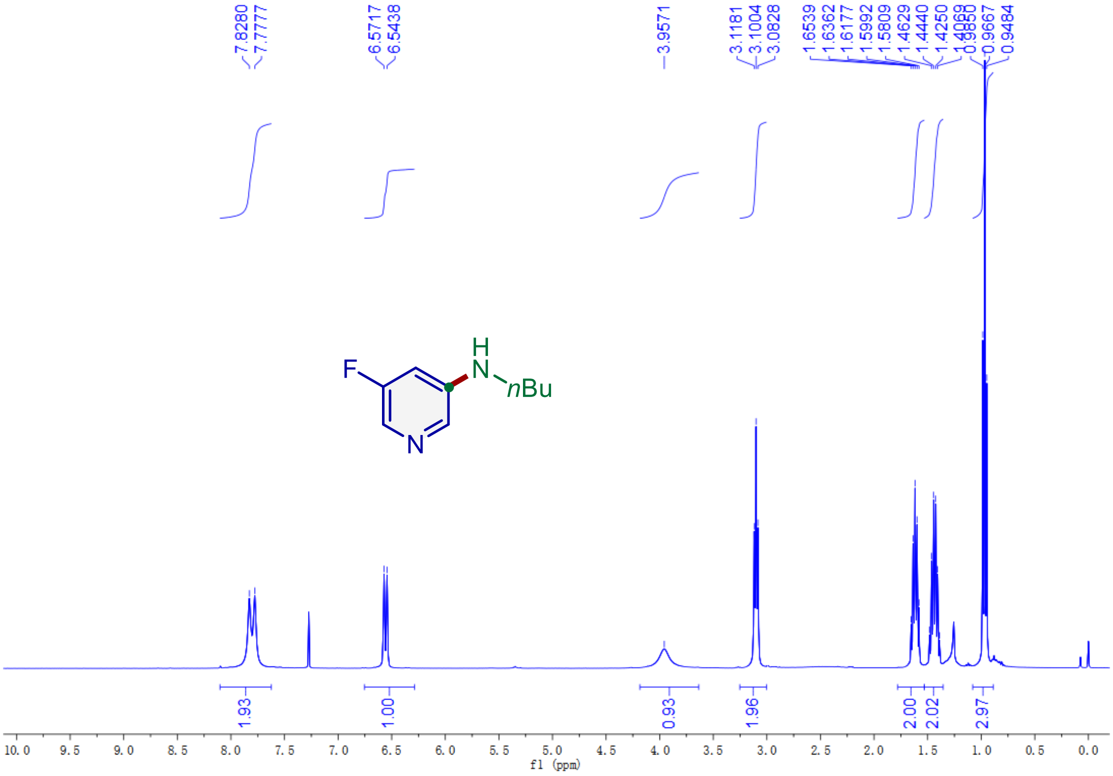

^{1}H NMR (400 MHz, $CDCl_3$) spectrum of compound 44

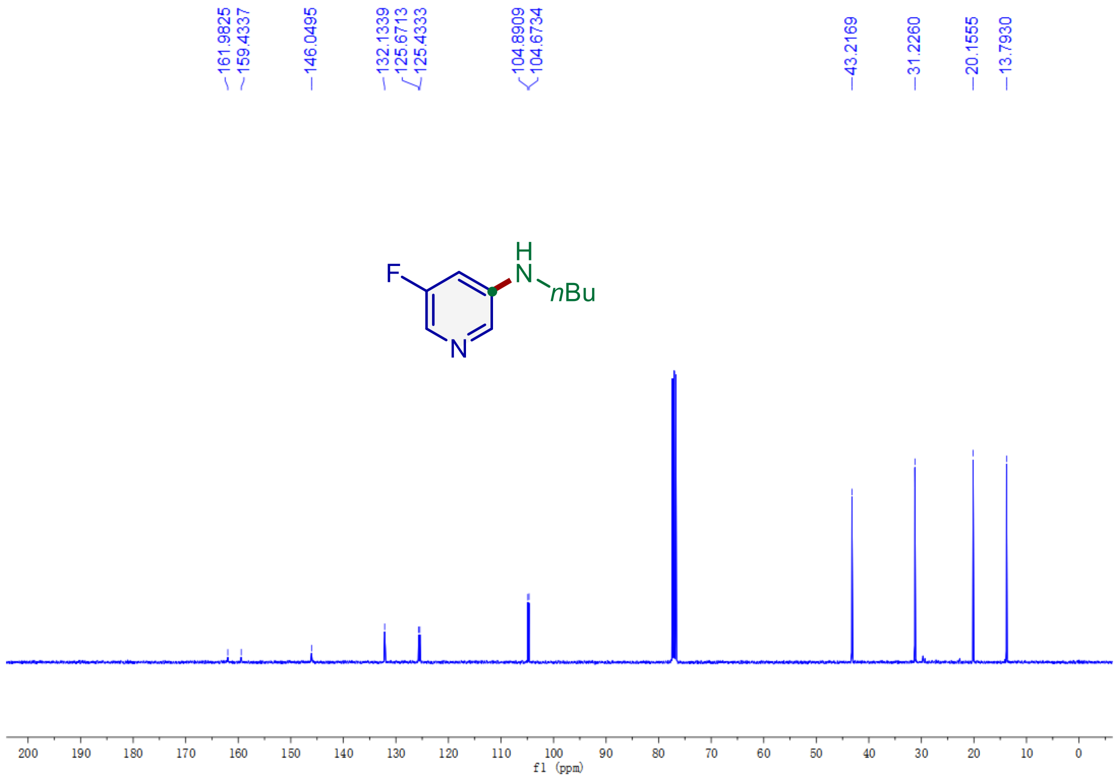

^{13}C NMR (100 MHz, $CDCl_3$) spectrum of compound 44

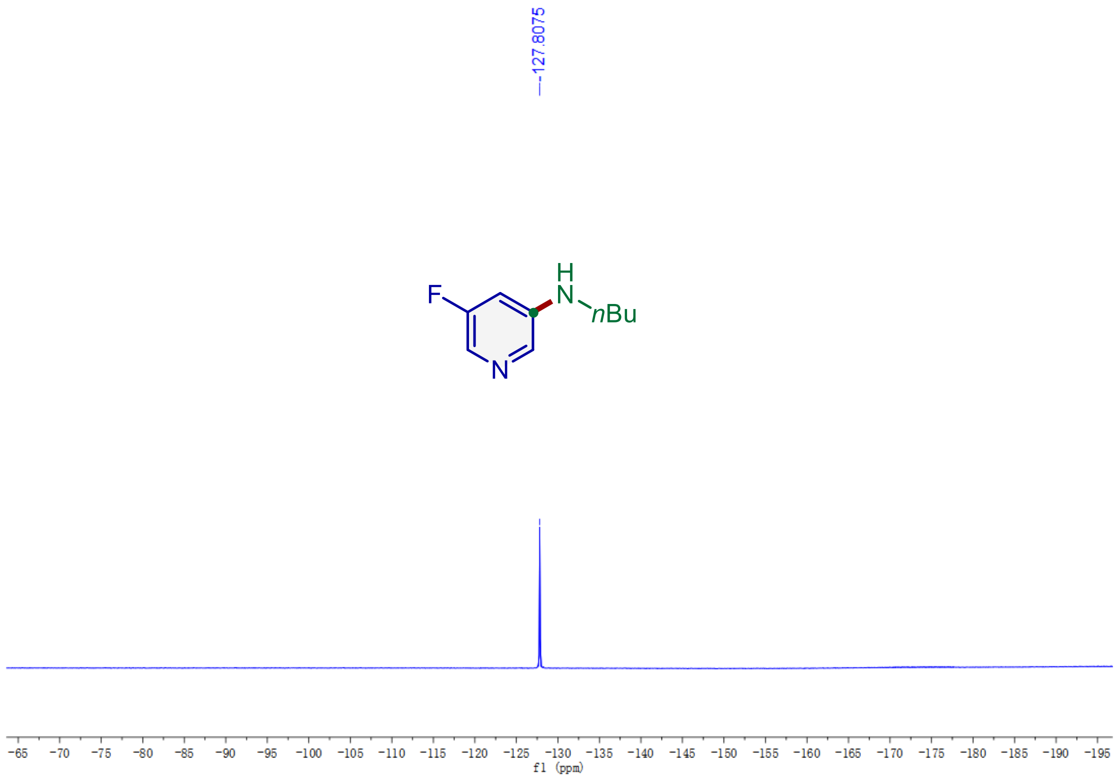

^{19}F NMR (376 MHz, $CDCl_3$) spectrum of compound 44

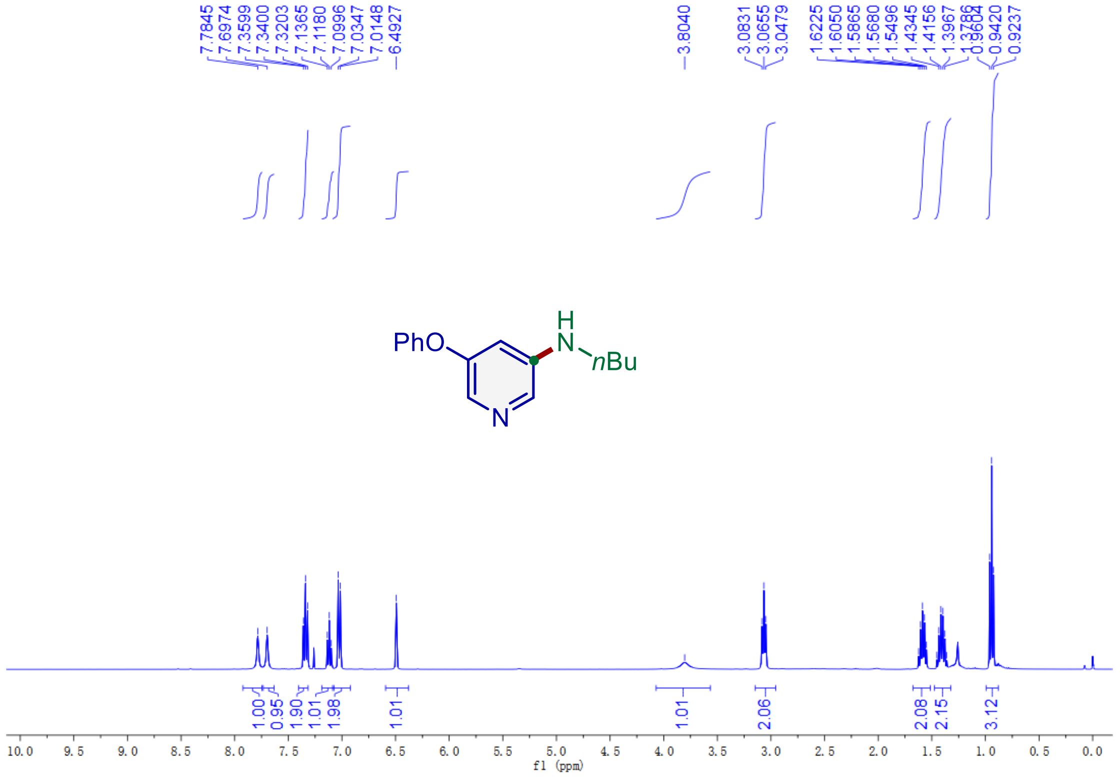

^{1}H NMR (400 MHz, $CDCl_3$) spectrum of compound 45

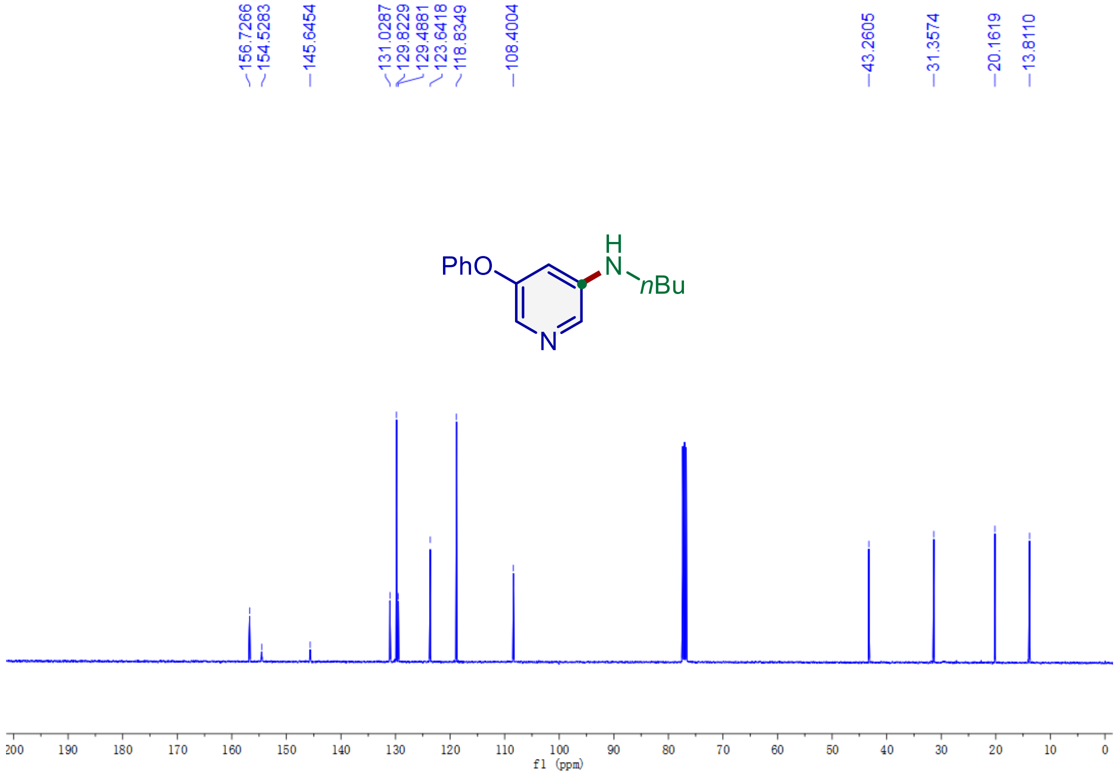

^{13}C NMR (100 MHz, $CDCl_3$) spectrum of compound 45

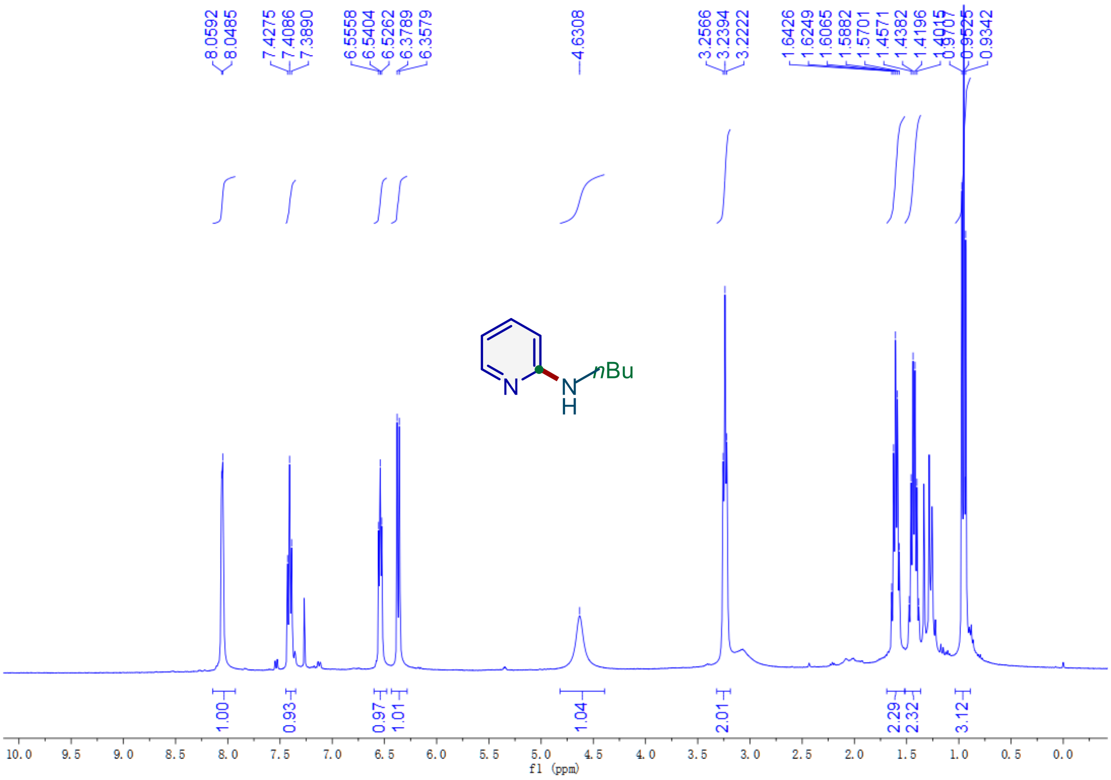

^{1}H NMR (400 MHz, $CDCl_3$) spectrum of compound 46

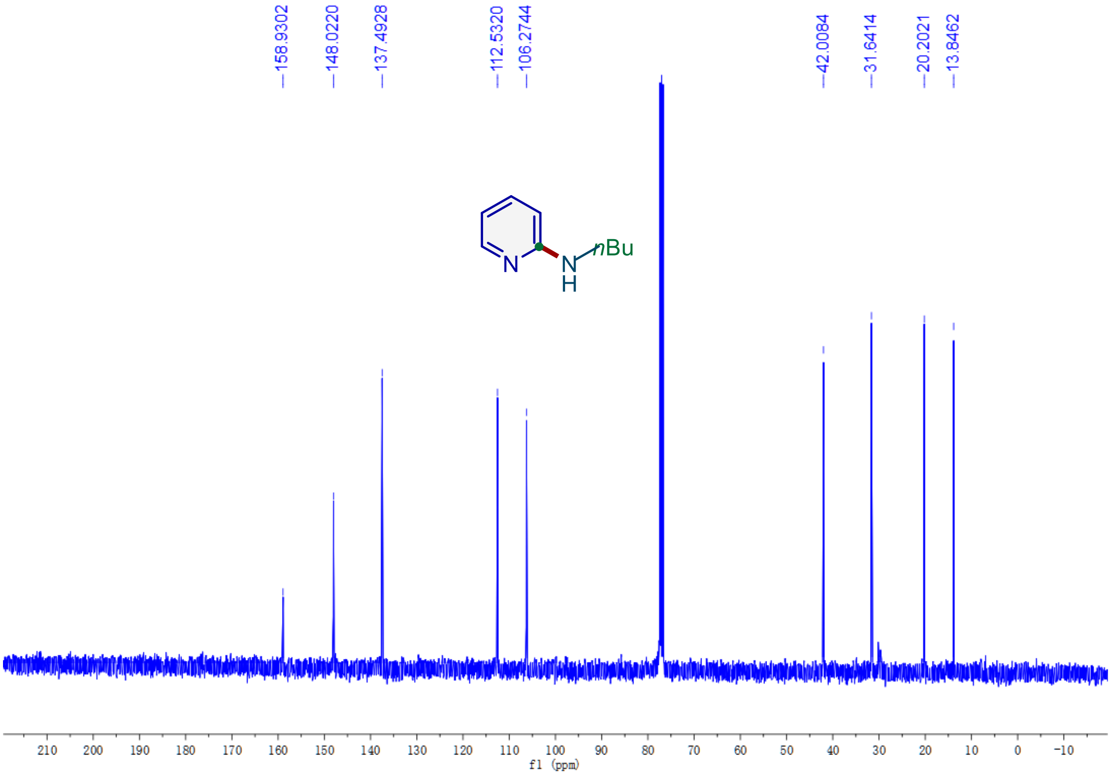

^{13}C NMR (100 MHz, $CDCl_3$) spectrum of compound 46

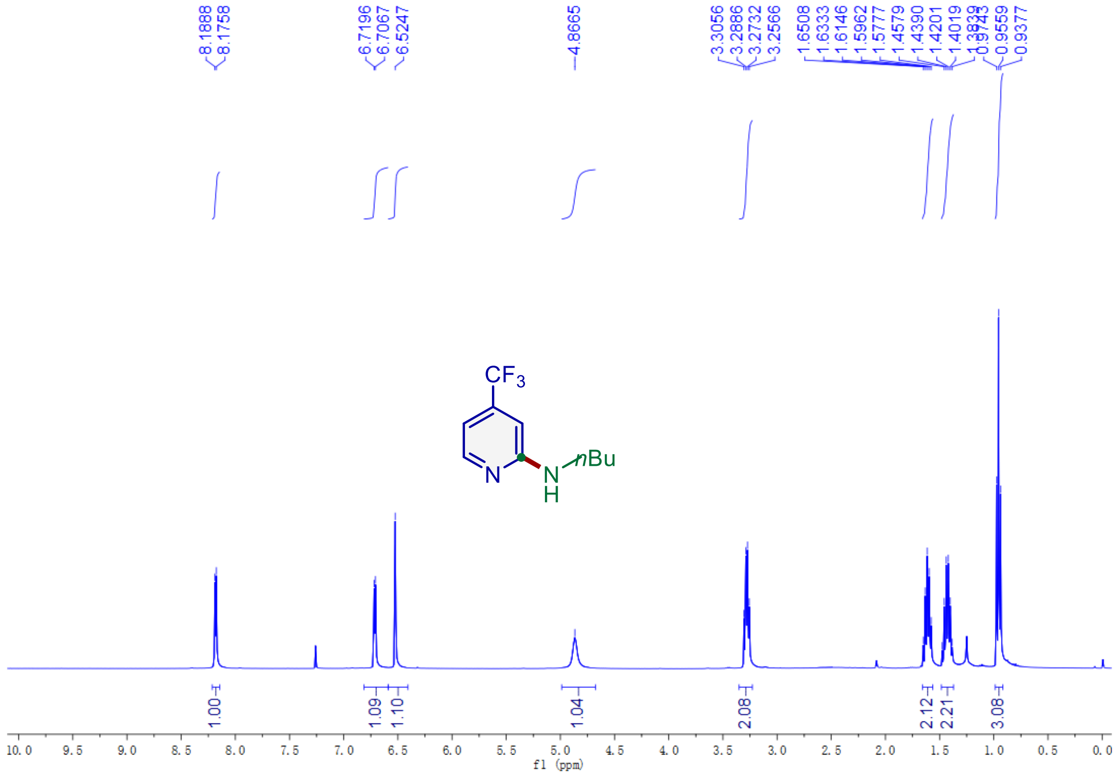

^{1}H NMR (400 MHz, $CDCl_3$) spectrum of compound 47

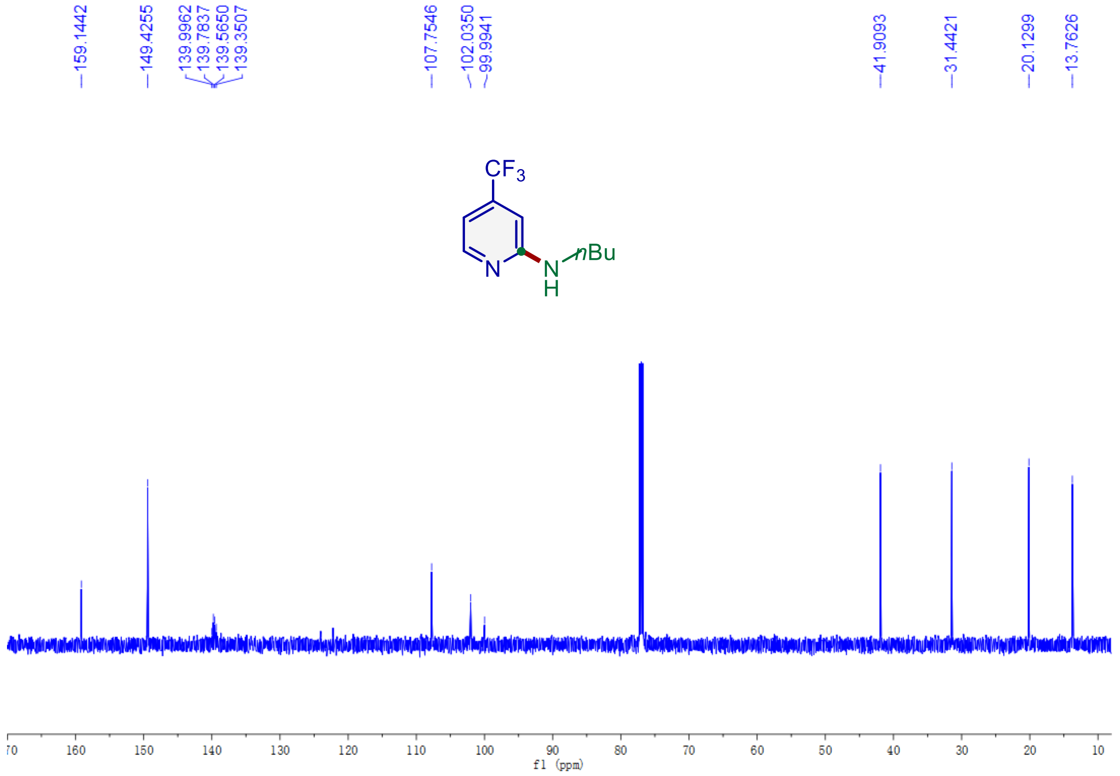

^{13}C NMR (100 MHz, $CDCl_3$) spectrum of compound 47

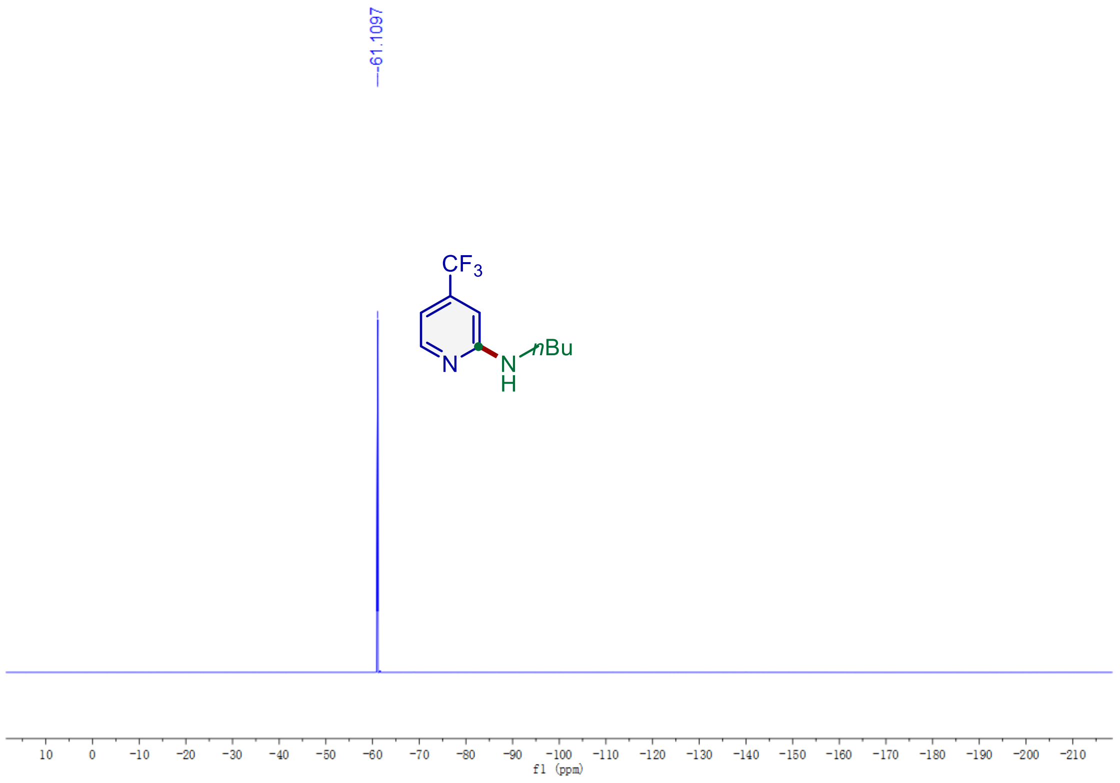

^{19}F NMR (376 MHz, $CDCl_3$) spectrum of compound 47

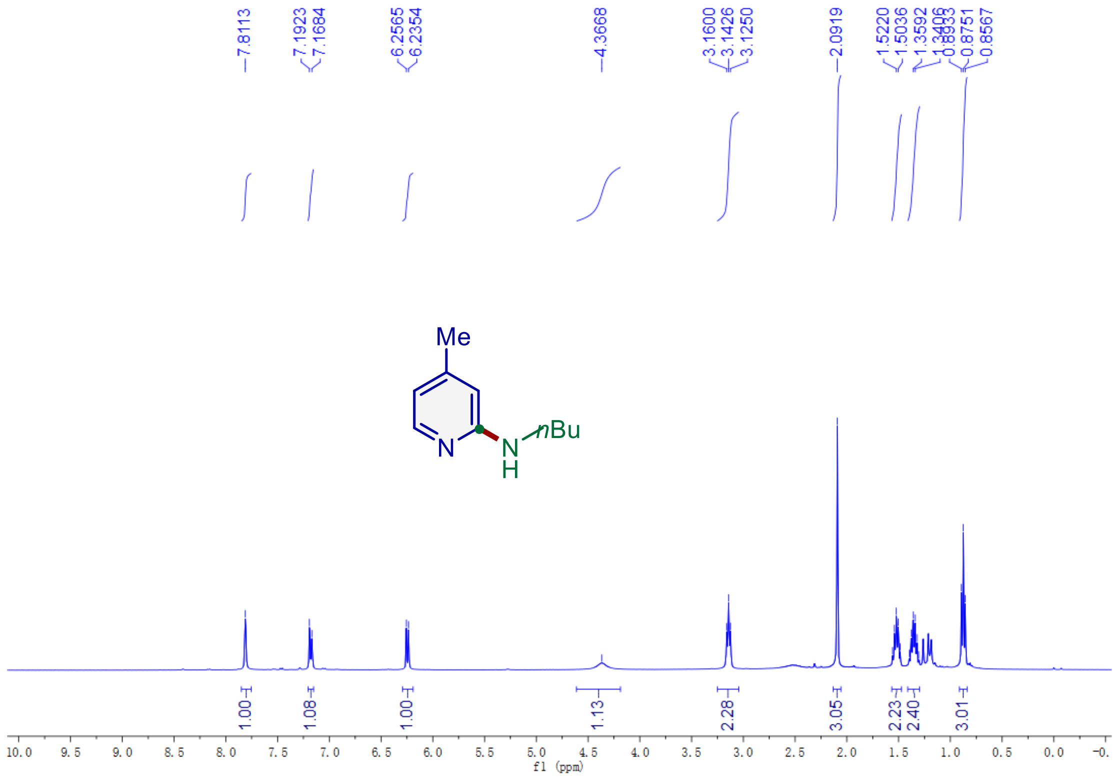

^{1}H NMR (400 MHz, $CDCl_3$) spectrum of compound 48

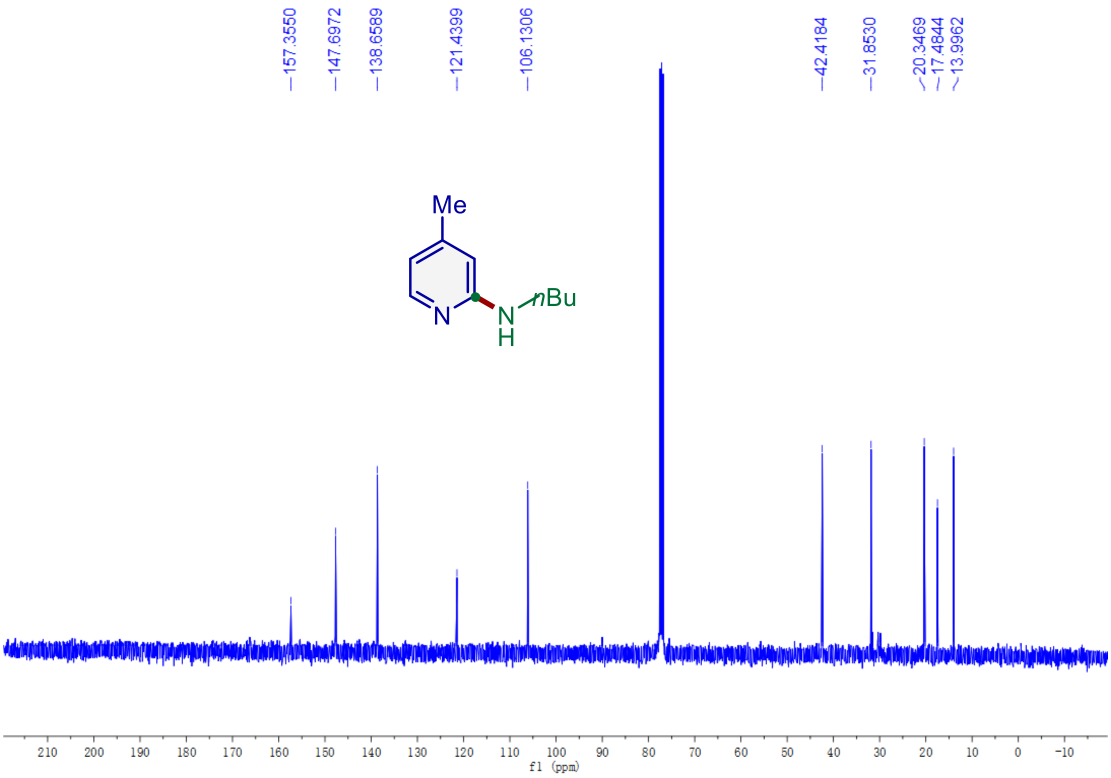

^{13}C NMR (100 MHz, $CDCl_3$) spectrum of compound 48

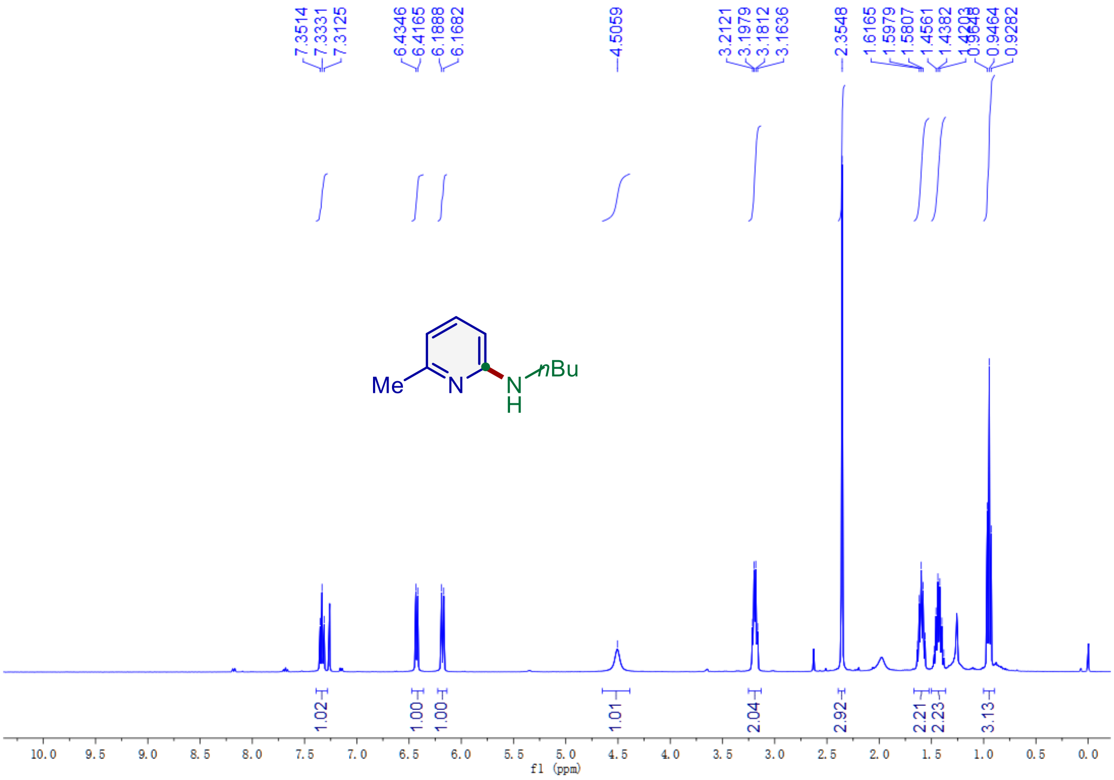

^{1}H NMR (400 MHz, $CDCl_3$) spectrum of compound 49

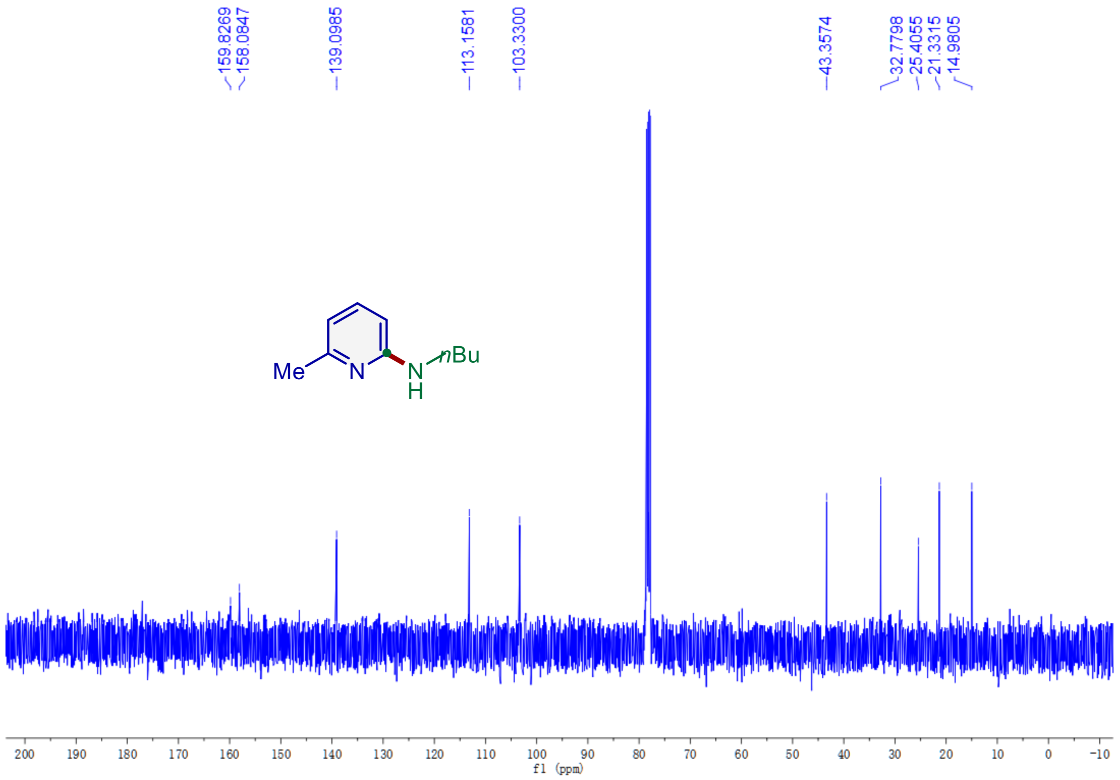

^{13}C NMR (100 MHz, $CDCl_3$) spectrum of compound 49

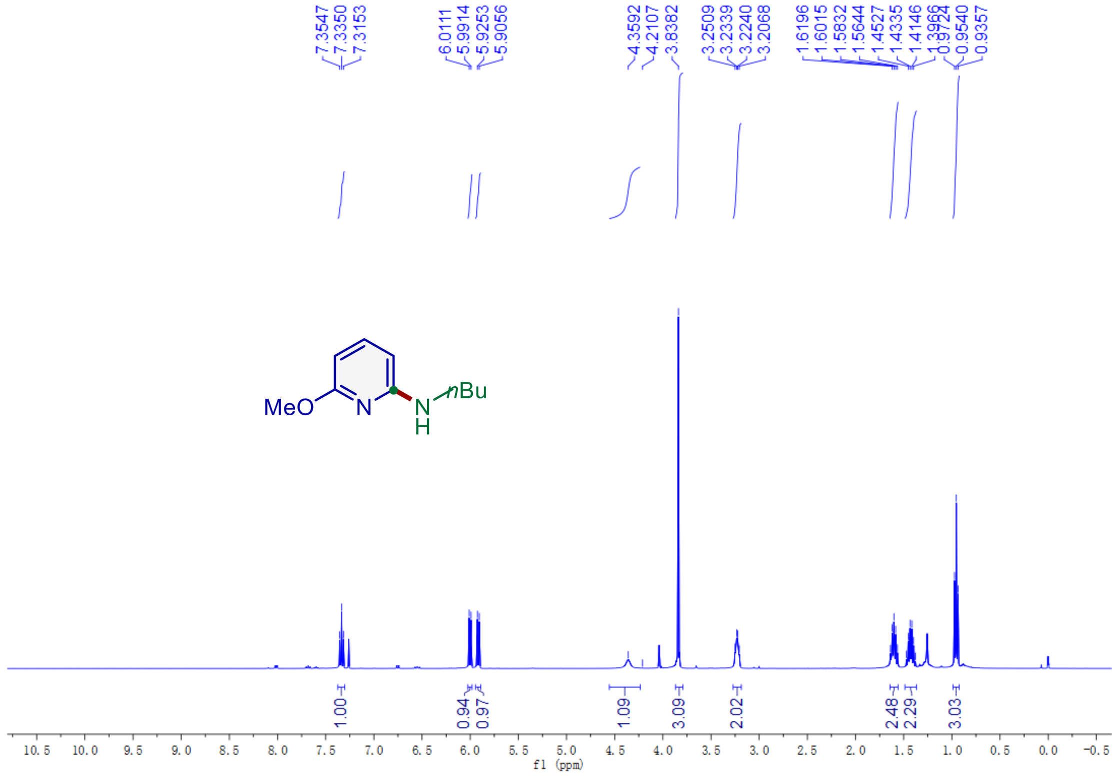

^1H NMR (400 MHz, $CDCl_3$) spectrum of compound 50

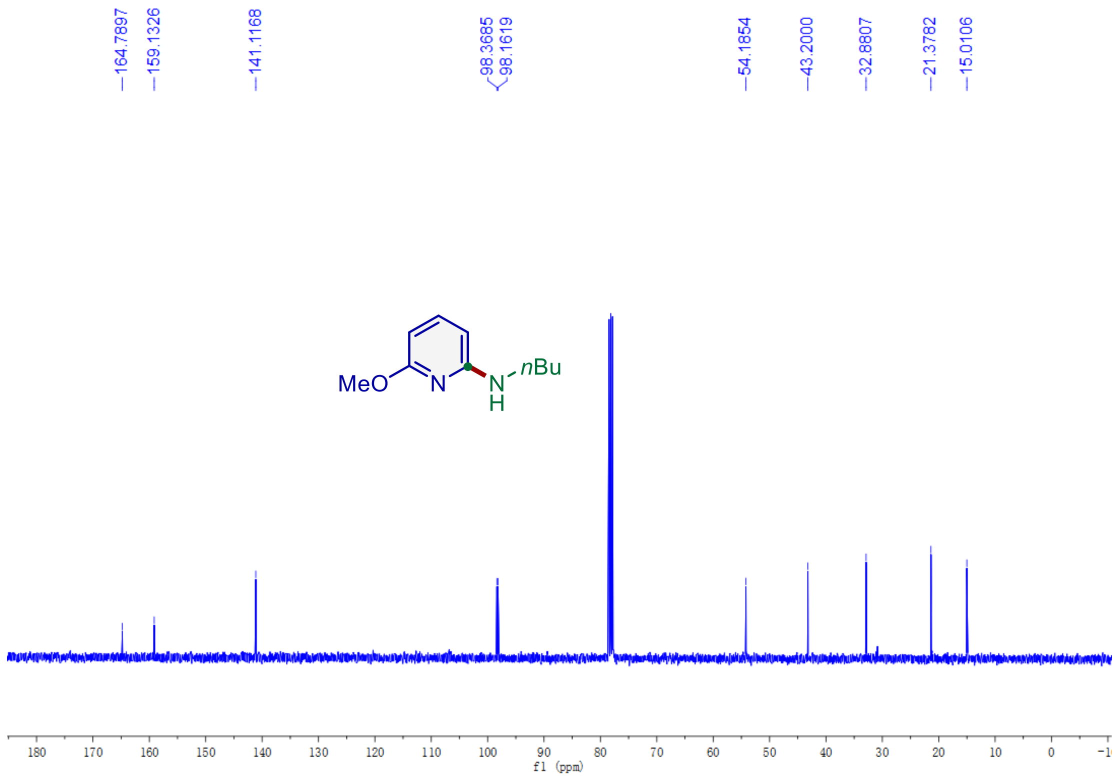

^{13}C NMR (100 MHz, $CDCl_3$) spectrum of compound 50

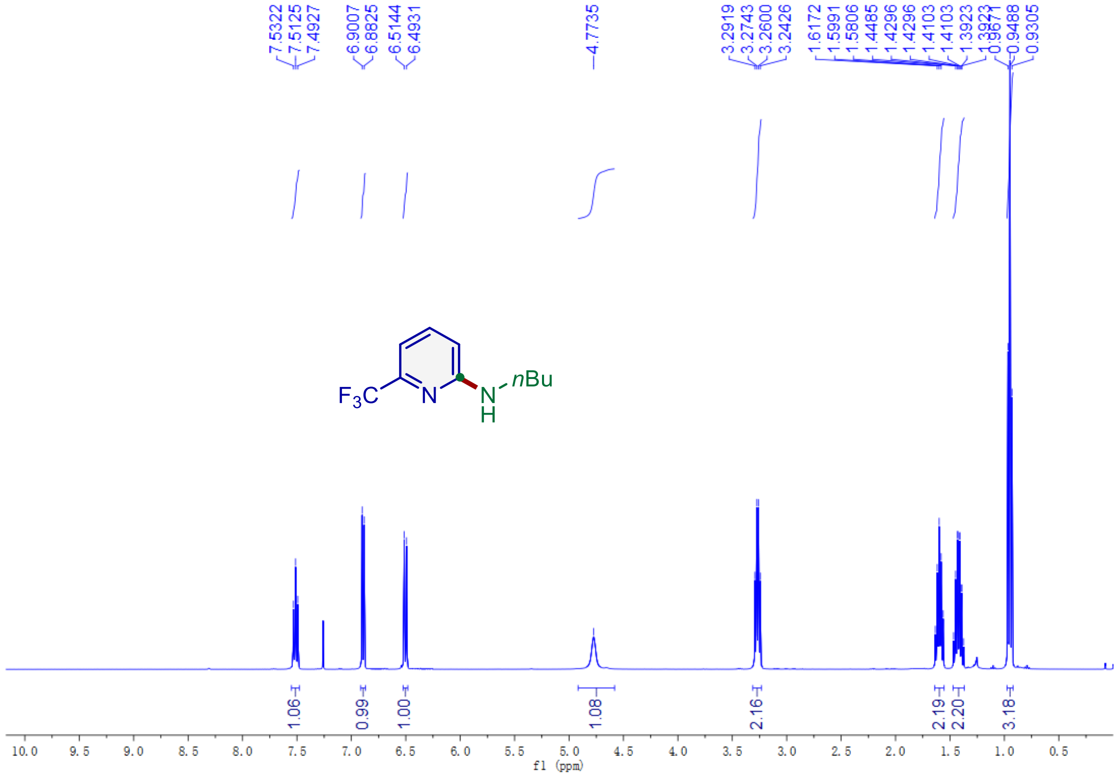

^{1}H NMR (400 MHz, $CDCl_3$) spectrum of compound 51

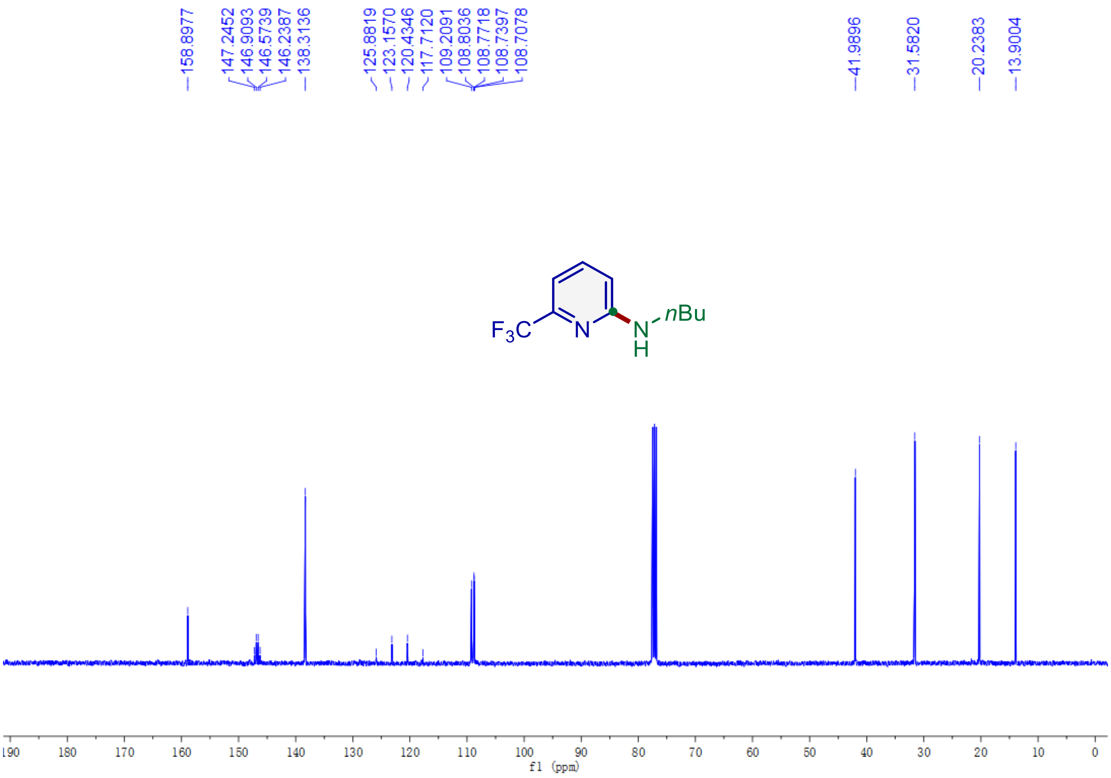

^{13}C NMR (100 MHz, $CDCl_3$) spectrum of compound 51

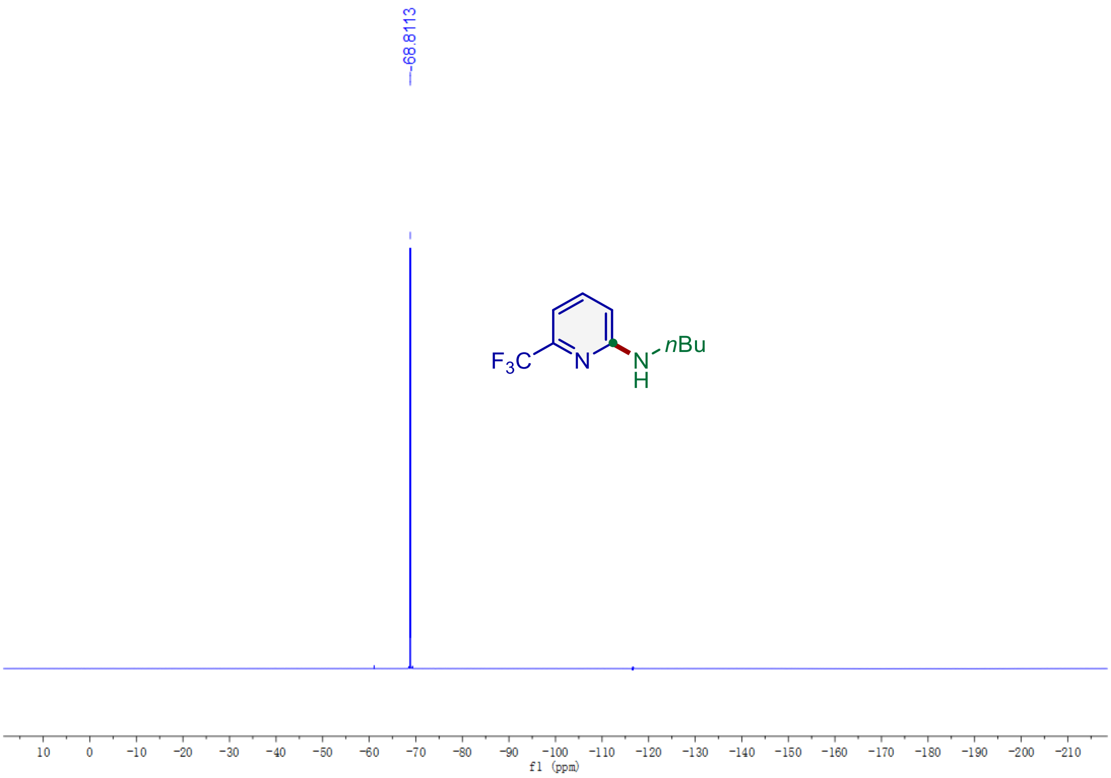

^{19}F NMR (376 MHz, $CDCl_3$) spectrum of compound 51

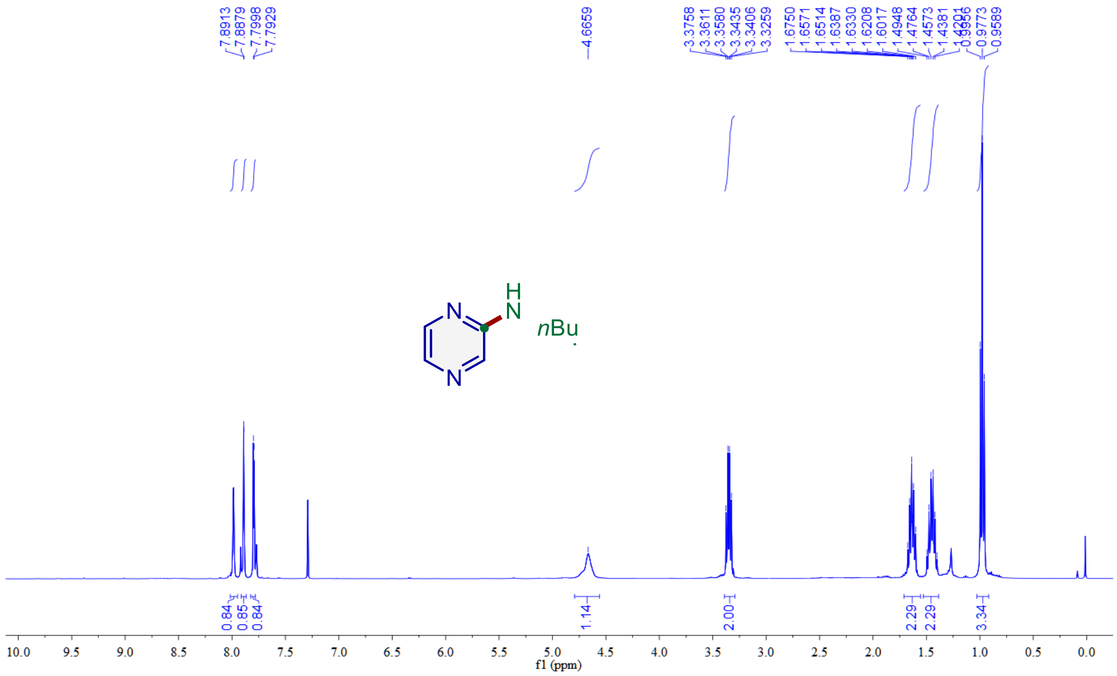

^{1}H NMR (400 MHz, $CDCl_3$) spectrum of compound 52

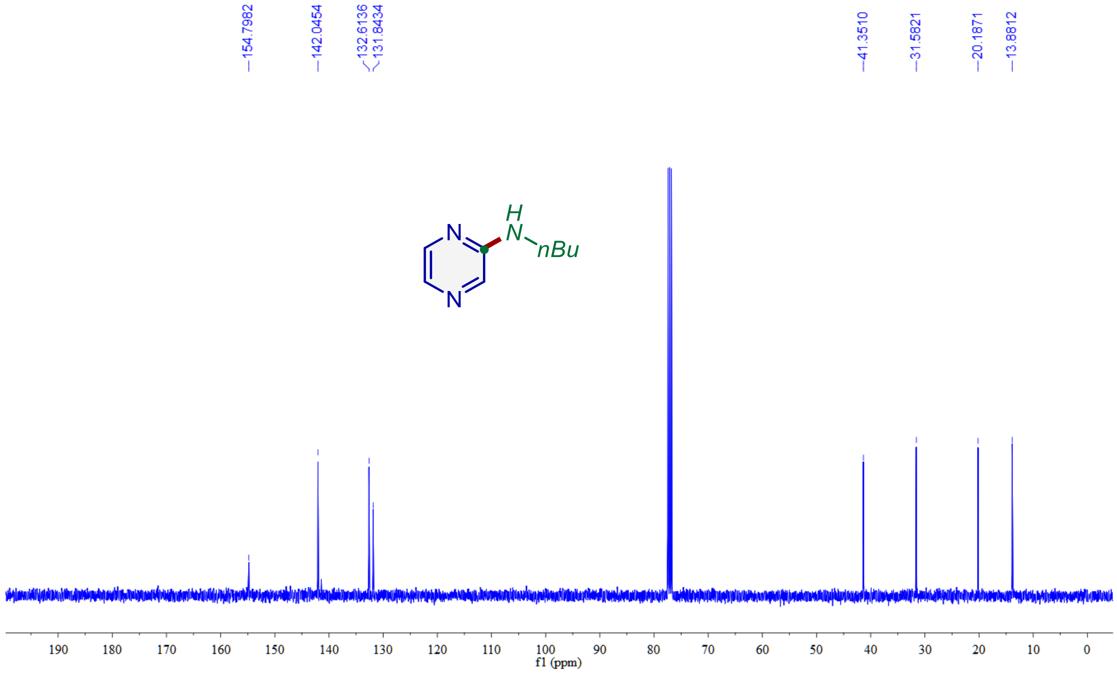

^{13}C NMR (100 MHz, $CDCl_3$) spectrum of compound 52

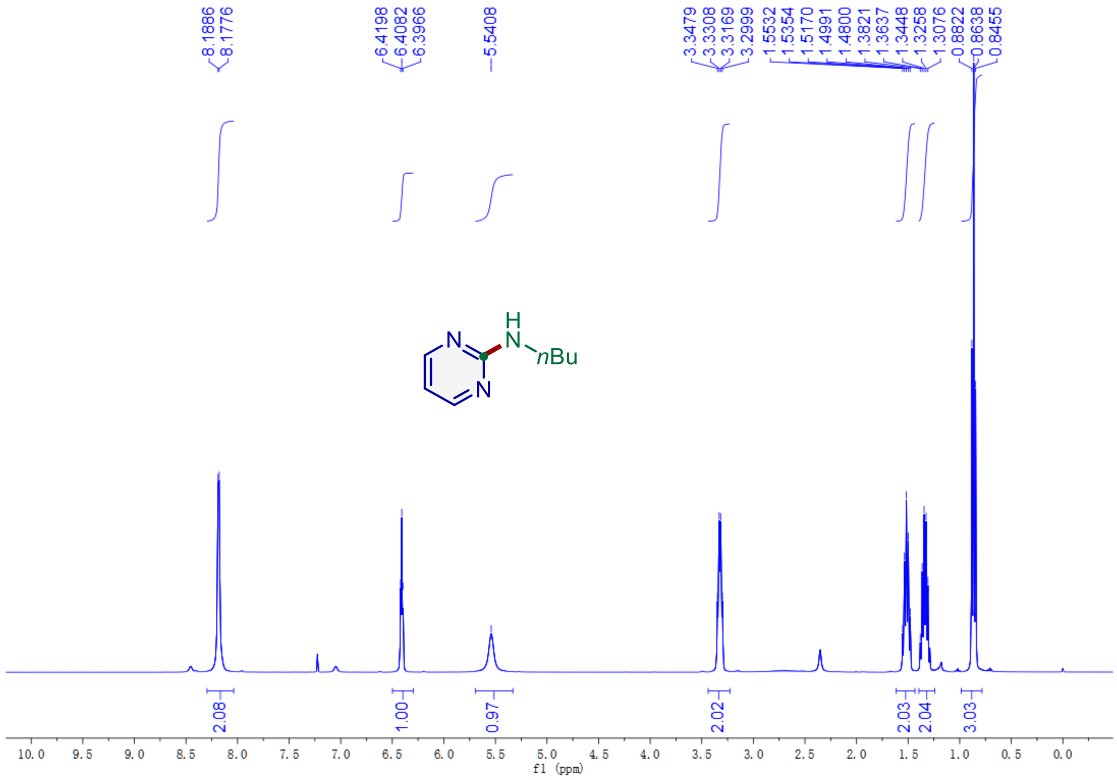

^{1}H NMR (400 MHz, $CDCl_3$) spectrum of compound 53

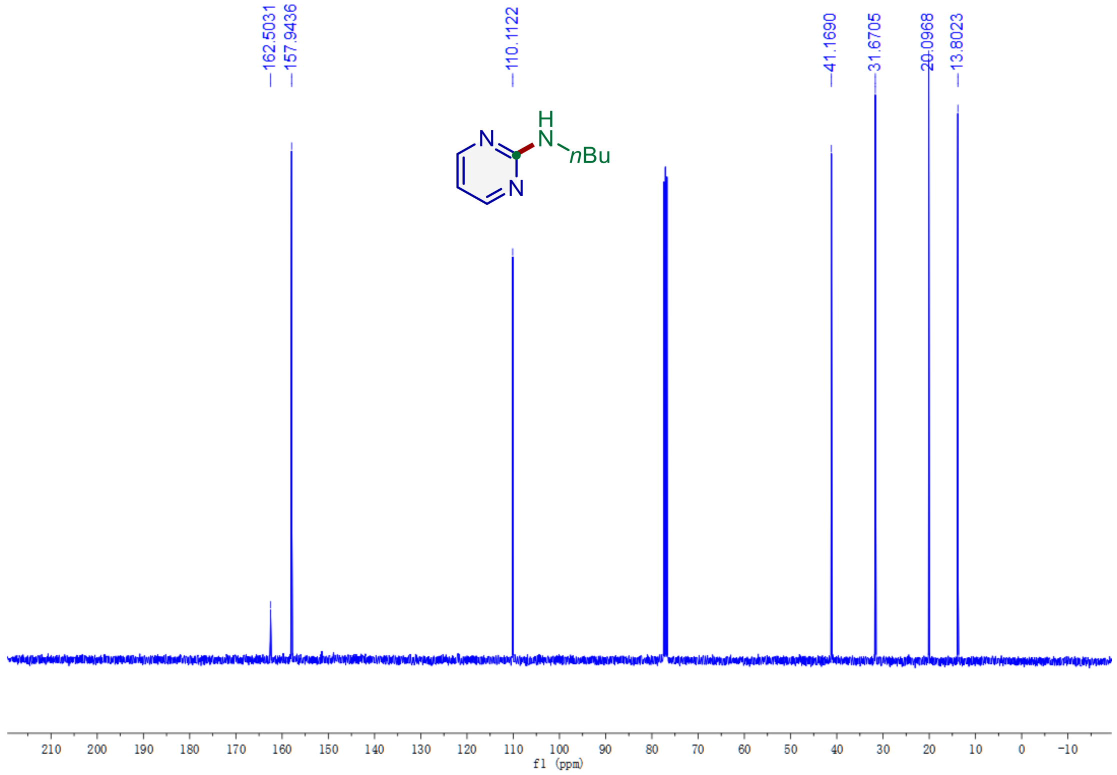

^{13}C NMR (100 MHz, $CDCl_3$) spectrum of compound 53

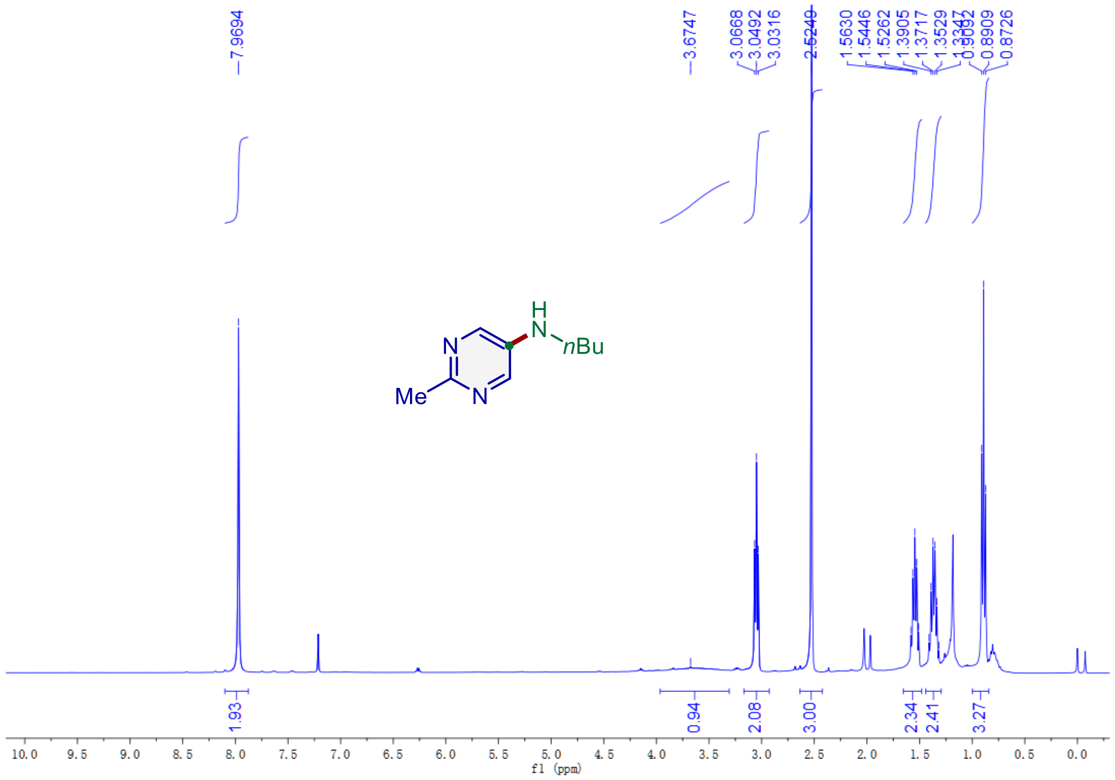

^{1}H NMR (400 MHz, $CDCl_3$) spectrum of compound 54

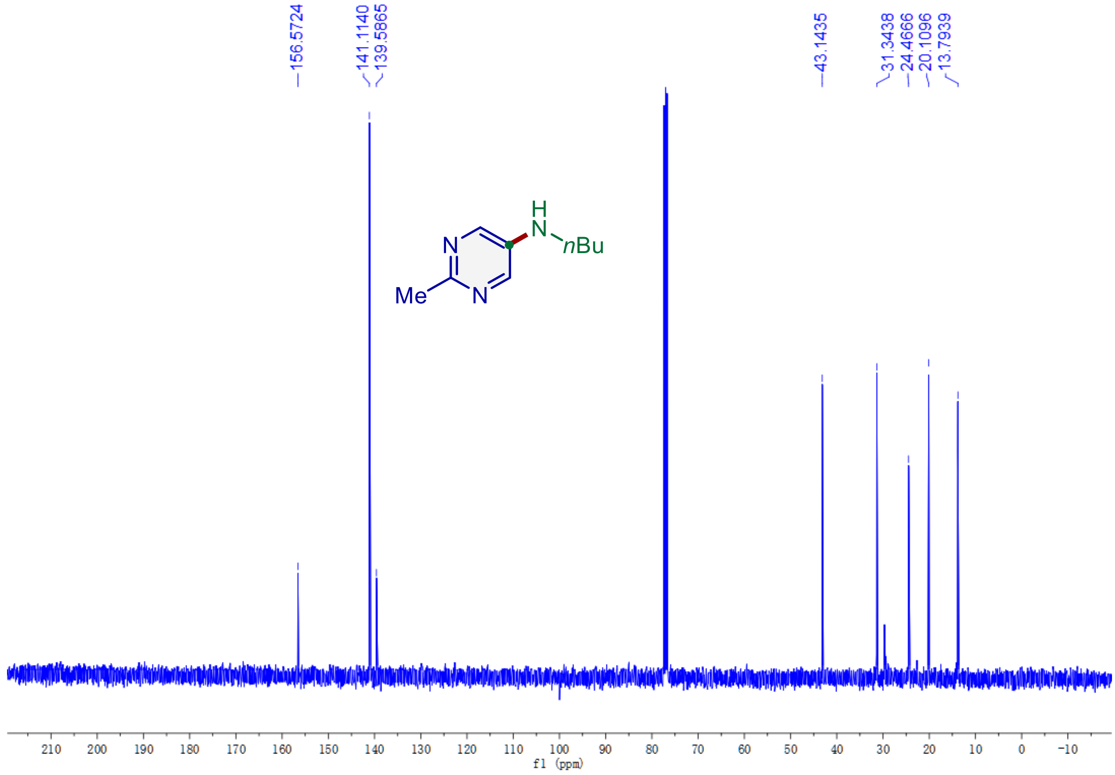

^{13}C NMR (100 MHz, $CDCl_3$) spectrum of compound 54

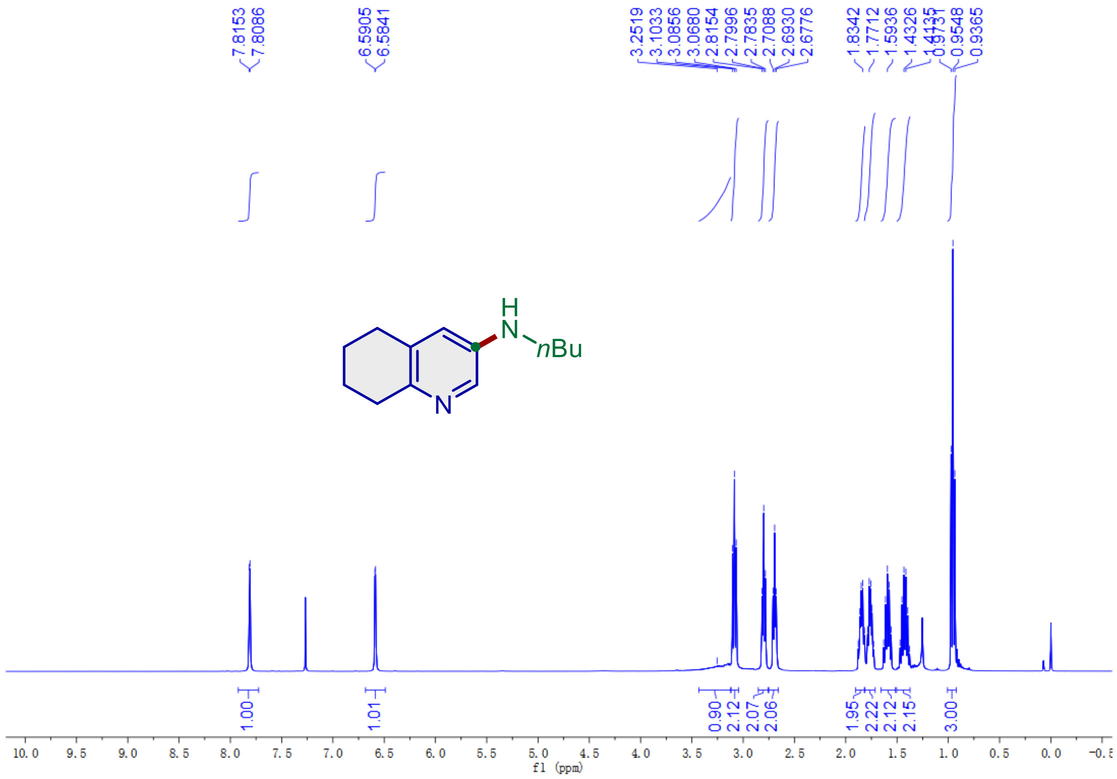

^{1}H NMR (400 MHz, $CDCl_3$) spectrum of compound 55

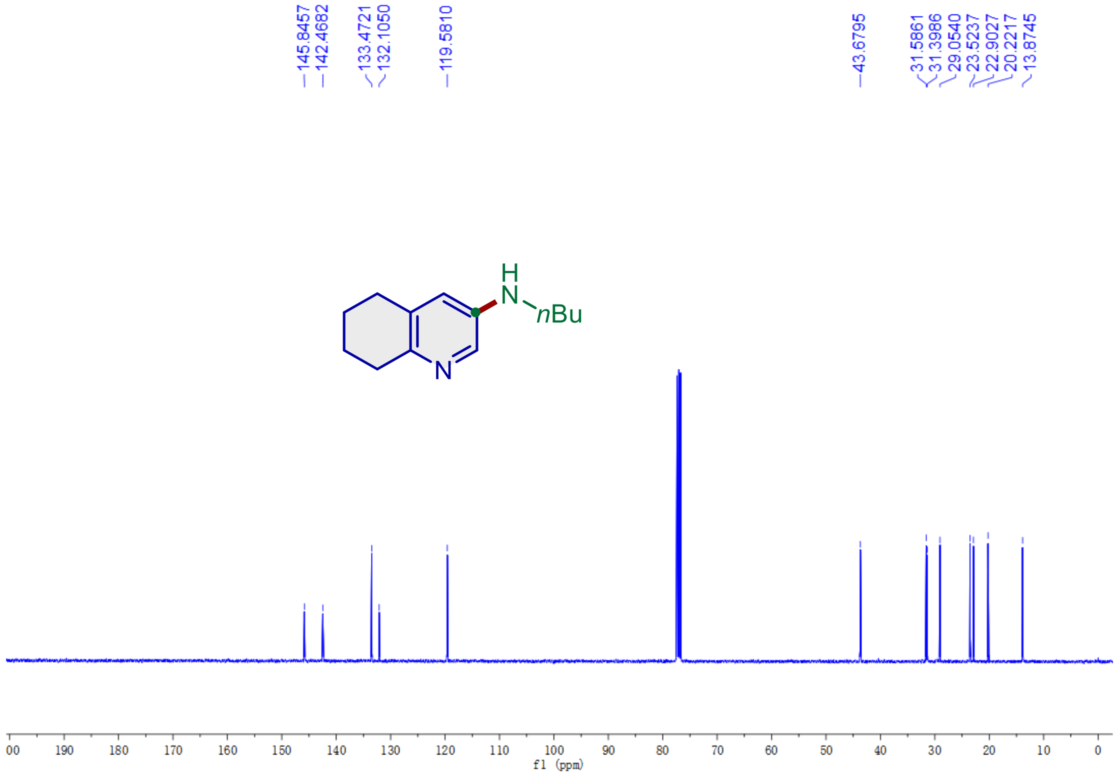

^{13}C NMR (100 MHz, $CDCl_3$) spectrum of compound 55

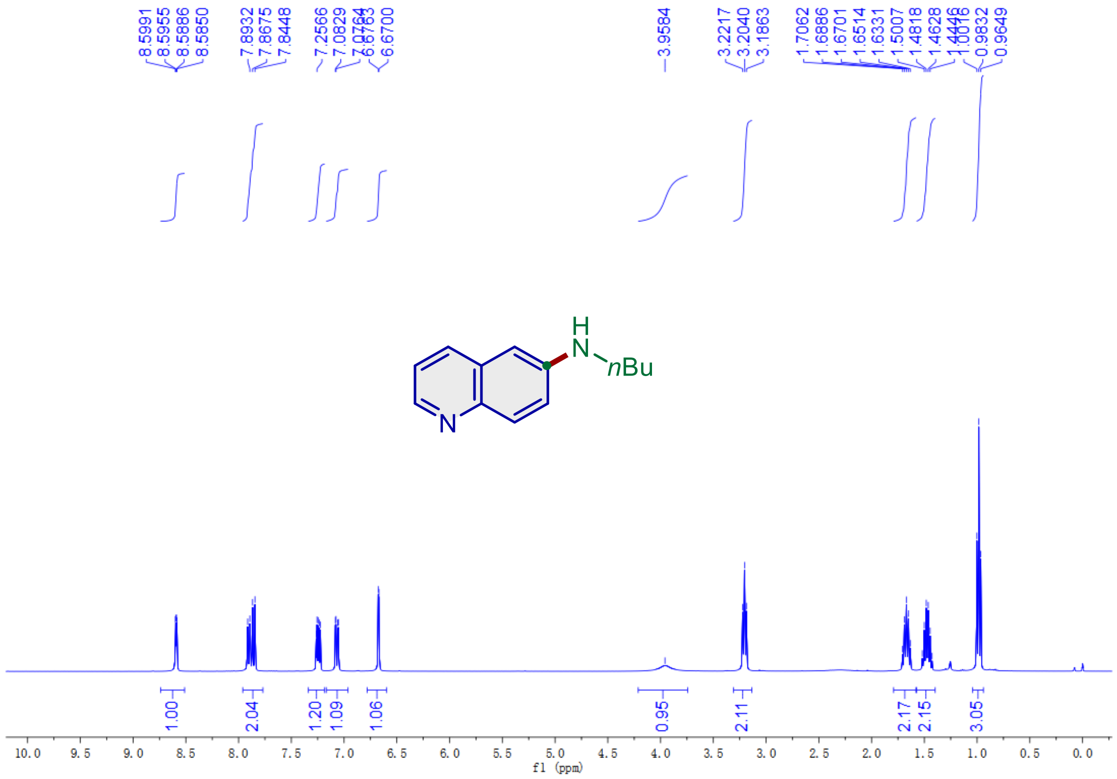

^{1}H NMR (400 MHz, $CDCl_3$) spectrum of compound 56

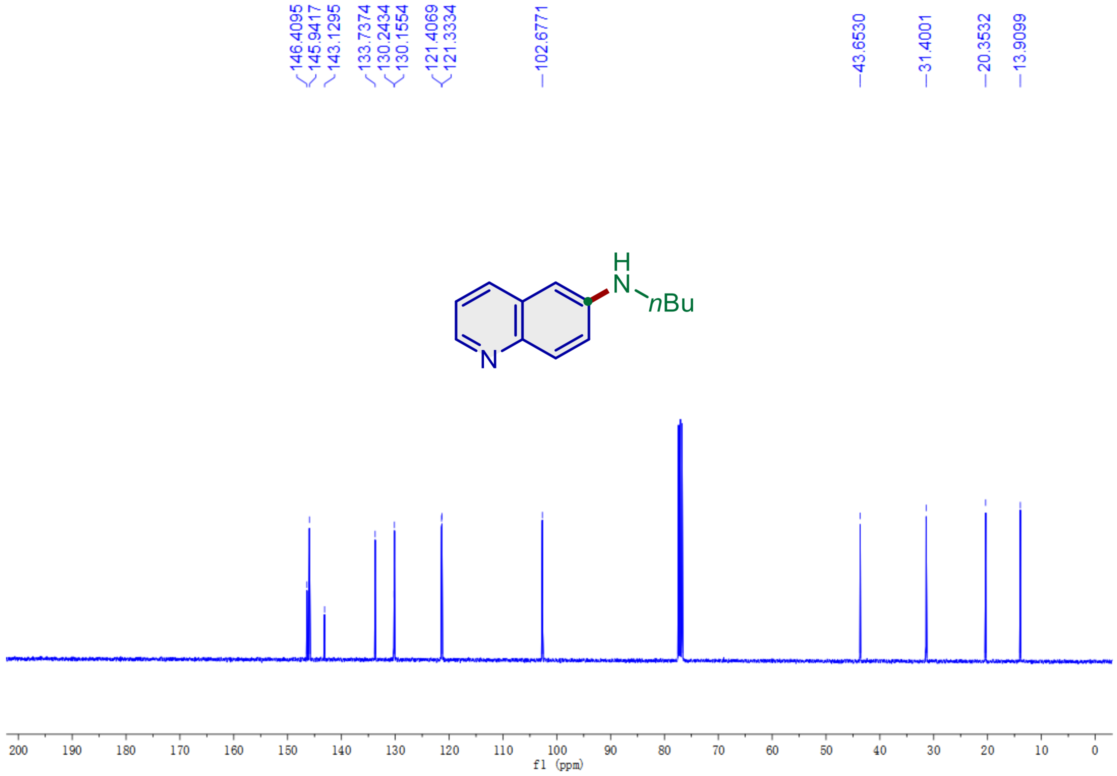

^{13}C NMR (100 MHz, $CDCl_3$) spectrum of compound 56

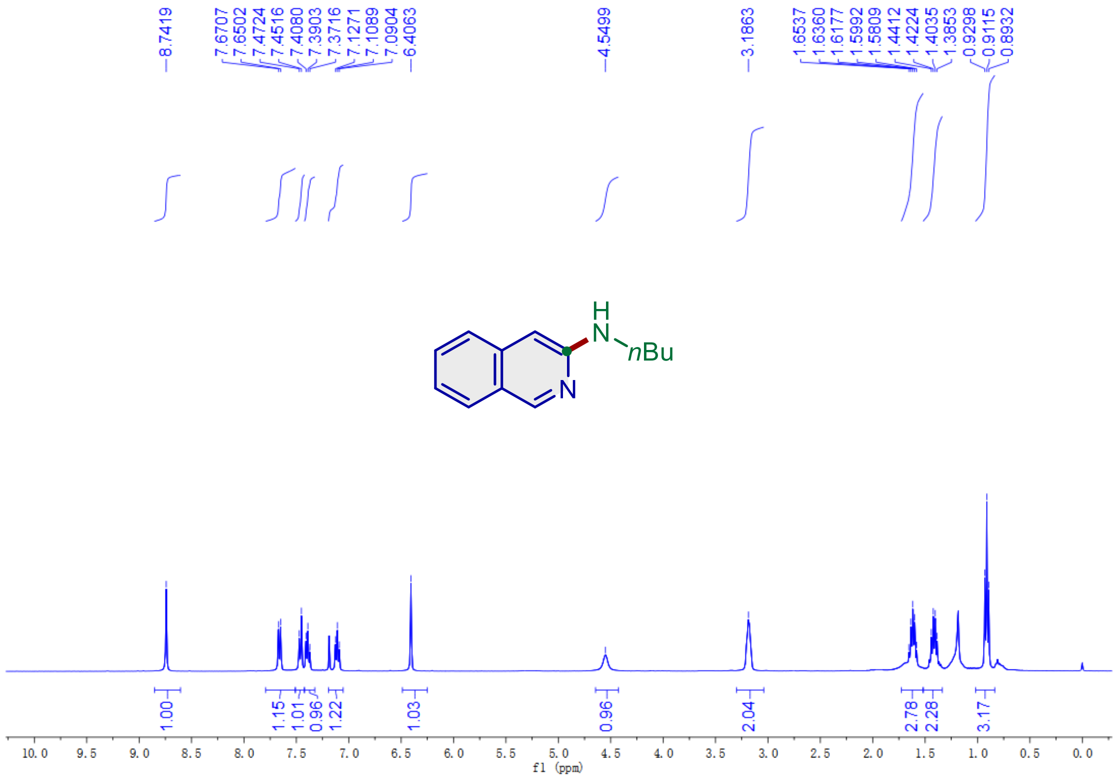

^{1}H NMR (400 MHz, $CDCl_3$) spectrum of compound 57

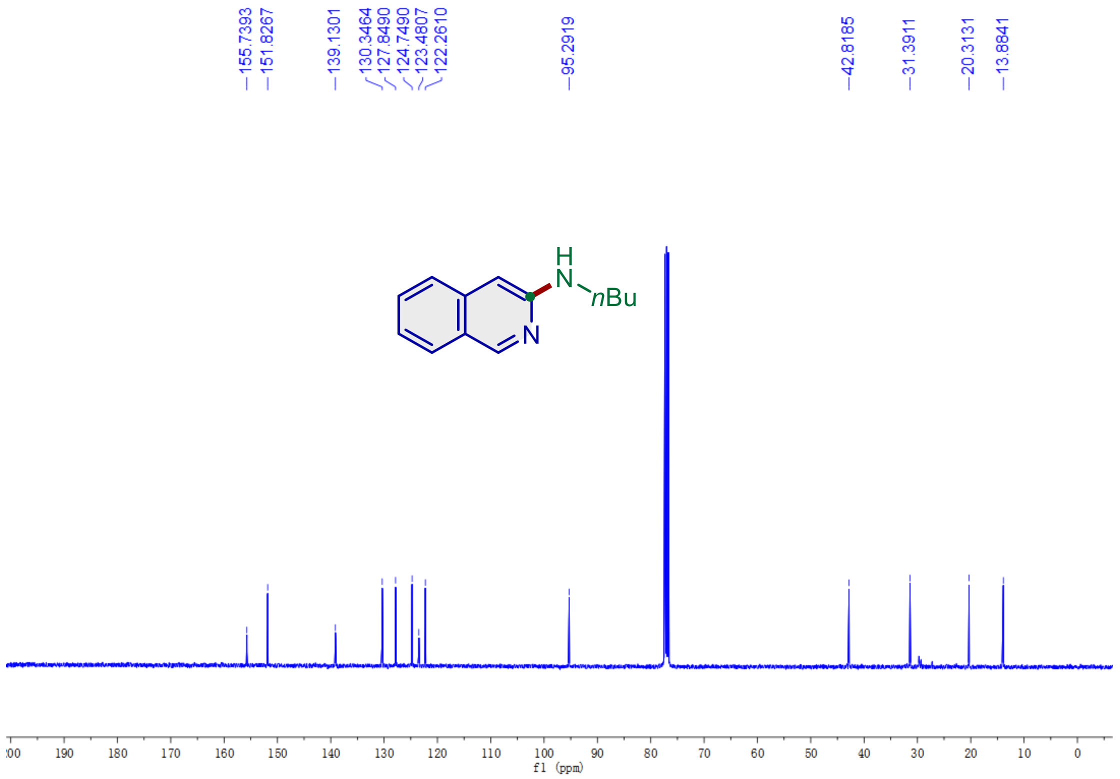

^{13}C NMR (100 MHz, $CDCl_3$) spectrum of compound 57

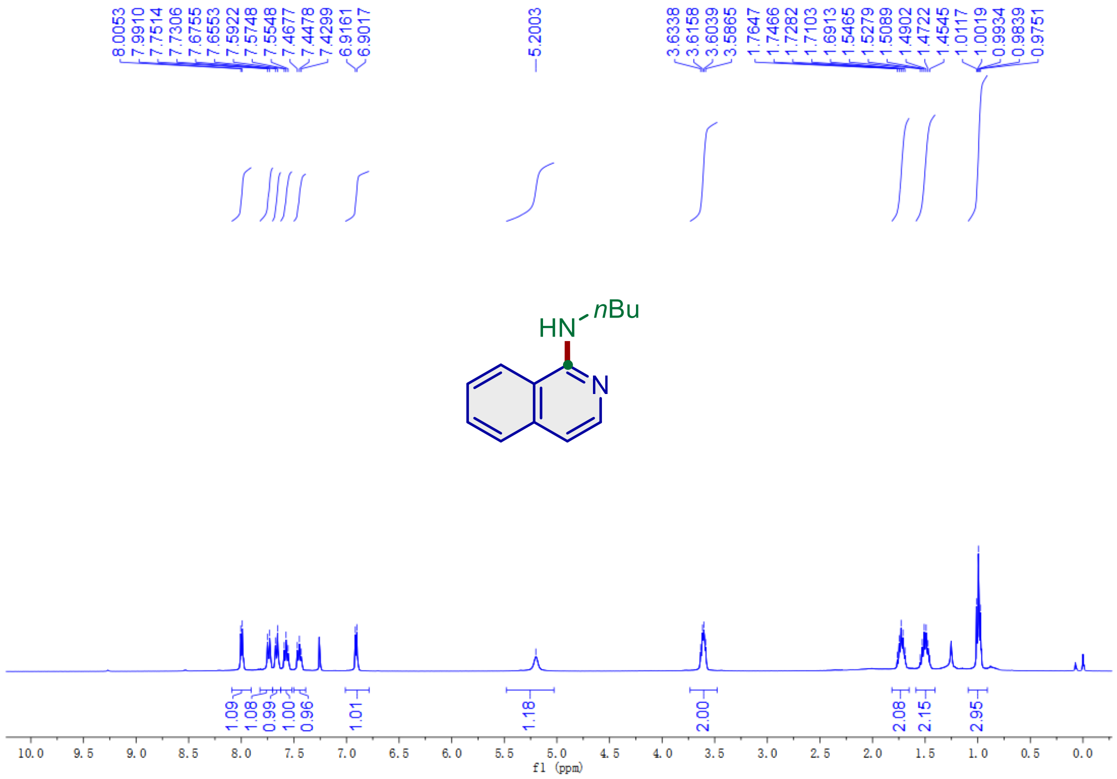

^{1}H NMR (400 MHz, CDCl$_3$) spectrum of compound 58

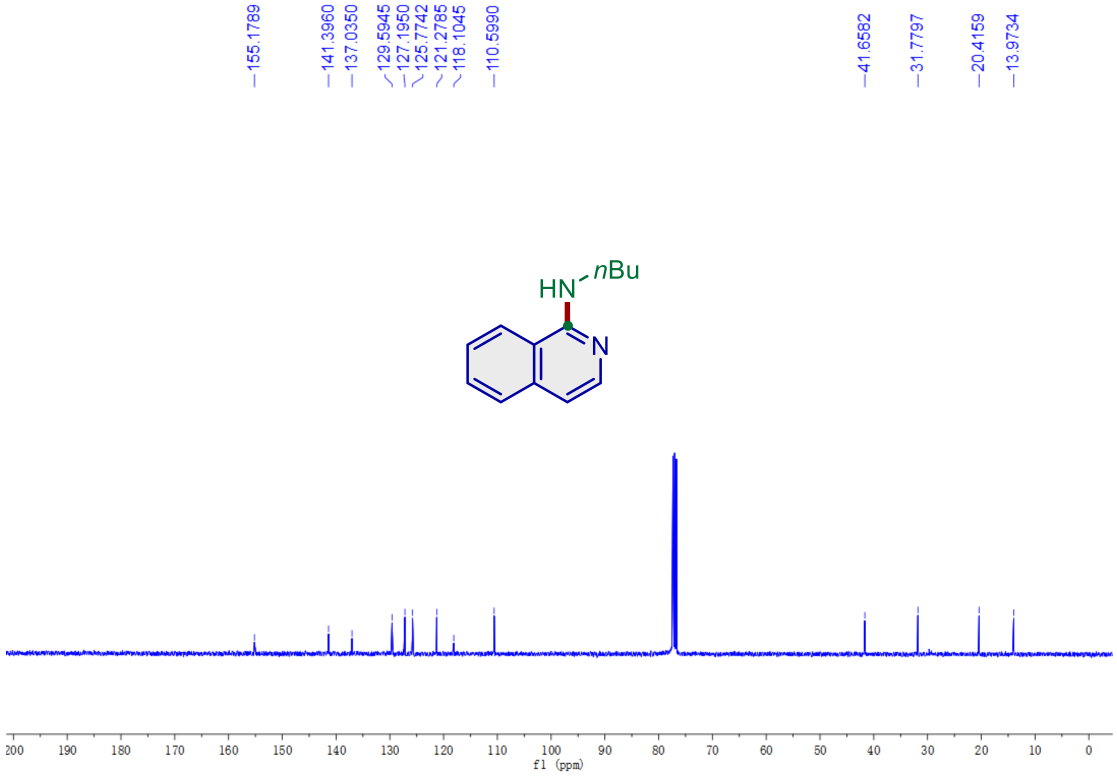

^{13}C NMR (100 MHz, CDCl$_3$) spectrum of compound 58

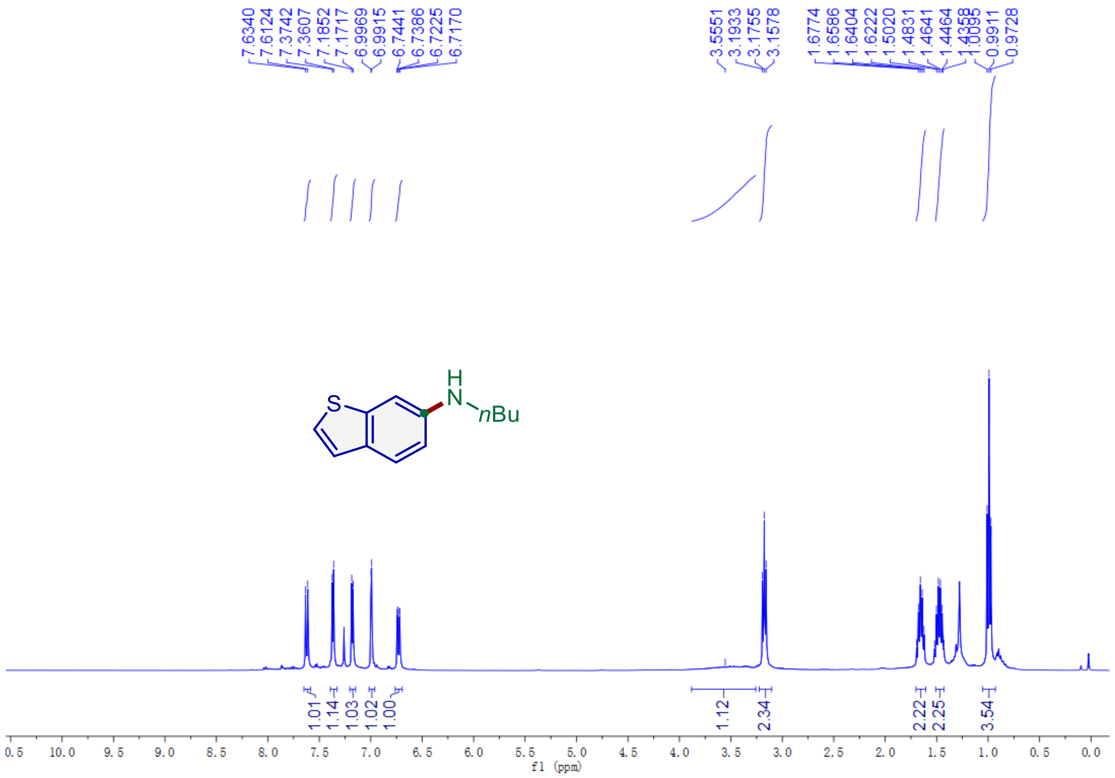

^{1}H NMR (400 MHz, $CDCl_3$) spectrum of compound 59

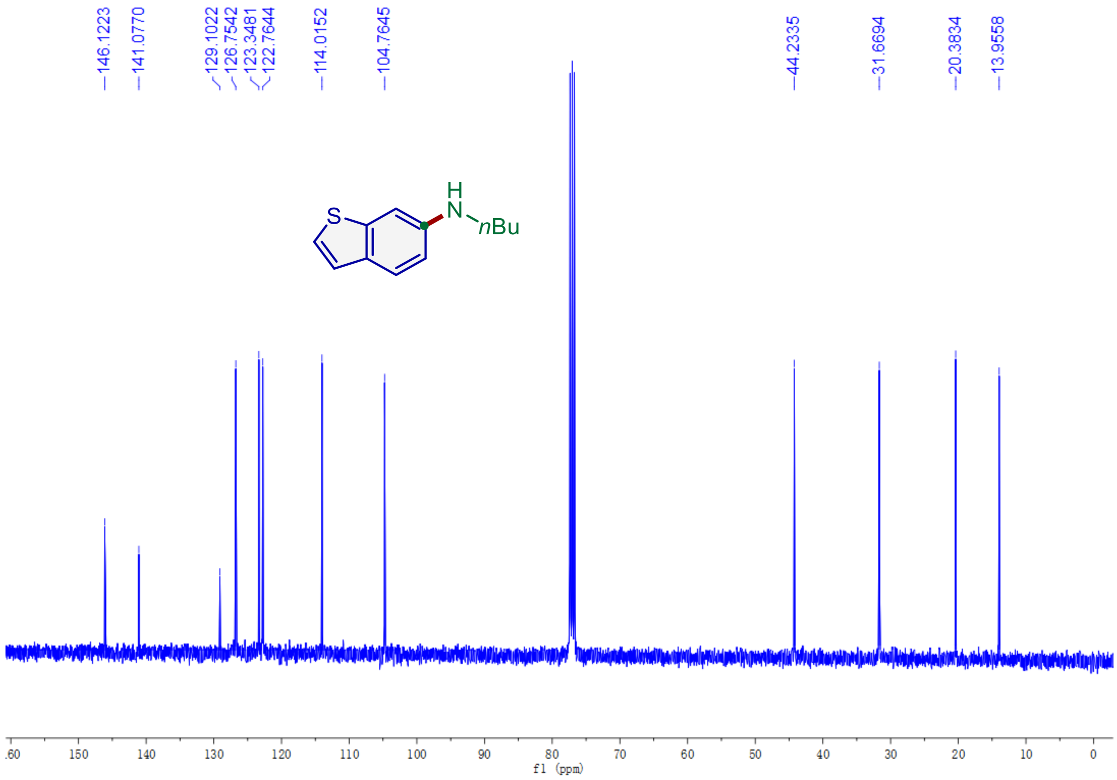

^{13}C NMR (100 MHz, $CDCl_3$) spectrum of compound 59

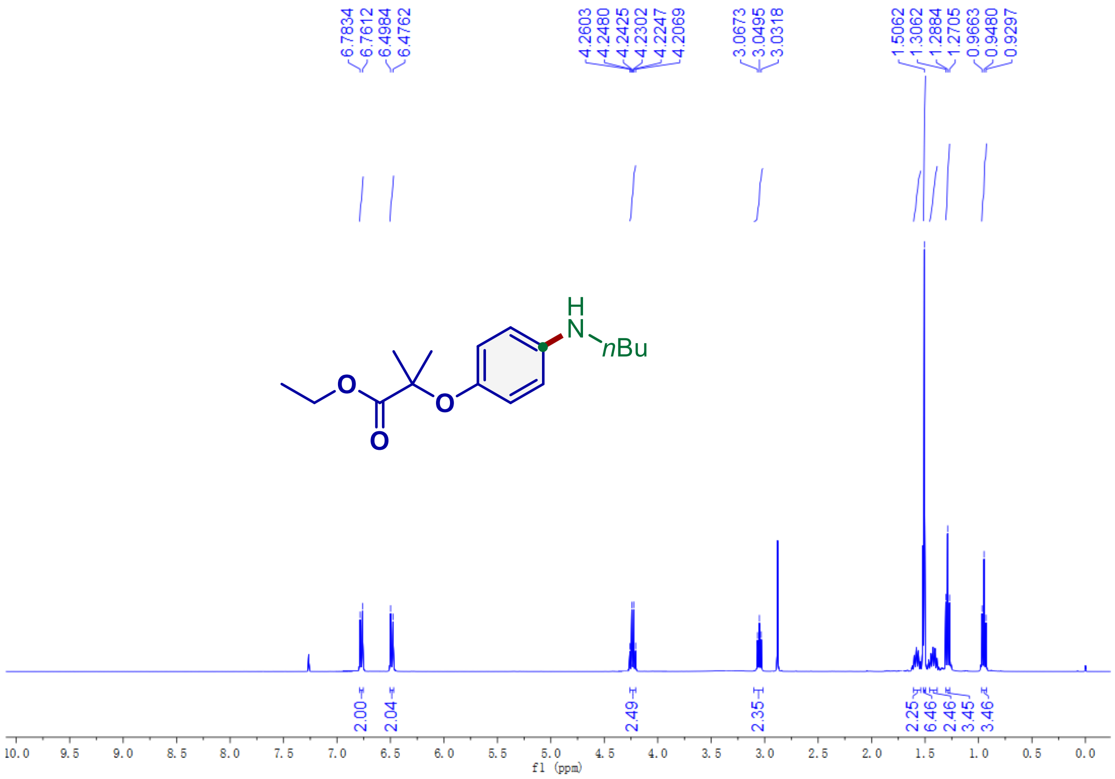

^{1}H NMR (400 MHz, $CDCl_3$) spectrum of compound 60

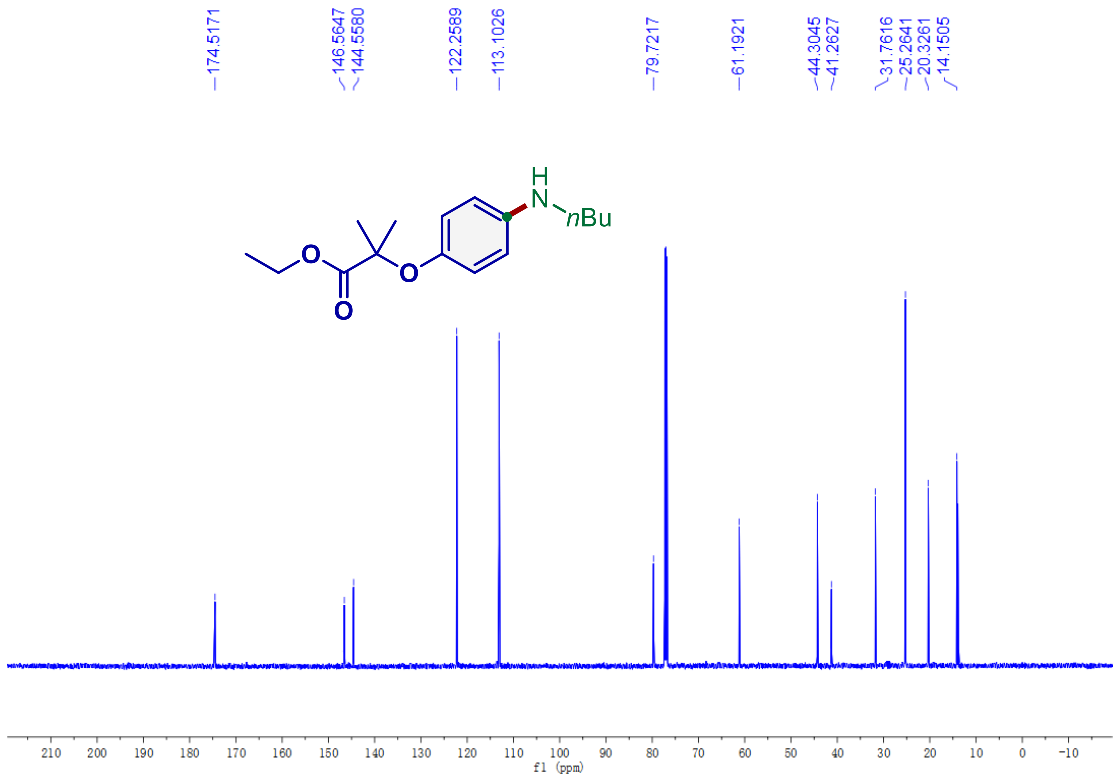

^{13}C NMR (100 MHz, $CDCl_3$) spectrum of compound 60

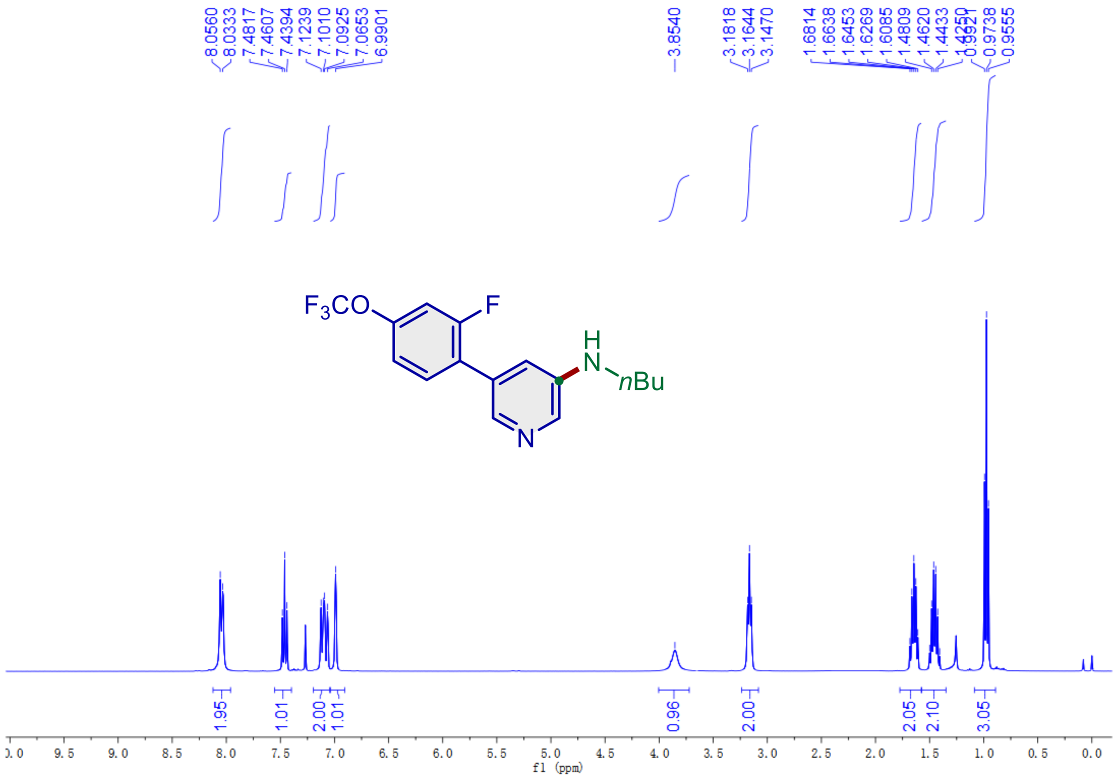

^{1}H NMR (400 MHz, $CDCl_3$) spectrum of compound 61

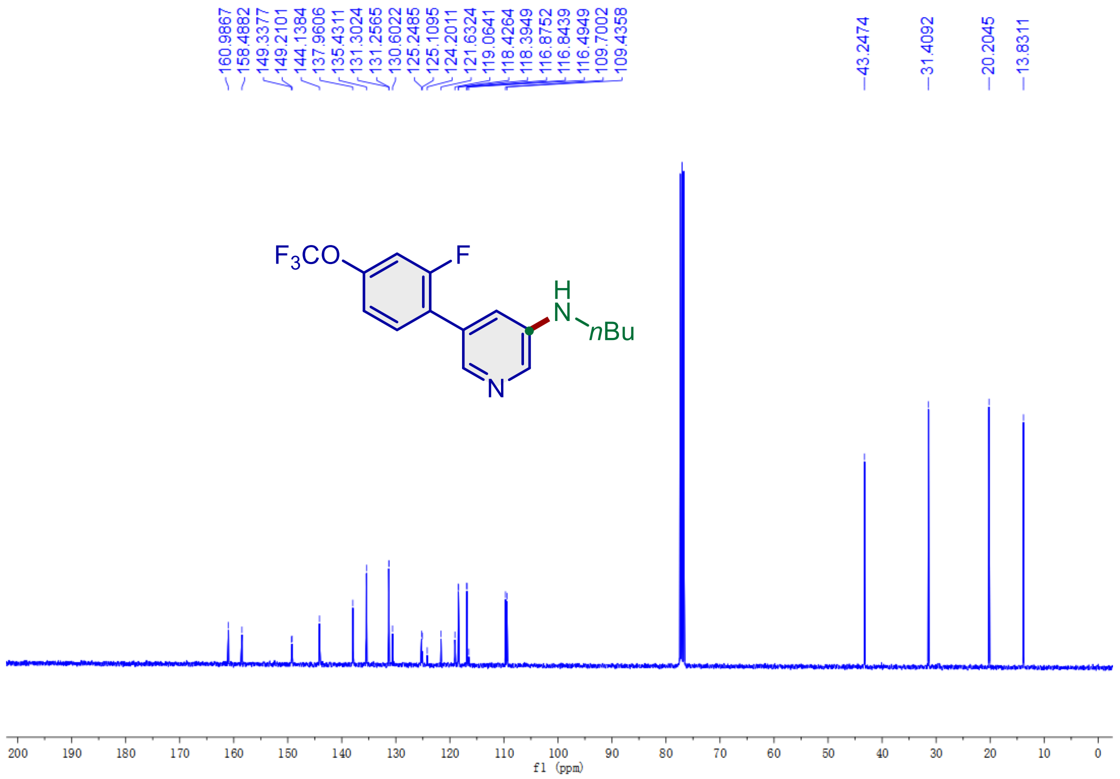

^{13}C NMR (100 MHz, $CDCl_3$) spectrum of compound 61

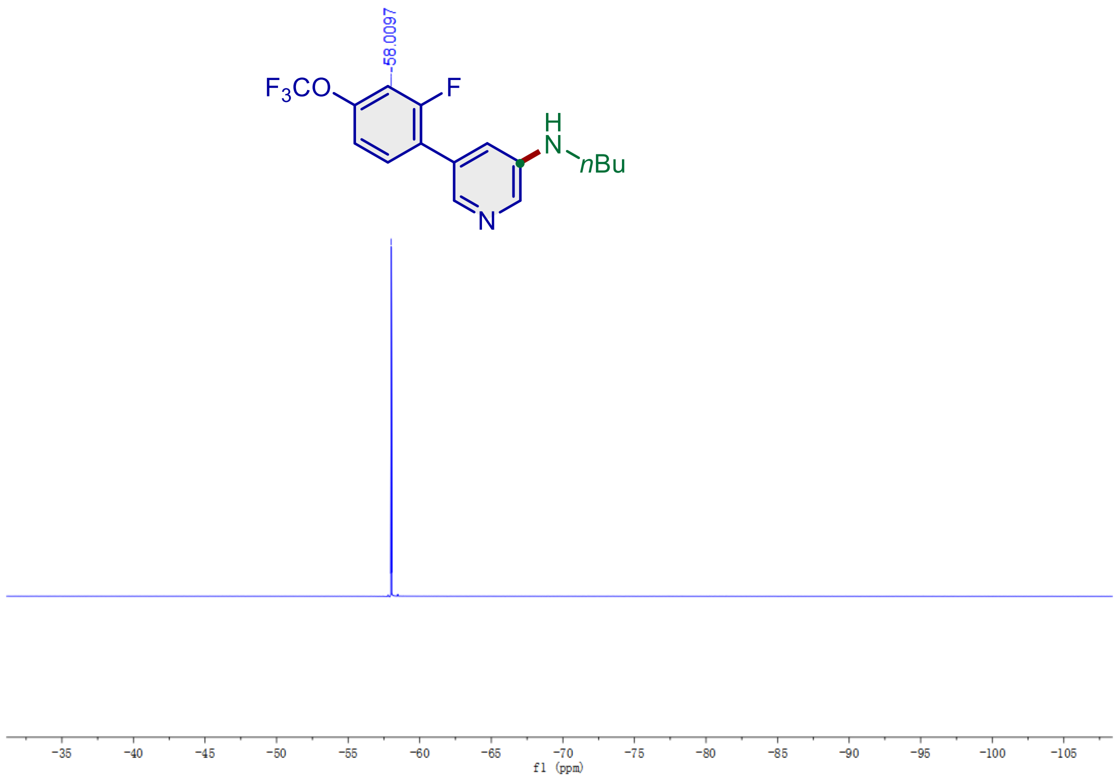

^{19}F NMR (376 MHz, $CDCl_3$) spectrum of compound 61

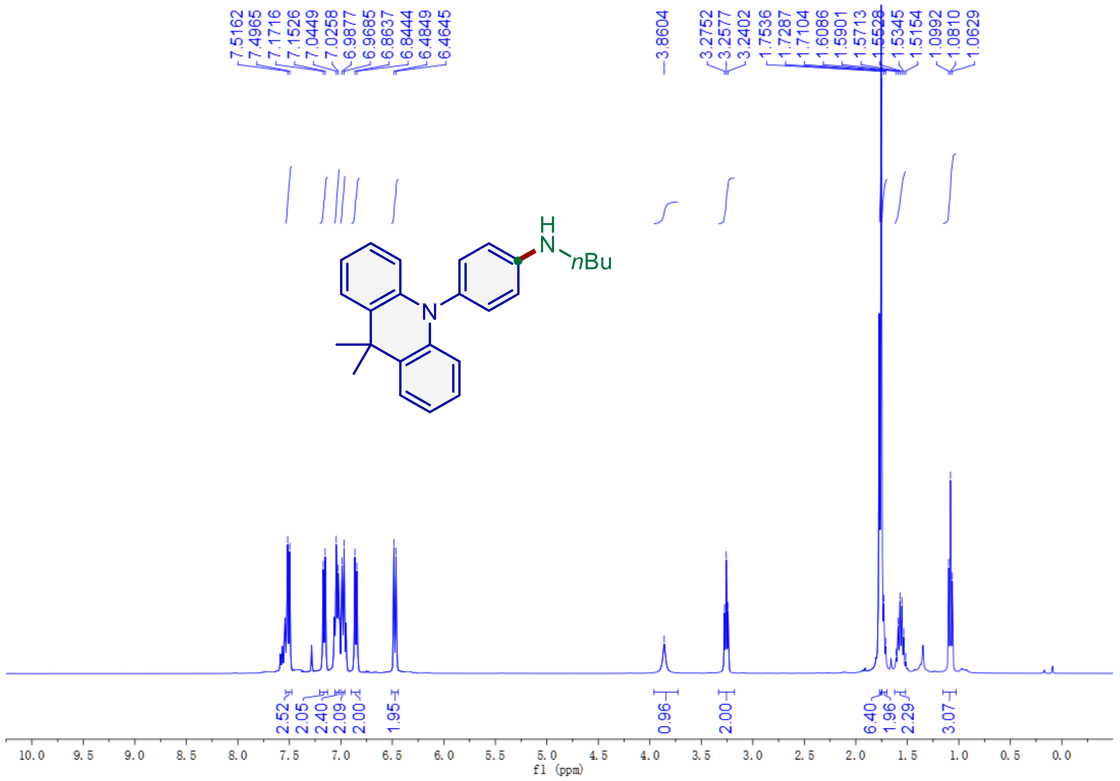

^{1}H NMR (400 MHz, $CDCl_3$) spectrum of compound 62

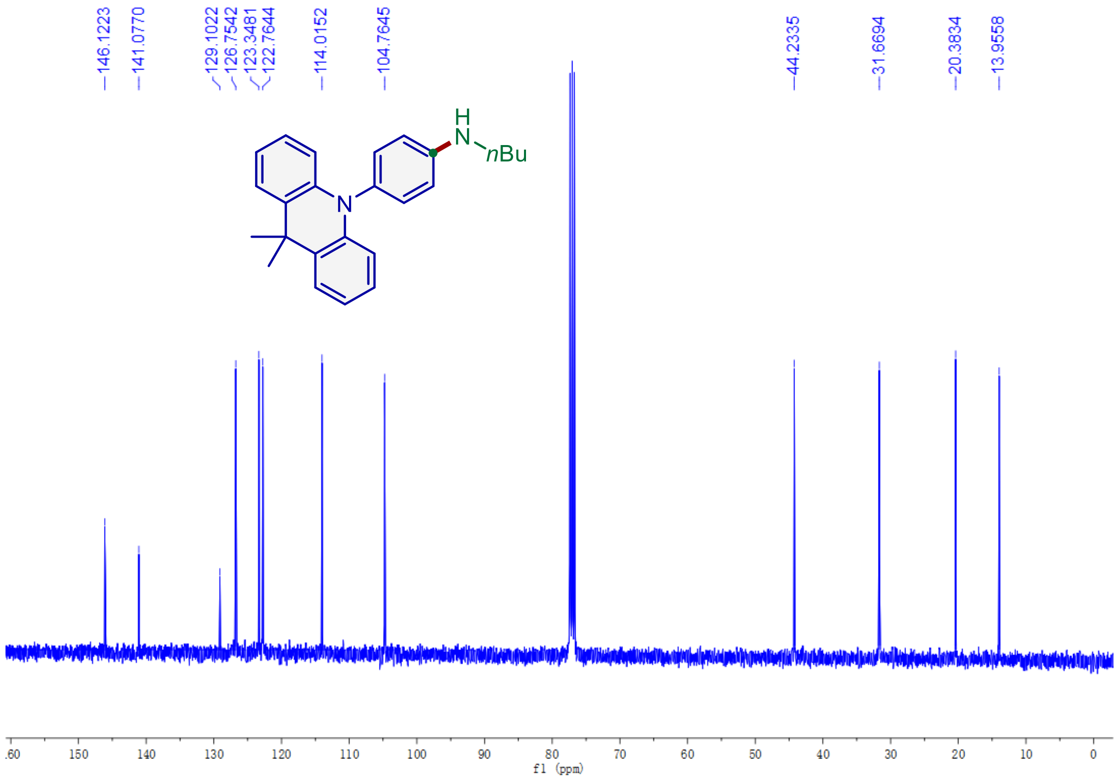

^{13}C NMR (100 MHz, $CDCl_3$) spectrum of compound 62

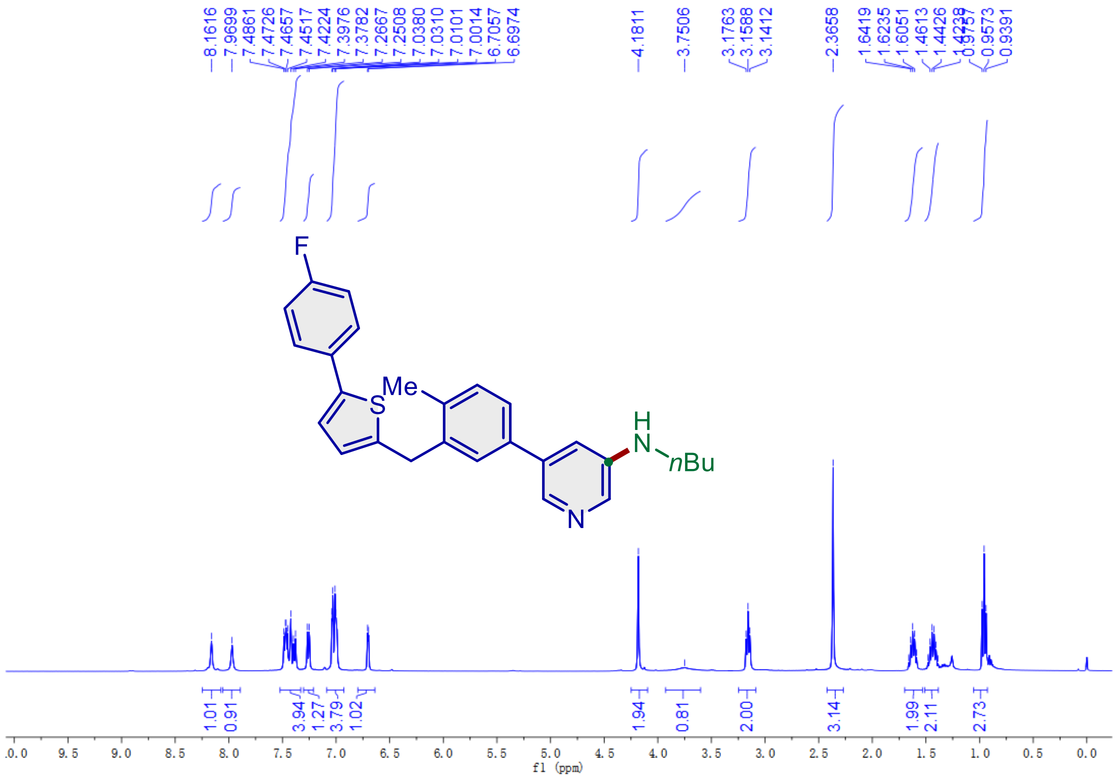

^{1}H NMR (400 MHz, $CDCl_3$) spectrum of compound 63

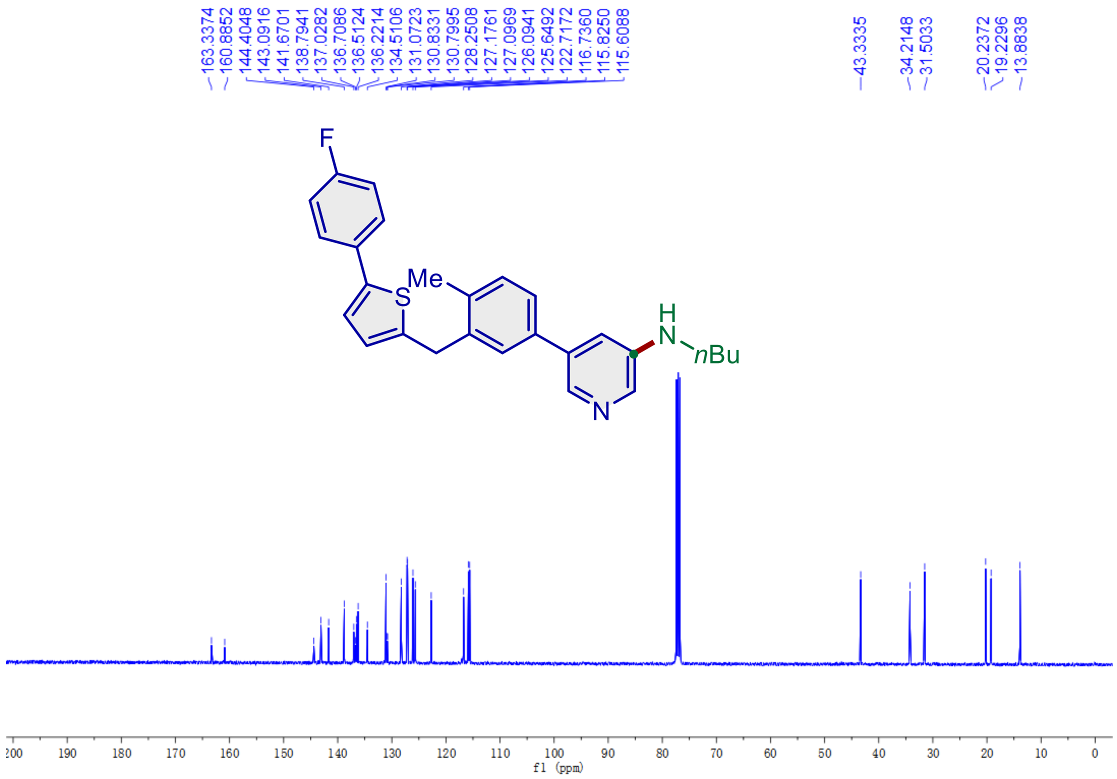

^{13}C NMR (100 MHz, $CDCl_3$) spectrum of compound 63

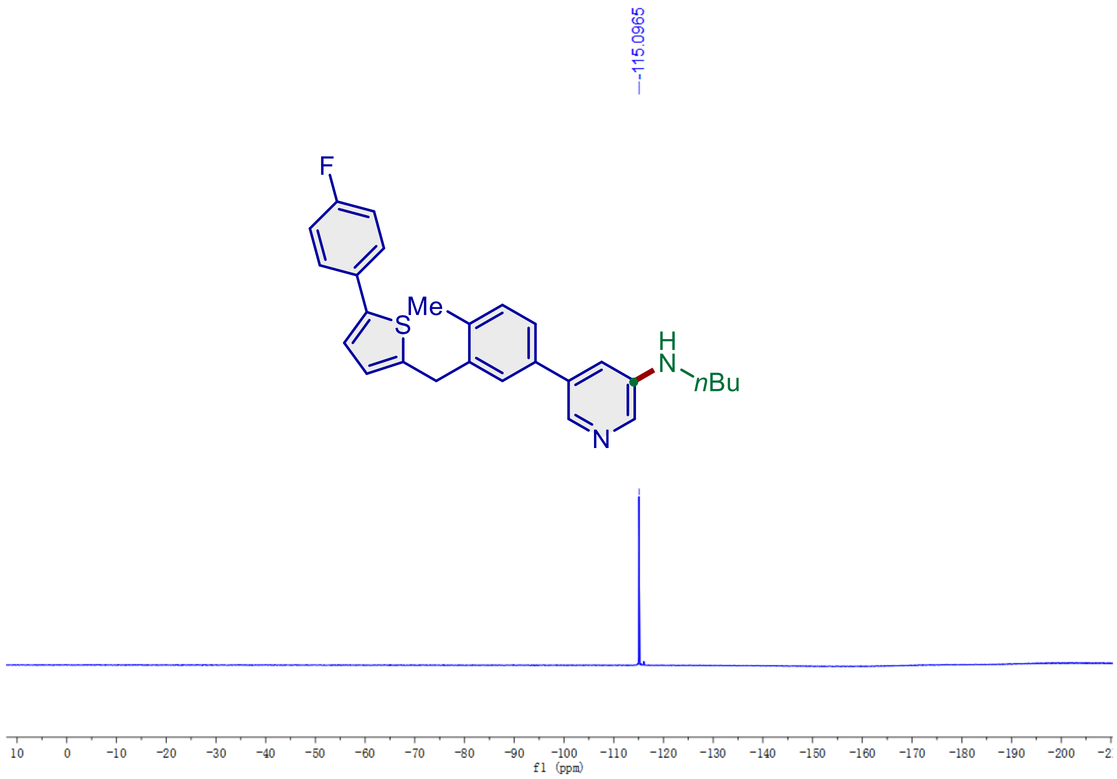

^{19}F NMR (376 MHz, $CDCl_3$) spectrum of compound 63

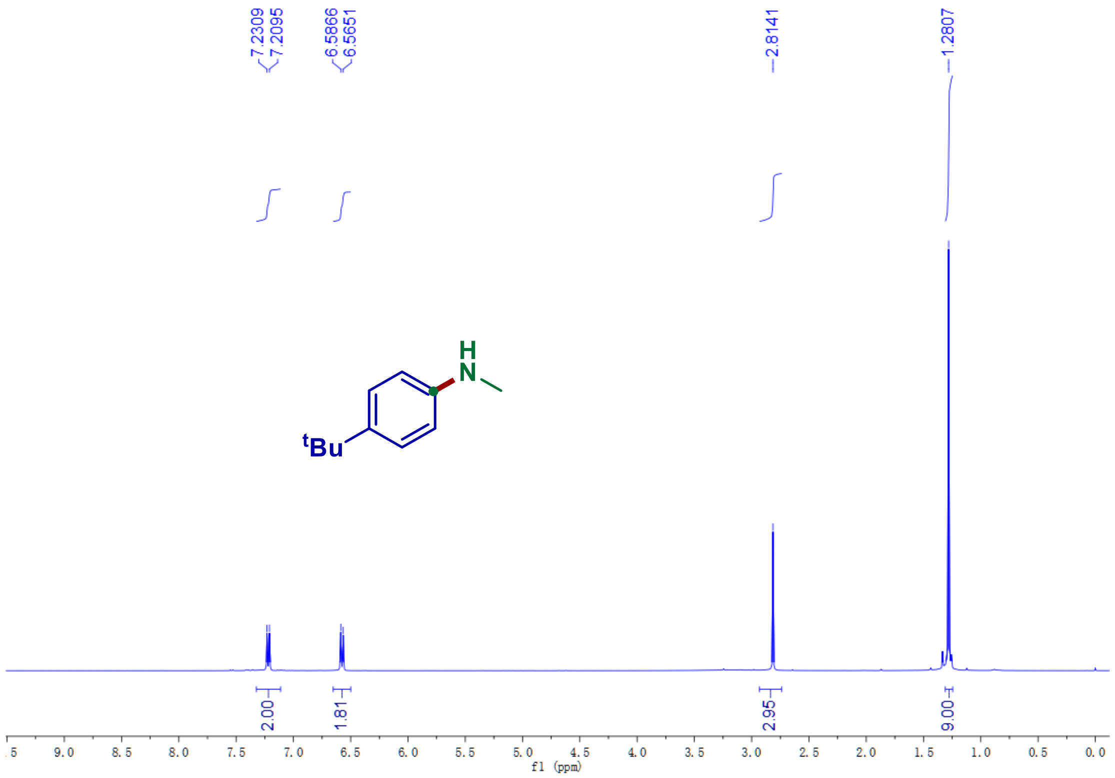

^{1}H NMR (400 MHz, $CDCl_3$) spectrum of compound 64

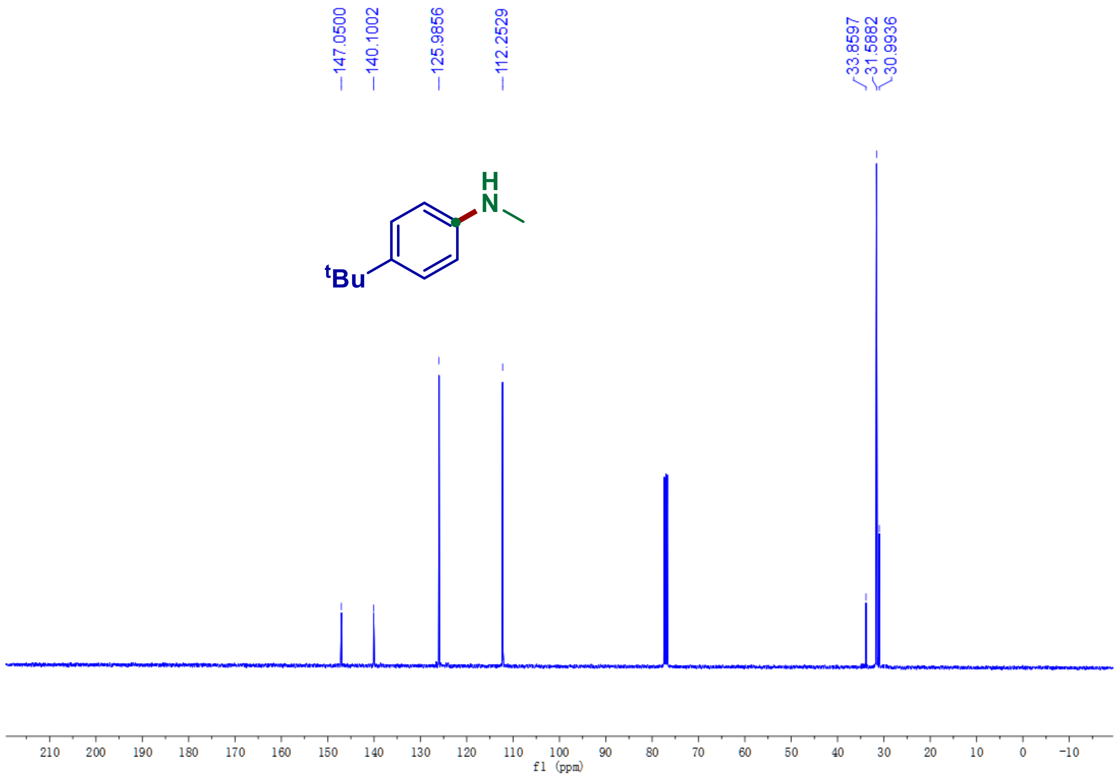

^{13}C NMR (100 MHz, $CDCl_3$) spectrum of compound 64

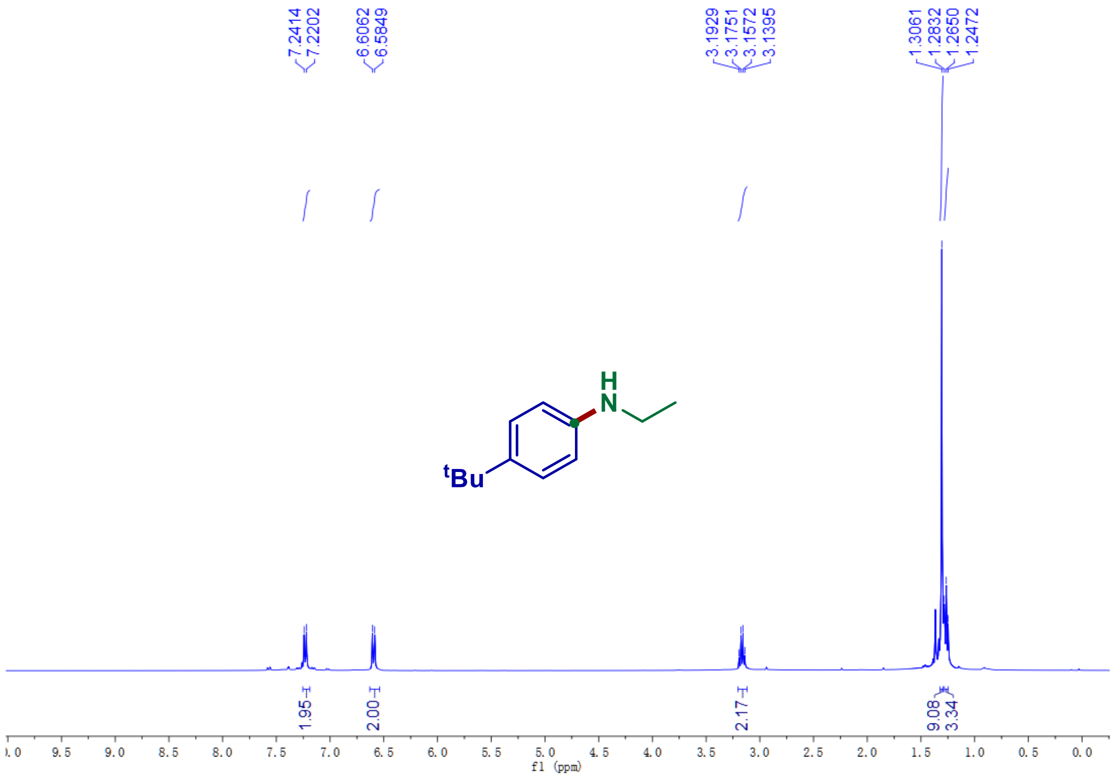

^{1}H NMR (400 MHz, $CDCl_3$) spectrum of compound 65

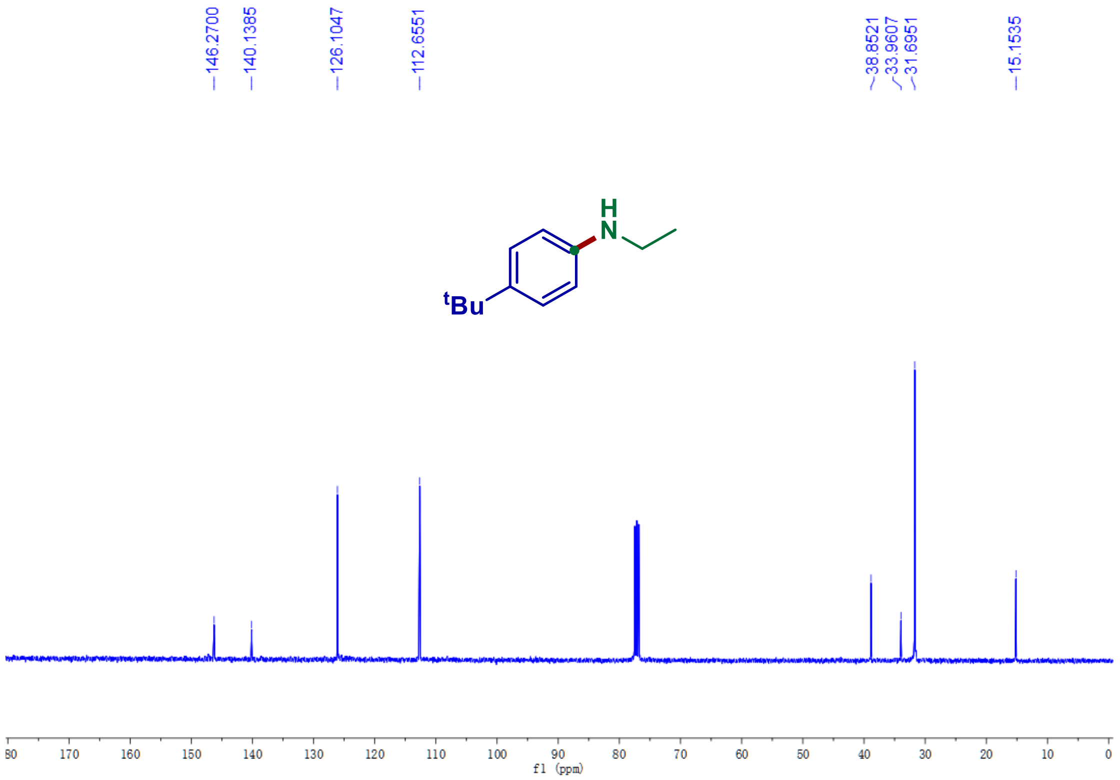

^{13}C NMR (100 MHz, $CDCl_3$) spectrum of compound 65

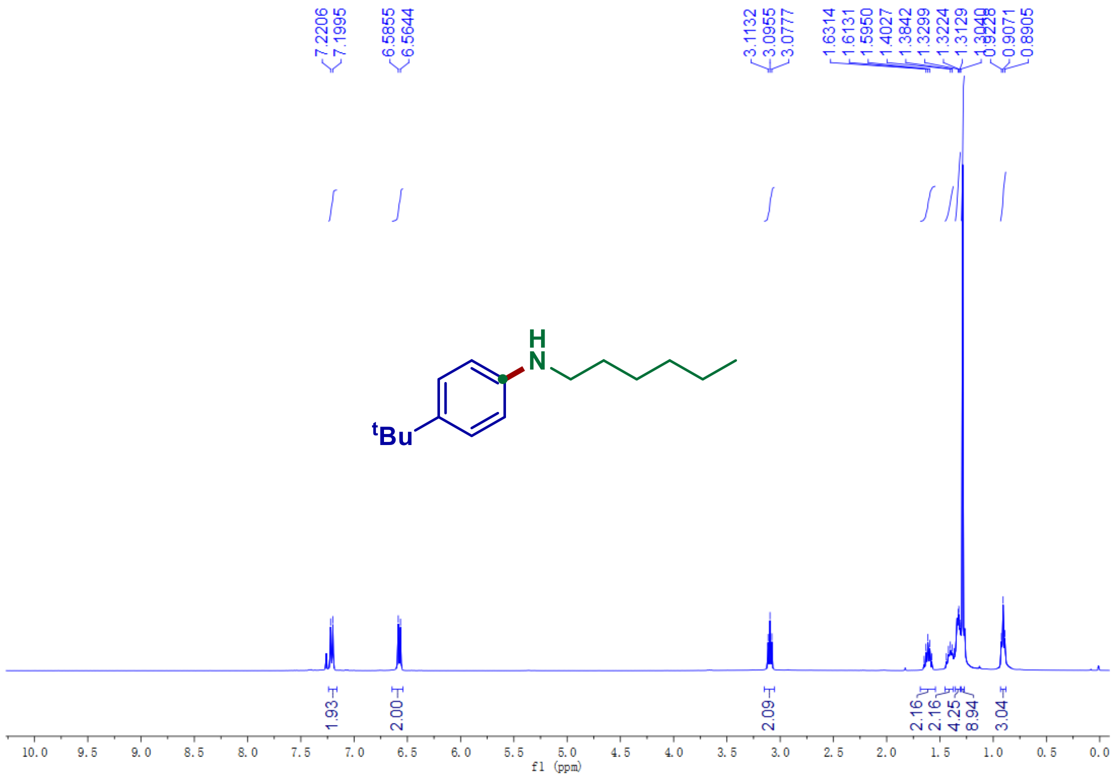

^{1}H NMR (400 MHz, $CDCl_3$) spectrum of compound 66

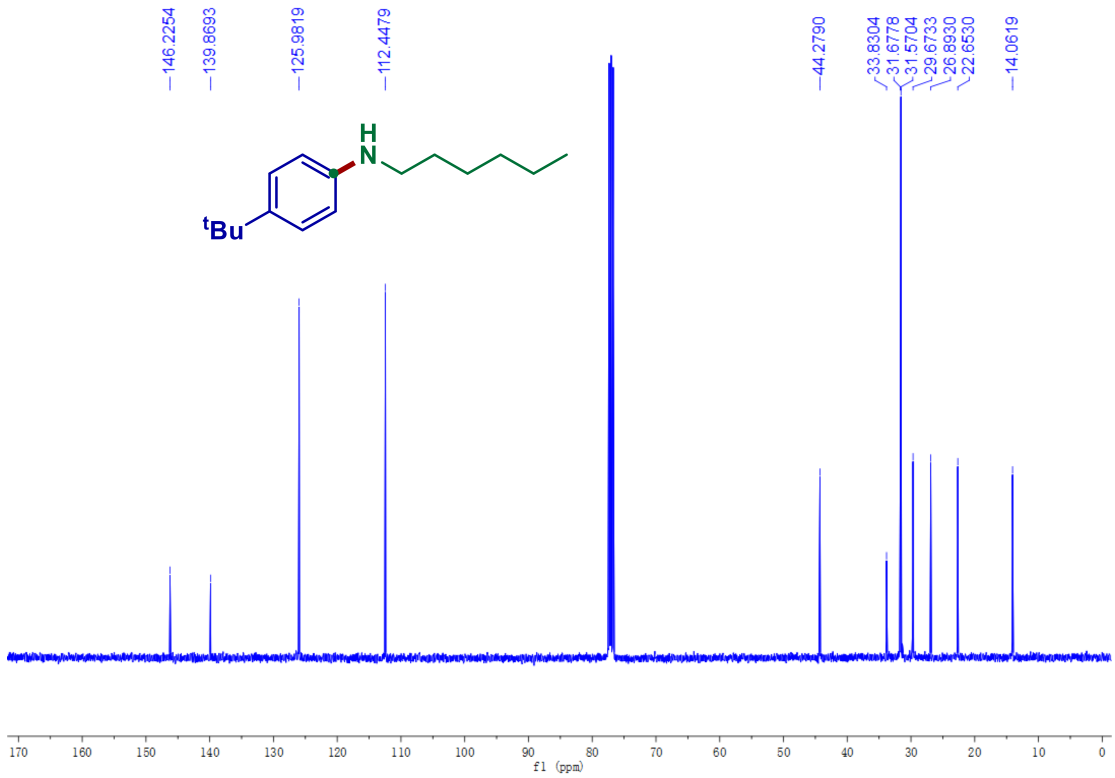

^{13}C NMR (100 MHz, $CDCl_3$) spectrum of compound 66

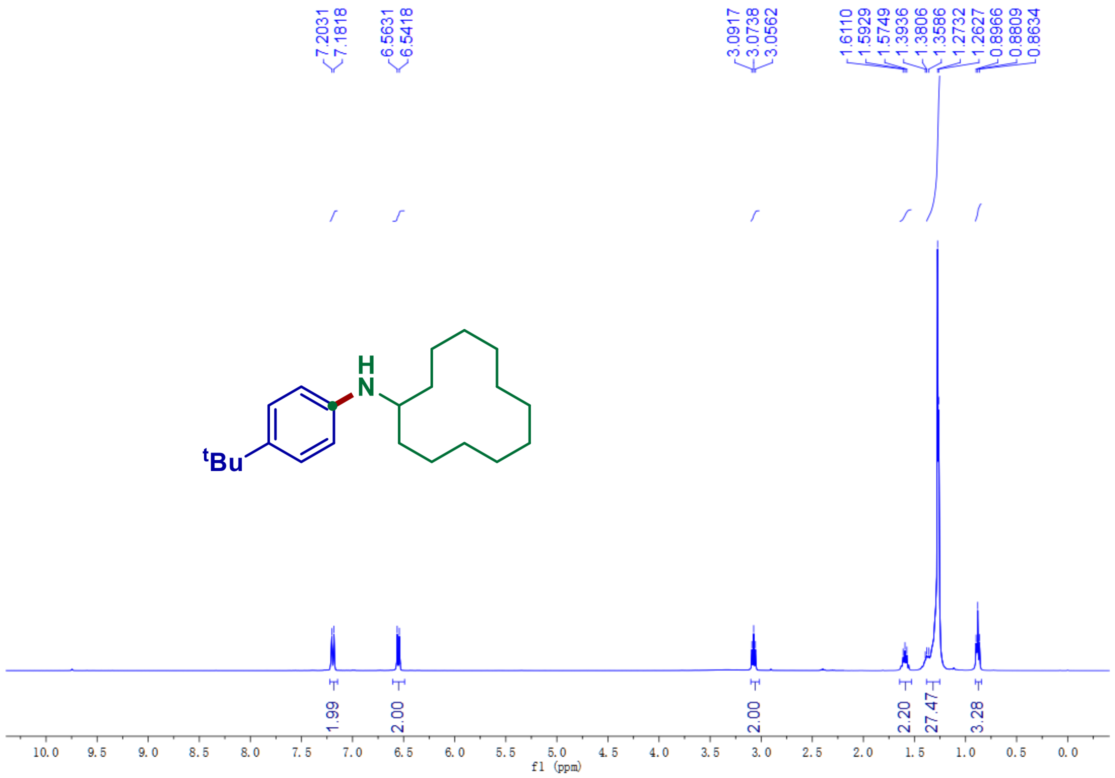

^{1}H NMR (400 MHz, $CDCl_3$) spectrum of compound 67

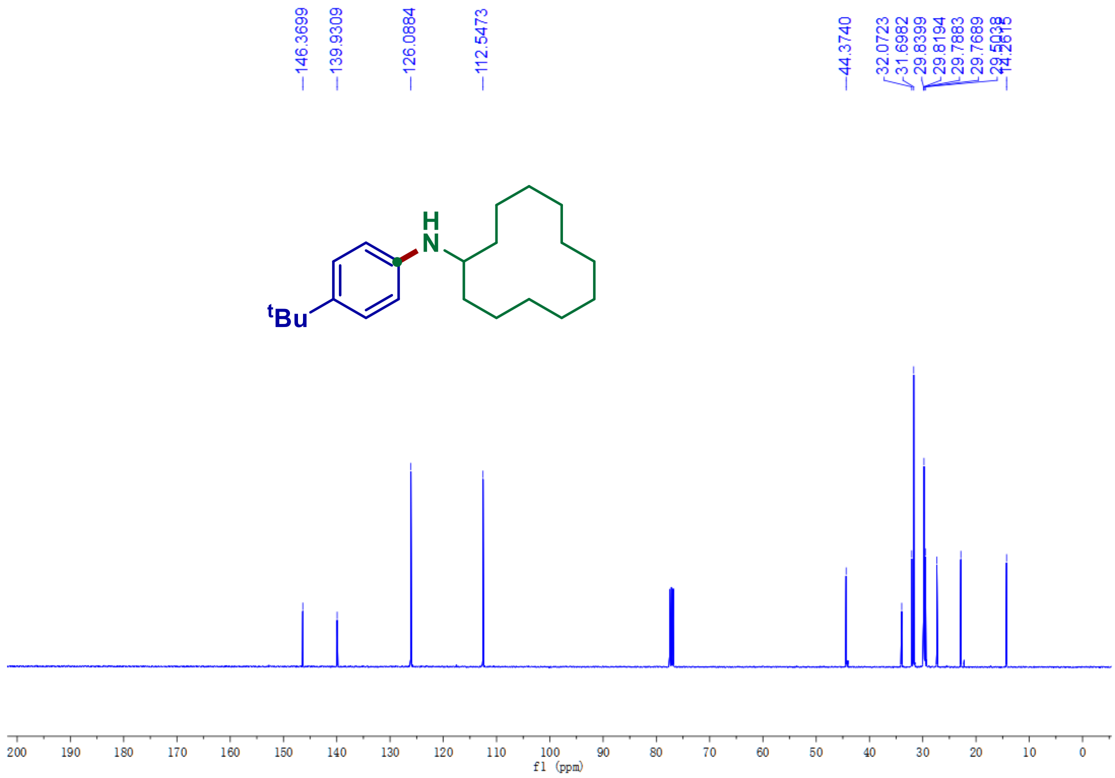

^{13}C NMR (100 MHz, $CDCl_3$) spectrum of compound 67

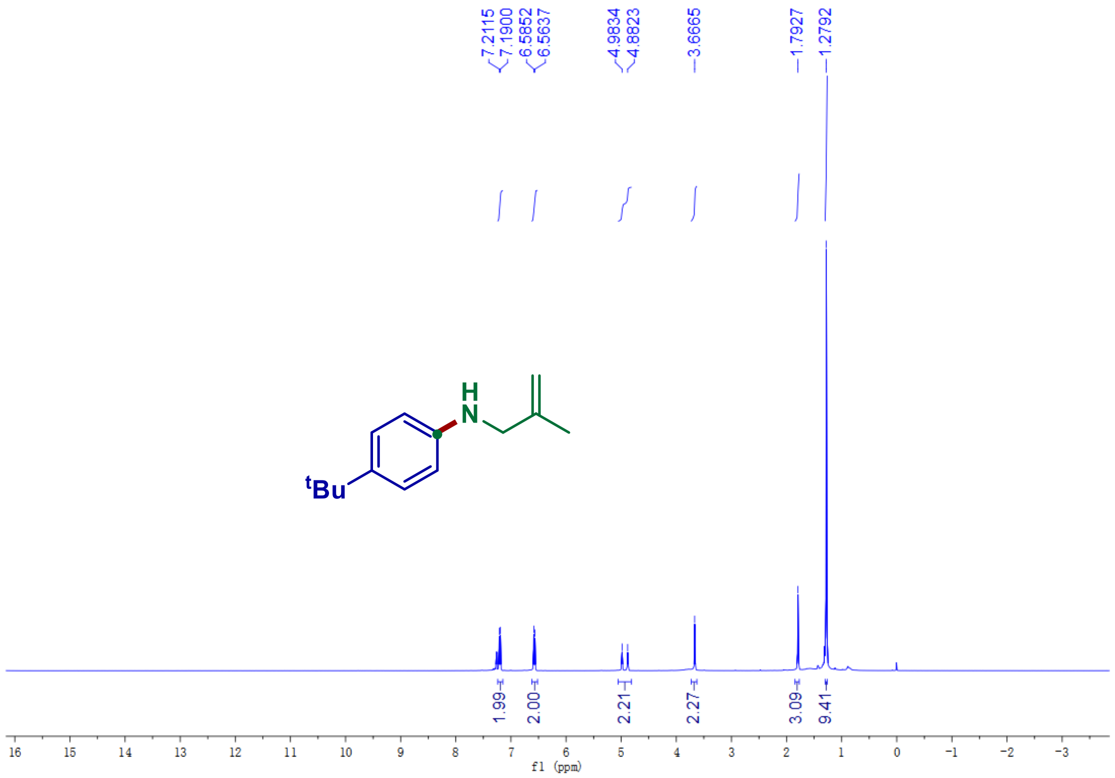

^{1}H NMR (400 MHz, $CDCl_3$) spectrum of compound 68

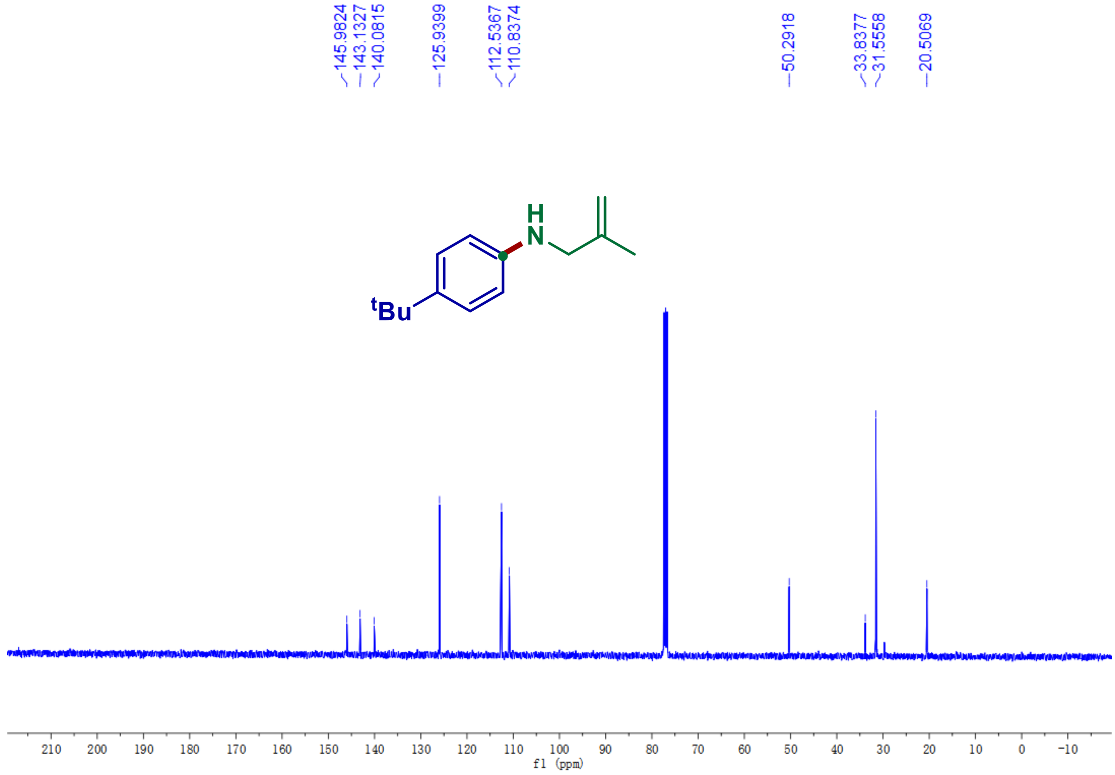

^{13}C NMR (100 MHz, $CDCl_3$) spectrum of compound 68

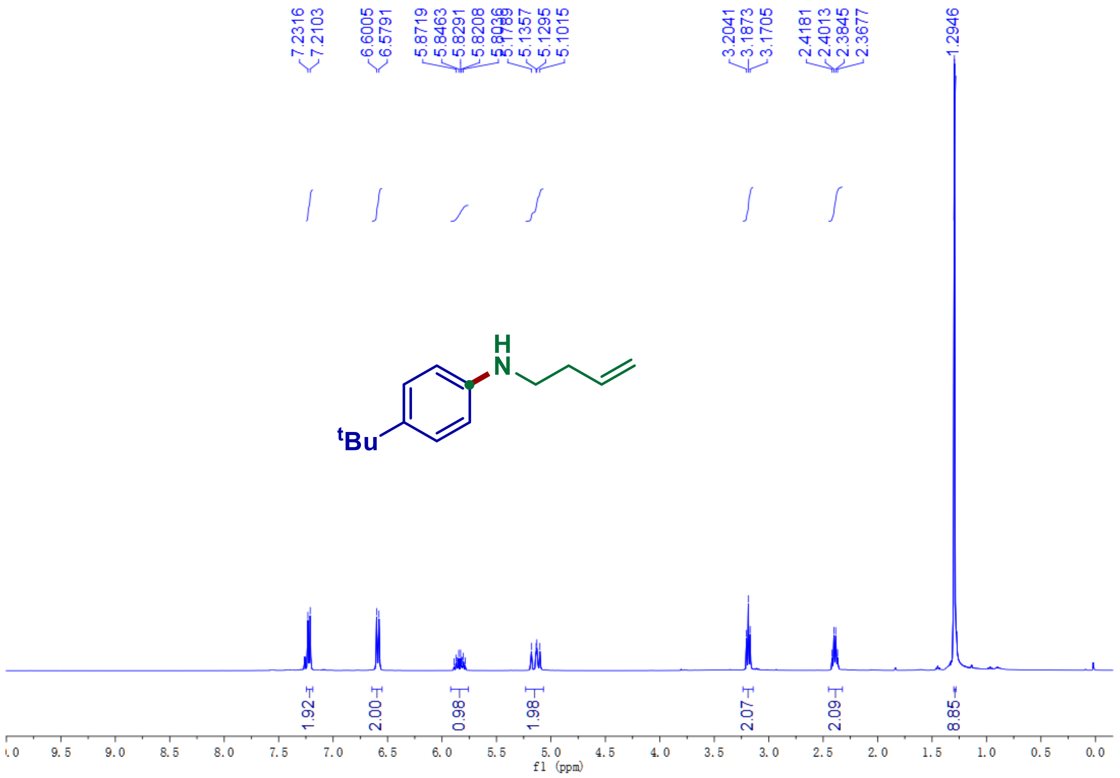

^{1}H NMR (400 MHz, $CDCl_3$) spectrum of compound 69

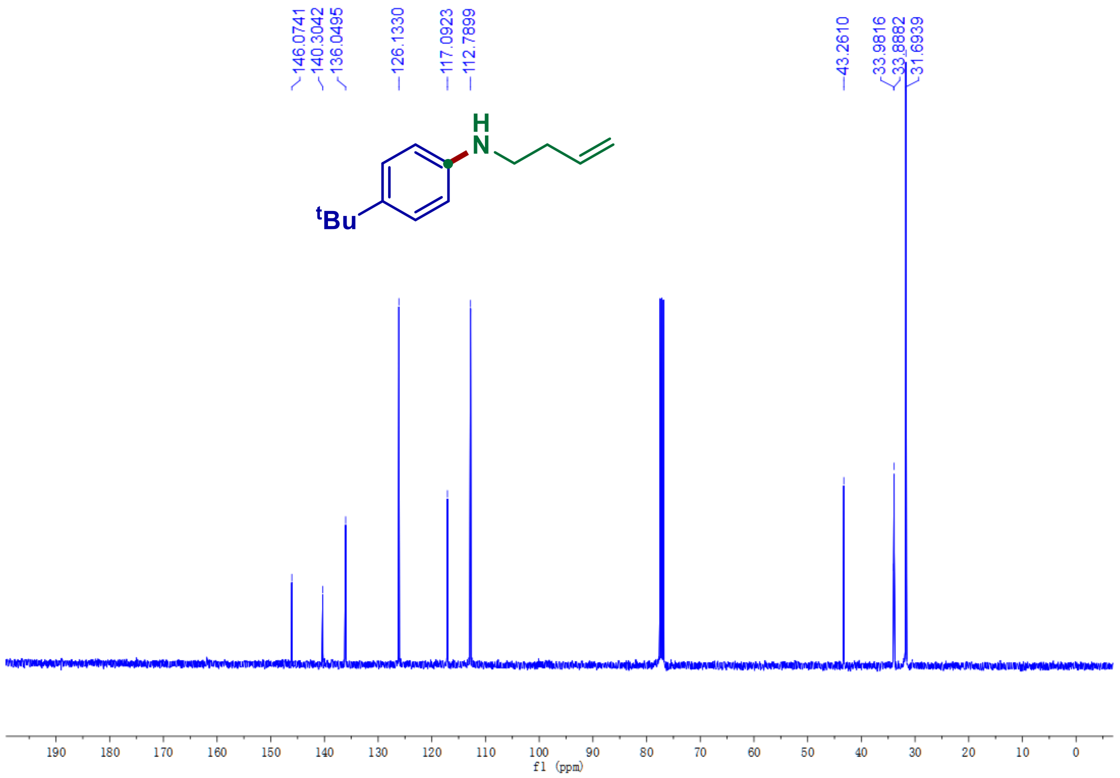

^{13}C NMR (100 MHz, $CDCl_3$) spectrum of compound 69

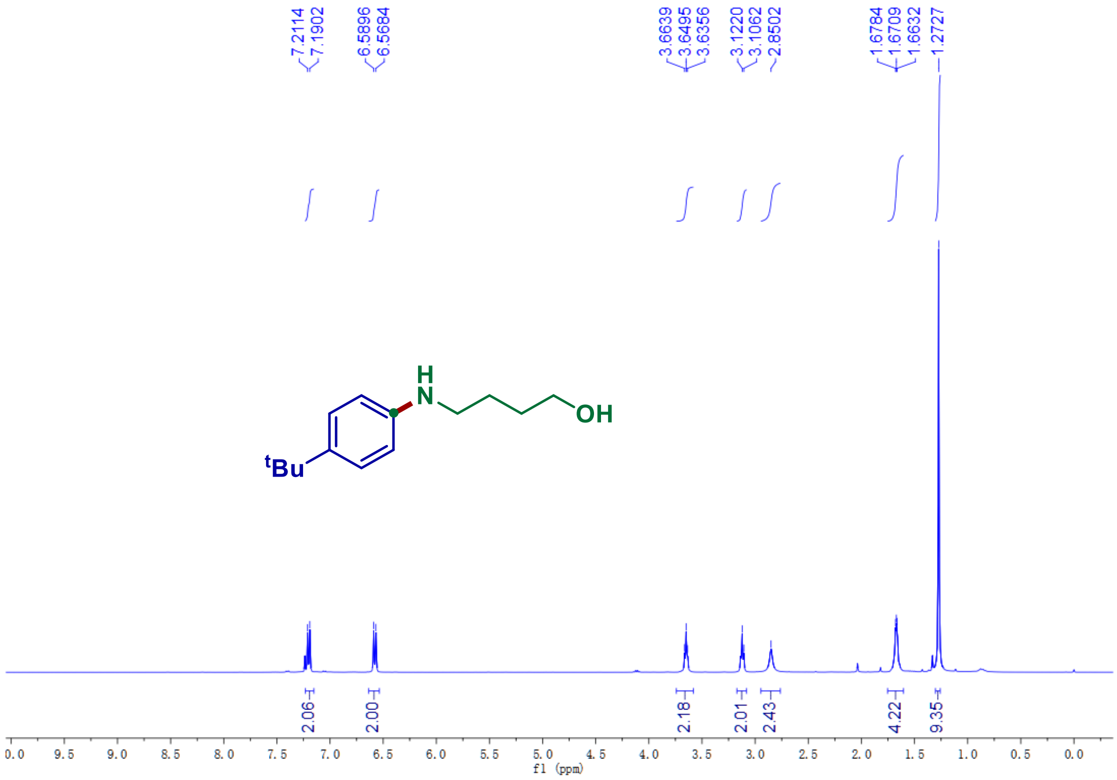

^{1}H NMR (400 MHz, $CDCl_3$) spectrum of compound 70

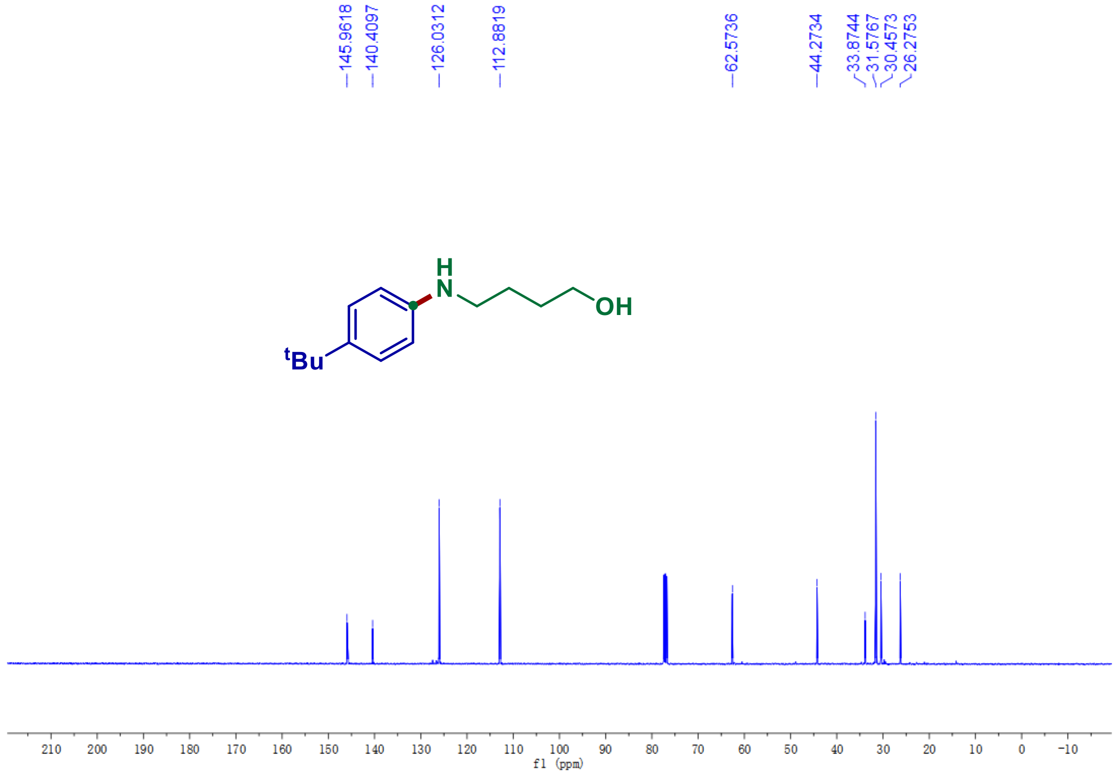

^{13}C NMR (100 MHz, $CDCl_3$) spectrum of compound 70

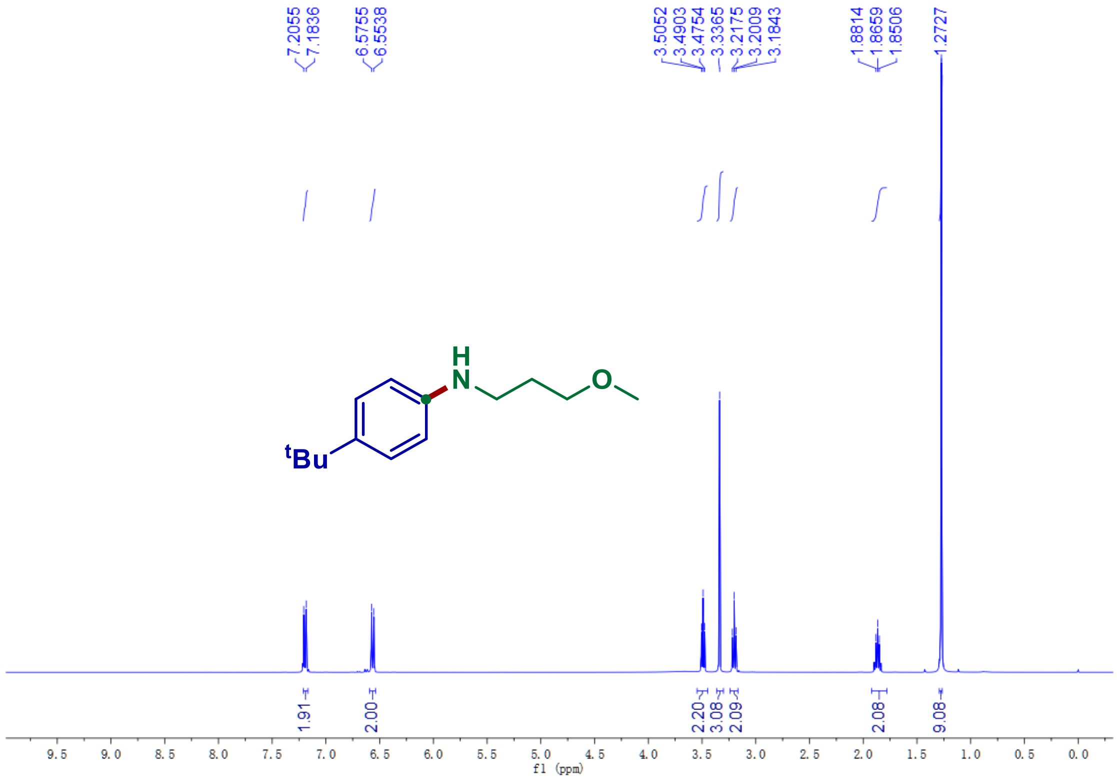

^{1}H NMR (400 MHz, $CDCl_3$) spectrum of compound 71

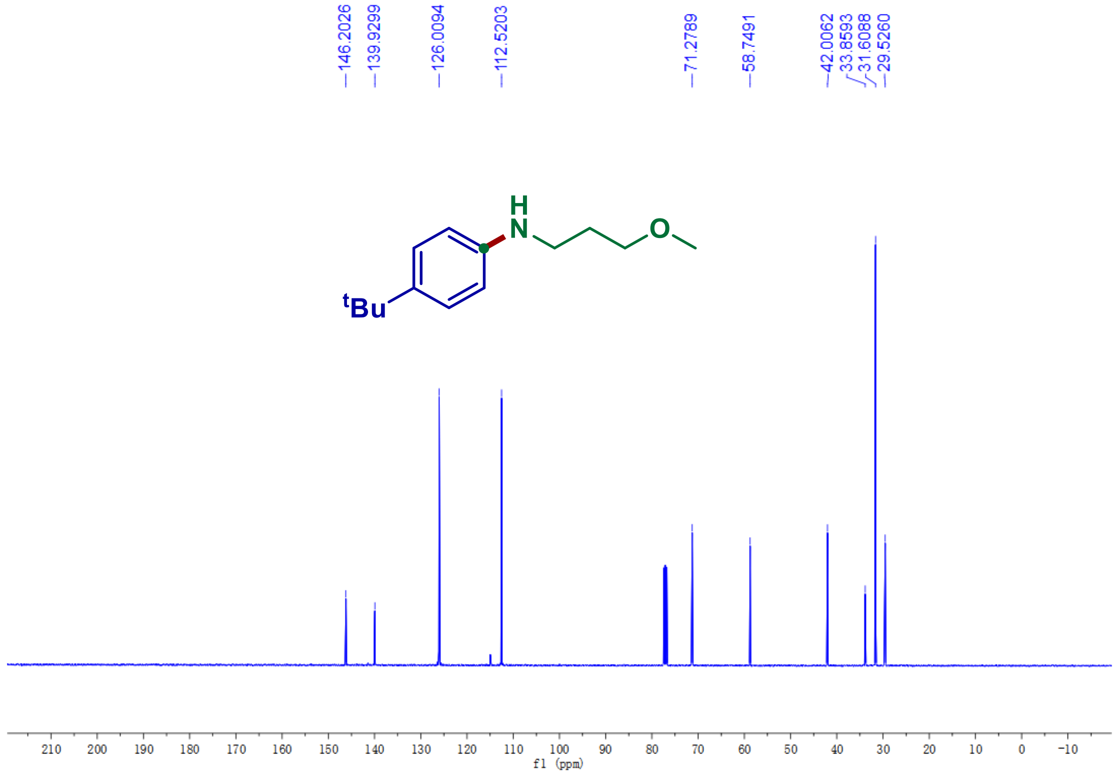

^{13}C NMR (100 MHz, $CDCl_3$) spectrum of compound 71

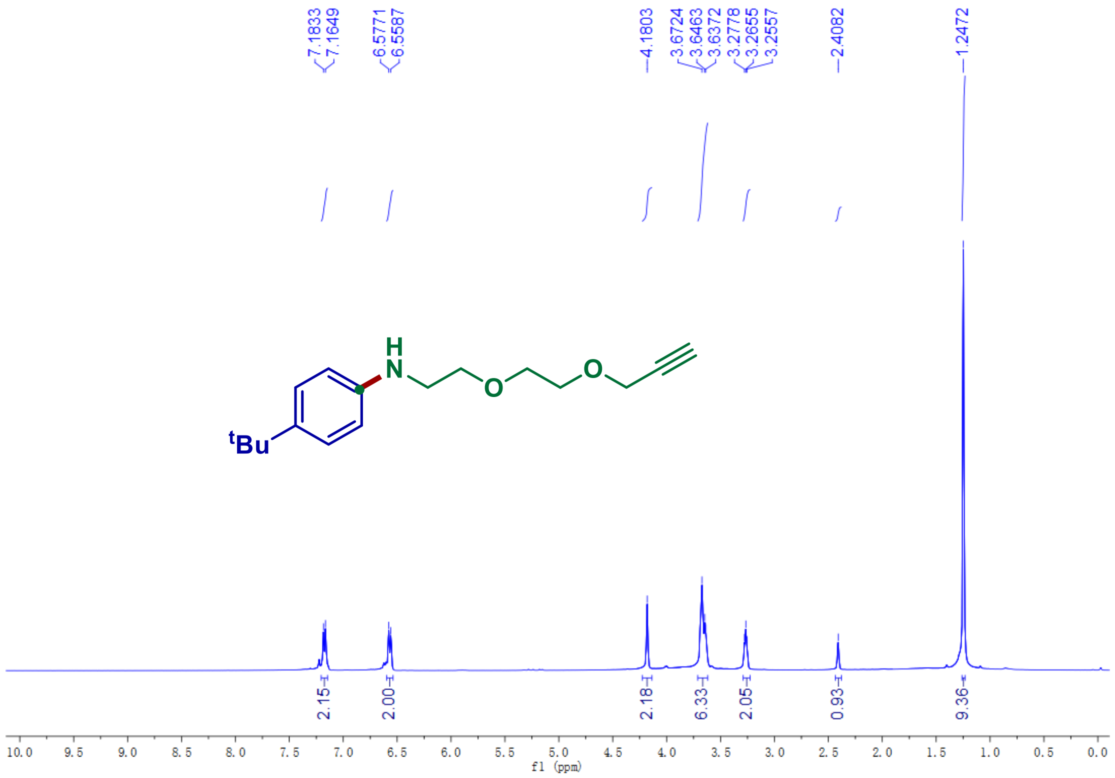

^{1}H NMR (400 MHz, $CDCl_3$) spectrum of compound 72

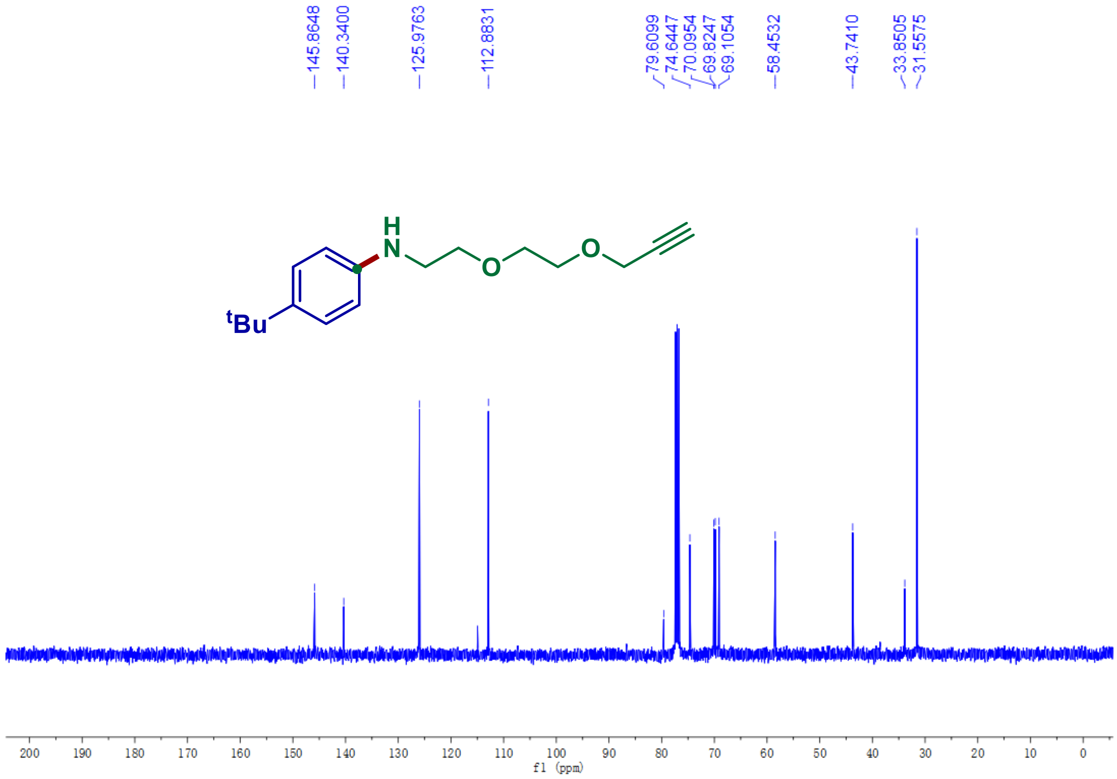

^{13}C NMR (100 MHz, $CDCl_3$) spectrum of compound 72

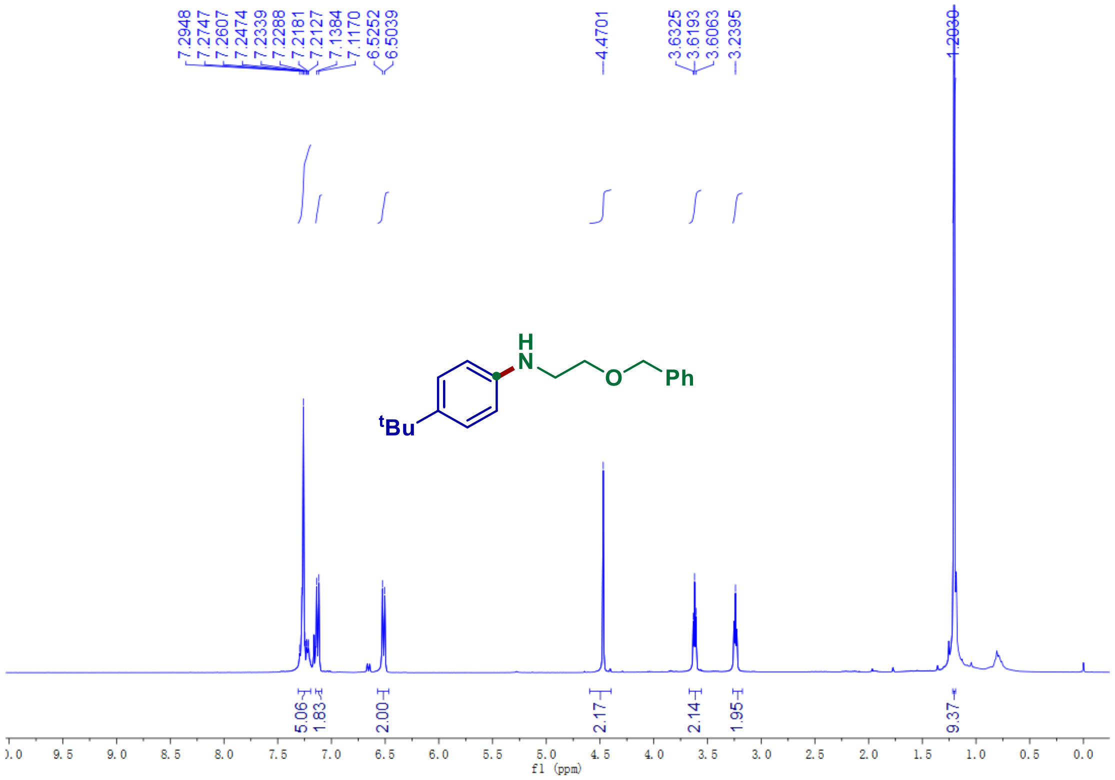

^{1}H NMR (400 MHz, $CDCl_3$) spectrum of compound 73

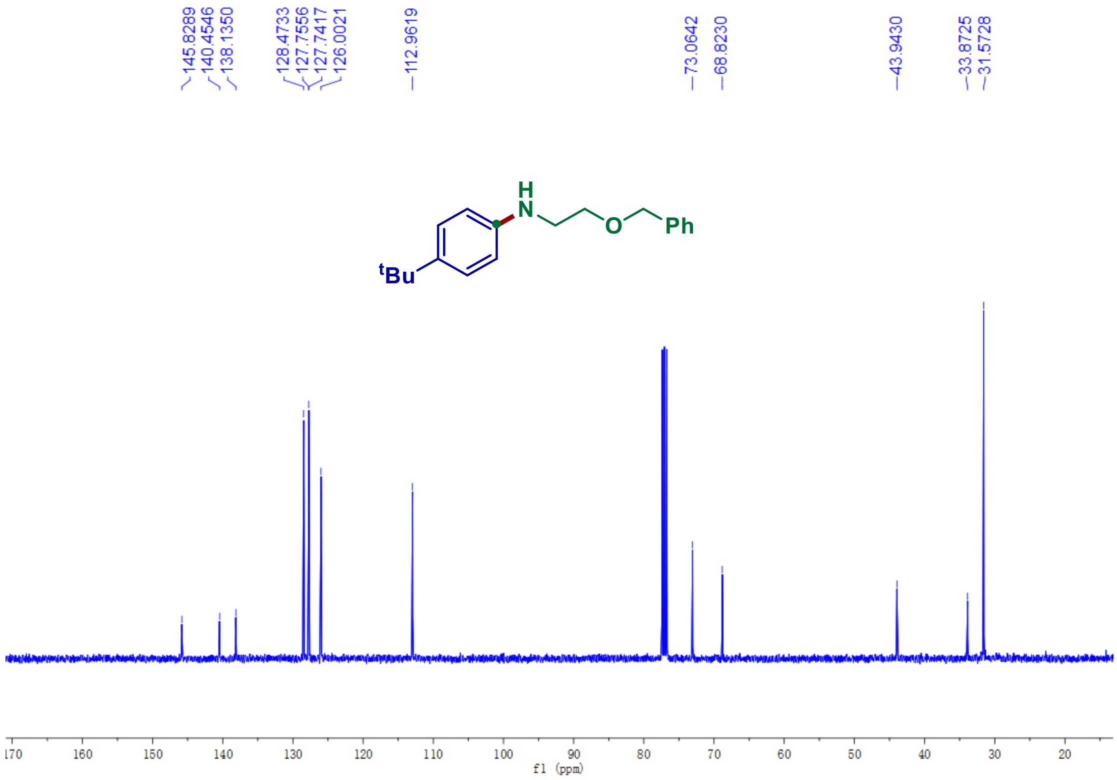

^{13}C NMR (100 MHz, $CDCl_3$) spectrum of compound 73

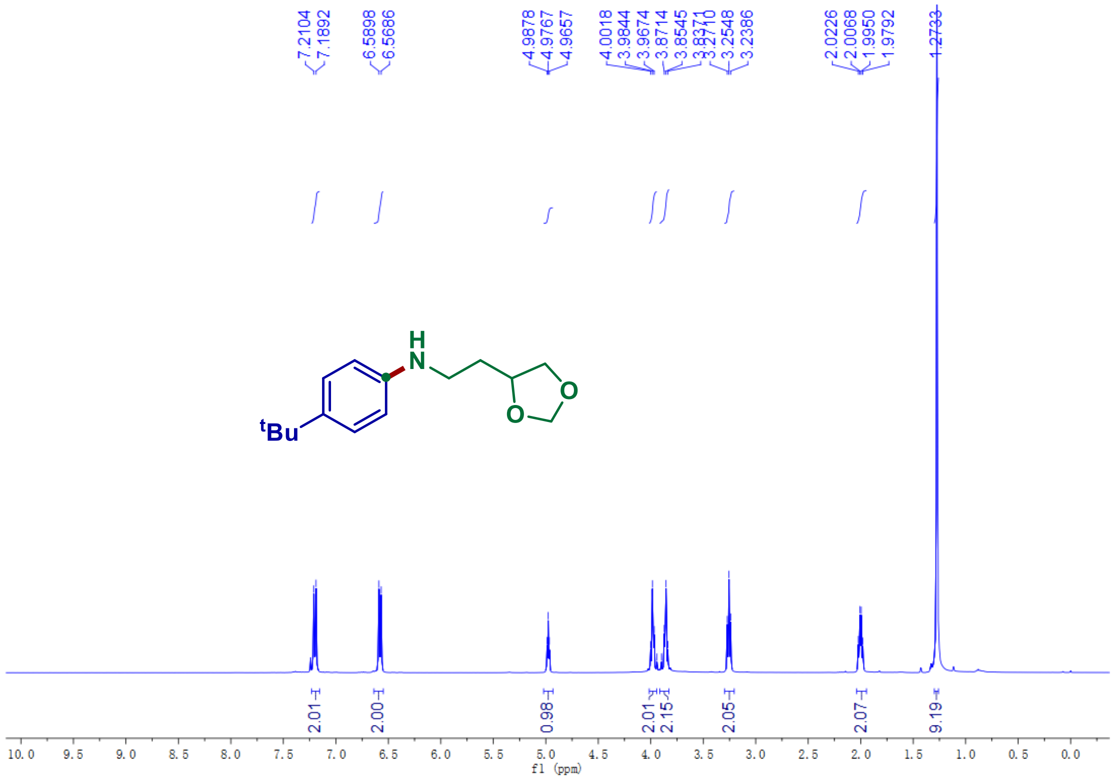

^{1}H NMR (400 MHz, $CDCl_3$) spectrum of compound 74

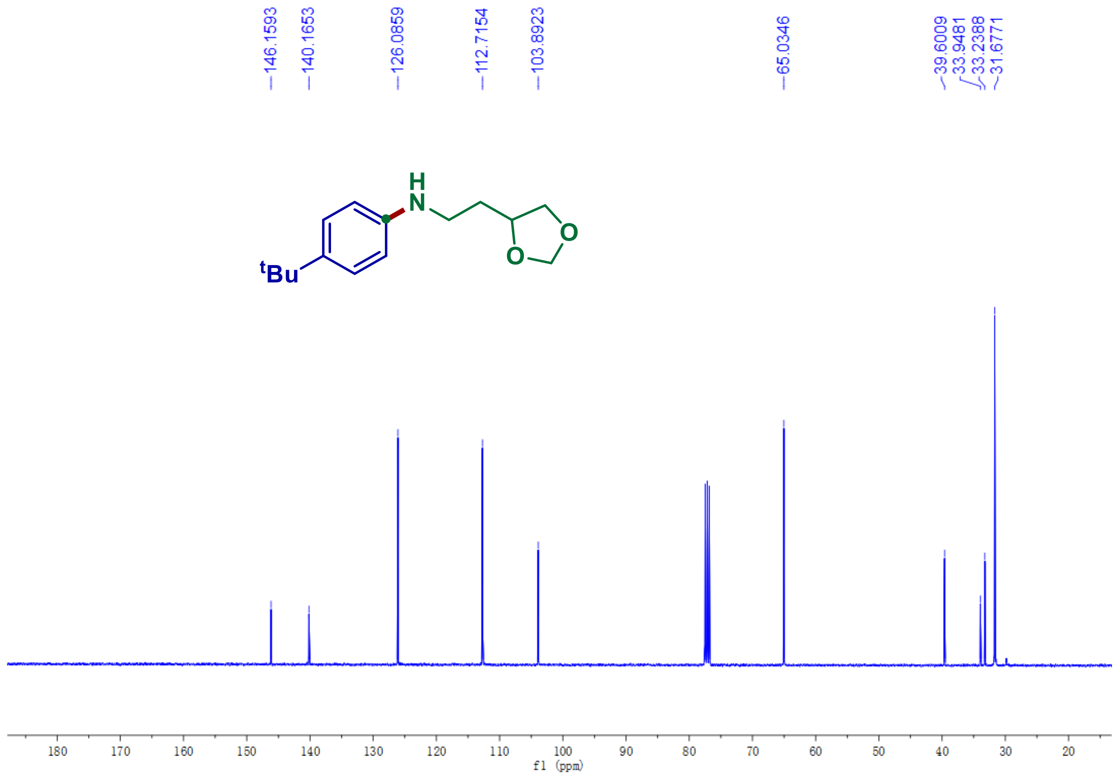

^{13}C NMR (100 MHz, $CDCl_3$) spectrum of compound 74

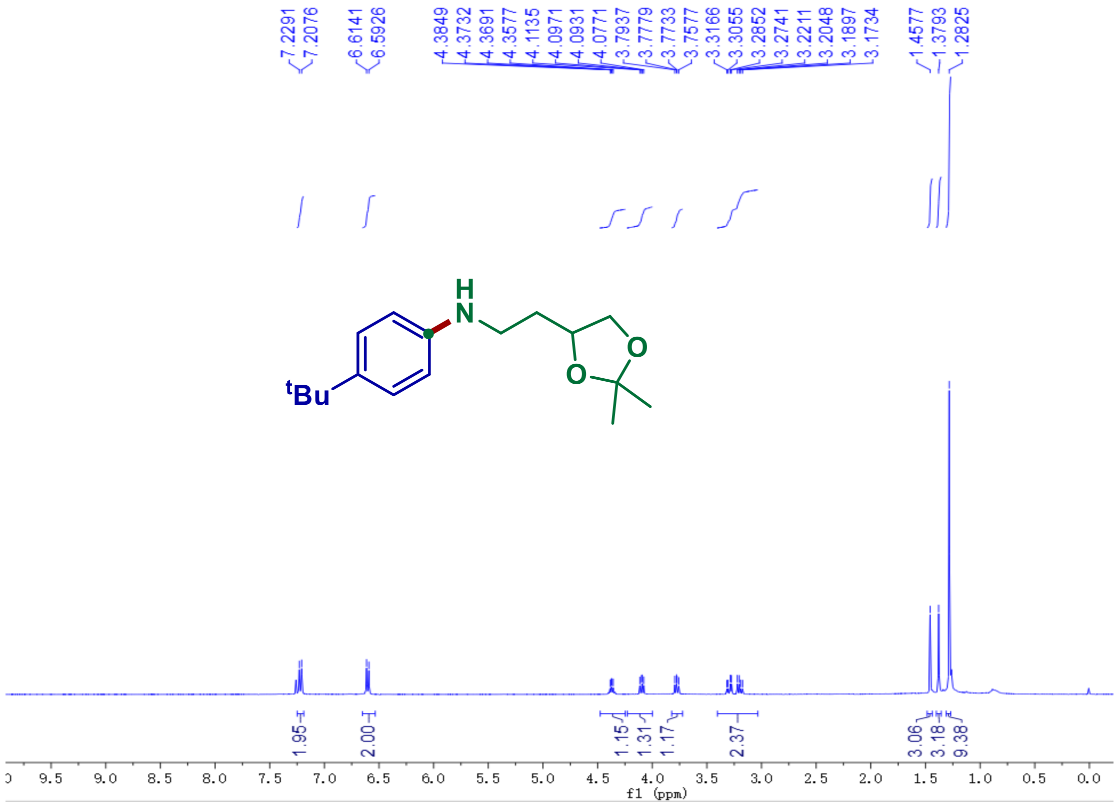

^{1}H NMR (400 MHz, CDCl$_3$) spectrum of compound 75

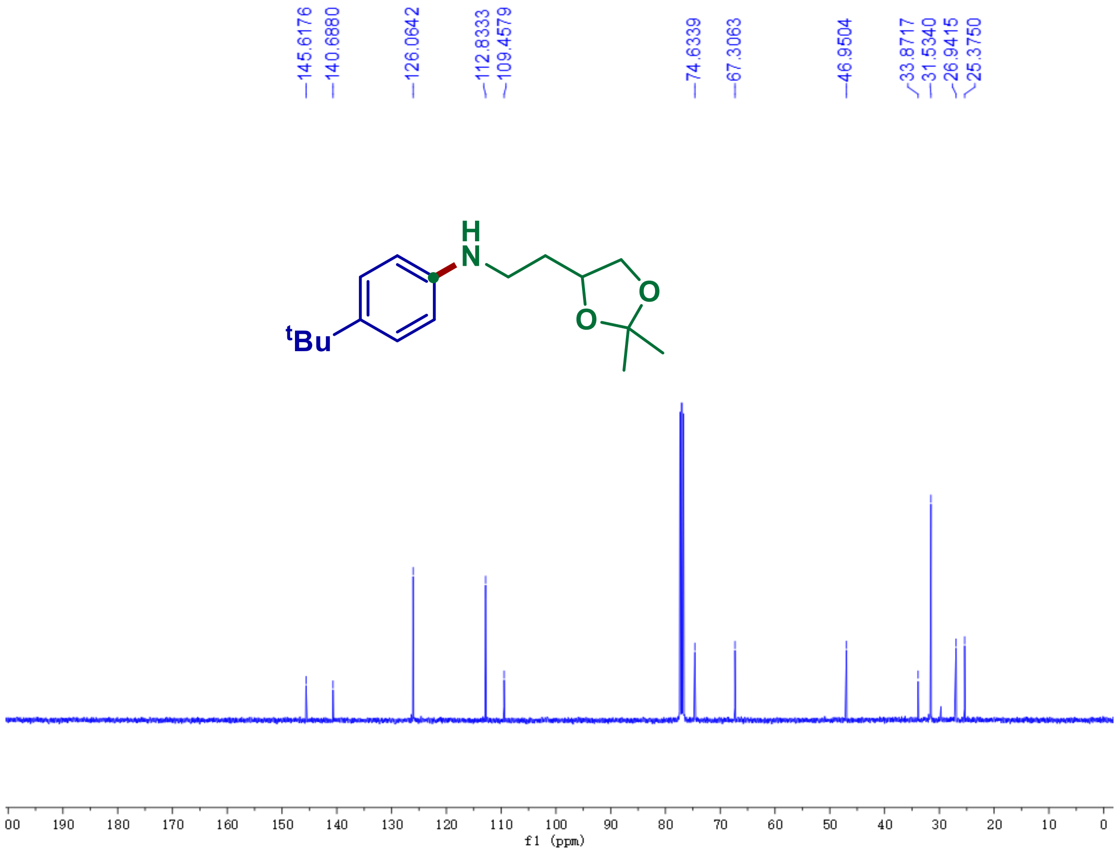

^{13}C NMR (100 MHz, CDCl$_3$) spectrum of compound 75

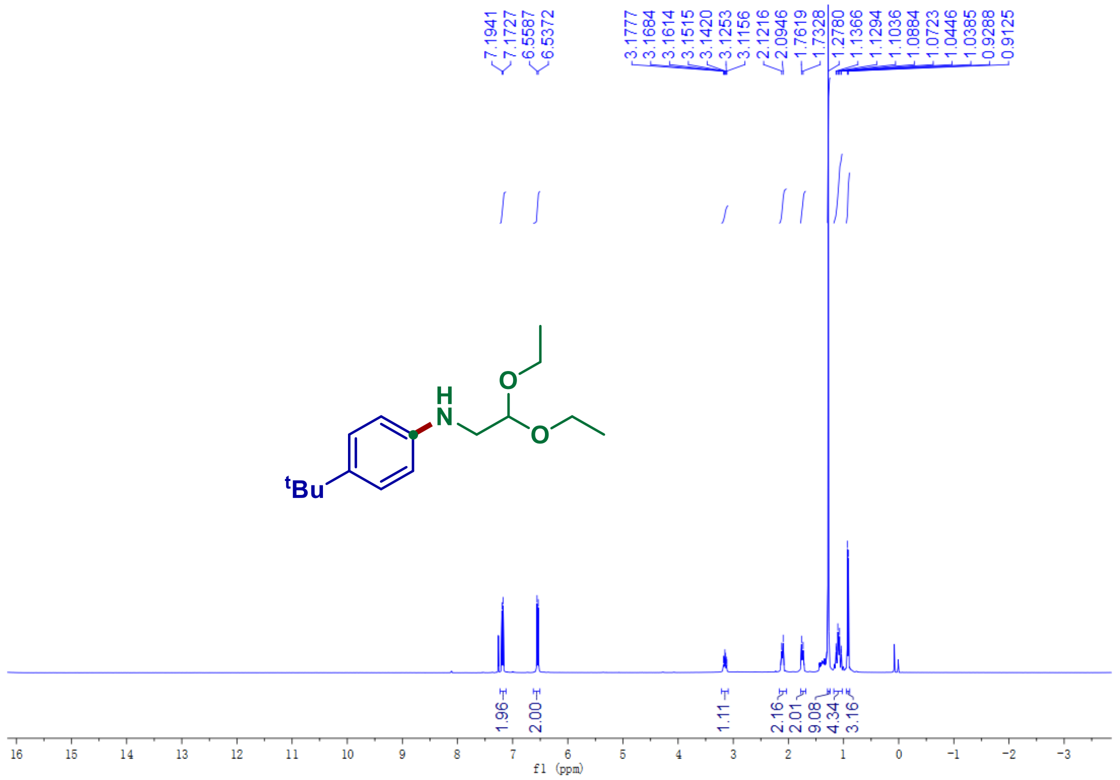

^{1}H NMR (400 MHz, $CDCl_3$) spectrum of compound 76

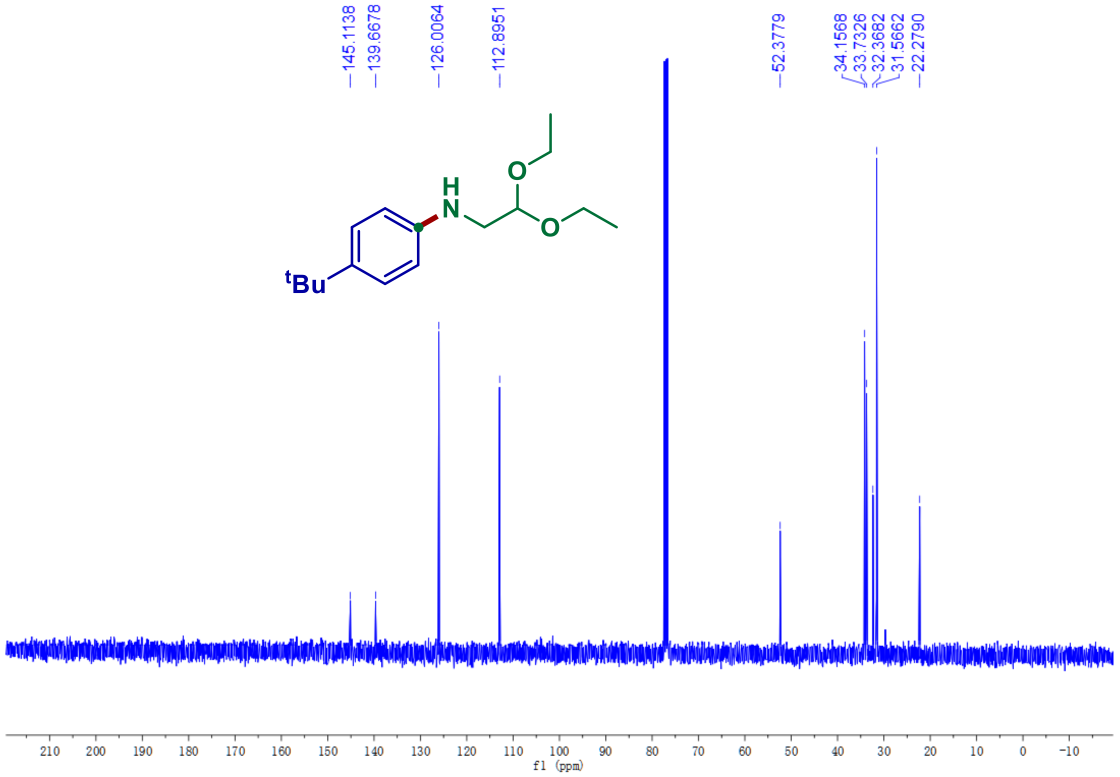

^{13}C NMR (100 MHz, $CDCl_3$) spectrum of compound 76

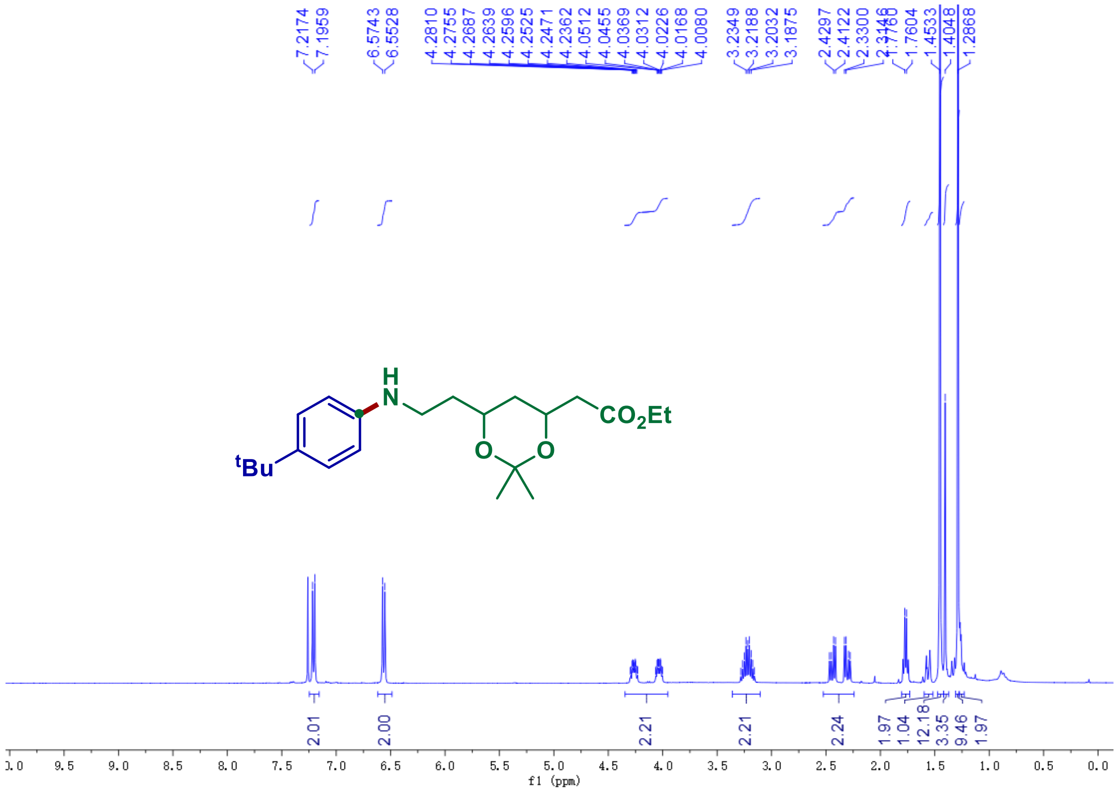

^{1}H NMR (400 MHz, $CDCl_3$) spectrum of compound 77

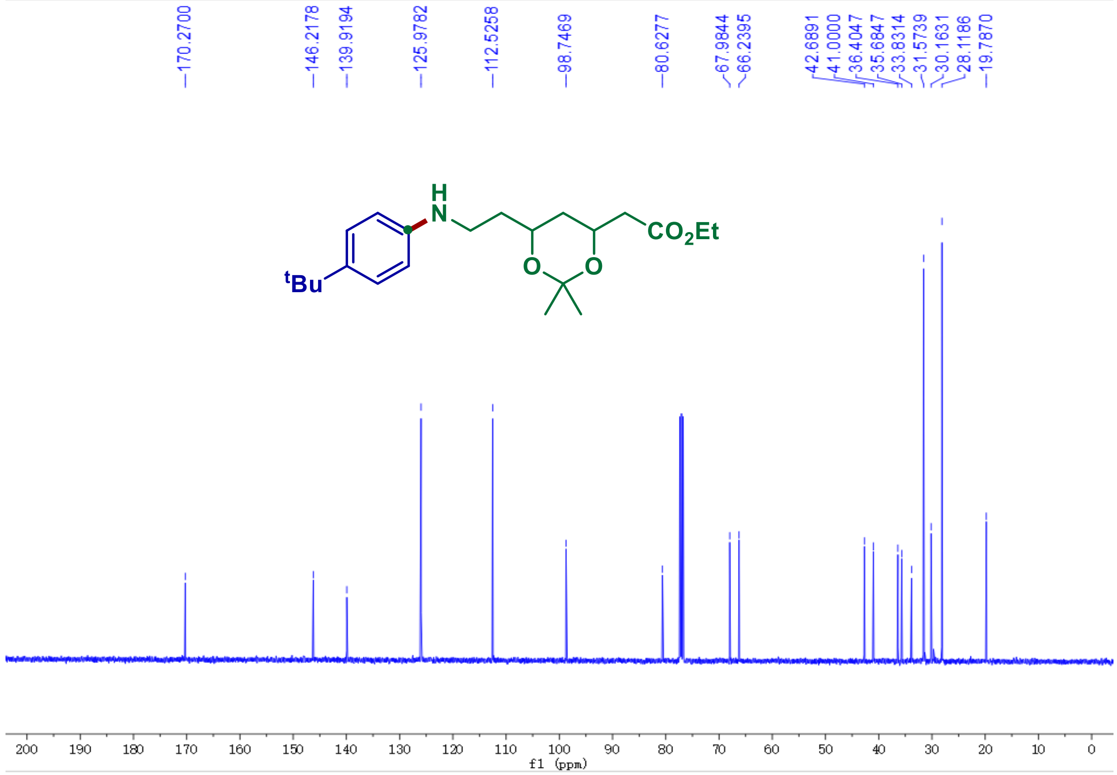

^{13}C NMR (100 MHz, $CDCl_3$) spectrum of compound 77

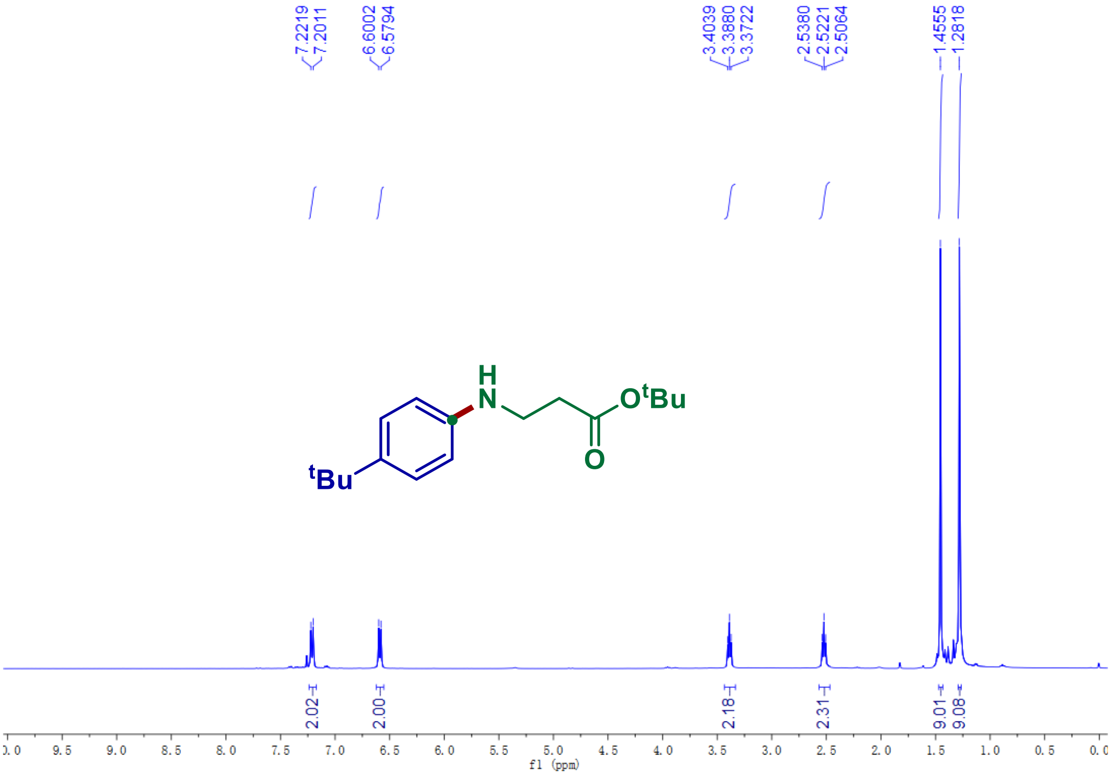

^{1}H NMR (400 MHz, $CDCl_3$) spectrum of compound 78

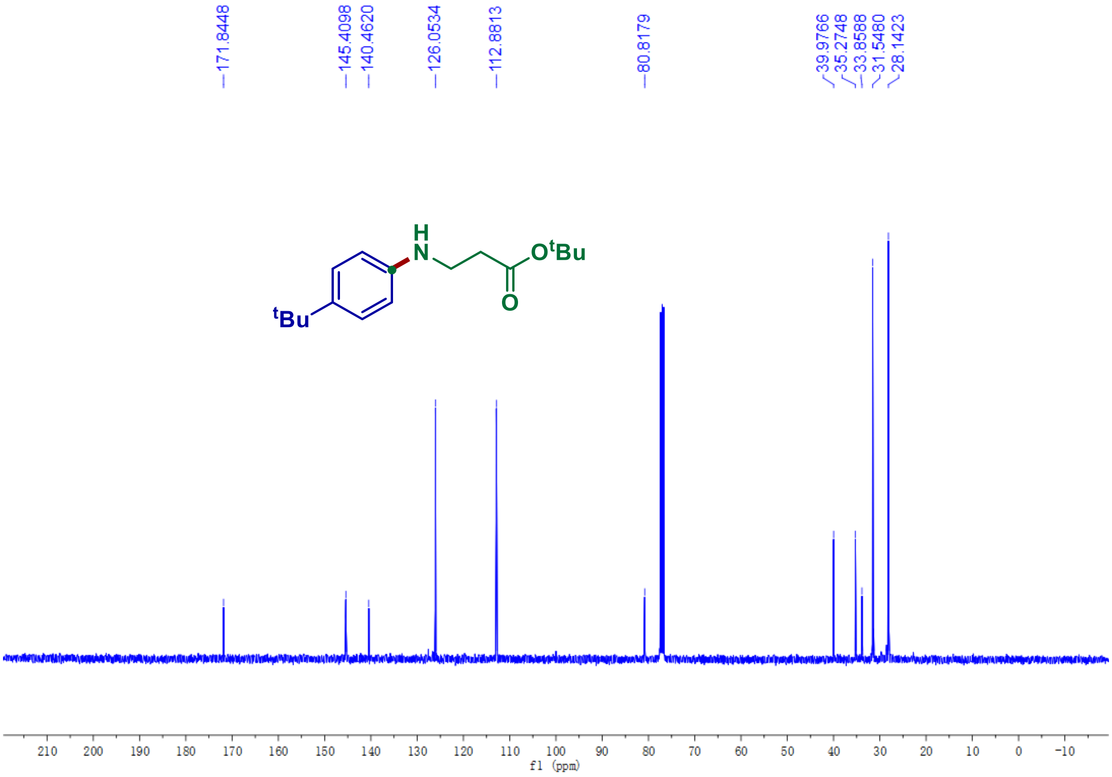

^{13}C NMR (100 MHz, $CDCl_3$) spectrum of compound 78

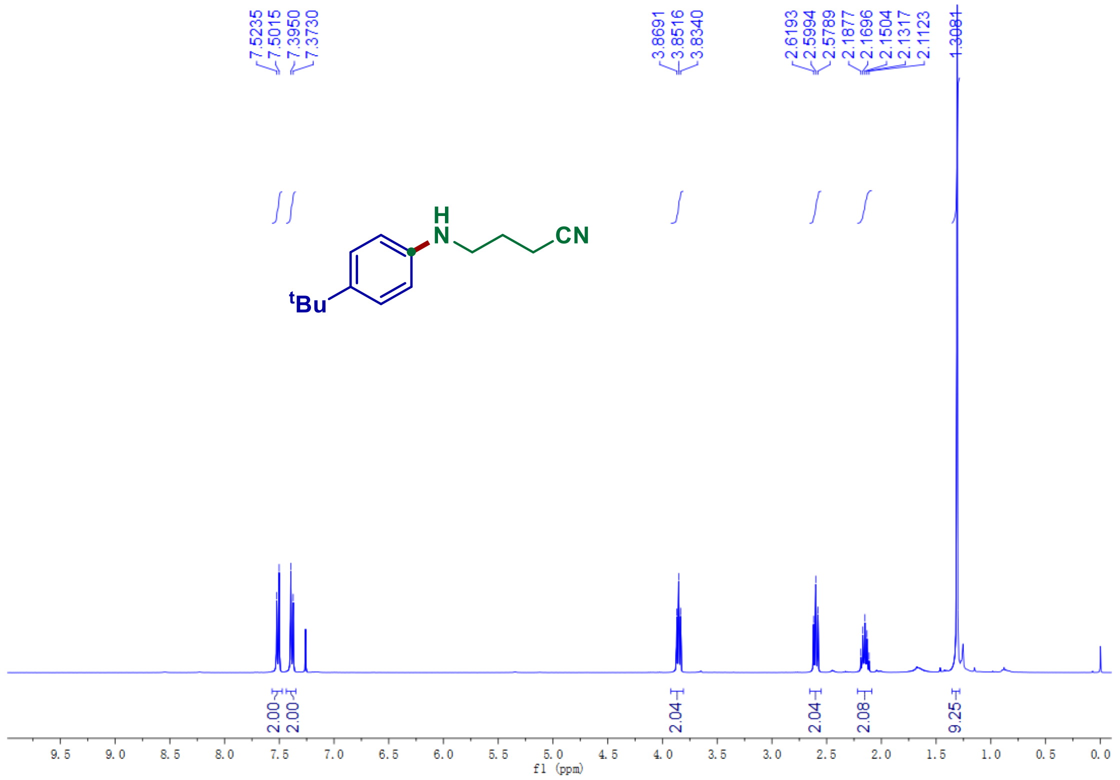

^{1}H NMR (400 MHz, CDCl$_3$) spectrum of compound 79

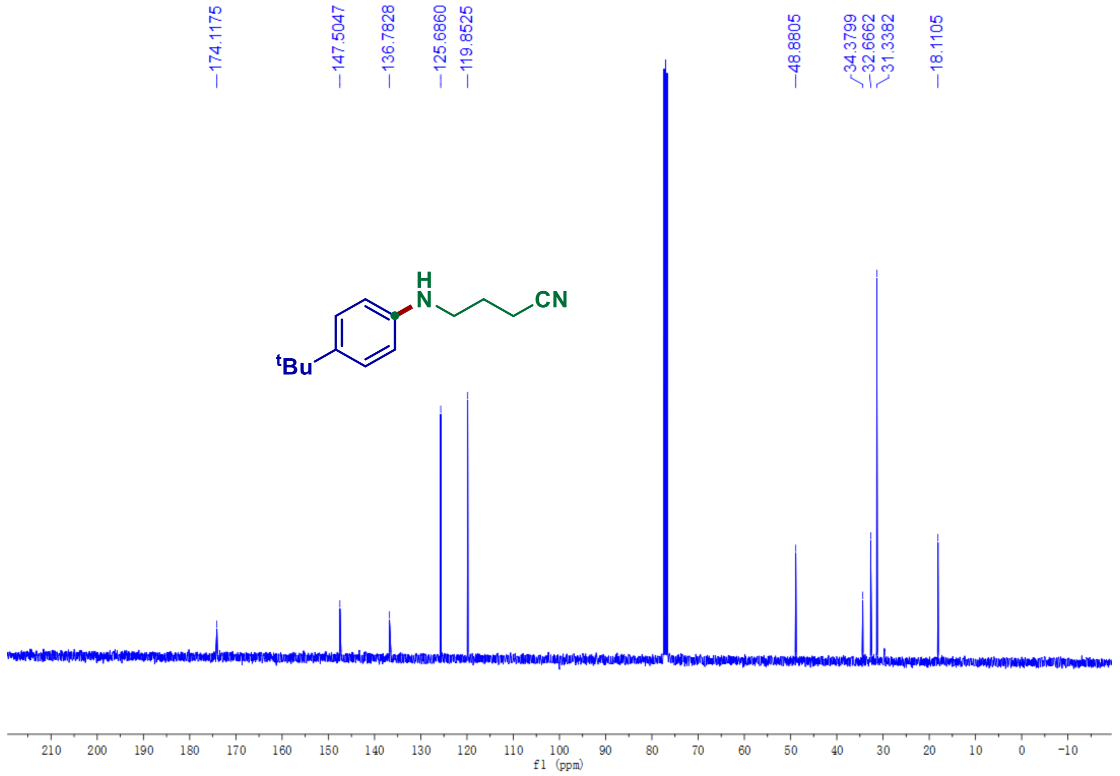

^{13}C NMR (100 MHz, CDCl$_3$) spectrum of compound 79

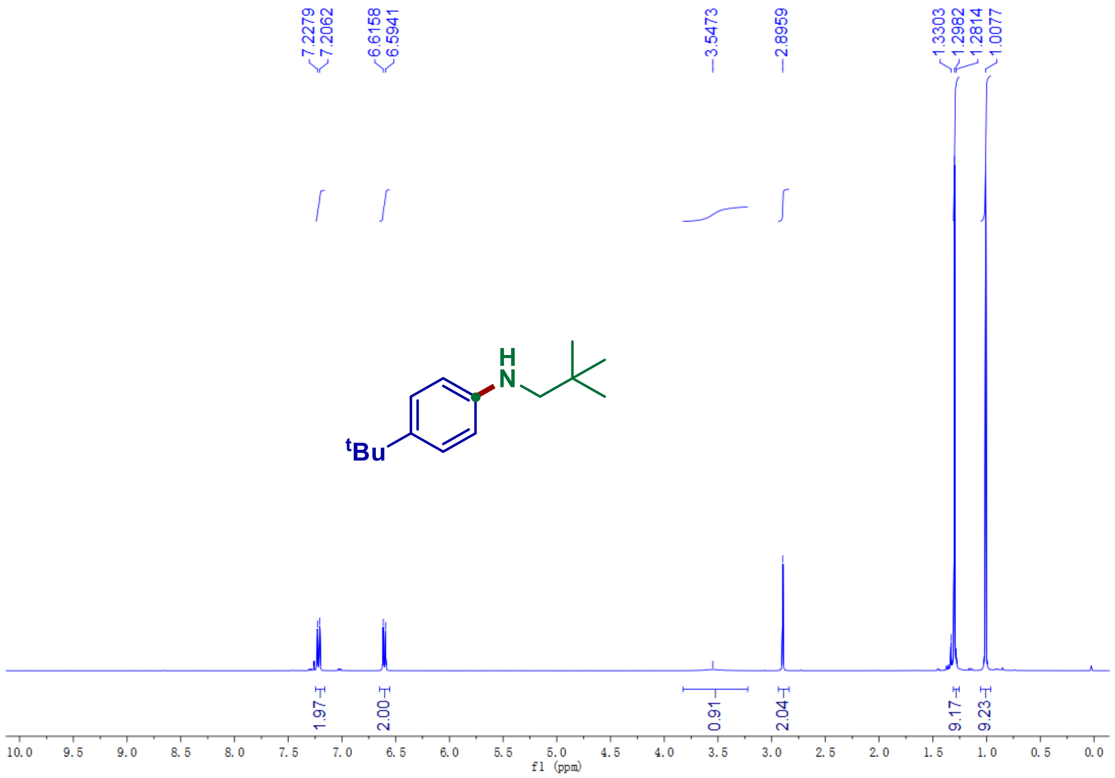

^{1}H NMR (400 MHz, $CDCl_3$) spectrum of compound 80

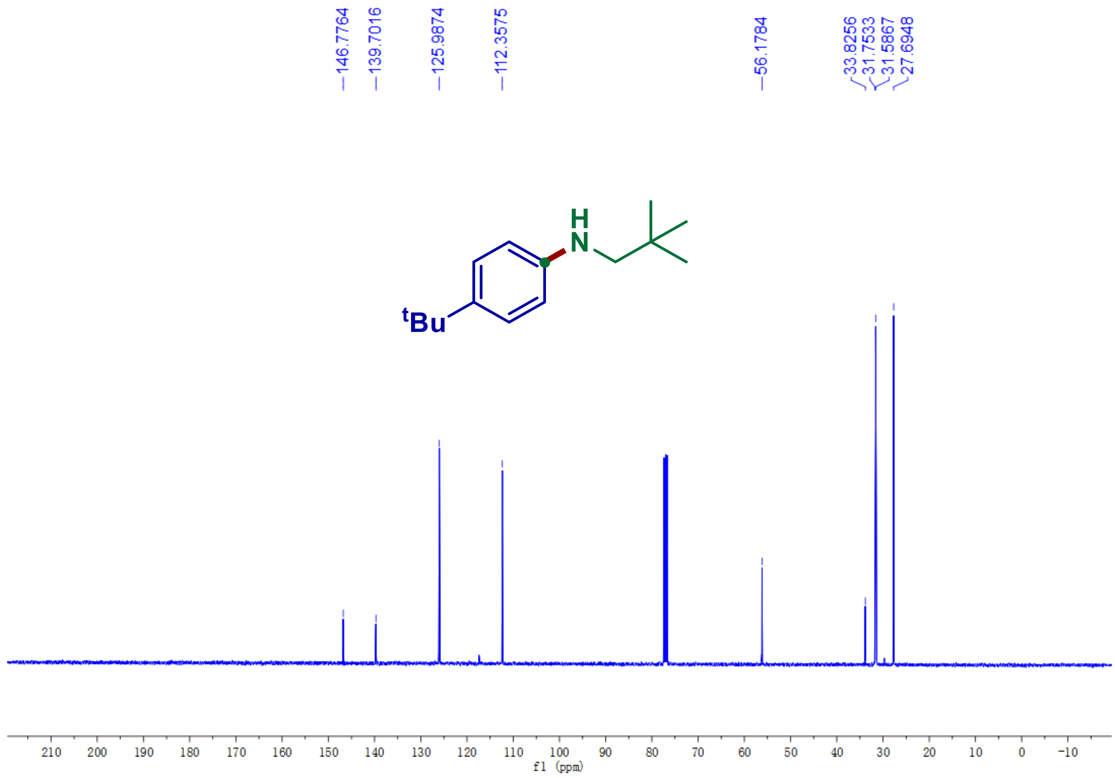

^{13}C NMR (100 MHz, $CDCl_3$) spectrum of compound 80

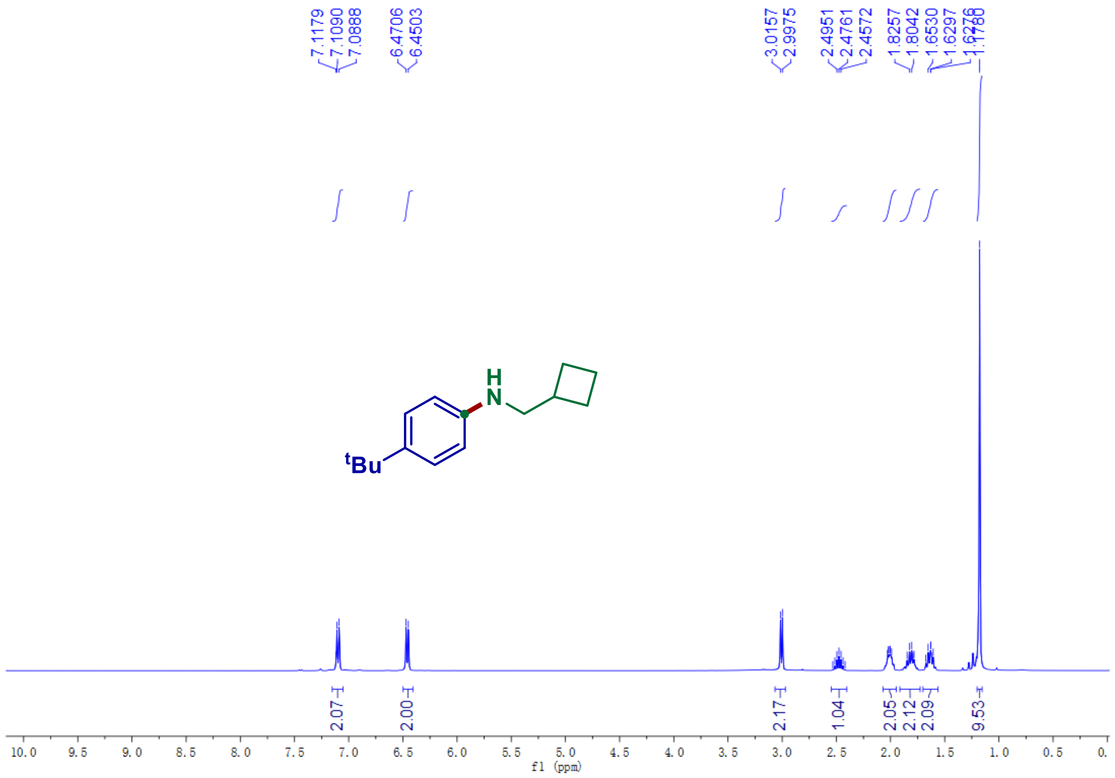

^{1}H NMR (400 MHz, $CDCl_3$) spectrum of compound 81

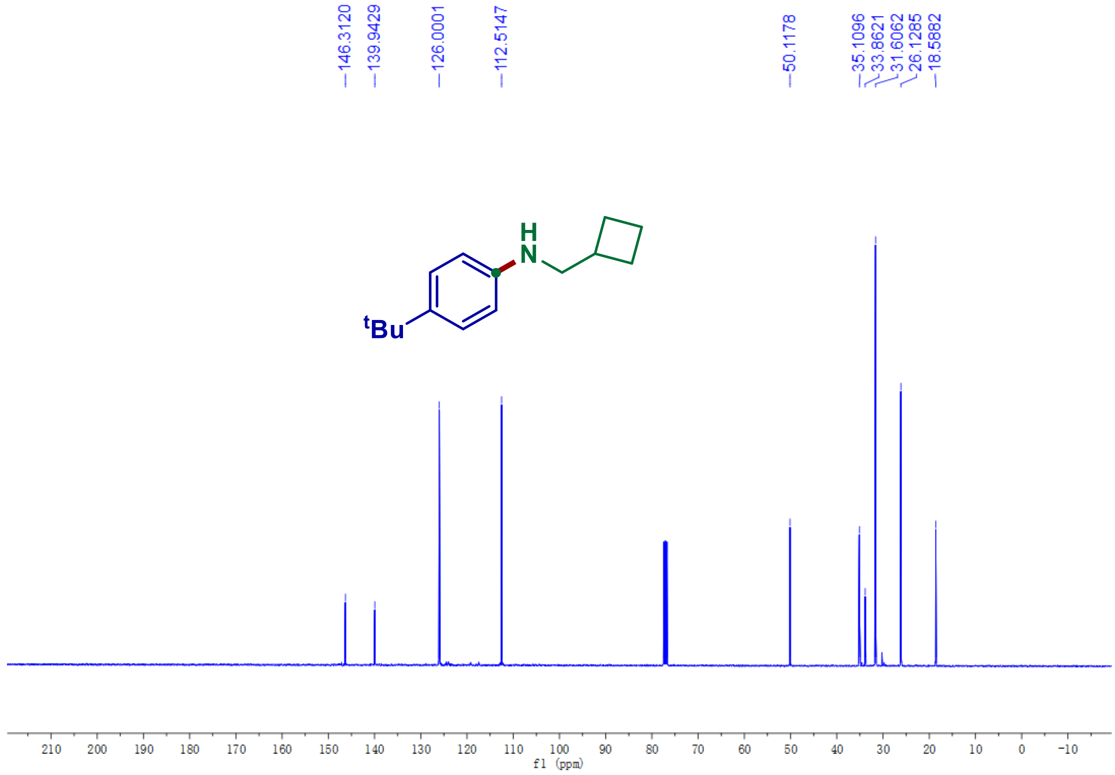

^{13}C NMR (100 MHz, $CDCl_3$) spectrum of compound 81

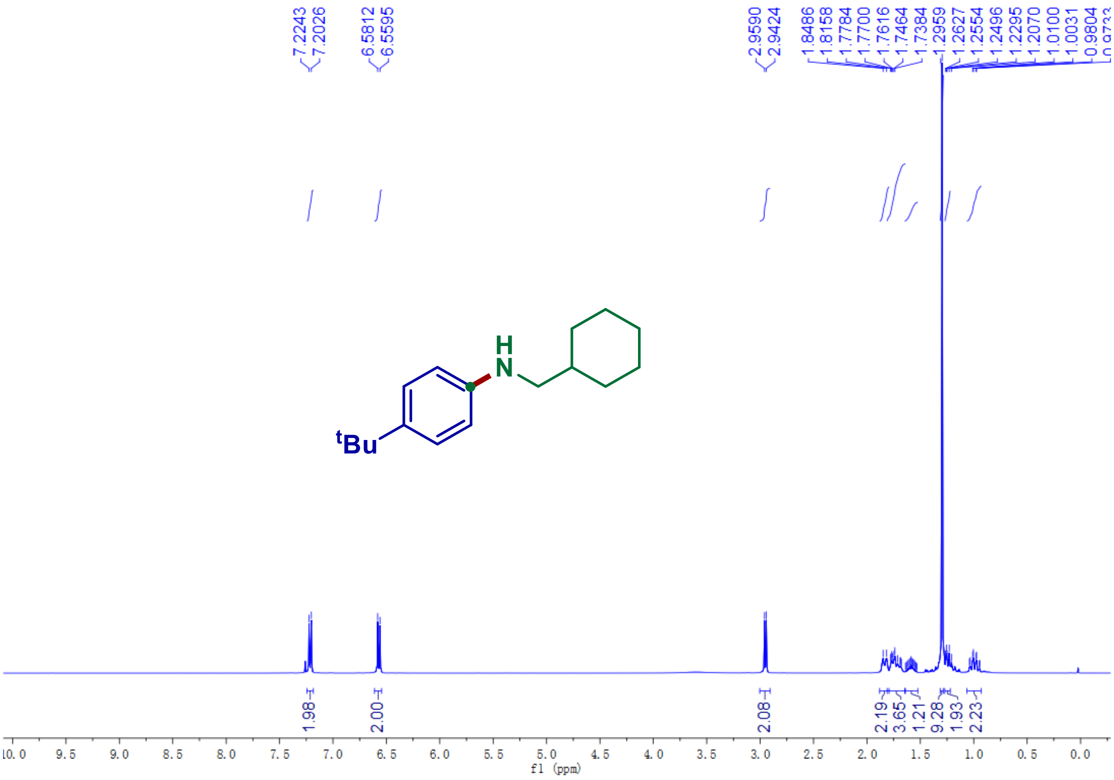

^{1}H NMR (400 MHz, $CDCl_3$) spectrum of compound 82

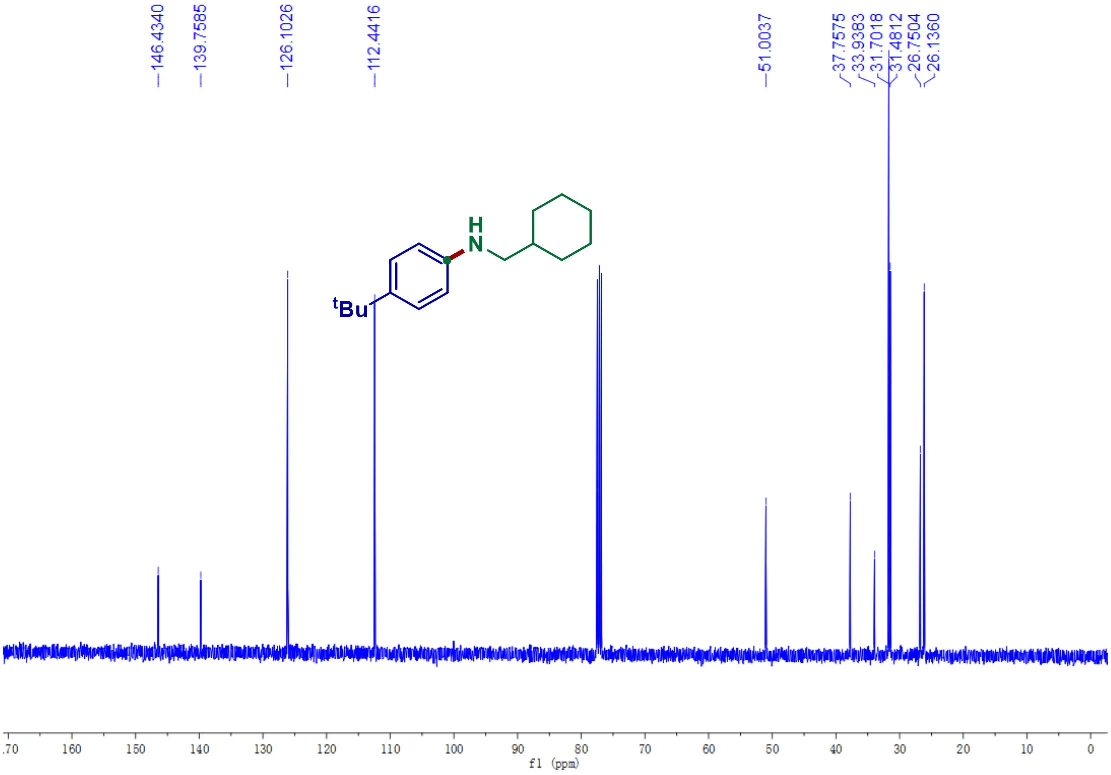

^{13}C NMR (100 MHz, $CDCl_3$) spectrum of compound 82

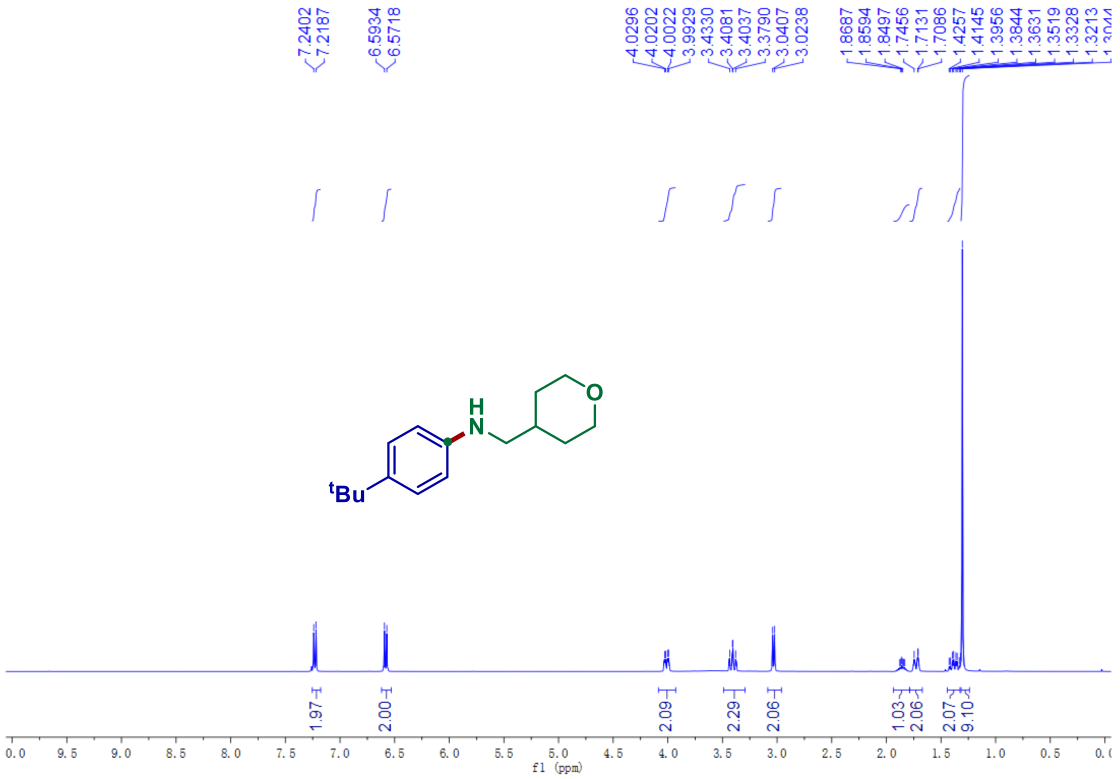

^{1}H NMR (400 MHz, $CDCl_3$) spectrum of compound 83

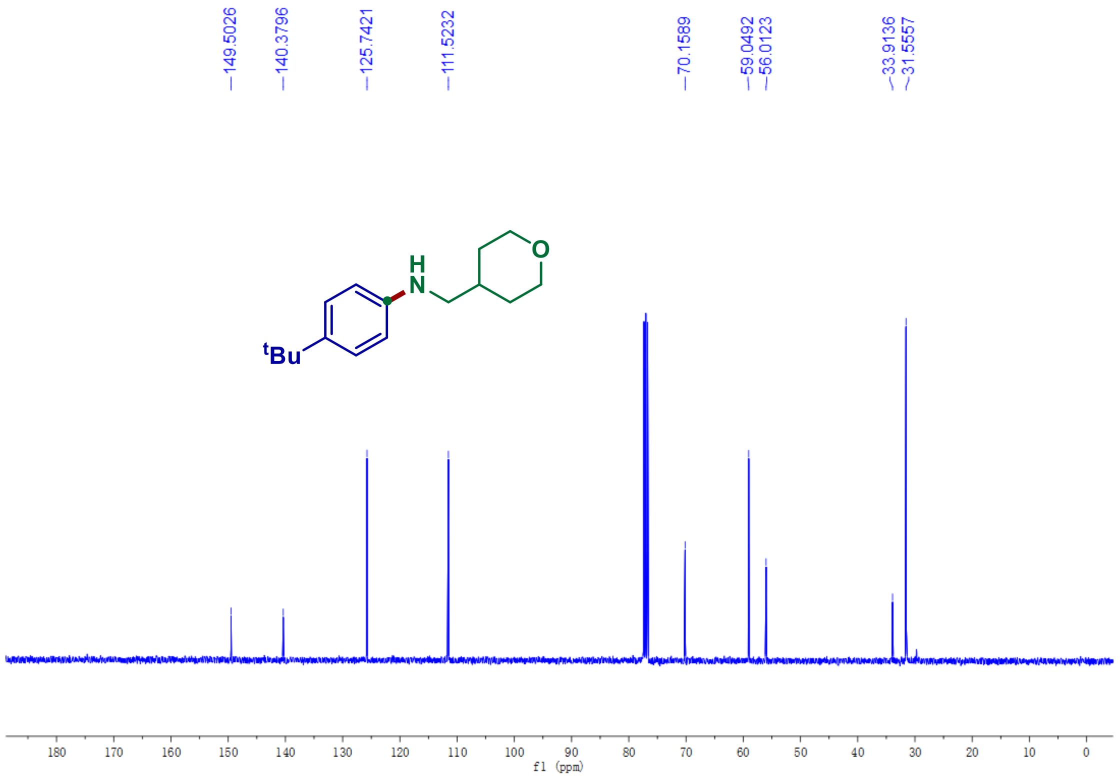

^{13}C NMR (100 MHz, $CDCl_3$) spectrum of compound 83

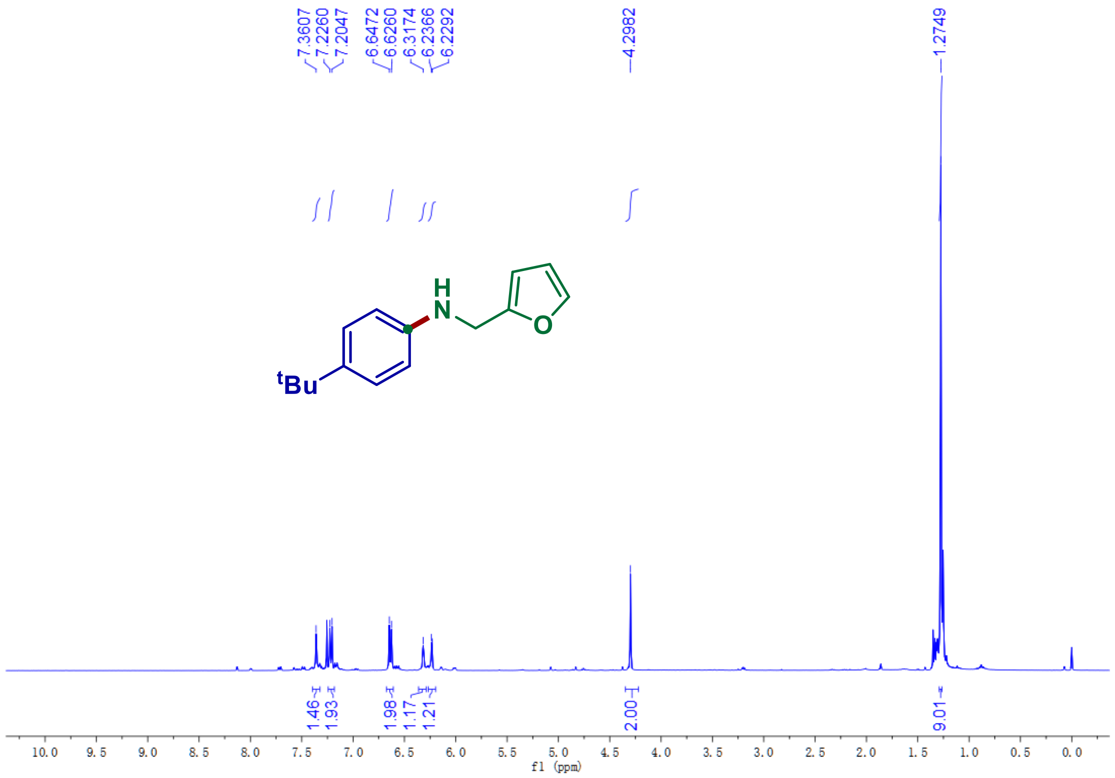

^1H NMR (400 MHz, $CDCl_3$) spectrum of compound 84

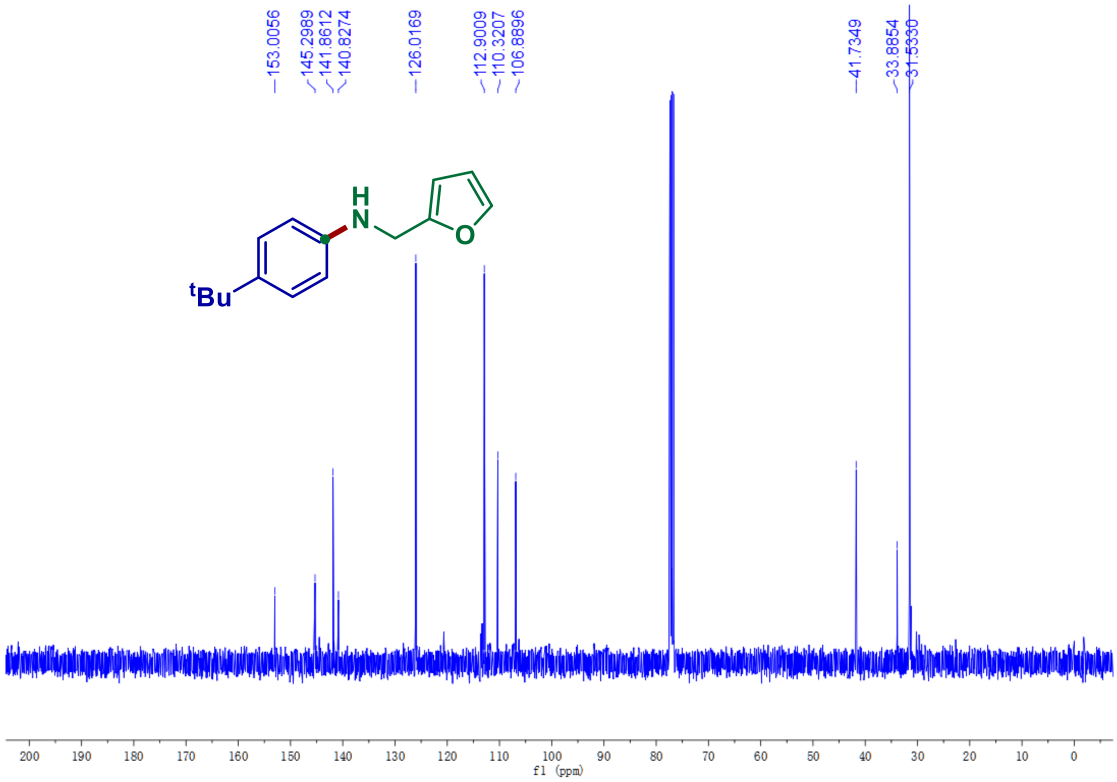

^{13}C NMR (100 MHz, $CDCl_3$) spectrum of compound 84

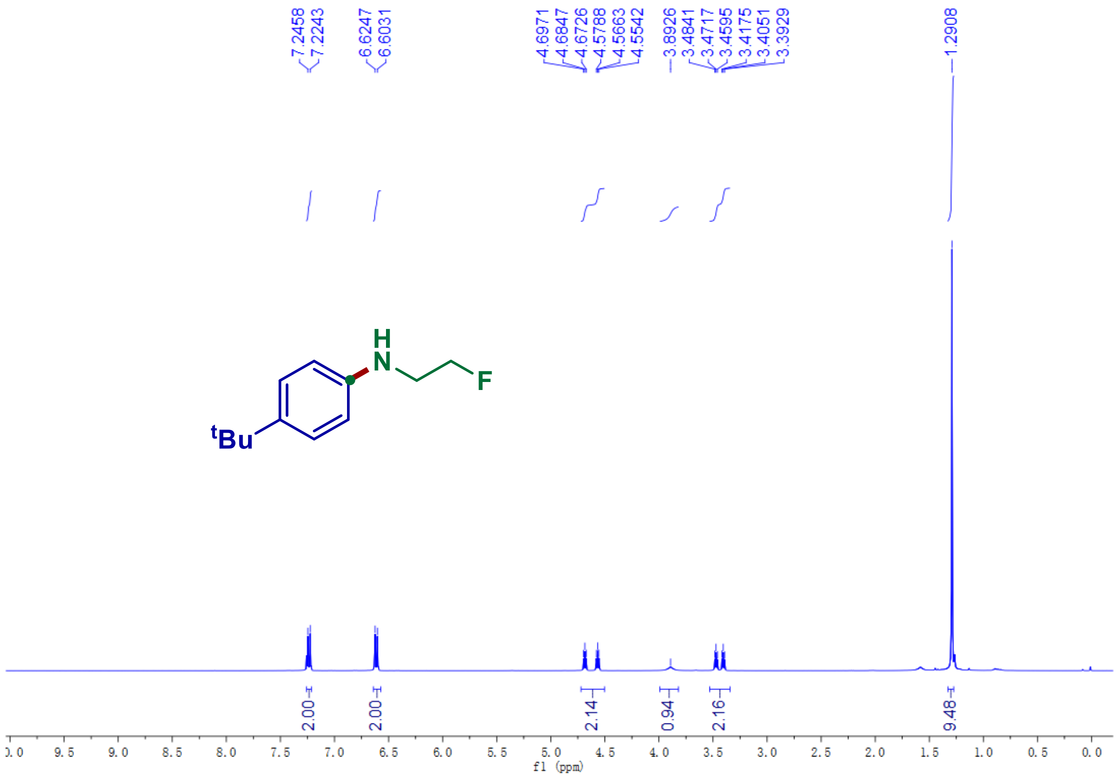

^{1}H NMR (400 MHz, $CDCl_3$) spectrum of compound 85

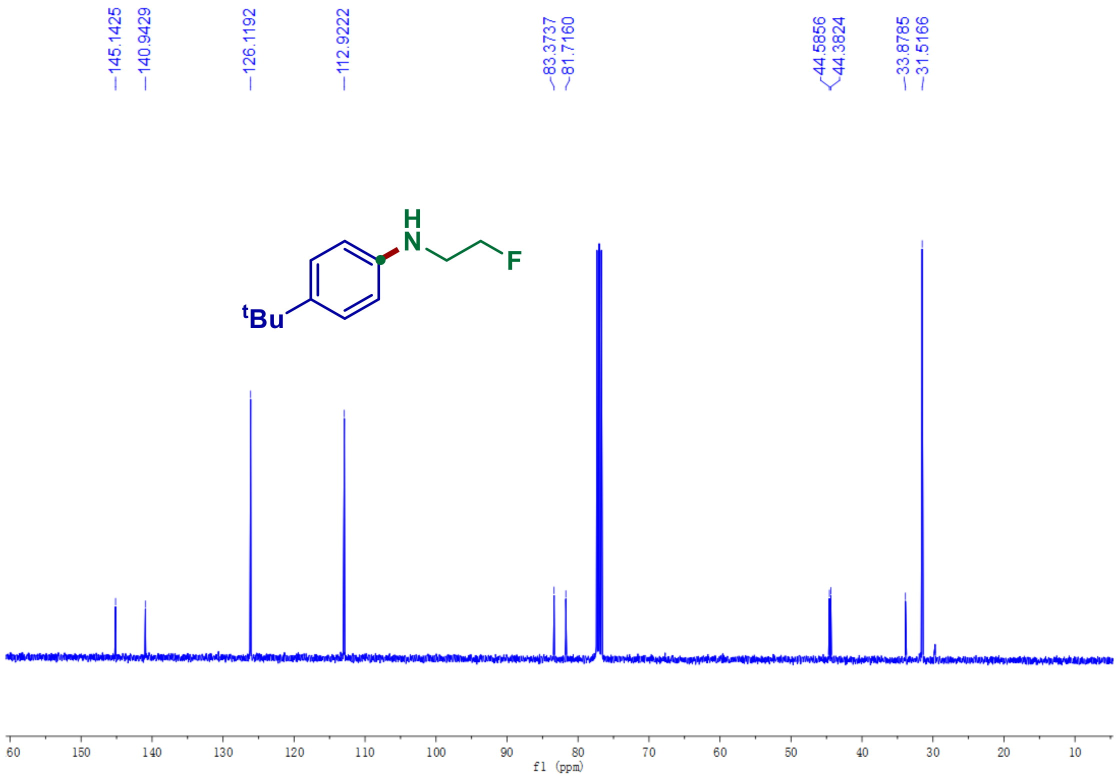

^{13}C NMR (100 MHz, $CDCl_3$) spectrum of compound 85

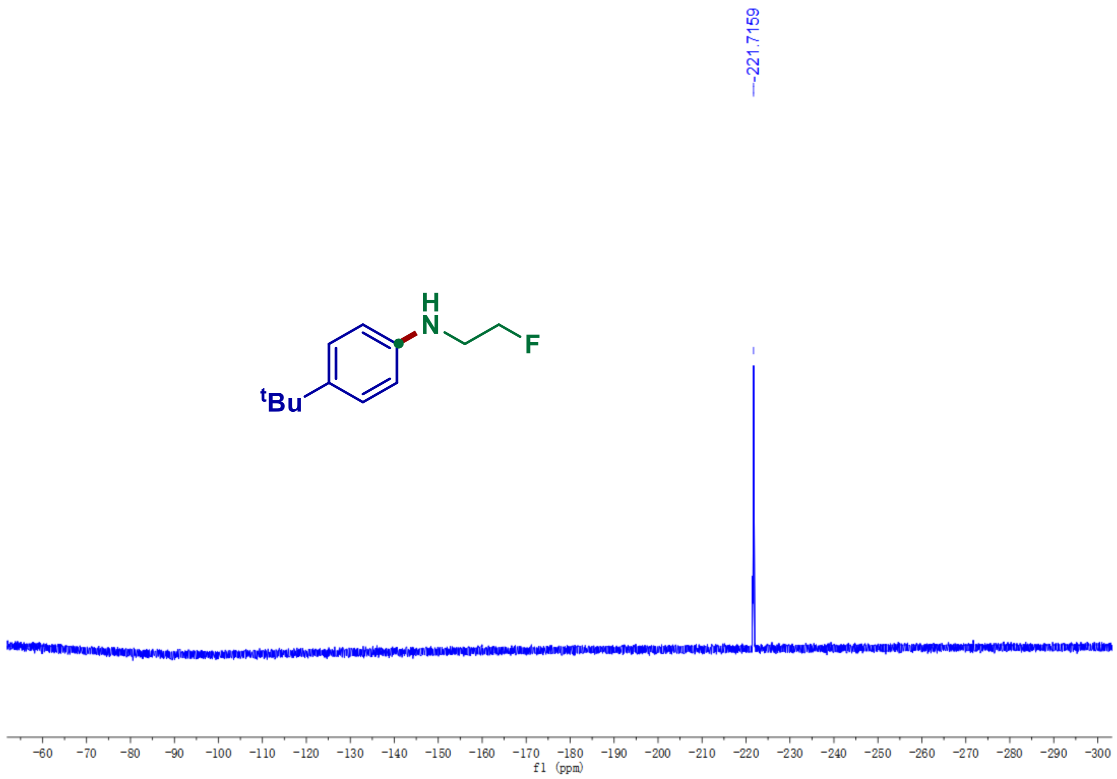

^{19}F NMR (376 MHz, $CDCl_3$) spectrum of compound 85

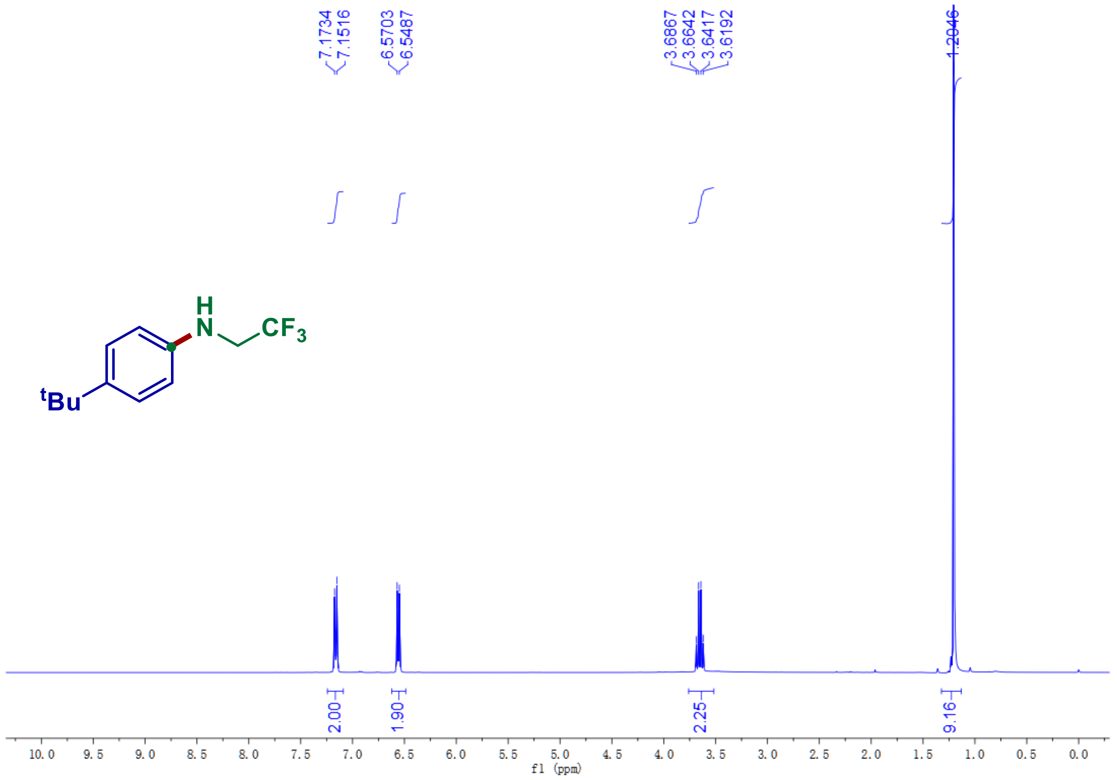

^{1}H NMR (400 MHz, $CDCl_3$) spectrum of compound 86

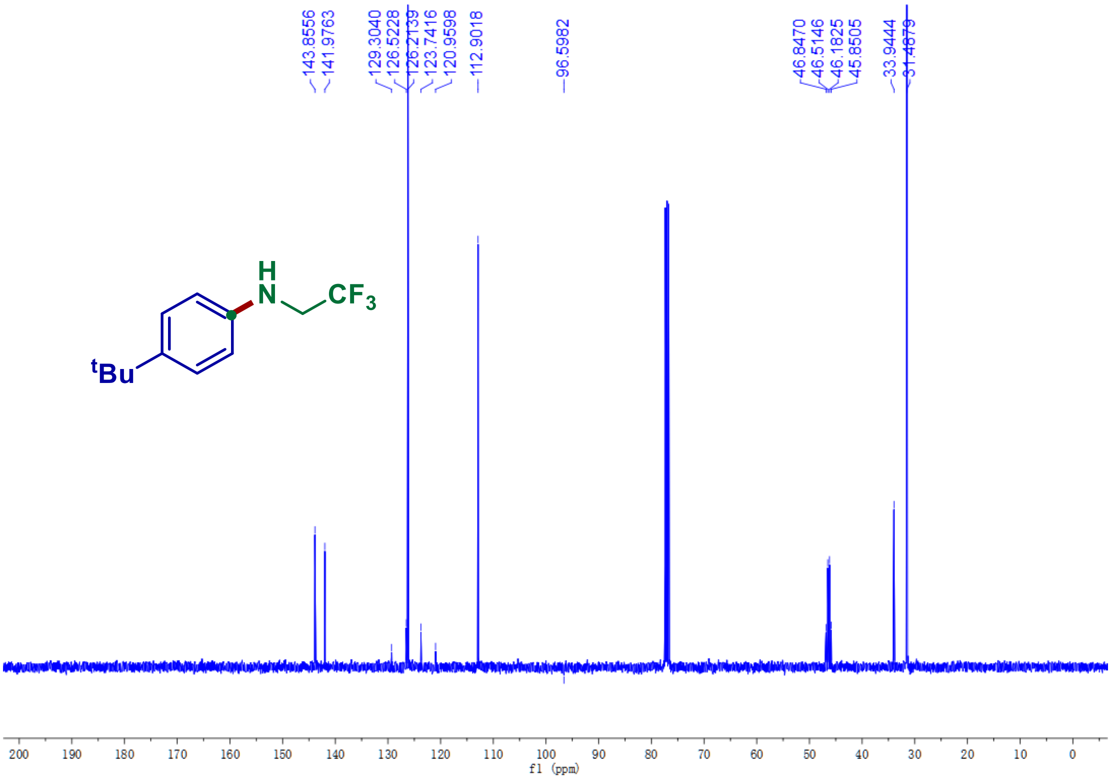

^{13}C NMR (100 MHz, $CDCl_3$) spectrum of compound 86

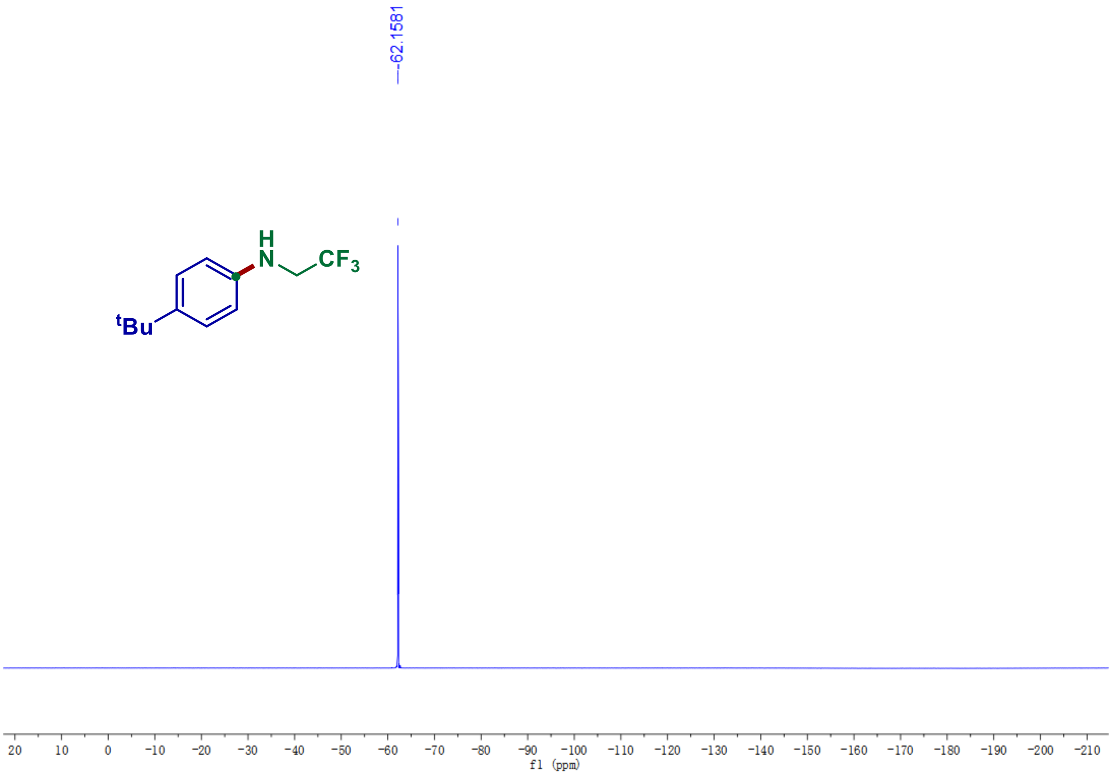

^{19}F NMR (100 MHz, $CDCl_3$) spectrum of compound 86

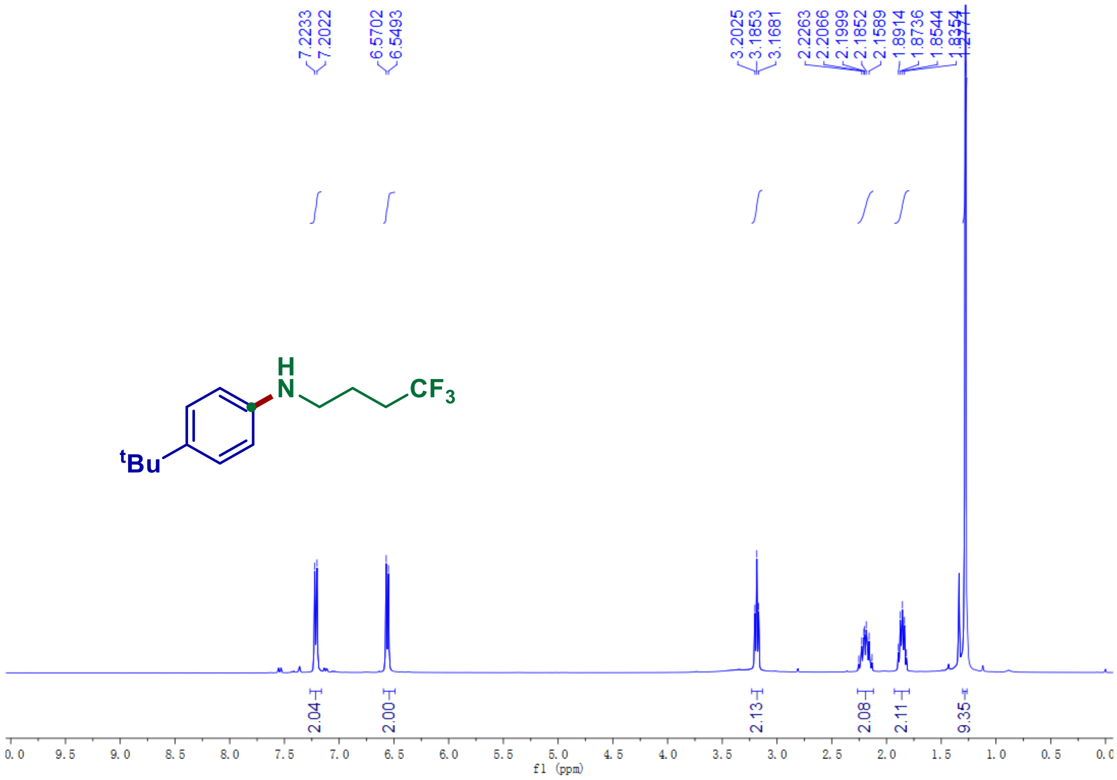

^{1}H NMR (400 MHz, $CDCl_3$) spectrum of compound 87

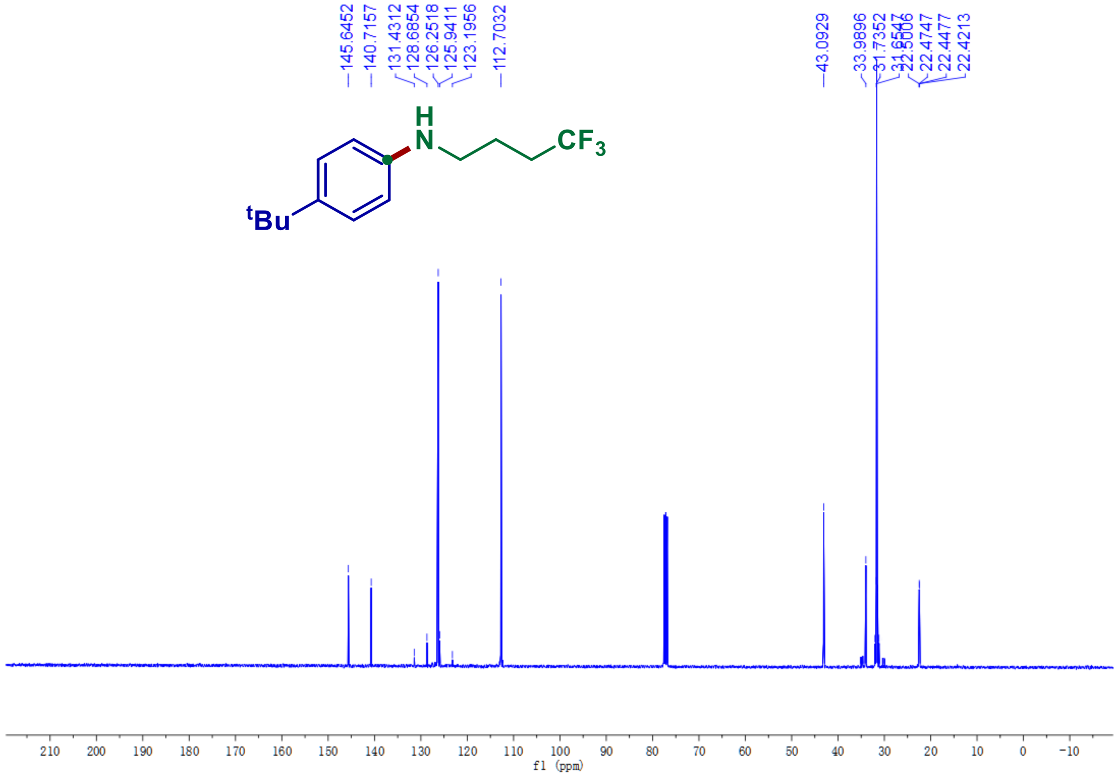

^{13}C NMR (100 MHz, $CDCl_3$) spectrum of compound 87

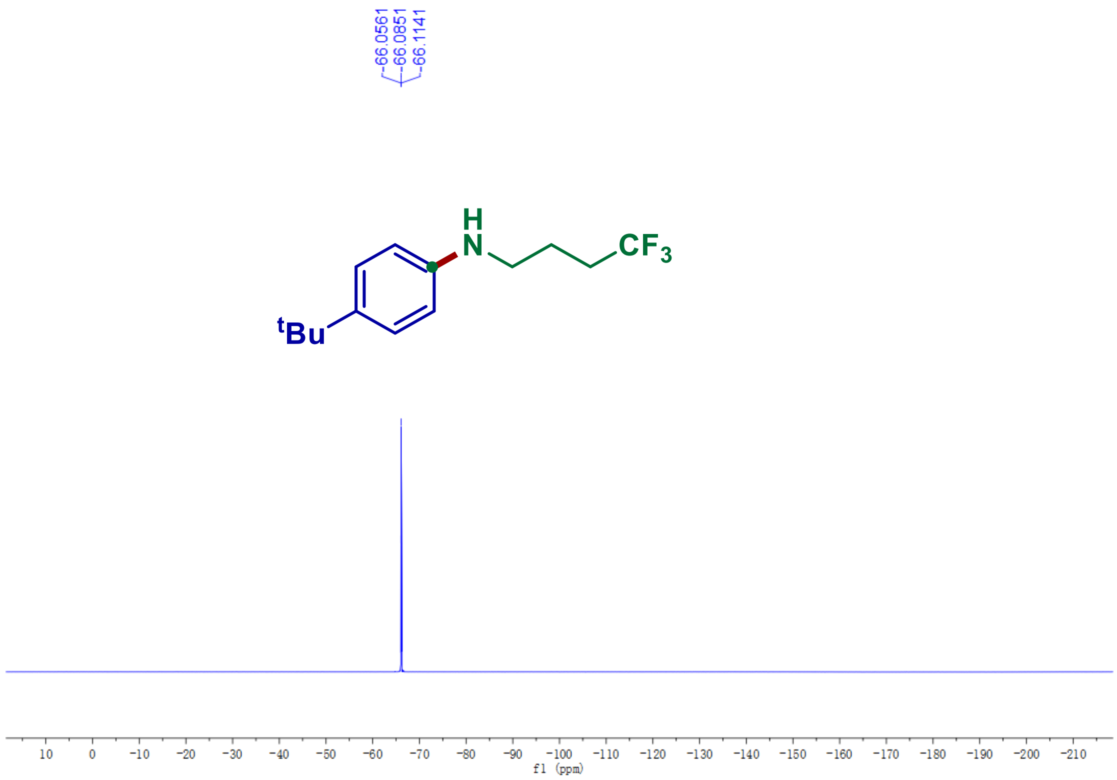

^{19}F NMR (376 MHz, $CDCl_3$) spectrum of compound 87

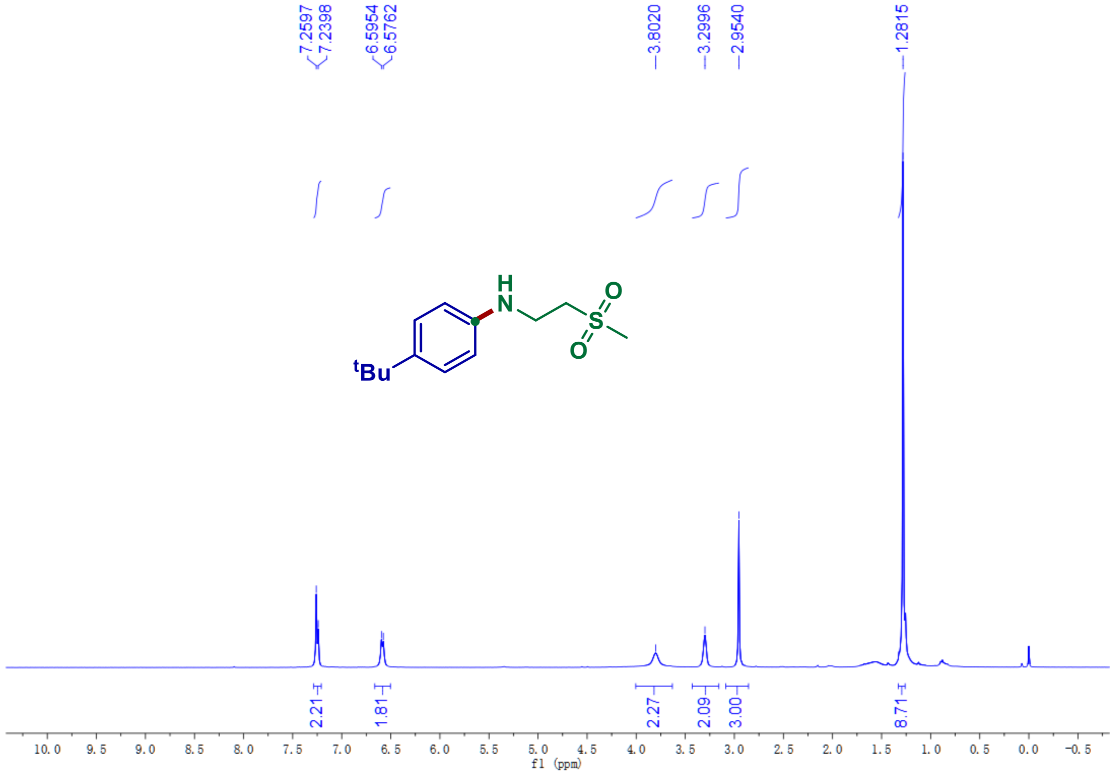

^{1}H NMR (400 MHz, $CDCl_3$) spectrum of compound 88

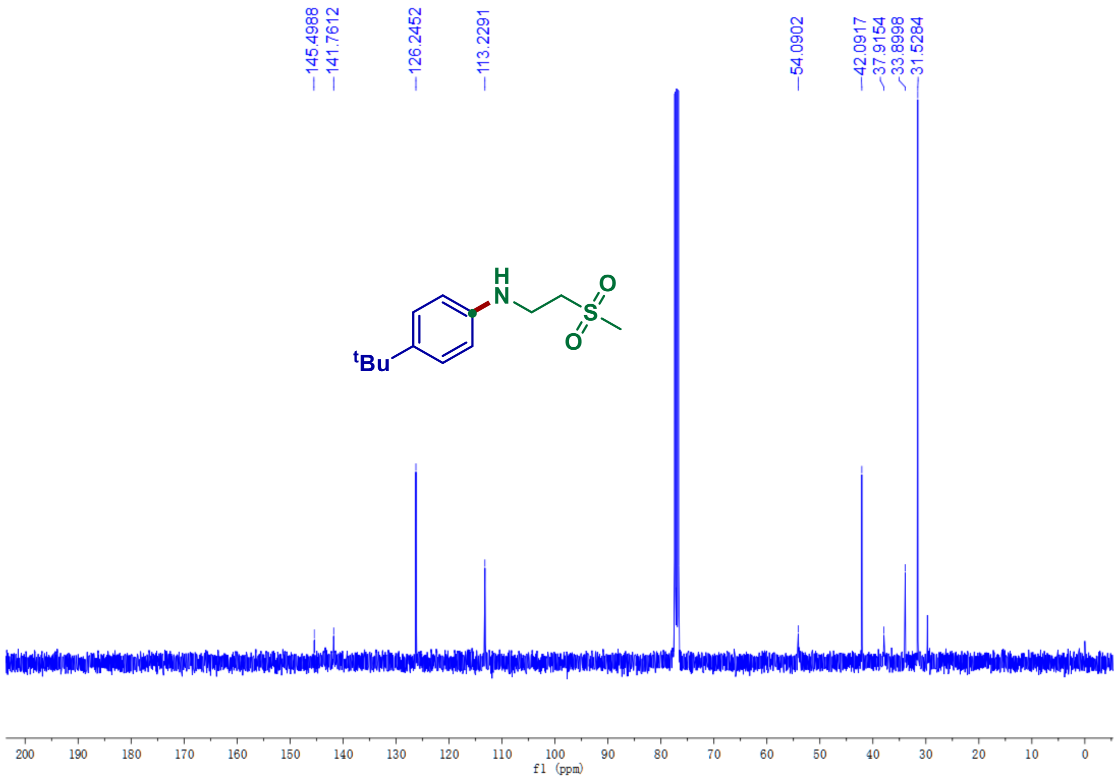

^{13}C NMR (100 MHz, $CDCl_3$) spectrum of compound 88

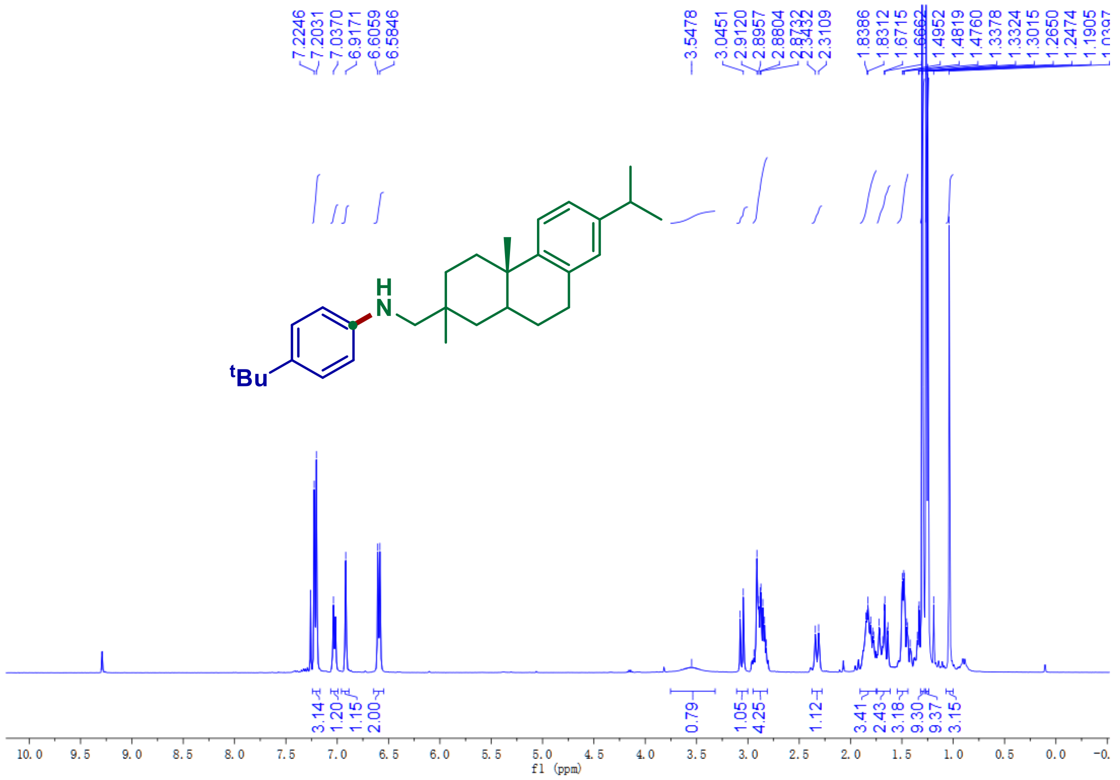

^{1}H NMR (400 MHz, $CDCl_3$) spectrum of compound 89

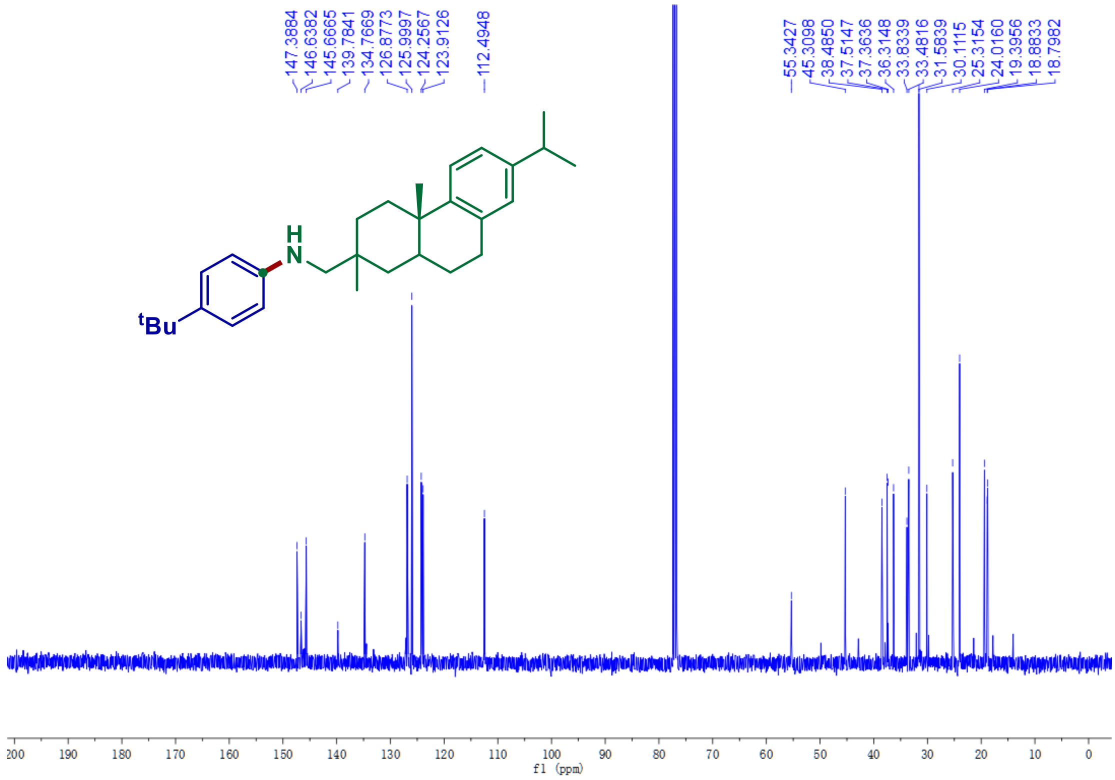

^{13}C NMR (100 MHz, $CDCl_3$) spectrum of compound 89

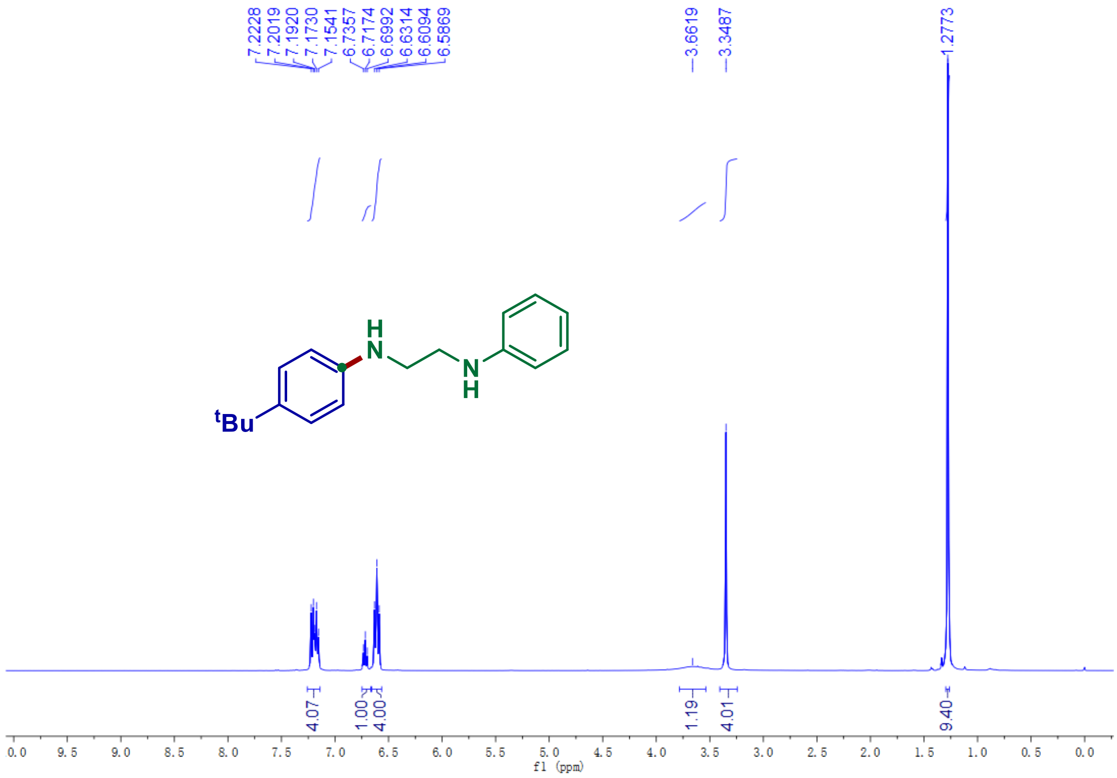

^{1}H NMR (400 MHz, $CDCl_3$) spectrum of compound 90

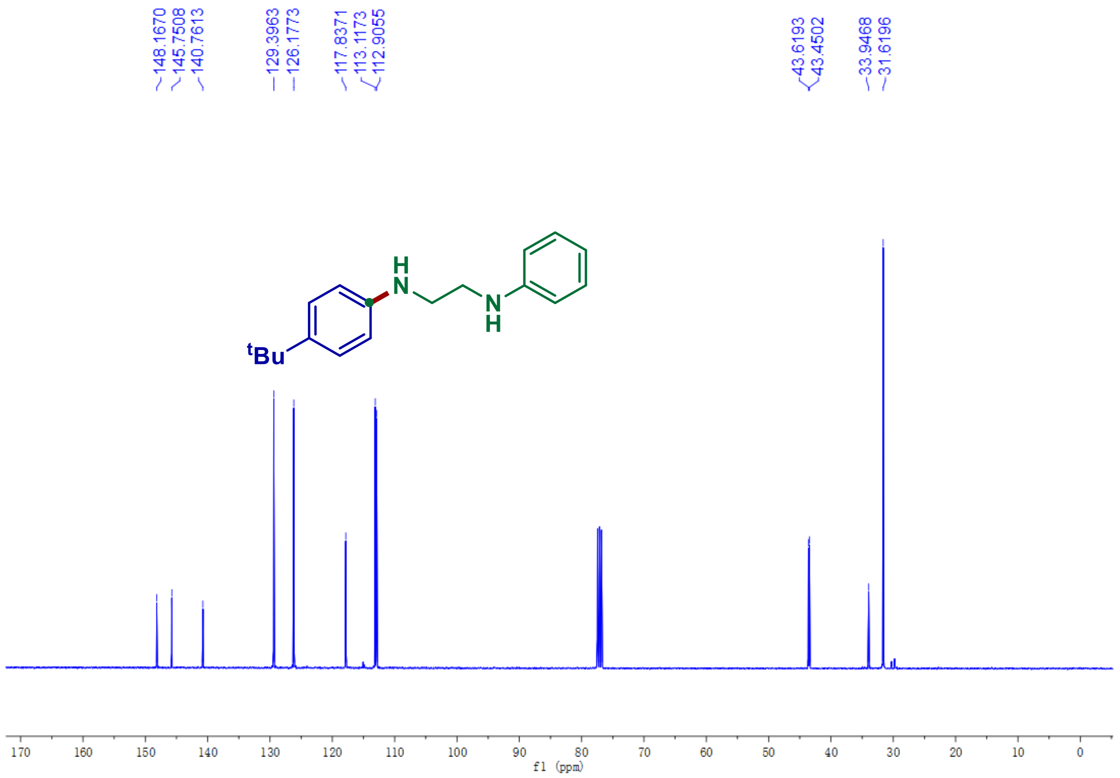

^{13}C NMR (100 MHz, CDCl$_3$) spectrum of compound 90

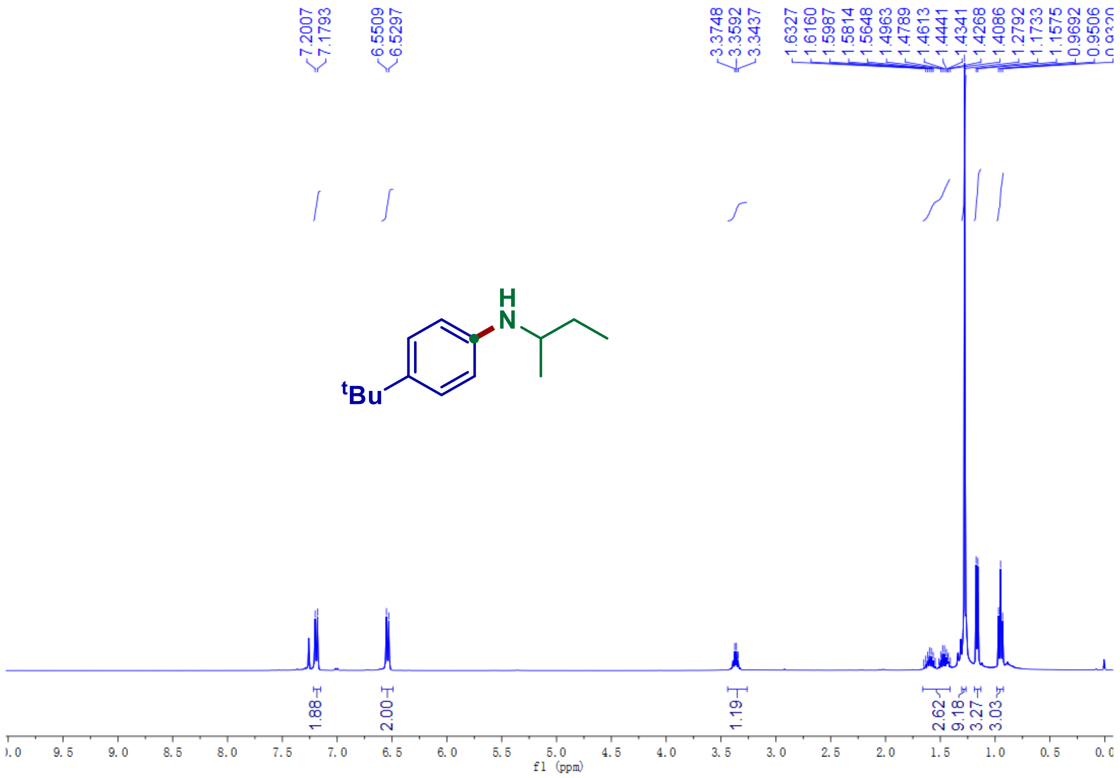

^{1}H NMR (400 MHz, CDCl$_3$) spectrum of compound 91

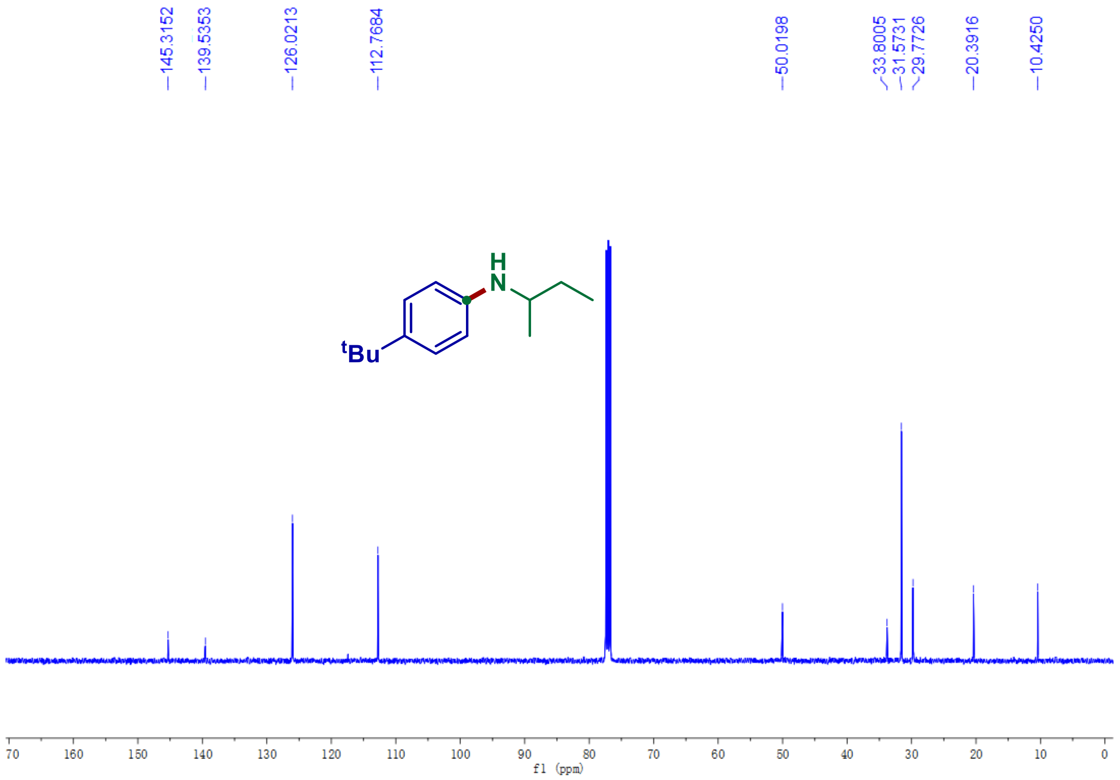

^{13}C NMR (100 MHz, $CDCl_3$) spectrum of compound 91

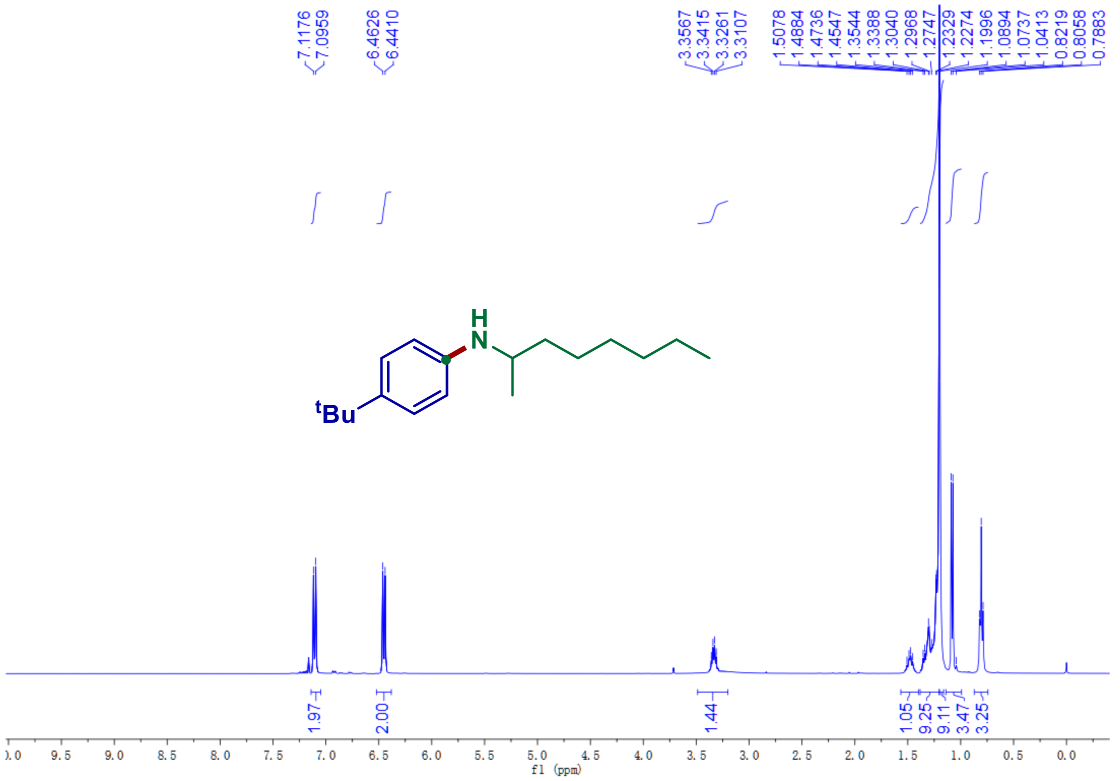

^{1}H NMR (400 MHz, $CDCl_3$) spectrum of compound 92

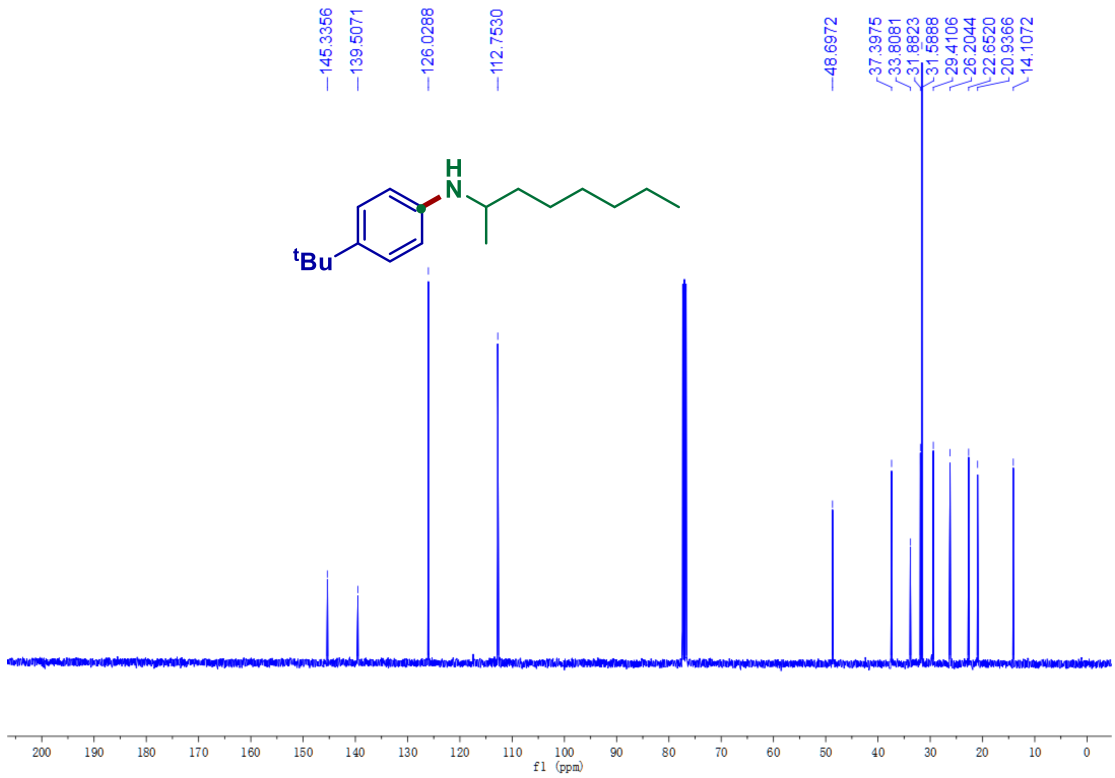

^{13}C NMR (100 MHz, $CDCl_3$) spectrum of compound 92

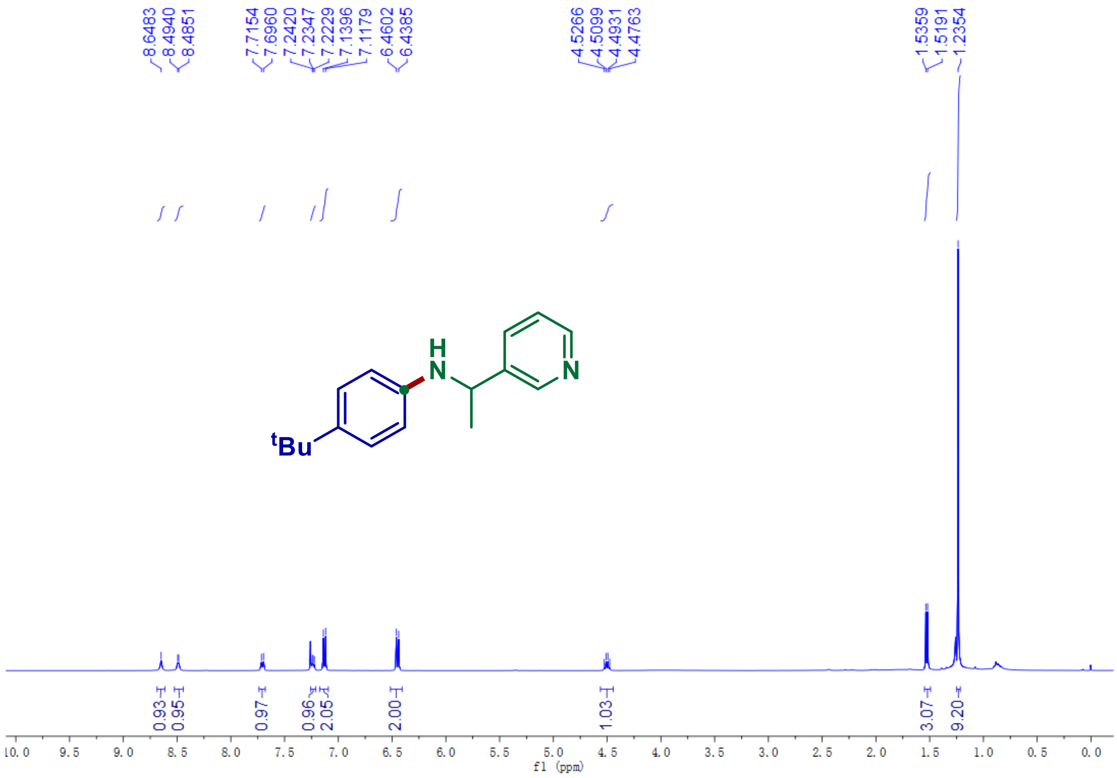

^{1}H NMR (400 MHz, $CDCl_3$) spectrum of compound 93

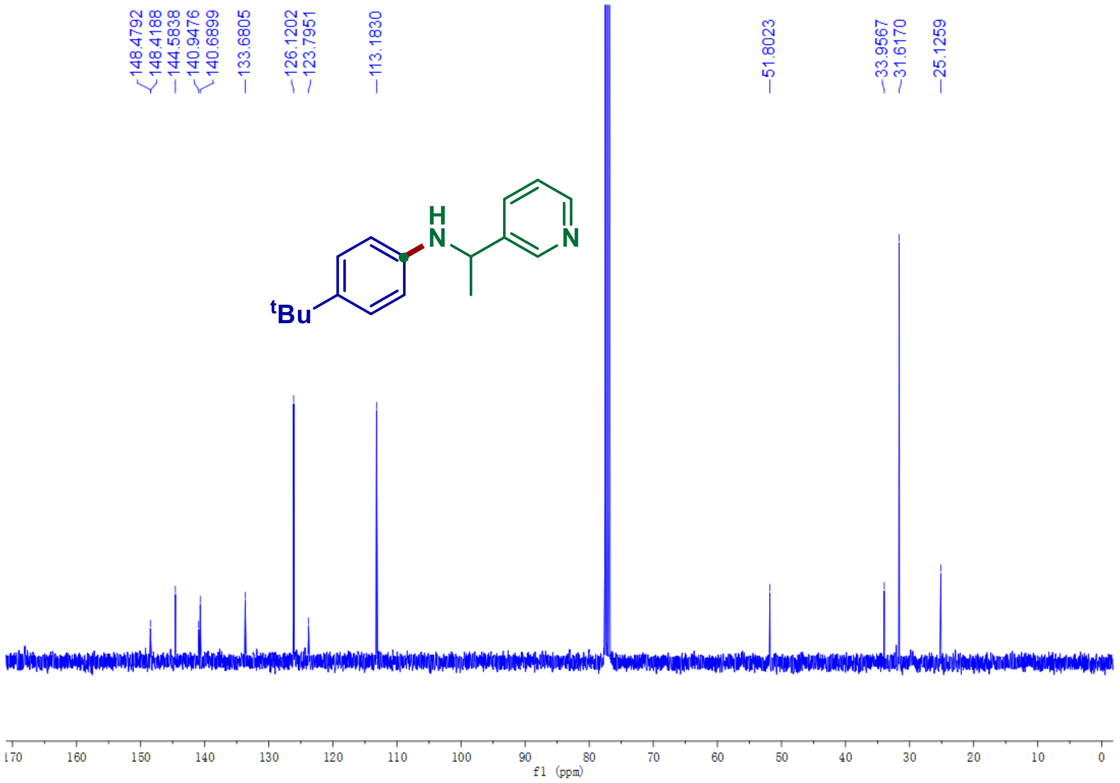

^{13}C NMR (100 MHz, $CDCl_3$) spectrum of compound 93

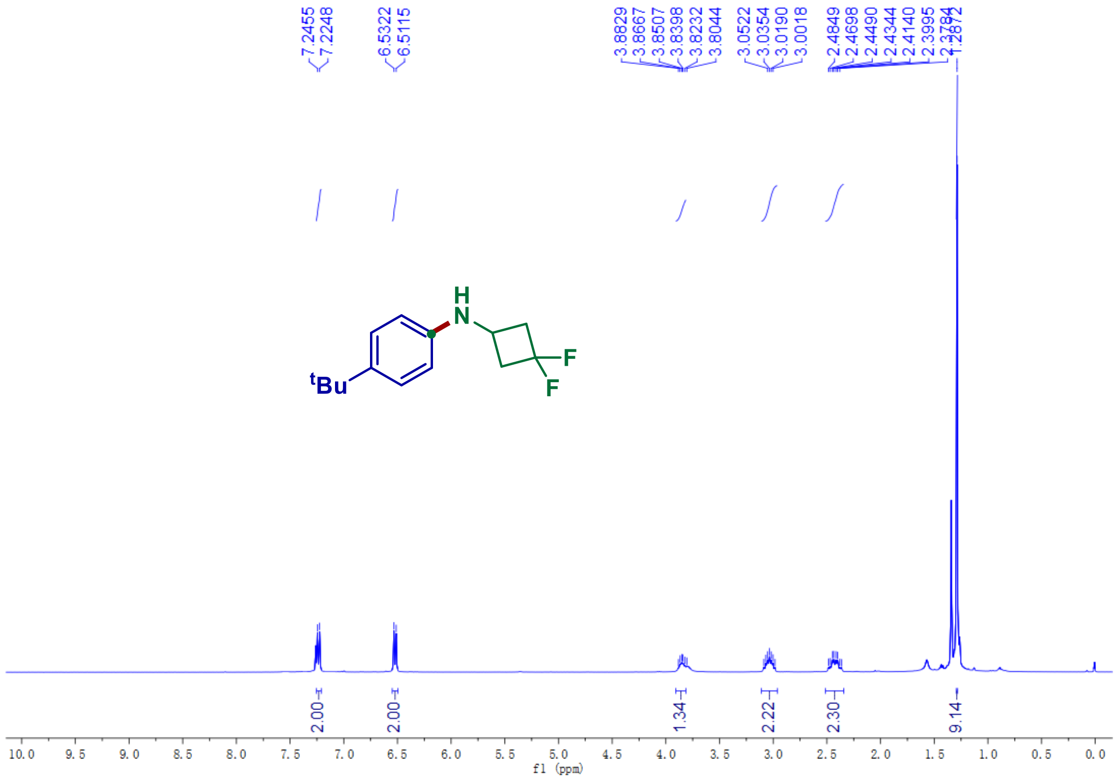

^{1}H NMR (400 MHz, $CDCl_3$) spectrum of compound 94

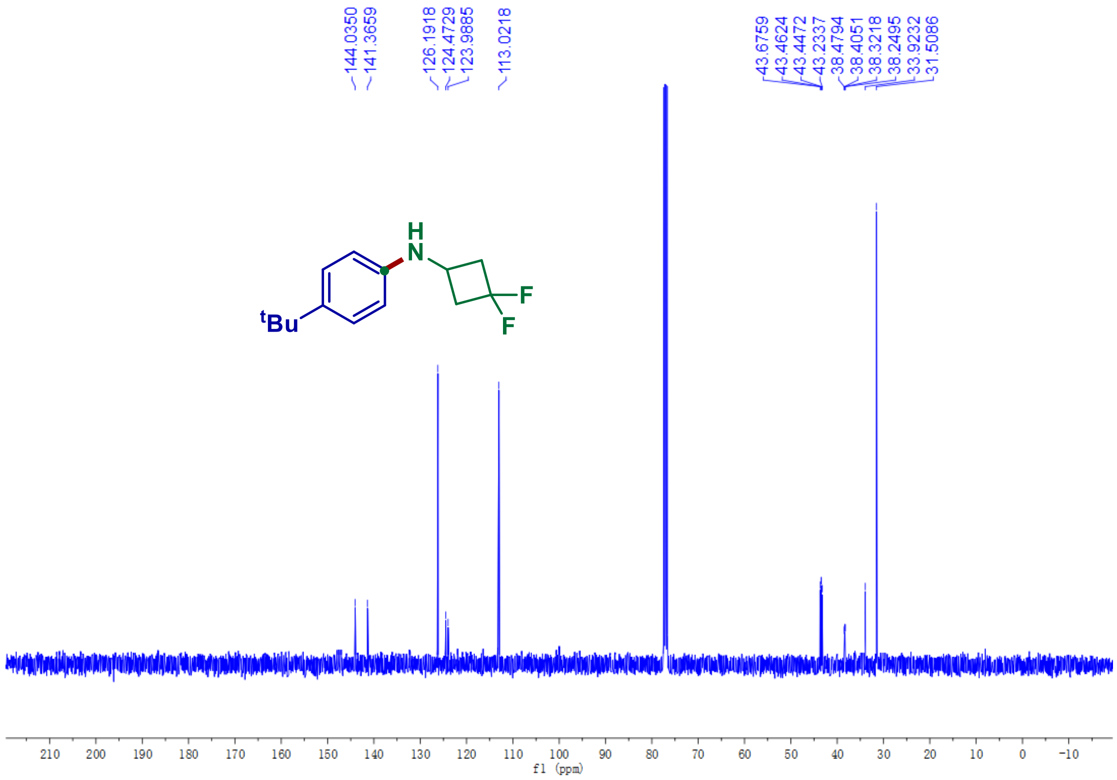

^{13}C NMR (100 MHz, $CDCl_3$) spectrum of compound 94

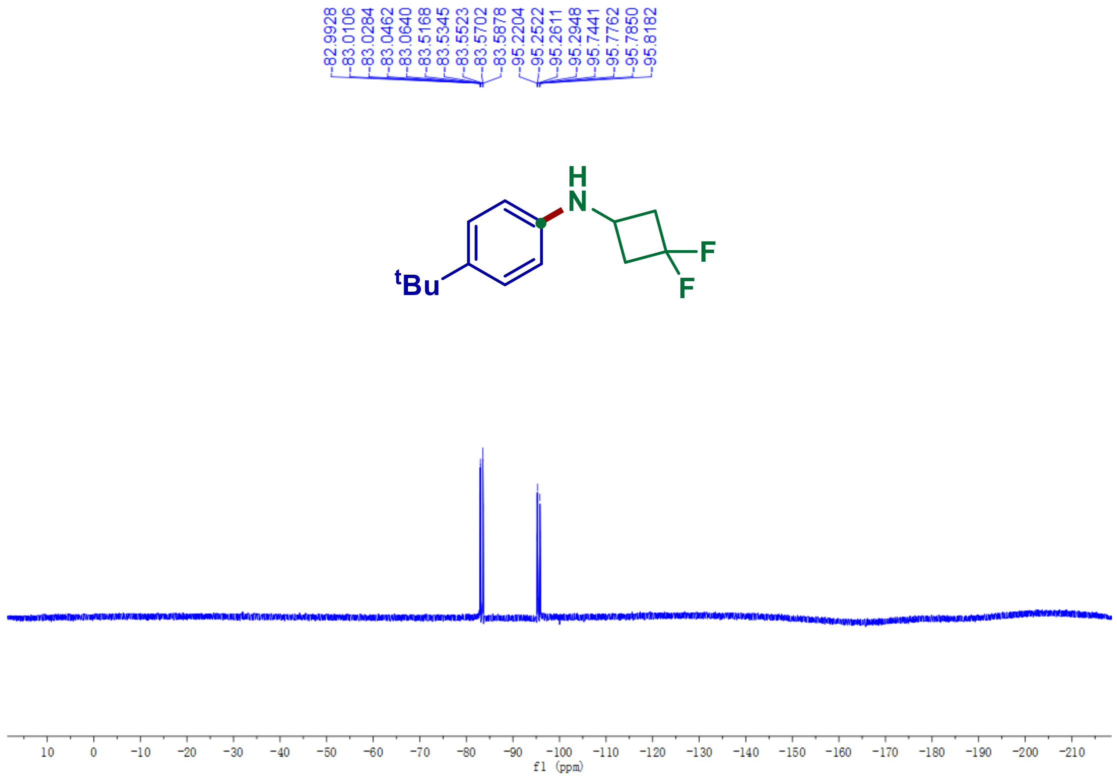

^{19}F NMR (376 MHz, $CDCl_3$) spectrum of compound 94

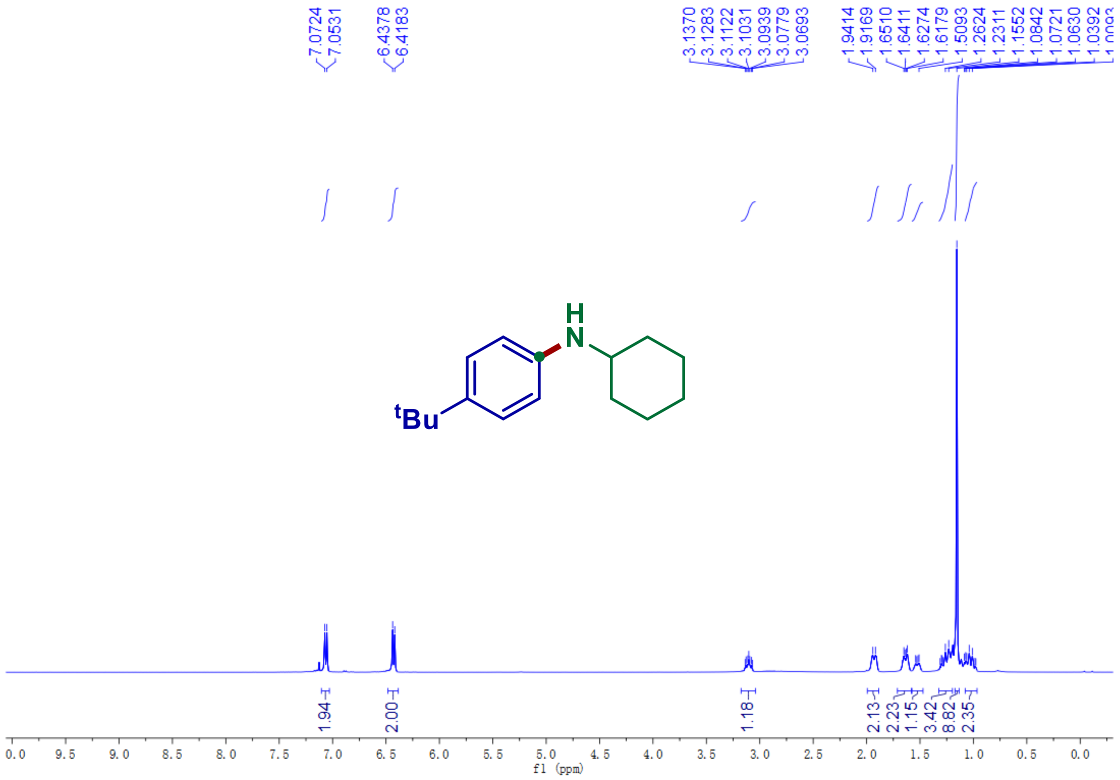

^{1}H NMR (400 MHz, CDCl$_3$) spectrum of compound 95

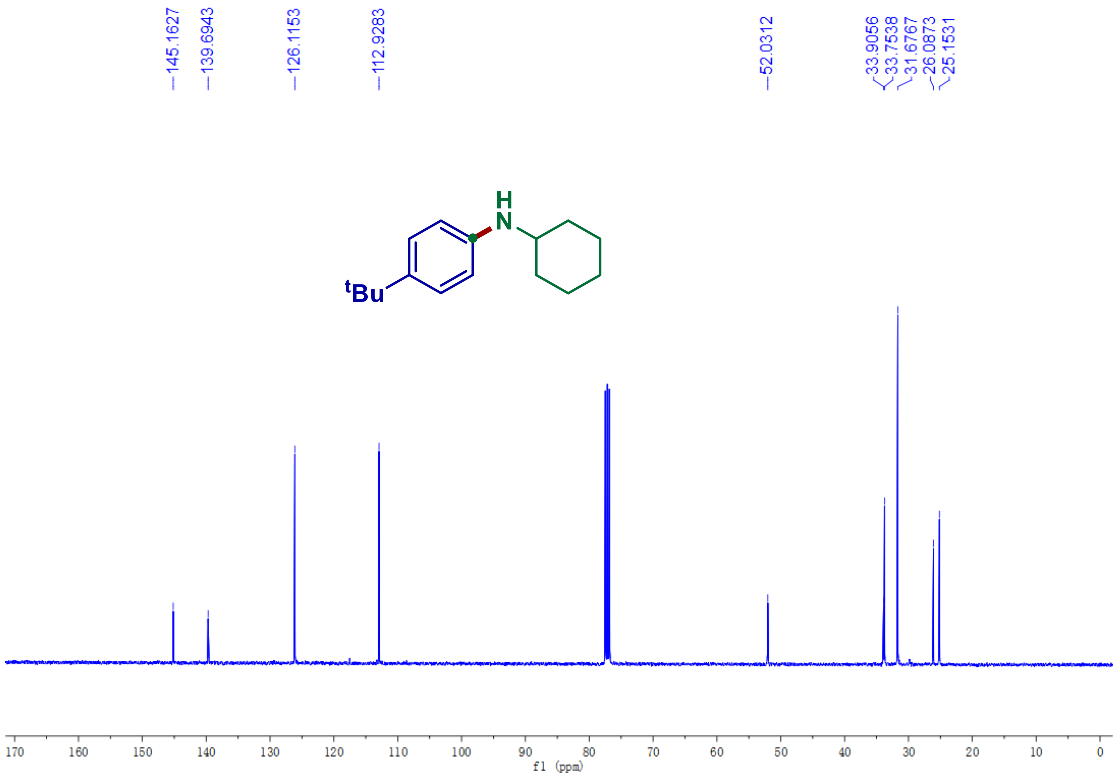

^{13}C NMR (100 MHz, CDCl$_3$) spectrum of compound 95

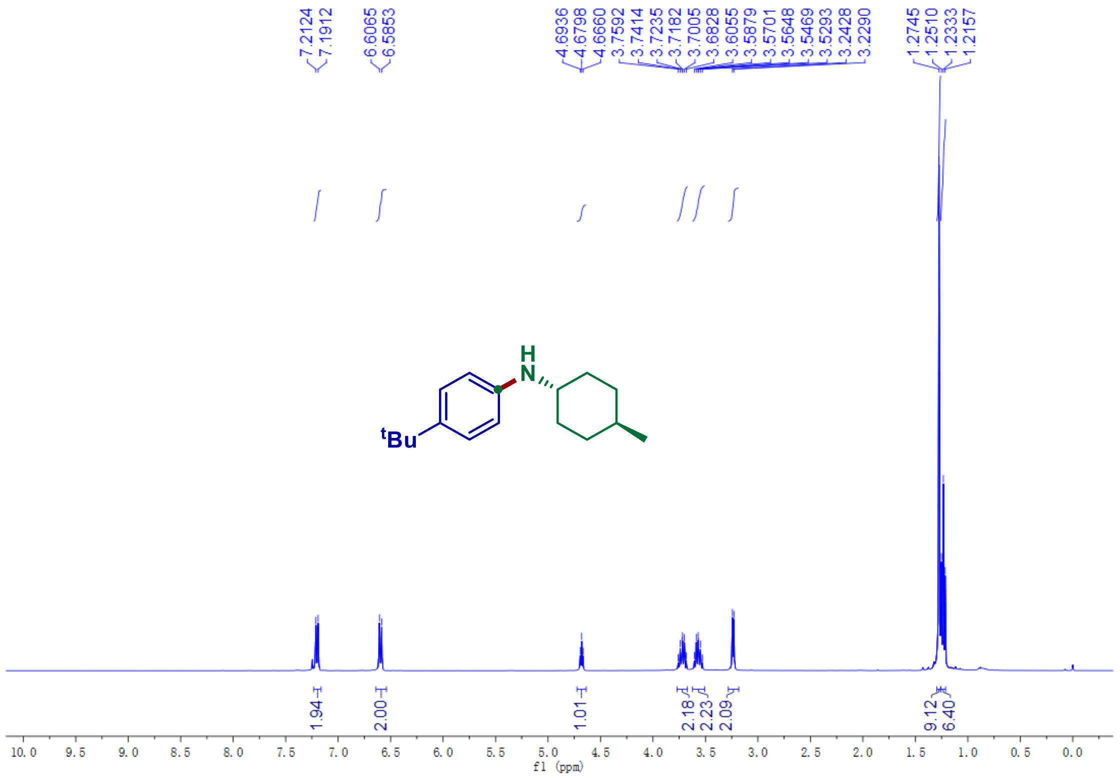

^{1}H NMR (400 MHz, $CDCl_3$) spectrum of compound 96

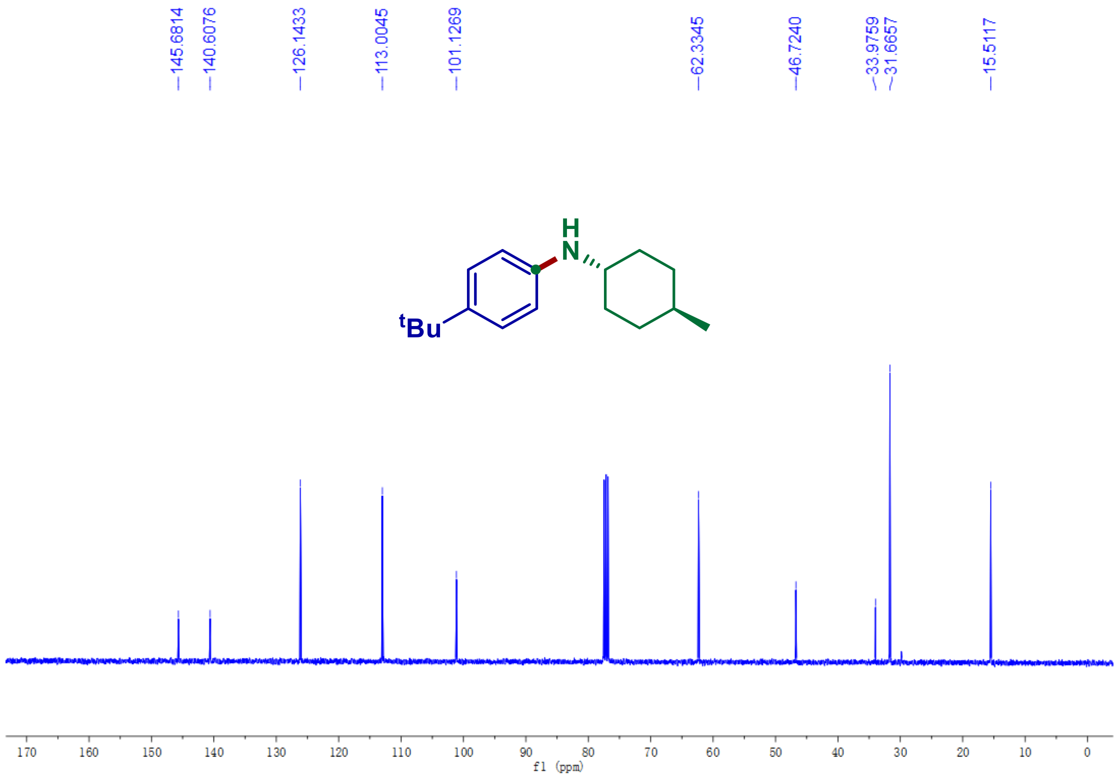

^{13}C NMR (100 MHz, $CDCl_3$) spectrum of compound 96

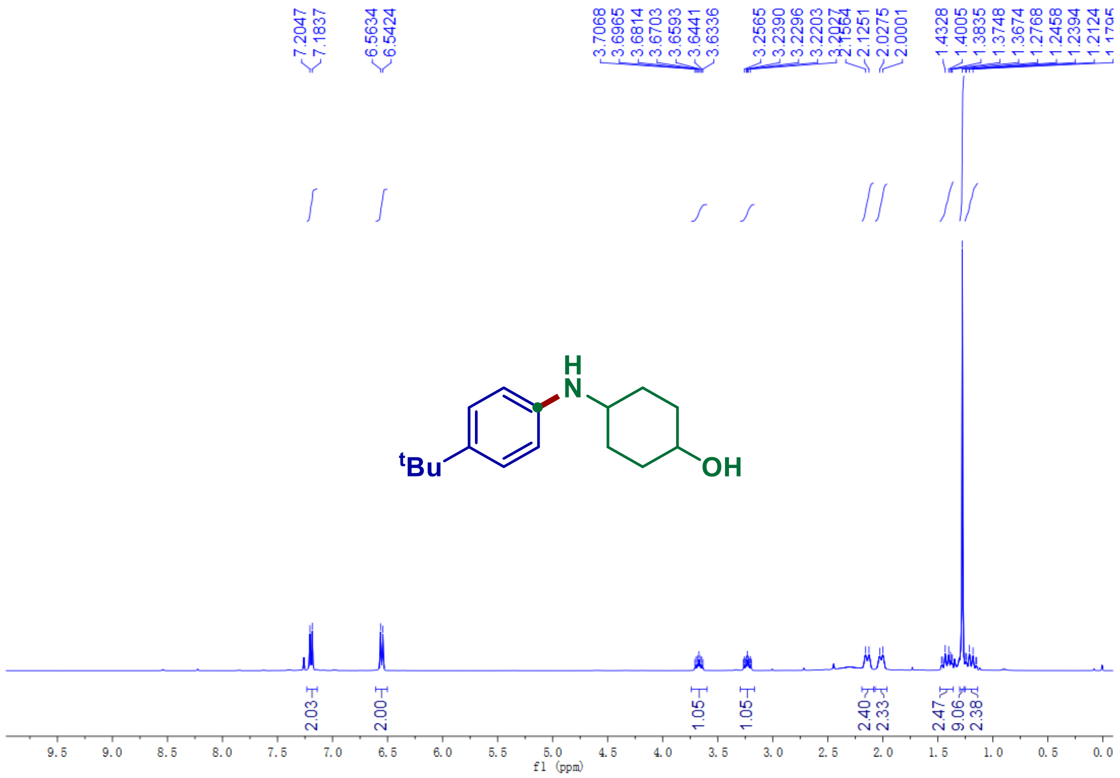

^{1}H NMR (400 MHz, $CDCl_3$) spectrum of compound 97

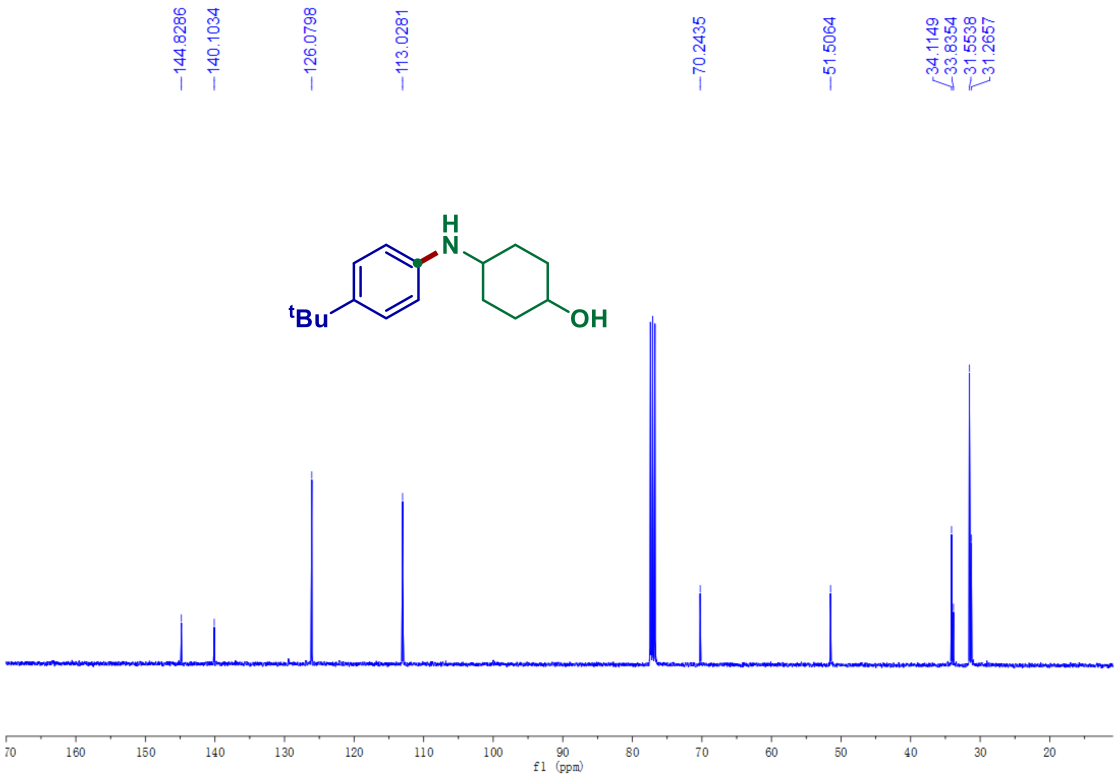

^{13}C NMR (100 MHz, $CDCl_3$) spectrum of compound 97

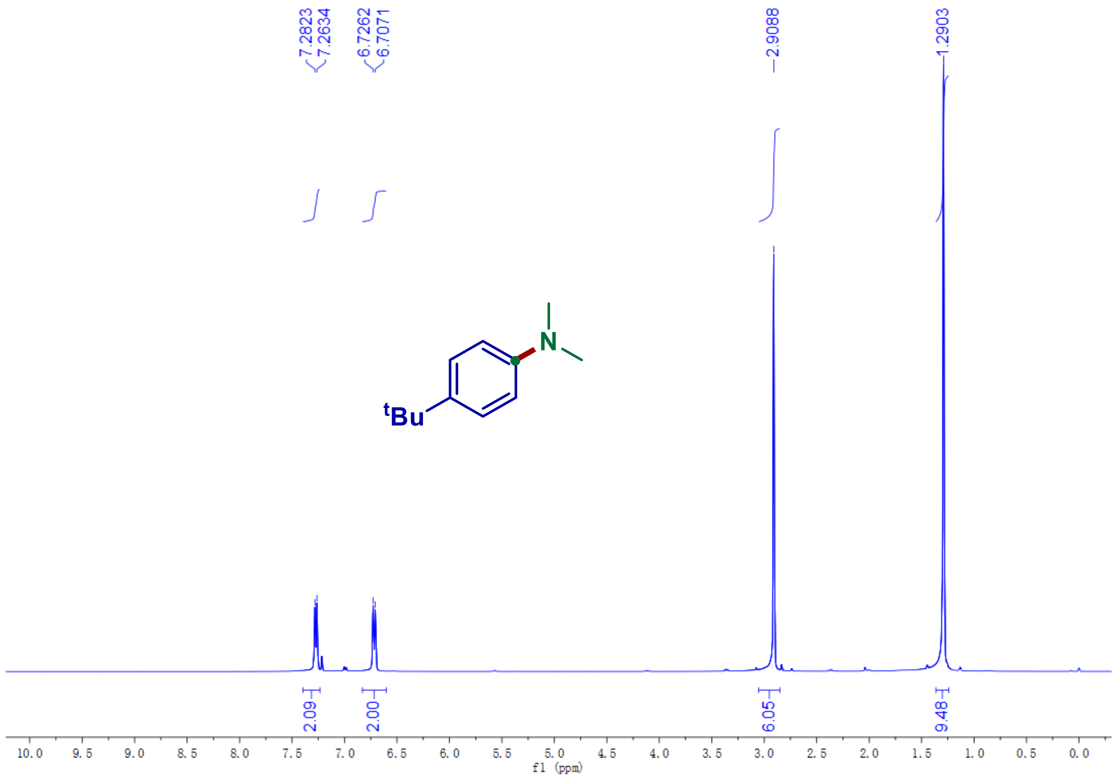

^{1}H NMR (400 MHz, $CDCl_3$) spectrum of compound 98

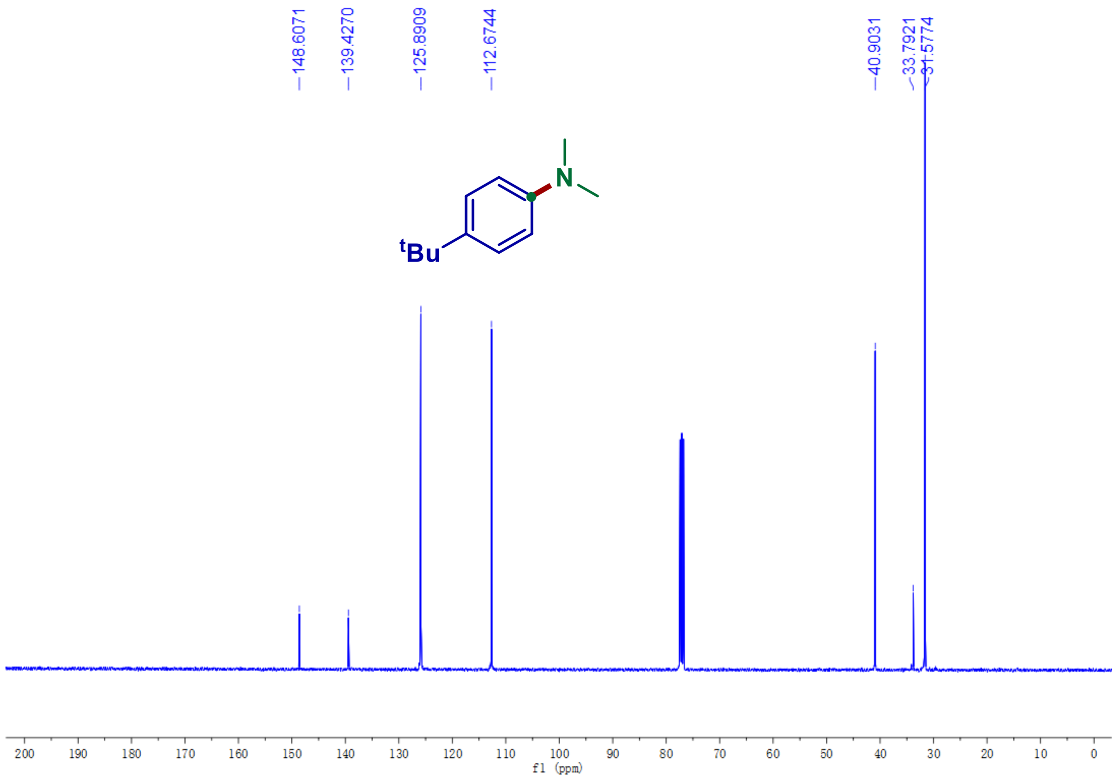

^{13}C NMR (100 MHz, $CDCl_3$) spectrum of compound 98

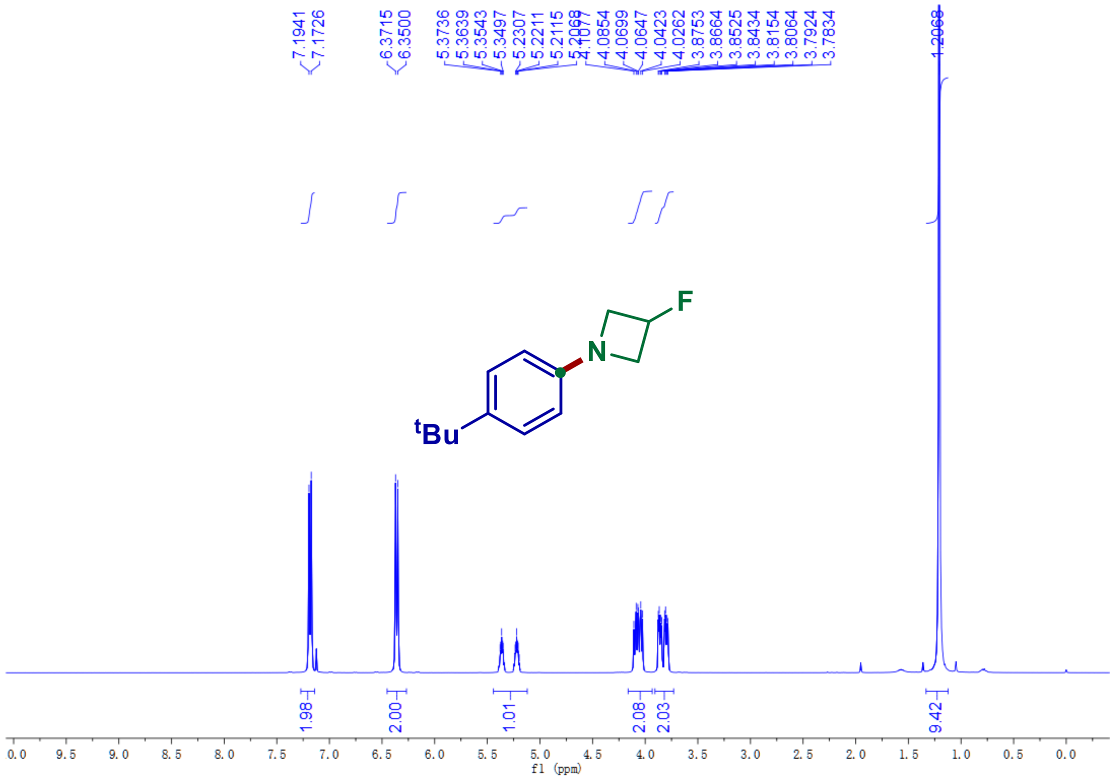

^{1}H NMR (400 MHz, $CDCl_3$) spectrum of compound 99

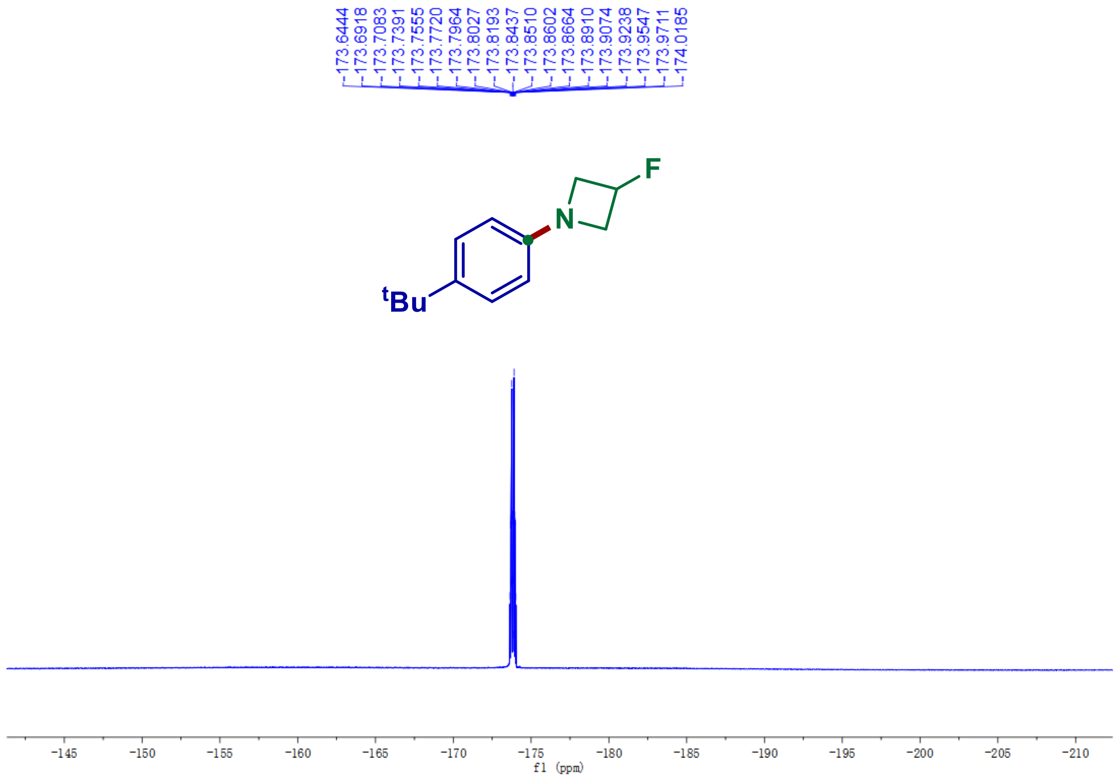

^{19}F NMR (376 MHz, $CDCl_3$) spectrum of compound 99

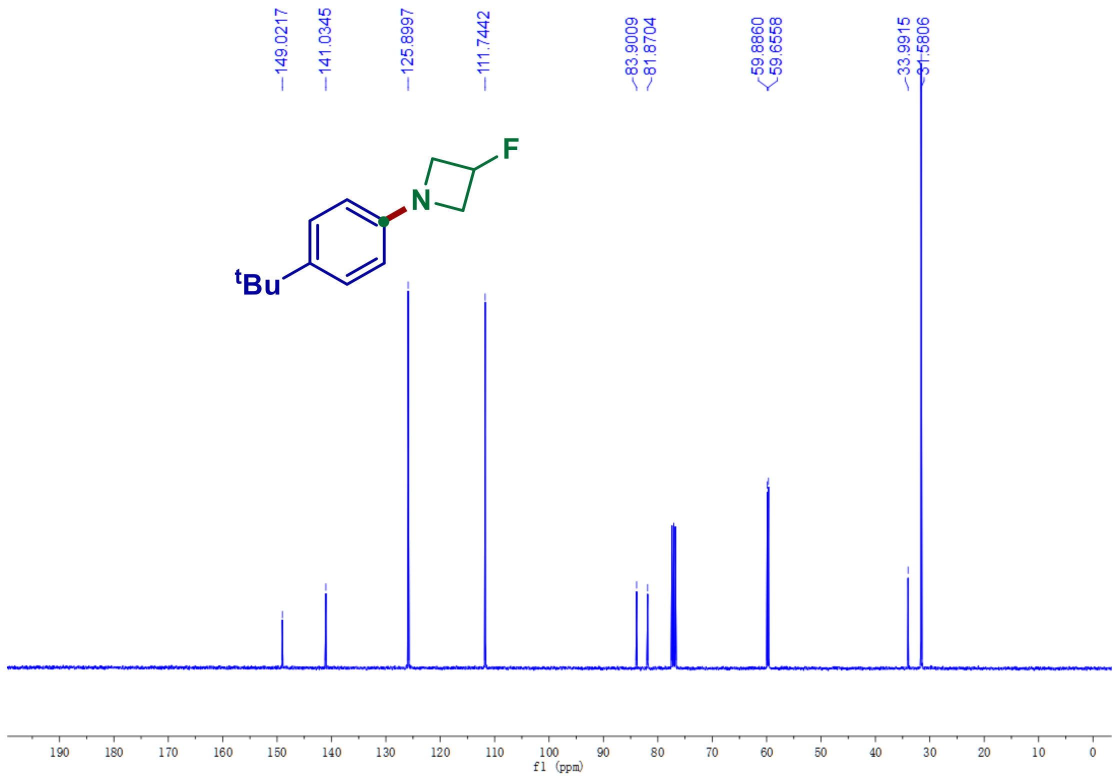

^{13}C NMR (100 MHz, $CDCl_3$) spectrum of compound 99

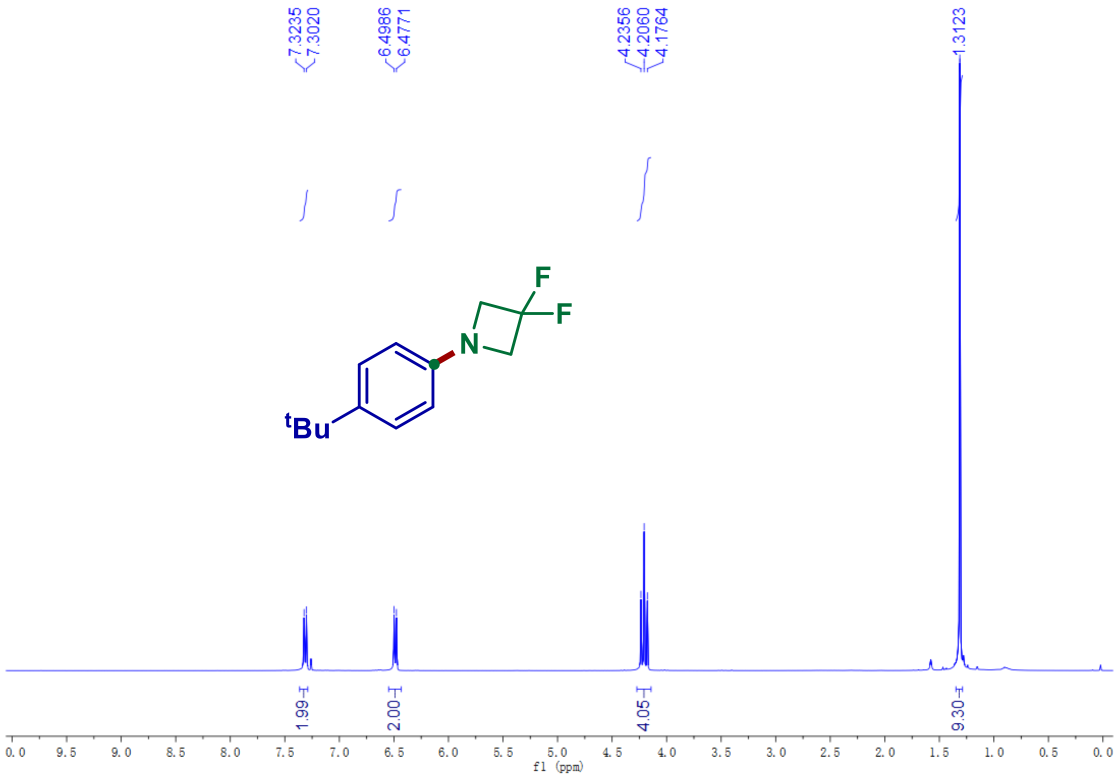

^{1}H NMR (400 MHz, $CDCl_3$) spectrum of compound 100

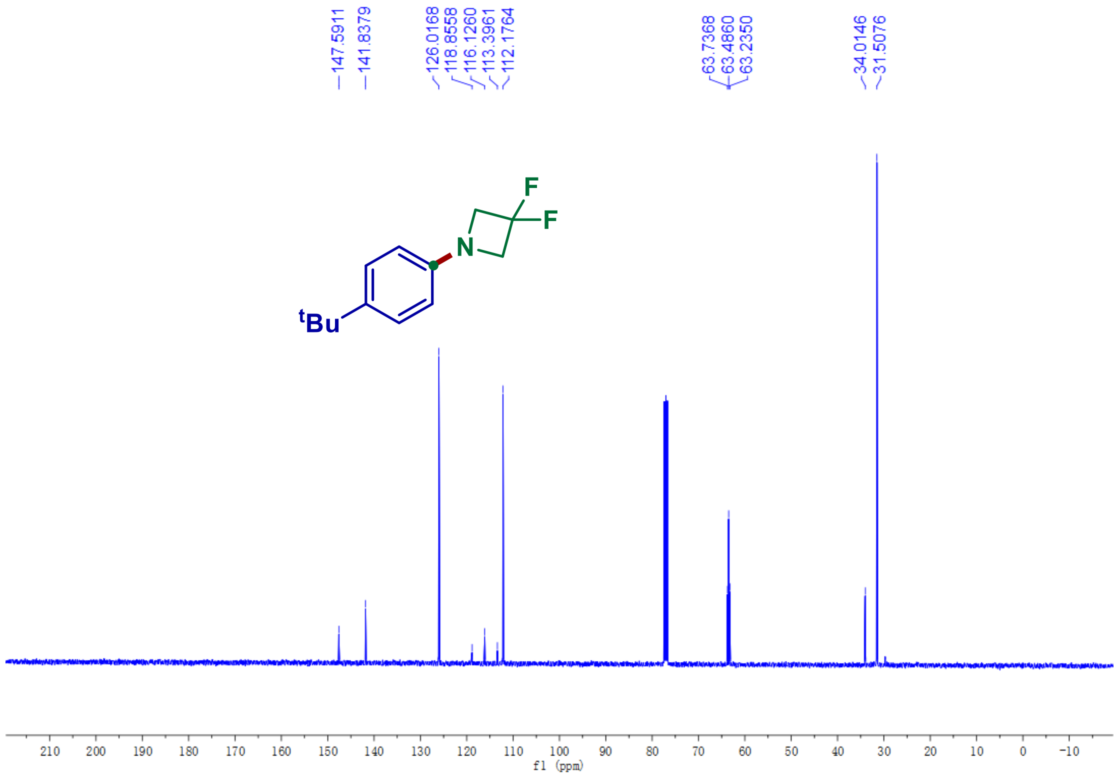

^{13}C NMR (100 MHz, $CDCl_3$) spectrum of compound 100

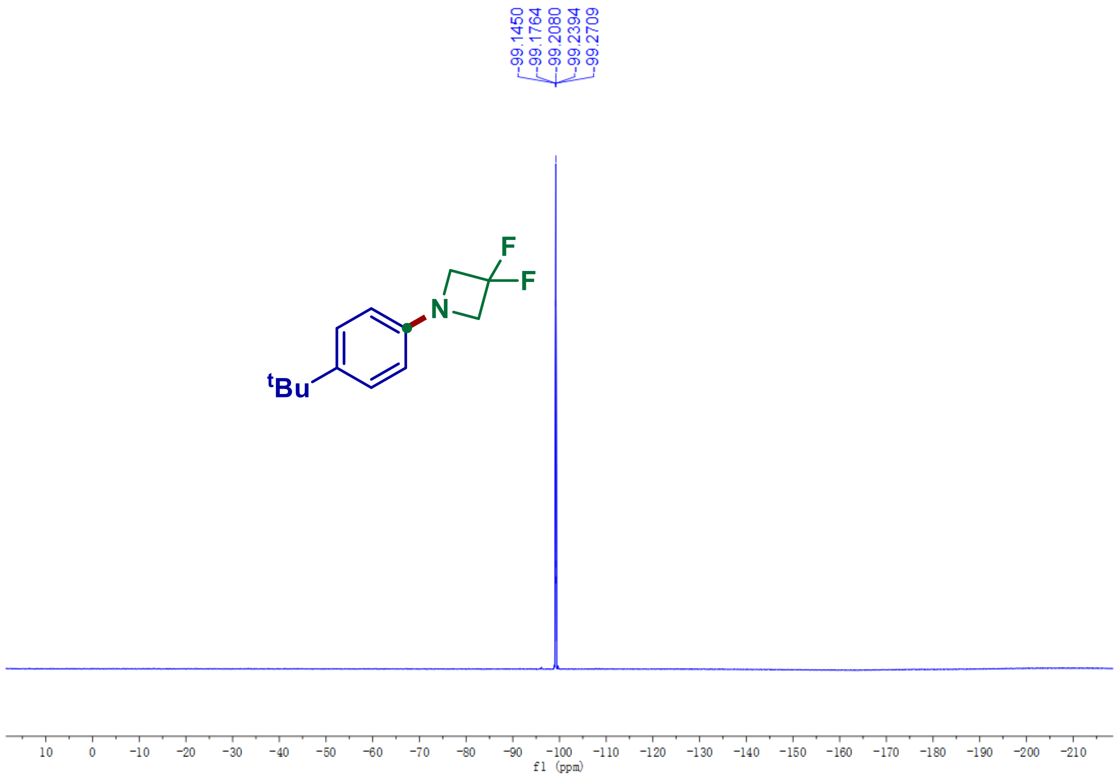

^{19}F NMR (376 MHz, $CDCl_3$) spectrum of compound 100

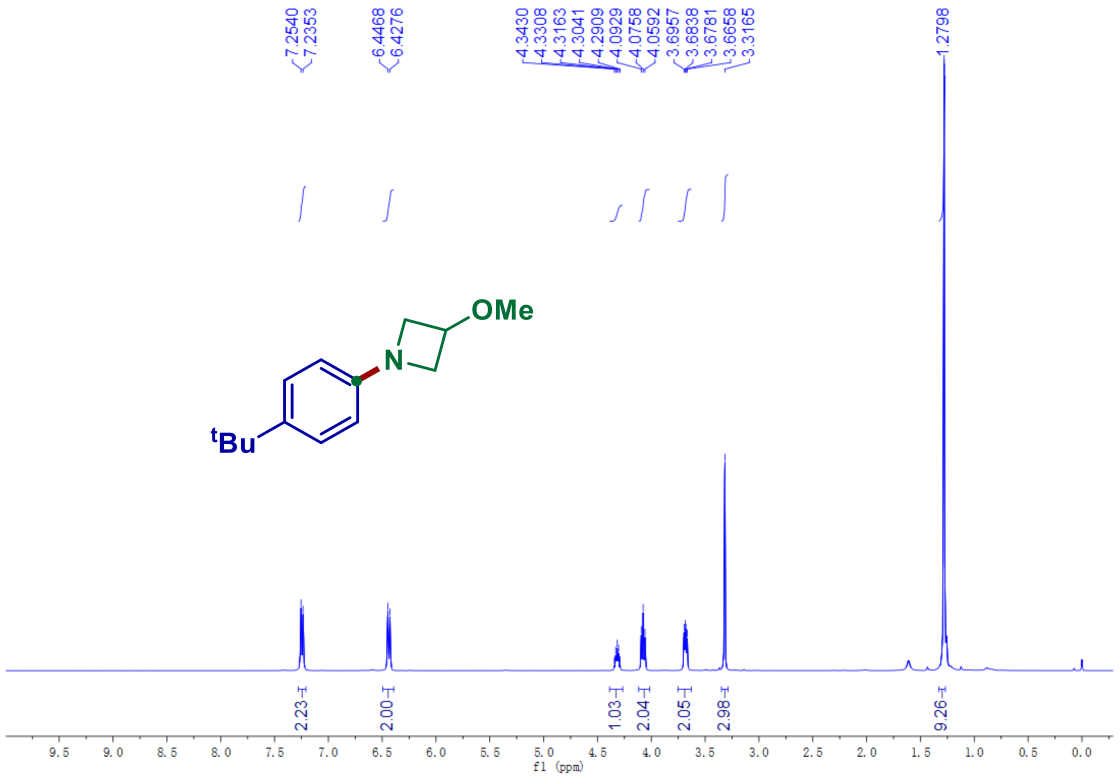

^{1}H NMR (400 MHz, $CDCl_3$) spectrum of compound 101

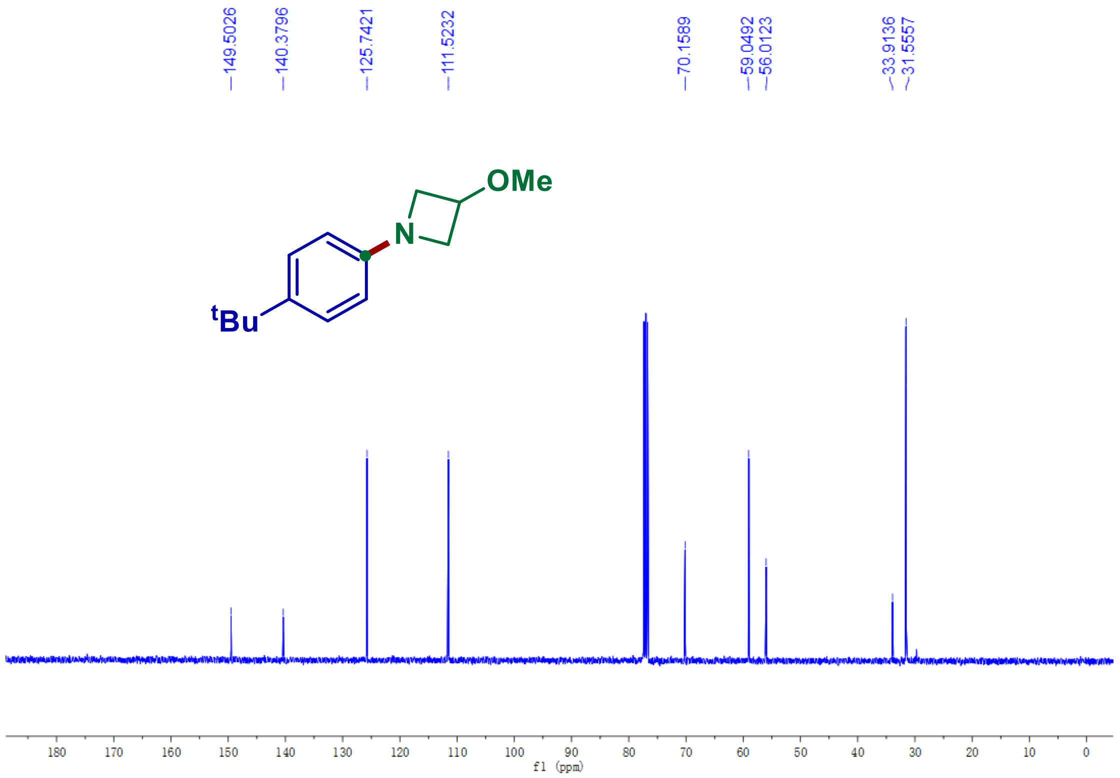

^{13}C NMR (100 MHz, $CDCl_3$) spectrum of compound 101

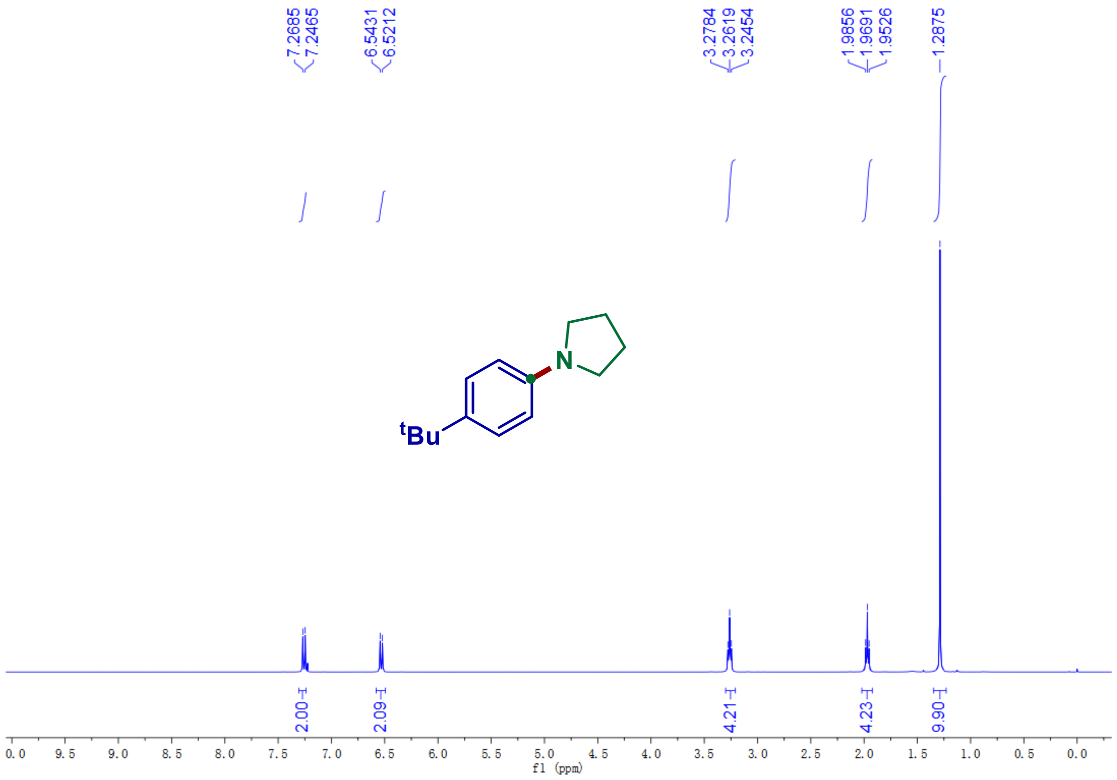

^{1}H NMR (400 MHz, $CDCl_3$) spectrum of compound 102

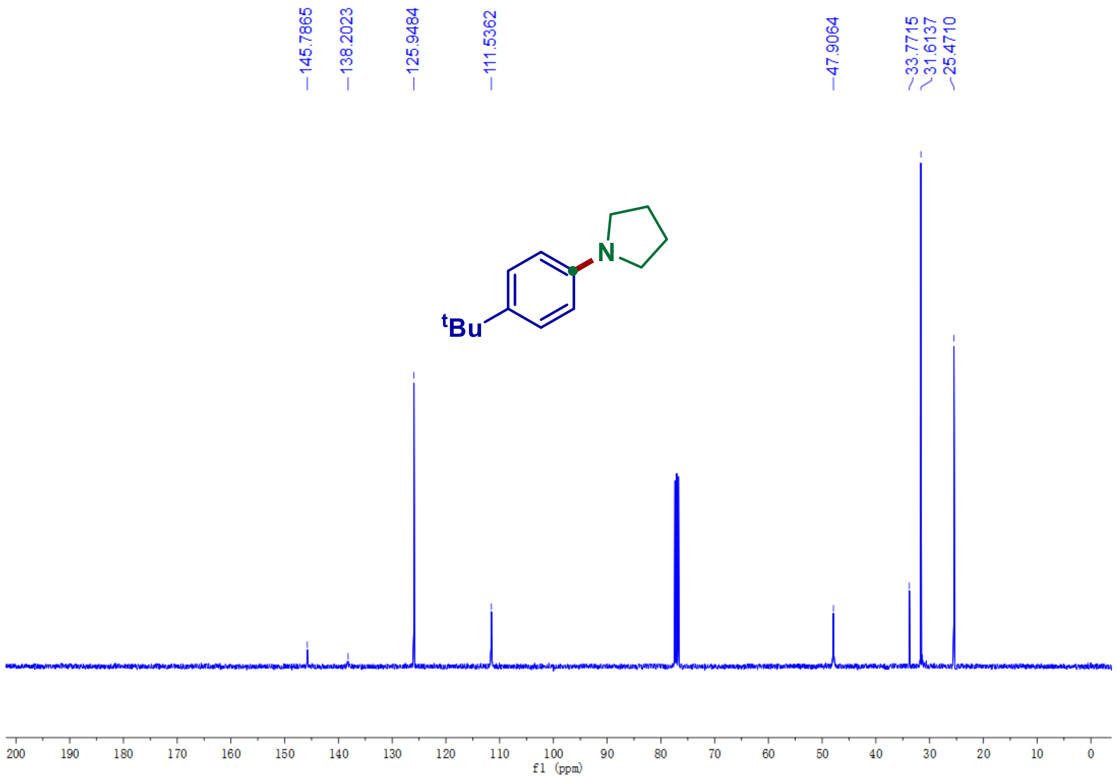

^{13}C NMR (100 MHz, $CDCl_3$) spectrum of compound 102

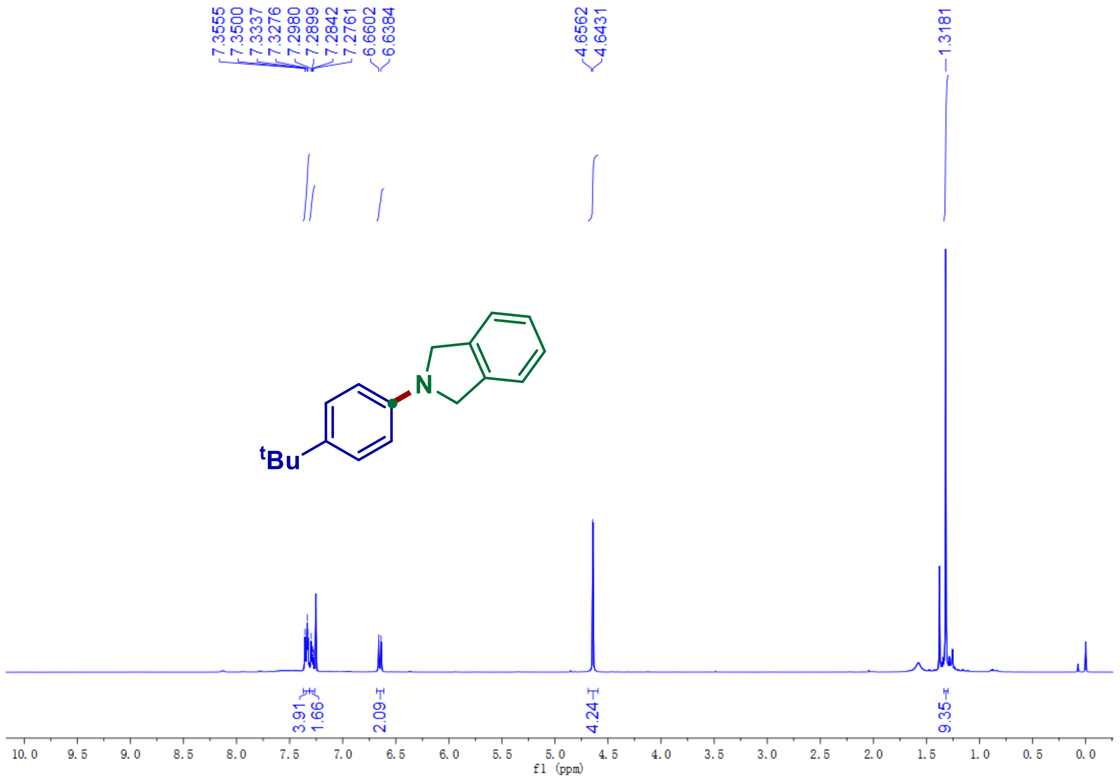

^{1}H NMR (400 MHz, $CDCl_3$) spectrum of compound 103

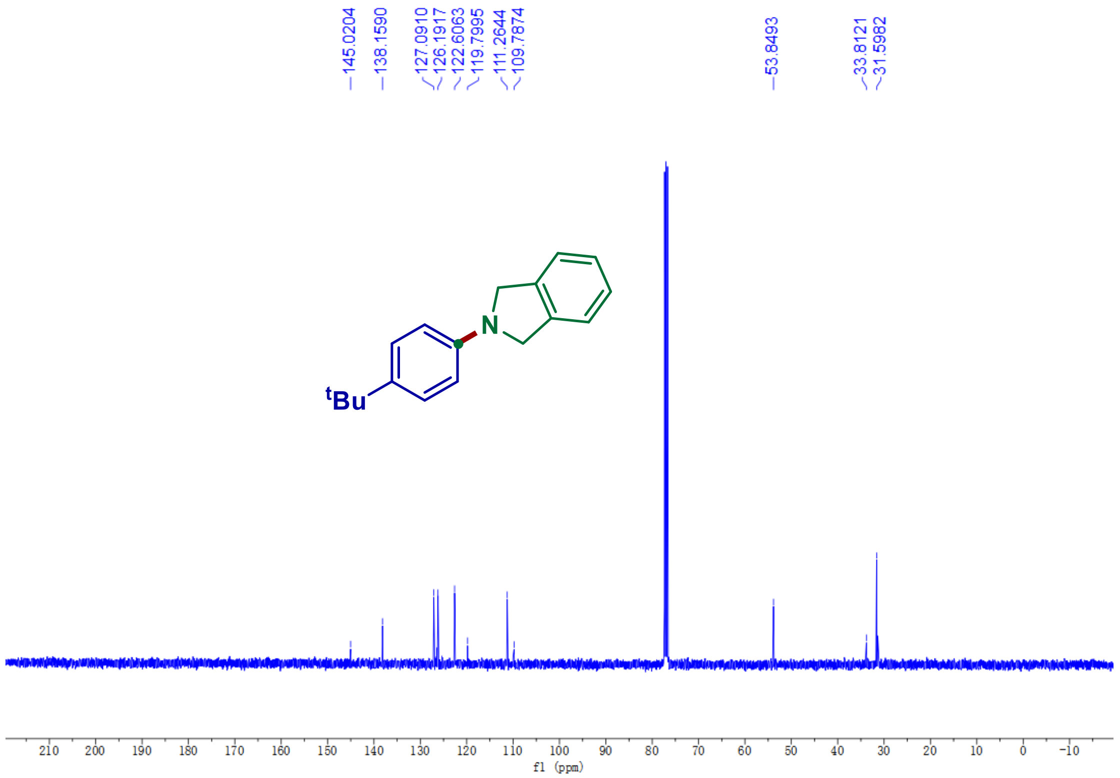

^{13}C NMR (100 MHz, $CDCl_3$) spectrum of compound 103

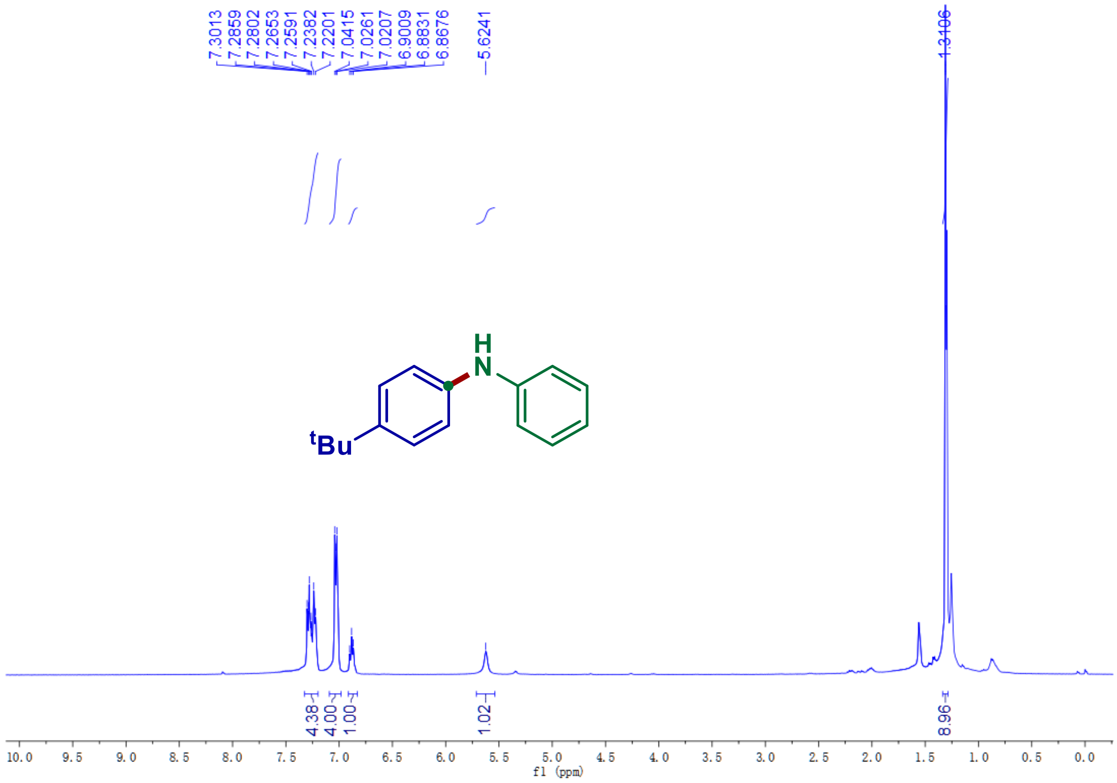

^{1}H NMR (400 MHz, $CDCl_3$) spectrum of compound 104

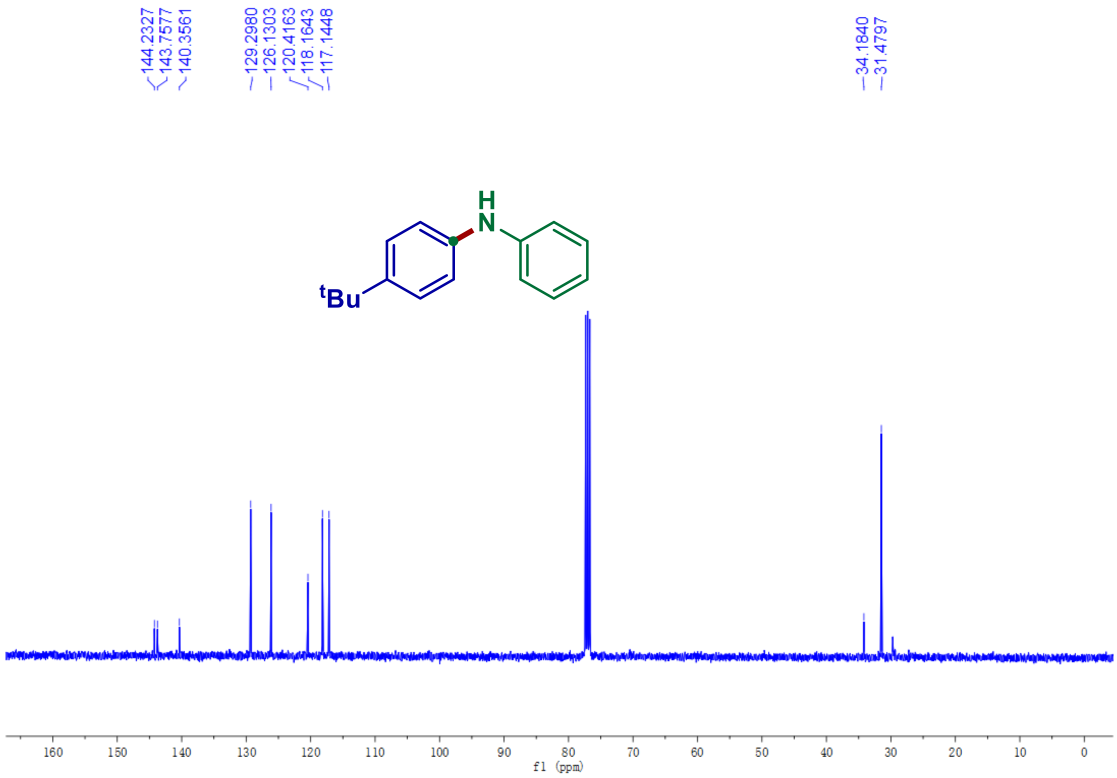

^{13}C NMR (100 MHz, $CDCl_3$) spectrum of compound 104

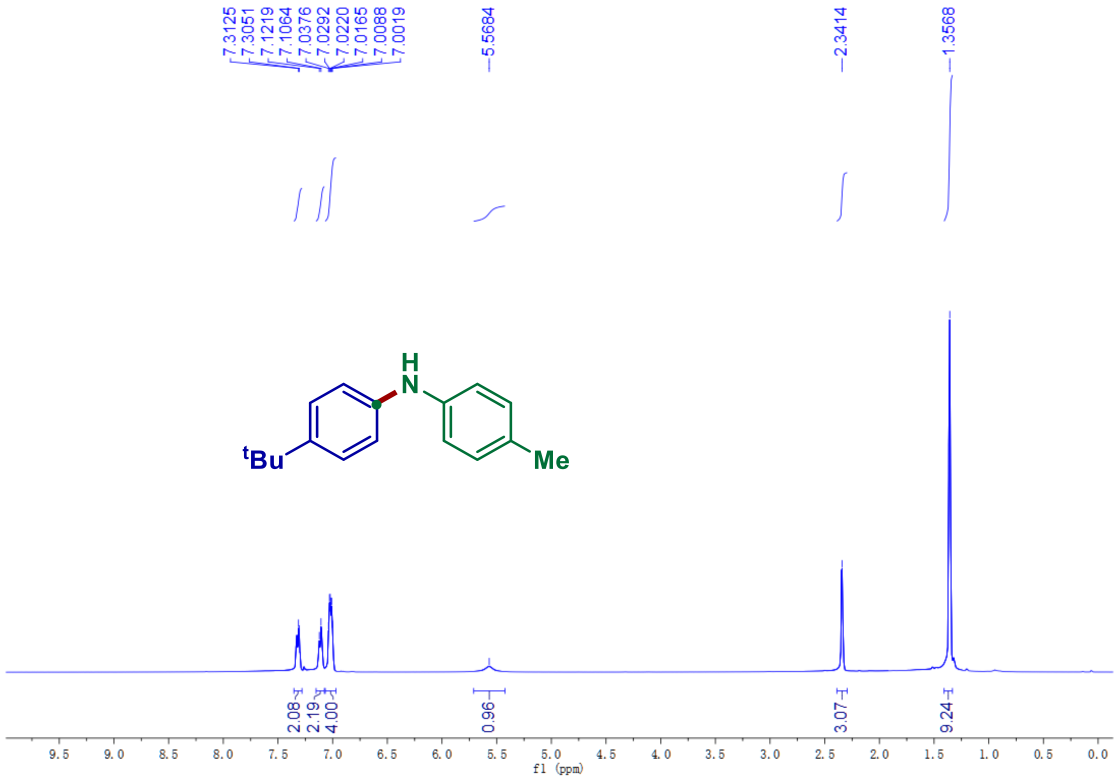

^{1}H NMR (400 MHz, $CDCl_3$) spectrum of compound 105

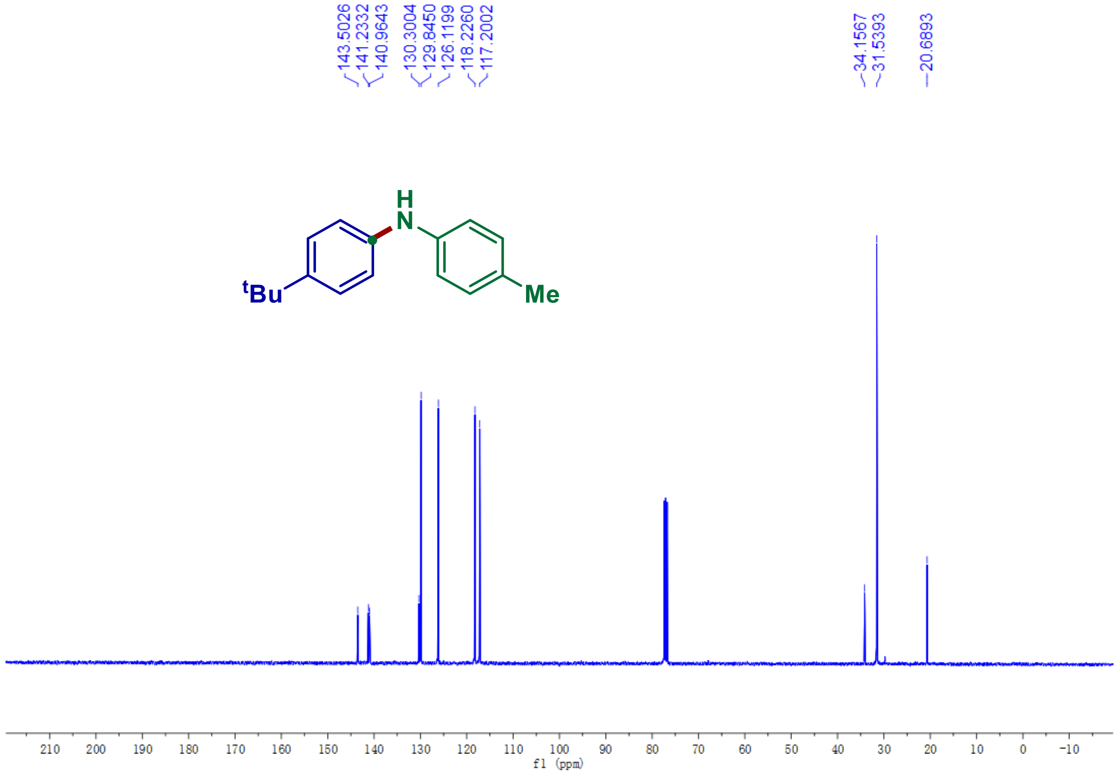

^{13}C NMR (100 MHz, $CDCl_3$) spectrum of compound 105

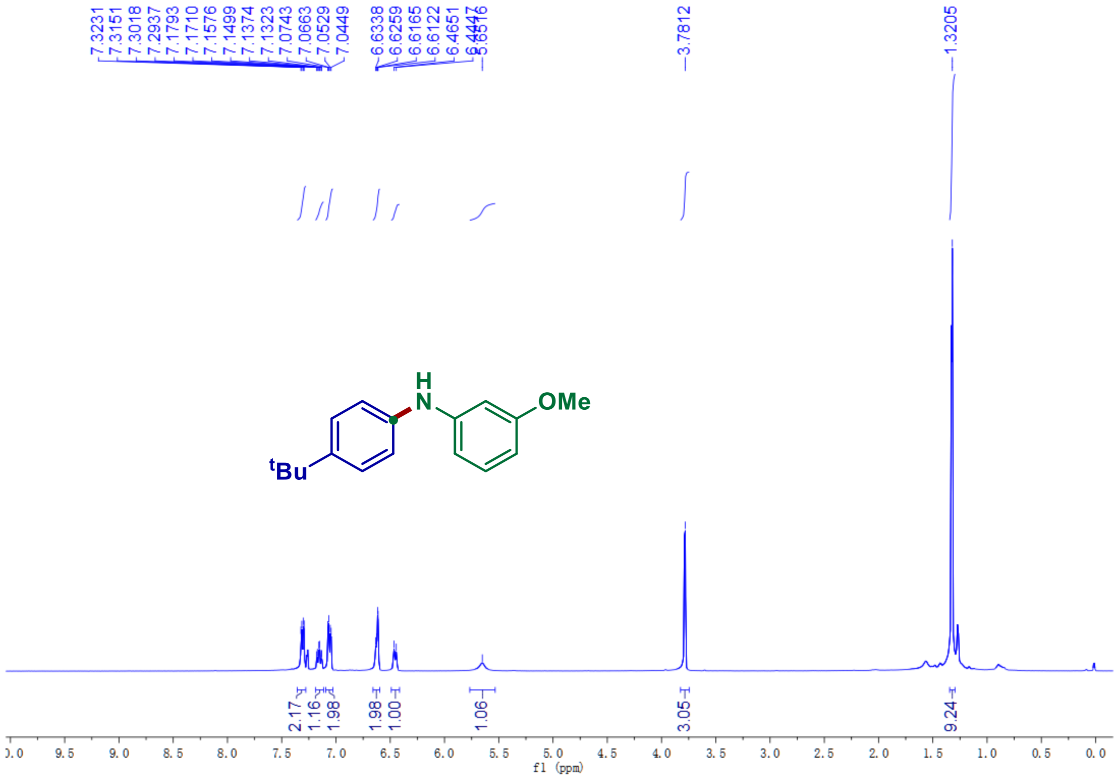

^{1}H NMR (400 MHz, $CDCl_3$) spectrum of compound 106

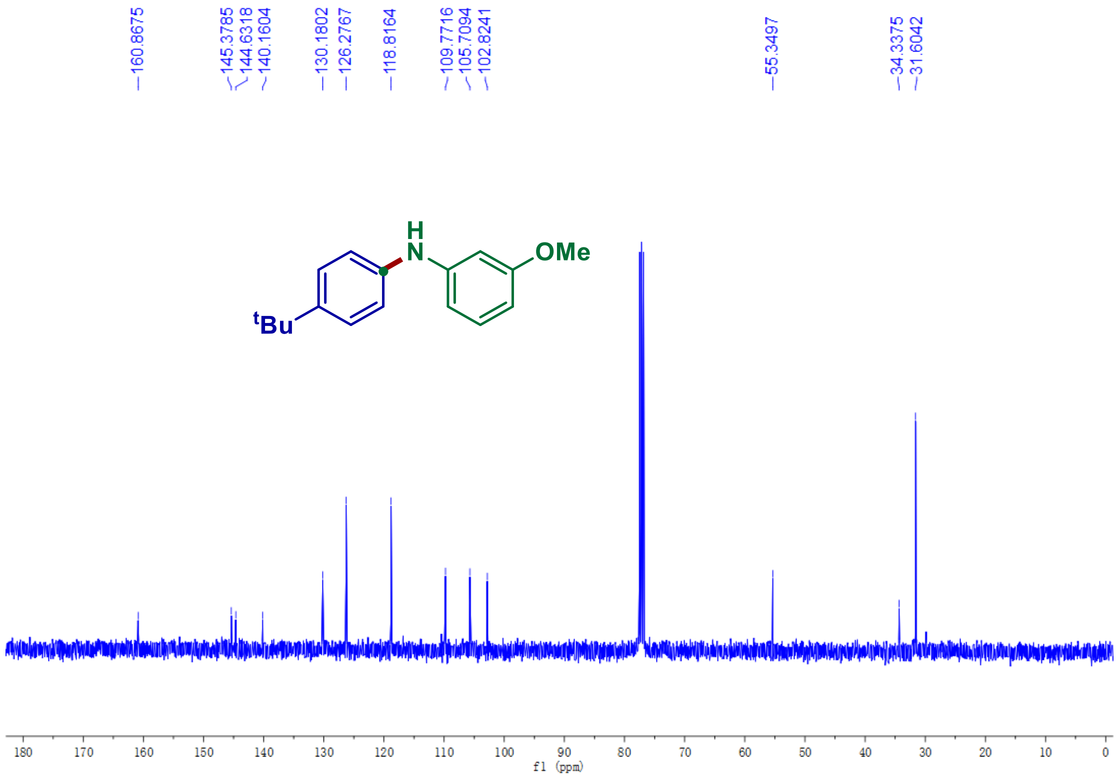

^{13}C NMR (100 MHz, $CDCl_3$) spectrum of compound 106

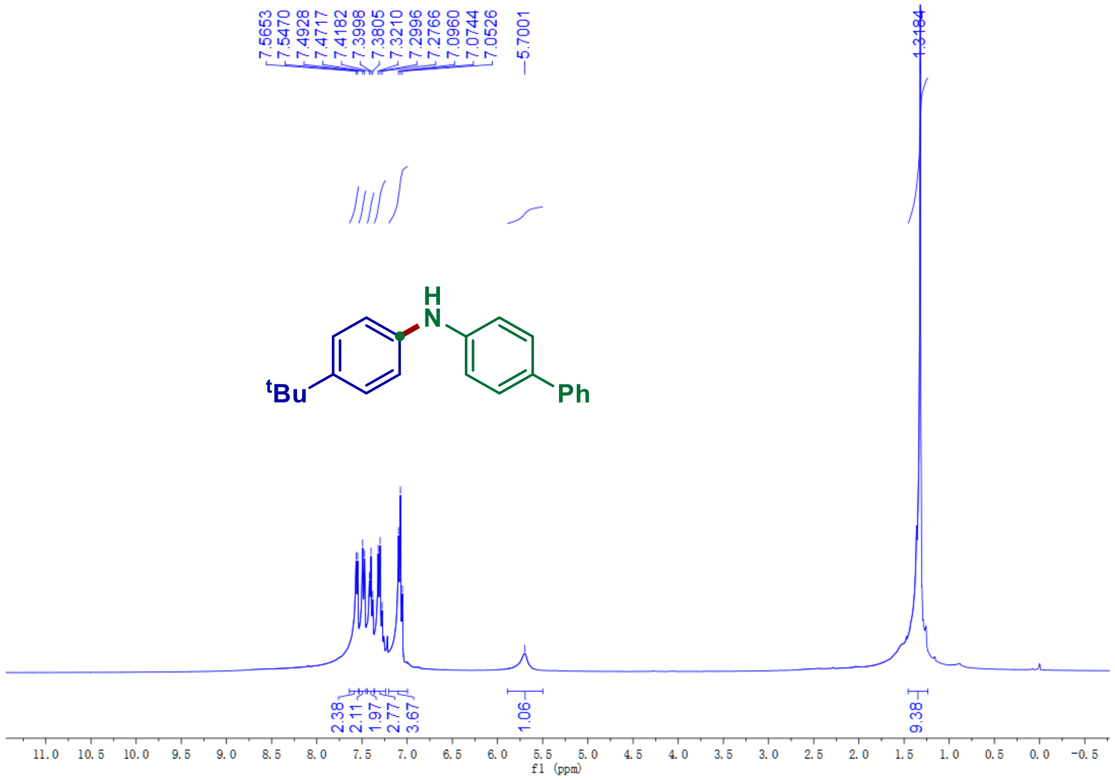

^{1}H NMR (400 MHz, $CDCl_3$) spectrum of compound 107

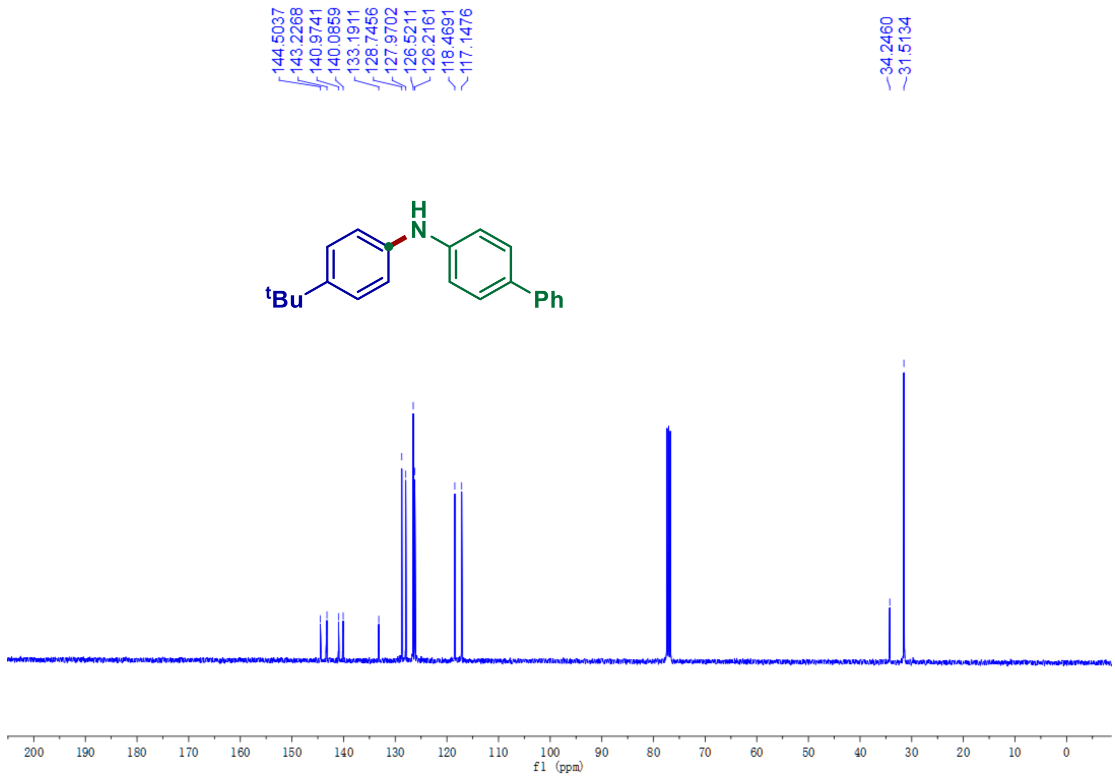

^{13}C NMR (100 MHz, $CDCl_3$) spectrum of compound 107

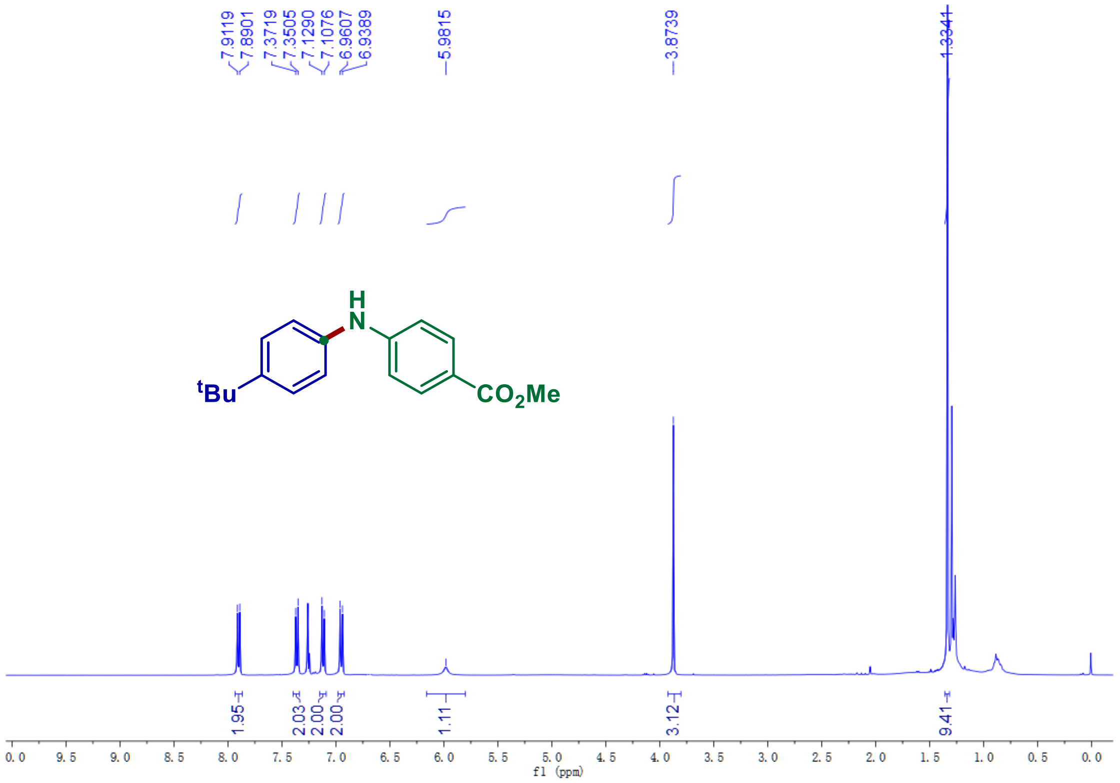

^{1}H NMR (400 MHz, $CDCl_3$) spectrum of compound 108

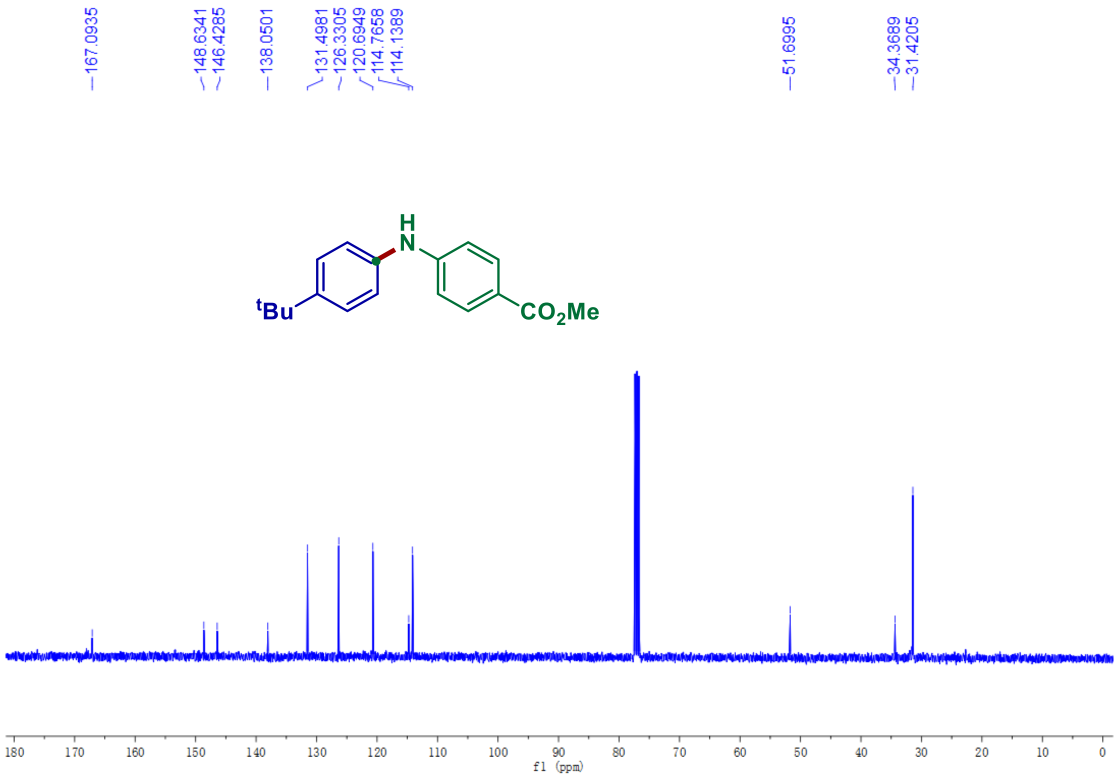

^{13}C NMR (100 MHz, $CDCl_3$) spectrum of compound 108

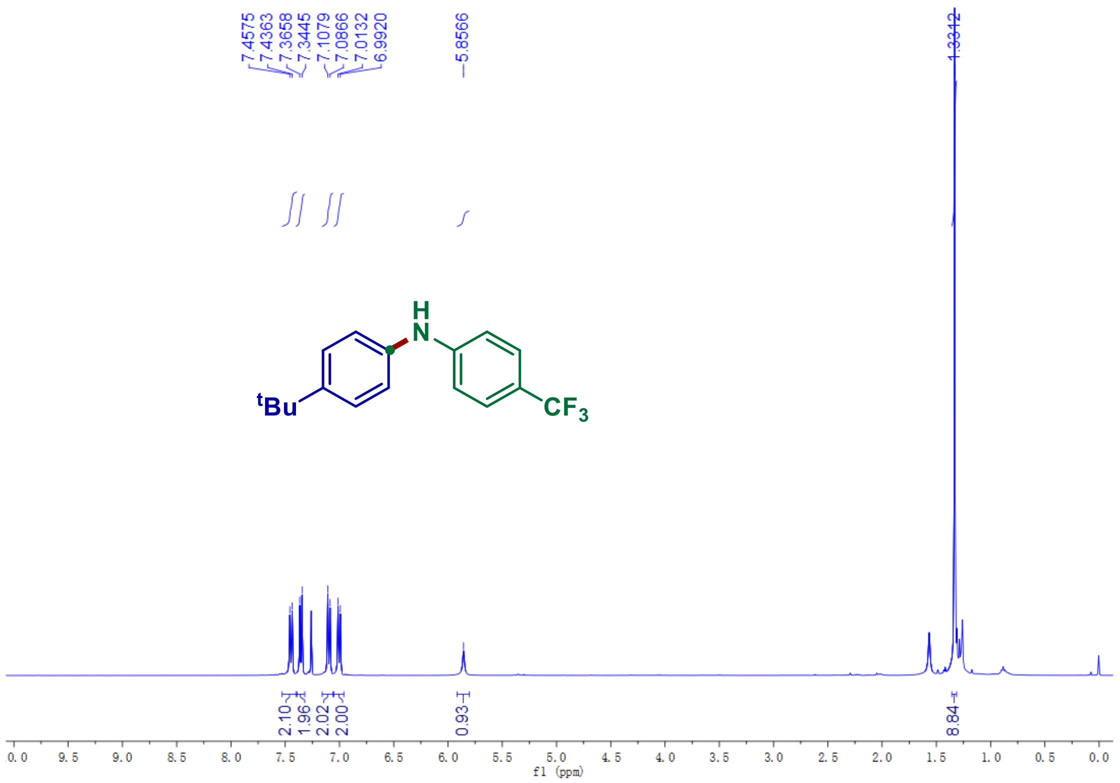

^{1}H NMR (400 MHz, $CDCl_3$) spectrum of compound 109

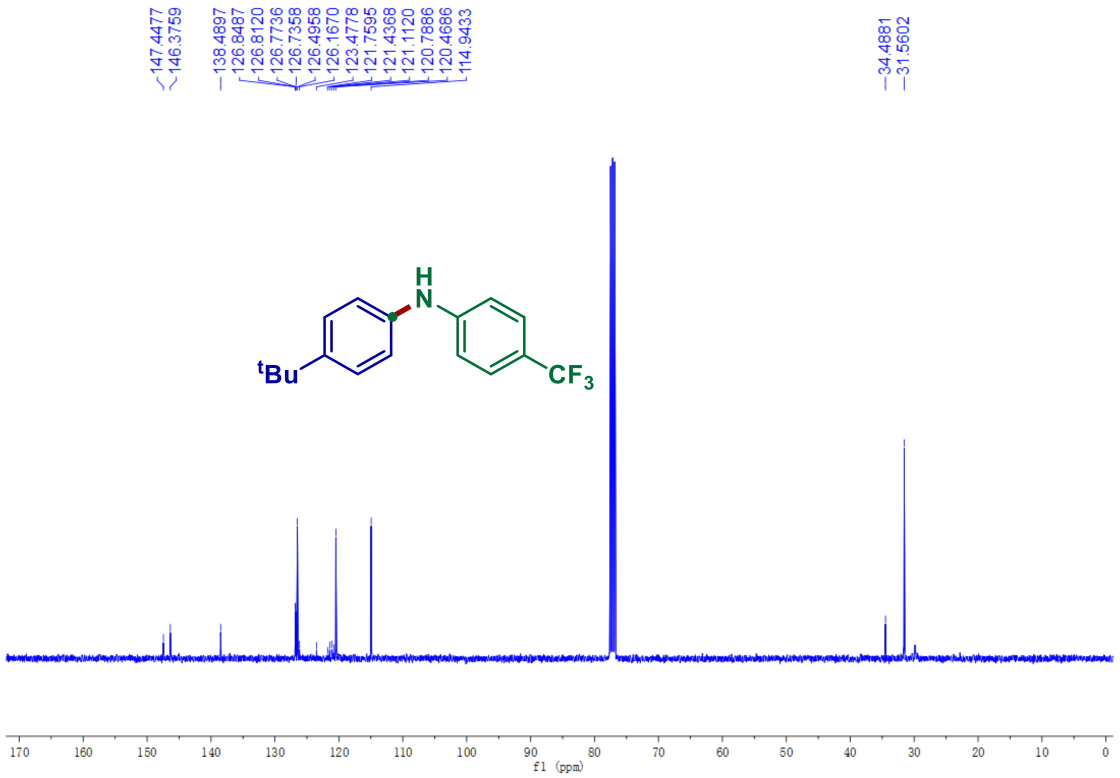

^{13}C NMR (100 MHz, $CDCl_3$) spectrum of compound 109

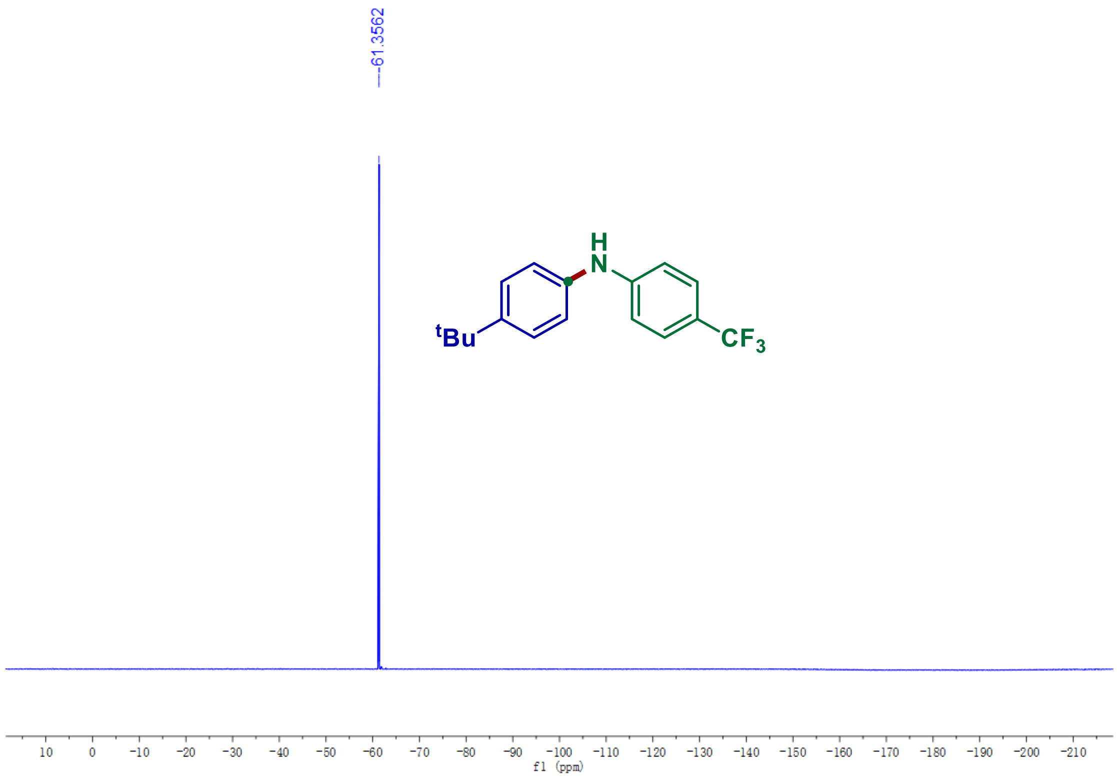

^{19}F NMR (376 MHz, $CDCl_3$) spectrum of compound 109

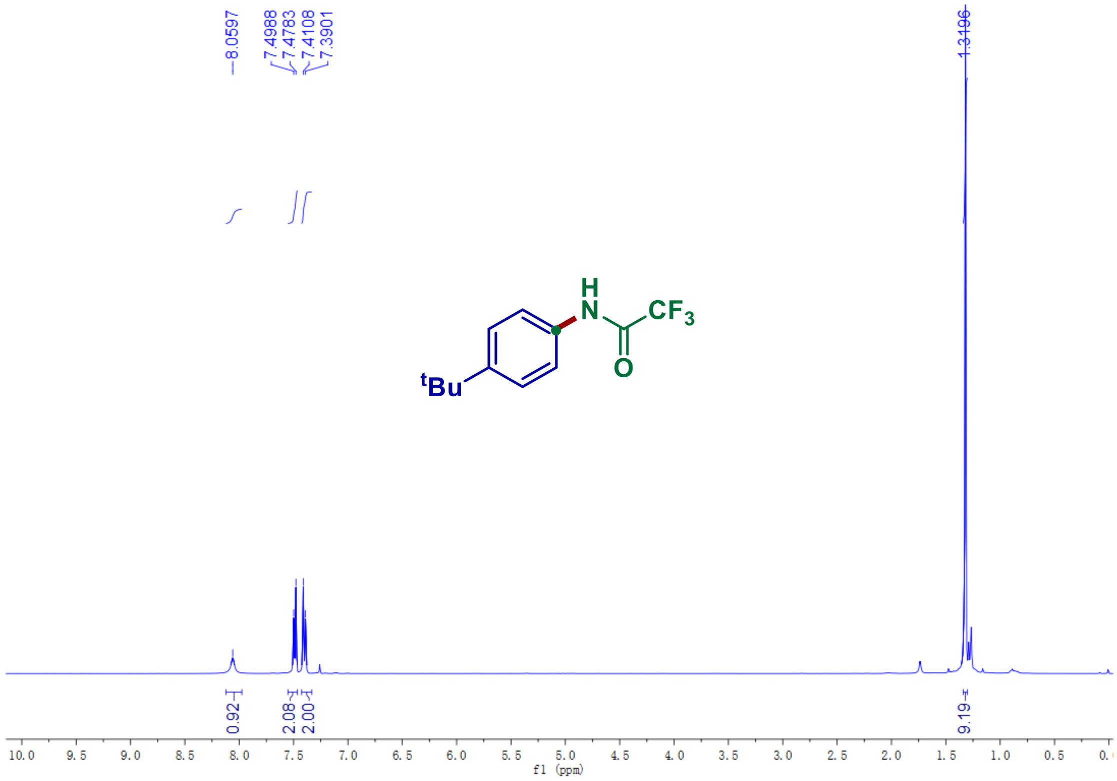

^{1}H NMR (400 MHz, $CDCl_3$) spectrum of compound 110

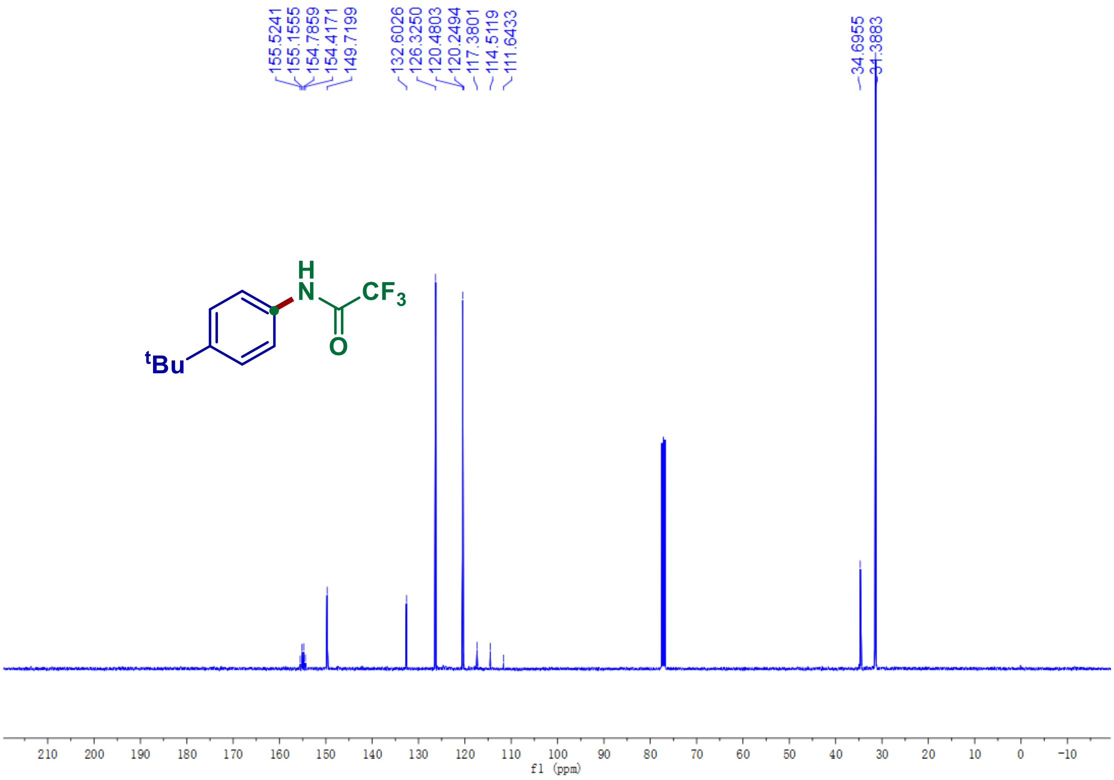

^{13}C NMR (100 MHz, $CDCl_3$) spectrum of compound 110

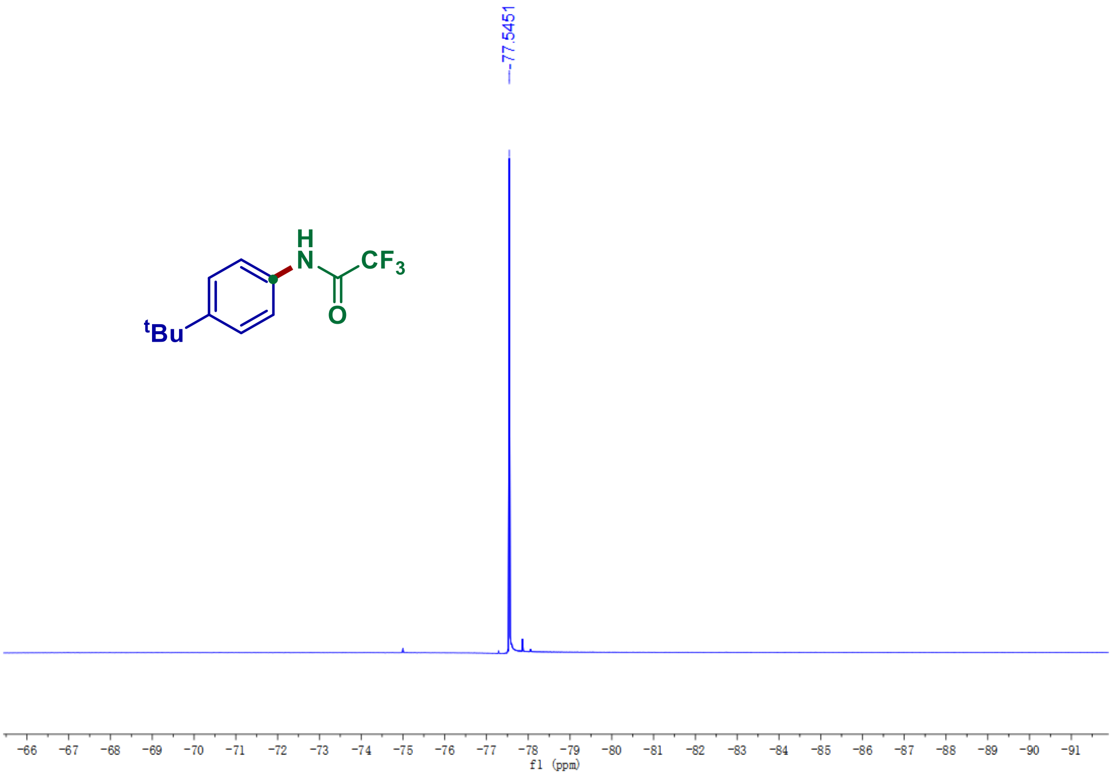

^{19}F NMR (376 MHz, $CDCl_3$) spectrum of compound 110

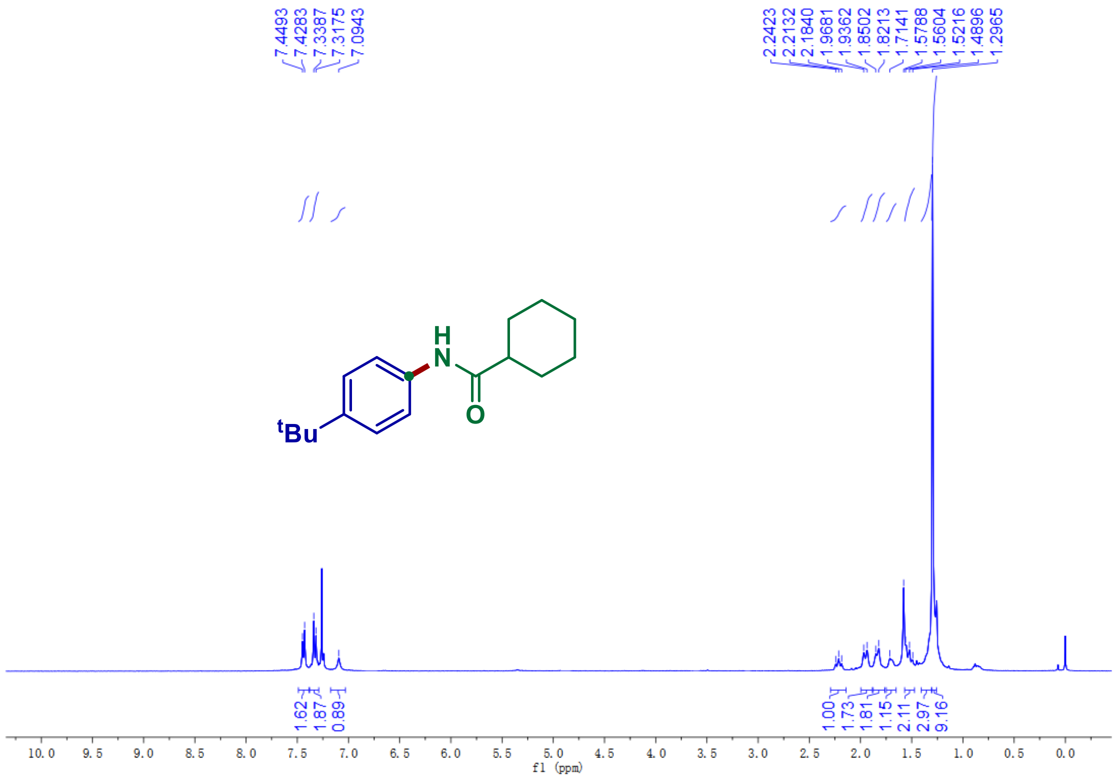

^{1}H NMR (400 MHz, $CDCl_3$) spectrum of compound 111

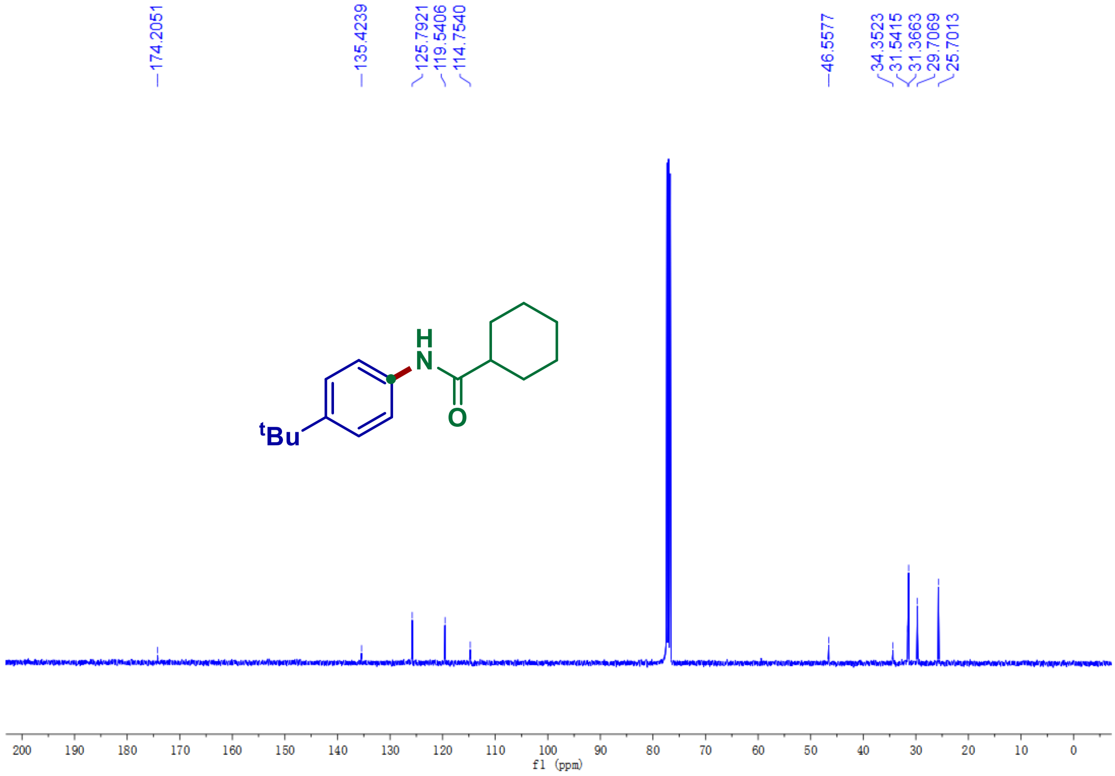

^{13}C NMR (100 MHz, $CDCl_3$) spectrum of compound 111

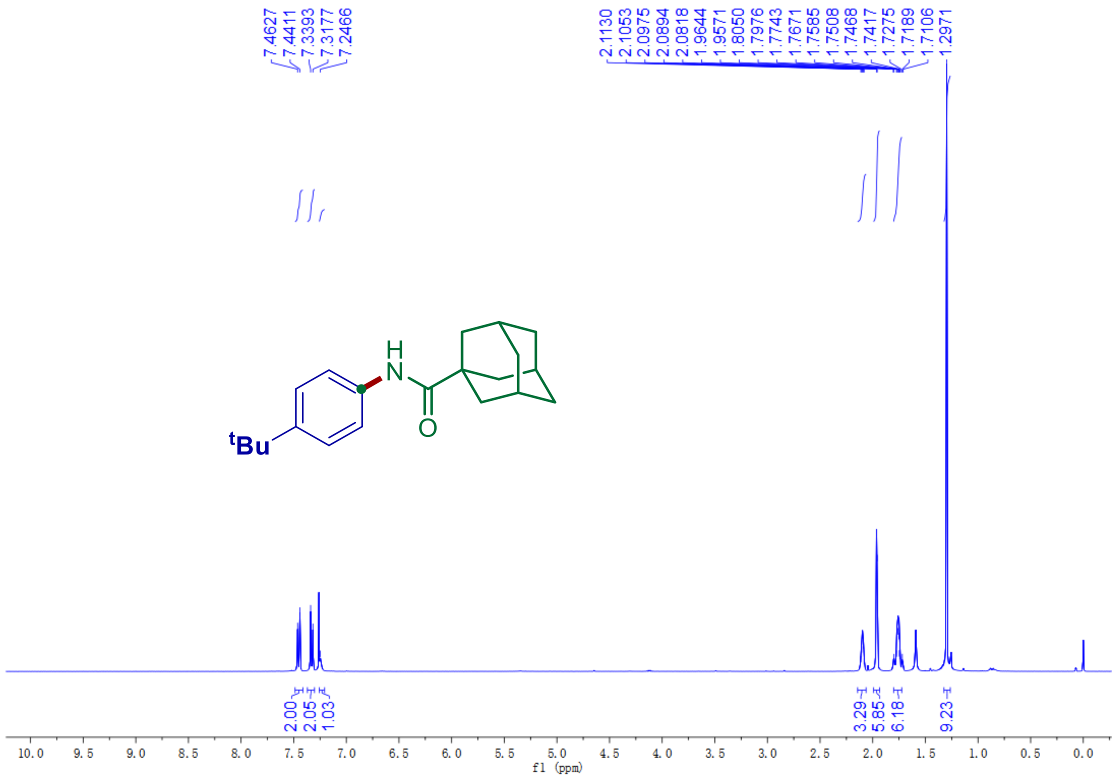

^{1}H NMR (400 MHz, $CDCl_3$) spectrum of compound 112

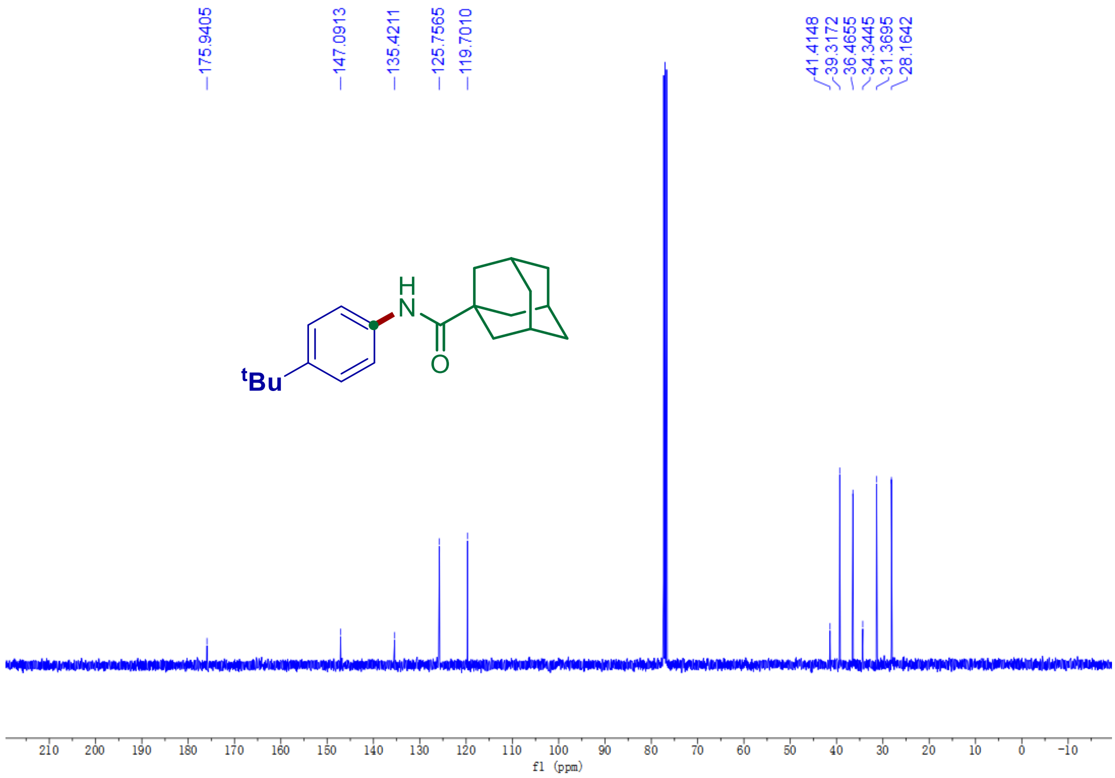

^{13}C NMR (100 MHz, $CDCl_3$) spectrum of compound 112

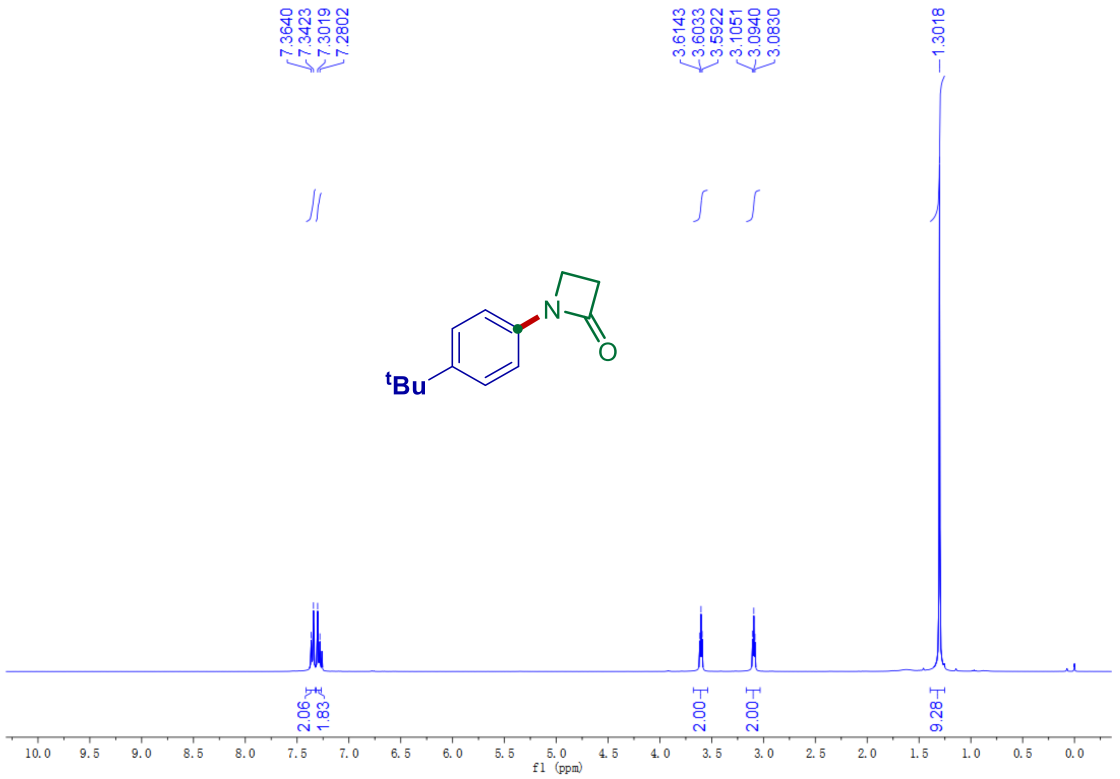

^{1}H NMR (400 MHz, $CDCl_3$) spectrum of compound 113

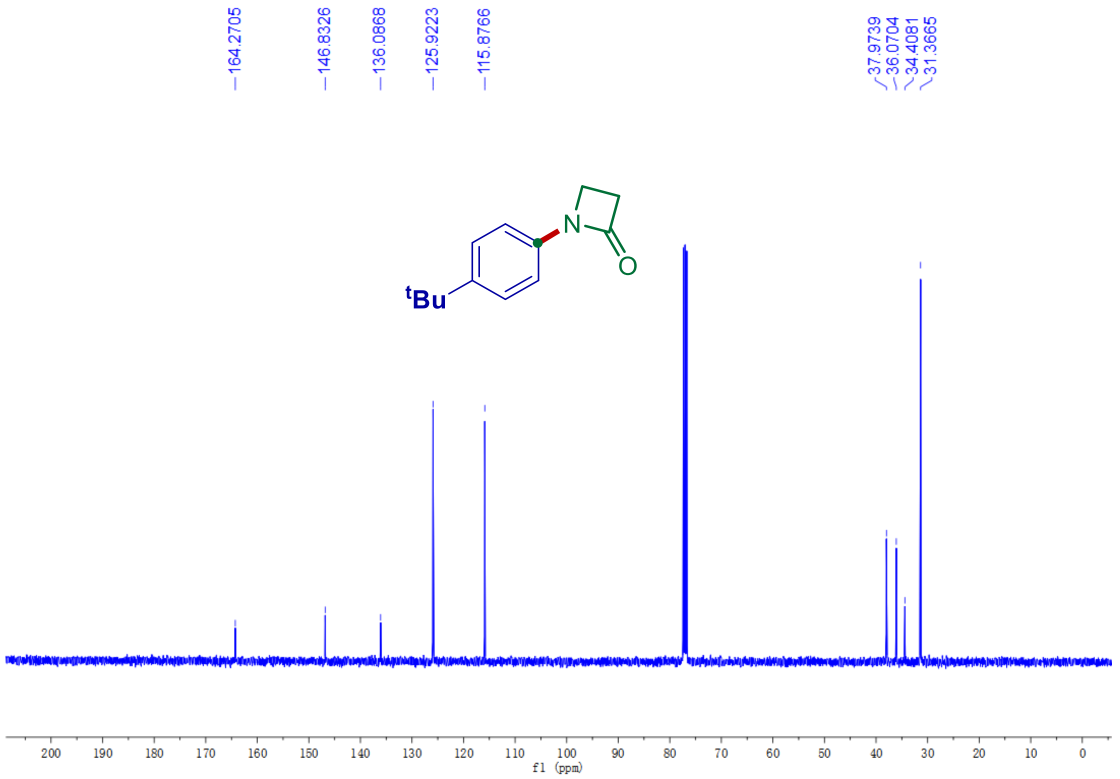

^{13}C NMR (100 MHz, $CDCl_3$) spectrum of compound 113

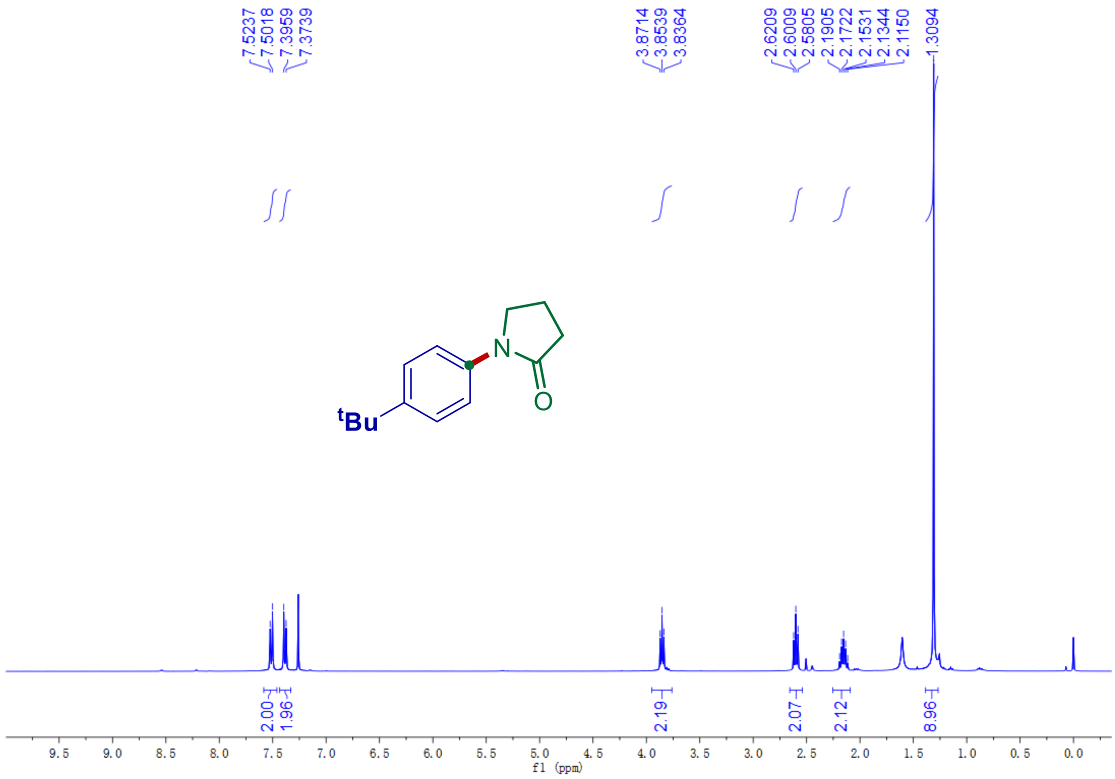

^{1}H NMR (400 MHz, $CDCl_3$) spectrum of compound 114

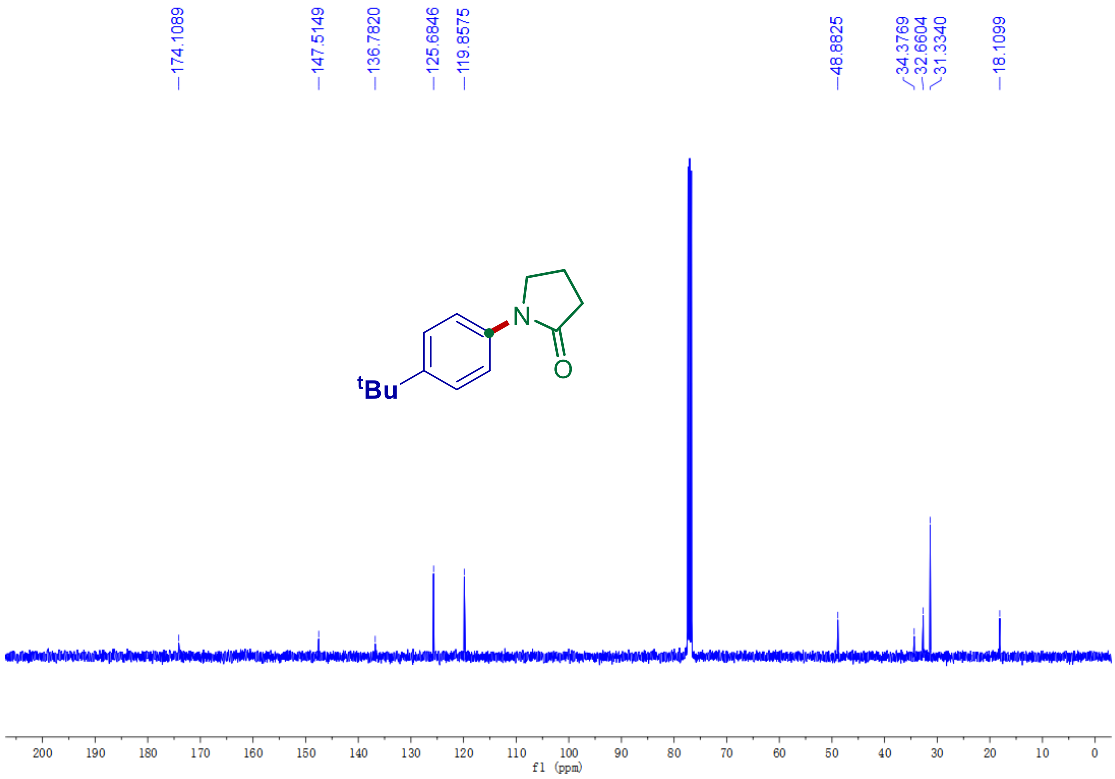

^{13}C NMR (100 MHz, $CDCl_3$) spectrum of compound 114

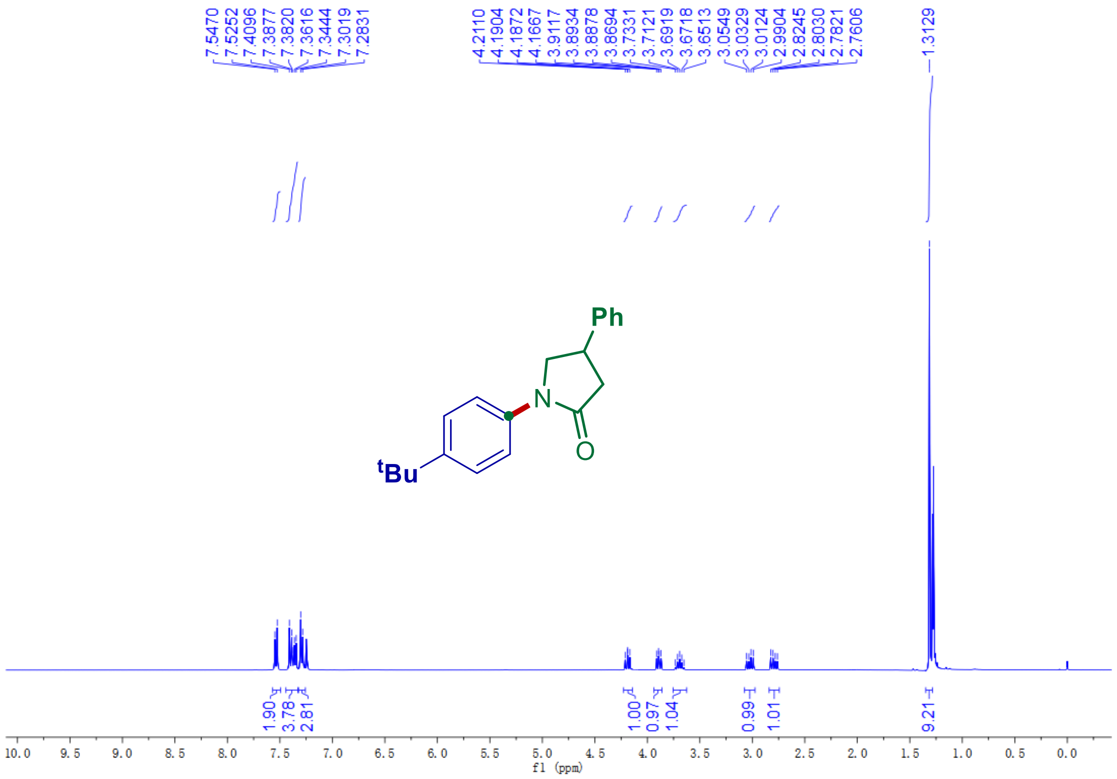

^{1}H NMR (400 MHz, $CDCl_3$) spectrum of compound 115

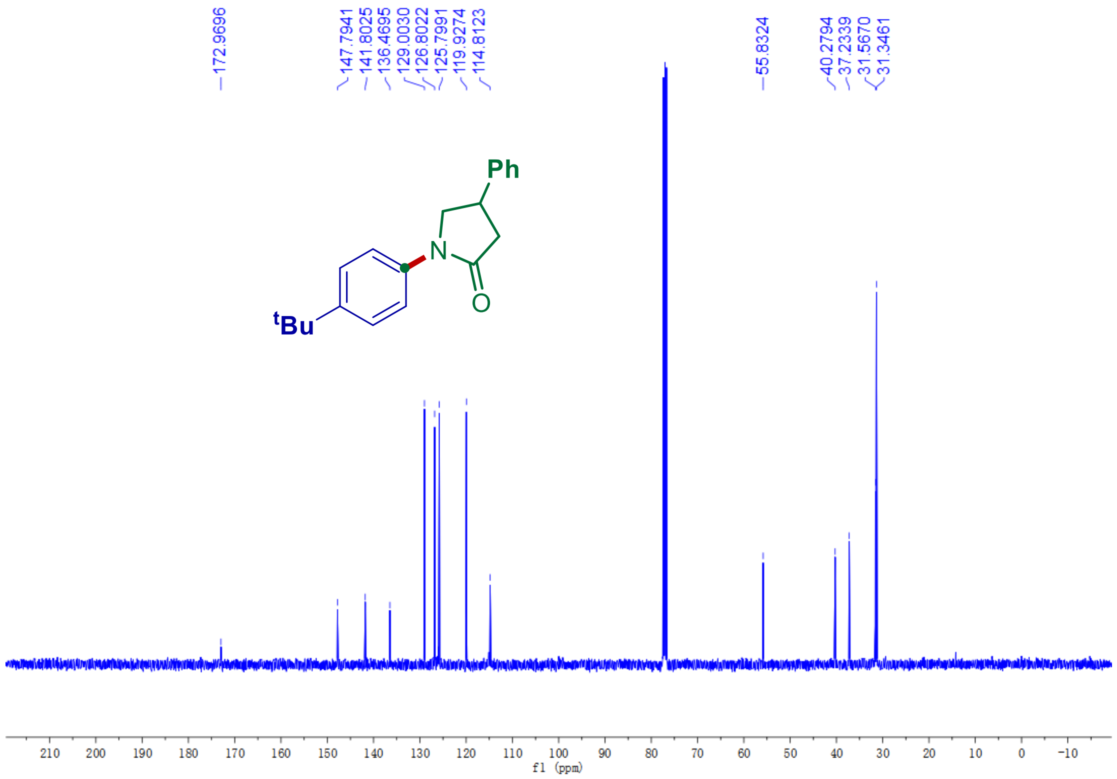

^{13}C NMR (100 MHz, $CDCl_3$) spectrum of compound 115

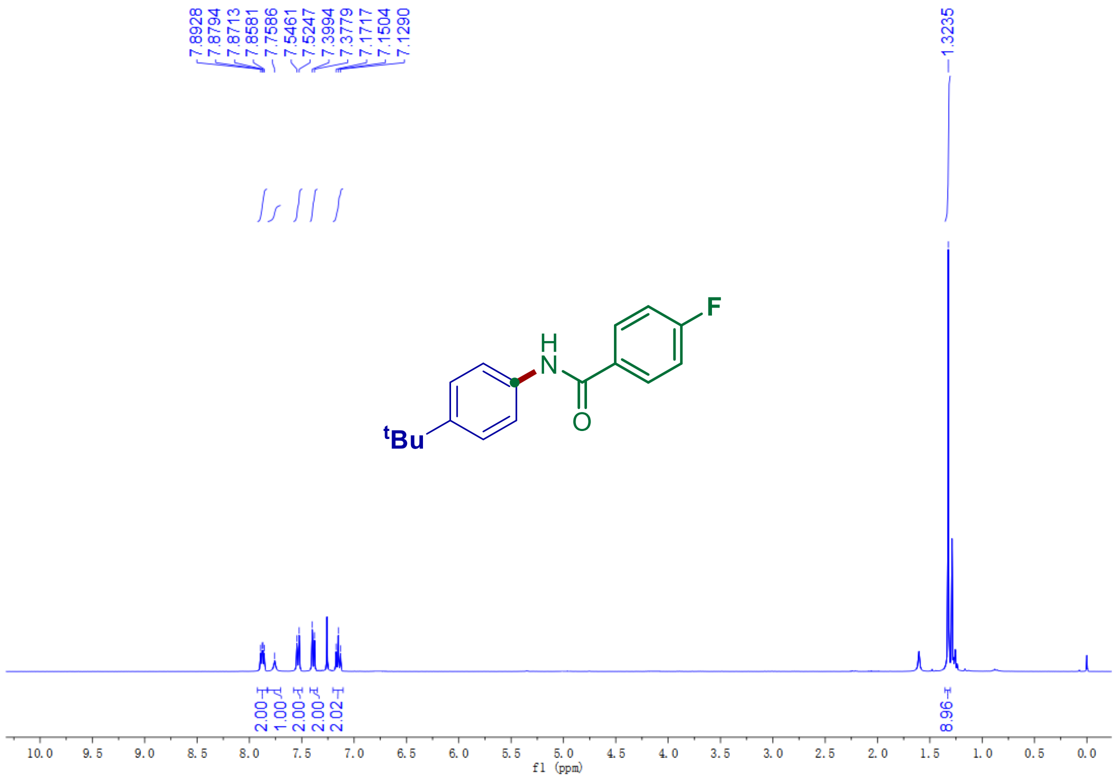

^{1}H NMR (400 MHz, $CDCl_3$) spectrum of compound 116

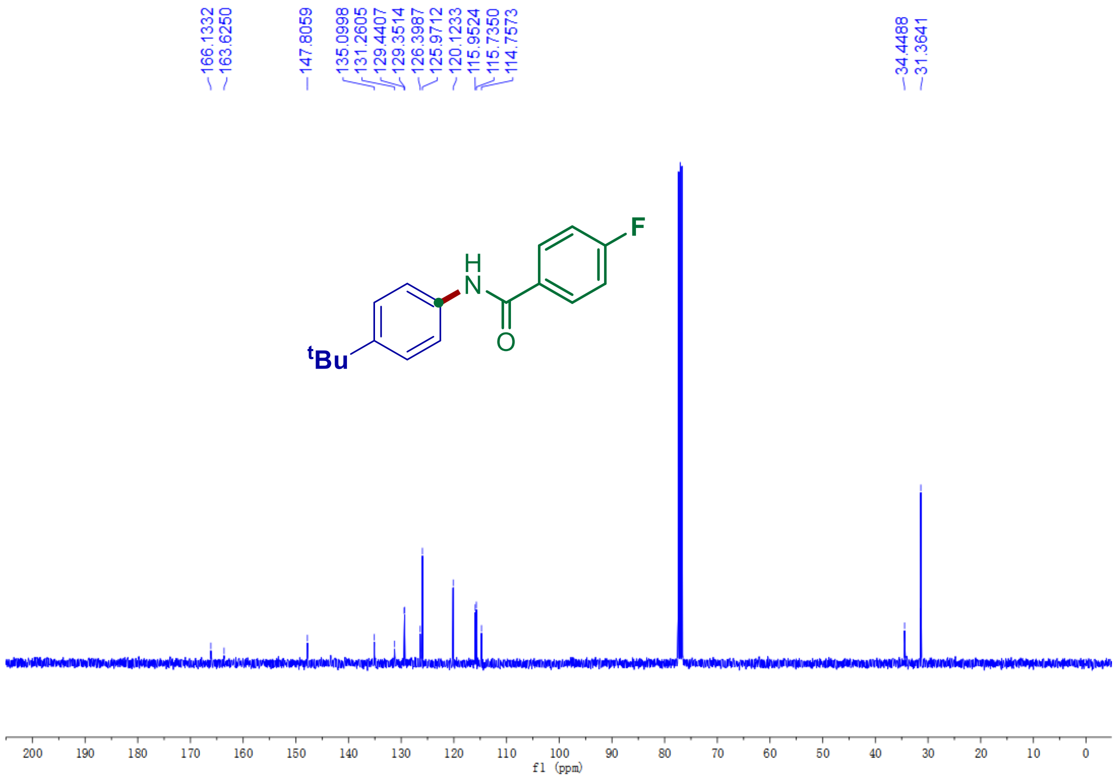

^{13}C NMR (100 MHz, $CDCl_3$) spectrum of compound 116

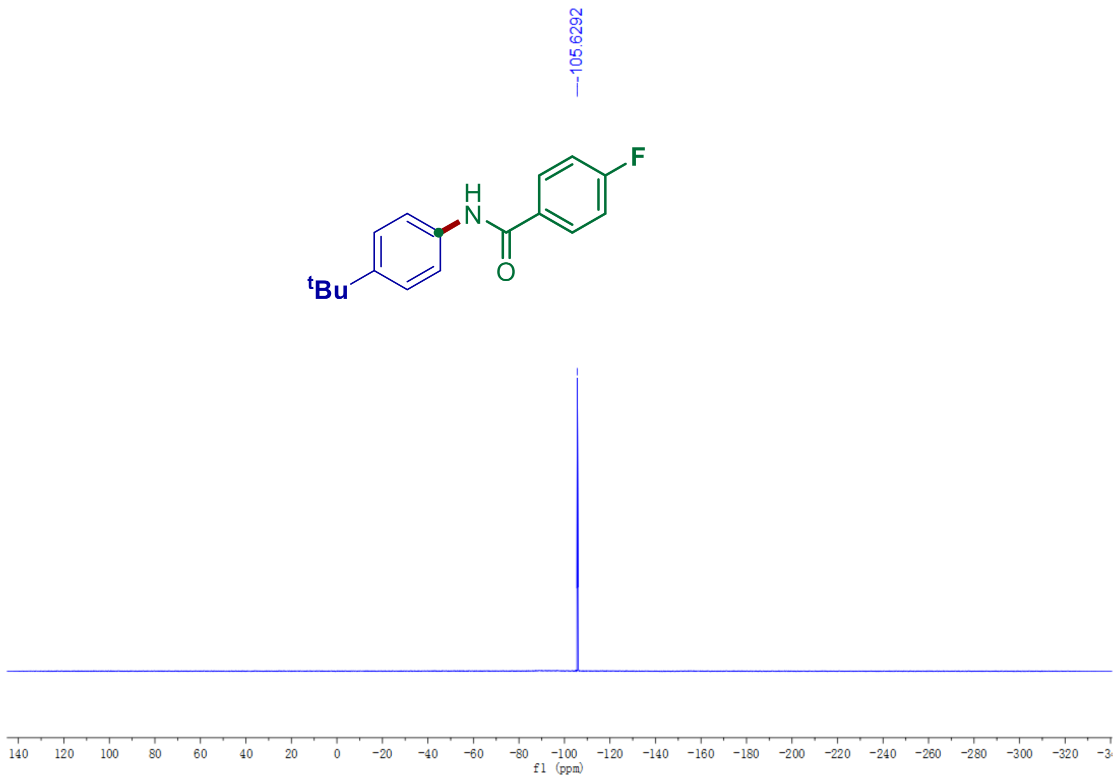

^{19}F NMR (376 MHz, CDCl$_3$) spectrum of compound 116

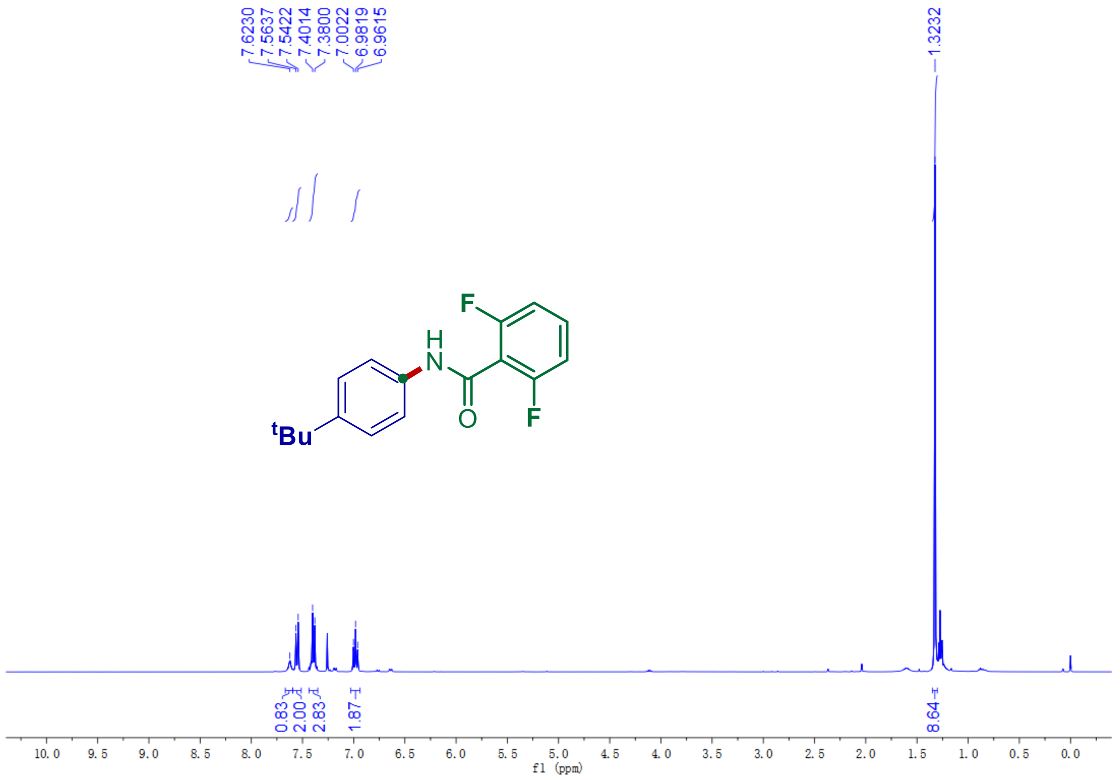

^{1}H NMR (400 MHz, CDCl$_3$) spectrum of compound 117

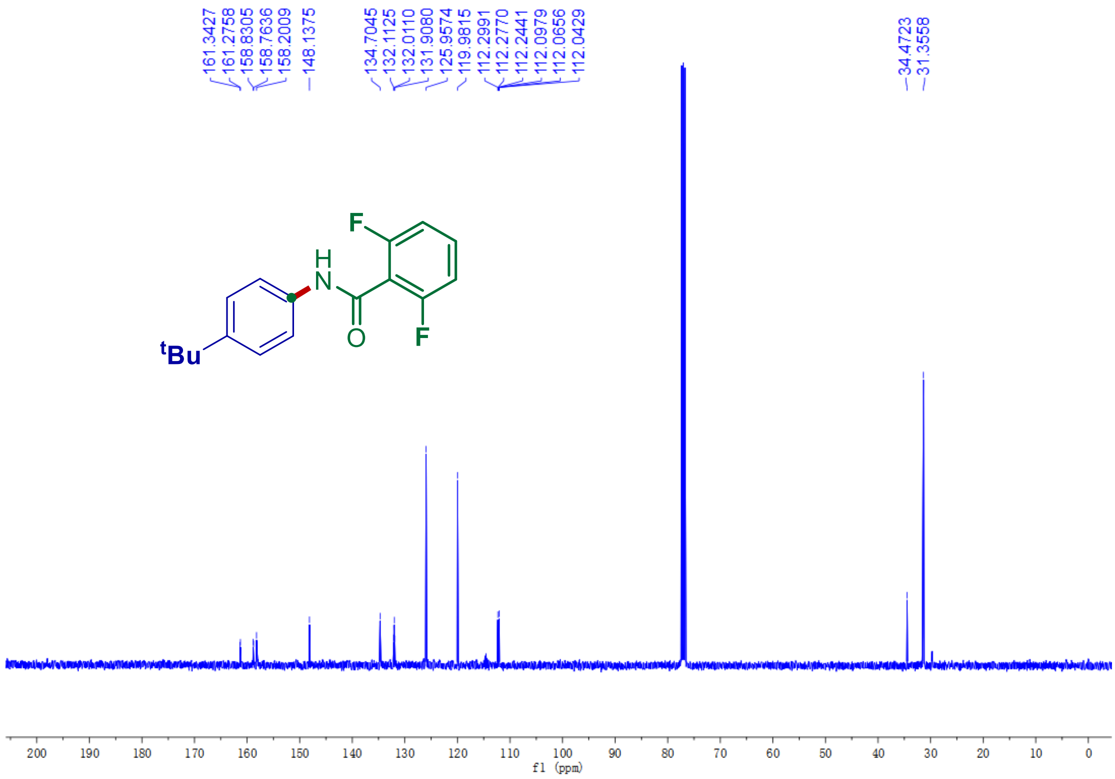

^{13}C NMR (100 MHz, $CDCl_3$) spectrum of compound 117

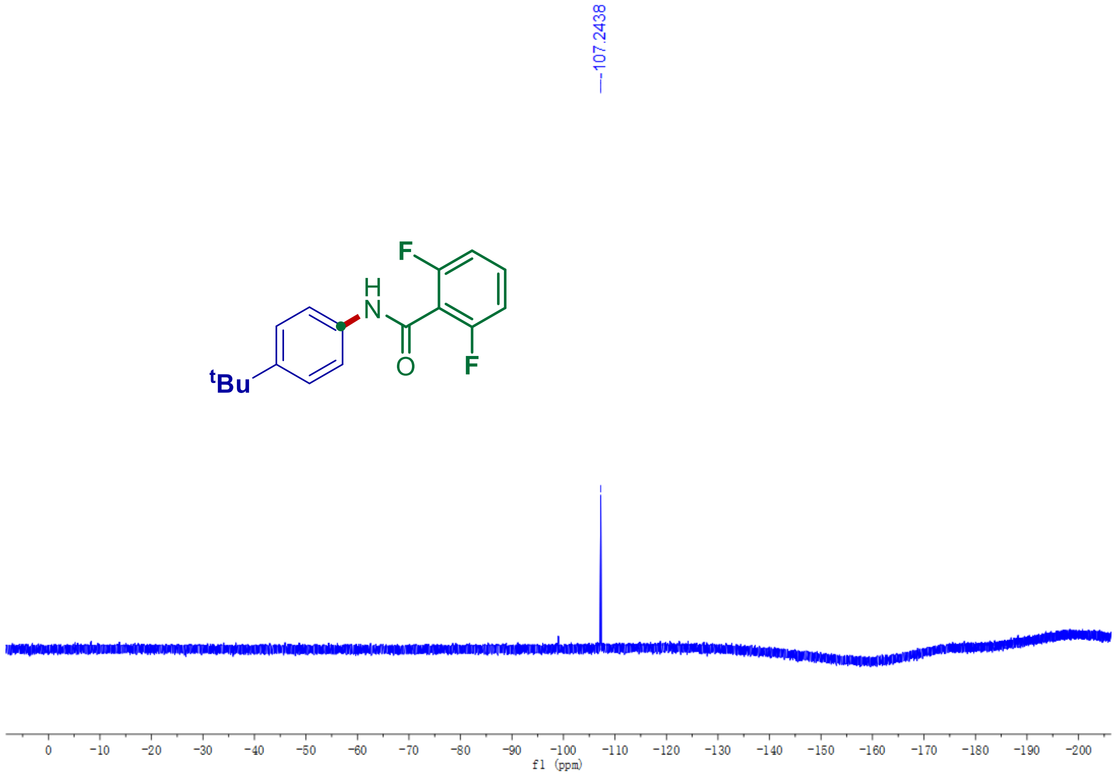

^{19}F NMR (376 MHz, $CDCl_3$) spectrum of compound 117

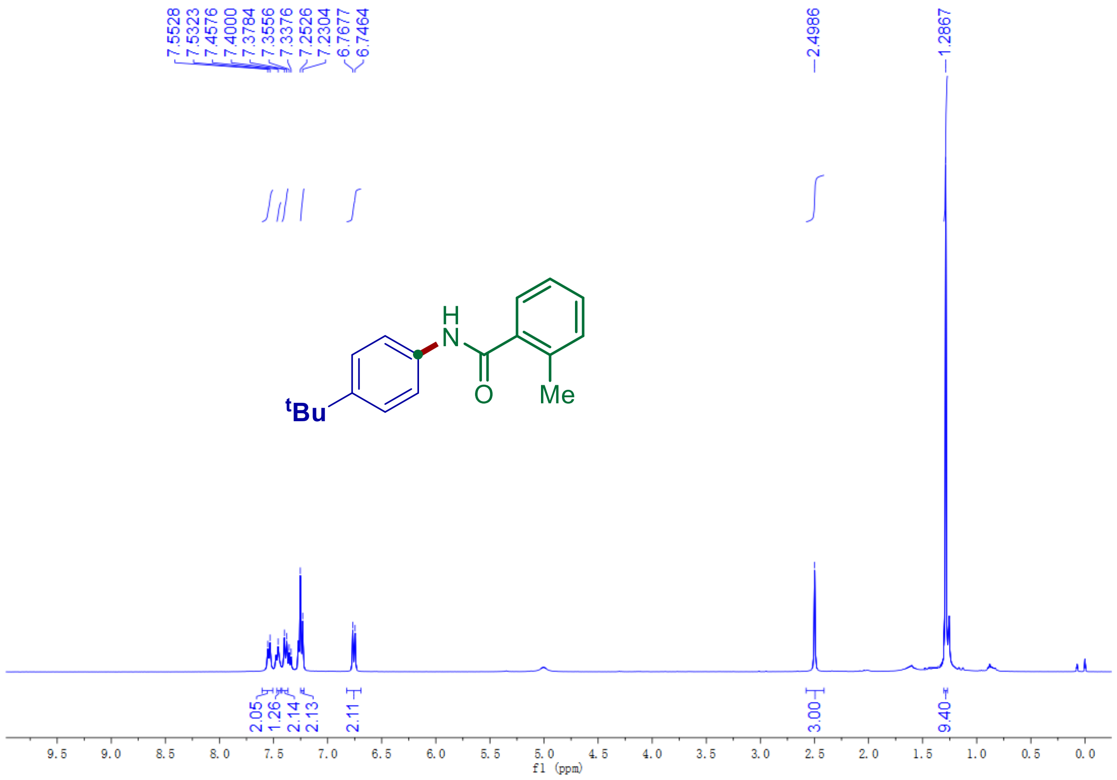

^{1}H NMR (400 MHz, $CDCl_3$) spectrum of compound 118

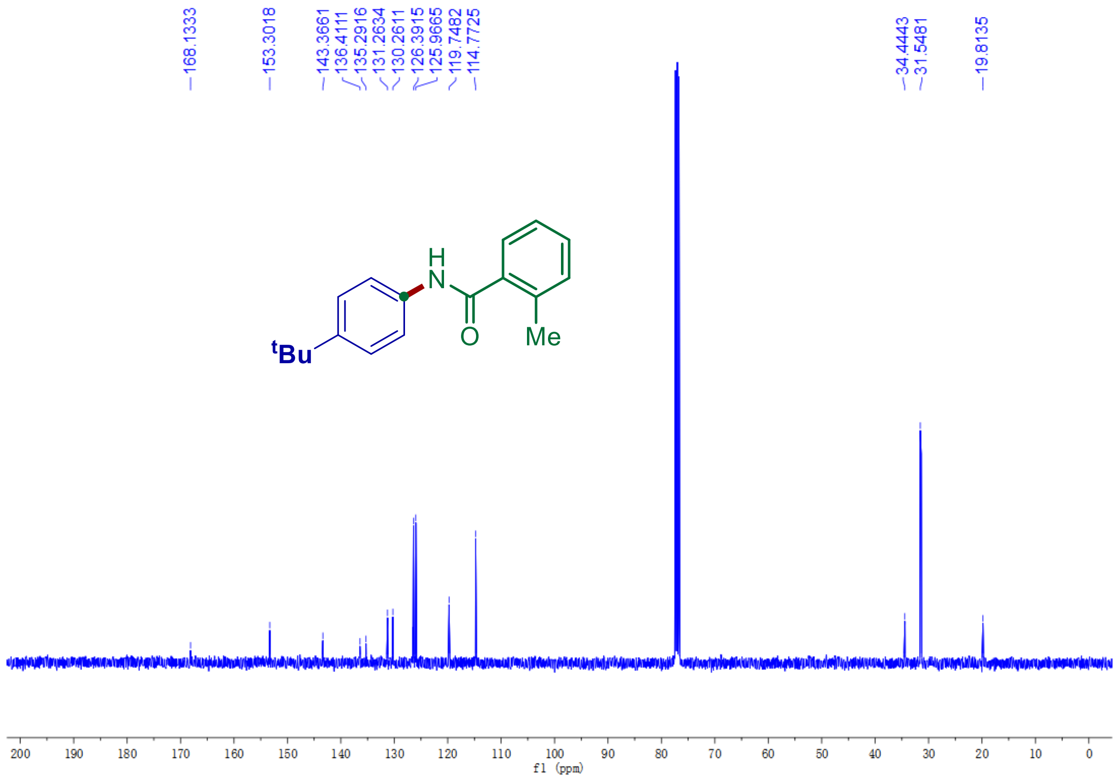

^{13}C NMR (100 MHz, $CDCl_3$) spectrum of compound 118

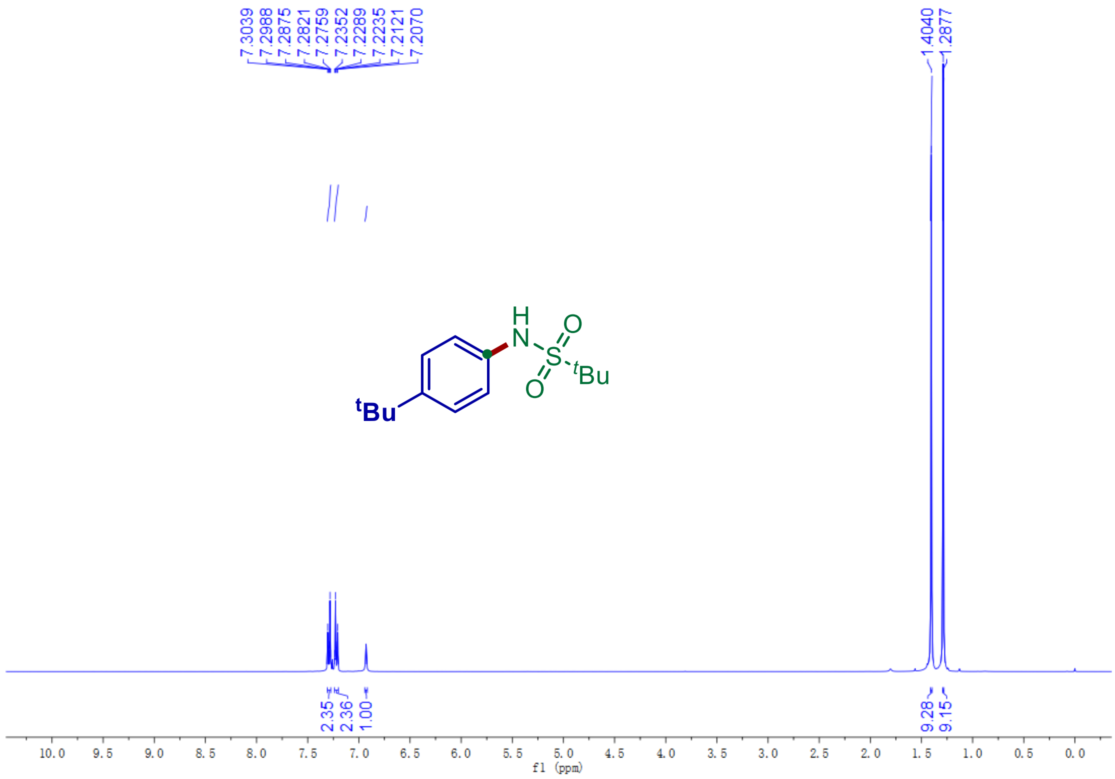

^{1}H NMR (400 MHz, $CDCl_3$) spectrum of compound 119

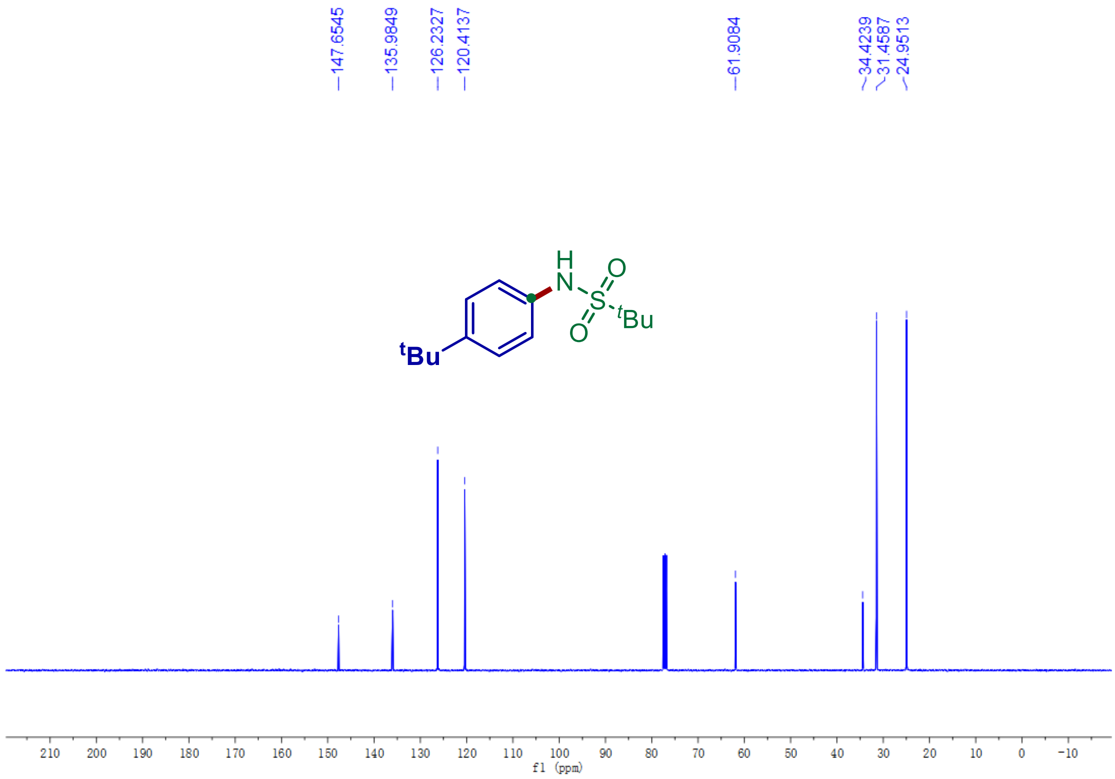

^{13}C NMR (100 MHz, $CDCl_3$) spectrum of compound 119

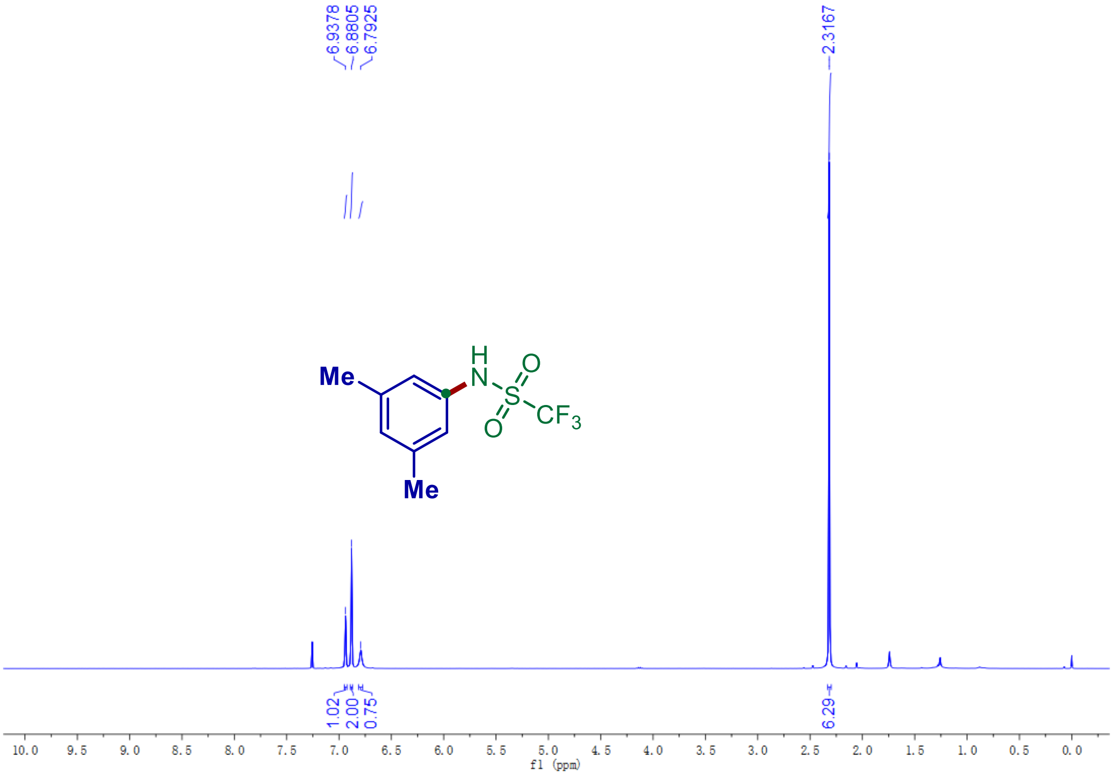

^{1}H NMR (400 MHz, $CDCl_3$) spectrum of compound 120

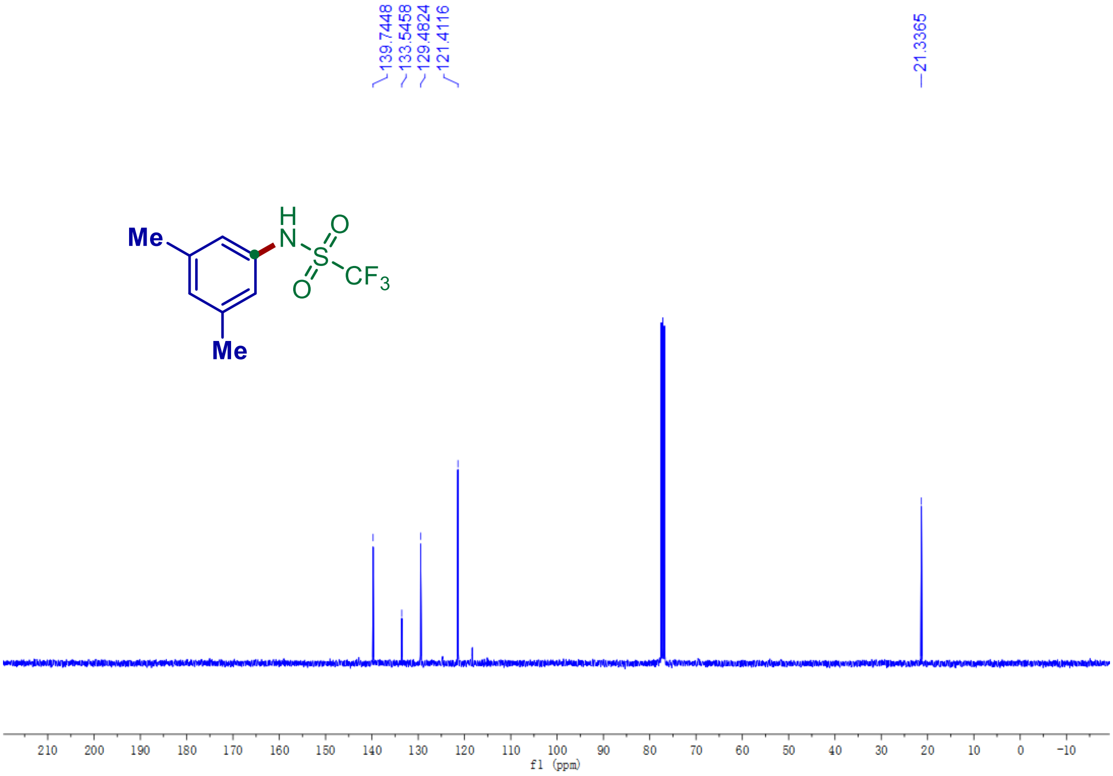

^{13}C NMR (100 MHz, $CDCl_3$) spectrum of compound 120

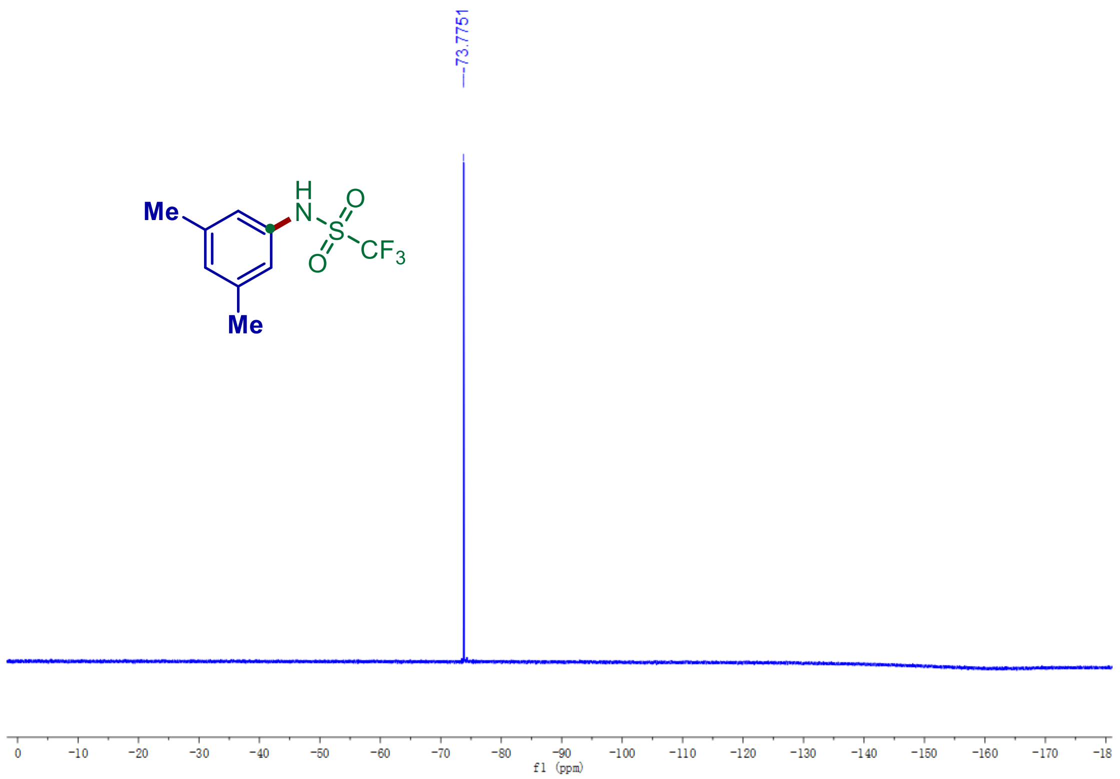

^{19}F NMR (376 MHz, $CDCl_3$) spectrum of compound 120

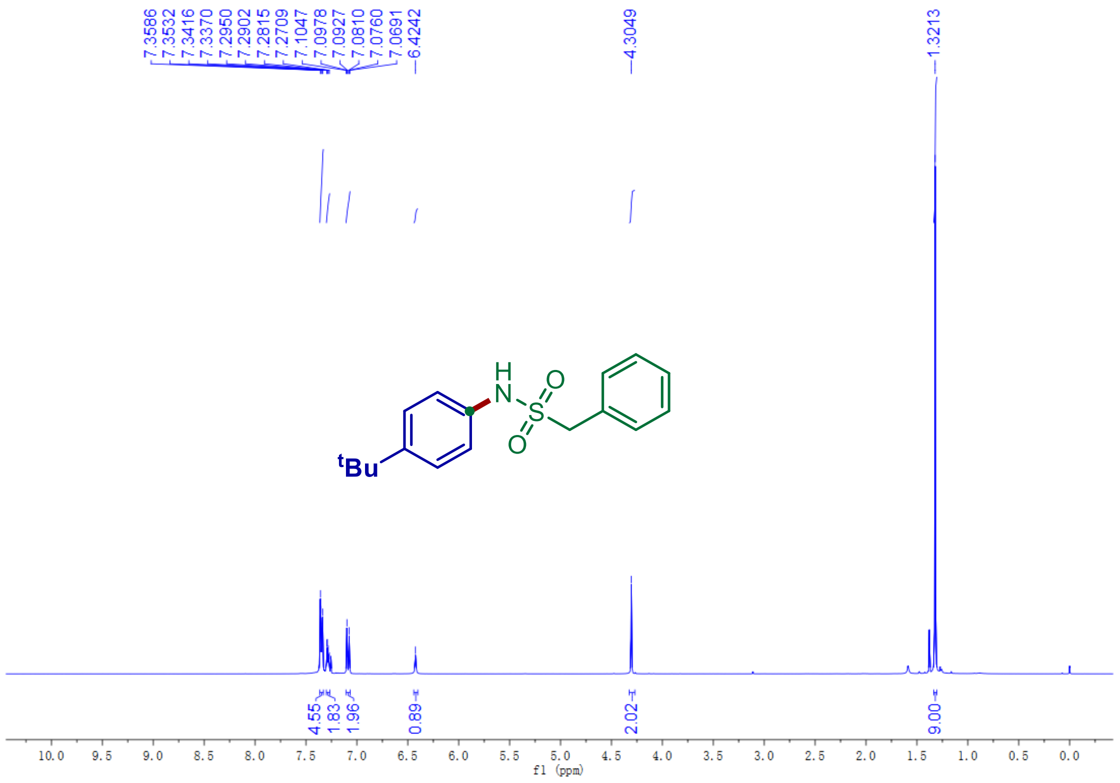

^{1}H NMR (400 MHz, $CDCl_3$) spectrum of compound 121

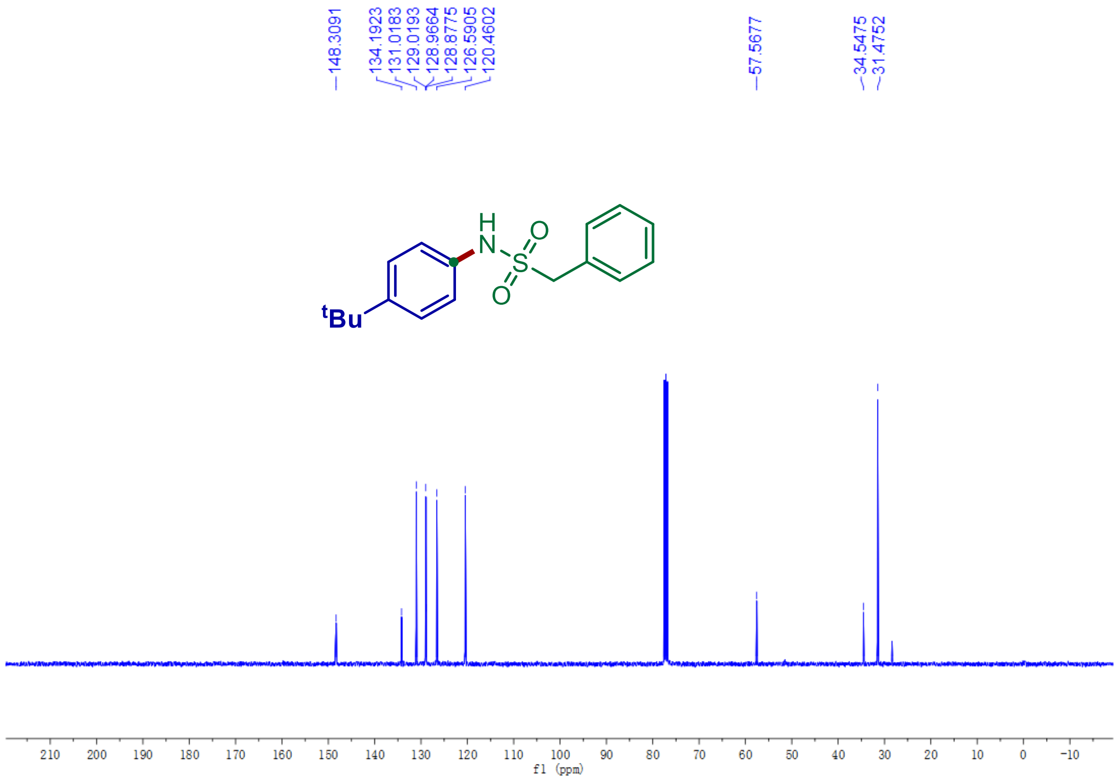

^{13}C NMR (100 MHz, $CDCl_3$) spectrum of compound 121

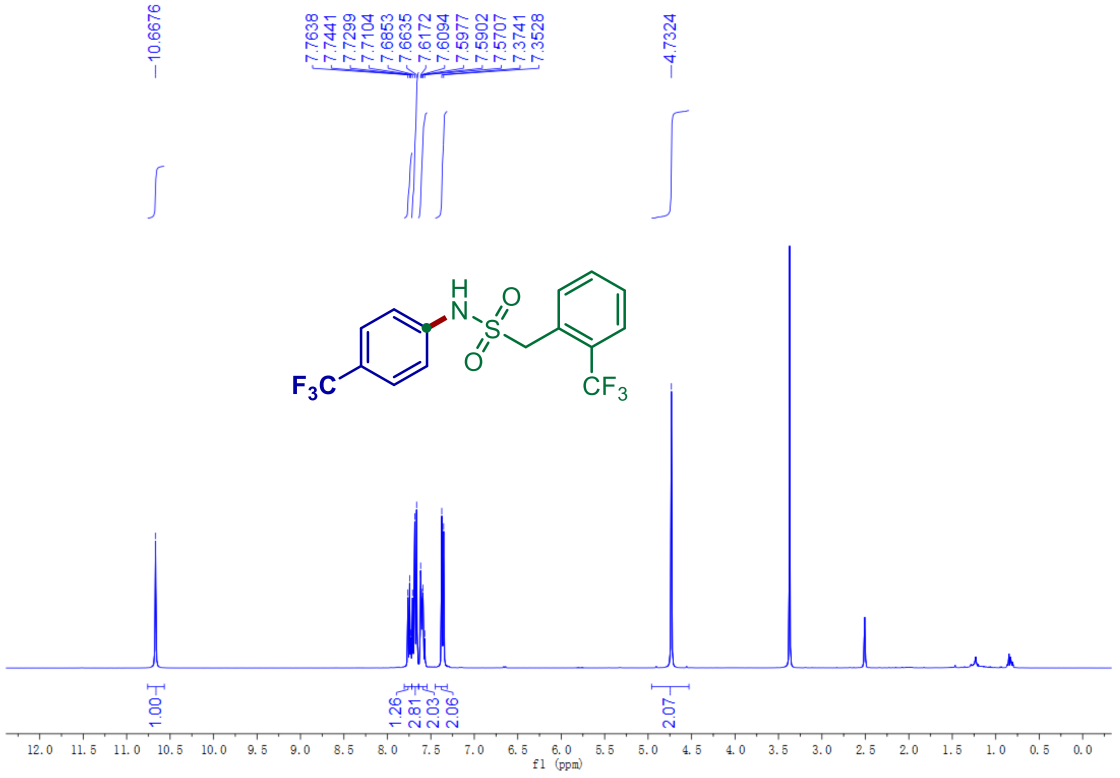

^{1}H NMR (400 MHz, d_6-DMSO) spectrum of compound 122

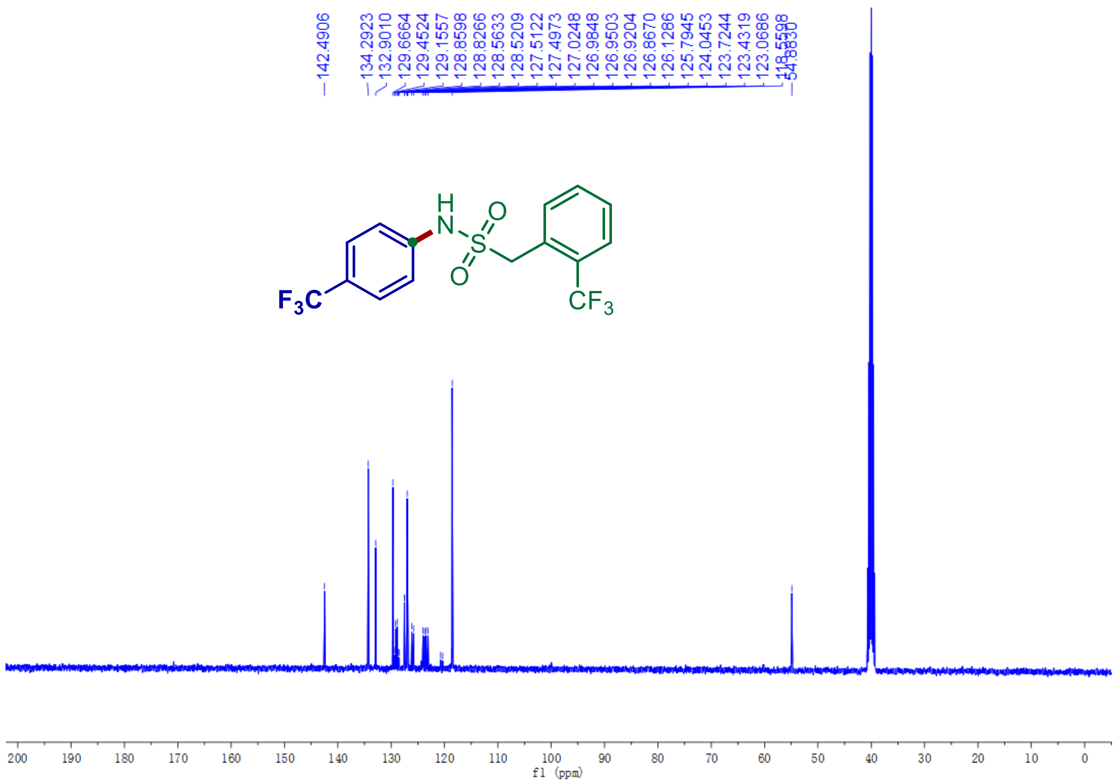

^{13}C NMR (100 MHz, d_6-DMSO) spectrum of compound 122

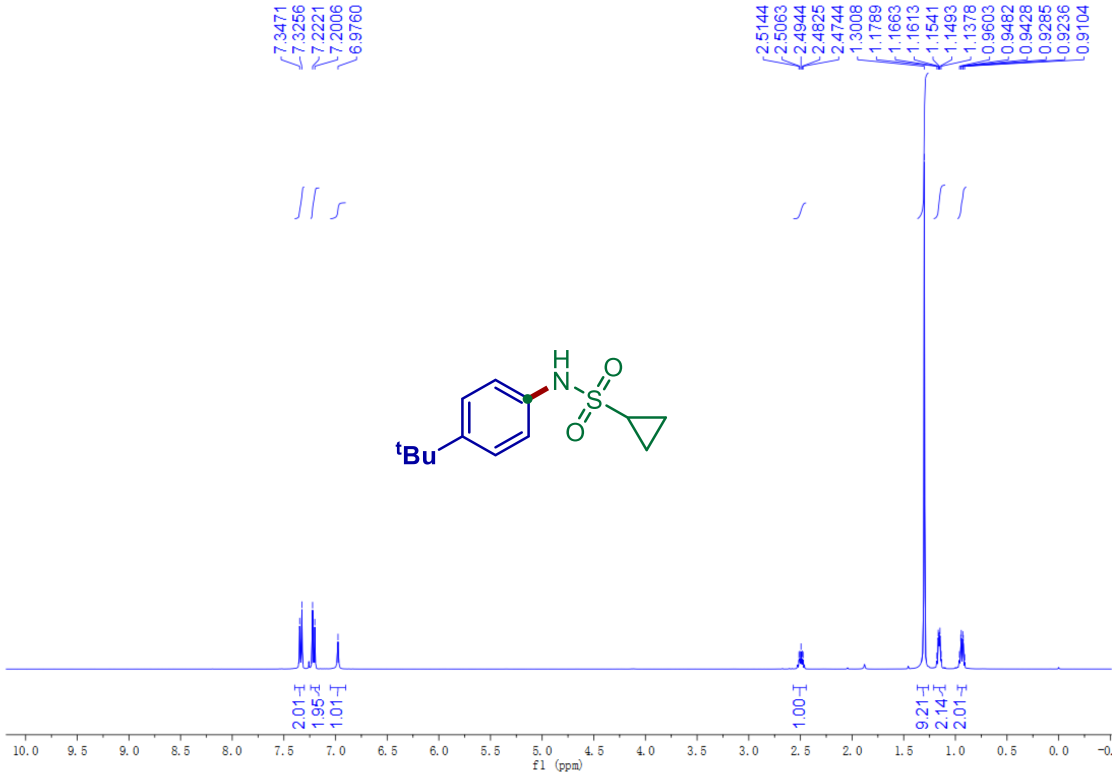

^{1}H NMR (400 MHz, $CDCl_3$) spectrum of compound 123

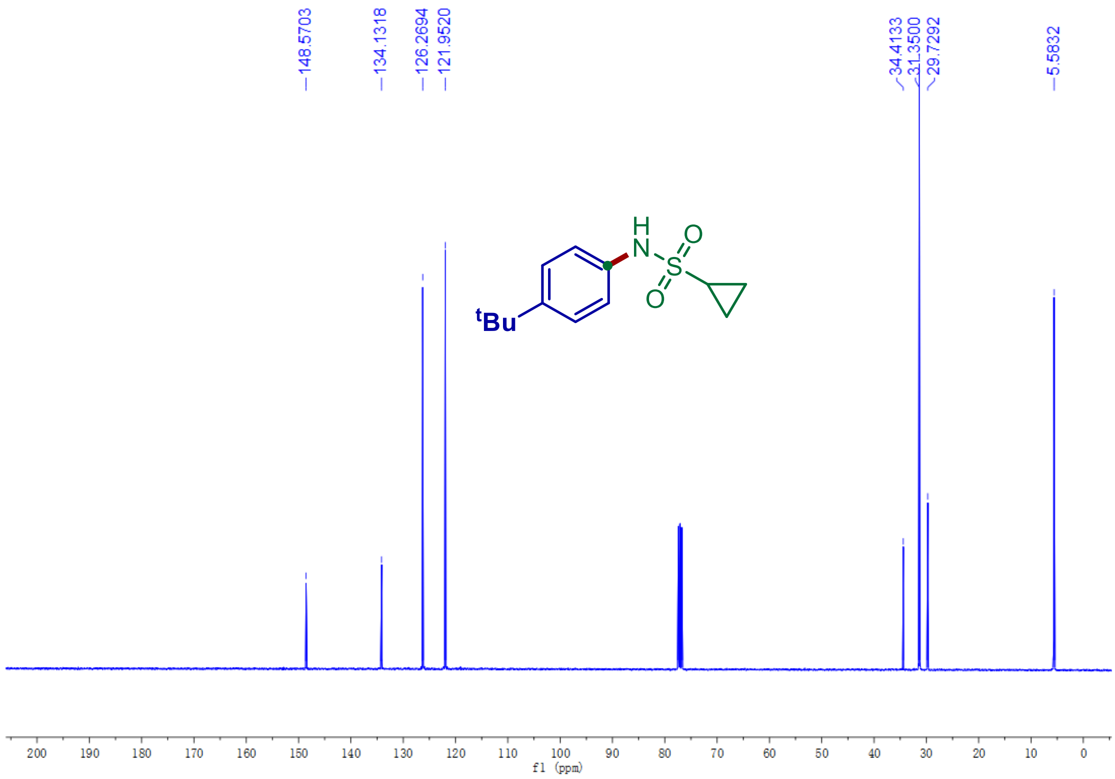

^{13}C NMR (100 MHz, $CDCl_3$) spectrum of compound 123

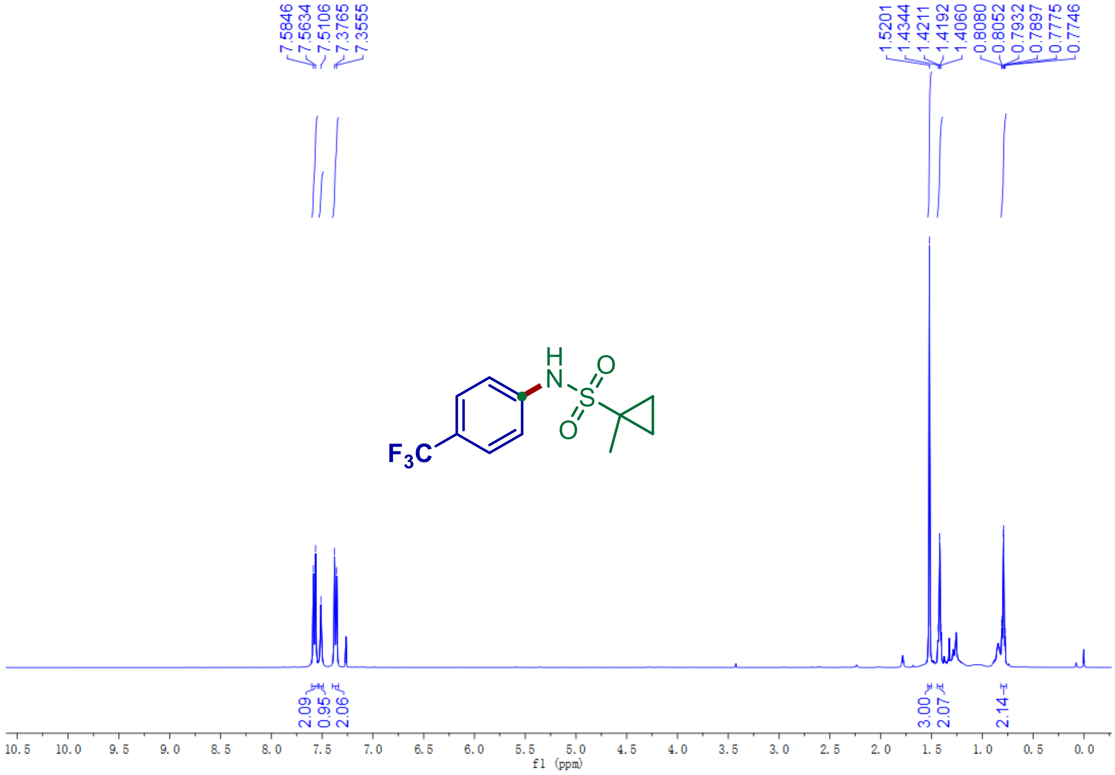

^{1}H NMR (400 MHz, $CDCl_3$) spectrum of compound 124

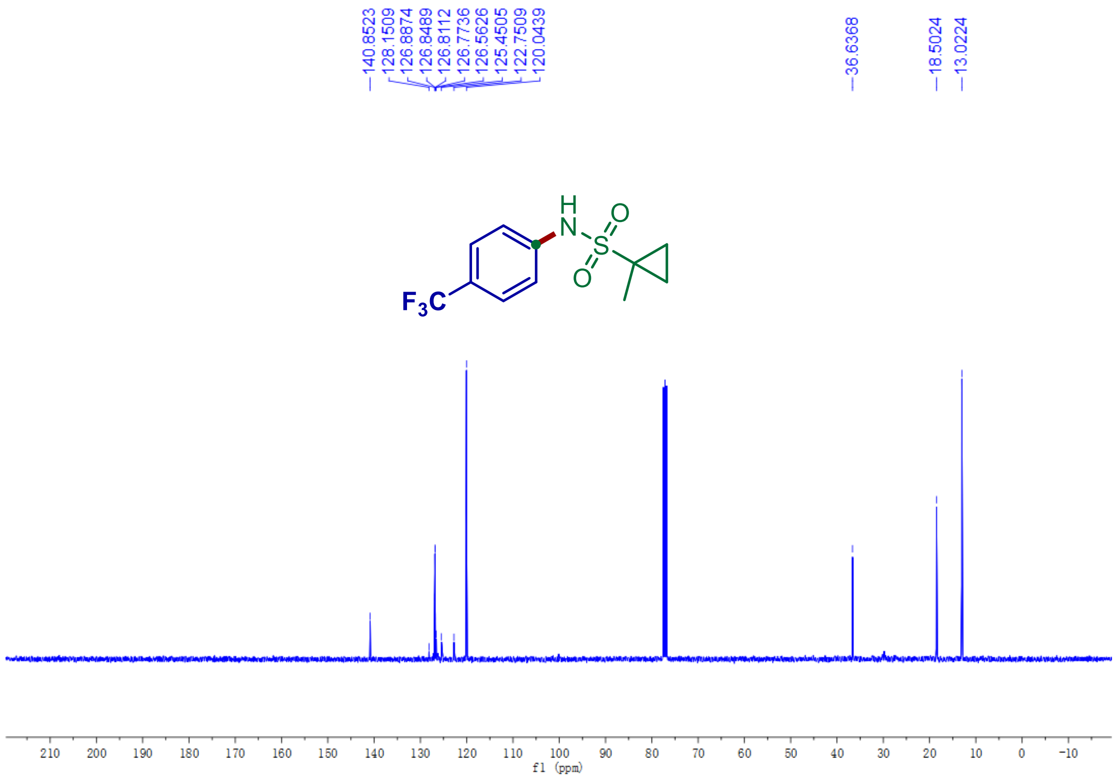

^{13}C NMR (100 MHz, $CDCl_3$) spectrum of compound 124

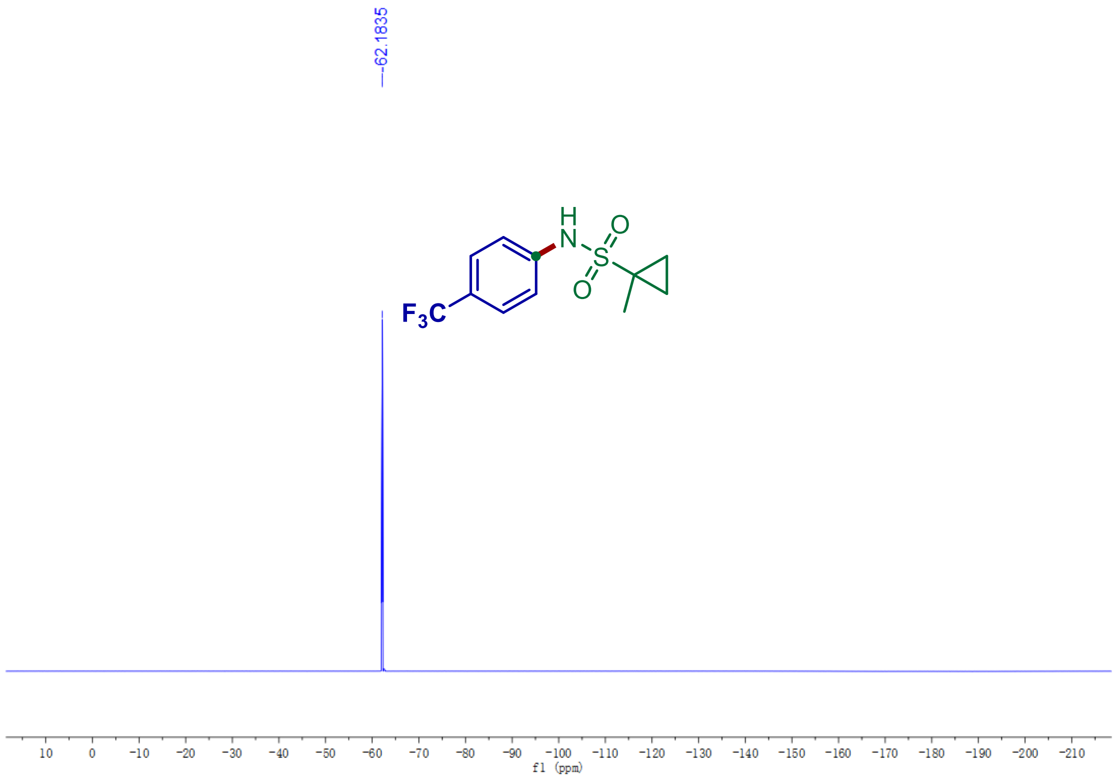

^{19}F NMR (376 MHz, $CDCl_3$) spectrum of compound 124

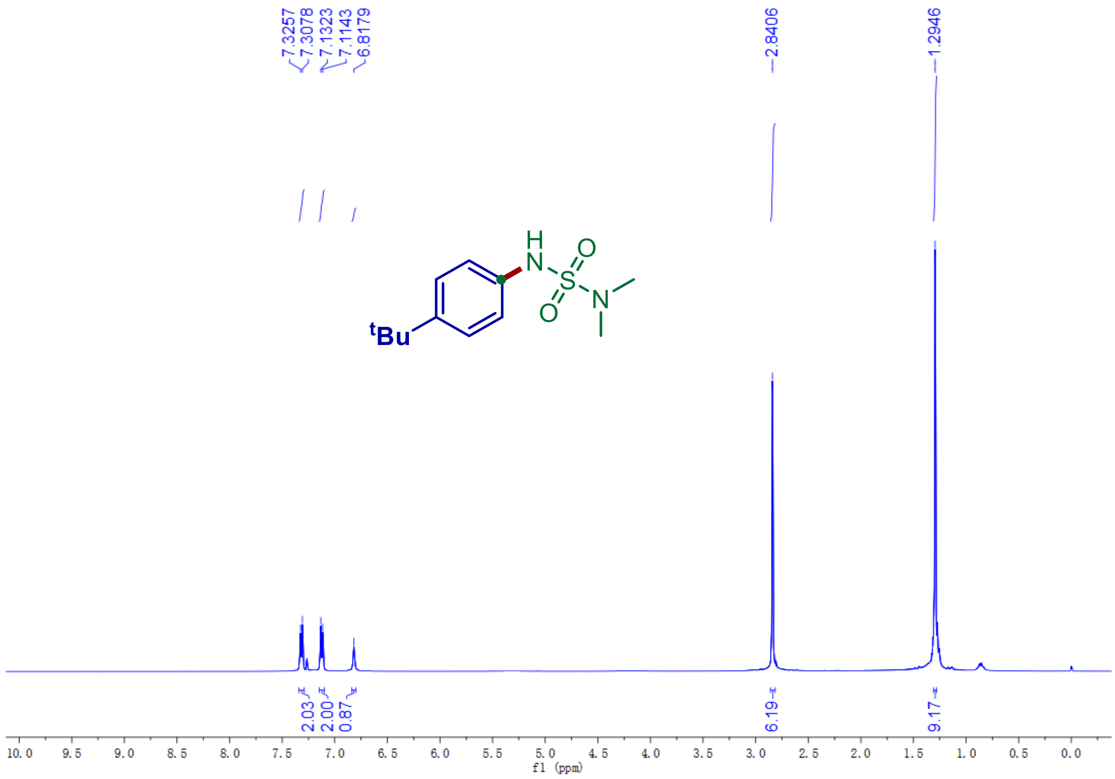

^{1}H NMR (400 MHz, $CDCl_3$) spectrum of compound 125

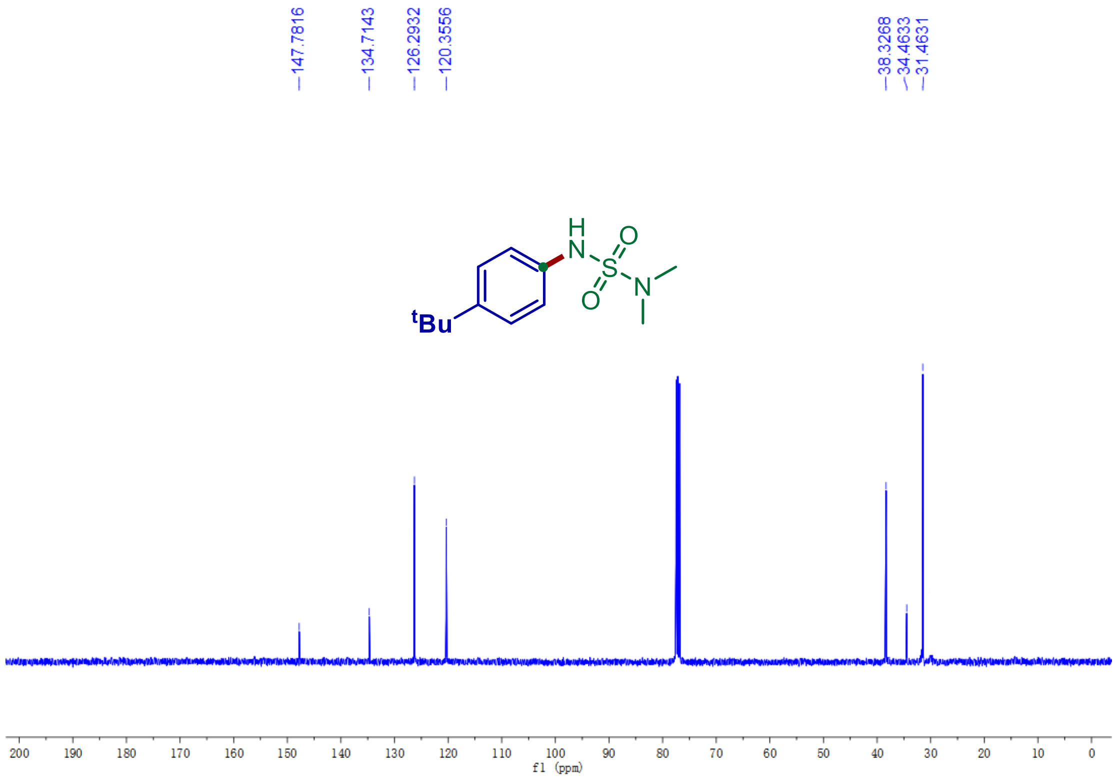

^{13}C NMR (100 MHz, $CDCl_3$) spectrum of compound 125

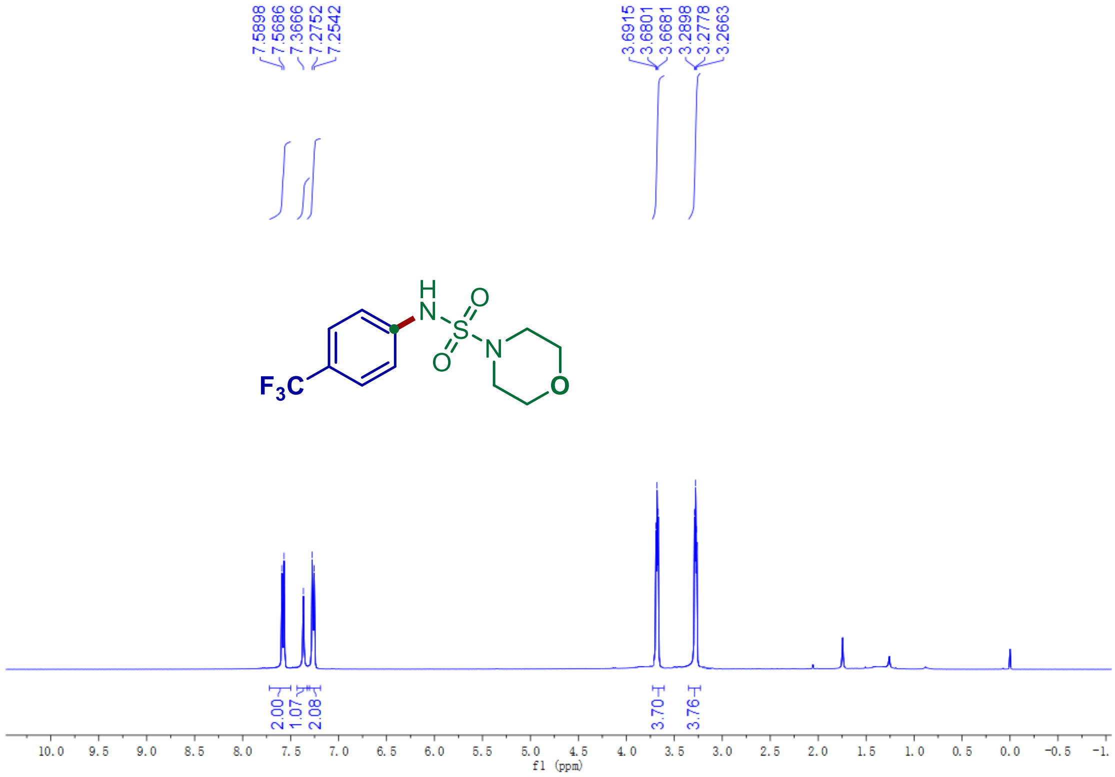

^{1}H NMR (400 MHz, $CDCl_3$) spectrum of compound 126

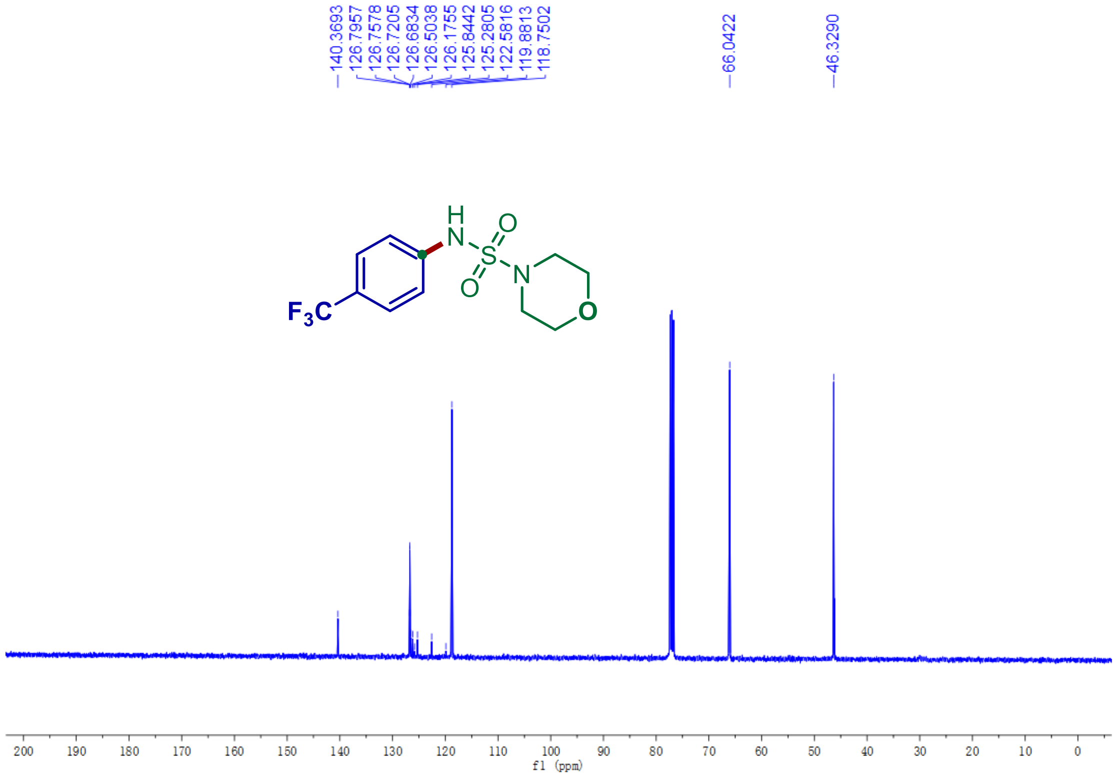

^{13}C NMR (100 MHz, $CDCl_3$) spectrum of compound 126

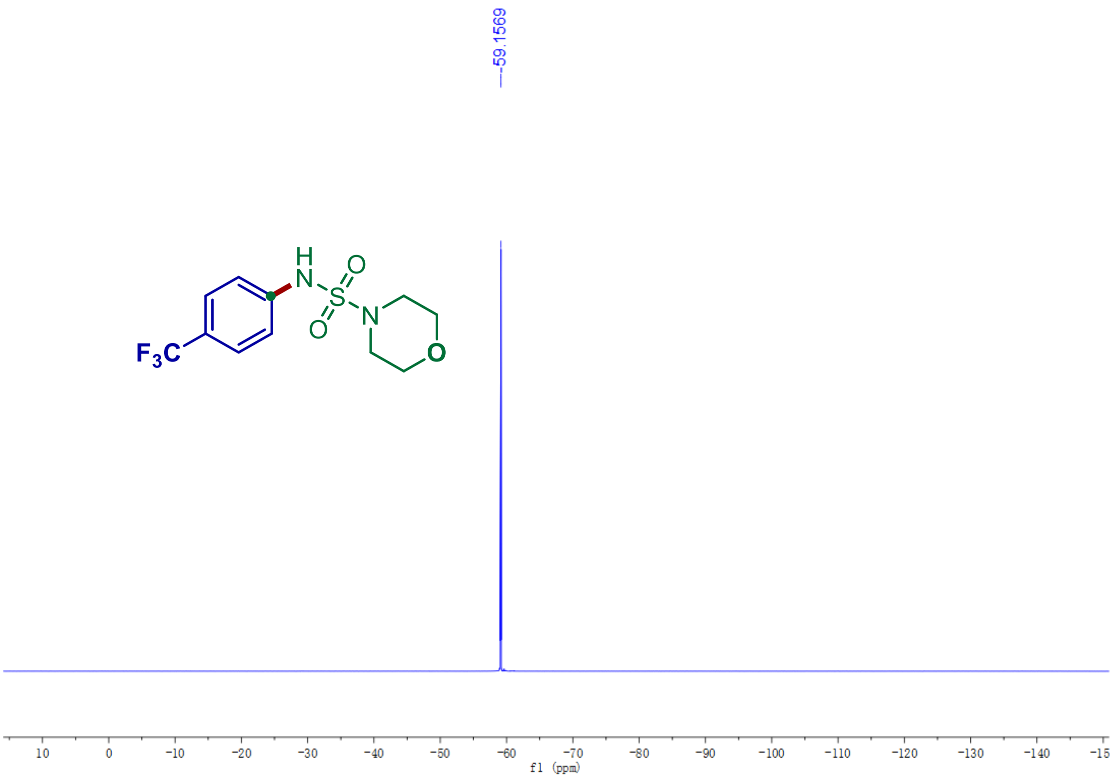

^{19}F NMR (376 MHz, $CDCl_3$) spectrum of compound 126

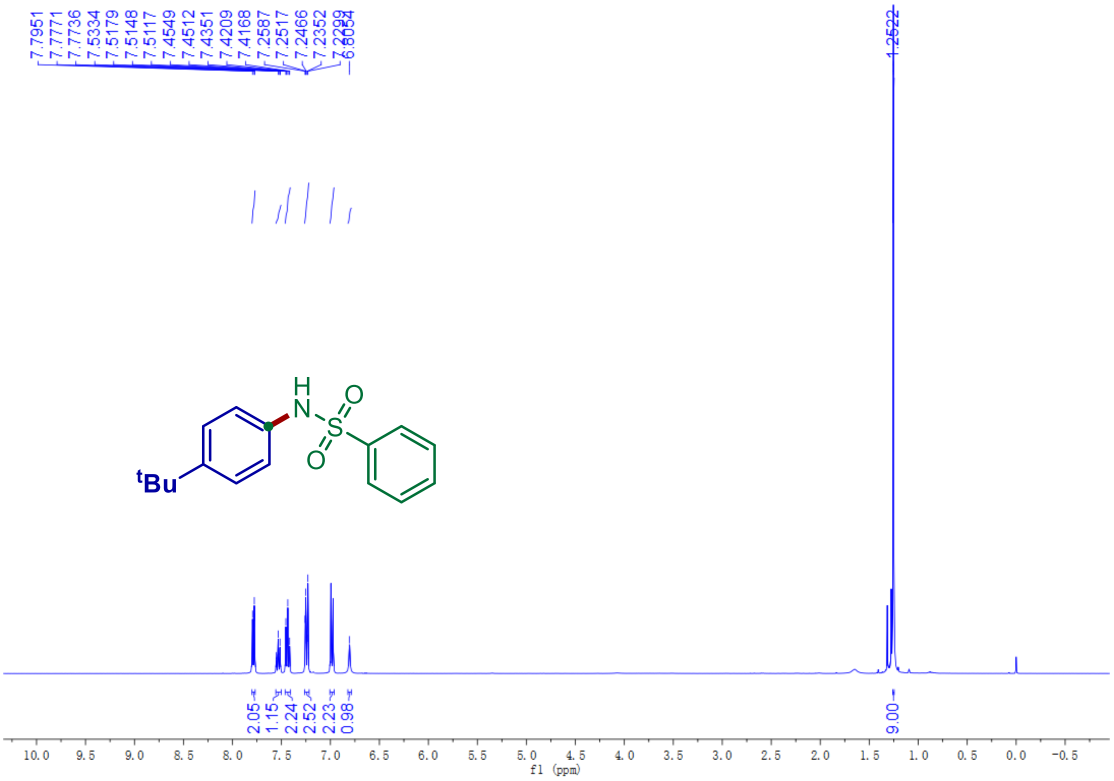

^{1}H NMR (400 MHz, $CDCl_3$) spectrum of compound 127

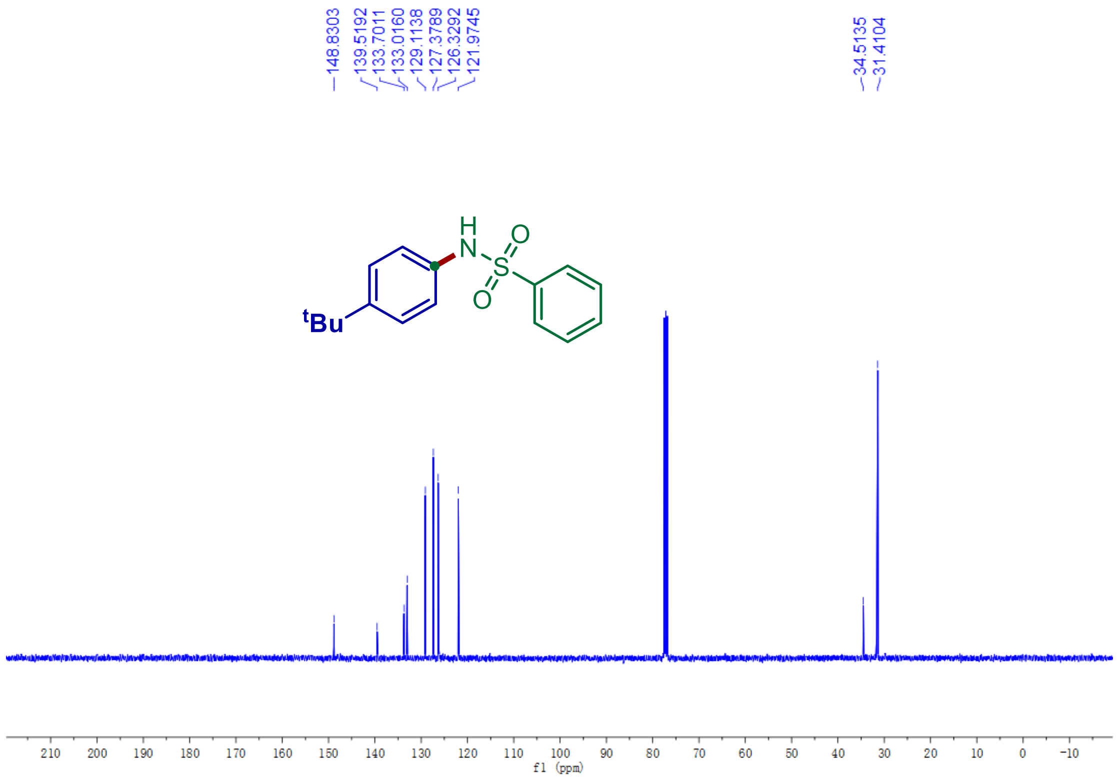

^{13}C NMR (100 MHz, CDCl$_3$) spectrum of compound 127

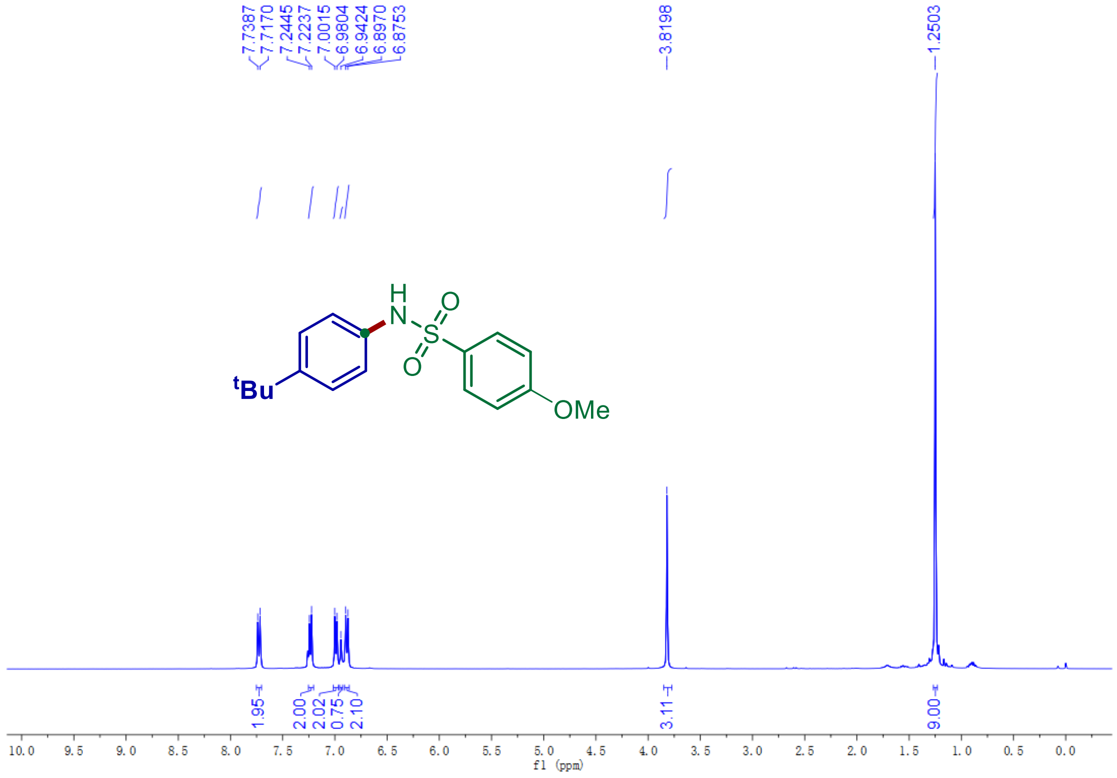

^{1}H NMR (400 MHz, CDCl$_3$) spectrum of compound 128

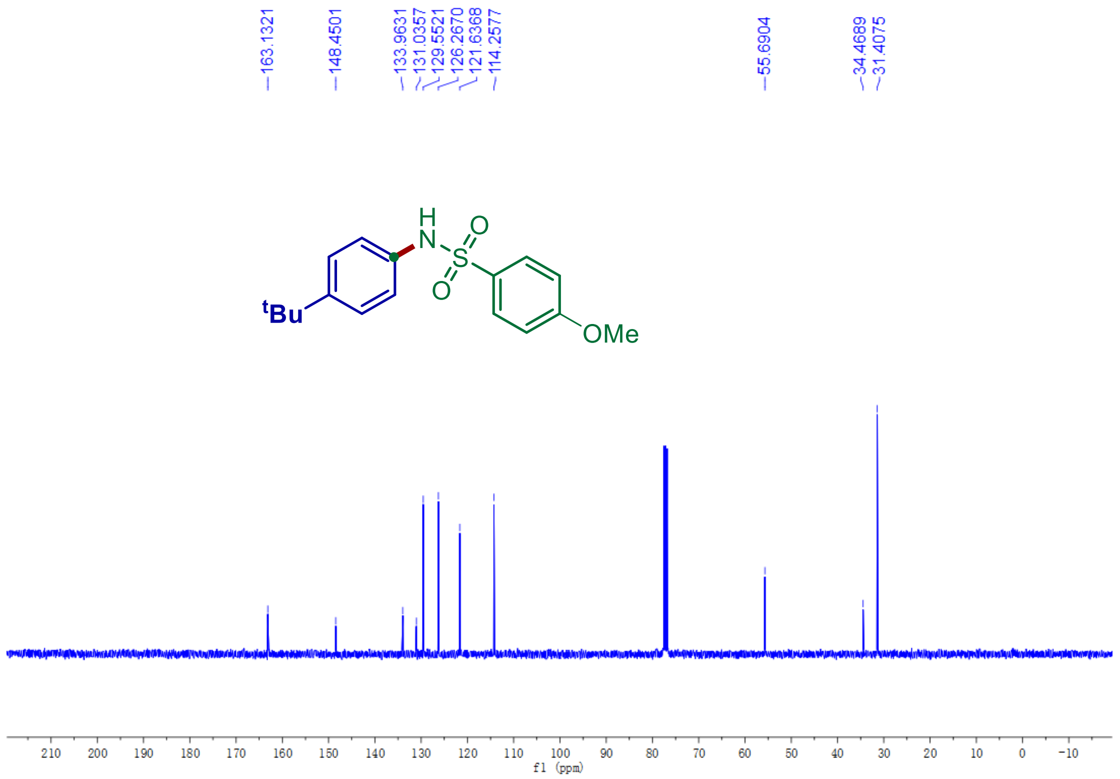

^{13}C NMR (100 MHz, $CDCl_3$) spectrum of compound 128

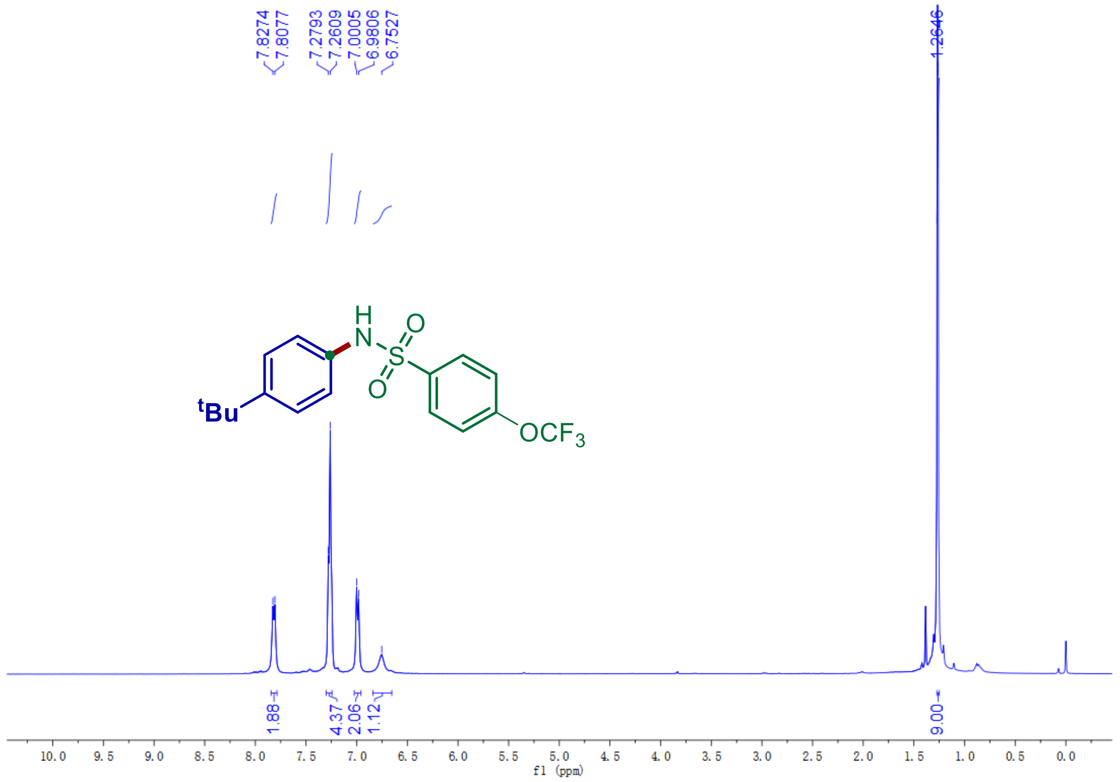

^{1}H NMR (400 MHz, $CDCl_3$) spectrum of compound 129

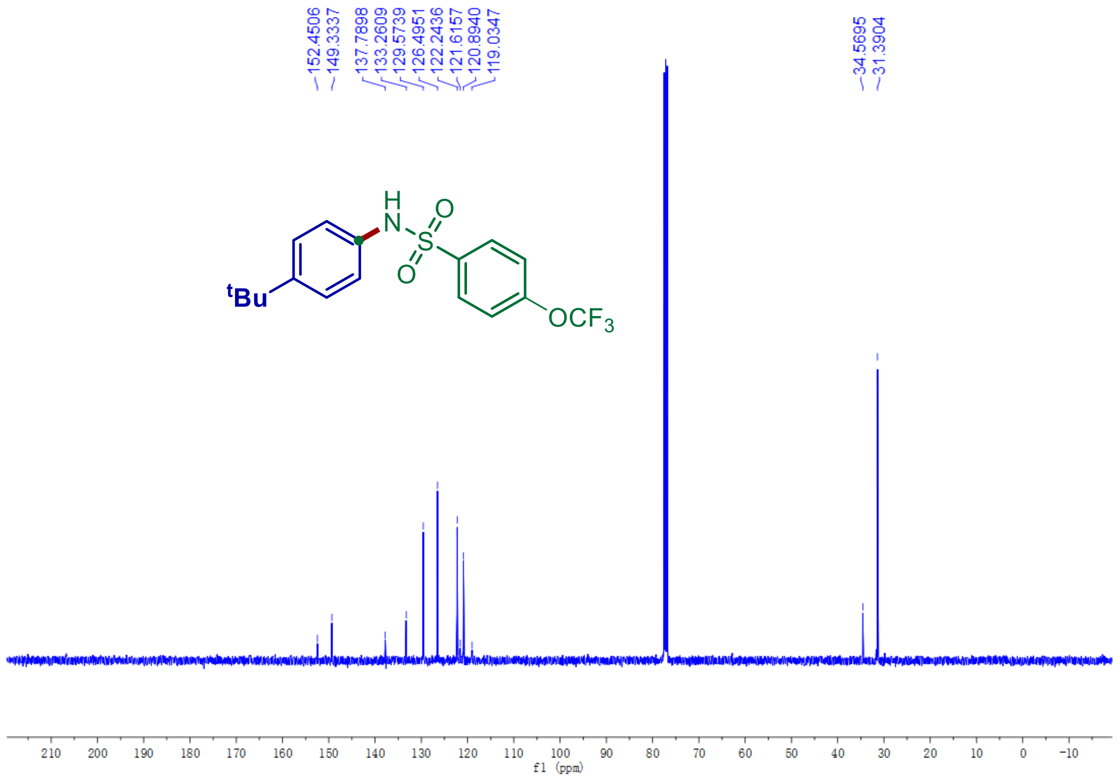

^{13}C NMR (100 MHz, $CDCl_3$) spectrum of compound 129

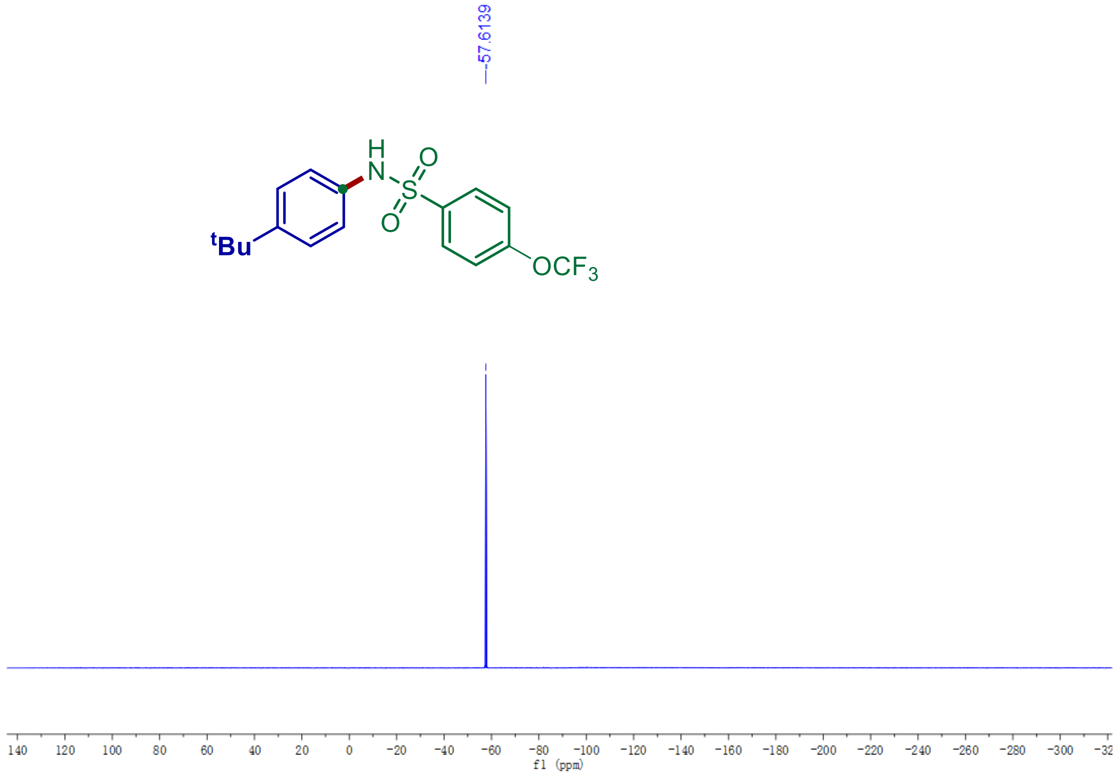

^{19}F NMR (376 MHz, $CDCl_3$) spectrum of compound 129

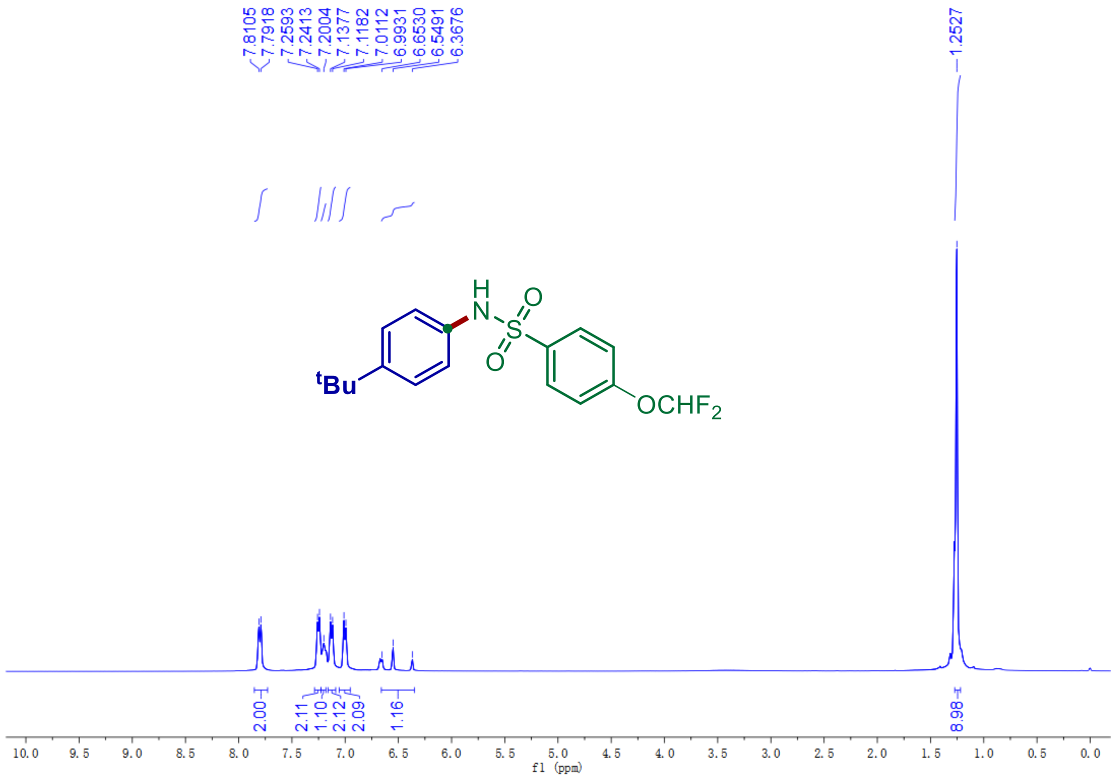

^{1}H NMR (400 MHz, CDCl$_3$) spectrum of compound 130

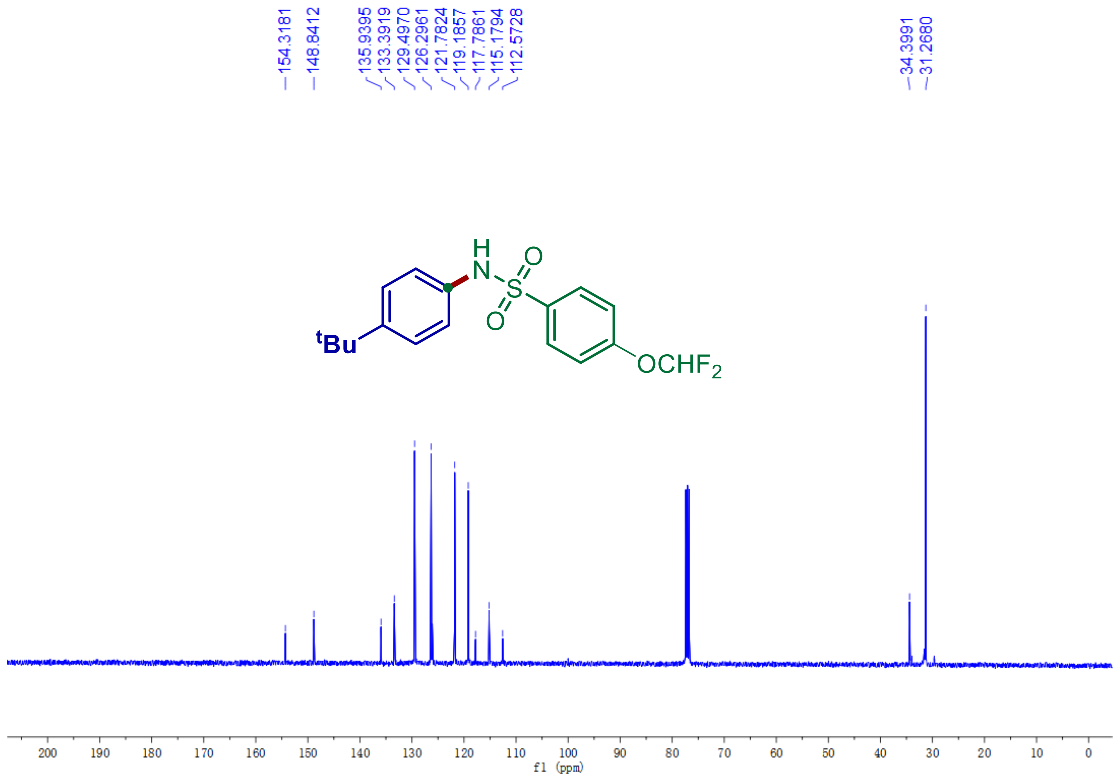

^{13}C NMR (100 MHz, CDCl$_3$) spectrum of compound 130

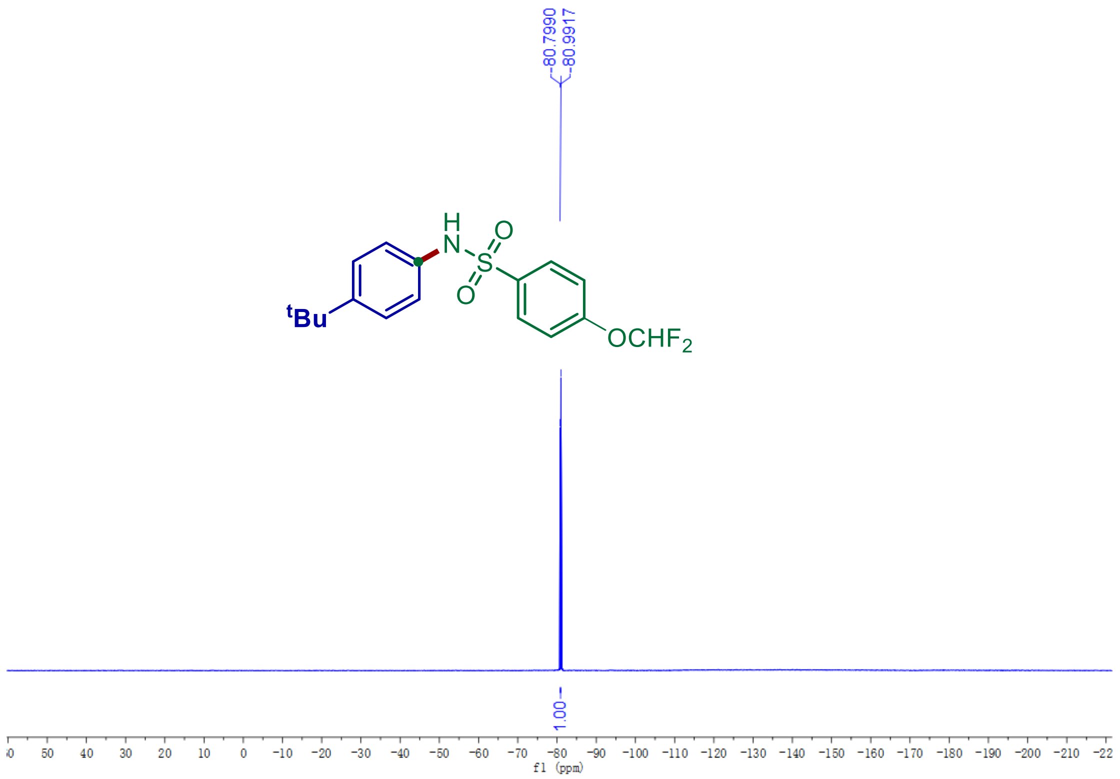

^{19}F NMR (376 MHz, $CDCl_3$) spectrum of compound 130

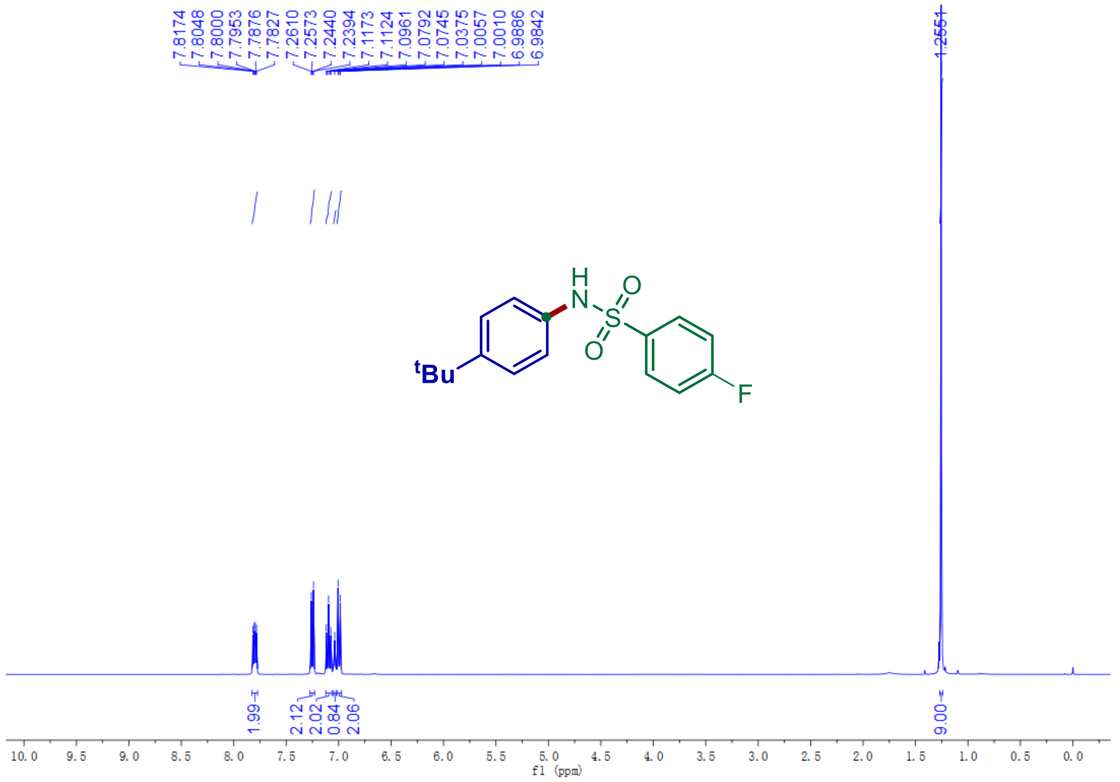

^{1}H NMR (400 MHz, $CDCl_3$) spectrum of compound 131

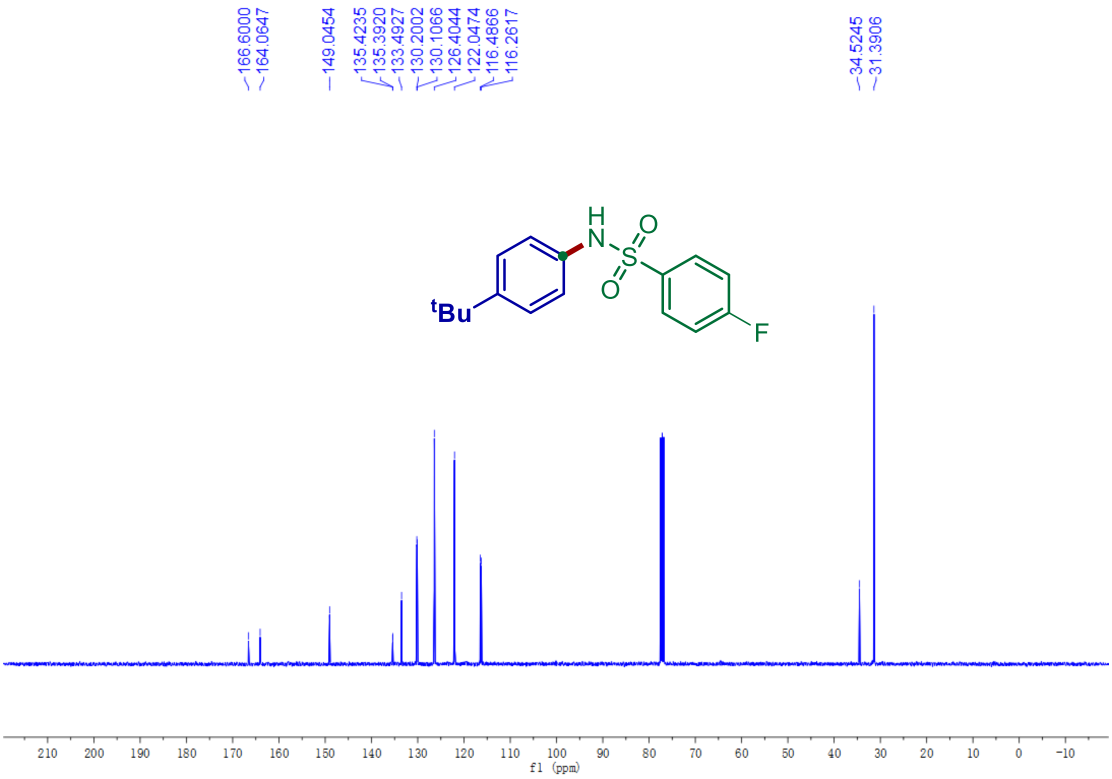

^{13}C NMR (100 MHz, $CDCl_3$) spectrum of compound 131

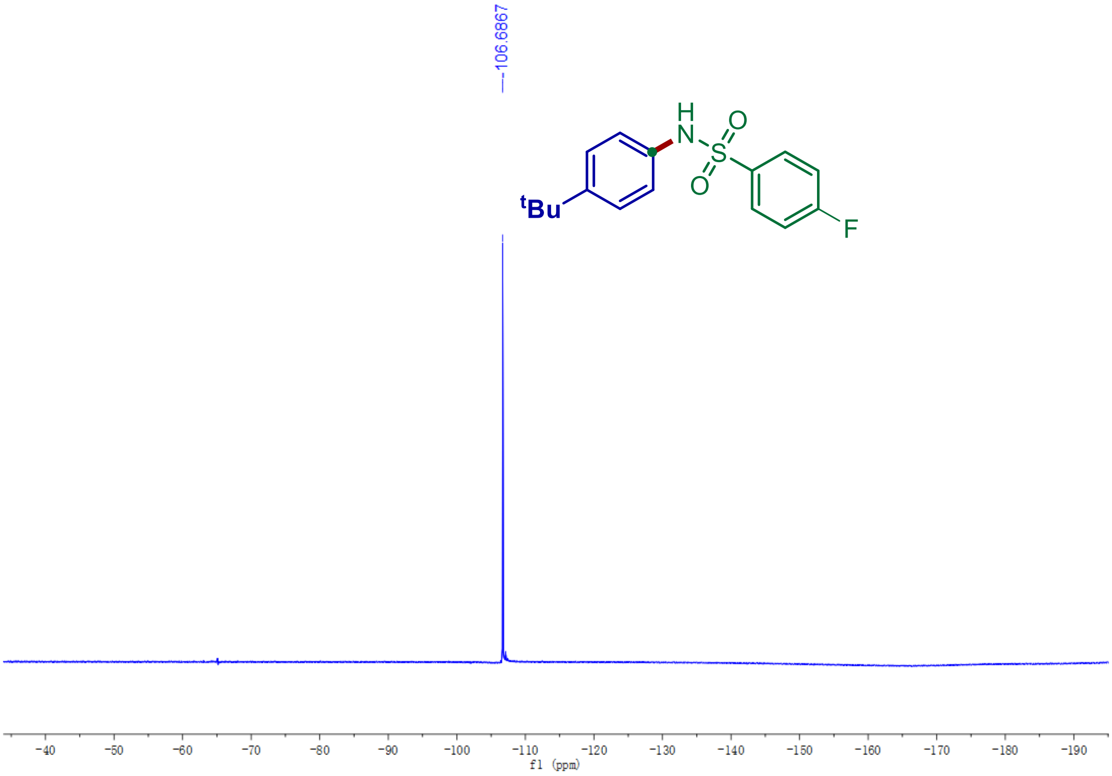

^{19}F NMR (376 MHz, $CDCl_3$) spectrum of compound 131

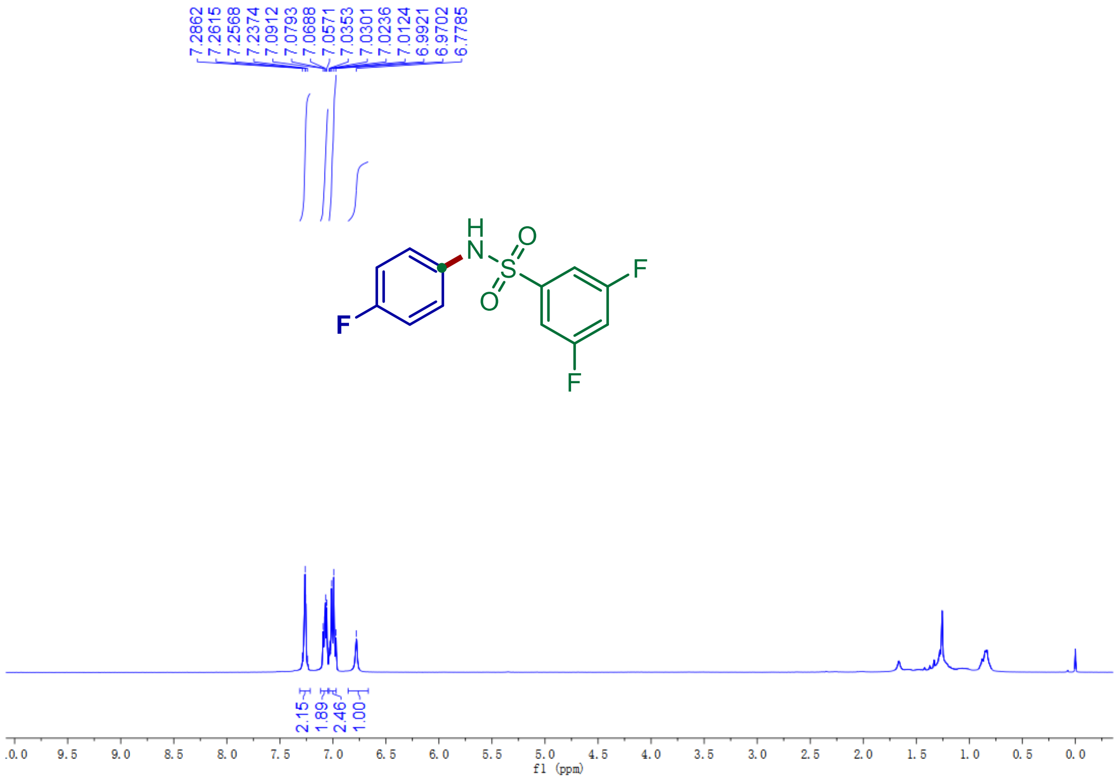

^{1}H NMR (400 MHz, $CDCl_3$) spectrum of compound 132

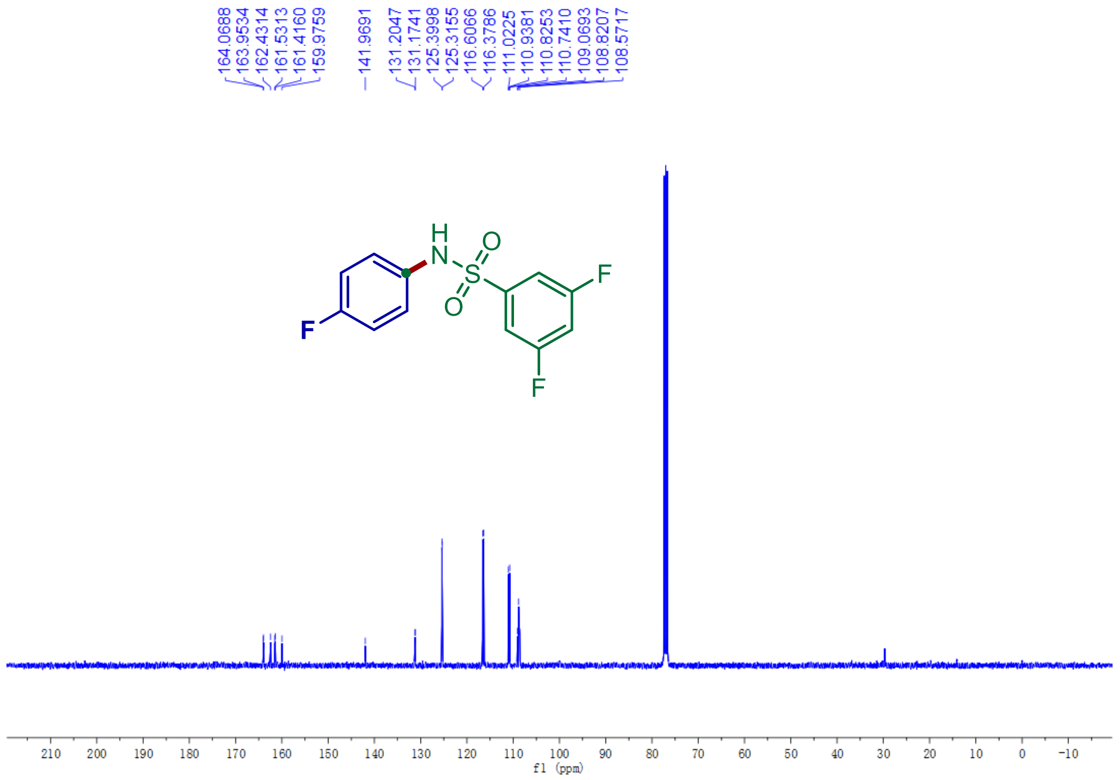

^{13}C NMR (100 MHz, $CDCl_3$) spectrum of compound 132

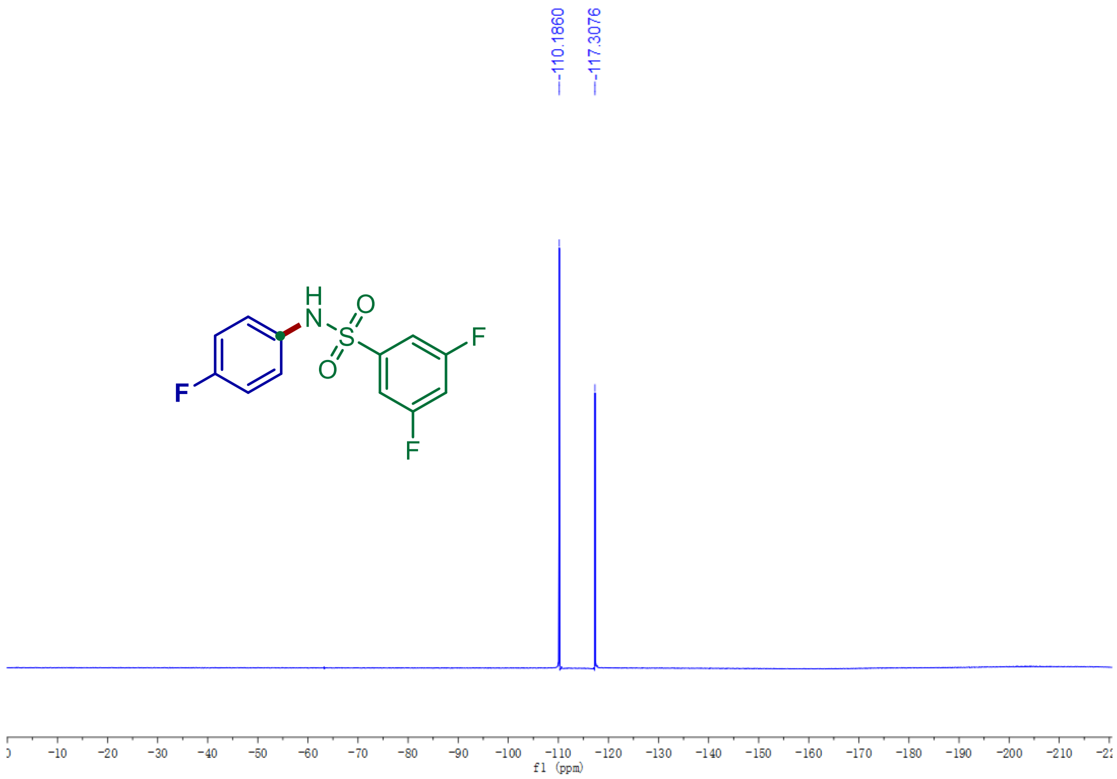

^{13}C NMR (100 MHz, $CDCl_3$) spectrum of compound 132

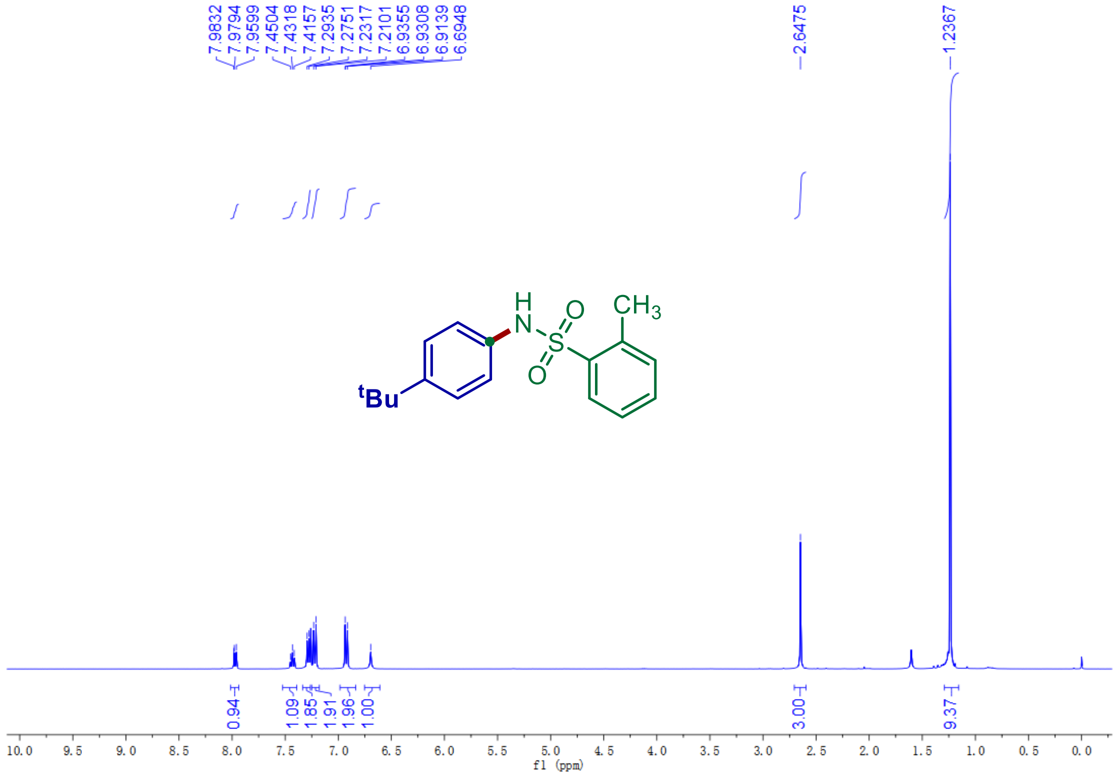

^{1}H NMR (400 MHz, $CDCl_3$) spectrum of compound 133

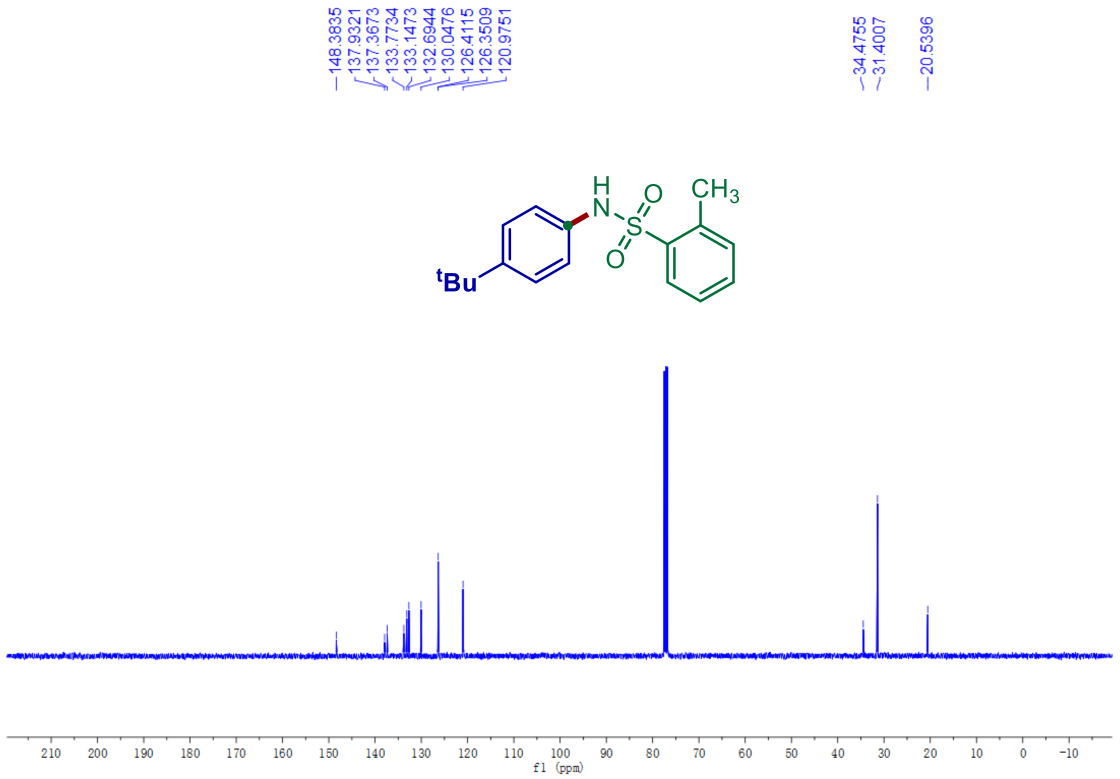

^{13}C NMR (100 MHz, $CDCl_3$) spectrum of compound 133

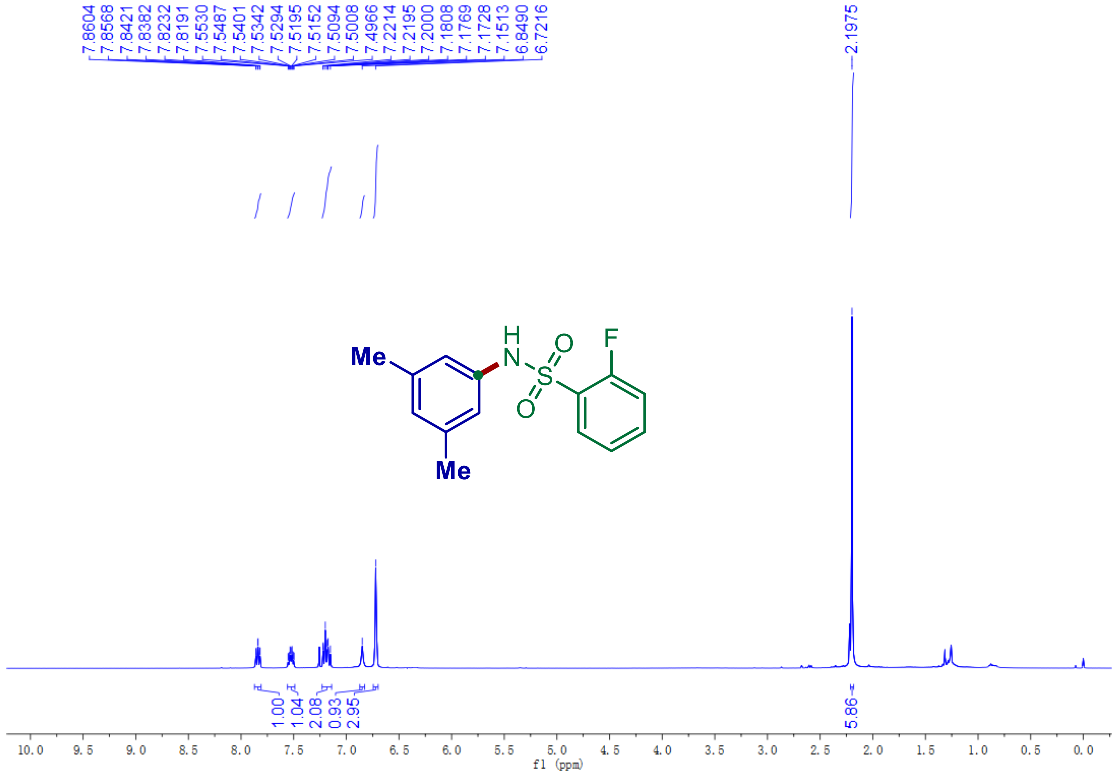

^{1}H NMR (400 MHz, $CDCl_3$) spectrum of compound 134

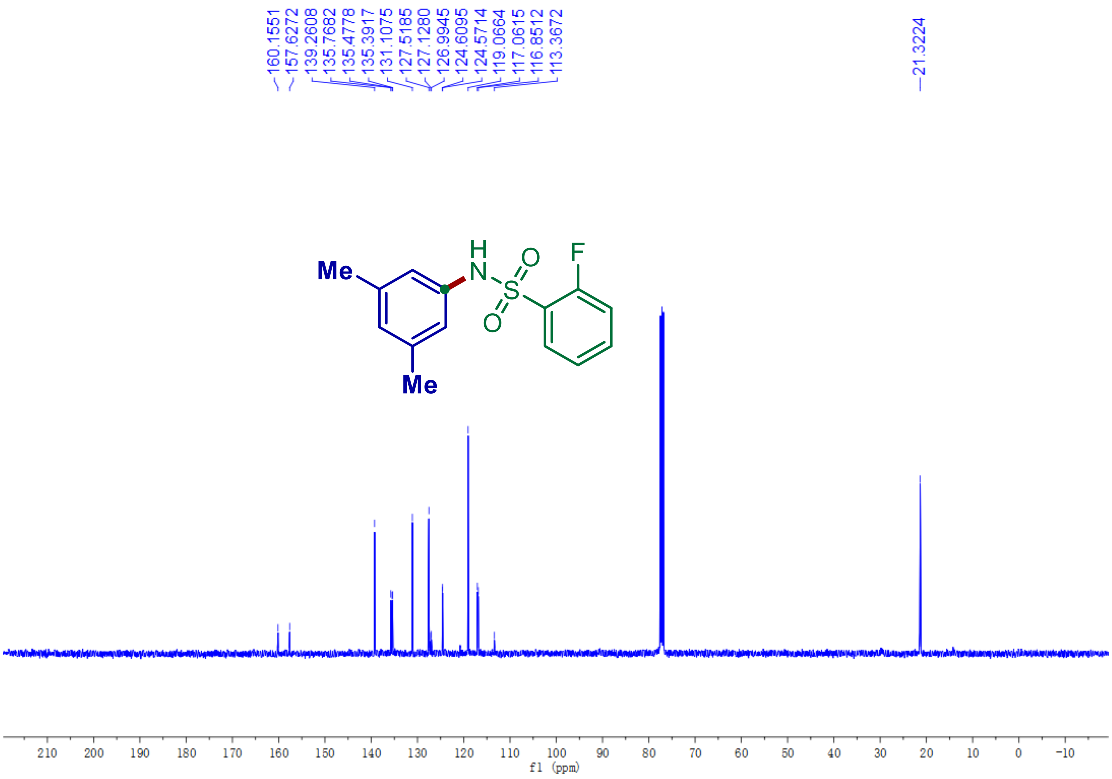

^{13}C NMR (100 MHz, $CDCl_3$) spectrum of compound 134

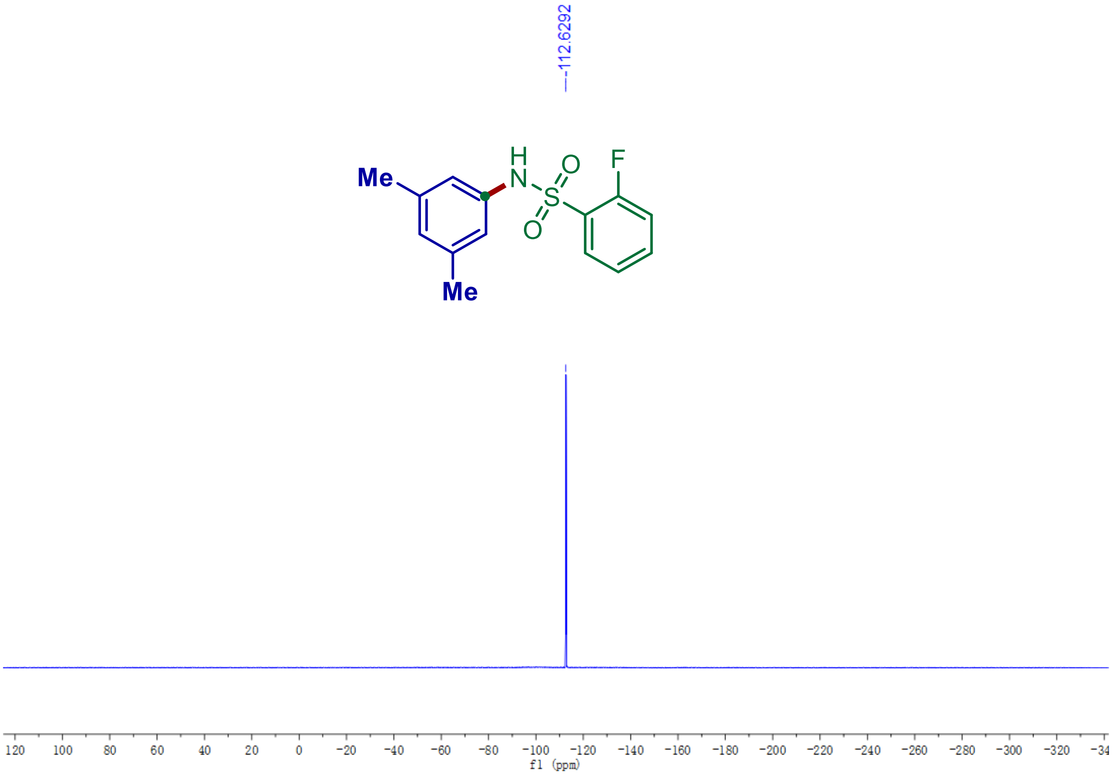

^{19}F NMR (376 MHz, $CDCl_3$) spectrum of compound 134

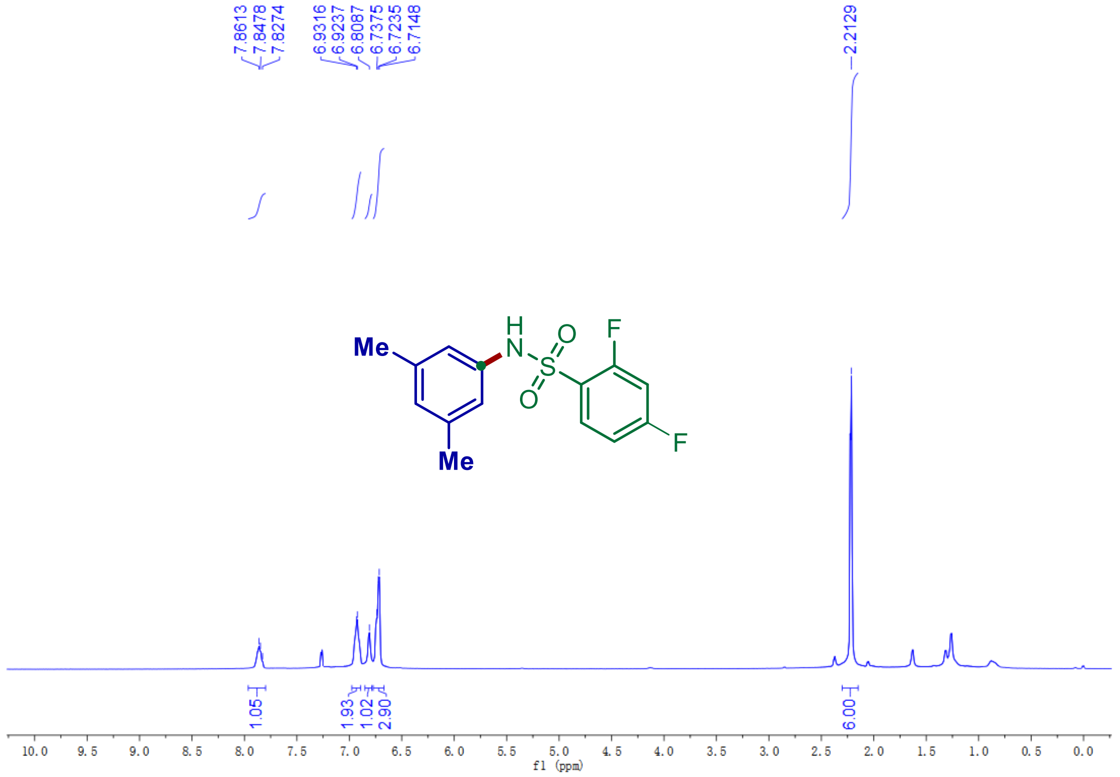

^{1}H NMR (400 MHz, $CDCl_3$) spectrum of compound 135

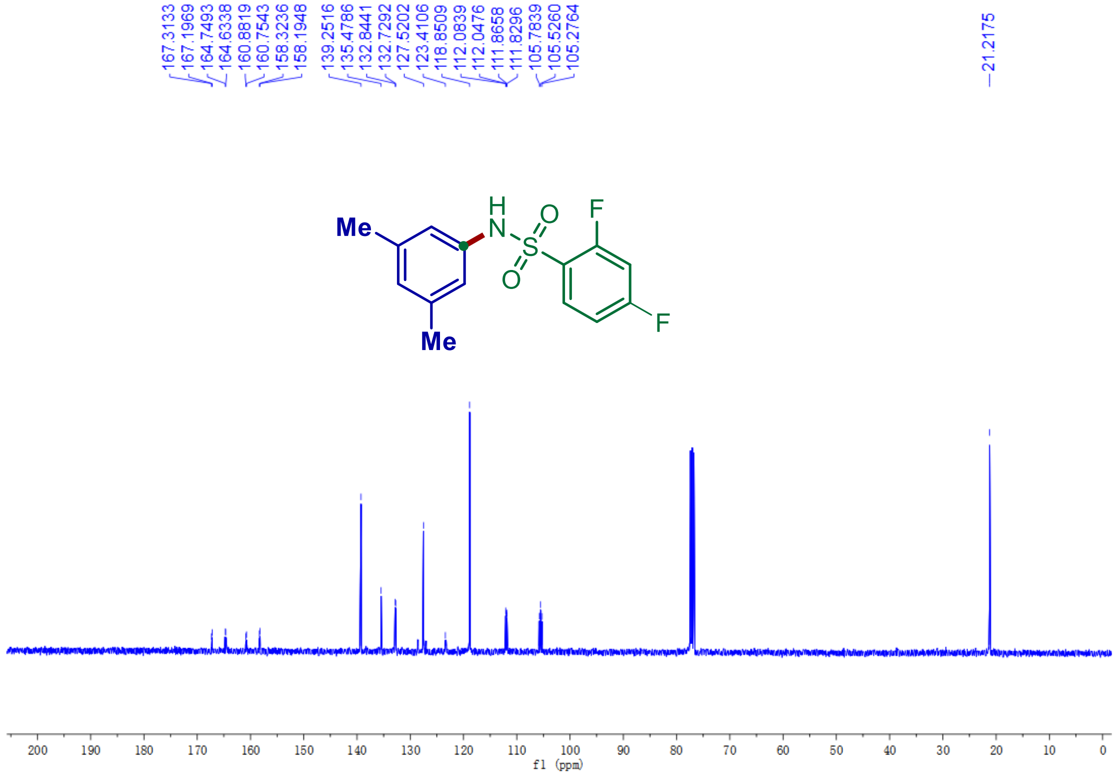

^{13}C NMR (100 MHz, $CDCl_3$) spectrum of compound 135

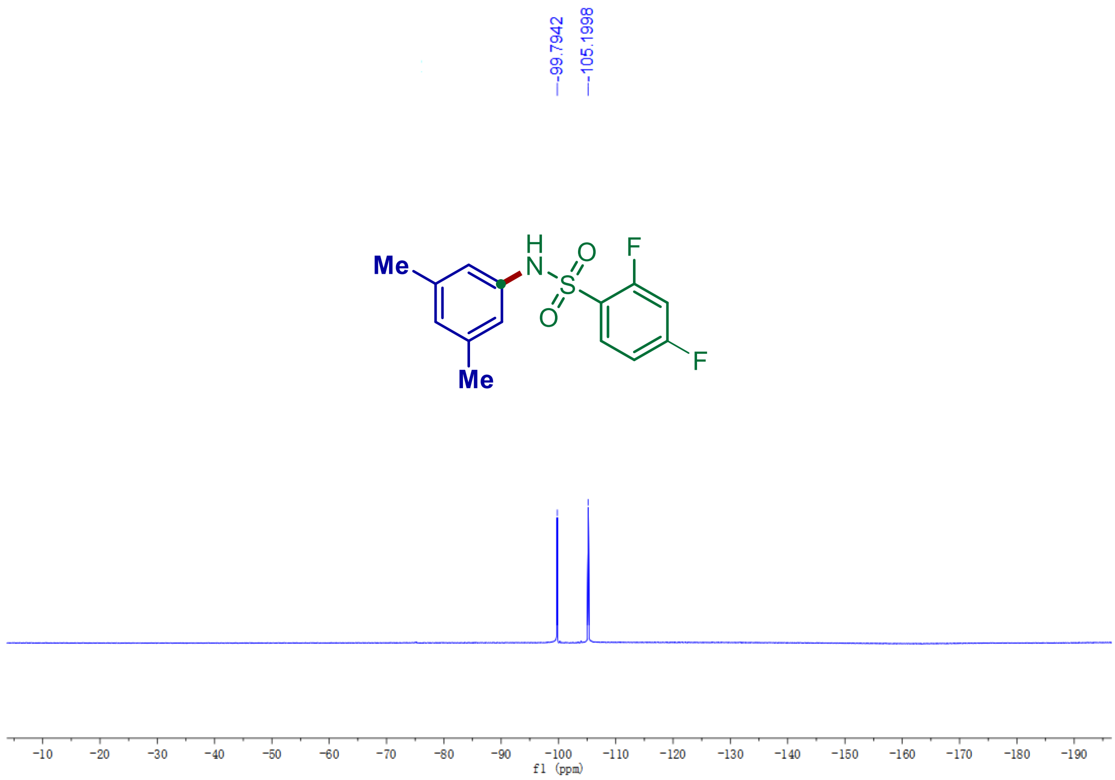

^{19}F NMR (376 MHz, $CDCl_3$) spectrum of compound 135

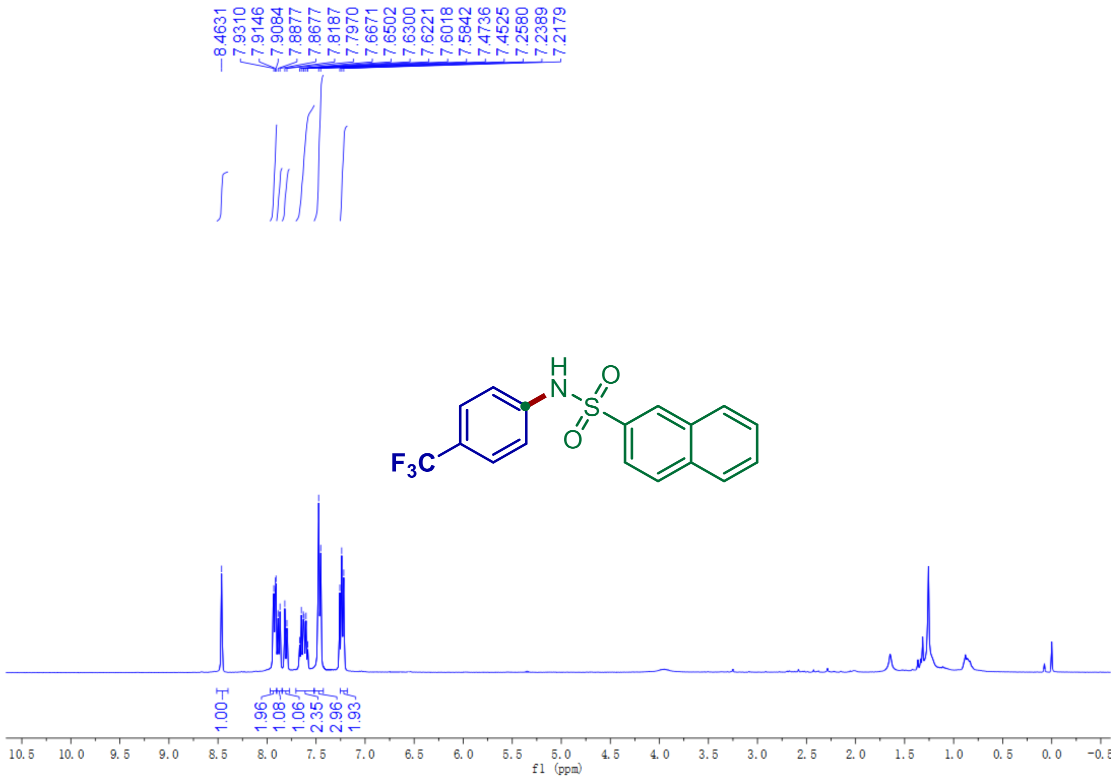

^{1}H NMR (400 MHz, $CDCl_3$) spectrum of compound 136

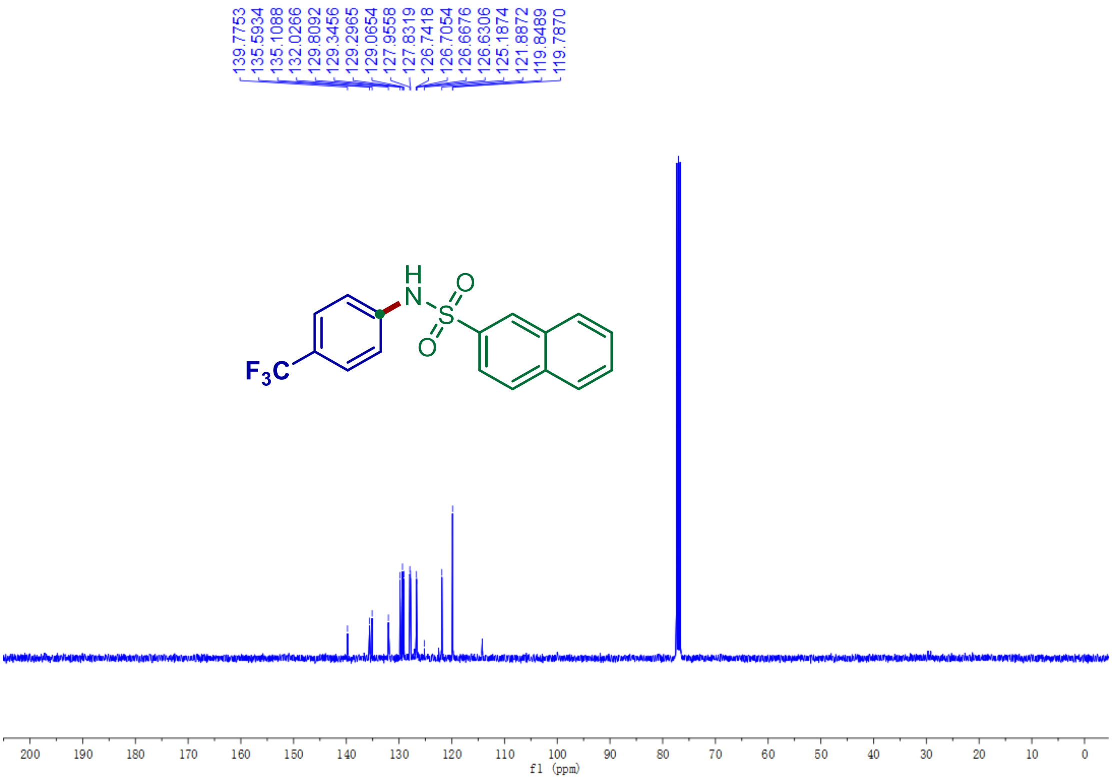

^{13}C NMR (100 MHz, $CDCl_3$) spectrum of compound 136

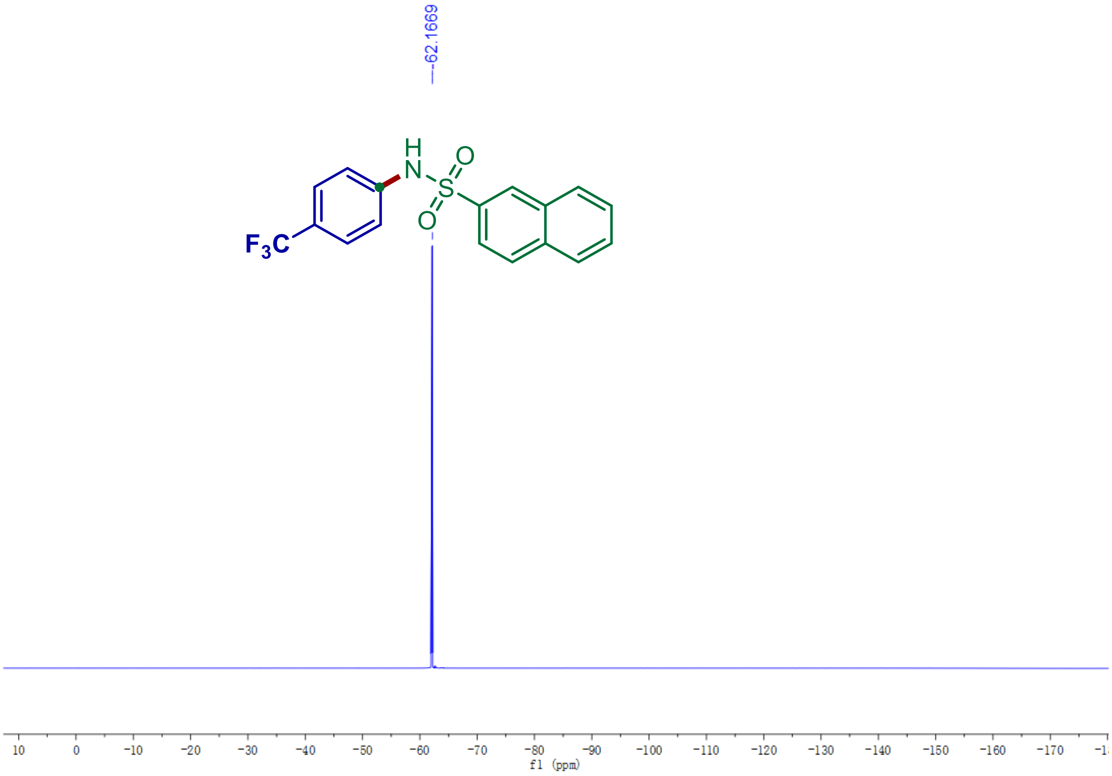

^{19}F NMR (376 MHz, $CDCl_3$) spectrum of compound 136

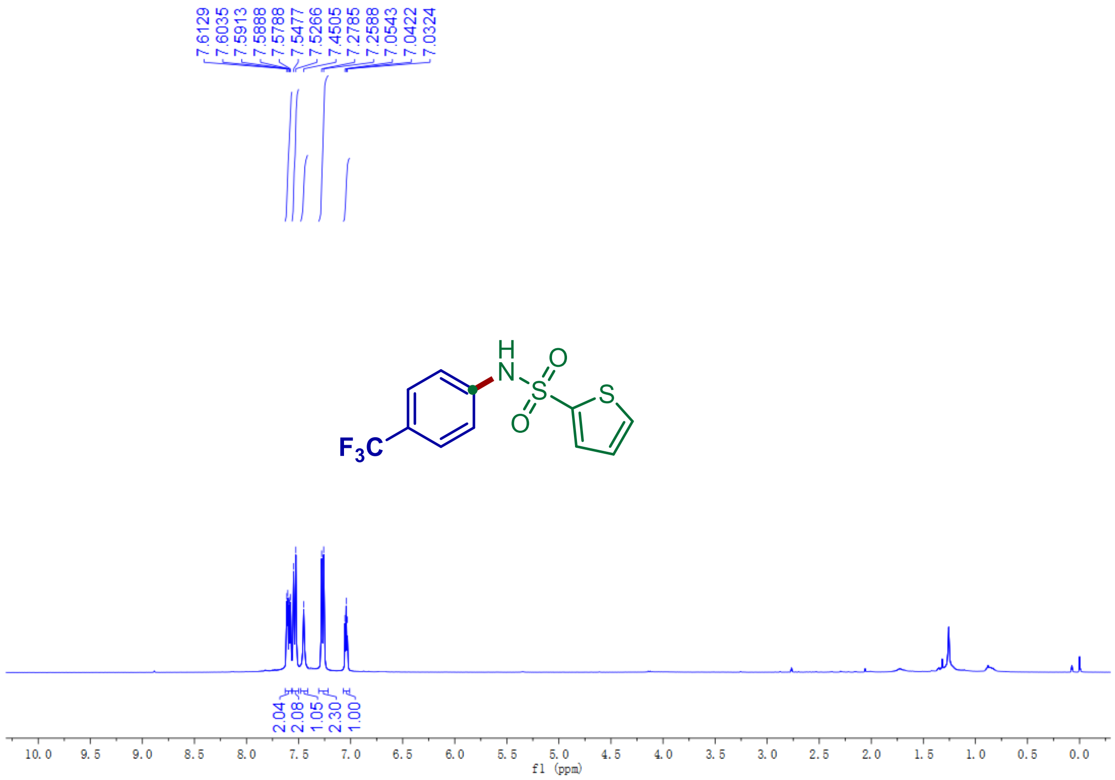

^{1}H NMR (400 MHz, $CDCl_3$) spectrum of compound 137

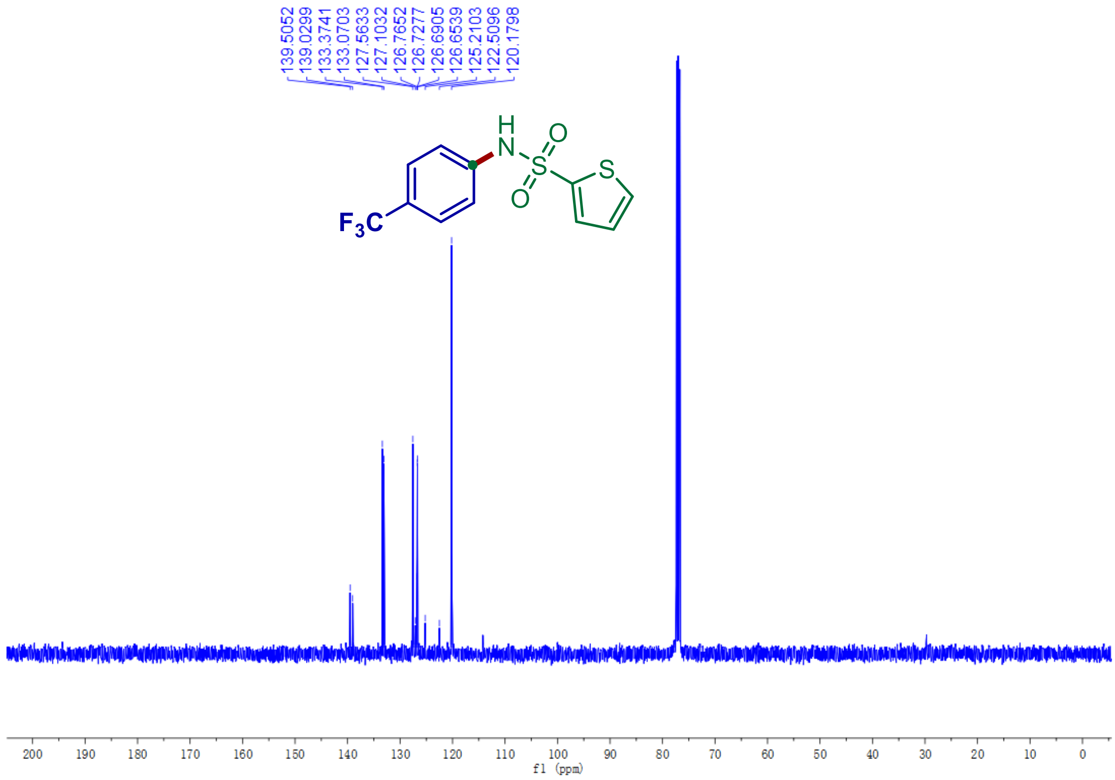

^{13}C NMR (100 MHz, $CDCl_3$) spectrum of compound 137

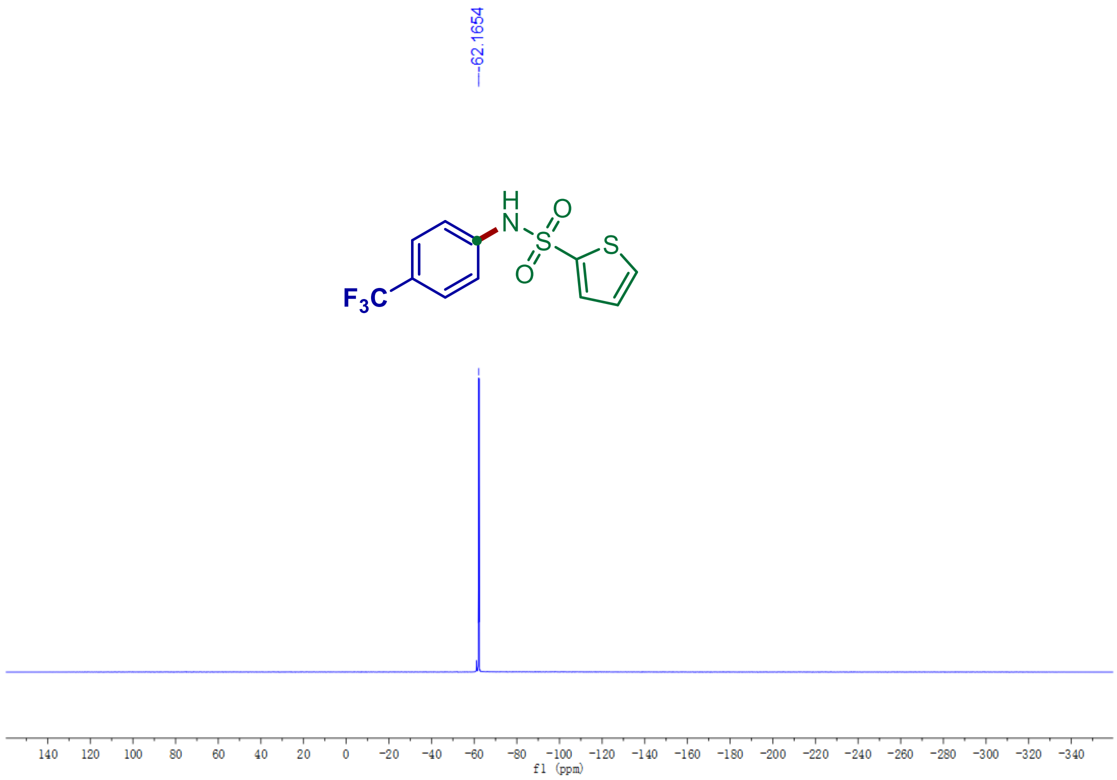

^{19}F NMR (376 MHz, CDCl$_3$) spectrum of compound 137

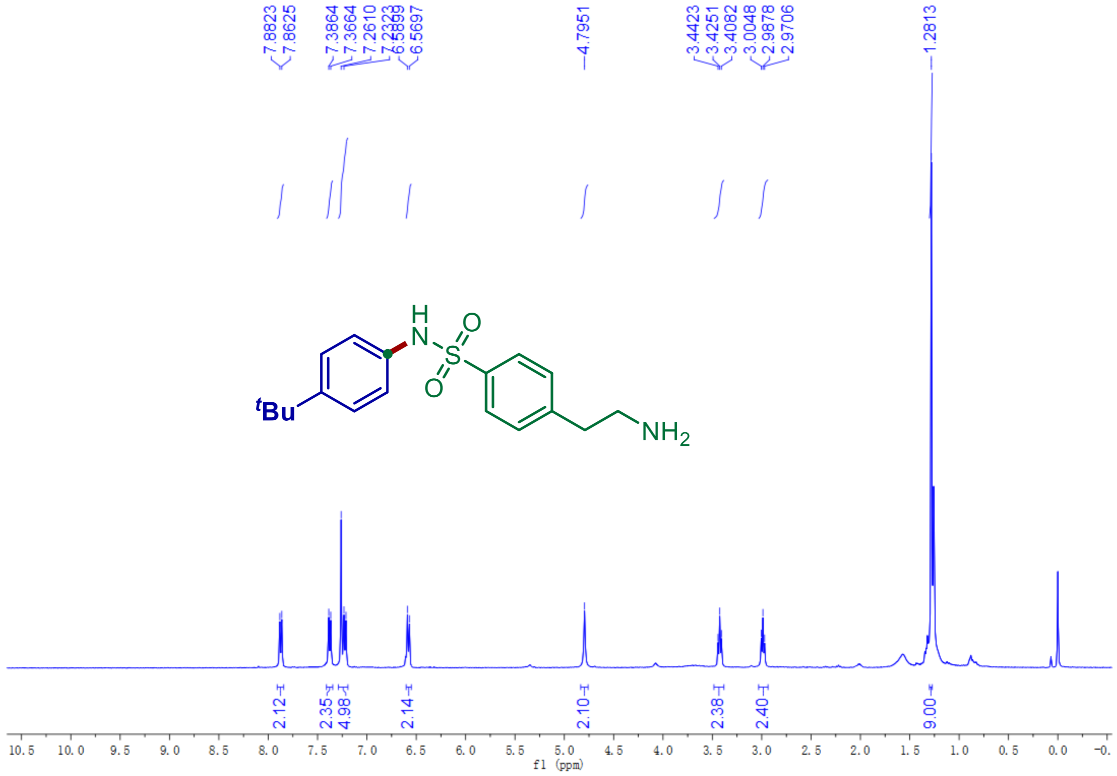

^{1}H NMR (400 MHz, CDCl$_3$) spectrum of compound 138

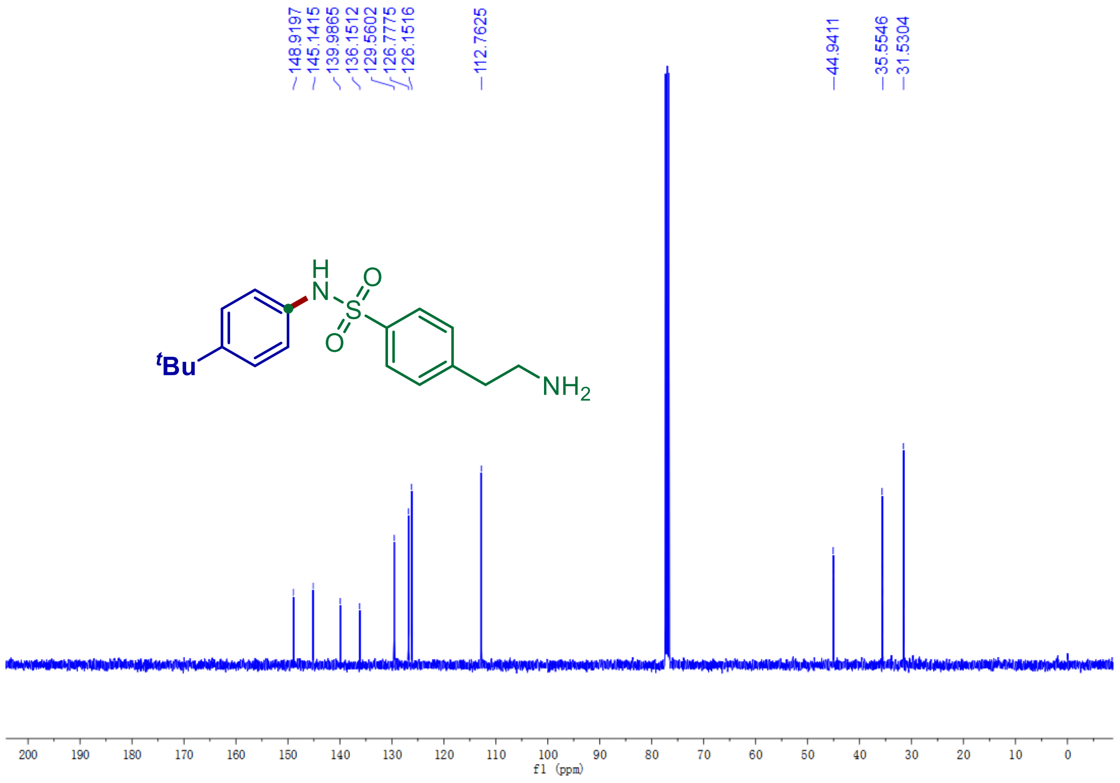

^{13}C NMR (100 MHz, $CDCl_3$) spectrum of compound 138

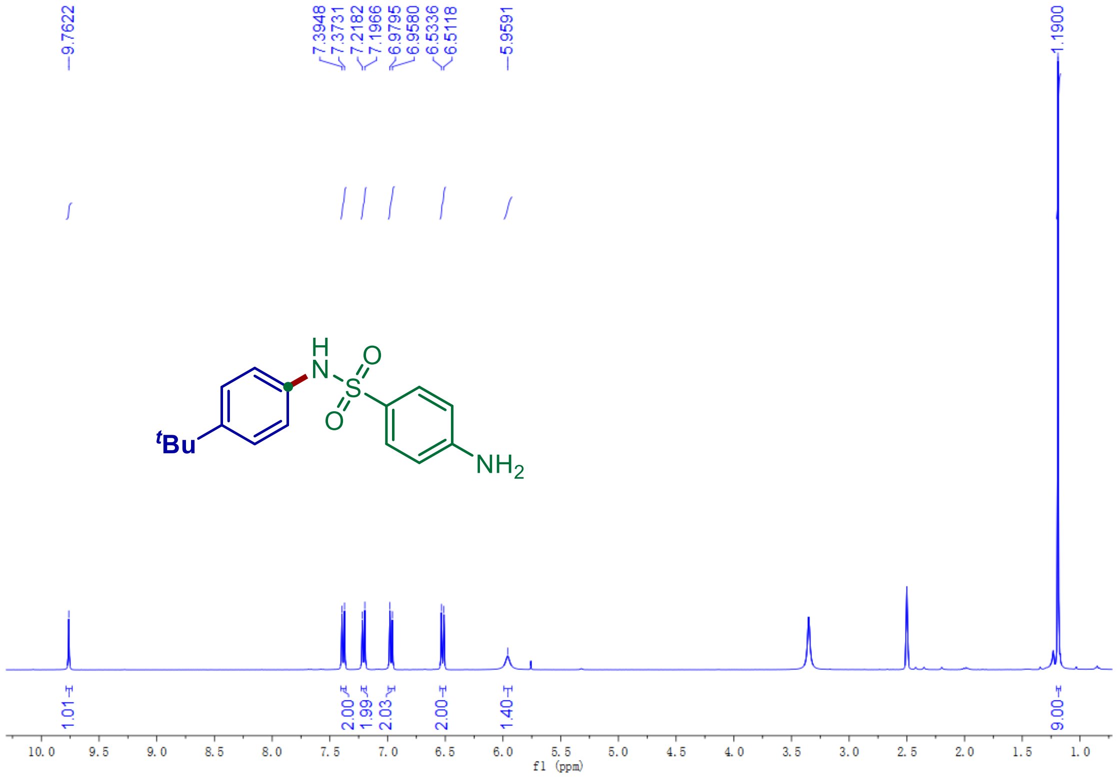

^{1}H NMR (400 MHz, d_6-DMSO) spectrum of compound 139

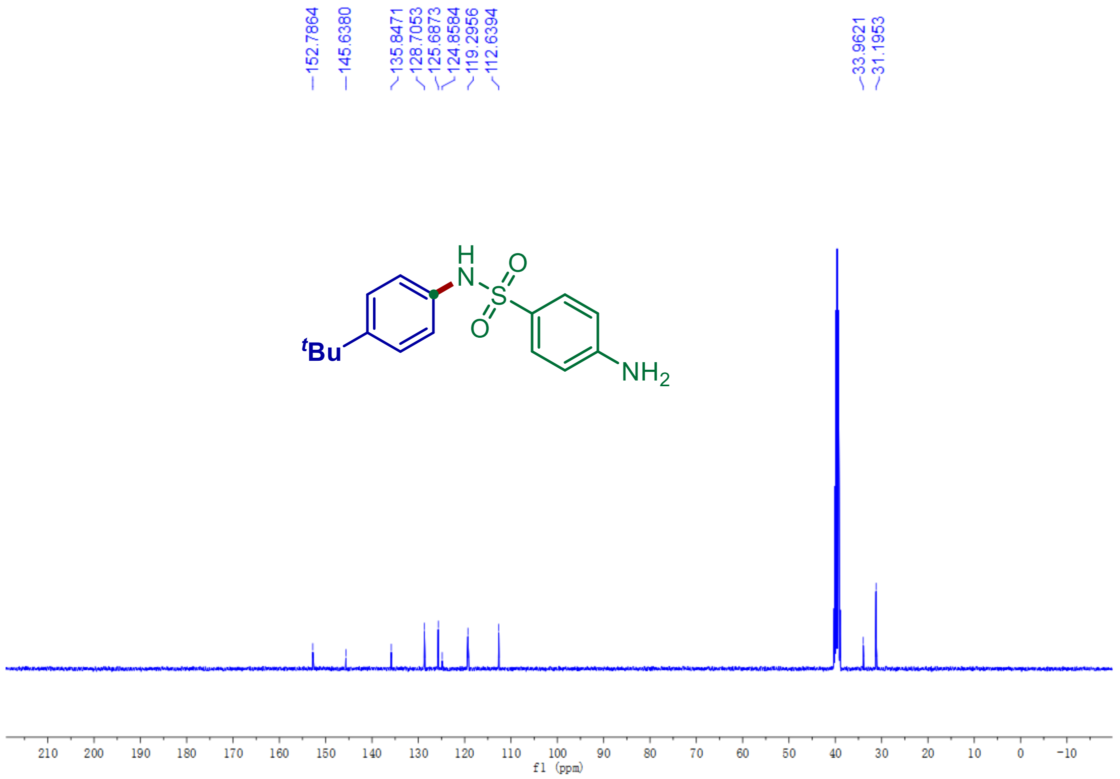

^{13}C NMR (100 MHz, d_6-DMSO) spectrum of compound 139

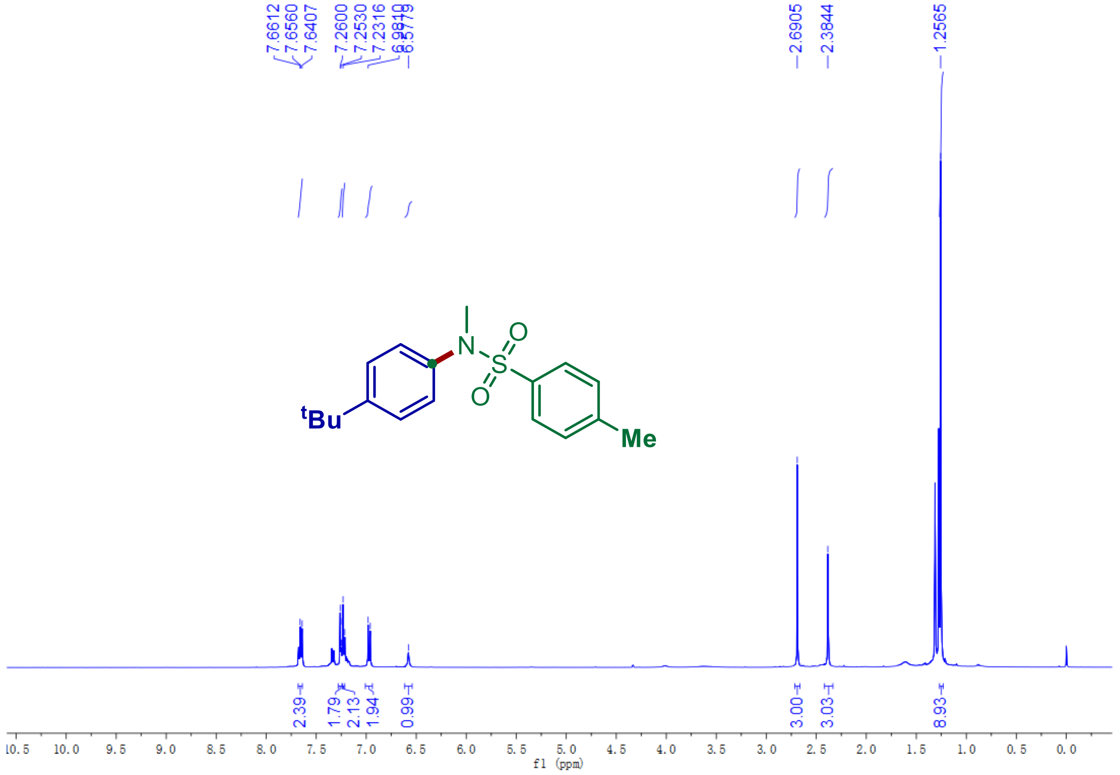

^{1}H NMR (400 MHz, $CDCl_3$) spectrum of compound 140

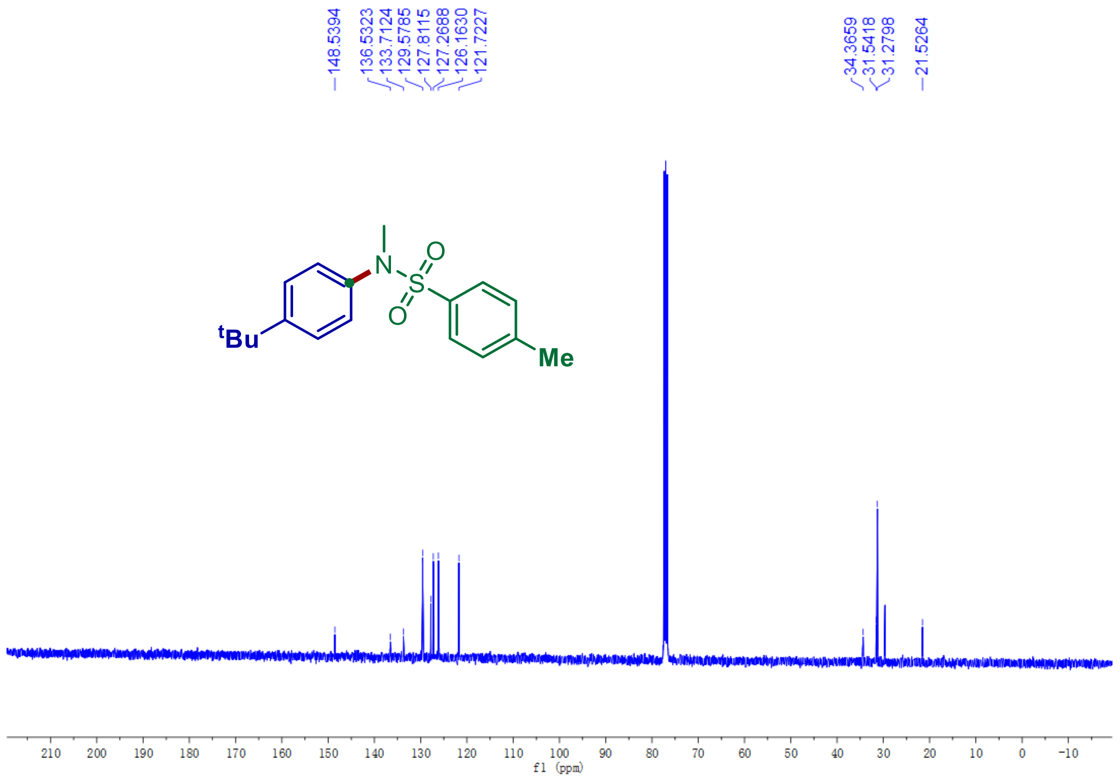

^{13}C NMR (100 MHz, $CDCl_3$) spectrum of compound 140

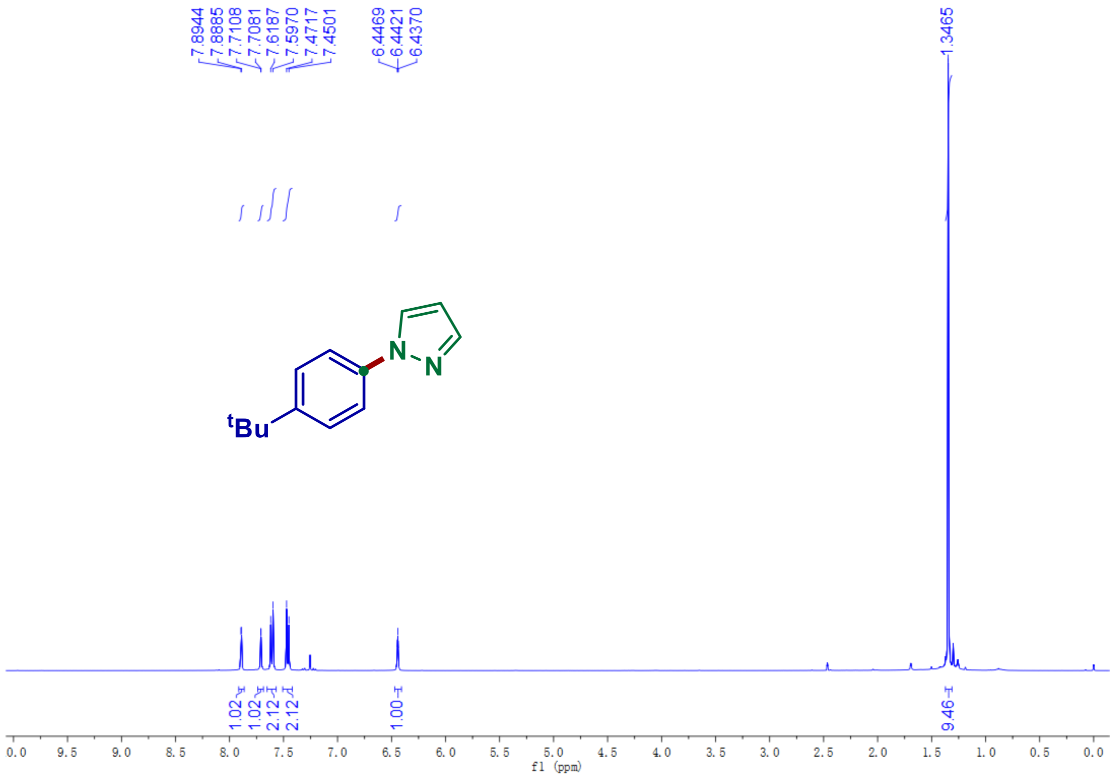

^{1}H NMR (400 MHz, $CDCl_3$) spectrum of compound 141

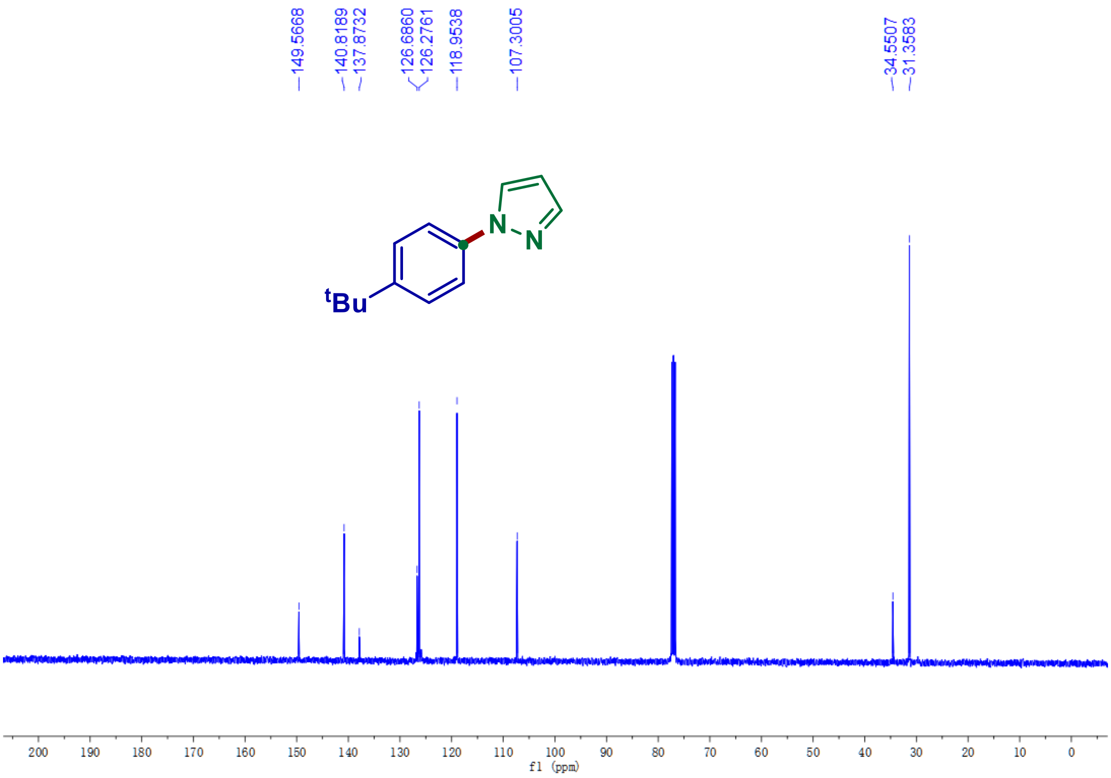

^{13}C NMR (100 MHz, $CDCl_3$) spectrum of compound 141

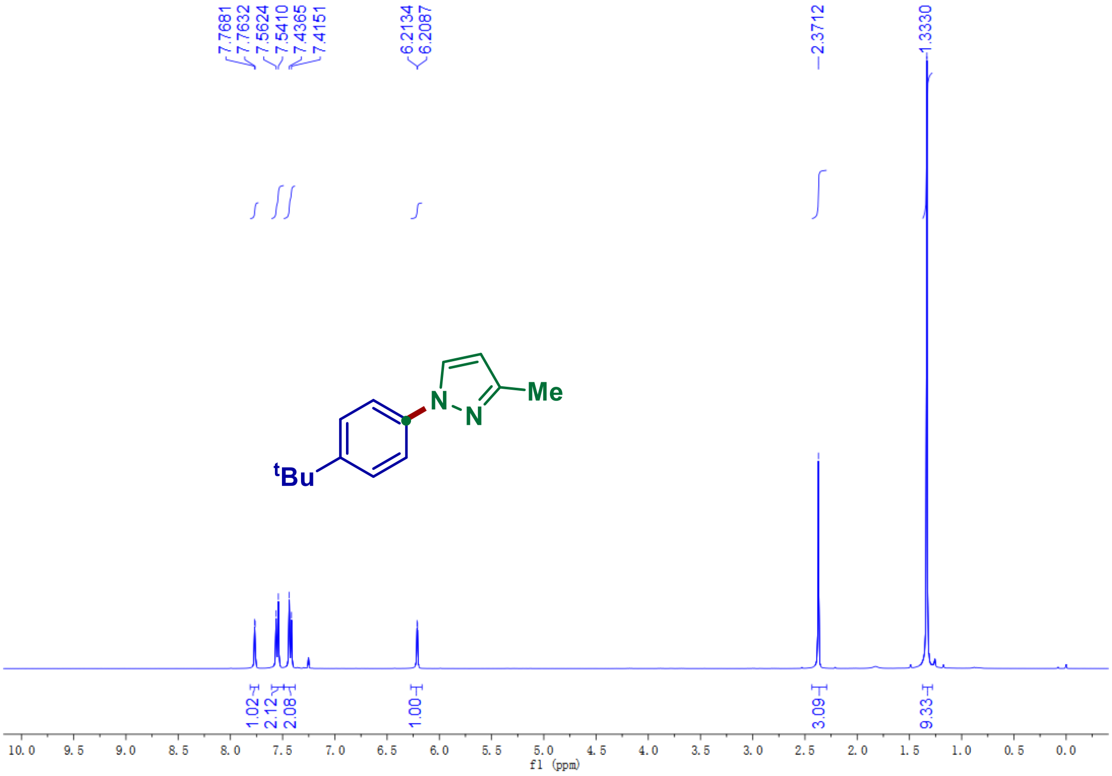

^{1}H NMR (400 MHz, $CDCl_3$) spectrum of compound 142

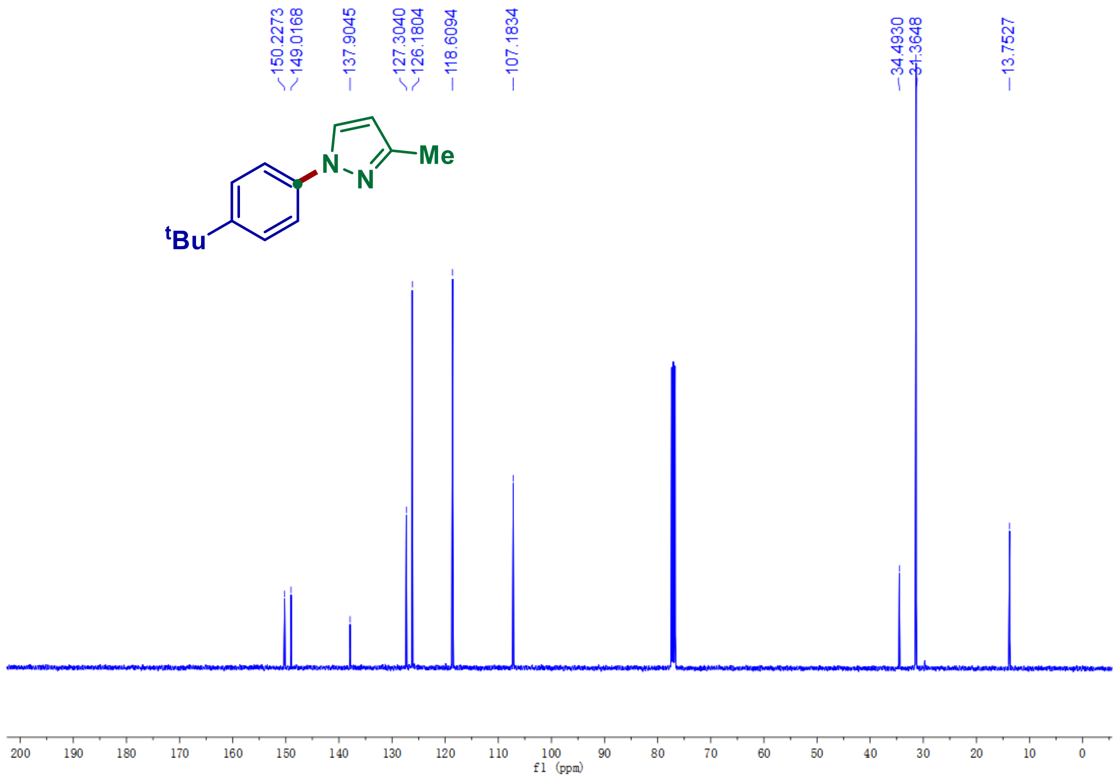

^{13}C NMR (100 MHz, $CDCl_3$) spectrum of compound 142

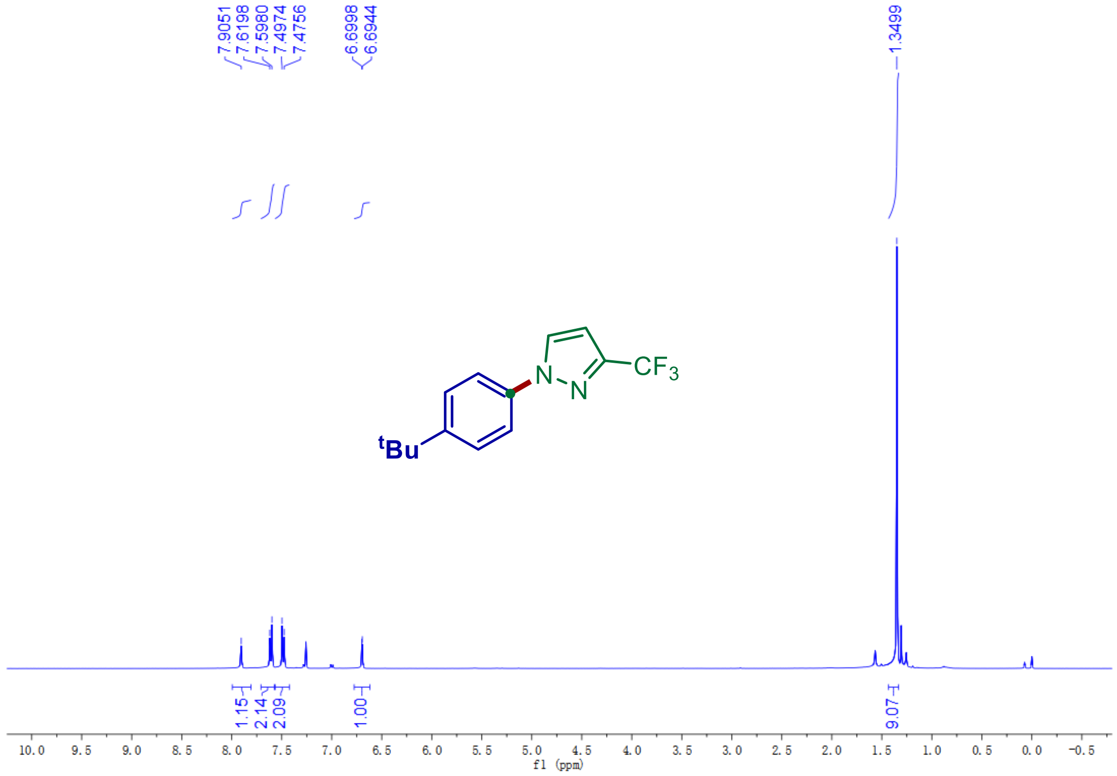

^{1}H NMR (400 MHz, $CDCl_3$) spectrum of compound 143

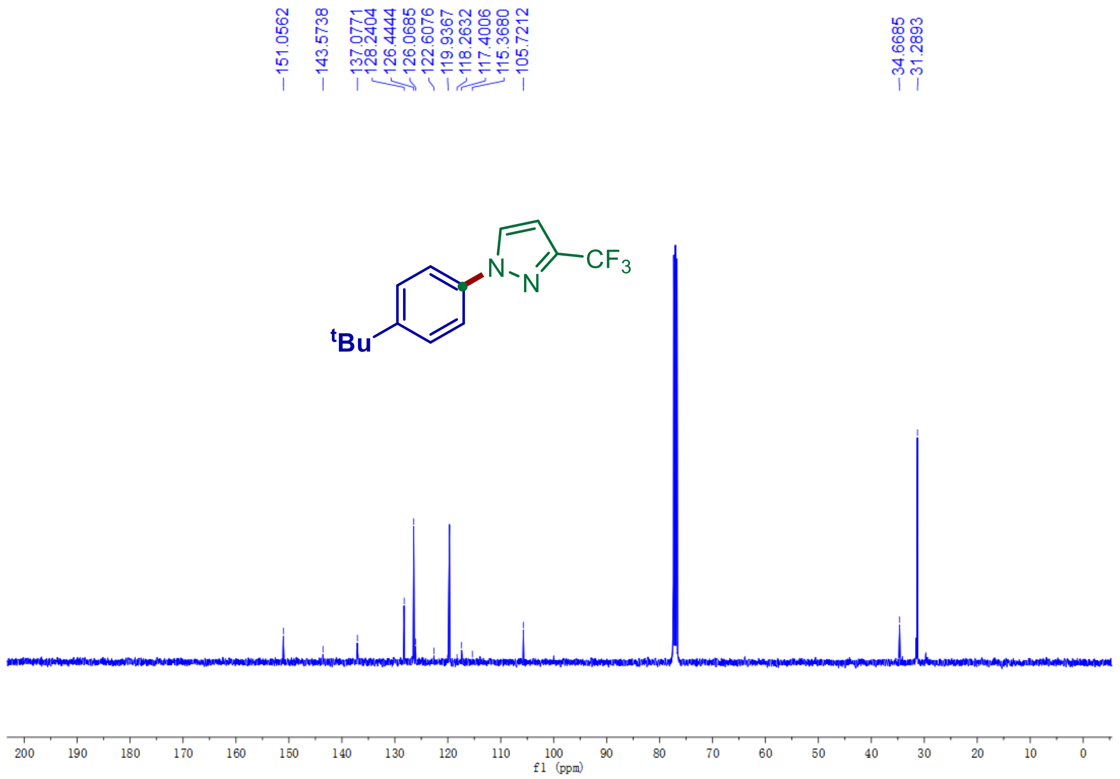

^{13}C NMR (100 MHz, $CDCl_3$) spectrum of compound 143

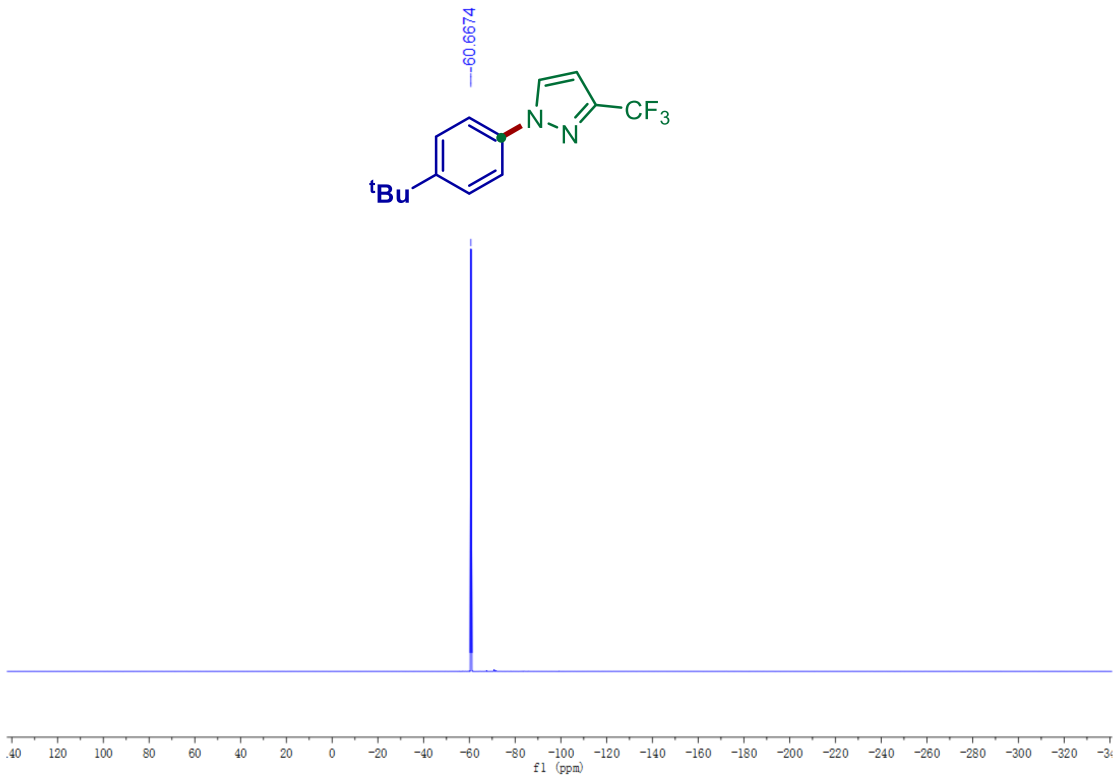

^{19}F NMR (376 MHz, $CDCl_3$) spectrum of compound 143

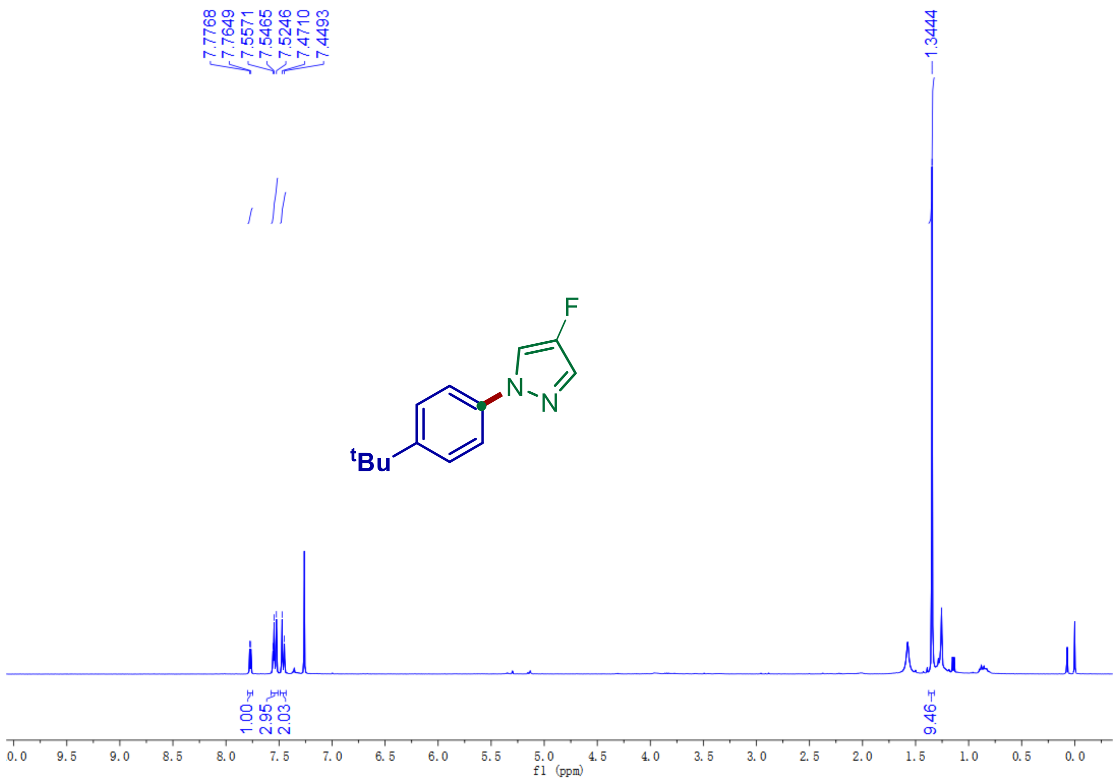

^{1}H NMR (400 MHz, $CDCl_3$) spectrum of compound 144

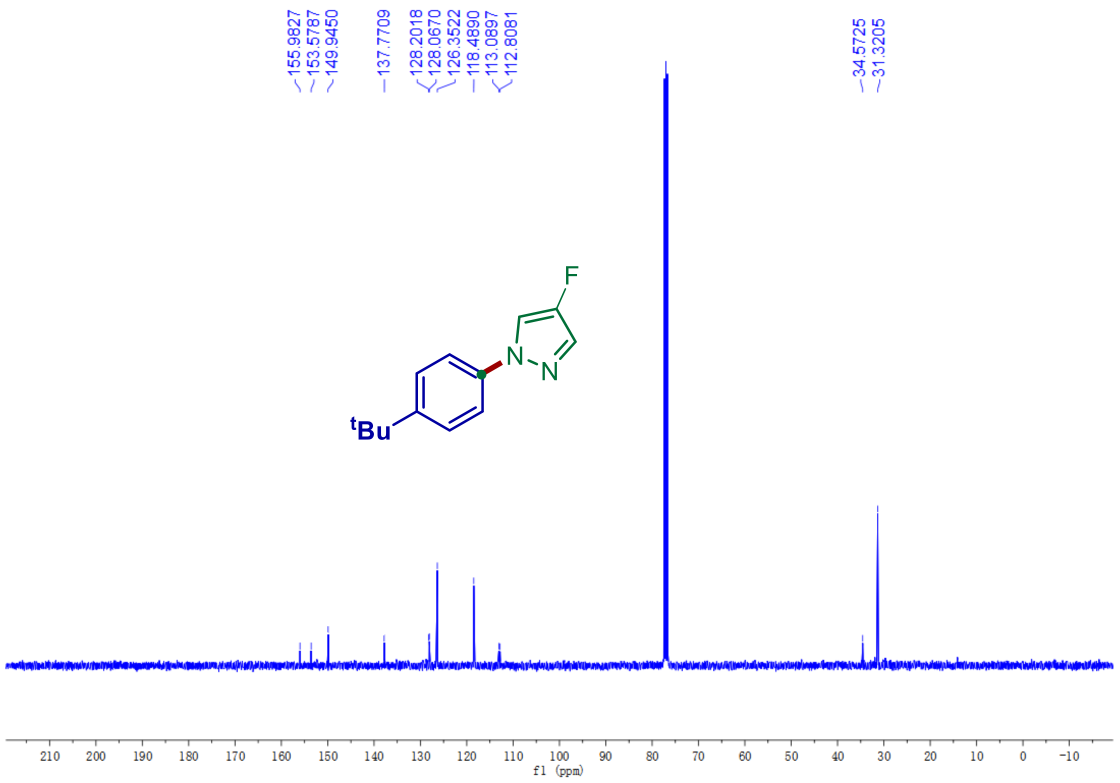

^{13}C NMR (100 MHz, $CDCl_3$) spectrum of compound 144

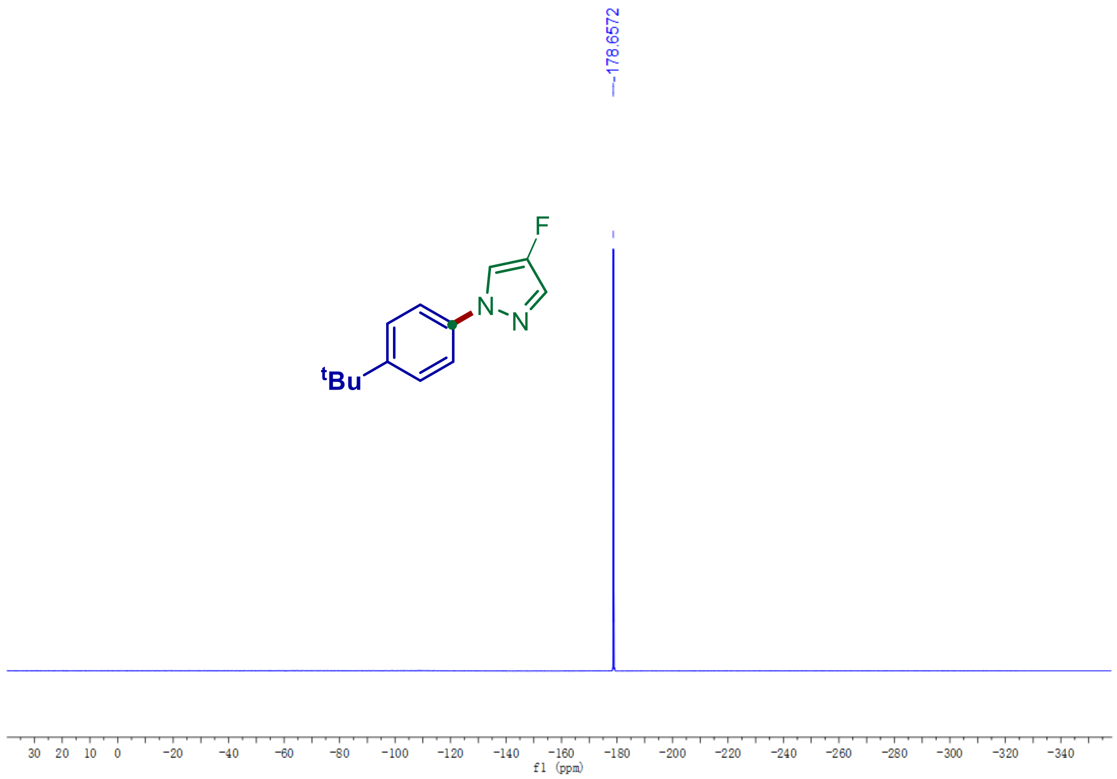

^{19}F NMR (376 MHz, CDCl$_3$) spectrum of compound 144

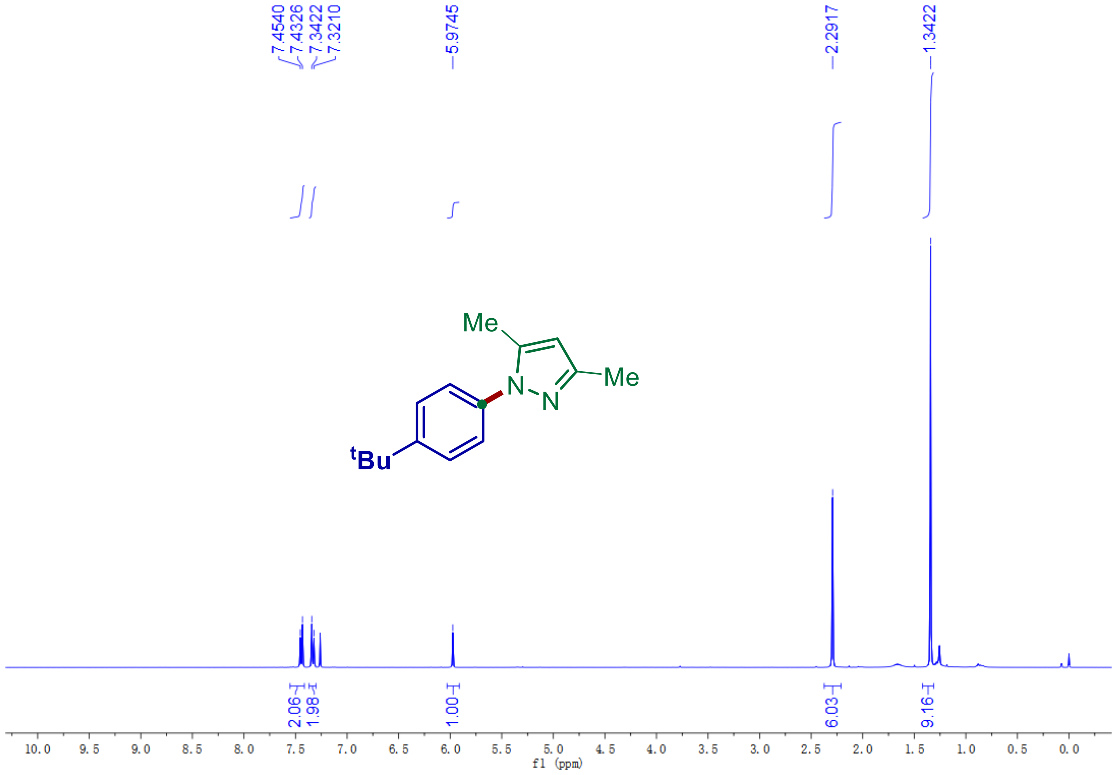

^{1}H NMR (400 MHz, CDCl$_3$) spectrum of compound 145

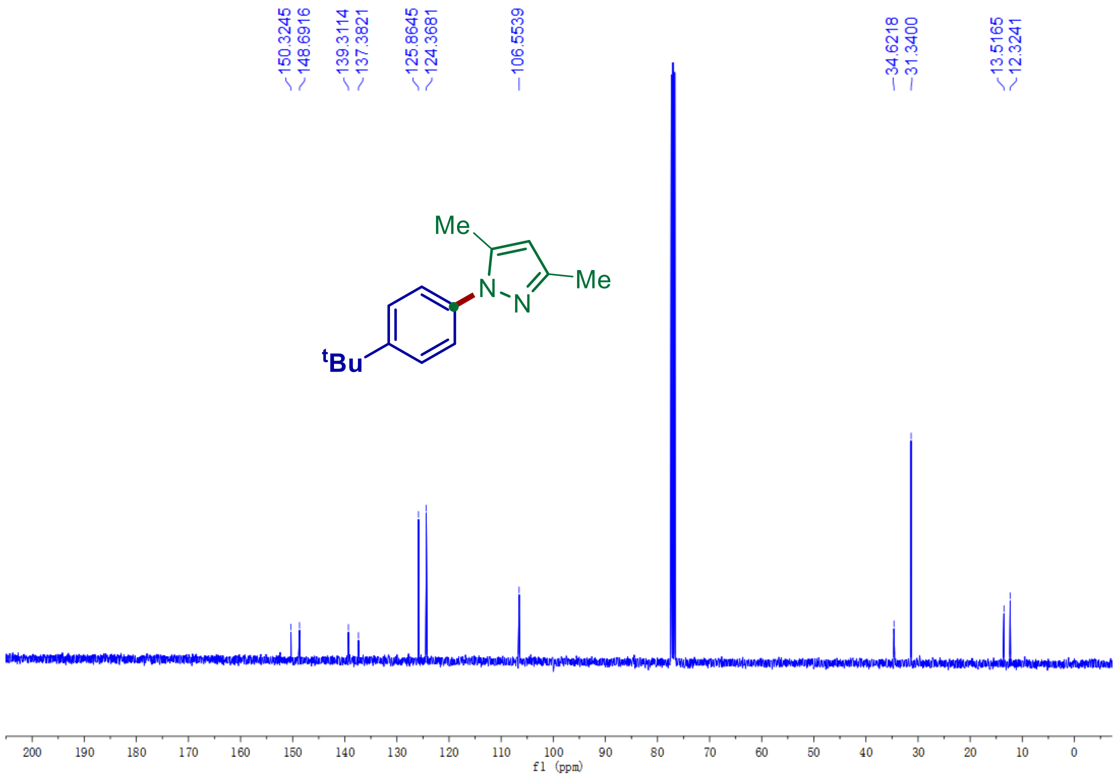

^{13}C NMR (100 MHz, $CDCl_3$) spectrum of compound 145

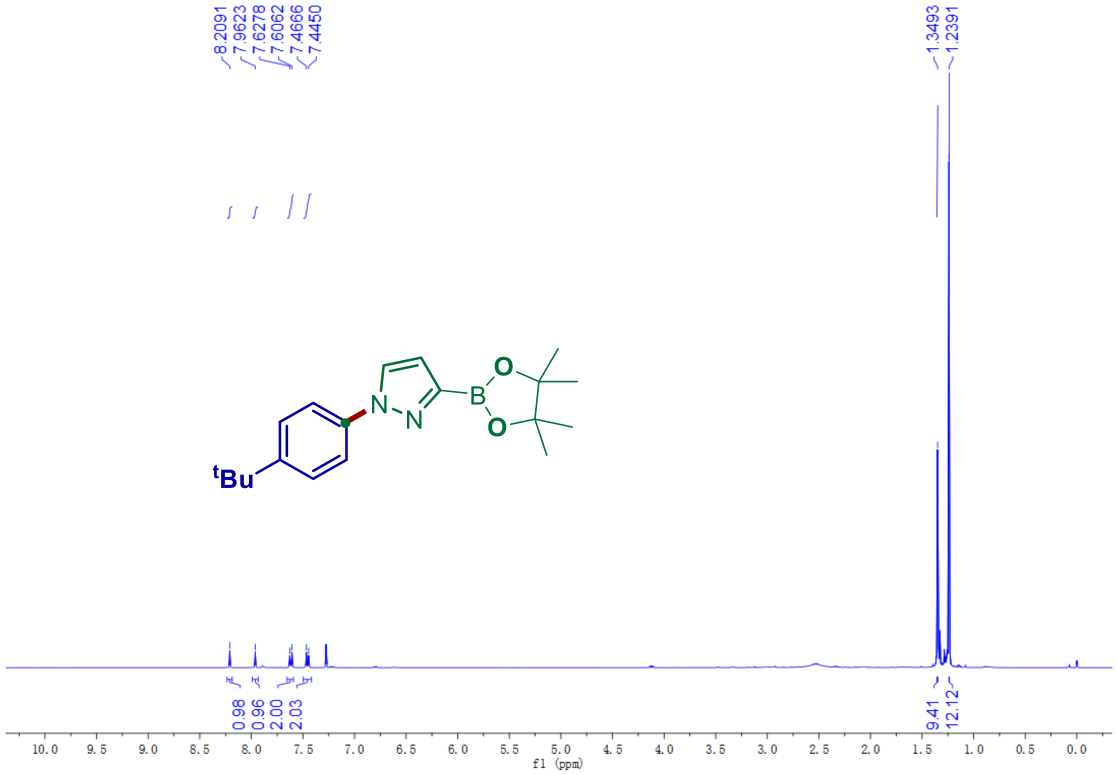

^{1}H NMR (400 MHz, $CDCl_3$) spectrum of compound 146

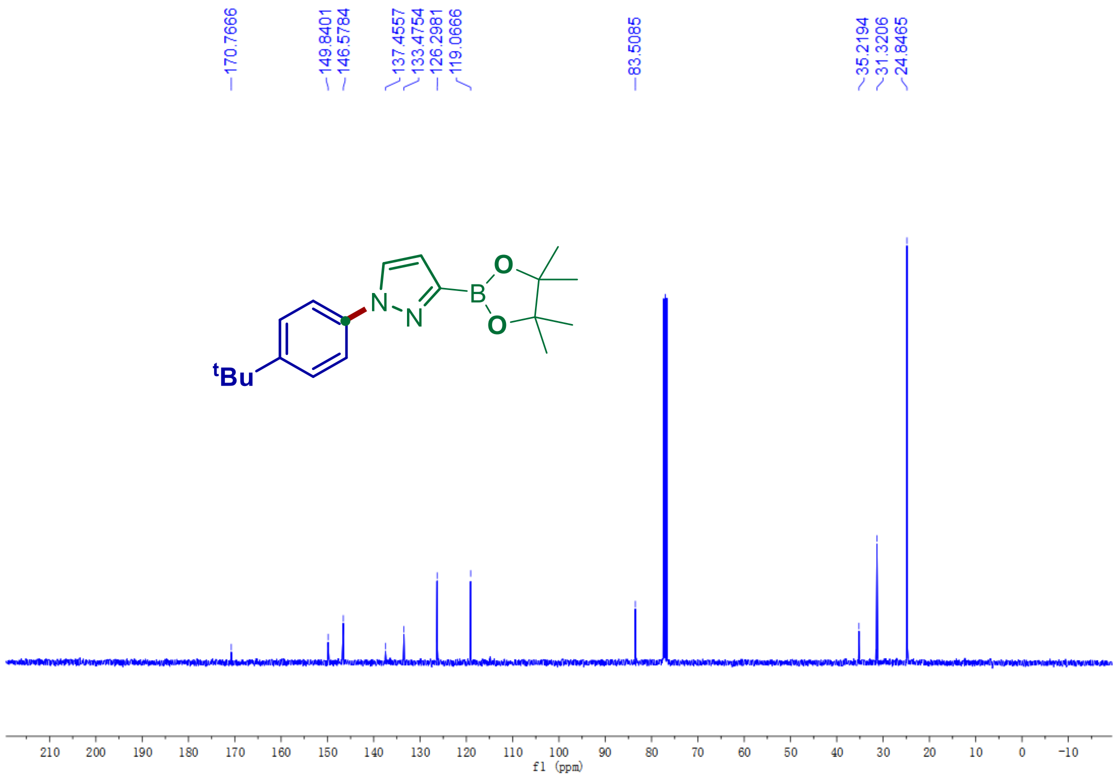

^{13}C NMR (100 MHz, $CDCl_3$) spectrum of compound 146

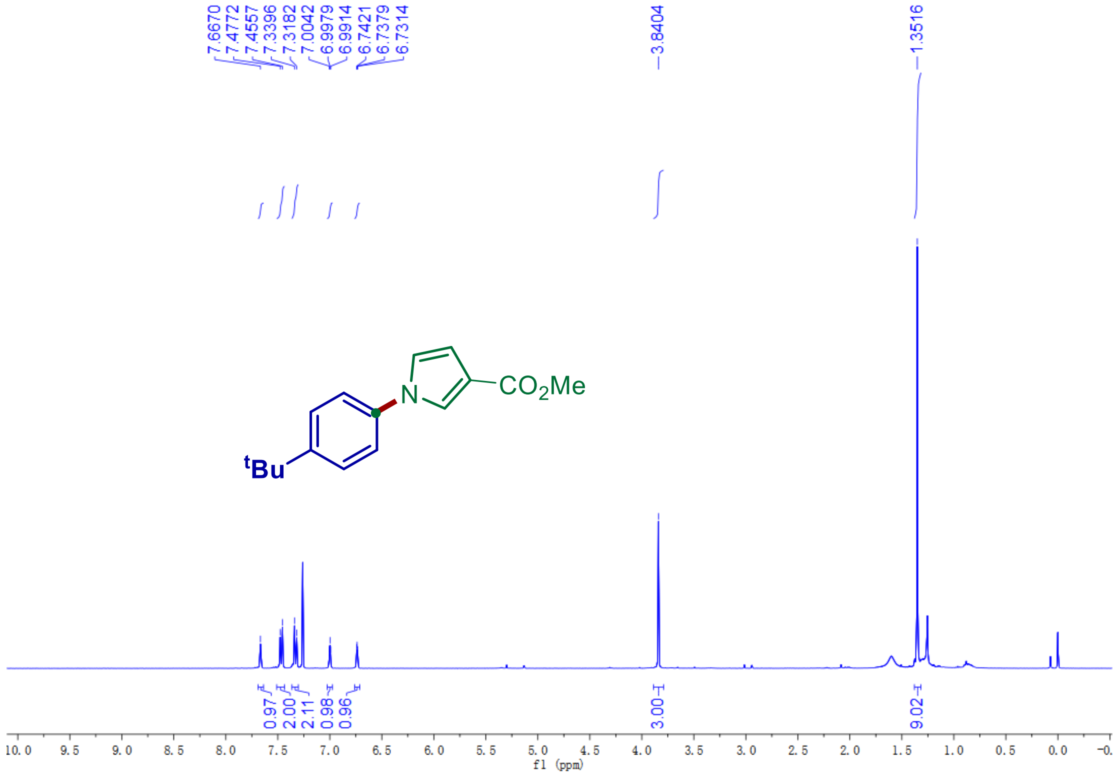

^{1}H NMR (400 MHz, $CDCl_3$) spectrum of compound 147

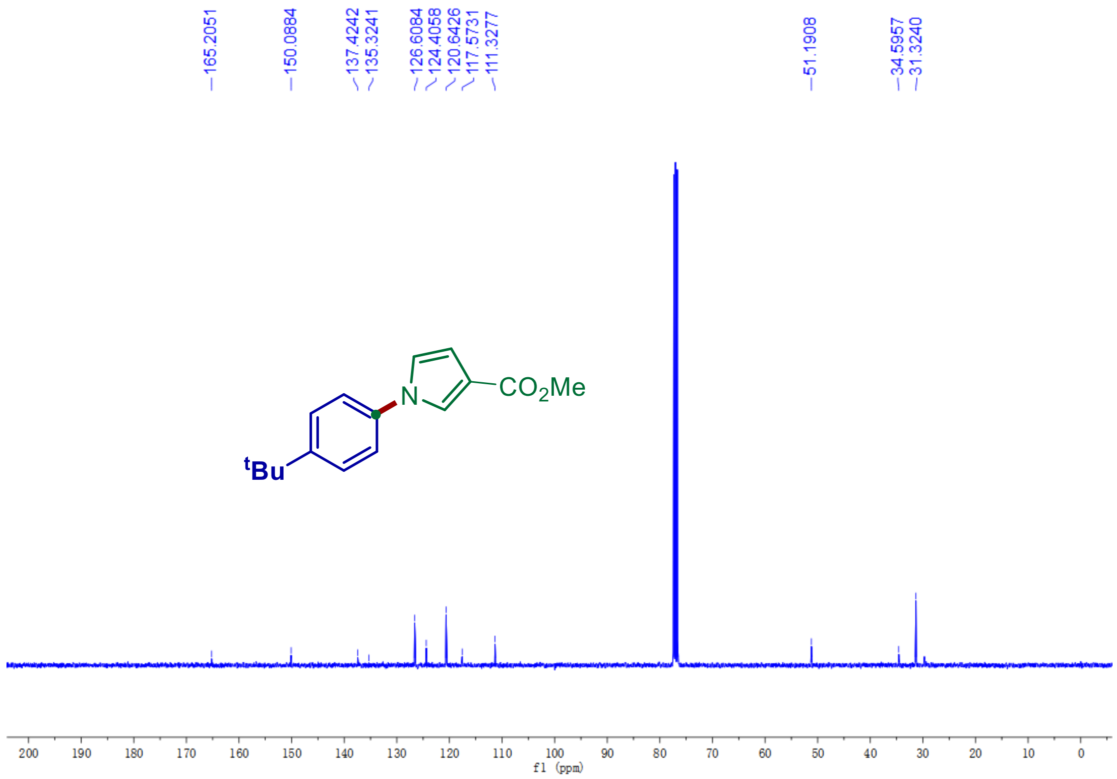

^{13}C NMR (100 MHz, $CDCl_3$) spectrum of compound 147

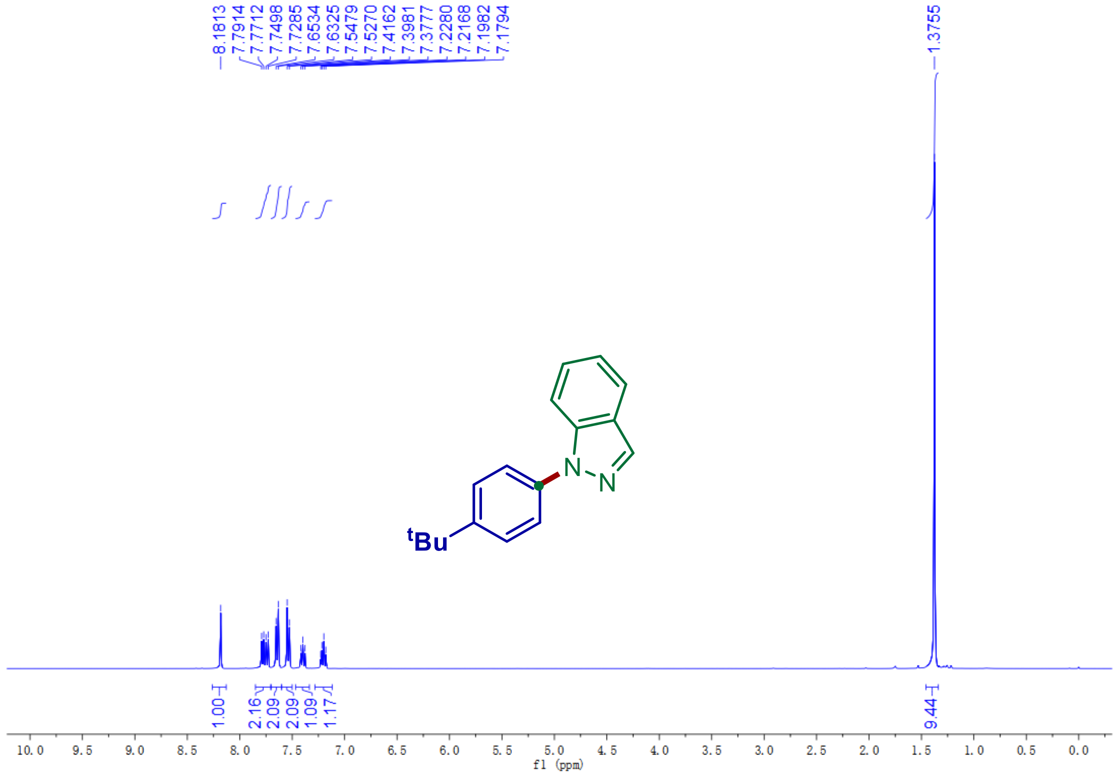

^{1}H NMR (400 MHz, $CDCl_3$) spectrum of compound 148

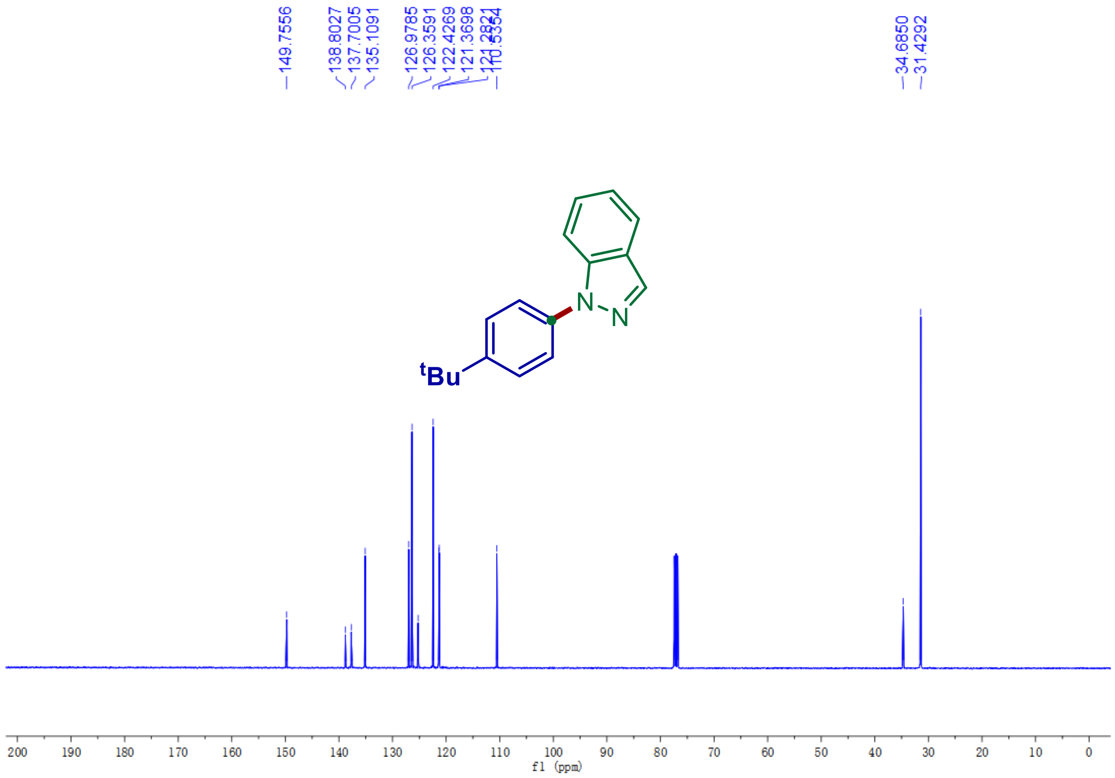

^{13}C NMR (100 MHz, $CDCl_3$) spectrum of compound 148

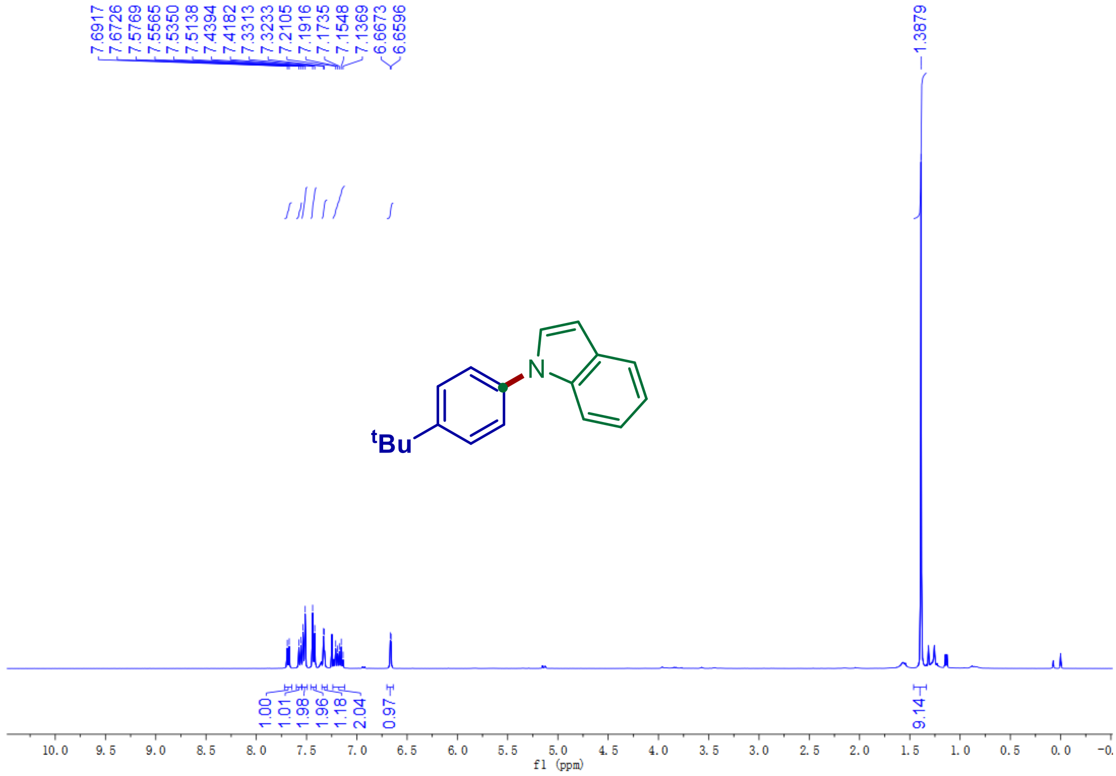

^{1}H NMR (400 MHz, $CDCl_3$) spectrum of compound 149

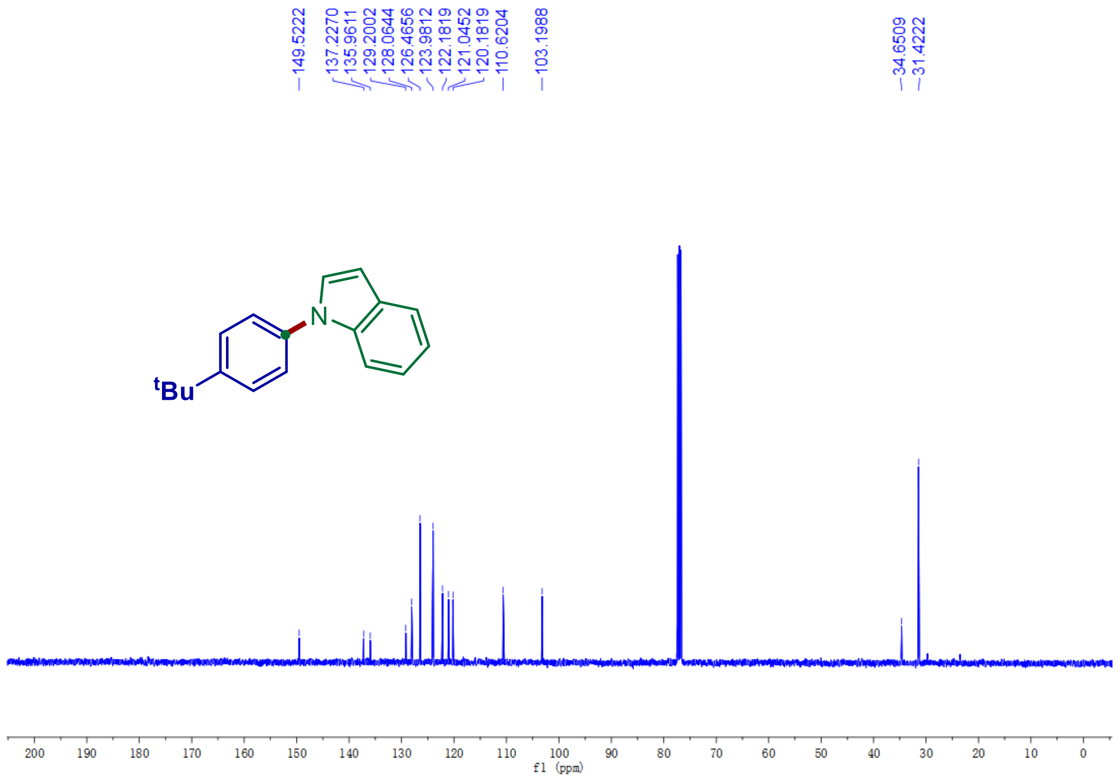

^{13}C NMR (100 MHz, $CDCl_3$) spectrum of compound 149

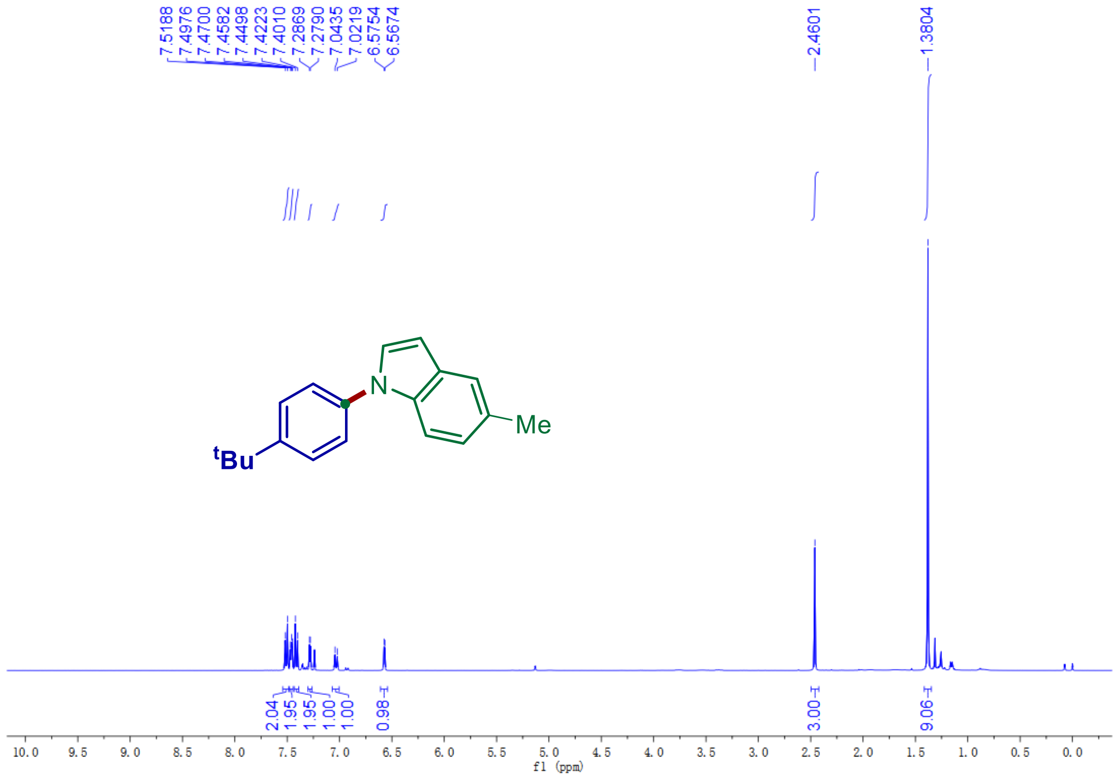

^{1}H NMR (400 MHz, $CDCl_3$) spectrum of compound 150

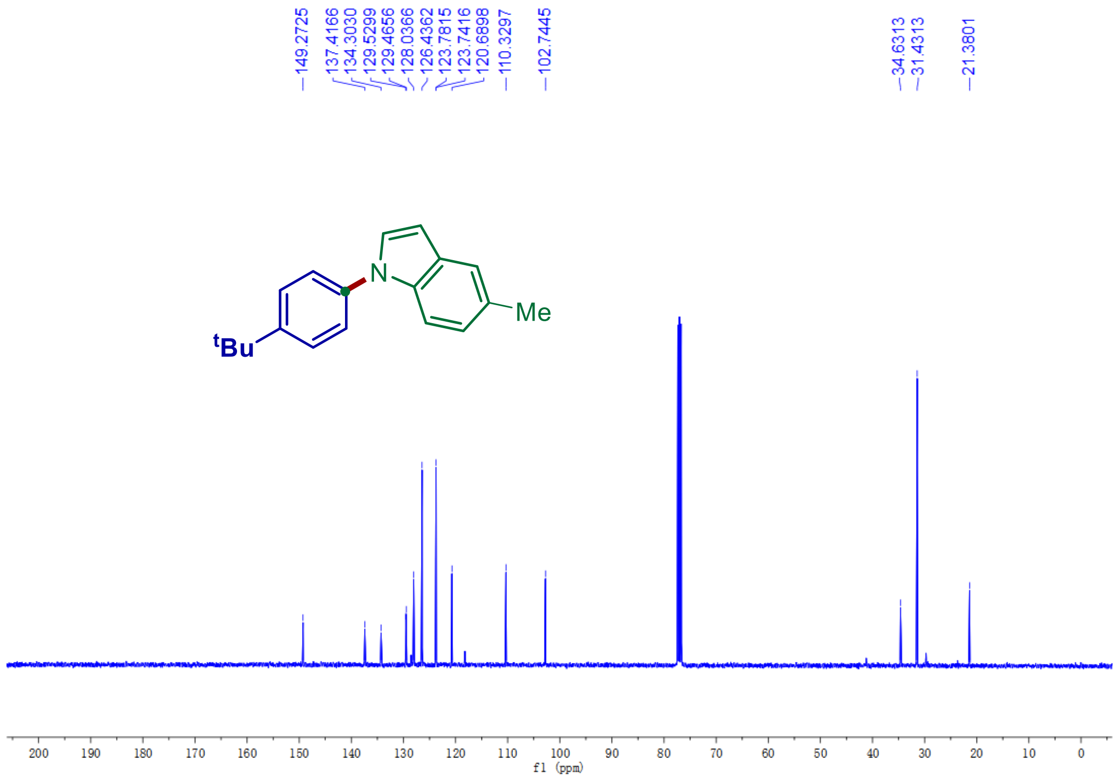

^{13}C NMR (100 MHz, $CDCl_3$) spectrum of compound 150

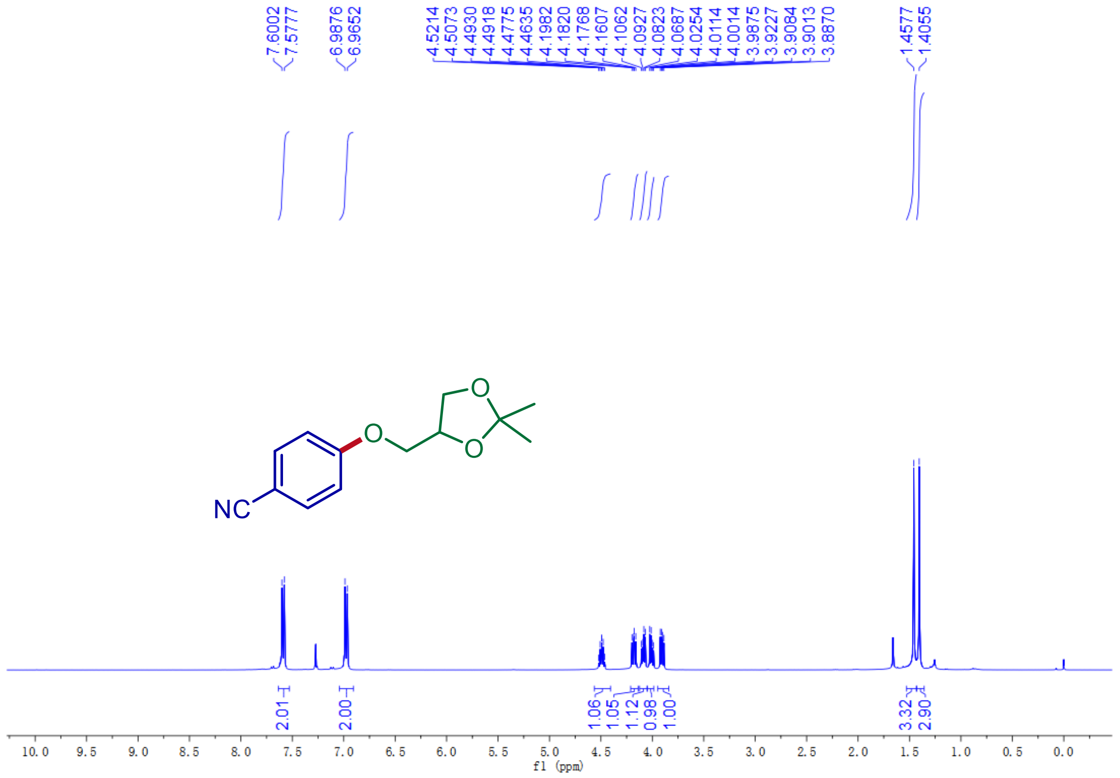

^{1}H NMR (400 MHz, $CDCl_3$) spectrum of compound 151

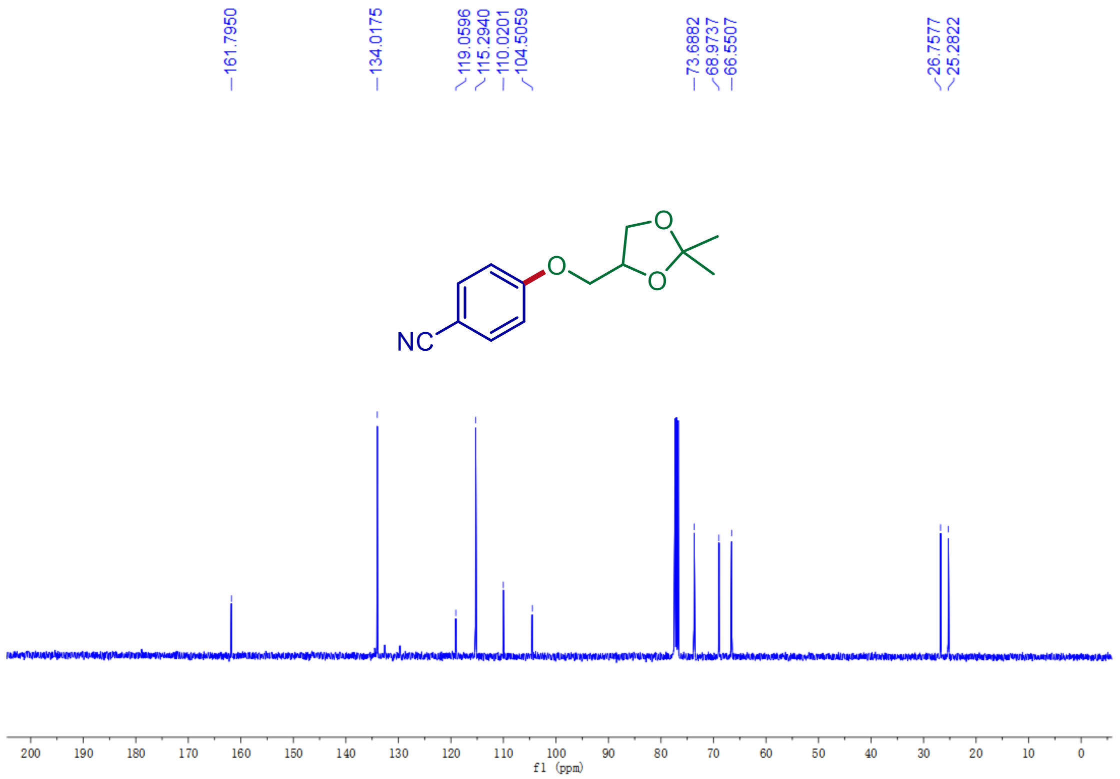

^{13}C NMR (100 MHz, $CDCl_3$) spectrum of compound 151

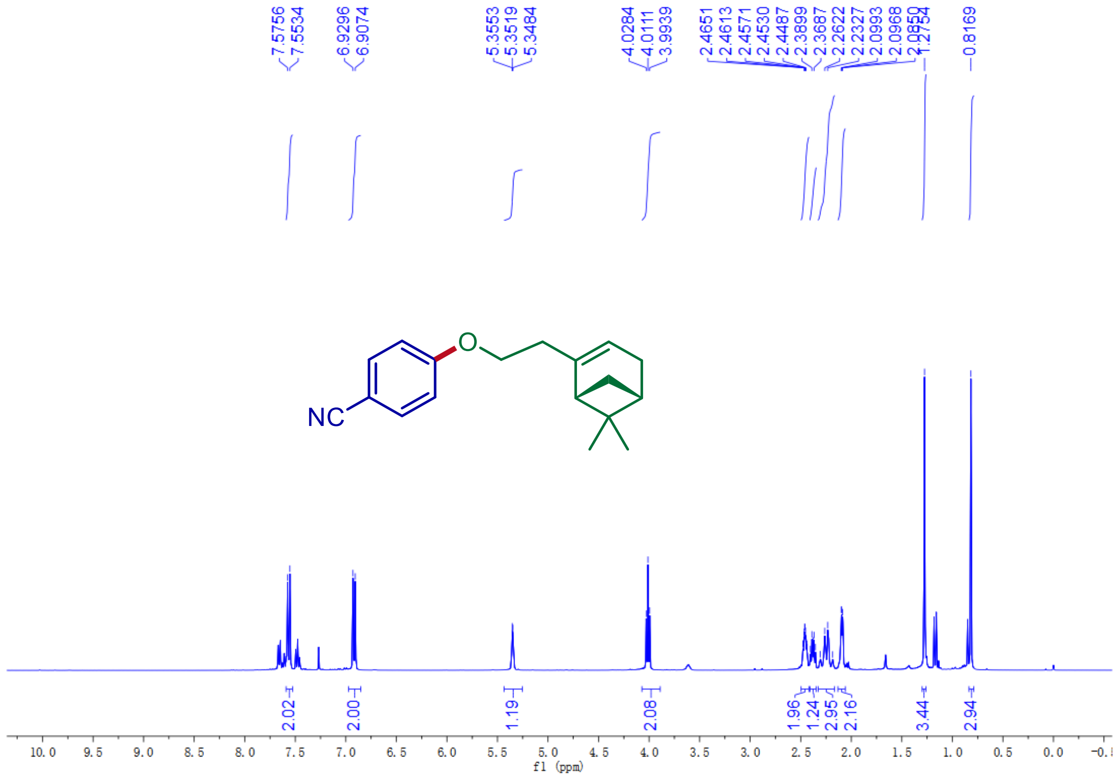

^{1}H NMR (400 MHz, $CDCl_3$) spectrum of compound 152

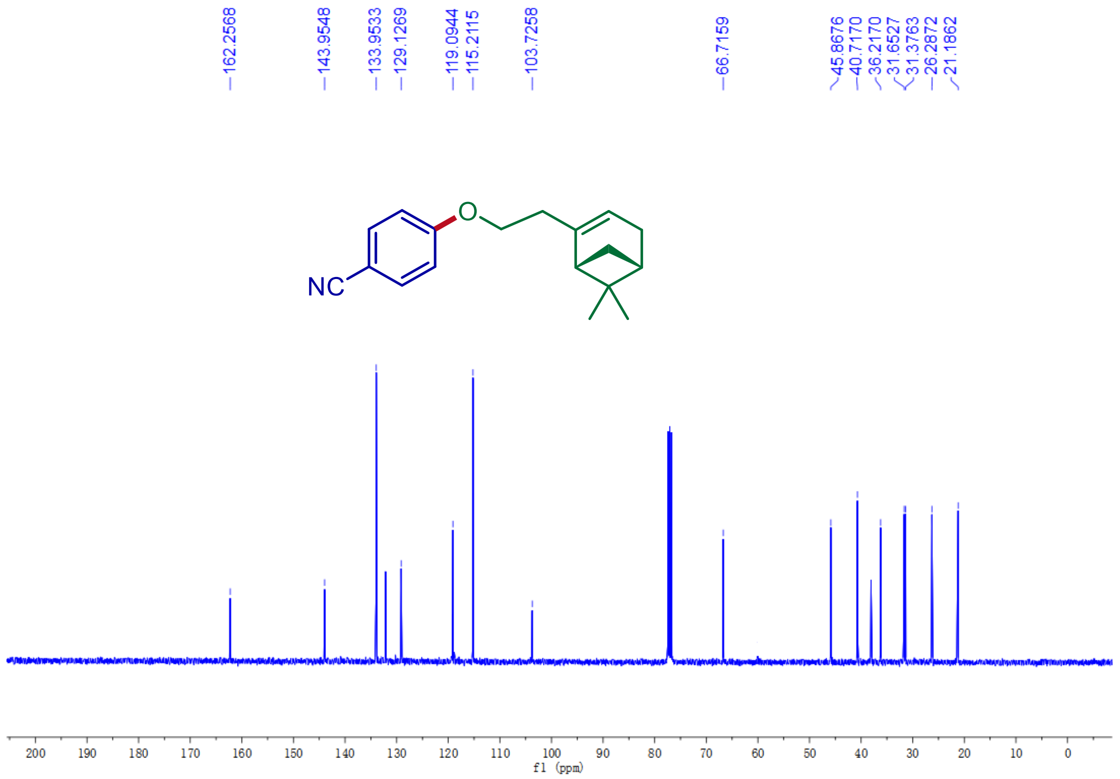

^{13}C NMR (100 MHz, $CDCl_3$) spectrum of compound 152

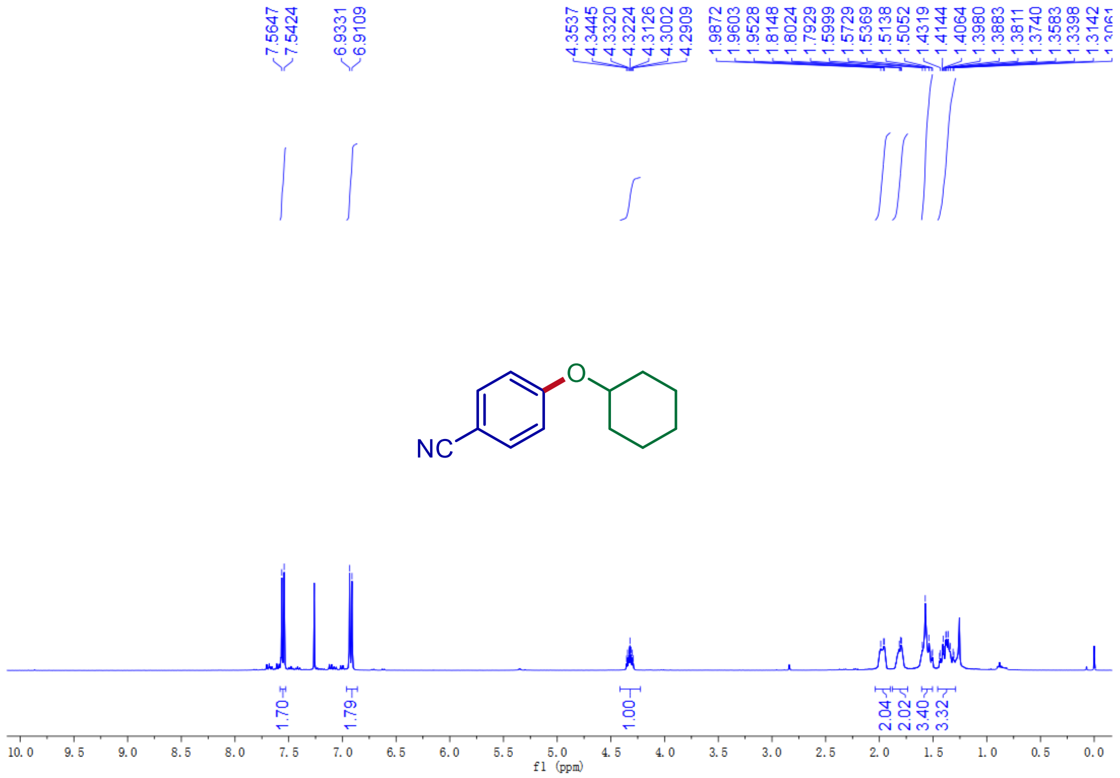

^{1}H NMR (400 MHz, $CDCl_3$) spectrum of compound 153

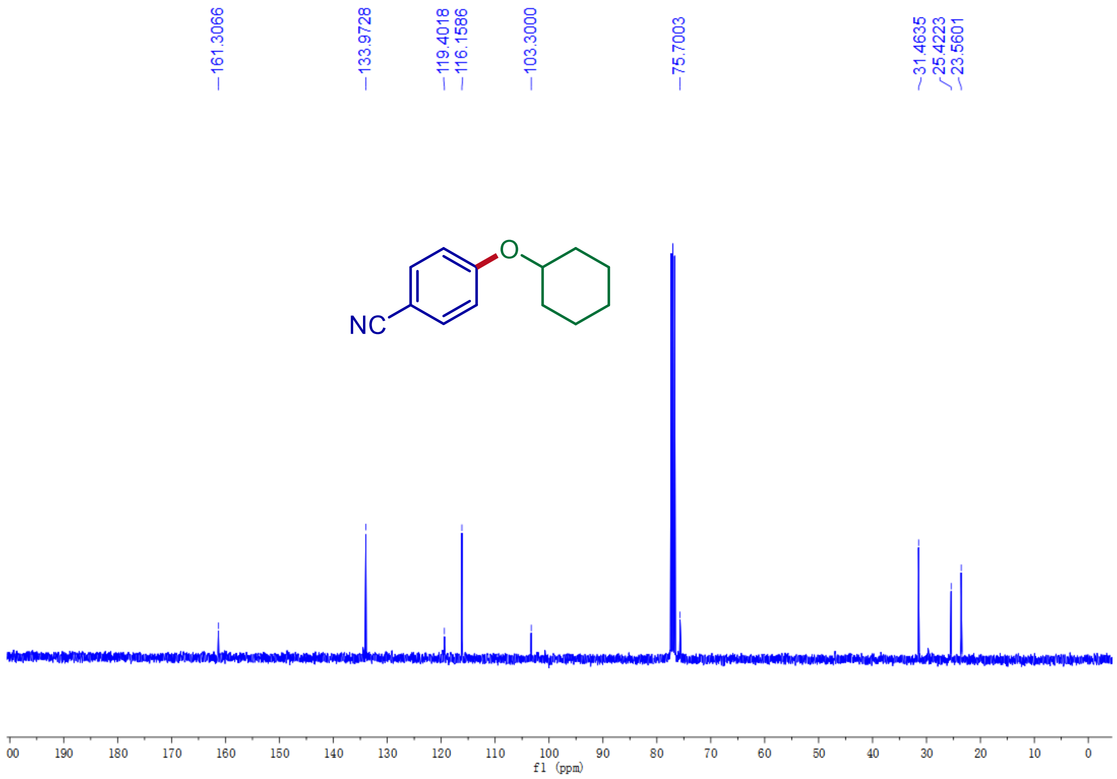

^{13}C NMR (100 MHz, $CDCl_3$) spectrum of compound 153

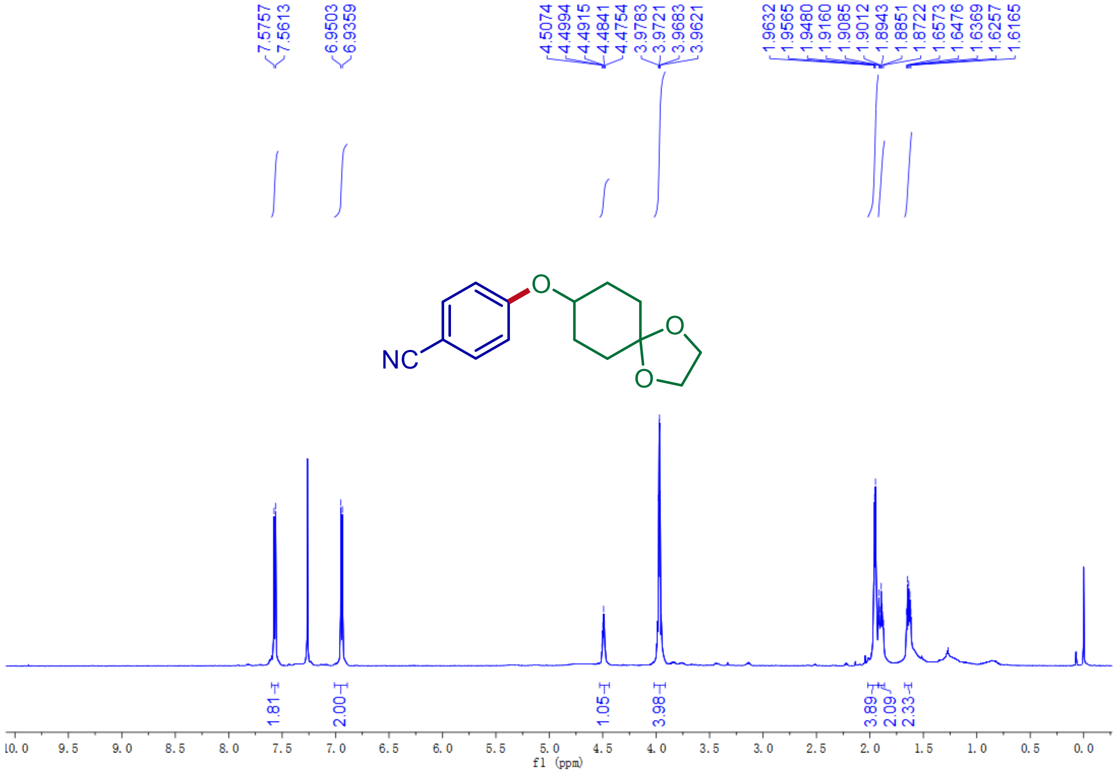

^{1}H NMR (400 MHz, $CDCl_3$) spectrum of compound 154

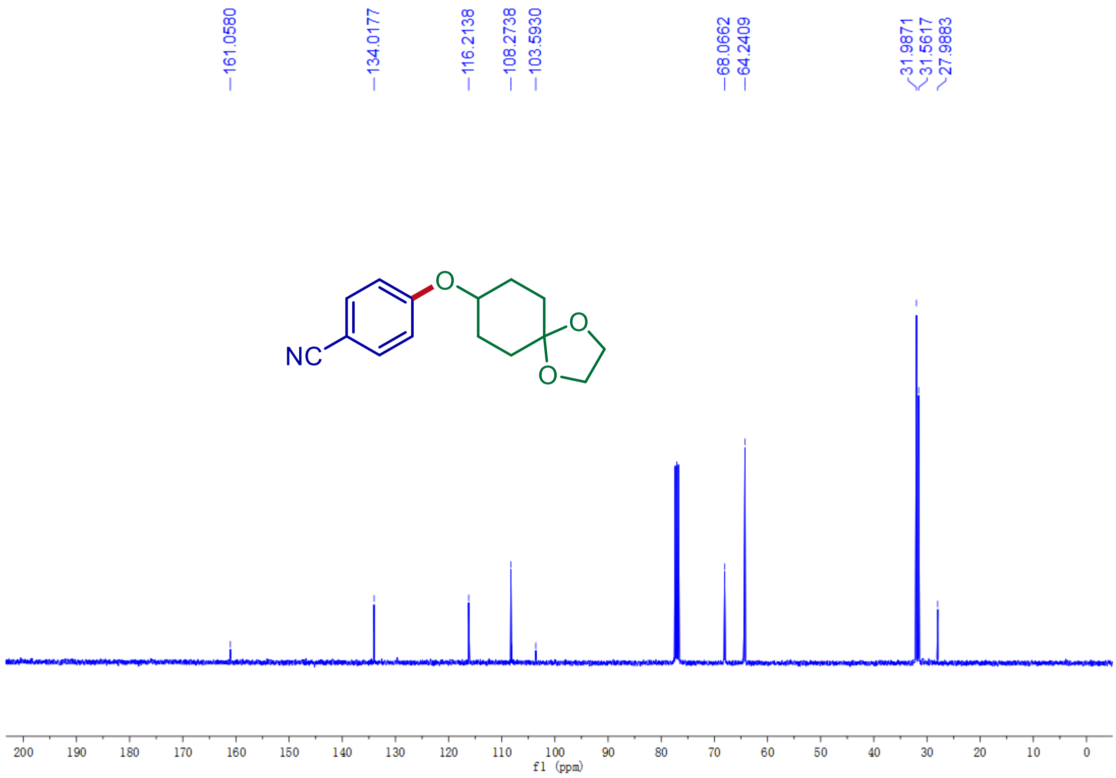

^{13}C NMR (100 MHz, $CDCl_3$) spectrum of compound 154

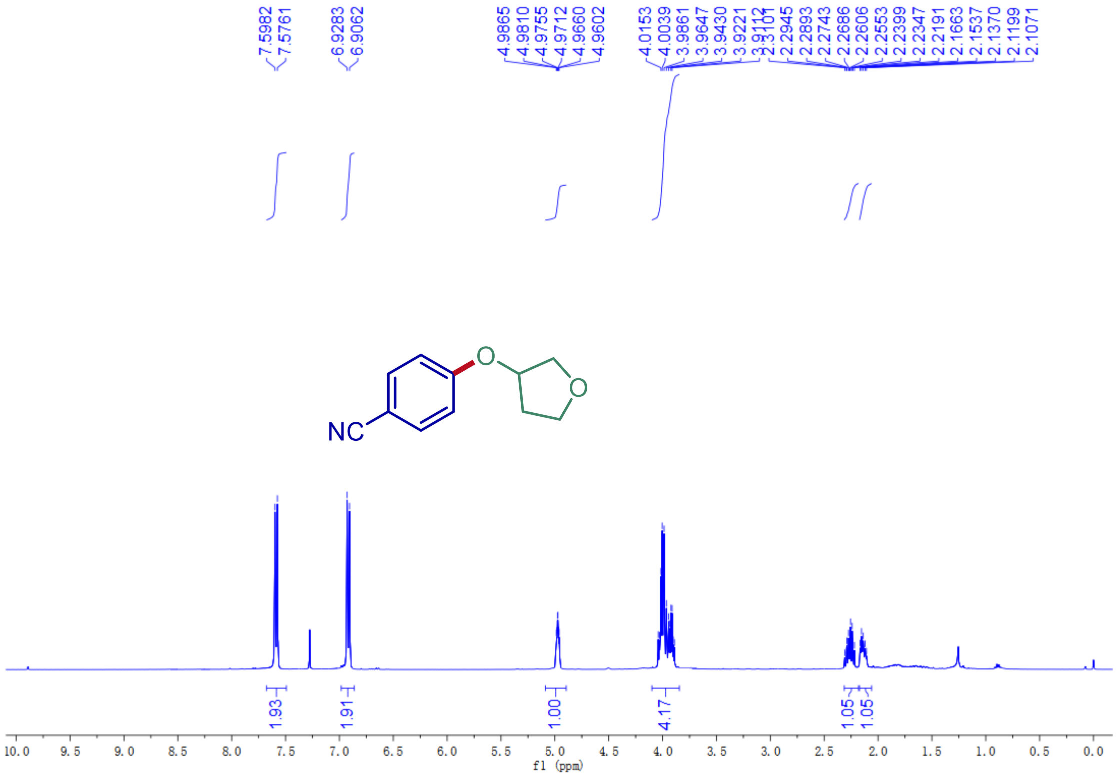

^{1}H NMR (400 MHz, $CDCl_3$) spectrum of compound 155

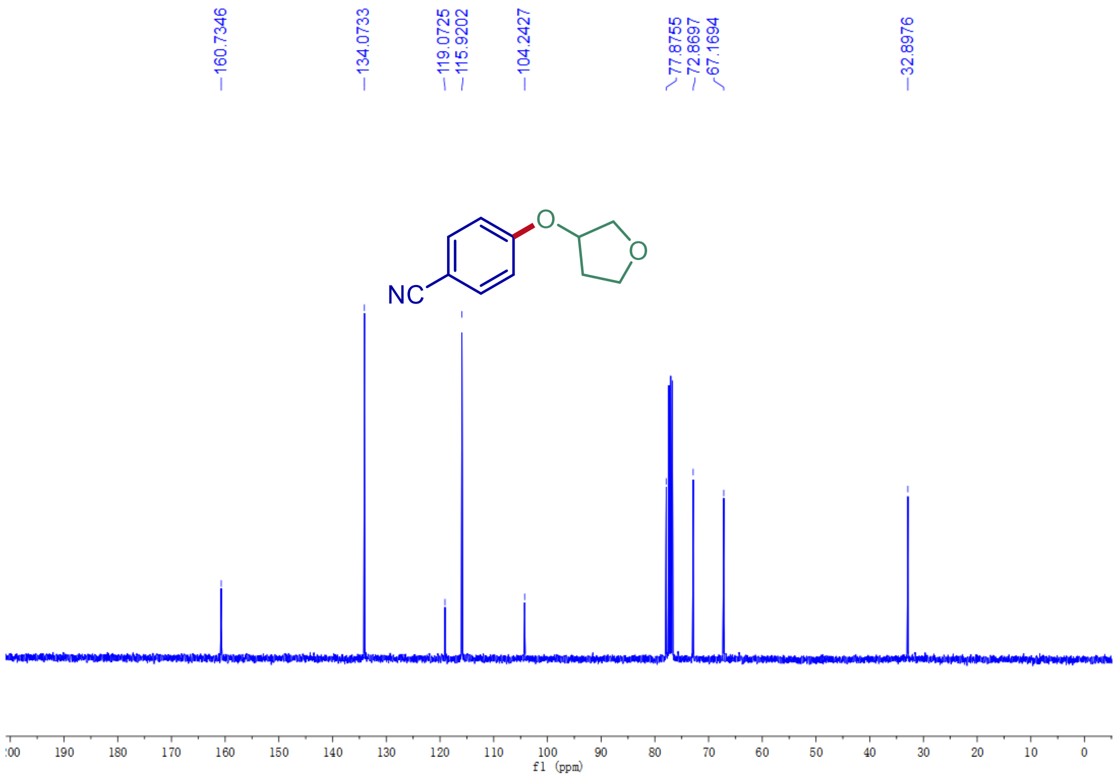

^{13}C NMR (100 MHz, CDCl$_3$) spectrum of compound 155

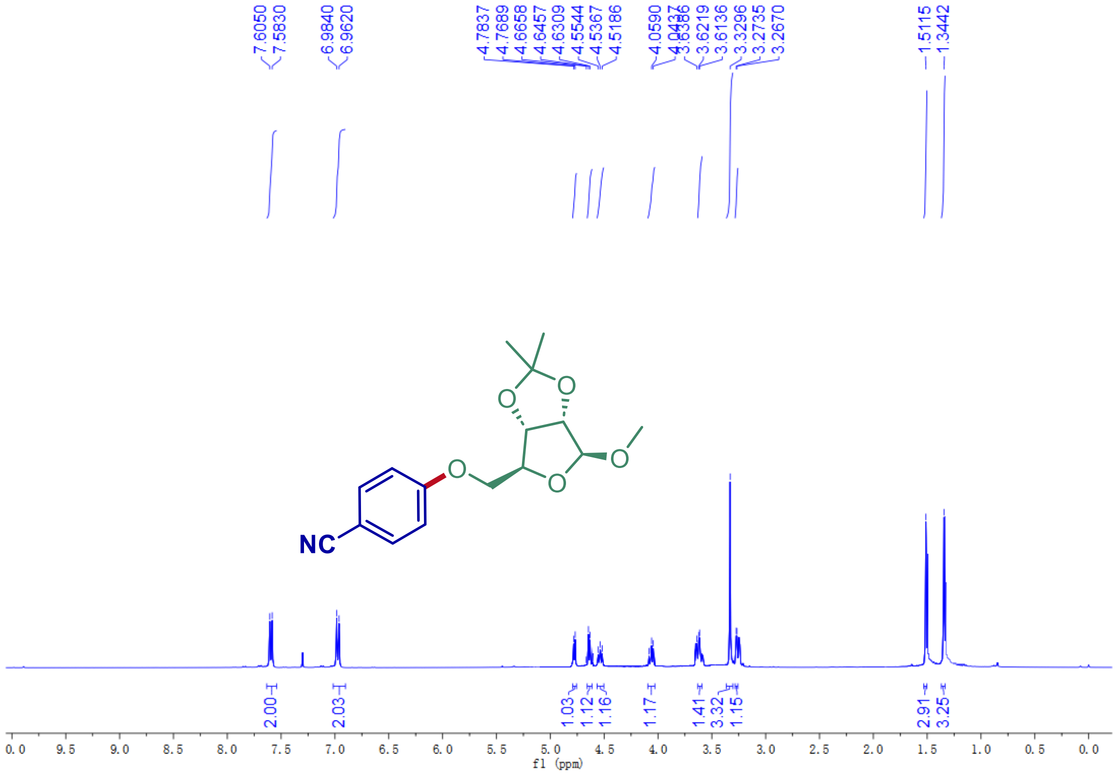

^{1}H NMR (400 MHz, CDCl$_3$) spectrum of compound 156

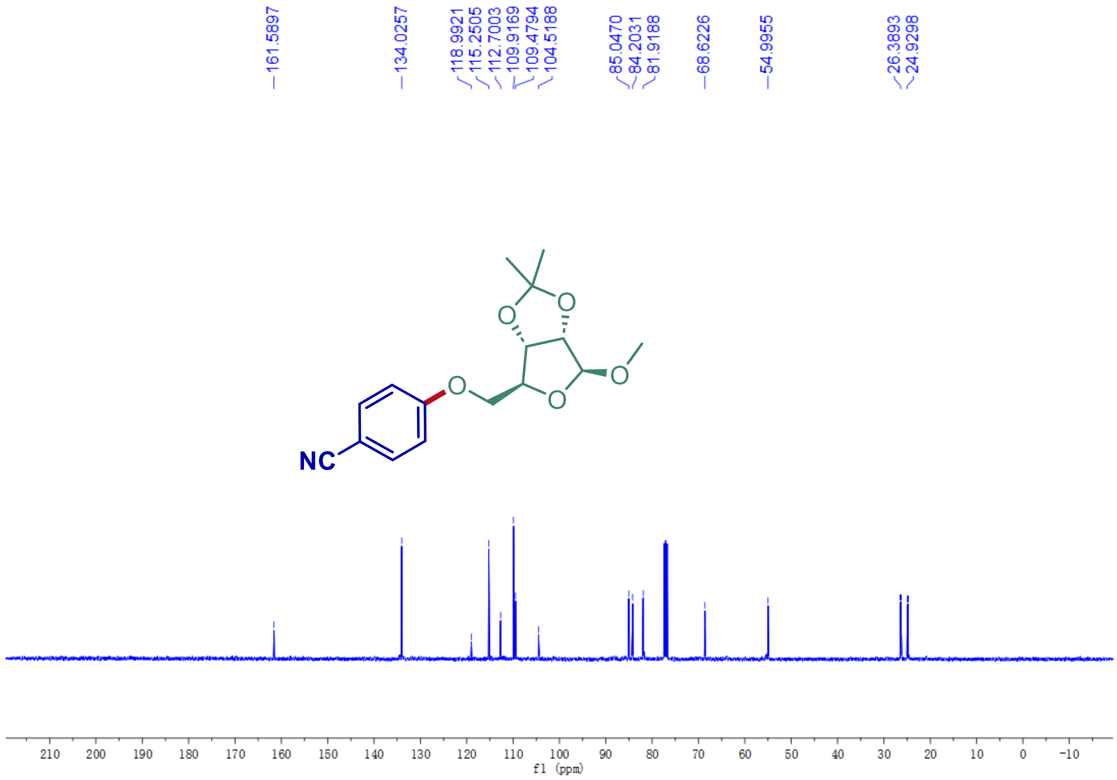

^{13}C NMR (100 MHz, $CDCl_3$) spectrum of compound 156

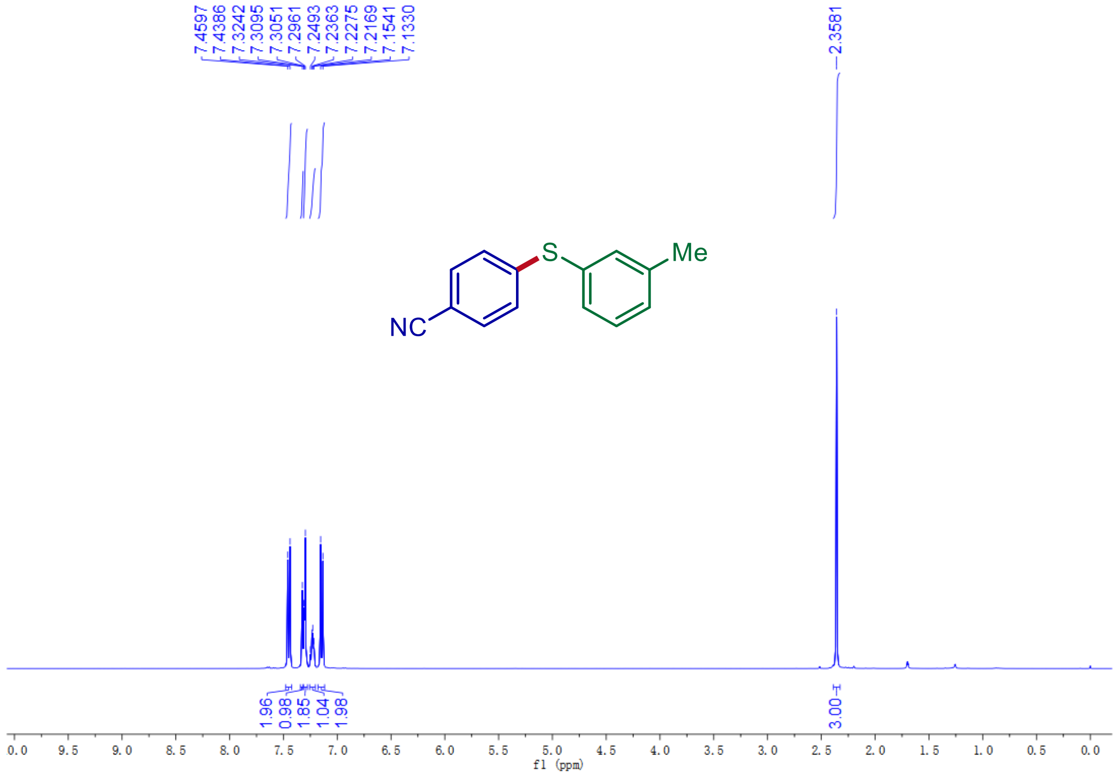

^{1}H NMR (400 MHz, $CDCl_3$) spectrum of compound 157

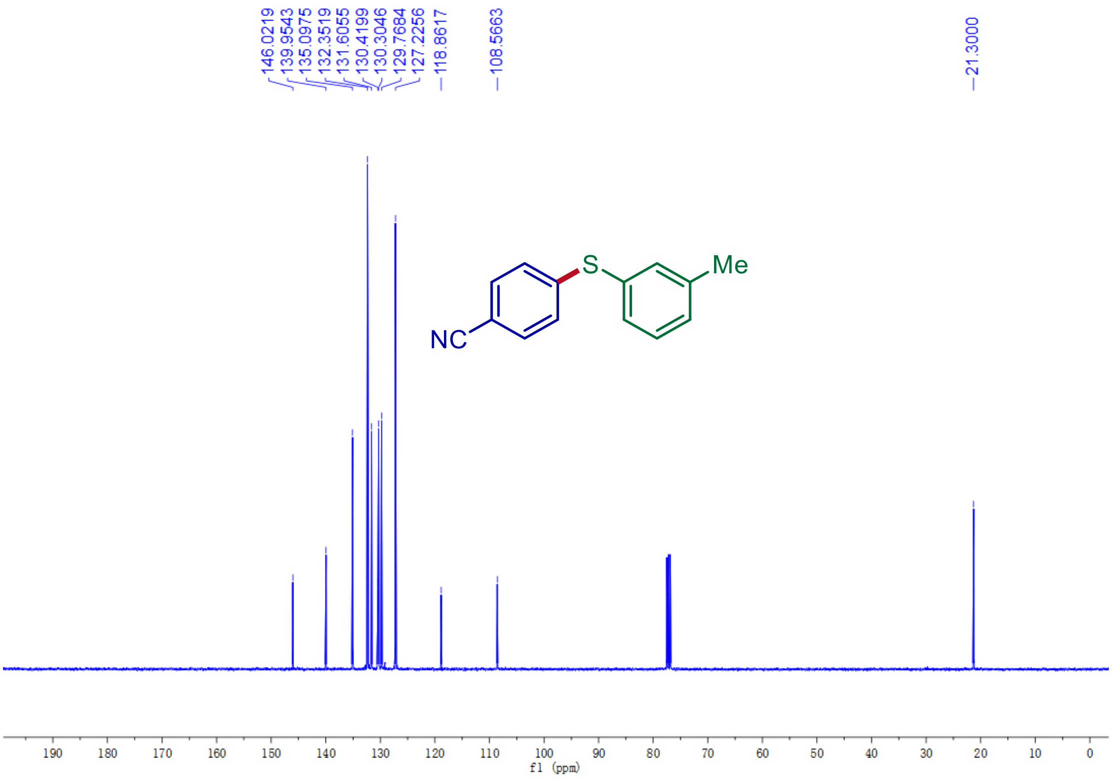

^{13}C NMR (100 MHz, $CDCl_3$) spectrum of compound 157

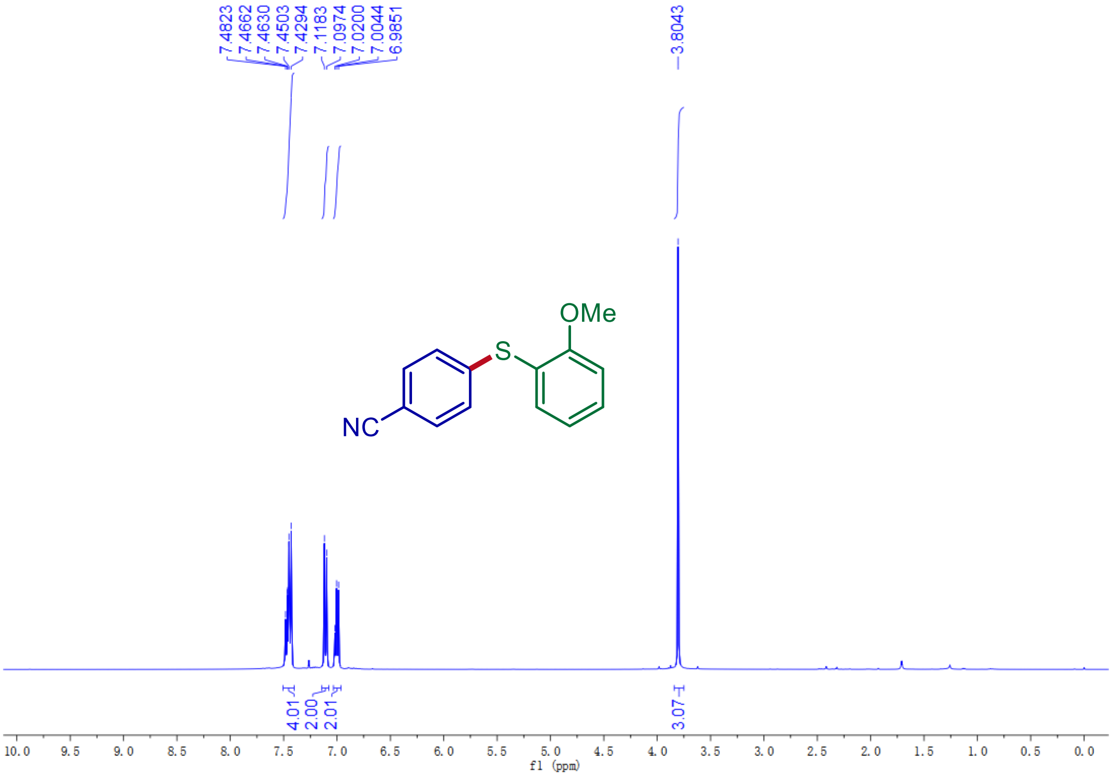

^{1}H NMR (400 MHz, $CDCl_3$) spectrum of compound 158

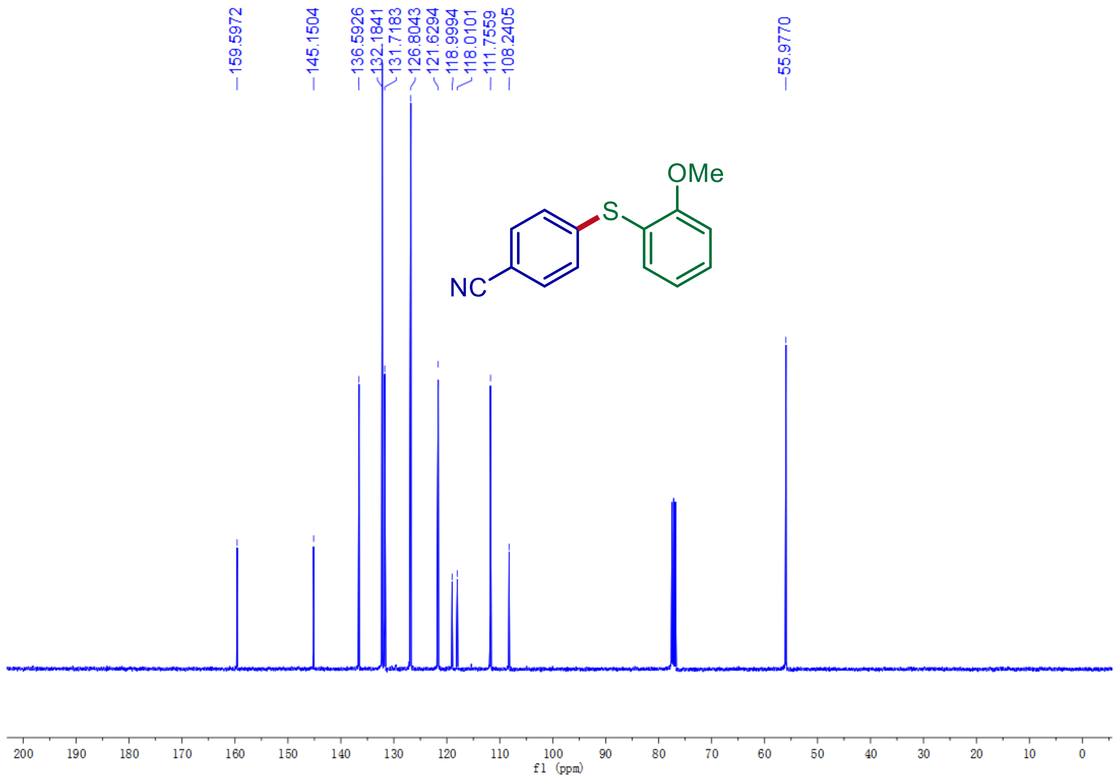

^{13}C NMR (100 MHz, $CDCl_3$) spectrum of compound 158

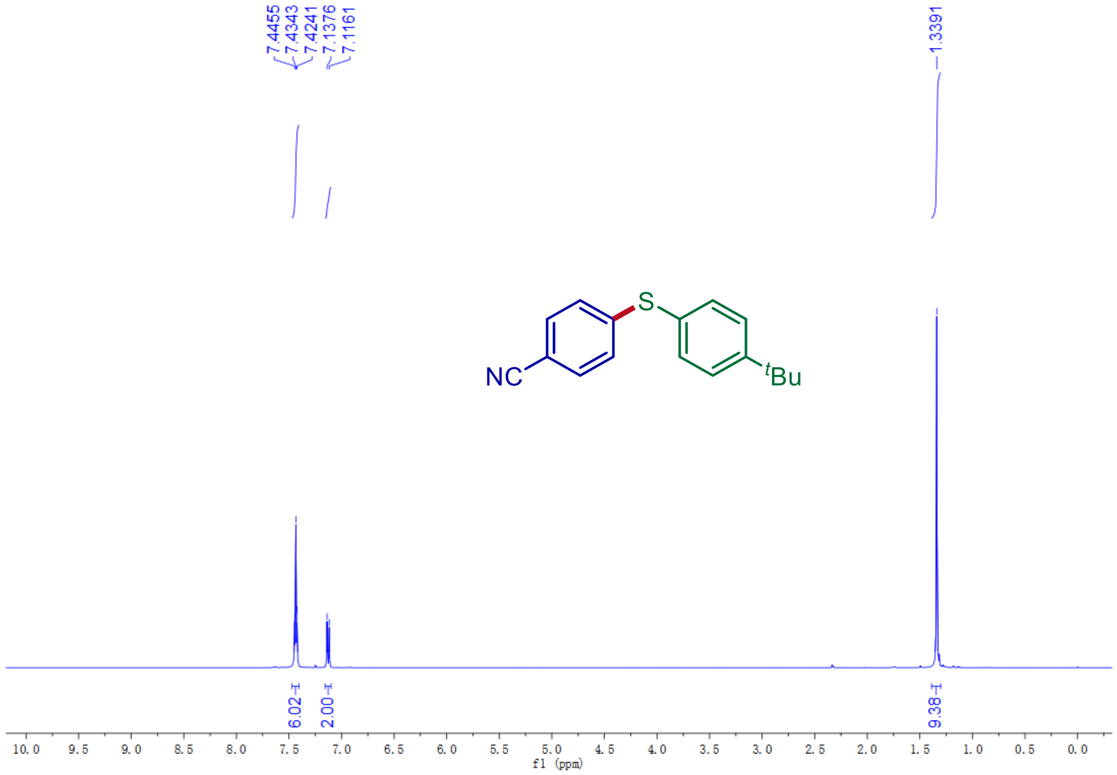

^{1}H NMR (400 MHz, $CDCl_3$) spectrum of compound 159

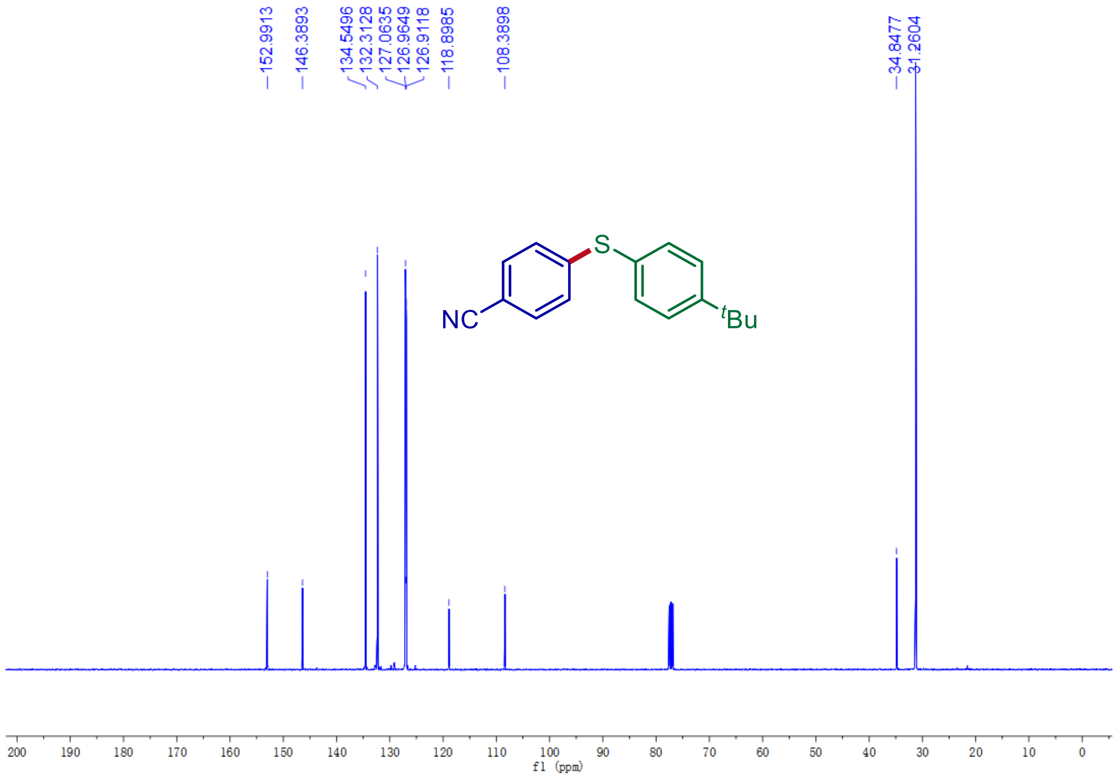

^{13}C NMR (100 MHz, $CDCl_3$) spectrum of compound 159

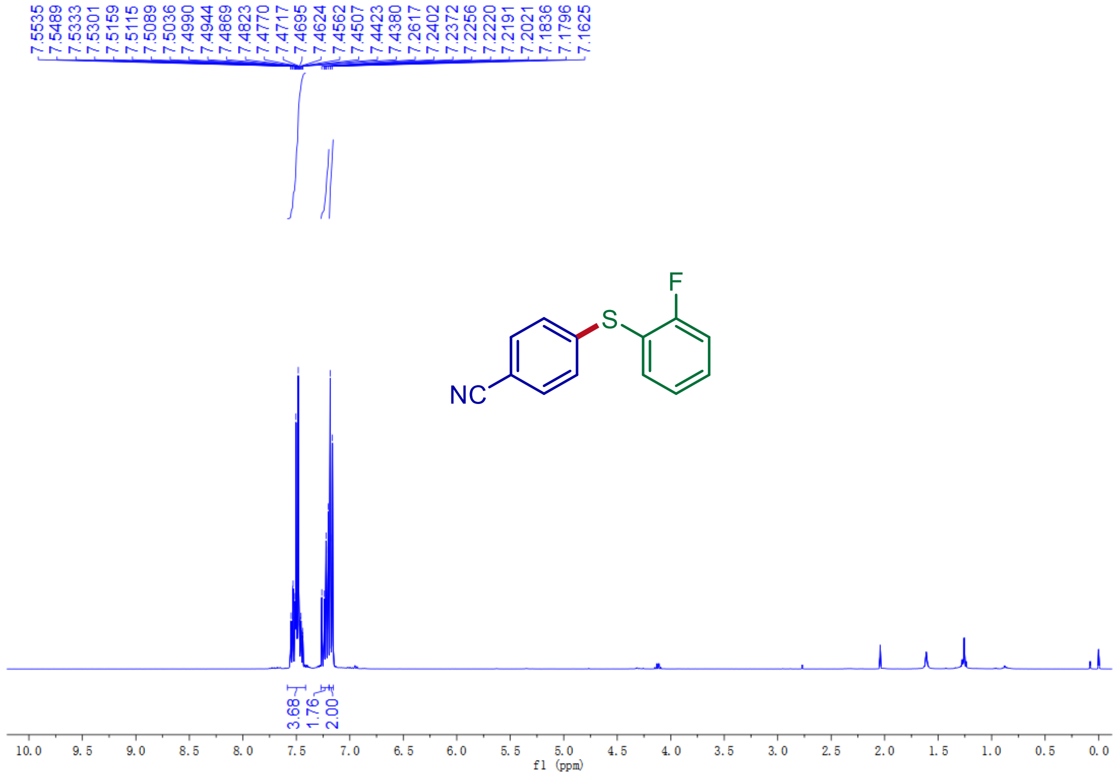

^{1}H NMR (400 MHz, $CDCl_3$) spectrum of compound 160

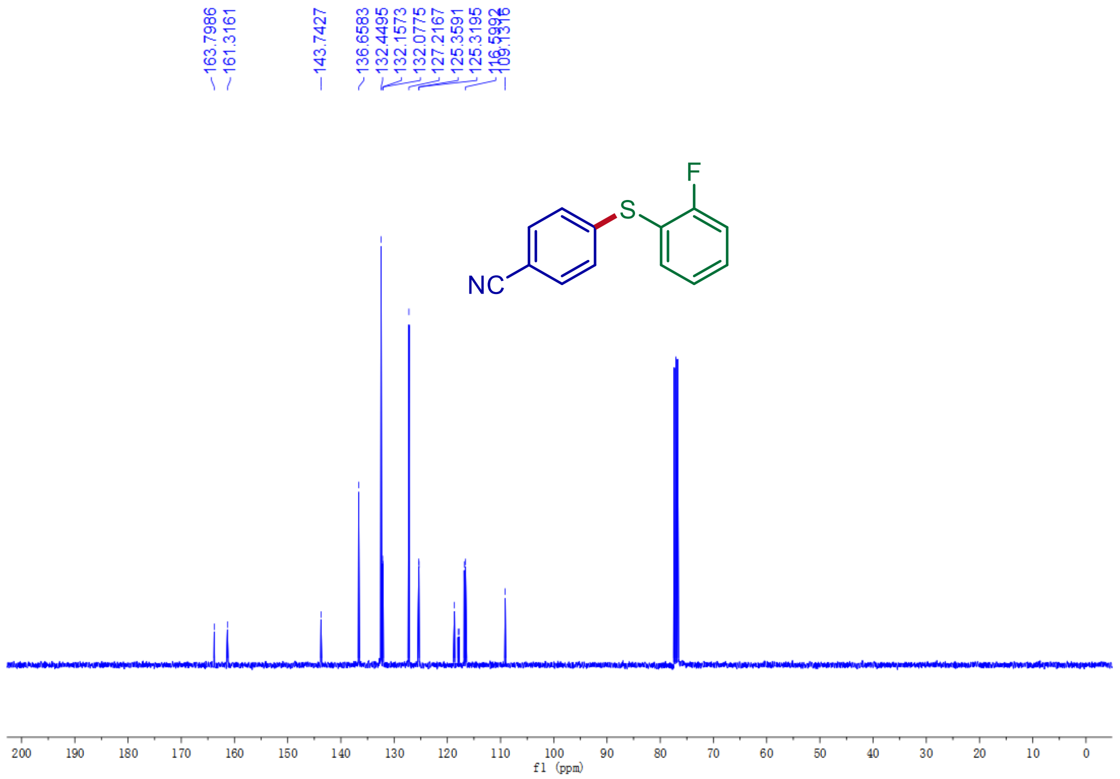

^{13}C NMR (100 MHz, CDCl$_3$) spectrum of compound 160

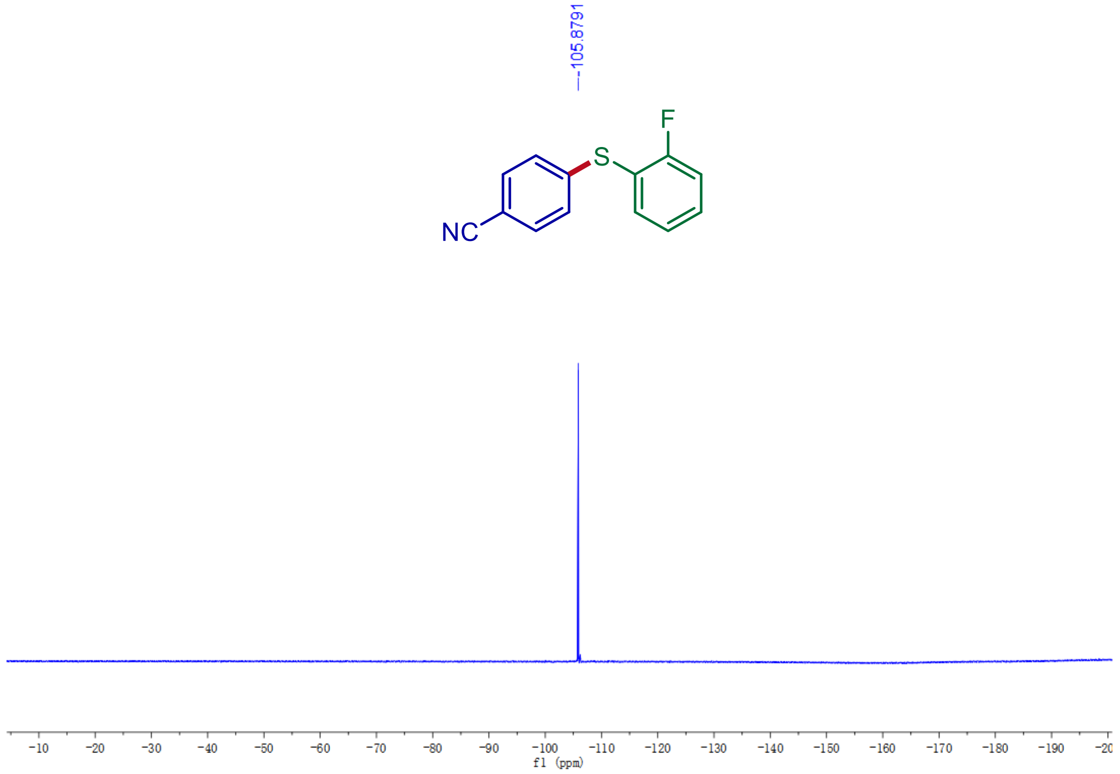

^{19}F NMR (376 MHz, CDCl$_3$) spectrum of compound 160

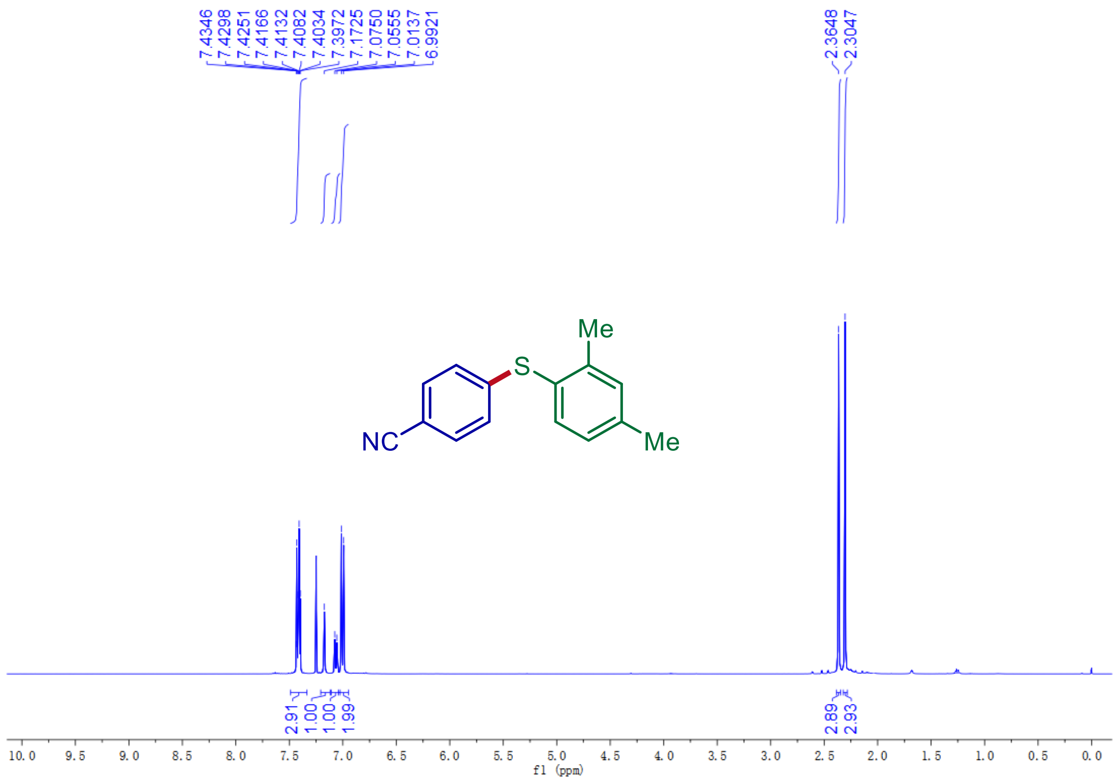

^1H NMR (400 MHz, $CDCl_3$) spectrum of compound 161

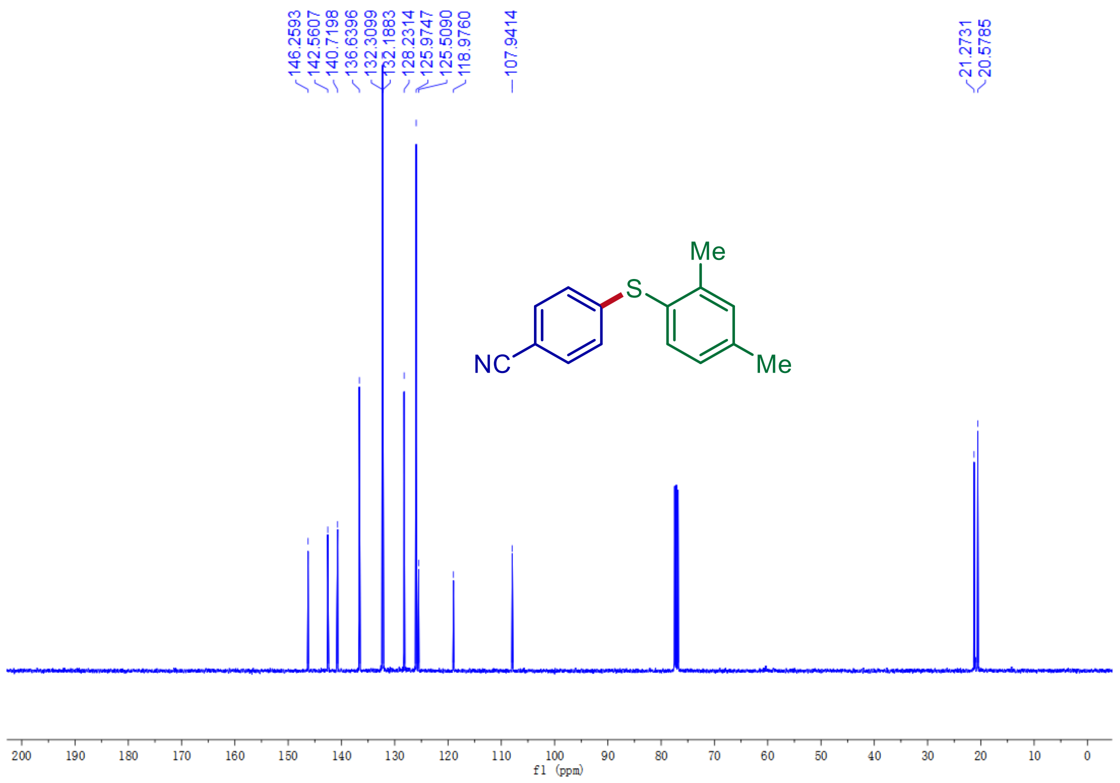

^{13}C NMR (100 MHz, $CDCl_3$) spectrum of compound 161

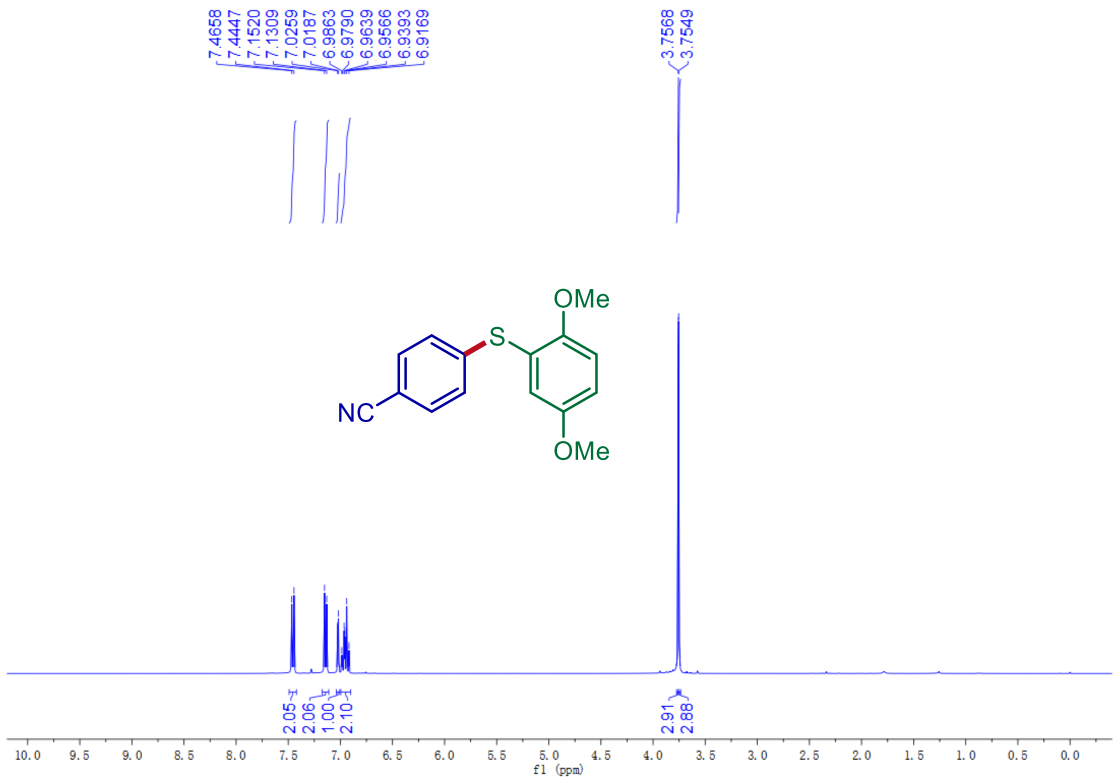

^{1}H NMR (400 MHz, $CDCl_3$) spectrum of compound 162

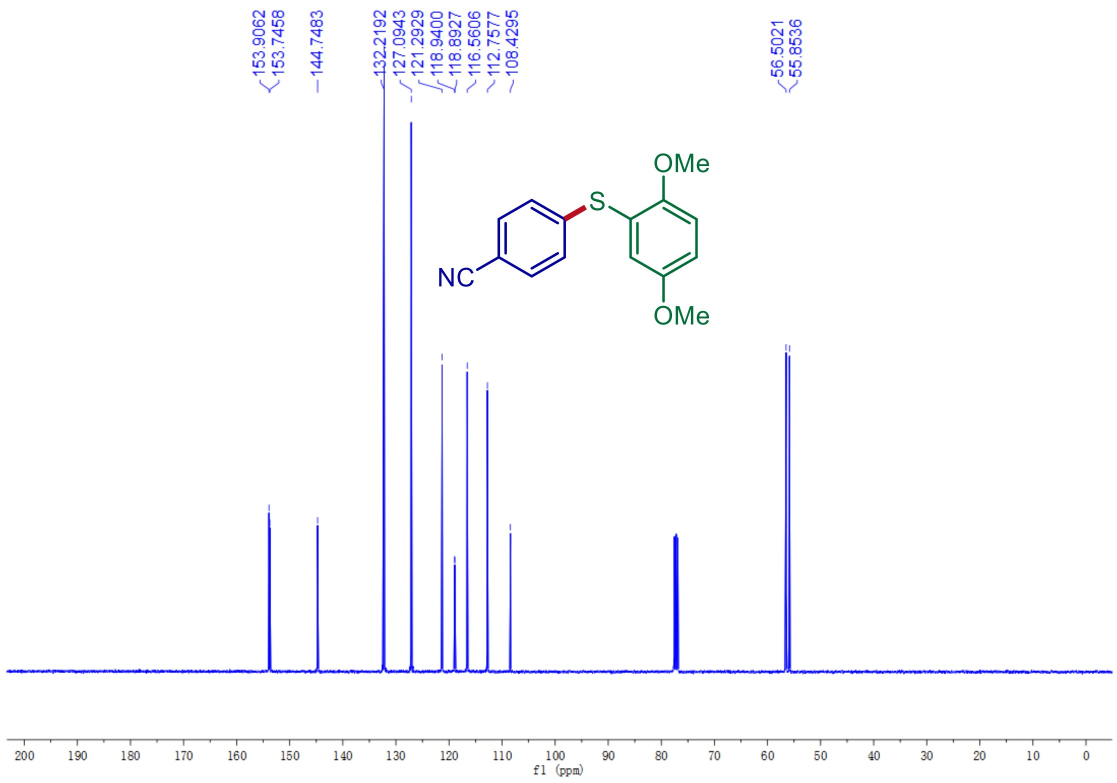

^{13}C NMR (100 MHz, $CDCl_3$) spectrum of compound 162

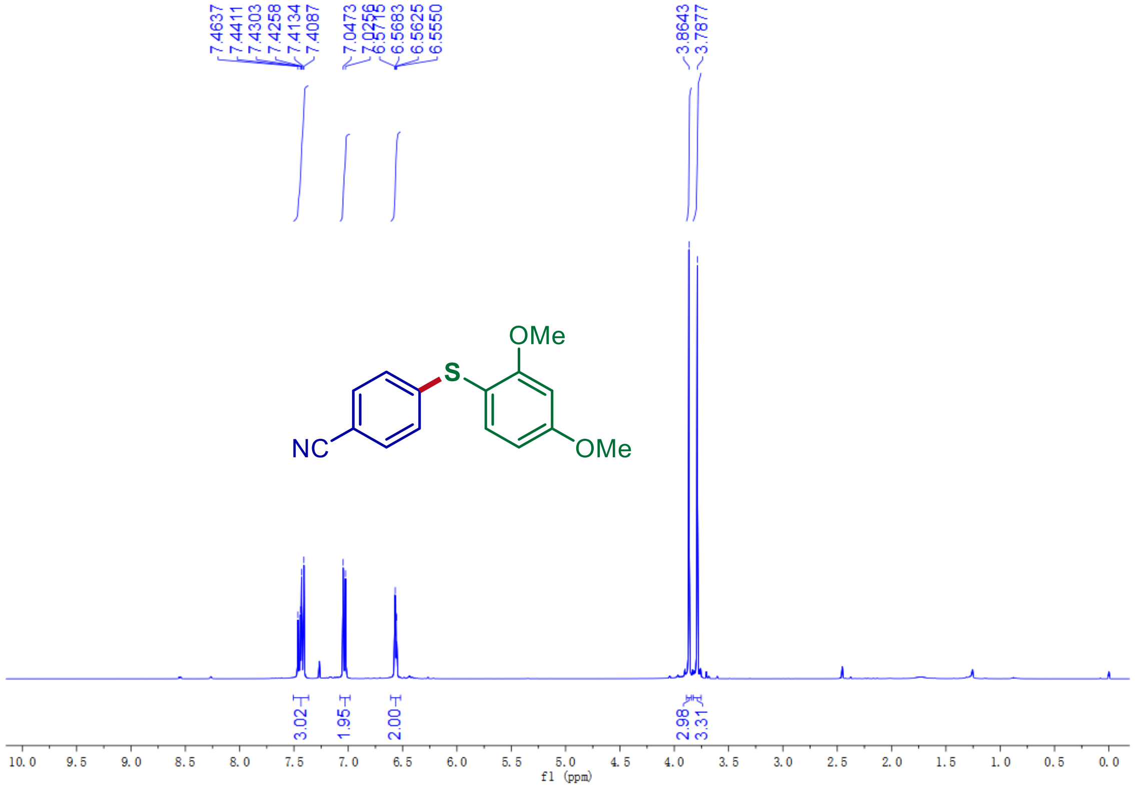

^{1}H NMR (400 MHz, $CDCl_3$) spectrum of compound 163

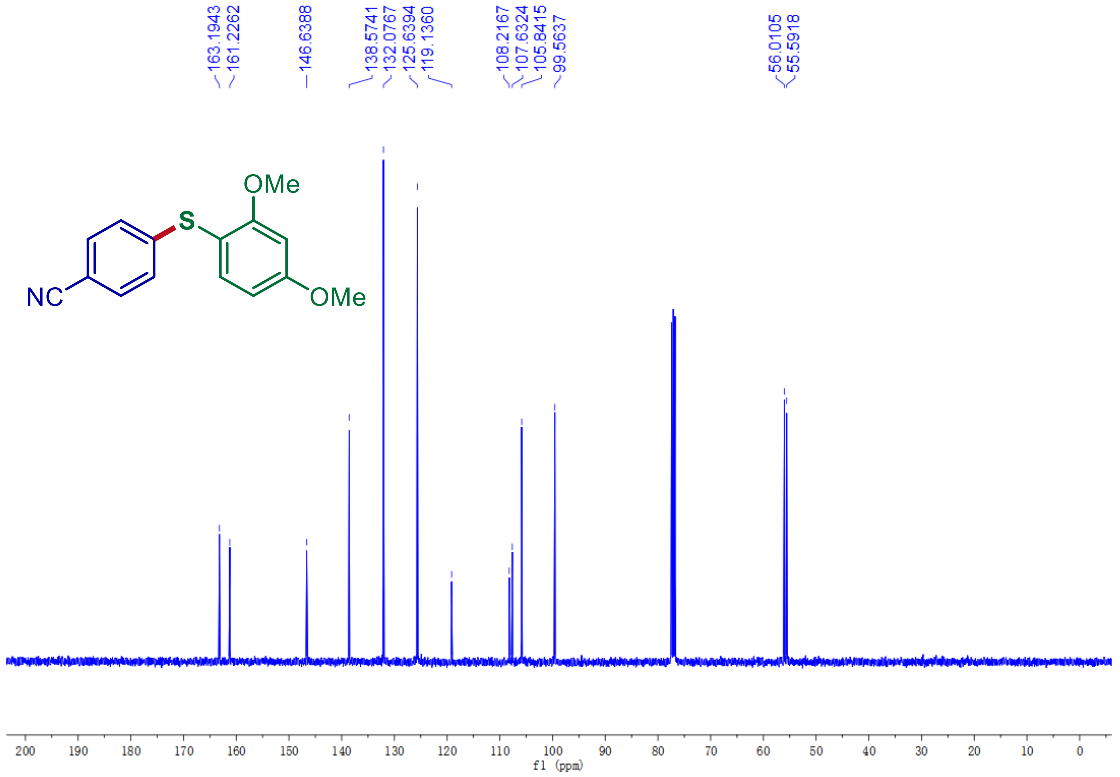

^{13}C NMR (100 MHz, $CDCl_3$) spectrum of compound 163

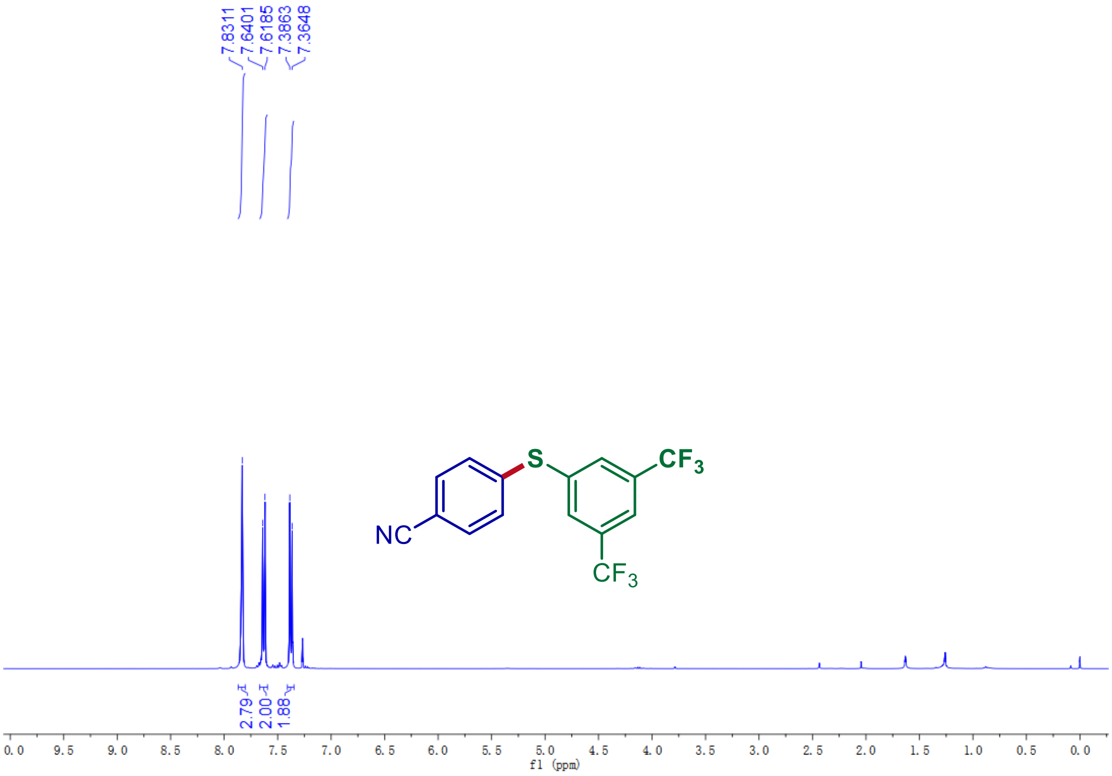

^{1}H NMR (400 MHz, $CDCl_3$) spectrum of compound 164

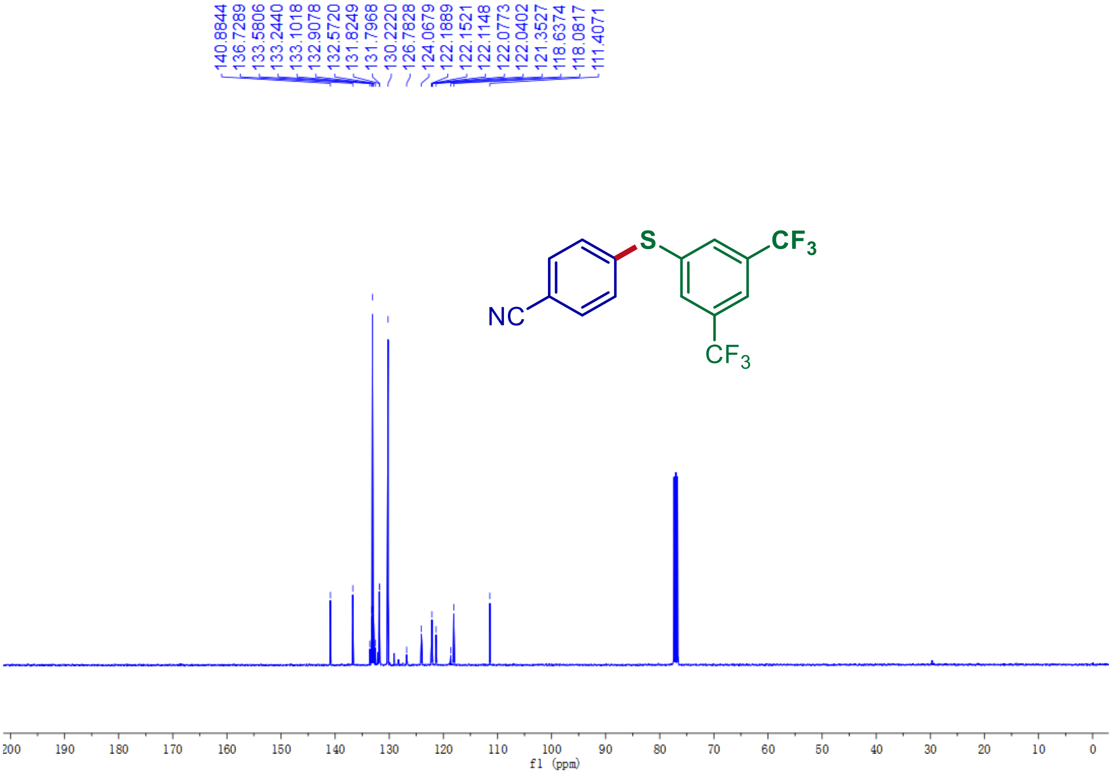

^{13}C NMR (100 MHz, $CDCl_3$) spectrum of compound 164

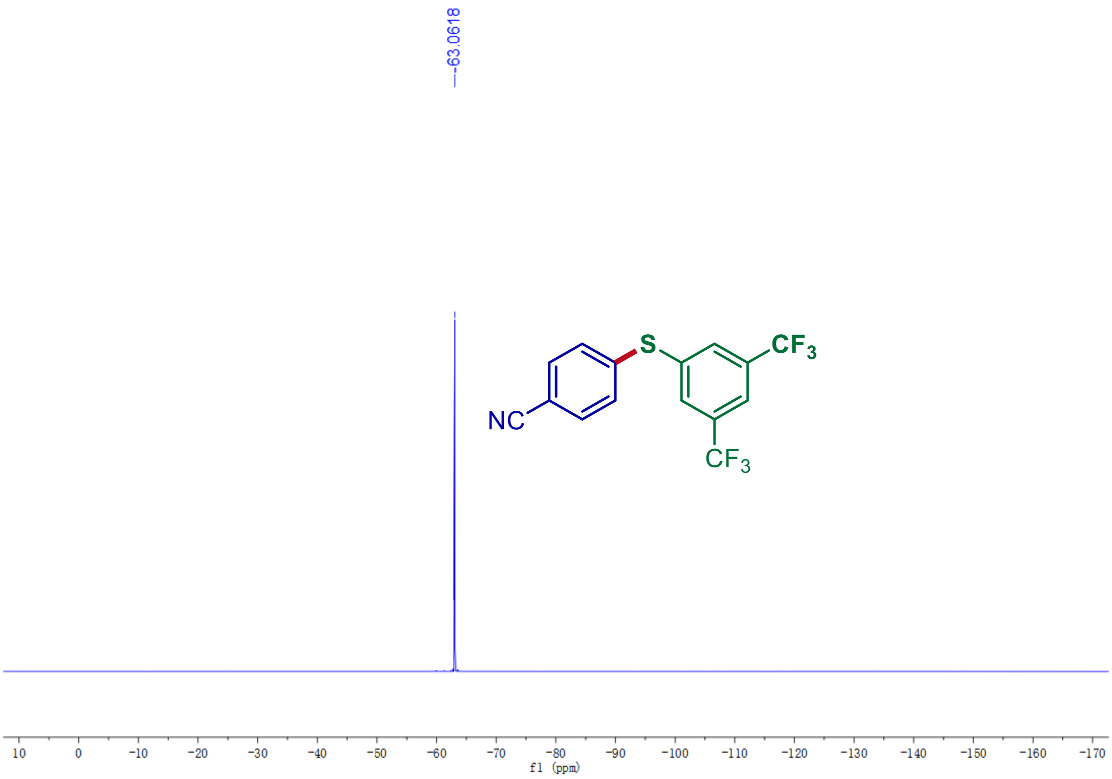

^{19}F NMR (376 MHz, $CDCl_3$) spectrum of compound 164

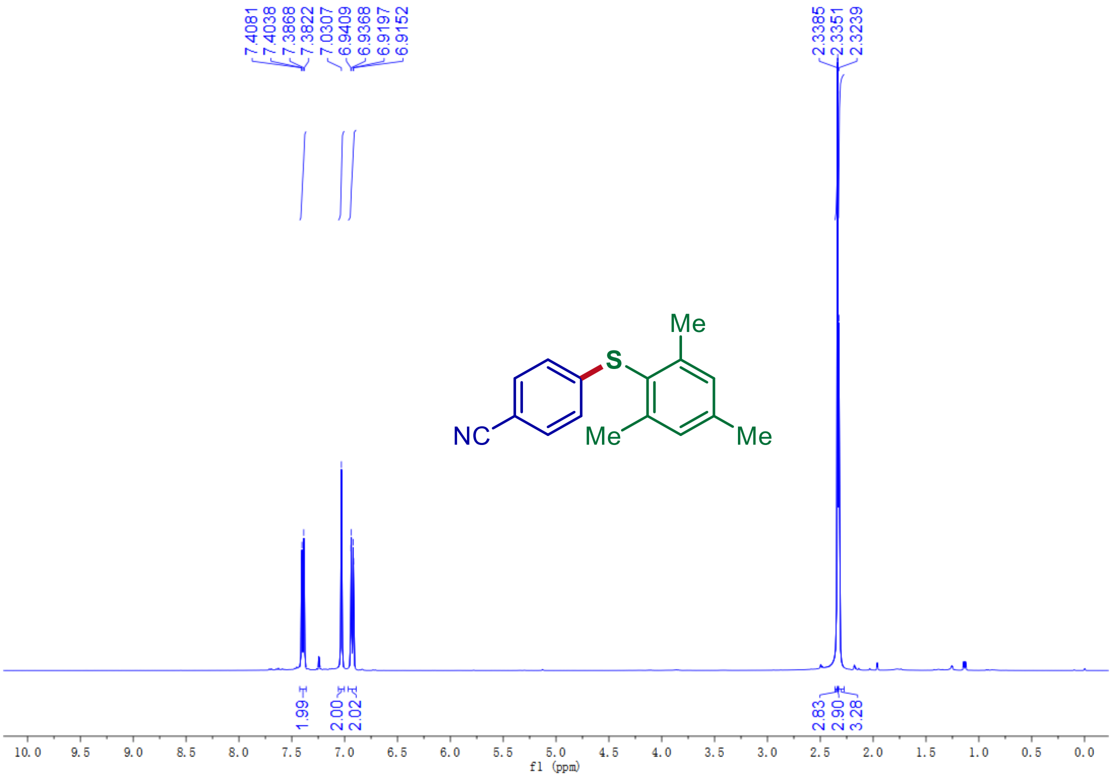

^{1}H NMR (400 MHz, $CDCl_3$) spectrum of compound 165

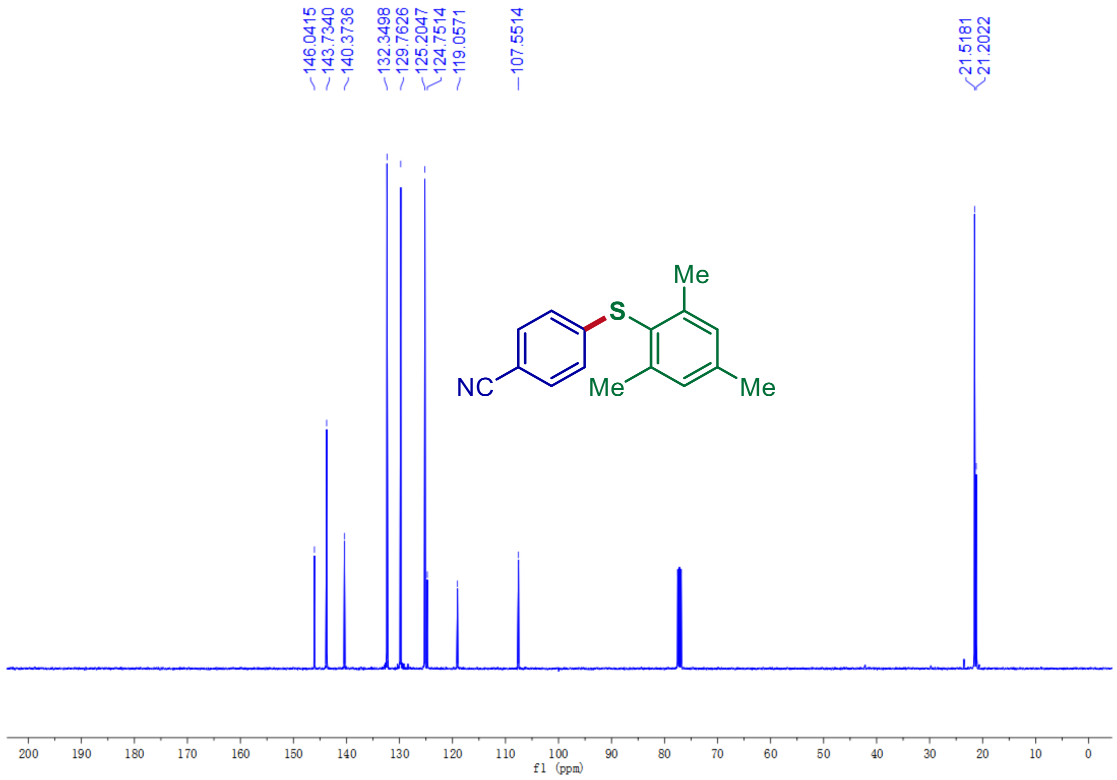

^{13}C NMR (100 MHz, $CDCl_3$) spectrum of compound 165

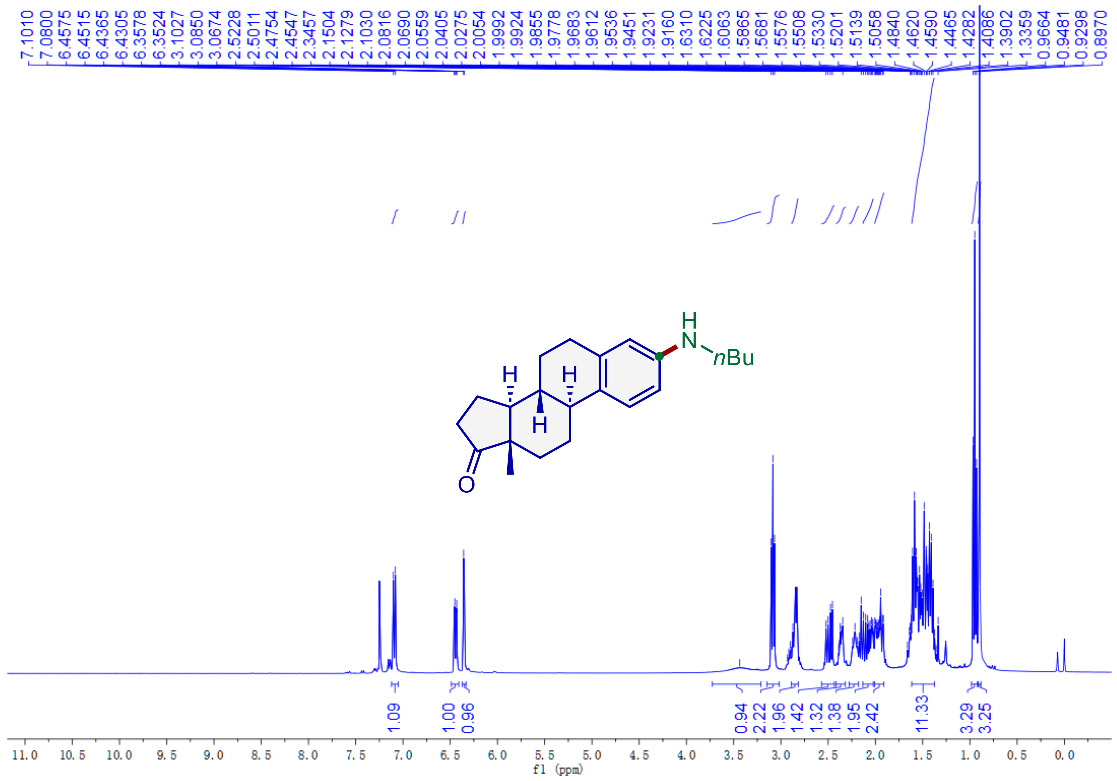

^{1}H NMR (400 MHz, $CDCl_3$) spectrum of compound 166

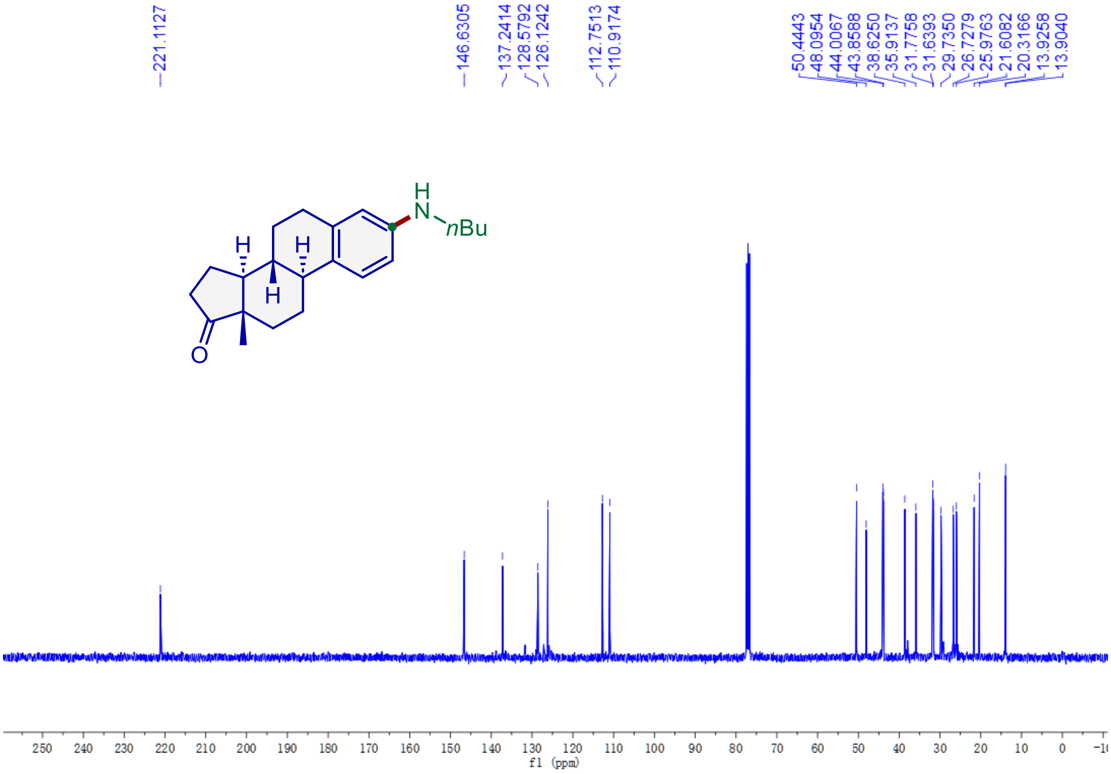

^{13}C NMR (100 MHz, $CDCl_3$) spectrum of compound 166

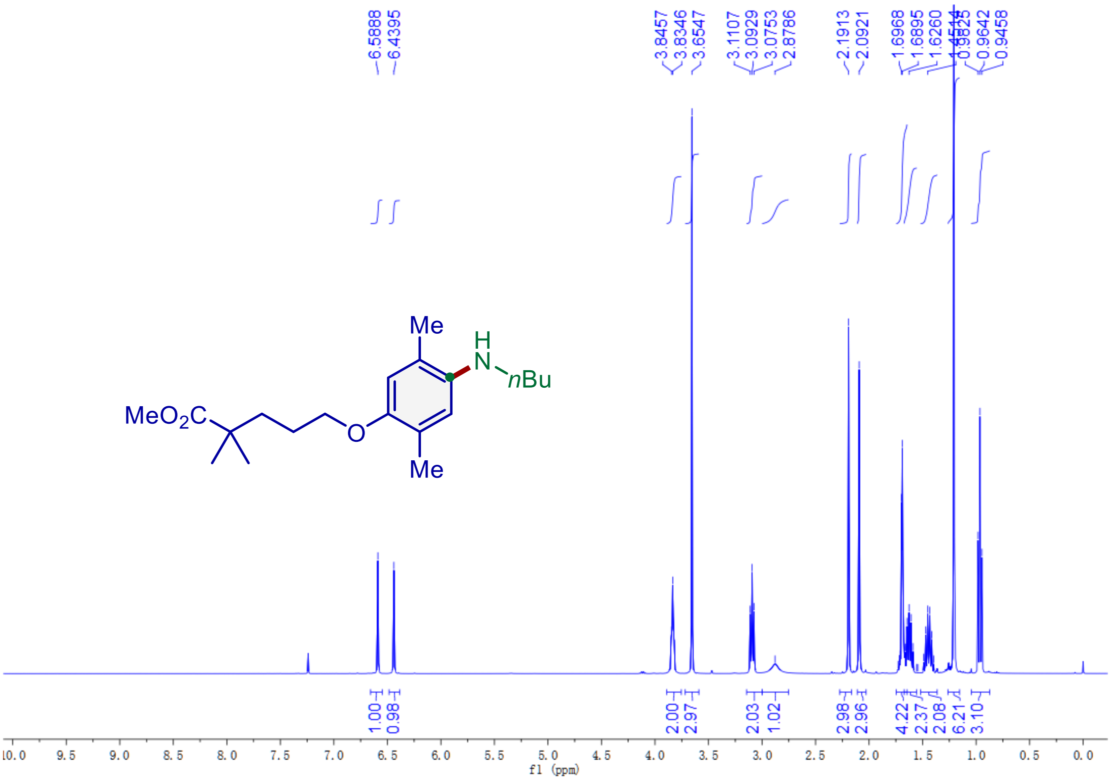

^{1}H NMR (400 MHz, $CDCl_3$) spectrum of compound 167

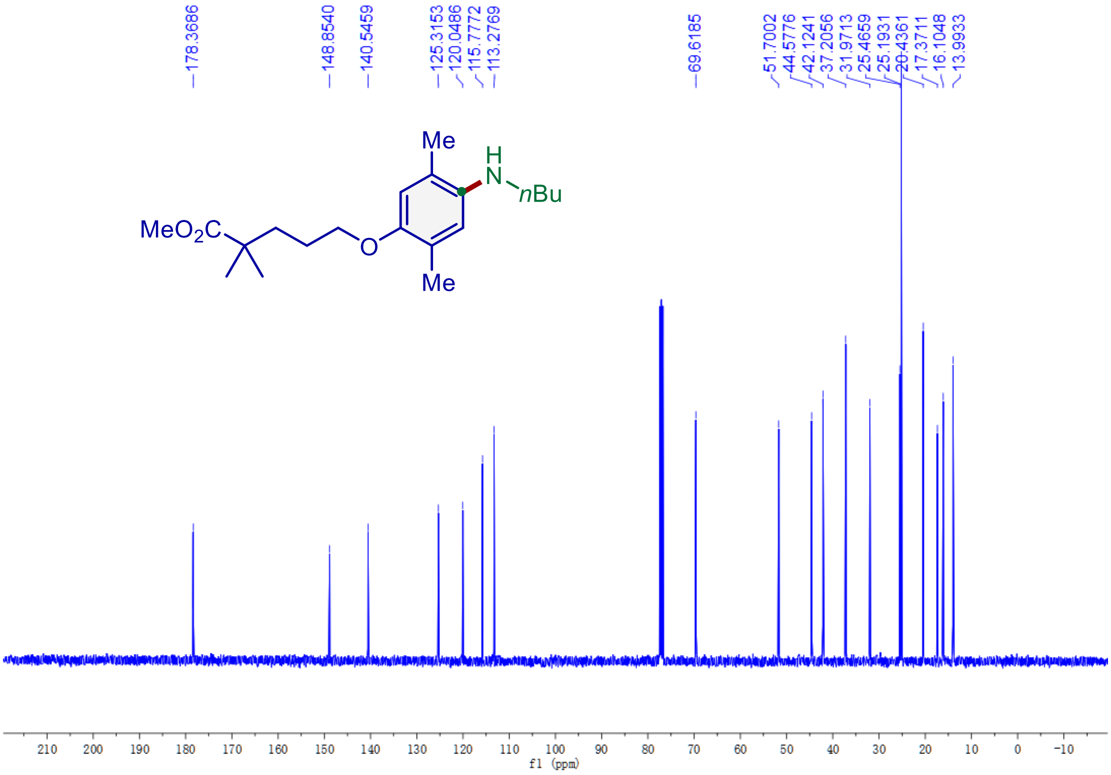

^{13}C NMR (100 MHz, $CDCl_3$) spectrum of compound 167

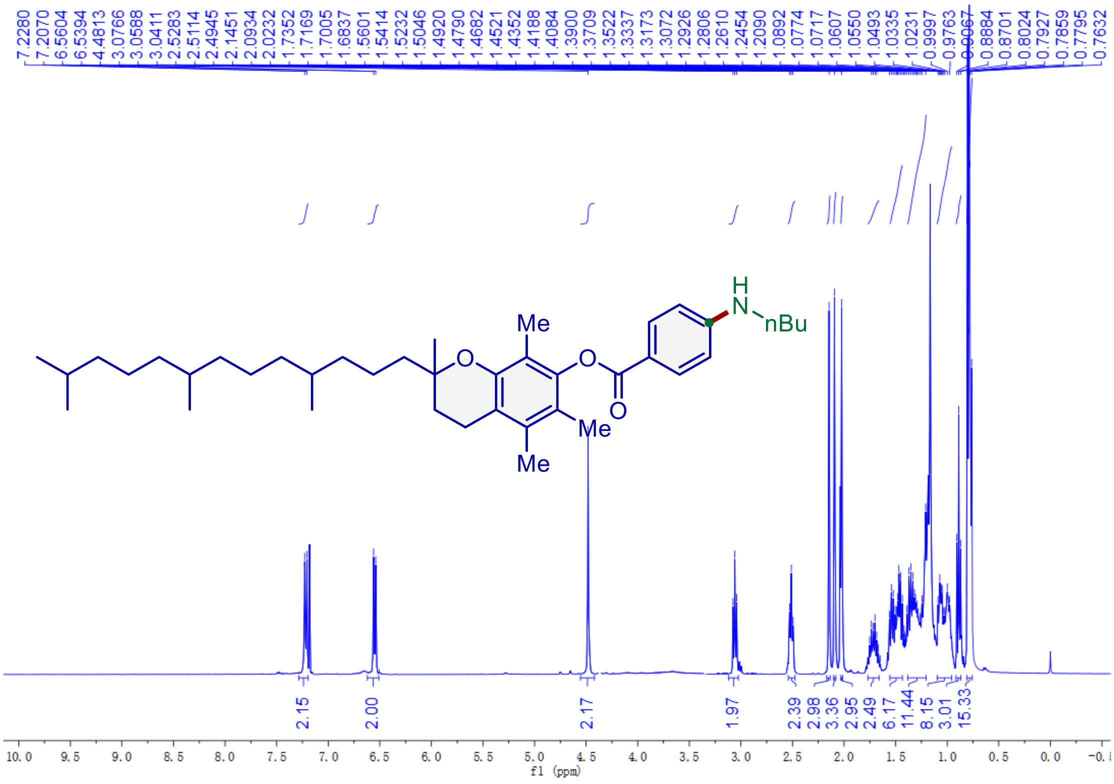

^{1}H NMR (400 MHz, $CDCl_3$) spectrum of compound 168

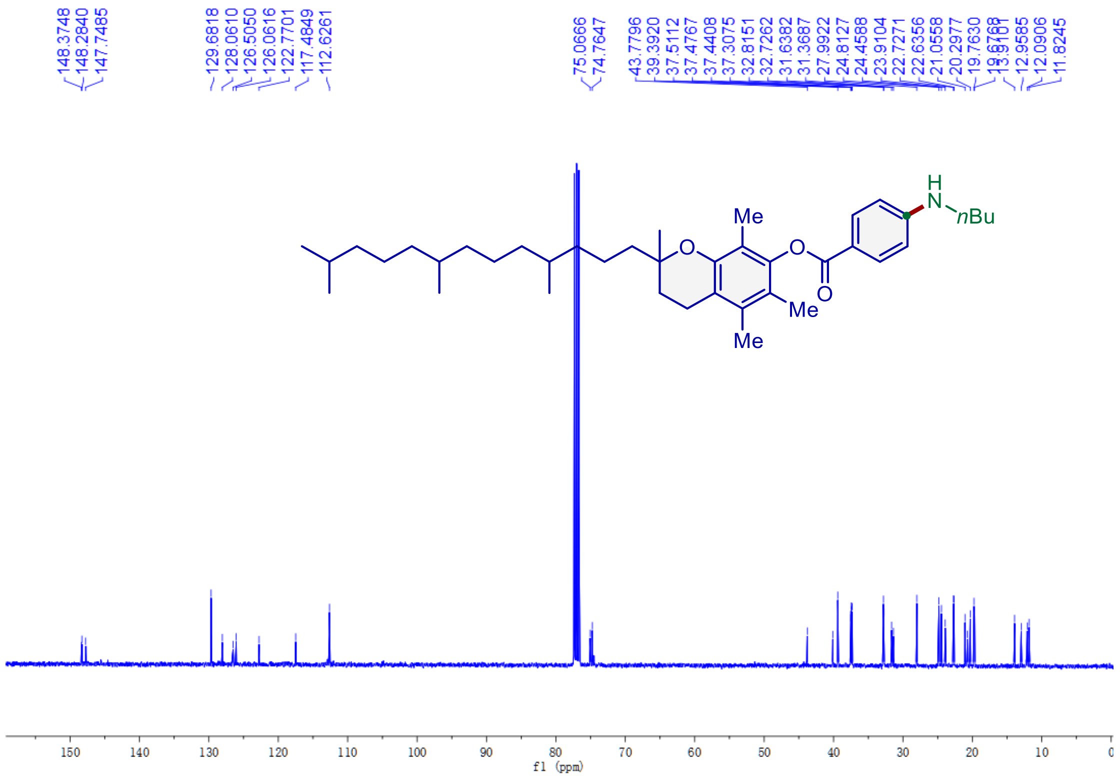

^{13}C NMR (100 MHz, $CDCl_3$) spectrum of compound 168

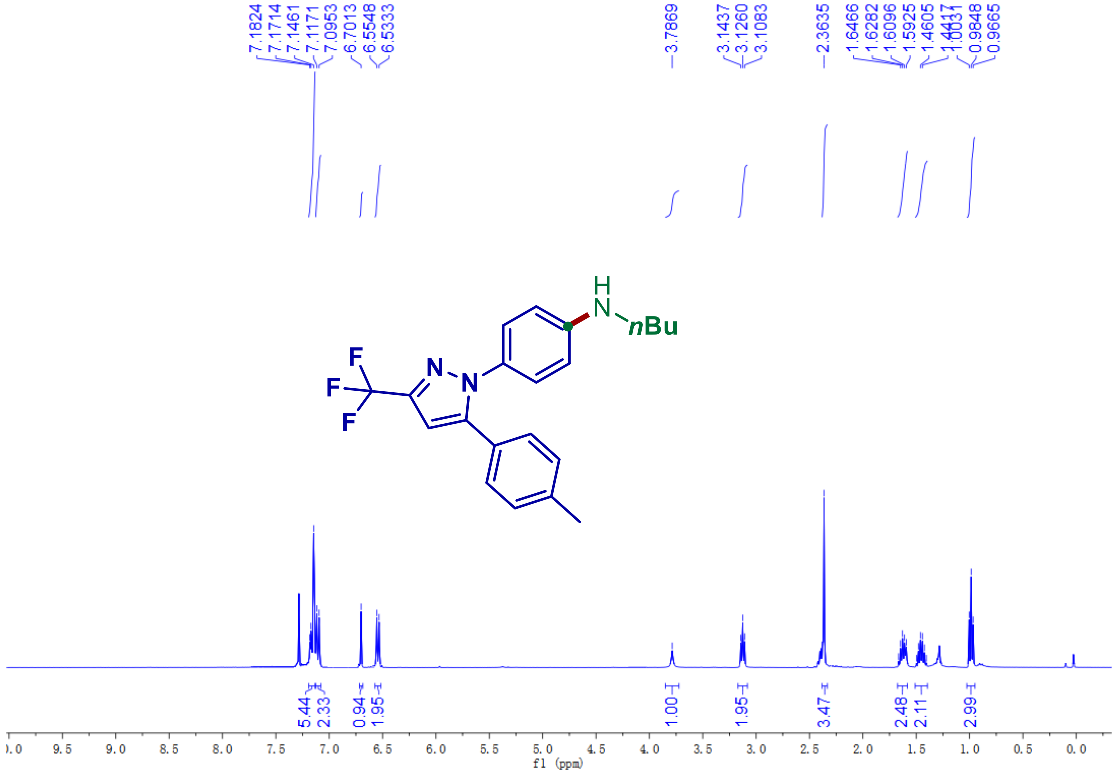

^{1}H NMR (400 MHz, $CDCl_3$) spectrum of compound 169

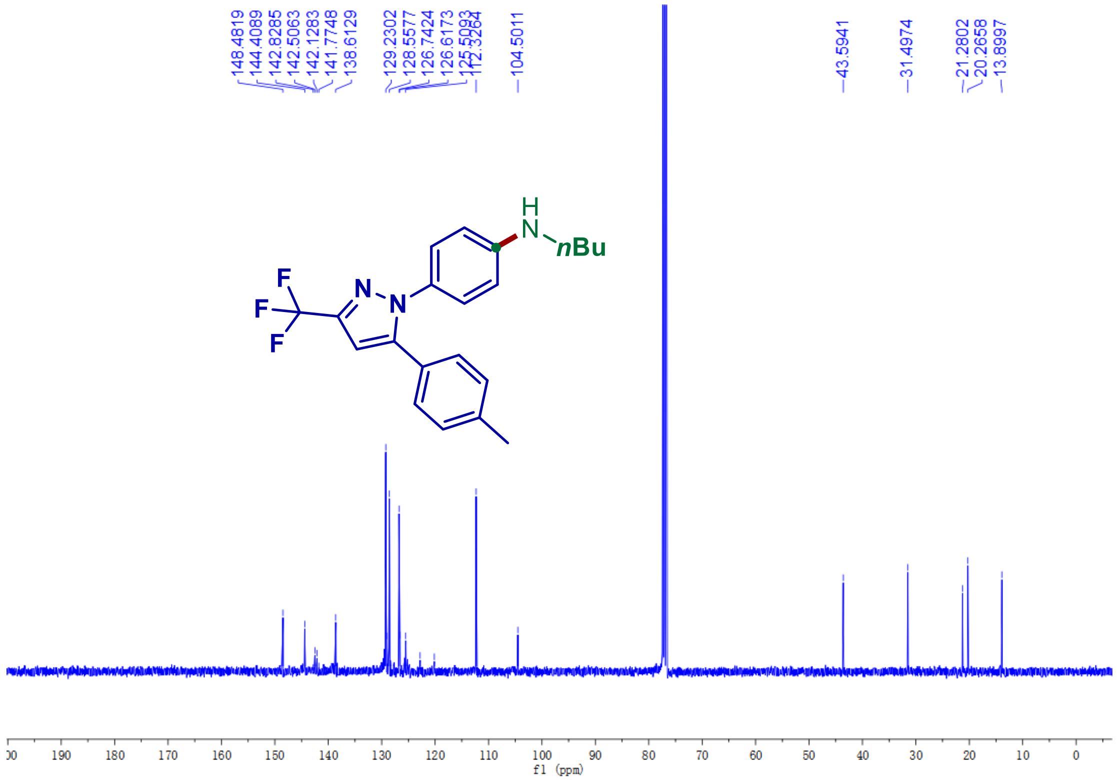

^{13}C NMR (100 MHz, $CDCl_3$) spectrum of compound 169

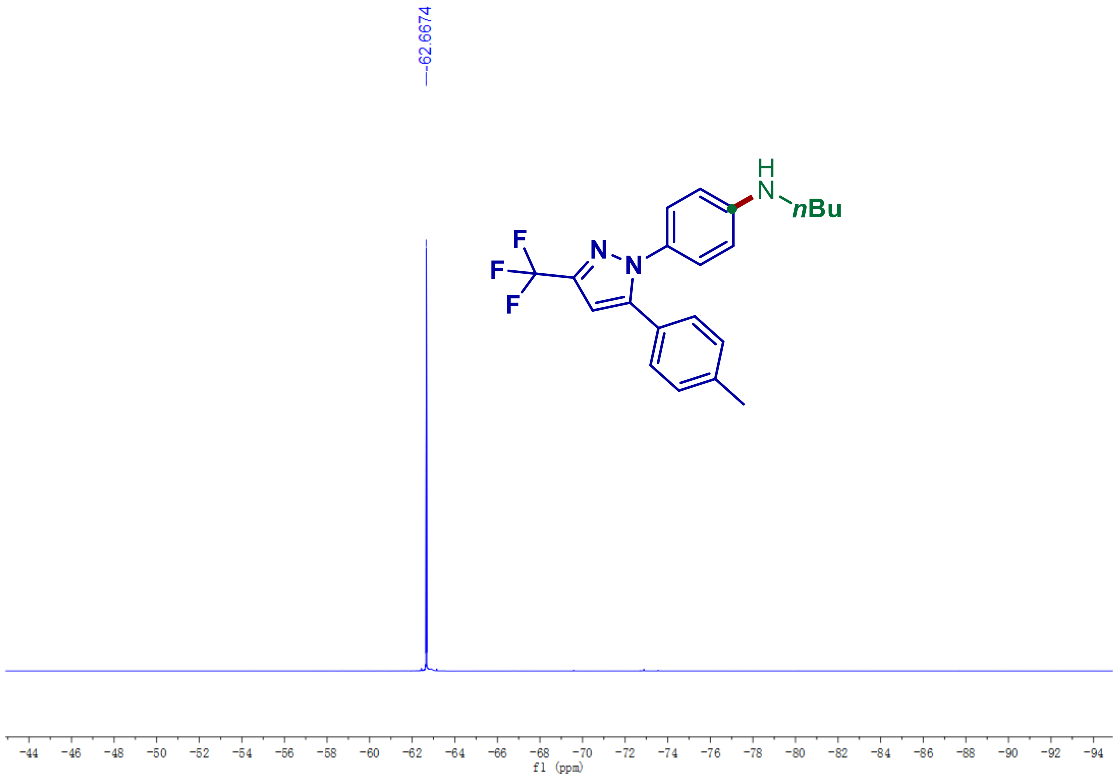

^{19}F NMR (376 MHz, $CDCl_3$) spectrum of compound 169

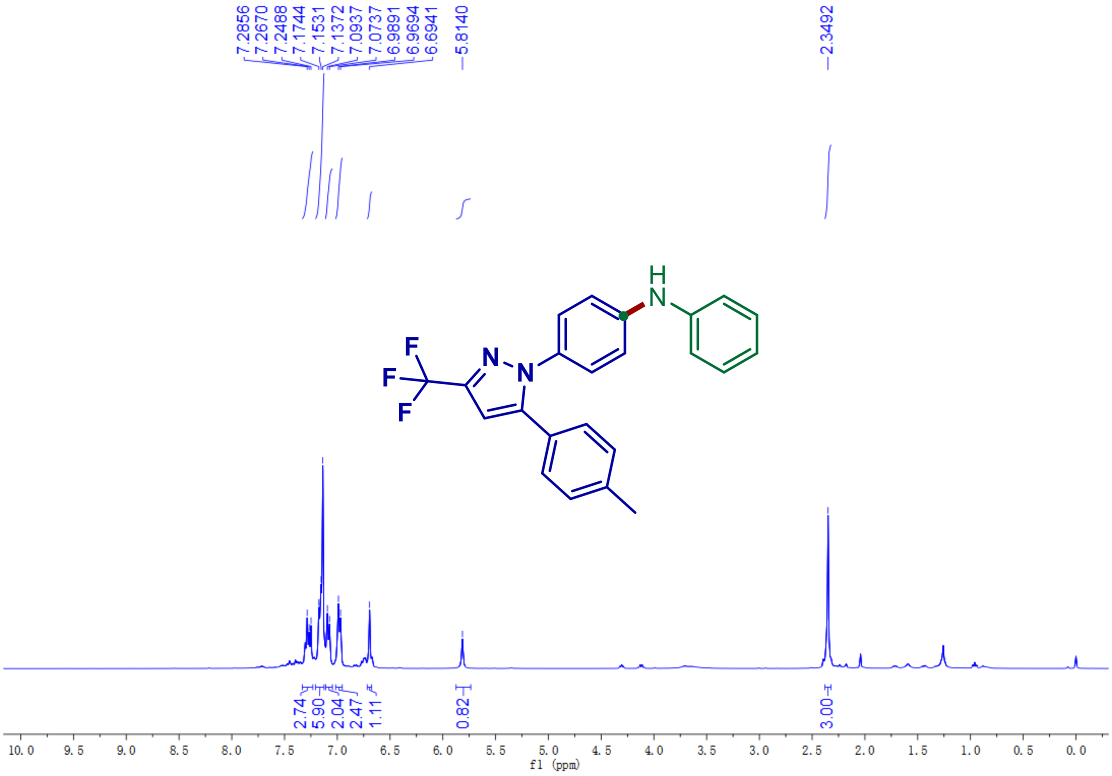

^1H NMR (400 MHz, $CDCl_3$) spectrum of compound 170

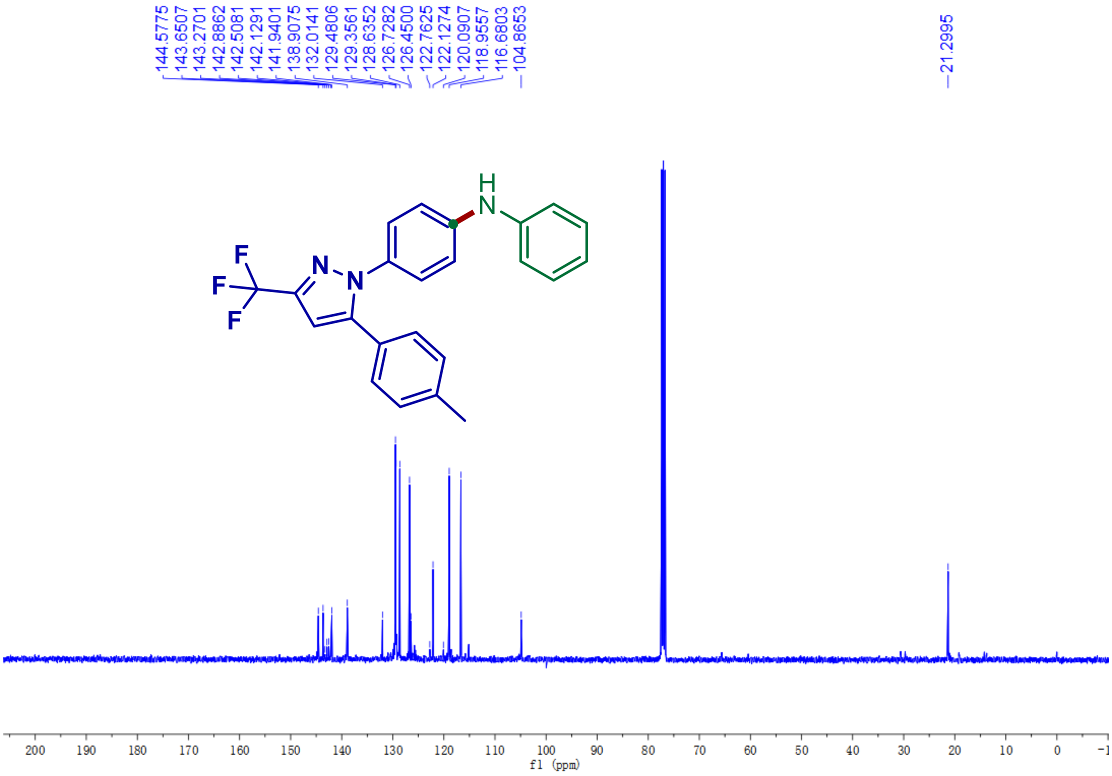

^{13}C NMR (100 MHz, $CDCl_3$) spectrum of compound 170

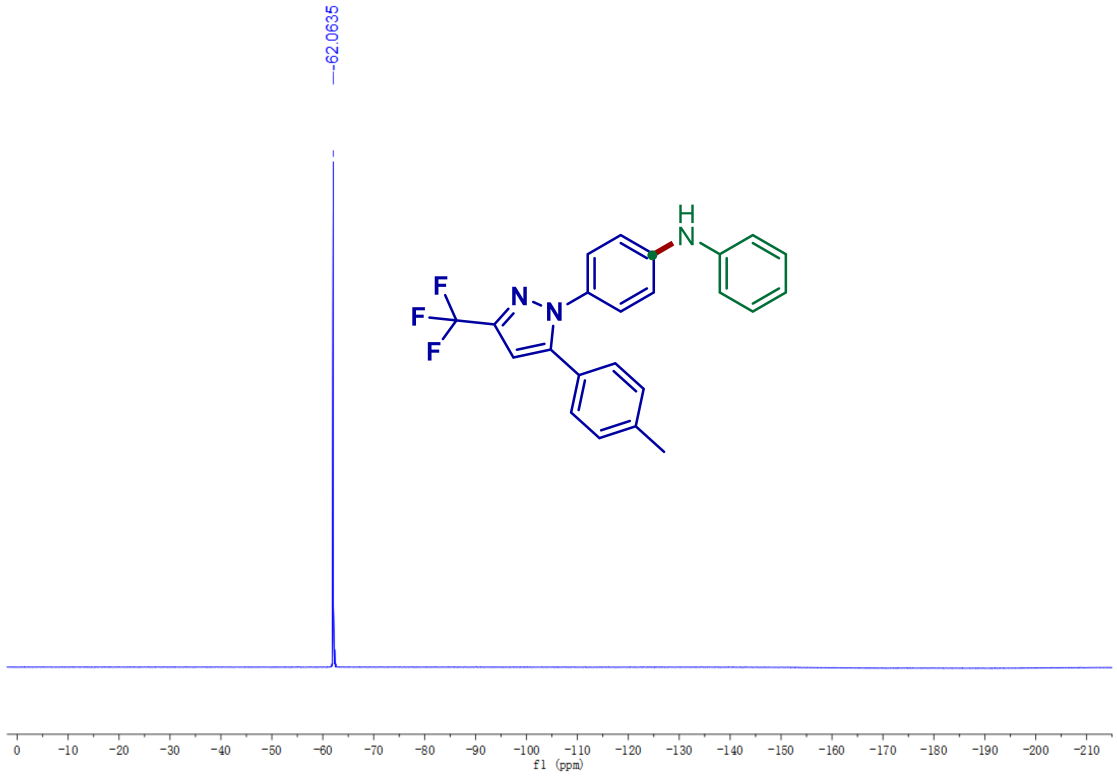

^{19}F NMR (376 MHz, $CDCl_3$) spectrum of compound 170

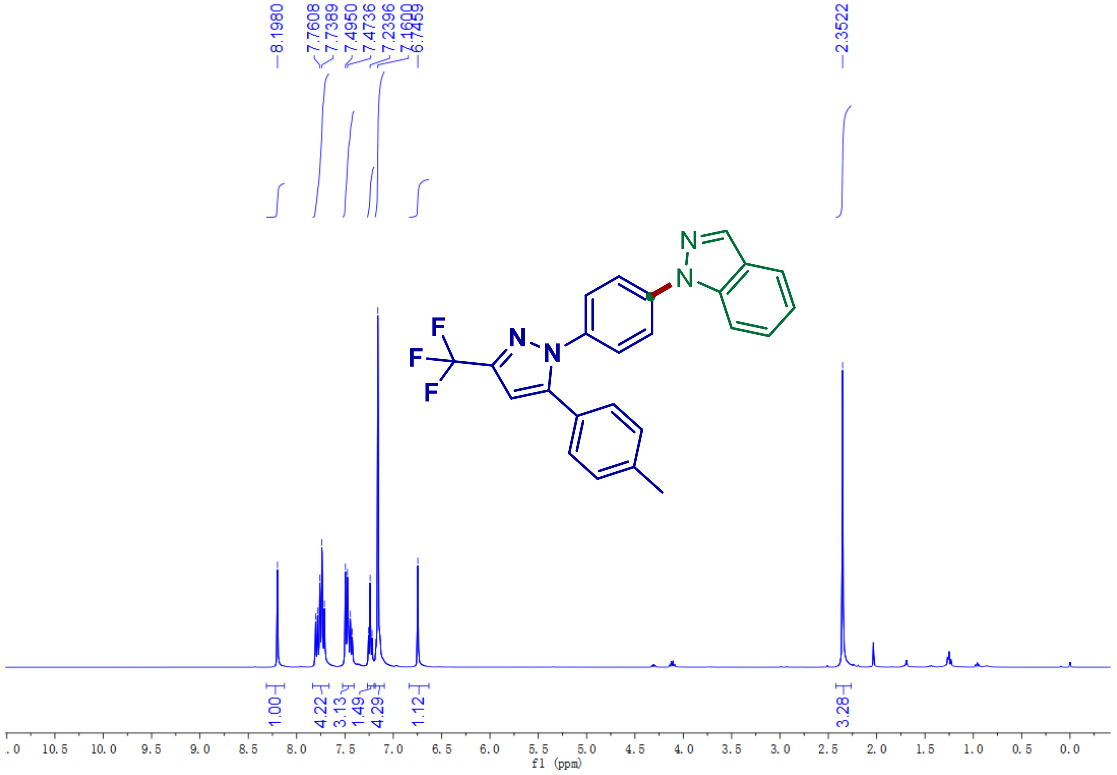

^{1}H NMR (400 MHz, $CDCl_3$) spectrum of compound 171

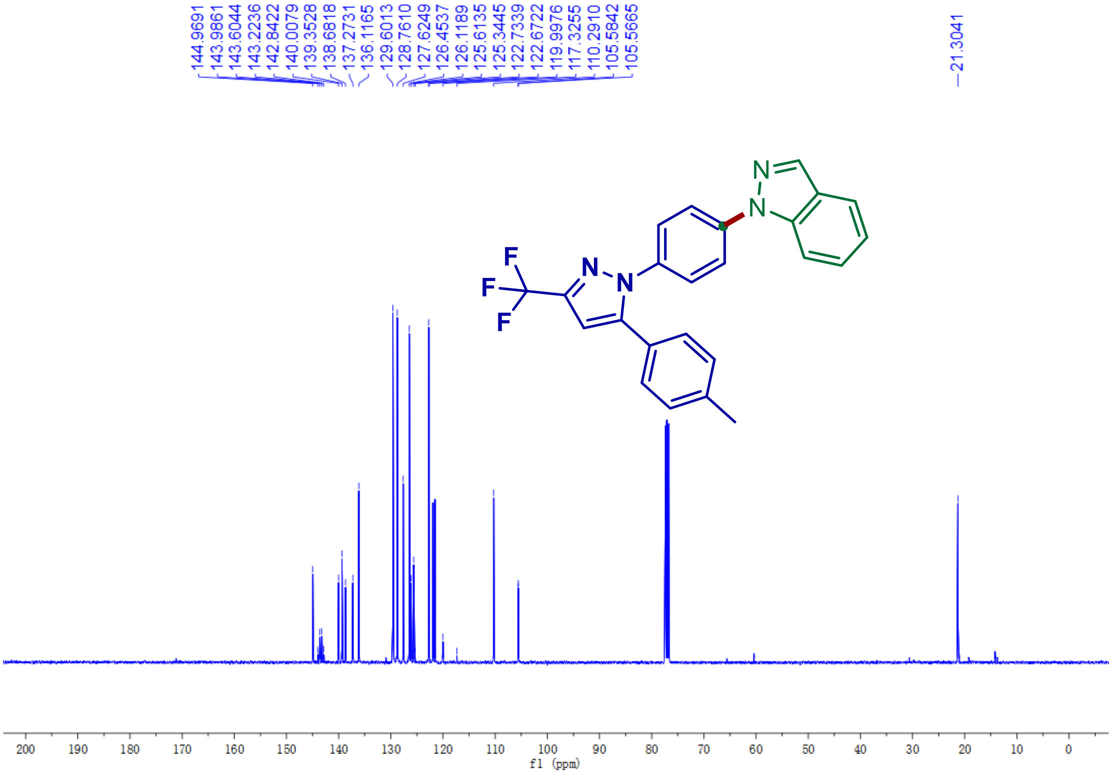

^{13}C NMR (100 MHz, $CDCl_3$) spectrum of compound 171

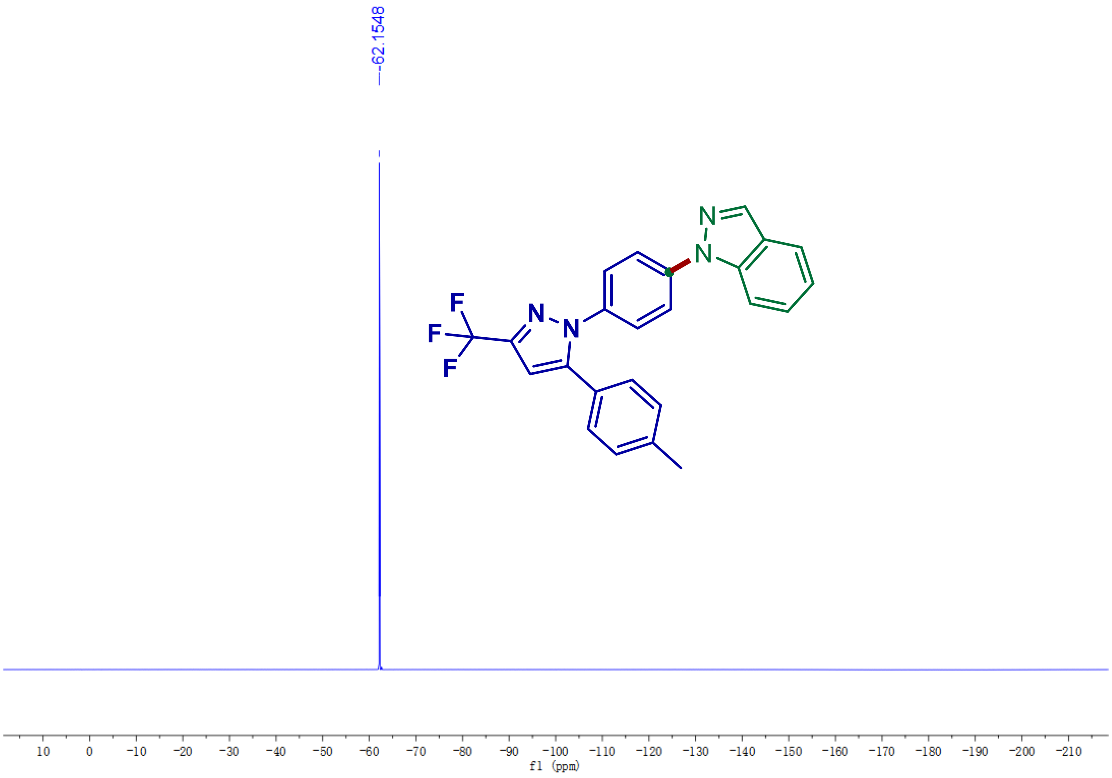

^{13}C NMR (100 MHz, $CDCl_3$) spectrum of compound 171

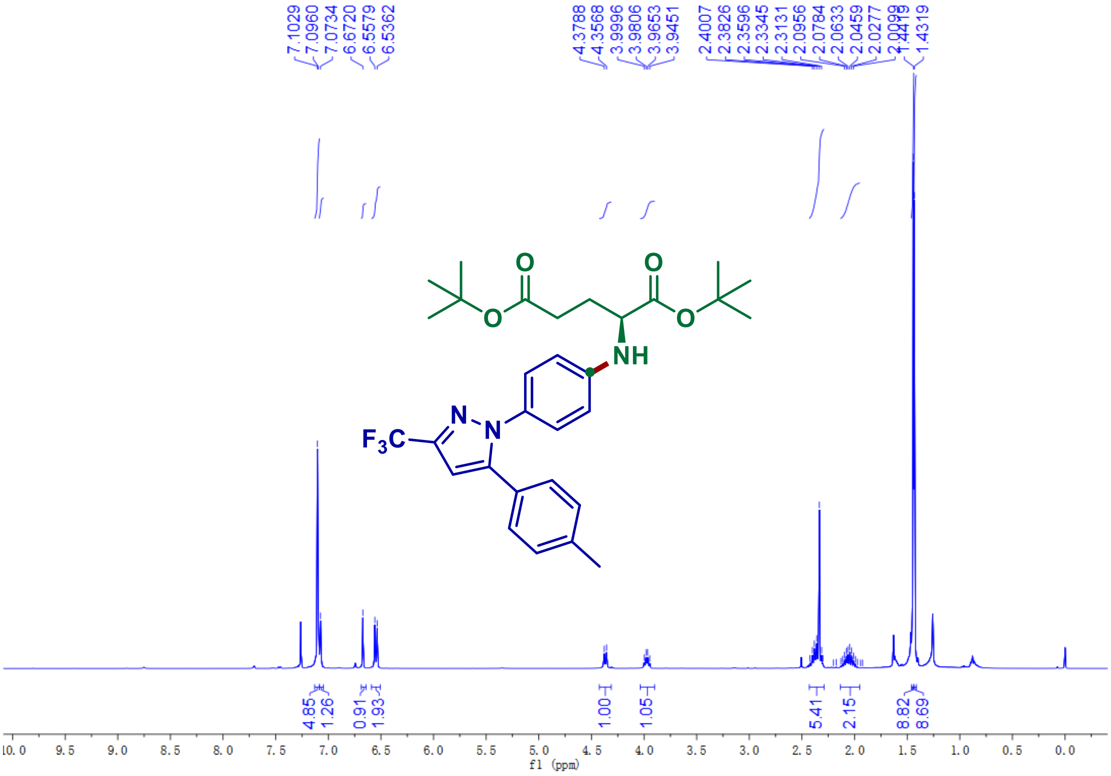

^{1}H NMR (400 MHz, $CDCl_3$) spectrum of compound 172

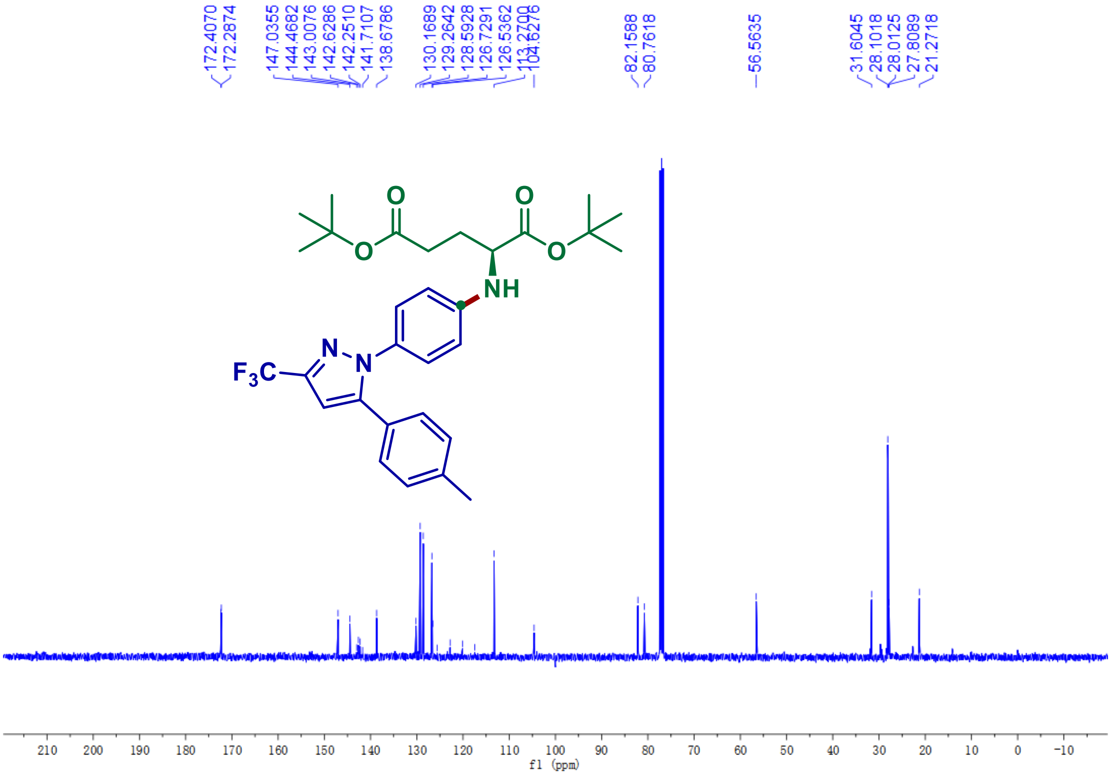

^{13}C NMR (100 MHz, $CDCl_3$) spectrum of compound 172

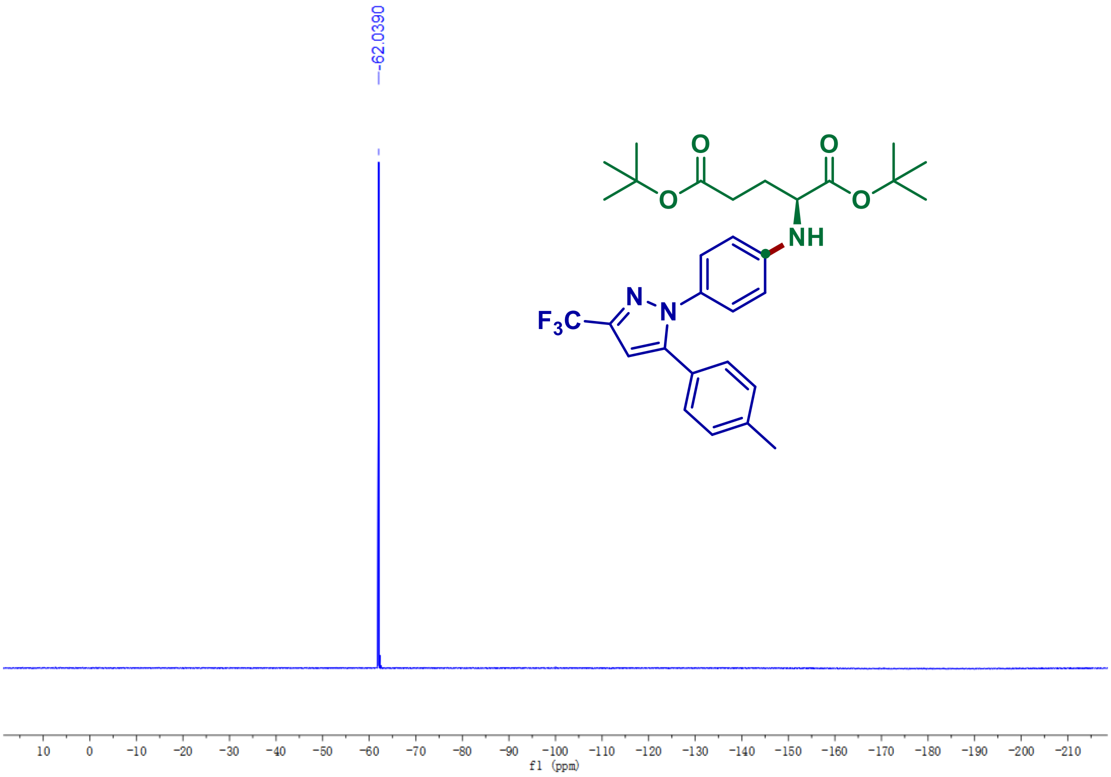

^{13}C NMR (100 MHz, CDCl$_3$) spectrum of compound 172

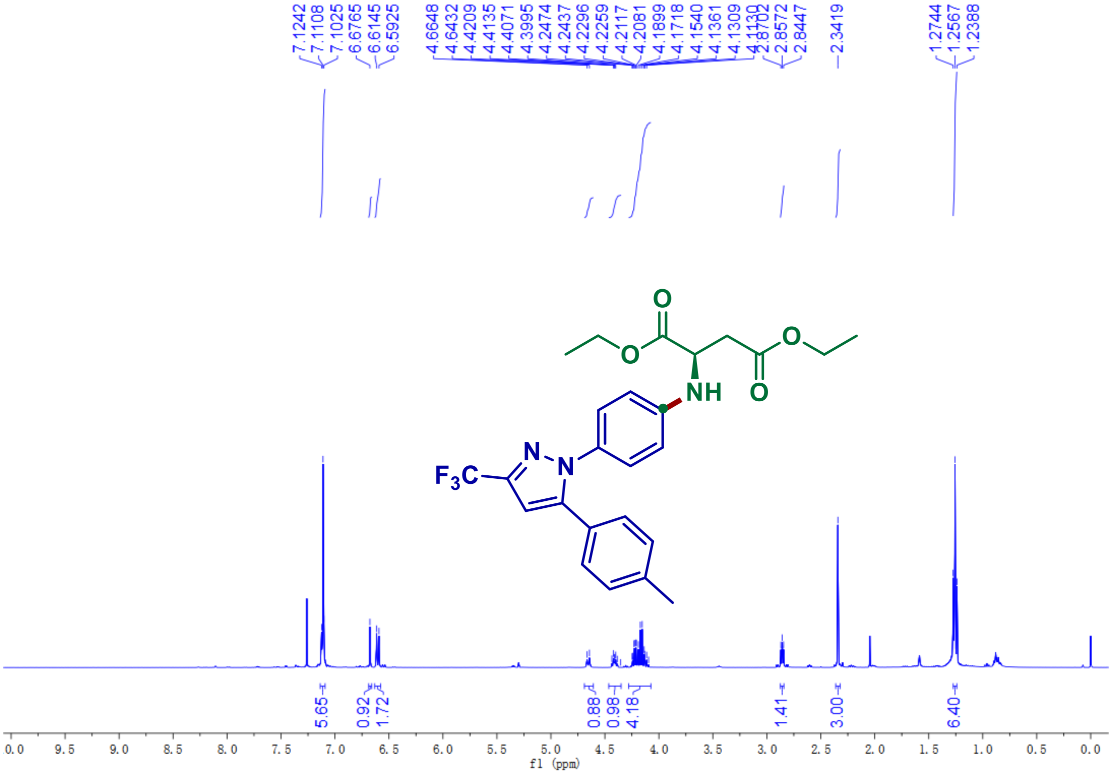

^{1}H NMR (400 MHz, CDCl$_3$) spectrum of compound 173

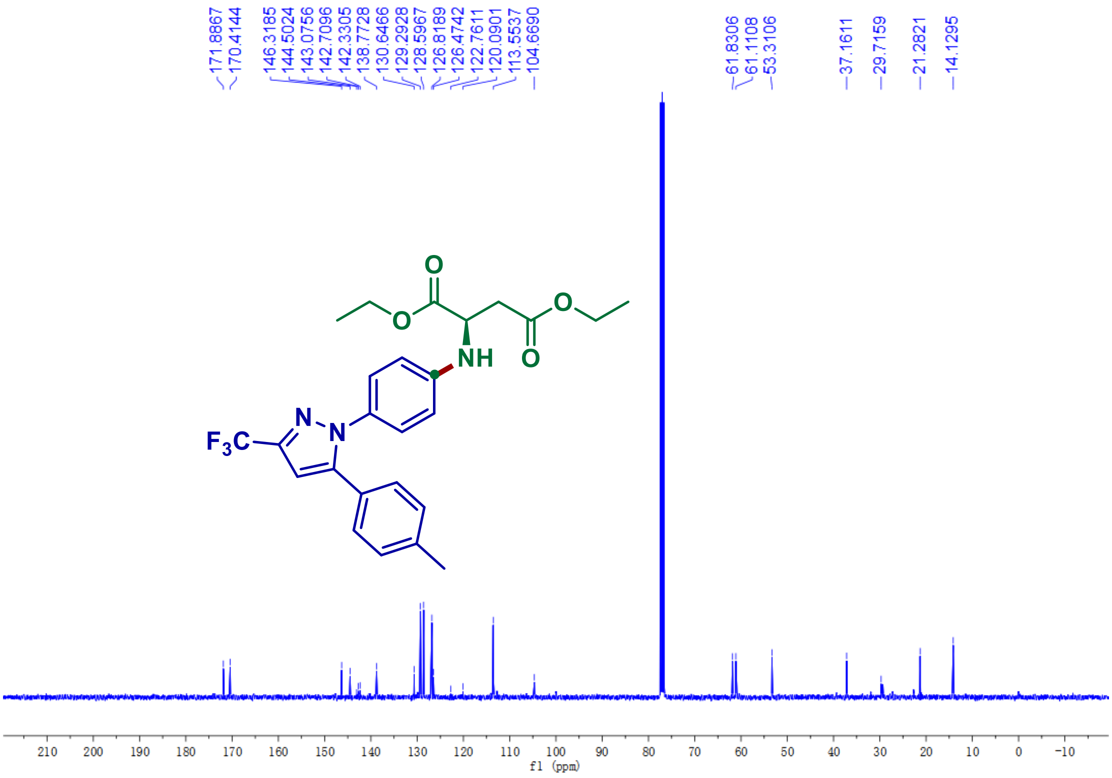

^{13}C NMR (100 MHz, CDCl$_3$) spectrum of compound 173

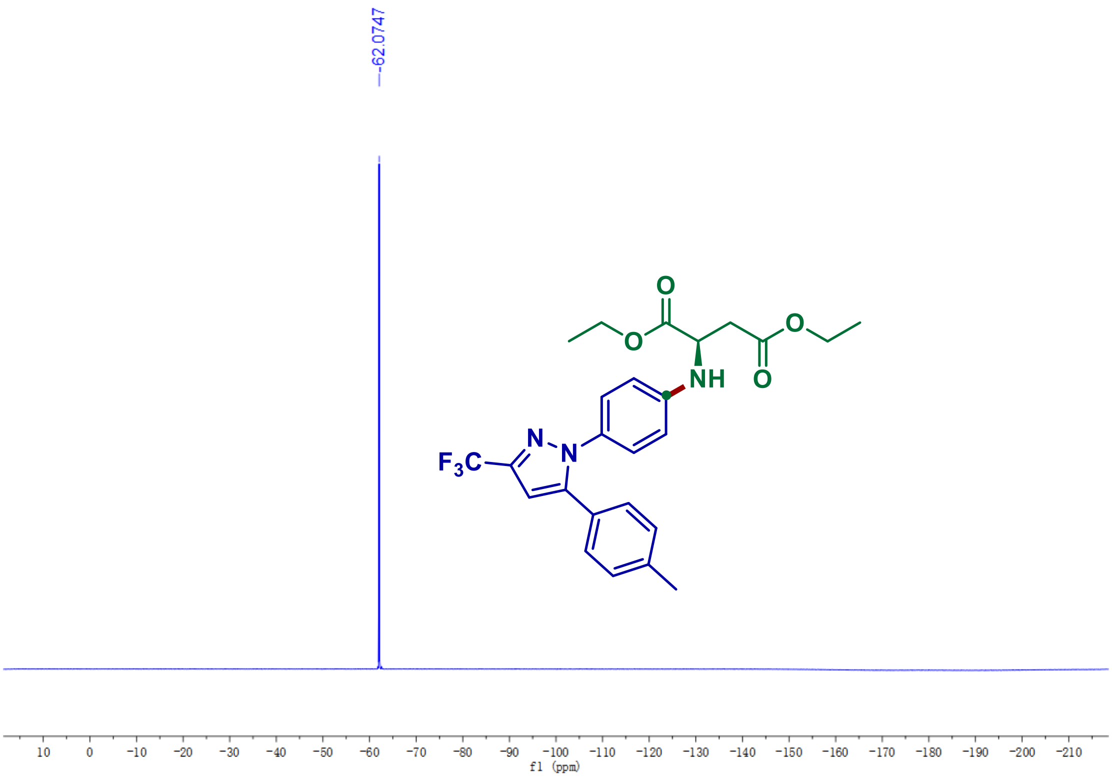

^{19}FNMR (376 MHz, CDCl$_3$) spectrum of compound 173
